# Phosphazenyl Phosphine Proton Sponges: Does the Proton-Chelating Effect Enhance Their Basicity?

**DOI:** 10.3390/ijms26115058

**Published:** 2025-05-24

**Authors:** Zoran Glasovac, Danijela Barić, Ines Despotović, Borislav Kovačević

**Affiliations:** 1 Division of Organic Chemistry and Biochemistry, Ruđer Bošković Institute, Bijenička c. 54, HR-10000 Zagreb, Croatia; zoran.glasovac@irb.hr; 2 Division of Physical Chemistry, Ruđer Bošković Institute, Bijenička c. 54, HR-10000 Zagreb, Croatia; danijela.baric@irb.hr (D.B.); ines.despotovic@irb.hr (I.D.)

**Keywords:** proton sponges, phosphazenyl phosphines, basicity, p*K*_a_, quantum chemical calculations

## Abstract

Gas-phase basicity and basicity in acetonitrile solvent were investigated for a series of proton sponges derived from phosphazenyl phosphines. A range of aromatic and aliphatic scaffolds bearing phosphazenyl phosphine substituents were employed to modulate the basicity of these compounds, primarily by varying the distance between the phosphazenyl phosphine units. These proton sponges were shown to be exceptionally strong organic bases, with p*K*_a_ values in acetonitrile reaching up to 42.0 units and gas-phase proton affinities (PA) up to 307.0 kcal mol^−1^. However, none exhibited higher basicity than the closely related phosphazenylphosphine systems, for which a p*K*_a_ value of 43.8 and PA value of 307.5 kcal mol^−1^ was previously reportedIt was found that the proton-chelating effect, typically defined as the difference in proton affinity between *bis-* and *mono*-substituted systems (ΔPA), moderately influences basicity. However, it was also established that ΔPA should not be attributed exclusively to the elimination of electron-pair repulsion and the formation of hydrogen bond upon protonation, as has been commonly assumed in most previous studies of proton sponges, but must also account for mesomeric and inductive effects, as well as dispersion interactions.

## 1. Introduction

**Scheme 1:**
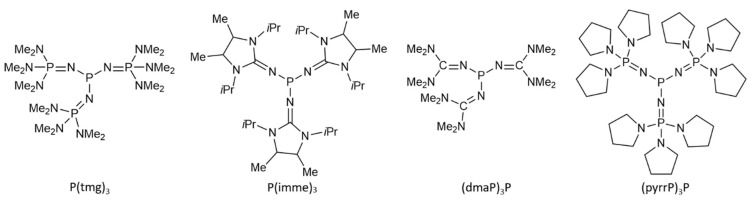
Structures of some recently synthesized superbasic phosphines.

While many organic (super)bases—such as guanidine derivatives, iminophosphoranes (phosphazenes), and vinamidines—are well established as strong Brønsted bases [1,2,3] with broad applications in organic synthesis [4,5], phosphines—excluding the Verkade base—have only in the past decade gained attention for their superbasic properties [6]. To recall, a superbase in the gas phase is defined as a compound with a gas-phase basicity (GB) greater than that of 1,8-bis(dimethylamino)naphthalene (DMAN), which has a GB of 237.8 kcal mol^−1^. In contrast, in solution, superbases are defined as compounds with p*K*_a_ values greater than that of the TMGN proton sponge, which has a p*K*_a_ of 25 in acetonitrile and 17 in tetrahydrofuran (THF) [7].The experimental and computational investigations of phosphine superbases, such as proazaphosphatrane (Verkade base) [8], tris(tetramethylguanidinyl) phosphine (P(tmg)_3_) [9], tris(imidazoline-2-ylidenamino) phosphine (P(imme)_3_) [10] and phosphazenyl phosphines (dmaP)_3_P and (pyrrP)_3_P [11] (Scheme 1), have highlighted their exceptional basicity, with some variants demonstrating p*K*_a_ values higher than those of traditional nitrogen-based superbases. Notably, imidazolidin-2-ylideneaminophosphines and phosphazenyl phosphines have been found to exhibit basicity comparable to or even surpassing that of well-established nitrogen superbases like Schwesinger’s P2- and P4- phosphazenes [12]. This emerging area of phosphine chemistry underscores the growing interest in exploring the basicity of phosphines as an essential feature in organic reaction mechanisms, particularly in the context of deprotonation steps, which are pivotal in many catalytic processes [6].

**Scheme 2:**
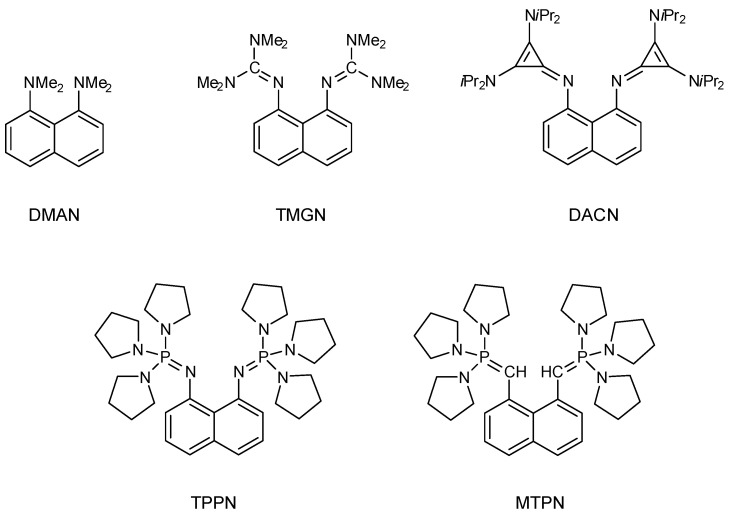
Structures of selected typical proton sponges.

In the design of new organic superbases, one of the factors contributing to their enhanced basicity is the proton-chelating effect (PCE) [13,14]. This effect was first observed in the DMAN molecule [14] and involves two primary mechanisms for basicity strengthening: the diminishing of electron-pair repulsion upon protonation and the formation of an intramolecular hydrogen bond in the conjugate acid. The proton-chelating effect has since been effectively utilized to further enhance the inherently high basicity of compounds such as guanidines [15,16], cyclopropenimines [17,18] and phosphazenes [13,19,20]. All these compounds are referred to as ‘proton sponges’ because of their structural similarity to the DMAN molecule. Some examples are presented in Scheme 2.

However, recent attempts to enhance the basicity of ylides, e.g., MTPN (Scheme 2), using the proton-chelating effect have led to conflicting conclusions. In a study by Saha et al. [21] it was concluded that the proton chelation effect (PCE) significantly contributes to the basicity of *bis*-ylides, primarily due to the strong stabilizing (–C^+^–H···C–) interaction in the protonated form of the ylide proton sponges. In contrast, a recent study by Kogel et al. [22] argued that PCE plays a negligible role in the basicity of similar compounds because (1) the (–C^+^–H···C–) interaction is very weak, and (2) the ylide’s lone pair is almost completely delocalized into the naphthalene backbone, which eliminates significant lone pair-electron repulsion in the neutral base and further decreases the potential for forming a –C^+^–H···C– hydrogen bond in the protonated base. However, it should be emphasized that in the study of Saha et al. an aliphatic carrier of ylide groups was used, whereas in the study of Kogel et al. an aromatic carrier (naphthalene) was utilized, so argument (2) for the absence of the –C^+^–H···C–bond is not applicable in the former study.

The proton-chelating effect in phosphine proton sponges has also been studied, although to a limited extent. In a study by Reiter et al. [23] experimental investigations of 1,8-di(phosphinyl)naphthalene revealed that P^+^–H···P hydrogen bonding is insignificant in this class of compounds. This conclusion was based on the crystal structures of the target compounds, though basicity was not specifically examined. However, it should be emphasized here that the interatomic distance imposed by the 1,8- substitution on naphthalene, which is 2.5 Å, is almost ideal for the formation of N^+^–H···N intramolecular hydrogen bonds [24], while, due to the substantially larger size of the phosphorus atom, this distance is too small for the formation of an optimal P^+^–H···P bond [25]. In a study by Haav et al. [26], p*K*_a_ measurements in acetonitrile solution were performed on several diphosphines, and it was concluded that there might be a weak intramolecular hydrogen bond in protonated diphosphines with an aromatic backbone and there is no intramolecular hydrogen bond in protonated diphosphines with an alkyl backbone. Since intramolecular hydrogen bonding is one of the two key factors in the proton-chelating effect, its potential absence suggests that the PCE in phosphine proton sponges could be small or negligible, particularly when compared to nitrogen-based proton sponges.

To address this question, we conducted a computational study on several compounds in which electron-rich phosphazenyl phosphines (PAPs), known for their exceptional basicity [11], were substituted onto various aromatic and aliphatic backbones. The use of various scaffolds as carriers for PAP groups aims to investigate the effect of the intergroup distance between phosphazenyl units on the basicity of phosphine proton sponges, as well as to assess the influence of aliphatic versus aromatic backbones on basicity, considering the possible delocalization of the electron pair of phosphine group(s) in the aromatic scaffold.

## 2. Results and Discussion

### 2.1. Hydrogen Bond in Phosphine Dimers

As mentioned in the introduction, one of the two main factors contributing to the proton-chelating effect is the formation of an intramolecular hydrogen bond in the conjugate base. It is well established that phosphines, unlike amines, do not form strong hydrogen bonds. This is primarily because, in contrast to the N^+^–H bond, the P^+^–H bond exhibits lower polarity due to the lower electronegativity of phosphorus relative to nitrogen. This likely explains why the P^+^–H···P hydrogen bond has not been as extensively studied, particularly in comparison to the N–H···N, N–H···O, O–H···O and other strong hydrogen bonds. In one of the few studies on this topic, using high-level ab initio methods, the authors investigated the P^+^–H···P hydrogen bond in complexes between protonated and neutral phosphine moieties, but only for simple phosphines such as PH_3_, CH_3_PH_2_, etc. [25]. They found that a linear hydrogen bond exists in some of these complexes, but its strength is relatively weak. For example, it was calculated that the binding energy in the CH_3_PH_3_^+^···PH_3_ complex is 7.7 kcal mol^−1^, whereas in the PH_4_^+^···PH_3_ complex, the binding energy is 9.3 kcal mol^−1^. This is much weaker than in the corresponding amines, where hydrogen bond strength can reach up to 23 kcal mol^−1^ [27]. However, according to classification by stabilizing interaction strength, P^+^–H···P hydrogen bonds can still be categorized as a moderately strong (*E* = 4–15 kcal mol^−1^) [28]. Given that the phosphazenyl group as a substituent in phosphines dramatically increases their inherent basicity, i.e., their gas basicity (GB) and proton affinity (PA) [11], and considering that the strength of the hydrogen bond depends on the PA of the participating species [29], the question arises as to how phosphazenyl substitution influence hydrogen bond strength between neutral and protonated phosphazenyl phosphine. H-bond strength obviously depends on the hydrogen bond accepting ability of the neutral phosphine and the hydrogen bond donating ability of the protonated phosphine. The simplest way to evaluate how the phosphazenyl group affects P^+^–H···P hydrogen bond strength is to calculate the interaction energy in dimers formed from neutral and protonated phosphazenyl phosphines, attributing this interaction to the hydrogen bond energy [25]. However, phosphazenyl phosphines are bulky species, and their bulkiness may sterically hinder or significantly reduce the likelihood of hydrogen-bond interactions within such a dimer potentially leading to misleading conclusions. It should be noted, however, that when phosphazenyl phosphine substituents are placed at positions 1 and 8 on the naphthalene backbone (or on a similar system), the phosphazenyl phosphine groups are arranged in a configuration where hydrogen-bond interactions could occur. Any potential steric repulsion caused by bulkiness of phosphazenyl groups is ‘absorbed’ by the molecular strain, which then typically manifests as a distortion of the aromatic backbone [19]. To better understand the behavior of phosphazenyl phosphines (along with other superbasic phosphines presented in Scheme 1) in H-bond formation, we separately examined their ability to function as hydrogen-bond acceptors. Additionally, we investigated their protonated forms to determine their potential to act as hydrogen-bond donors. This involved calculating the stability of the complexes formed between these phosphines and small molecules that could act as hydrogen-bond donors or acceptors.

To evaluate the H-bond acceptor ability of phosphines, hydrogen fluoride (HF) was selected as the H-bond donor. The interaction energy between the HF molecule and the neutral form of each target phosphine was determined by calculating the enthalpy difference between the HF-phosphine dimer and the sum of the enthalpies of the isolated species. It should be noted that studied phosphines (Scheme 1) can form hydrogen bonds with HF via either the phosphorus or the nitrogen atom of the substituent.

The H-bond donating ability of the protonated phosphines was tested by the calculation of interaction energies for dimers formed between protonated phosphines and ammonia (NH_3_), which served as the H-bond acceptor. For comparative purposes, the interaction energies for dimers consisting of trimethylamine with HF, as well as protonated trimethylamine with NH_3_, were also computed. The interaction energies are presented in Table 1.

Interaction energies between HF and neutral phosphines show that the H-bond acceptor strength of phosphines increases markedly with the introduction of progressively stronger electron-donating substituents. For example, substitution of hydrogen atoms in PH_3_ with dimethylaminophosphazenyl groups to form (dmaP)_3_P increases the interaction energy from 4.4 kcal mol^−1^ to 18.4 kcal mol^−1^, which means that the phosphazenyl group on phosphorus significantly enhances the H-bond acceptor properties of the resulting phosphine. In the case of (pyrrP)_3_P, which features the highly electron-donating pyrrolidinophosphazenyl substituent, the interaction energy for the phosphorus atom could not be determined due to an intermolecular proton transfer from HF to the phosphine, resulting in the formation of a zwitterionic dimer. For reference, trimethylamine N(CH_3_)_3_ exhibits an interaction energy of 14.9 kcal mol^−1^. These results indicate that phosphines bearing strong electron-donating groups can display H-bond acceptor strength comparable to those of simple amines. It should be noted, however, that interactions between HF and nitrogen atom of substituents in compounds depicted in Scheme 1 are even higher than those with the phosphorus atom. Regarding the H-bond donating ability of protonated phosphines, it generally decreases with the substitution by stronger electron-donating groups. Namely, protonated trimethylphosphine has an interaction energy of 10.3 kcal mol^−1^ with NH_3_ molecule, whereas in (dma)P_3_P, this energy drops to only 3.3 kcal mol^−1,^ which is only a fifth of the value obtained for protonated trimethylamine. This could be explained by stronger charge delocalization in these electron-rich phosphines, which decreases the positive charge on the hydrogen atom in the P^+^–H bond. Given that substituting strong electron-donating groups on phosphorus atoms simultaneously enhances electron-accepting character and weakens electron-donating properties, we can conclude that in the case of phosphazenyl phosphine proton sponges, the strength of the P^+^–H···P intramolecular hydrogen bond would most likely be comparable to the strength of the hydrogen bond in simple phosphines such as PH_3_ and P(CH_3_)_3_ [25].

### 2.2. Proton-Chelating Effect in Bisphosphazenyl Phosphines

**Figure 1:**
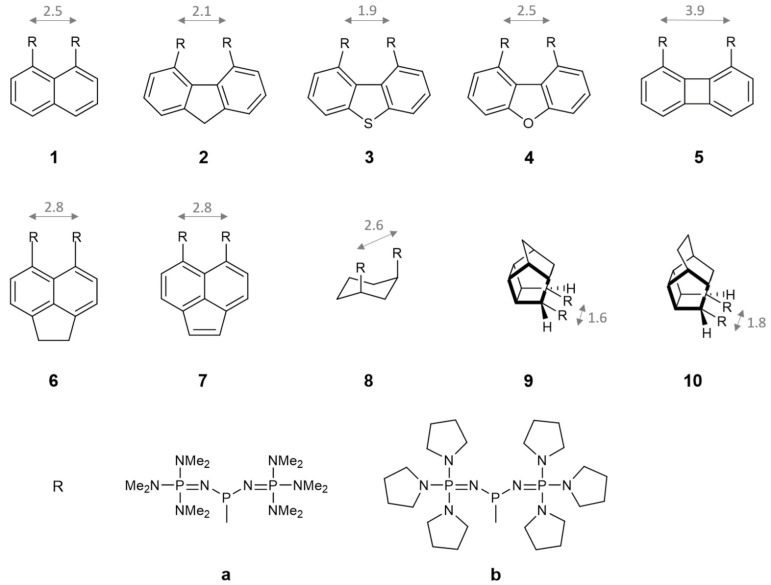
Schematic representation of *bis*-phosphines **1aa**–**10bb** computationally investigated for their basicity. The distances between the R groups (in Å) are given for the aromatic/aliphatic system when R = H, but the C–H bond distance is specified as the average P–C bond length, which in this case is 1.85 Å.

To address the question posed in the title—whether the proton-chelating effect increases the basicity of bis(phosphazenyl)phosphines—we investigated the basicity of the compounds shown in Figure 1. Various backbones (or carriers) to which the phosphazenyl phosphines (PAP) groups are attached were utilized to modulate the distance between the PAP groups (see Figure 1), which, in turn, influences the repulsive interactions of electron pairs on phosphorus within neutral molecular systems. This adjustment also allows us to either approach or deviate from the optimal distance (which is approximately 4 Å) necessary for forming a P^+^–H···P hydrogen bond in the protonated form of these molecules systems, as well as approach or deviate from the ideal P^+^–H···P bond angle of 180° [25]. A distance shorter than 4 Å between the PAP groups induces greater strain in neutral systems but also moves the system further from the optimal distance required for intramolecular P^+^–H···P hydrogen bond formation. The optimal distance for the latter is present only in biphenylene backbone. However, it should be noted from the calculations presented in Table 1 that the nitrogen atom in phosphazenyl substituents is a better hydrogen-bond acceptor than the phosphorus atom in phosphines. Therefore, it is possible that in systems where the distance between PAP groups is shorter than the optimal distance for P^+^–H···P bond formation, P^+^–H···N bonds may form instead. As can be seen in Figure 1, the backbones used in this study are mostly aromatic systems with three exceptions (**8**, **9** and **10**). Pentacyclic moieties **9** and **10** are recently utilized in the investigation of C^+^–H···C bond in protonated *bis*-ylides and proved to be superior for hydrogen bond formation in these systems [21]. These aliphatic backbones have a twofold effect: (1) due to their intrinsic geometry, they potentially enable better P^+^–H···P (or P^+^–H···N) interaction, facilitating the formation of a nearly linear H-bond [21], and (2) they prevent the possible delocalization of the electron pair on phosphorus into the backbone, an effect that has been found to decrease basicity as well as the IHB strength in *bis*-ylides [22] and *bis*-phosphazenes attached to the aromatic scaffold [11,19,20].

**Scheme 3:**
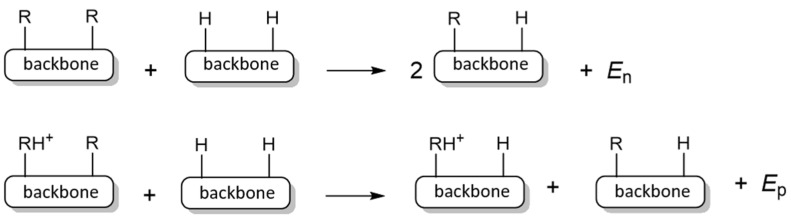
Homodesmic reactions employed to decompose the ΔPA into contributions from the neutral and protonated forms of the proton sponges.

Data presented in Table 2 reveal that among 20 *bis*-phosphine proton sponges studied, four exhibit proton affinity higher than 300 kcal mol^−1^, classifying them as hyperbases [30]. It should be noted, however, that none of them reached or exceeded the proton affinity (PA) and gas-phase basicity (GB) values of the corresponding trisubstituted phosphazenyl phosphines (dmaP)_3_P and (pyrrP)_3_P examined recently in the study by Sundermeyer et al. [11]. Specifically, (dmaP)_3_P has PA and GB values of 297.4 and 291.3 kcal mol^−1^, respectively, while (pyrrP)_3_P has PA and GB values of 307.5 and 300.2 kcal mol^−1^, correspondingly [11]. Interestingly, systems **5aa** and **5bb**, which exhibit the optimal P···P distance for hydrogen bond formation, have among the lowest PA and GB values. This suggests that the P^+^–H···P hydrogen bond may not significantly contribute to the basicity of this type of compound which is confirmed by AIM analysis (see below). Systems **8aa** and **8bb** with PA/GB values of 296.8/288.6 and 307.0/296.3 kcal mol^−1^, respectively, approach the PA/GB values of the corresponding phosphines (dmaP)_3_P and (pyrrP)_3_P, which equals 297.4/291.3 and 307.5/300.2 kcal mol^−1^, correspondingly, although they remain slightly lower. Based on these observations, it might be concluded that, in phosphazenyl phosphine proton sponges—unlike those based on phosphazenes, cyclopropenimines, guanidines, or amines—the intrinsic (gas-phase) basicity does not increase by placing two basic moieties in close proximity. However, in the latter-mentioned proton sponges, the increase in basicity due to interactions between two basic moieties is typically compared against a basic moiety where the substituent on the basic nitrogen atom is hydrogen (e.g., P1–H phosphazene, 1,1,3,3-tetramethylguanidine, 2,3-bis(dicyclohexylamino)cyclopropenimine)-3H, dimethylamine, etc). Consequently, a proper comparison for phosphazenyl phosphine sponges should be made with di-phosphazenyl phosphines (dma)_2_HP and (pyrr)_2_HP, having PA/GB values of 276.6/269.6 kcal mol^−1^ and 283.0/276.2 kcal mol^−1^, respectively. When comparing the PA/GB values of all 20 studied systems (**1aa** to **10bb**) against these corresponding phosphines (dma)_2_HP and (pyrr)_2_HP—which will be hereafter referred to as the core phosphine systems—a clear increase in intrinsic basicity emerges, with differences in PA/GB values ranging between 15 and 20 kcal mol^−1^. Another important observation from the data presented in Table 2 and Figure 1 is the absence of a direct correlation between the proton affinity (PA) or gas-phase basicity (GB) values and the distance between the phosphazenyl substituents. This suggests that structural proximity alone is not sufficient to reliably predict the basicity of phosphine proton sponges. Table 2 also includes PA/GB values for monophosphine derivatives, labeled **1a** through **10b**. These derivatives arise from the replacement of one phosphazenyl phosphine fragment in the *bis*-phosphine system with a hydrogen atom. Comparing these monophosphine derivatives with the corresponding core phosphines [(dma)_2_HP and (pyrr)_2_HP] offers valuable insight into how aromatic and aliphatic backbones influence the basicity of the core phosphine structure. Analysis of the PA and GB values indicates that substituting the hydrogen atom in (dma)_2_HP and (pyrr)_2_HP phosphines with either an aliphatic or aromatic moiety leads to an increase in the basicity of the core phosphine framework. This behavior contrasts sharply with that observed in phosphazene, guanidine, cyclopropenimine, and ylide systems, where incorporation of an aromatic backbone typically results in a significant decrease in basicity [15,16,17,18,19,20]. Interestingly, no significant difference is observed between aliphatic and aromatic backbones in their influence on the PA and GB values. This suggests that, in phosphines, the lone pair on the phosphorus atom does not substantially delocalize into the aromatic system—a delocalization effect known to diminish basicity in phosphazenes, guanidines, and ylides [19,20,22]. Examining the ΔPA values presented in Table 2, which represent differences in PA between *mono*- and *bis*-phosphines, typically attributed to proton-chelating effect, provides further insights. The ΔPA values range from 8.8 kcal mol^−1^ in **5bb** to 19.2 kcal mol^−1^ in **3bb**, indicating a potentially substantial proton-chelating effect in some of the studied systems. However, in nitrogen-based proton sponges such as TPPN and P2-TPPN, the ΔPA value is typically higher, reaching up to 28 kcal mol^−1^. Employing the homodesmic reactions shown in Scheme 3, these ΔPA values can be decomposed into contributions arising from the neutral base, *E*_n_ (typically attributed to lone pair repulsion) and from the conjugate acid, *E*_p_ (typically attributed to the intramolecular hydrogen bonding.

**Figure 2:**
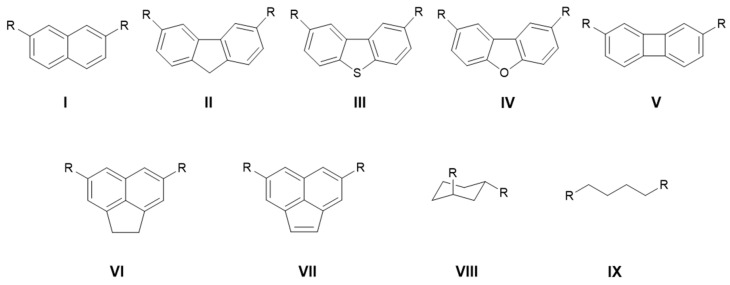
*Bis*-phosphazenyl phosphines, where proton sponge-like interactions between substituents are not present.

These separated effects are also summarized in Table 2. Inspecting these values reveals a classical behavior consistent with previous studies of proton sponges for most systems. Namely, *E*_n_ is a positive number, indicating destabilization of the neutral base, while *E*p values are negative, indicating stabilization of the protonated base. The most notable exceptions are systems **5aa** and **5bb,** where *E*_n_ values are negative. This result indicates not only the absence of destabilization but also the presence of a stabilizing interaction arising from the interplay between the phosphazenyl groups. One reason for this behavior could be the larger distance between the phosphazenyl groups in these systems, reducing the likelihood of repulsive interactions. Nevertheless, the negative *E*_n_ value also suggests additional stabilization, which can hardly be explained by the mesomeric effect. Interestingly, negative numbers of *E*_n_ are also observed in some of the aliphatic systems, despite smaller distances between phosphazenyl groups. Notably, the *E*_n_ values are more negative in systems bearing bulky pyrrolidyl substituents, where greater steric repulsion between groups would typically be expected, compared to systems with -NMe_2_ substituents. This observation may indicate the presence of stabilizing interactions probably arising from dispersion forces, which generally become more significant in larger molecular systems. To substantiate this hypothesis, we performed Hartree–Fock plus London dispersion (HFLD) calculations [31] for both the neutral and protonated forms of molecules **5bb** and **5aa**. Molecule **5bb** was specifically chosen for its most negative *E*n and *E*p values among the studied systems, while **5aa** was included for comparison. Details of the computational methodology are provided in the Computational Details section below. The results reveal a notable dispersion interaction in the neutral form of **5bb**, contributing 24.5 kcal mol^−1^, which increases to 29.5 kcal mol^−1^ upon protonation. In contrast, the corresponding values for **5aa** are lower—16.4 and 17.6 kcal mol^−1^—indicating weaker dispersion interactions, as previously proposed. Even weaker dispersion interaction is present in model system where phosphazenyl group on phosphorus are replaced by dimethylamino groups. For this model system, obtained values are 3.9 and 4.1 for neutral and protonated form, respectively. It is important to note that the HFLD calculations were carried out using a computational model different from that employed for the results presented in Table 2; thus, the calculated interaction energies are not directly comparable. The primary purpose of this HFLD analysis was to confirm the existence of significant dispersion interactions between the phosphazenyl phosphine substituents. These findings point toward a complex interaction pattern between phosphazenyl phosphine groups in neutral bases, which cannot be explained exclusively by electron-pair repulsion. Since the energies obtained from the HFLD calculations are not directly comparable to those in Table 2, it is challenging to quantitatively distinguish between the destabilizing effect of electron-pair repulsion and the stabilizing influence of dispersion forces. Conversely, *E*_p_ values are negative, confirming the presence of stabilizing interactions in all proton sponges. The *E*_p_ values go up to 19.3 kcal mol^−1^. Given that previous results have established the expected energy contribution from forming the P^+^–H···P bond, it would be incorrect to attribute the entire stabilization energy observed here solely to this hydrogen-bonding interaction in the protonated systems. Further, a recent study on ylide proton sponges demonstrated that the observed ΔPA should not be attributed only to the proton-chelating effect. A comparison of 1,8- and 2,7-ylide substitutions on the naphthalene moiety revealed that the ΔPA values, ranging between 8 and 14 kcal mol^−1^, can be fully explained by the mesomeric effect, as nearly identical ΔPA values were found for both substitution patterns [22]. Therefore, we performed additional calculations on systems in which phosphazenyl phosphine substituents were positioned on the backbones in locations presumed to lack direct (through-space) interactions between them (Figure 2). For the cage *bis*-phosphines **9aa-10bb**, it was not possible to position the substituents in a manner analogous to the aromatic counterparts. Due to the bulkiness of backbones 9 and 10, it was also not feasible to construct a conformation analogous to **VIIIa**/**VIIIb**, where we considered the axial-equatorial orientation of the substituents. Therefore, the backbones of compounds **9aa** and **10bb** were modeled using a butyl chain, as the phosphazenyl phosphine groups in both molecules are separated by the same number of carbon atoms. It is important to note that in these systems with aliphatic backbones, the mesomeric effect is absent, while inductive effects are present. PA and GB values for systems **I**–**IX** are presented in Table 3. A comparison of the PA values between systems **1aa**–**9bb** and **Iaa**–**Ibb** reveals that the latter are approximately 4–13 kcal mol^−1^ lower. Assuming that the mesomeric and inductive effect in systems **1aa**–**10bb** is the same as in systems **Iaa**–**IXbb**, only the difference in PA between **1aa**–**9bb** and **Iaa**–**Ibb** should be attributed to the proton-chelating effect. These values range from 1.1 kcal mol^−1^ for **4bb** to 14.8 kcal mol^−1^ for **8bb**. However, even in systems **Iaa**–**IXbb**, the energies obtained from the homodesmotic reaction suggest the presence of a stabilizing interaction in the neutral form, which appears to be more pronounced in systems bearing the pyrrolidyl substituent (**bb** series of proton sponges). Attributing this stabilizing effect to a mesomeric contribution would be counterintuitive; therefore, it is more likely that it arises from dispersion forces as it was discussed for systems **5aa** and **5bb**. Although the substituents in systems **Iaa**–**IXbb** are spatially more distant compared to those in systems **1aa**–**10bb**, they still, in some cases, engage in mutual interaction, as evidenced by the case of molecule **IIIbb** (Figure S1). For this system, HFLD calculations reveal substantial dispersion interactions of 10.3 and 11.2 kcal mol^−1^ for the neutral and protonated forms, respectively.

### 2.3. Basicity in Acetonitrile

Although the basicity in solution generally mirrors that in the gas phase to some extent, the correlation is not fully satisfactory. This is particularly evident in systems such as proton sponges, where the basic site is sterically shielded, limiting solvent accessibility. Therefore, we calculated the p*K*_a_ values of phosphine proton sponges **1aa**–**10bb** using two computational models: (i) model M1, previously successfully applied for calculating the p*K*_a_ values of phosphazenyl phosphines [11], and (ii) model M2, recently developed by our team and validated across a wide range of phosphine bases [32]. As byproduct of the p*K*_a_ calculation using model M2, the PA and GB values calculated at the MP2(fc)/6-311+G(2df,p)//B3LYP/6-31G(d) level of theory are presented in Table S1. Since phosphine proton sponges possess two basic centers, we calculated both the p*K*_a_ for protonation of the first phosphine moiety (p*K*_a1_) and the p*K*_a_ corresponding to the second protonation, i.e., p*K*_a_ of the diprotonated species (p*K*_a2_). The results are presented in Table 4. A review of these p*K*_a_ values indicates that all studied systems qualify as superbases (p*K*_a_ > 25). Notably, even the p*K*_a2_ values for all systems exceed 25 units, indicating that the *mono*protonated forms also exhibit superbasic properties. A comparison between the p*K*_a_ values obtained from models M1 and M2 reveals relatively good general agreement, with differences usually within 3 p*K*_a_ units. However, in some cases, significant discrepancies arise. The most extreme example is system **3bb**, where the p*K*_a1_ calculated by models M1 and M2 differ by nearly 6 units. The exact cause of such a large discrepancy remains unclear, particularly because the two models yield similar results for p*K*_a2_. Another unusual result was observed for systems **5aa** and **5bb**. For system **5aa**, according to model M1, p*K*_a2_ exceeds p*K*_a1_, while for **5bb**, the same counterintuitive trend was obtained by model M2. These two systems differ from others studied in that their phosphazenyl phosphine groups are significantly farther apart from each other, substantially reducing repulsion between the two protons in the diprotonated forms. Additionally, their basic phosphorus atoms are considerably more solvent-accessible, allowing enhanced stabilization through solvation. These arguments lead to the expectation that p*K*_a1_ and p*K*_a2_ should be approximately equal; however, they do not explain why p*K*_a2_ would be higher than p*K*_a1_. However, it should also be emphasized that the accuracy of the model used for calculating p*K*_a_ values is approximately ±1 p*K*_2_ unit. Excluding the systems **5aa** and **5bb**, the difference between the p*K*_a1_ and p*K*_a2_ values ranges from 2.8 to 8.7 kcal mol^−1^. It should be noted that the experimentally measured difference in p*K*_a1_ and p*K*_a2_ for the *bis*-phosphines BINAP and BIPHEP, which are structurally similar to the systems examined here, is 4 p*K*_a_ units [26].

### 2.4. AIM and NBO Analysis

NBO and AIM analyses were performed to determine whether significant delocalization of the phosphorus lone pair into the aromatic system occurs in studied proton sponges, and to investigate the presence of a P^+^–H···P hydrogen bond in their protonated forms. Comparisons were made with the phosphazene proton sponge TPPN. NBO second order perturbation energies *E*^(2)^ for the selected donor–acceptor interactions of the investigated systems are presented in Table S2 in Supporting Information. The key difference between phosphazene and phosphine proton sponges lies in the additional nitrogen atom present in the former, which is bonded to the phosphorus atom via a strongly polarized double bond, exhibiting characteristics similar to those of the lone pair on the nitrogen atom. Substitution at the sp^2^-hybridized nitrogen atom of the phosphazene with an aromatic system—naphthalene in this case—leads to significant delocalization of the nitrogen’s second lone pair into the naphthyl moiety. This is evident from the interaction LP(2)N1 → π*C6–C7(naphthalene), which amounts to 47.88 kcal mol^−1^. In contrast, for the phosphine proton sponge **1bb**, the interaction between the phosphorus lone pair and the aromatic π* orbital, LP(1)P1 → π*C6–C7(naphthalene) is only 2.3 kcal mol^−1^. This confirms that there is no significant delocalization of the phosphorus lone pair into the aromatic system. In the protonated form of the phosphazene sponge TPPN, a strong interaction is observed between the lone pair on the nitrogen atom of the non-protonated phosphazene unit and the antibonding orbital of the N^+^–H bond in the protonated unit (LP(1)N8 → σ*N1–H = 35.5 kcal mol^−1^). Similarly, *E*^(2)^ data also provide additional arguments for the weak HB in the *bis*-phosphines: namely, in the protonated form of the phosphazene sponge TPPN, a strong electron donation from the lone pair on the nitrogen atom of the non-protonated phosphazene moiety to the antibonding orbital of the N^+^–H bond in the protonated subunit (LP(1)N8 → σ*N1–H = 35.5 kcal mol^−1^). In contrast, the corresponding interaction in compound **1bb**, LP(1)P8 → σ*P1–H is only 3.0 kcal mol^−1^, indicating a very weak P^+^–H···P hydrogen bond. To further evaluate the existence and strength of the P^+^–H···P hydrogen bond, an AIM (Atoms in Molecules), analysis was performed, and the hydrogen bond strength was estimated based on the electron density at the bond critical point according to the equation derived in the paper of Affonin et al. [33]:

*E*_HB_(*V*) = −0.277|*V*| − 0.45. (1)

Results are presented in Table 5. For all systems except **5aa** and **5bb**, we identified a bond critical point along the non-covalent part of the P^+^–H···P hydrogen bond. I Interestingly, although **5aa** and **5bb** have the optimal P···P distance for P^+^–H···P hydrogen bond formation, the P^+^–H···P bond does not form; instead, a P^+^–H···N bond is established. This could be explained by the result presented in Table 1, which indicates that the imine nitrogen of the phosphazene part of the phosphazenyl phosphine group is a stronger H-bond acceptor than the phosphorus atom of the same group. Furthermore, the facilitated rotation due to the increased distance between the phosphazenyl phosphine groups in the biphenylene system allowed for the proximity of the nitrogen atom of the non-protonated group to the P^+^–H bond. In protonated system **2bb(H^+^)** a bifurcated P^+^–H···P, P^+^–H···N hydrogen bond is observed.

Although the values obtained from equation 1 for hydrogen bond strength cannot, strictly speaking, be considered quantitative, they nonetheless provide valuable insight into the strength of the P^+^–H···P interactions. This is particularly evident when compared to similar systems exhibiting strong hydrogen bonding, such as the TPPN proton sponge. Based on the values in Table 5, it can be inferred that the P^+^–H···P interaction in phosphine proton sponges is approximately three times weaker than the N^+^–H···N bond in the TPPN proton sponge.

## 3. Computational Details

All calculations were carried out using the Gaussian16 program package [34]. Gas-phase geometry optimizations were performed at the B3LYP-D3/6-311+G(2df,p)//B3LYP-D3/6-31+G(d) level of theory. All structures were fully optimized without any geometric constraints and confirmed as true minima on the potential energy surface by analytical vibrational frequency calculations. For all calculations, we used the default SCF and geometry-optimization convergence thresholds, as well as the default integration grid, in Gaussian 16. These correspond to a (75,302) grid and convergence criteria of 0.00045 Hartree/Bohr for maximum force and 0.0018 Bohr for maximum displacement. Solvation free energies in acetonitrile (model M1) were computed using the SMD solvation model [35] in combination with the M06-2X [36] functional and the 6-31+G(d) basis set. For model M2, used in the p*K*_a_ calculations, geometries were re-optimized at the B3LYP/6-31G(d) level of theory, followed by single-point energy calculations at the MP2(fc)/6-311+G(2df,p) level. Solvation energy contributions in model M2 are calculated using the IPCM continuum solvation model [37], assuming a dielectric constant (ε) of 36.64 for acetonitrile. All optimized structures were visualized using MOLDEN 5.9 [38]. Gas-phase basicities (GB) have been calculated as the Gibbs free energy Δ*G* of the gas-phase protonation reaction: B + H+ → BH^+^. Thus, gas-phase basicity is calculated as GB = *G*(BH^+^) − [*G*(B) + *G*(H^+^)]. The Gibbs free energies (*G*) of the neutral and protonated species contains the electronic energy E_el_ obtained at B3LYPD3/6-311+G(2df,p)//B3LYP-D3/6-31+G(d)) level of theory, and the thermal correction to free energy, *G*_therm_, which sums the zero point vibrational energy (ZPVE), enthalpic and entropic contribution at 298 K. Proton affinities (PA) in the gas phase were calculated as reaction enthalpies for the same protonation reaction, given by PA = H(BH^+^) − [H(B) + H(H^+^)].

p*K*_a_ values in model M1 have been estimated as a relative value utilizing following thermodynamic cycle [11]: 
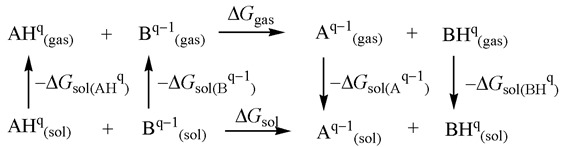


A total charge of the acids and the conjugate bases are represented by q and q^−1^, respectively. The main advantage of this approximation in p*K*_a_ calculations is the expected error cancellation of solvation free energies of the charged molecules in both sides of the chemical equation. The reference base was selected based on similar geometry, electronic structure, and accurately measured p*K*_a_ value. Trimethyphosphine (PCH_3_)_3_ with a p*K*_a_ value of 15.5 [26] served as a reference base for the calculation of p*K*_a_ values in MeCN solvent. According to the above thermodynamic cycle, the basicity of a base B can be calculated relative to the known basicity of base A by the following equation:

p*K*(BH^+^) = p*K*(AH^+^) + {*G*_gas_(B) − *G*_gas_(A) − *G*_gas_(BH^+^) + *G*_gas_(AH^+^) + Δ*G*_sol_(B) − Δ*G*_sol_(A) − Δ*G*_sol_(BH^+^) + Δ*G*_sol_(AH^+^)}/2.303RT (2)

where symbols have their usual meaning.

p*K*_a_ values in model M2 are calculated using equation from ref [20]:

p*K*_a_(BH^+^) = 0.636 × ∆*G*’_a,sol_(BH^+^) − 158.5 (3)

where ∆*G*’_a,sol_(BH^+^) = *G*_gas_(B) + ∆*G*_sol_(B) − G_gas_(BH^+^) − ∆*G*_sol_(BH^+^)

Hartree–Fock plus London dispersion (HFLD) calculations were performed using the ORCA 6.0.0 software [39,40,41,42,43,44,45,46,47]. The optimized structures of selected proton sponges were modified by removing the backbone and replacing it with a hydrogen atom on the phosphorus center. The P–H bond length was fixed at 1.5 Å. Consequently, the dispersion interaction was calculated as the interaction between two phosphazenyl phosphine moieties positioned and oriented exactly as they are in the original proton sponge structures.

## 4. Conclusions

This study investigates the gas-phase and solution-phase basicity (in acetonitrile) of a series of proton sponges derived from phosphazenyl phosphines. A range of aromatic and aliphatic frameworks incorporating phosphazenyl phosphine substituents were employed to modulate basicity, primarily by varying the distance between the phosphazenyl phosphine units. Additionally, the difference in proton affinities (ΔPA) between *bis*- and *mono*-phosphazenyl phosphines—commonly associated with the proton-chelating effect in proton sponges—was analyzed. The ΔPA values determined for the studied systems are lower than those reported for proton sponges based on phosphazenes, cyclopropenimines, and other nitrogen-containing moieties. However, contrary to earlier assumptions, the observed ΔPA values cannot be attributed exclusively to the reduction in electron-pair repulsion or the formation of intramolecular hydrogen bonds upon protonation. Rather, mesomeric and inductive effects, as well as dispersion interactions, were also found to contribute significantly. Model systems were used to evaluate the hydrogen-bond donating and accepting abilities of various phosphines bearing strongly electron-donating substituents. The results indicate that stronger electron-donating groups enhance hydrogen-bond acceptor capacity while simultaneously reducing hydrogen-bond donor ability. The presence and strength of intramolecular hydrogen bonds in the studied proton sponges were assessed using Atoms in Molecules (AIM) analysis. While most systems exhibit a P^+^–H···P hydrogen bond—and some additionally show a P^+^–H···N interaction—these interactions are relatively weak compared to the N^+^–H···N hydrogen bonds characteristic of nitrogen-based proton sponges. This weaker P^+^–H···P interaction is identified as the main factor contributing to the reduced proton-chelating effect in phosphine-based proton sponges. Although all examined compounds display superbasic character, none surpass the basicity of previously reported phosphazenyl phosphines (dma)_3_P and (Pyrr)_3_P. Namely, the strongest proton sponge studied here, **10bb,** has a p*K*_a_ value of 42.0 in acetonitrile, whereas the corresponding phosphazenylphosphine, (pyrr)P_3_P, has a p*K*_a_ value of 43.8 [11]—both values calculated using the same theoretical model. In the gas phase, the highest proton affinity is exhibited by compound **8bb**, with a PA value of 307.0 kcal mol^−1^, which is high but still lower than that of (pyrrP)_3_P, whose PA is 307.5 kcal mol^−1^ This limitation primarily arises from the moderate proton-chelating effect observed in the studied systems. Nonetheless, in contrast to (dma)_3_P and (Pyrr)_3_P superbases, the phosphazenyl phosphine-based proton sponges presented here are capable of accepting a second proton. The calculated p*K*_a2_ values indicate that their monoprotonated forms also retain superbasicity, possessing p*K*_a2_ values in the range from 26.2 to 36.6, underscoring the distinctive bifunctional basic character of these compounds.

## Figures and Tables

**Table 1:** Interaction energies (in kcal mol^−1^) between neutral and protonated phosphines and the molecules HF and NH_3_, respectively, used as probes to estimate H-bond accepting and donating abilities of phosphines ^a^.

	PH_3_	P(CH_3_)_3_	P(Ph)_3_	P(tmg)_3_	P(imme)_3_	(dmaP)_3_P	(pyrrP)_3_P	N(CH_3_)_3_
HF	4.4	9.1	9.0	15.2 (18.3)	17.9 (23.8)	18.4 (18.8)	pt^a^ (23.8)	14.9
	PH_4_^+^	PH(CH_3_)_3_^+^	PH(Ph)_3_^+^	PH(tmg)_3_^+^	PH(imme)_3_^+^	(dma)P_3_PH^+^	(pyrr)P_3_PH^+^	NH(CH_3_)_3_^+^
NH_3_	pt ^b^	10.3	8.3	3.6	6.0	3.3	4.9	20.1

^a^ Numbers given in parentheses refer to the nitrogen acting as an HB acceptor. ^b^ pt refers to the occurrence of the intermolecular proton transfer.

**Table 2:** Calculated values of the proton affinity (PA) and gas basicity (GB) of *bis*-phosphines depicted in Figure 1, compared with PA and GB values for corresponding *mono*-substituted species. ΔPA represents the difference in PA between *mono* and *bis*-phosphines, whereas *E*n and *E*p represent the contributions to ΔPA arising from the neutral base and its conjugate acid, respectively. PA and GB values for (dmaP)_3_P and (pyrrP)_3_P are taken from ref. [11] and are provided for comparison. All units are in kcal mol^−1^.

Molecule	PA	GB	Molecule	PA	GB	ΔPA	*E*n	*E*p
**1aa**	292.9	285.1	**1a**	281.5	273.5	11.4	3.6	−7.8
**1bb**	301.0	292.4	**1b**	284.5	276.7	16.5	2.9	−13.7
**2aa**	292.7	284.8	**2a**	280.9	274.5	11.8	2.1	−9.7
**2bb**	297.7	290.8	**2b**	288.1	279.3	9.6	−1.5	−11.1
**3aa**	291.2	283.9	**3a**	278.7	272.3	12.6	3.2	−9.4
**3bb**	296.8	291.1	**3b**	283.8	276.5	13.0	−1.2	−14.2
**4aa**	291.1	283.7	**4a**	280.1	272.5	10.9	1.3	−9.6
**4bb**	295.8	289.0	**4b**	284.3	276.9	11.5	−3.9	−15.4
**5aa**	294.0	286.4	**5a**	280.8	273.9	13.2	−2.0	−15.2
**5bb**	295.5	288.4	**5b**	286.7	278.6	8.8	−9.0	−17.9
**6aa**	292.0	285.4	**6a**	283.0	275.6	9.1	0.7	−8.4
**6bb**	299.1	292.8	**6b**	288.6	280.8	10.5	0.5	−10.1
**7aa**	290.7	283.4	**7a**	279.9	273.6	10.8	−2.9	−13.7
**7bb**	296.8	289.4	**7b**	285.7	277.9	11.1	0.5	−10.6
**8aa**	296.8	288.6	**8a**	282.6	276.0	14.2	−4.0	−18.2
**8bb**	307.0	296.3	**8b**	288.8	282.2	18.1	1.1	−17.0
**9aa**	292.8	285.4	**9a**	282.1	275.6	10.7	0.0	−10.7
**9bb**	300.6	294.2	**9b**	289.7	281.3	10.8	−5.7	−16.6
**10aa**	296.6	288.4	**10a**	282.0	275.6	14.6	5.7	−8.9
**10bb**	305.3	297.8	**10b**	288.8	282.1	16.5	−2.7	−19.3
(dmaP)_3_P	297.4	291.3	(pyrrP)_3_P	307.5	300.2			

**Table 3:** Calculated values of the proton affinity (PA) and gas basicity (GB) of *bis*-phosphines **Iaa**-**IXbb** (in kcal mol^−1^). compared with PA and GB values for corresponding *mono*-substituted species. ΔPA represents the difference in PA between *mono* and *bis*-phosphines, whereas *E*n and *E*p represent the contributions to ΔPA arising from the neutral base and its conjugate acid, respectively.

Molecule	PA	GB	Molecule	PA	GB	ΔPA	*E*n	*E*p
**Iaa**	286.2	277.5	**Ia**	281.5	274.1	4.7	−0.2	−4.9
**Ibb**	293.1	285.4	**Ib**	287.3	279.5	5.9	−3.0	−8.9
**IIaa**	288.6	281.5	**IIa**	282.4	275.0	6.1	−0.3	−6.4
**IIbb**	293.5	285.4	**IIb**	289.2	282.2	4.3	−9.4	−13.7
**IIIaa**	284.1	275.3	**IIIa**	281.1	273.2	3.0	−3.3	−6.3
**IIIbb**	289.7	281.6	**IIIb**	287.9	281.2	1.8	−10.0	−11.8
**IVaa**	287.2	280.2	**Iva**	281.2	273.2	6.0	0.6	−5.4
**IVbb**	294.7	284.5	**IVb**	288.4	281.8	6.4	−8.9	−15.3
**Vaa**	285.3	277.5	**Vbb**	280.5	273.3	4.8	0.2	−4.6
**Vbb**	290.6	285.1	**Vb**	287.6	279.9	3.0	−1.6	−4.5
**VIaa**	287.5	279.8	**Via**	282.8	275.9	4.7	−0.3	−5.0
**VIbb**	291.3	283.7	**VIb**	286.9	280.3	4.4	−1.7	−6.1
**VIIaa**	286.3	279.6	**VIIa**	281.1	273.9	5.3	0.1	−5.2
**VIIbb**	292.8	284.7	**VIIb**	287.0	279.9	5.8	−1.9	−7.7
**VIIIaa**	288.6	279.6	**VIIIa**	282.7	276.3	5.9	−3.9	−9.8
**VIIIbb**	293.2	286.2	**VIIIb**	289.3	281.1	4.1	−8.8	−11.7
**IXaa**	287.7	280.5	**IXa**	282.0	274.7	5.6	−2.0	−7.6
**IXbb**	289.5	282.3	**IXb**	288.0	280.2	1.6	−5.7	−7.3

**Table 4:** p*K*_a_ values of neutral and monoprotonated phosphine sponges calculated using two computational models.

Molecule	M1		M2		Molecule	M1		M2	
	p*K*_a1_	p*K*_a2_	p*K*_a1_	p*K*_a2_		**p*K*_a1_**	p*K*_a2_	p*K*_a1_	p*K*_a2_
**1aa**	34.0	29.9	36.2	29.7	**6aa**	35.0	29.4	38.2	27.4
**1bb**	36.0	30.0	36.5	28.4	**6bb**	37.0	30.7	37.2	29.4
**2aa**	34.2	28.5	36.2	27.9	**7aa**	33.4	28.7	33.2	31.2
**2bb**	38.9	33.1	37.5	31.0	**7bb**	34.9	30.2	35.9	28.9
**3aa**	34.1	26.2	35.2	25.6	**8aa**	36.3	33.5	36.4	31.8
**3bb**	39.5	30.7	33.7	32.1	**8bb**	37.5	35.8	38.5	29.8
**4aa**	33.5	28.3	34.7	27.8	**9aa**	35.3	31.2	37.0	29.2
**4bb**	37.2	31.4	36.4	31.6	**9bb**	40.2	36.6	39.6	34.8
**5aa**	30.5	31.3	34.0	28.3	**10aa**	37.8	30.3	36.9	29.4
**5bb**	32.8	32.8	31.9	33.5	**10bb**	42.0	34.6	39.2	35.2

**Table 5:** Selected topological parameters of the electron density *ρ*(*rc*) and potential energy (*V*(*r_c_*)) at a critical point as well as bond energies, *E*, of the hydrogen bonds in investigated phosphine proton sponges. TPPN proton sponge is given for comparison.

Compound	BCP	*ρ*(*r_c_*) ^a^	*V*(*r_c_*) ^b^	*E* ^c^
**1aa**, **H^+^**	P···H–^+^P	0.0220	−0.0147	−2.96
**1bb**, **H^+^**	P···H–^+^P	0.0225	−0.0151	−3.02
**2aa**, **H^+^**	P···H–^+^P	0.0262	−0.0171	−3.40
**2bb**, **H^+^**	P···H–^+^P	0.0226	−0.0164	−3.27
	N_imine_···H–^+^P	0.0236	−0.0175	−3.46
**3aa**, **H^+^**	P···H–^+^P	0.0293	−0.0195	−3.84
**3bb**, **H^+^**	P···H–^+^P	0.0229	−0.0167	−3.34
**4aa**, **H^+^**	P···H–^+^P	0.0208	−0.0132	−2.71
**4bb**, **H^+^**	P···H–^+^P	0.0350	−0.0238	−4.53
**5aa**, **H^+^**	N_imine_···H–^+^P	0.0147	−0.0093	−2.02
**5bb**, **H^+^**	N_imine_···H–^+^P	0.0185	−0.0124	−2.58
**6aa**, **H^+^**	P···H–^+^P	0.0173	−0.0093	−2.02
**6bb**, **H^+^**	P···H–^+^P	0.0220	−0.0136	−2.77
**7aa**, **H^+^**	P···H–^+^P	0.0170	−0.0091	−2.02
**7bb**, **H^+^**	P···H–^+^P	0.0213	−0.0131	−2.71
**8aa**, **H^+^**	P···H–^+^P	0.0191	−0.0113	−2.40
**8bb**, **H^+^**	P···H–^+^P	0.0171	−0.0104	−2.21
**9aa**, **H^+^**	P···H–^+^P	0.0197	−0.0116	−2.46
**9bb**, **H^+^**	P···H–^+^P	0.0188	−0.0111	−2.33
**10aa**, **H^+^**	P···H–^+^P	0.0205	−0.0124	−2.58
**10bb**, **H^+^**	P···H–^+^P	0.0210	−0.0135	−2.77
**TPPN**, **H^+^**	N···H–^+^N	0.0545	−0.0460	−8.42

^a^ *ρ*(*r_c_*) in *e* Å^−3^, ^b^ *V*(*r_c_*) in au, ^c^ *E* in kcal mol^−1^ calculated according to *E*_HB_(*V*) = −0.277|*V*| − 0.45 [33].

## Data Availability

There is no data other than what is included in the manuscript and Supporting Information.

## Supplementary Materials

The following supporting information can be downloaded at: https://www.mdpi.com/article/10.3390/ijms26115058/s1.
