# Supplementary material for: Phosphazenyl Phosphine Proton Sponges: Does the Proton-Chelating Effect Enhance Their Basicity?
Source: Int J Mol Sci. 2025 May 24;26(11):5058. doi: 10.3390/ijms26115058 (PMC12155318; doi:10.3390/ijms26115058)
Supplement: Supplementary file 1 [file ijms-26-05058-s001.zip › ijms-3650285-supplementary.pdf]

Phosphazenyl phosphine proton sponges: Does the proton-chelating effect enhance their basicity?

(Supporting material)

Zoran Glasovac[1], Danijela Barić[2], Ines Despotović[2], Borislav Kovačević[2]*

[1] Division of Organic Chemistry and Biochemistry, Ruđer Bošković Institute, Bijenička c. 54, HR-10000 Zagreb, Croatia

[2] Division of Physical Chemistry, Ruđer Bošković Institute, Bijenička c. 54, HR-10000 Zagreb, Croatia

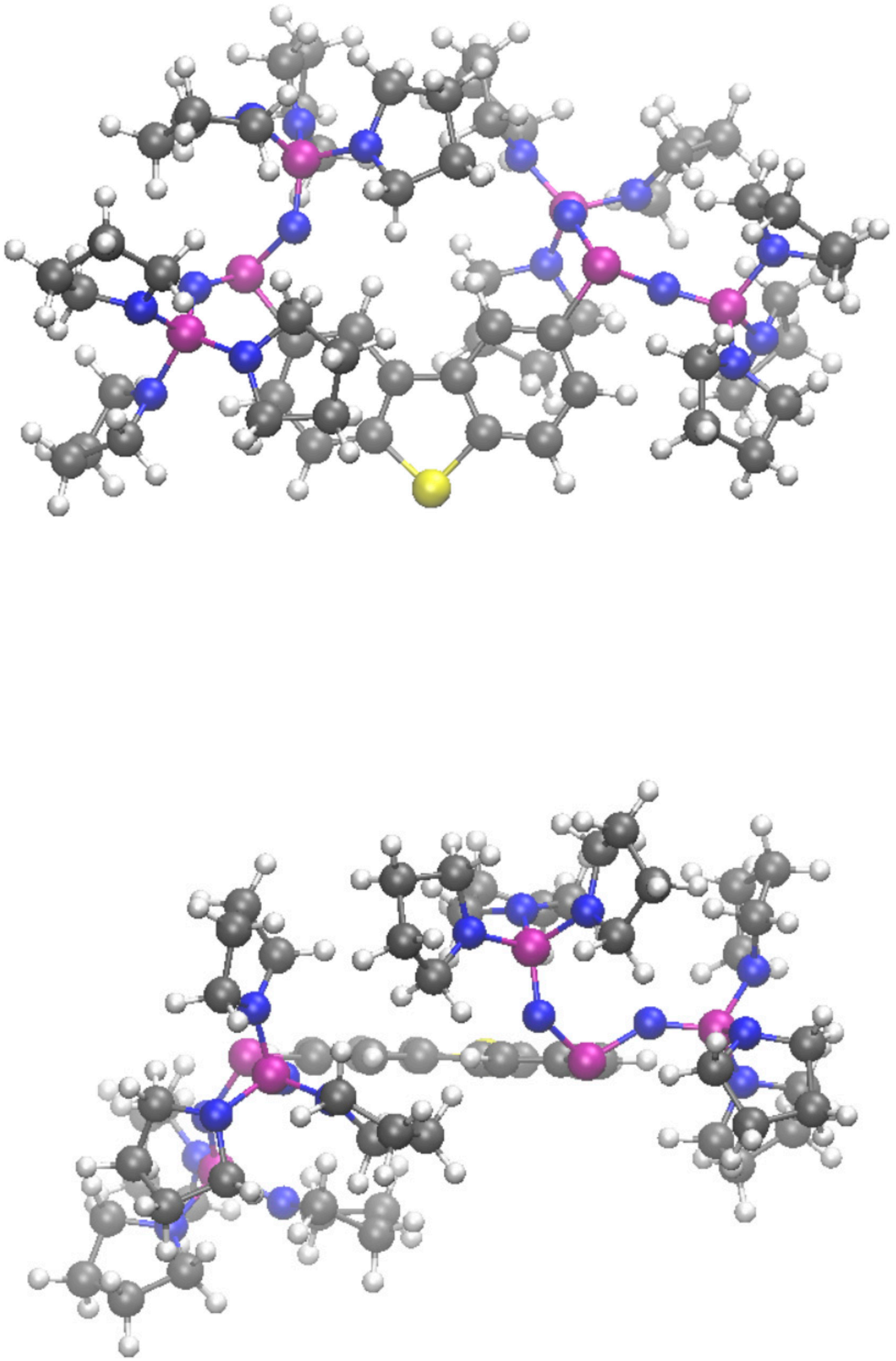

Figure S1 Side and top view of neutral form of molecule **IIIbb**

Table S1 Calculated values of the proton affinity (PA) and gas basicity (GB) of *bis* phosphines calculated at MP2(fc)/6-311+G(2df,p)//B3LYP/6-31G(d) level of theory

**Molecule**	PA	GB	**Molecule**	PA	GB
**1aa**	287.0	279.6	**6aa**	291.6	284.2
**1bb**	293.6	284.1	**6bb**	290.7	284.7
**2aa**	285.7	279.0	**7aa**	283.5	274.1
**2bb**	291.4	284.6	**7bb**	289.7	283.4
**3aa**	286.0	278.1	**8aa**	285.8	279.5
**3bb**	288.2	276.7	**8bb**	294.6	285.5
**4aa**	283.2	276.7	**9aa**	286.5	279.1
**4bb**	293.2	283.2	**9bb**	293.7	285.2
**5aa**	283.7	276.6	**10aa**	287.0	279.6
**5bb**	281.8	275.5	**10bb**	295.0	286.3

Table S2. Second order perturbation energies $E^{(2)}$ in kcal mol^{-1}, for the selected donor-acceptor interactions of the investigated systems

Molecule	Interaction	$E^{(2)}$
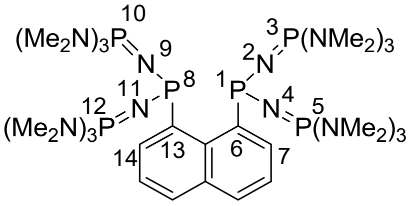 **1aa**	LP(1)P1 → σ* C6-C7<br>LP(1)P1 → π* C6-C7<br>LP(1)P1 → σ* N2-P3<br>LP(1)P1 → σ* P8-N11<br>LP(1)P8 → σ* C13-C14<br>LP(1)P8 → π* C13-C14<br>LP(1)P8 → σ* N11-P12<br>LP(1)P8 → σ* P1-N2	2.08<br>2.31<br>7.30<br>6.39<br>2.23<br>2.10<br>7.51<br>6.77
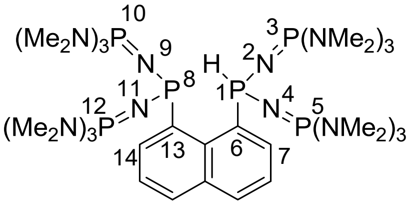 **1aa,H$^+$**	LP(1)P8 → σ* P1-H<br>LP(1)P8 → σ* C13-C14<br>LP(1)P8 → π* C13-C14<br>LP(1)P8 → σ* N11-P12<br>LP(1)P8 → σ* P1-N2<br>LP(1)N11→σ* P(1)-H	1.41<br>2.16<br>2.54<br>7.11<br>2.78<br>1.52
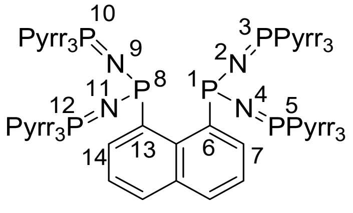 **1bb**	LP(1)P1 → σ* C6-C7<br>LP(1)P1 → π* C6-C7<br>LP(1)P1 → σ* N2-P3<br>LP(1)P1 → σ* P8-N11<br>LP(1)P8 → σ* C13-C14<br>LP(1)P8 → π* C13-C14<br>LP(1)P8 → σ* N11-P12<br>LP(1)P8 → σ* P1-N2	1.93<br>2.82<br>6.61<br>4.76<br>2.68<br>0.68<br>7.08<br>7.52
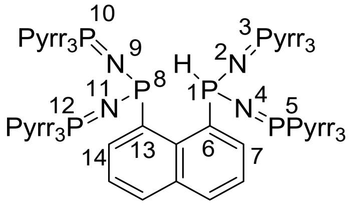 **1bb,H$^+$**	LP(1)P8 → σ* P1-H<br>LP(1)P8 → σ* C13-C14<br>LP(1)P8 → π* C13-C14<br>LP(1)P8 → σ* N11-P12<br>LP(1)P8 → σ* P1-N2<br>LP(1)N11→σ* P(1)-H	3.02<br>2.08<br>3.44<br>6.57<br>1.92<br>0.67
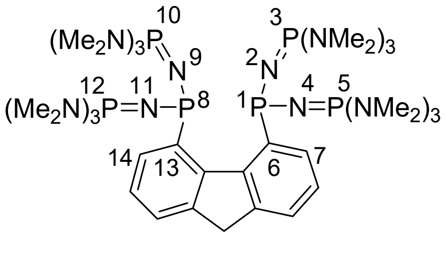 **2aa**	LP(1)P1 → σ* C6-C7<br>LP(1)P1 → π* C6-C7<br>LP(1)P1 → σ* N2-P3<br>LP(1)P1 → σ* P8-N11<br>LP(1)P8 → σ* C13-C14<br>LP(1)P8 → π* C13-C14<br>LP(1)P8 → σ* N11-P12<br>LP(1)P8 → σ* P1-N2	2.36<br>3.33<br>7.14<br>7.41<br>1.37<br>2.70<br>7.05<br>4.20

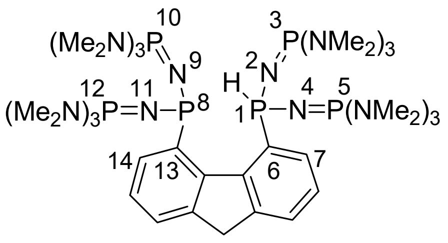<br>**2aa,H+**	LP(1)P8 → σ* P1-H<br>LP(1)P8 → σ* C13-C14<br>LP(1)P8 → π* C13-C14<br>LP(1)P8 → σ* N11-P12<br>LP(1)P8 → σ* P1-N2	5.21<br>2.04<br>4.07<br>6.63<br>2.57
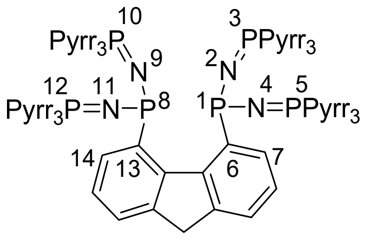<br>**2bb**	LP(1)P1 → σ* C6-C7<br>LP(1)P1 → π* C6-C7<br>LP(1)P1 → σ* N2-P3<br>LP(1)P1 → σ* P8-N11<br>LP(1)P8 → σ* C13-C14<br>LP(1)P8 → σ* N11-P12<br>LP(1)P8 → σ* P1-N2	1.55<br>3.24<br>6.99<br>1.28<br>3.34<br>1.46<br>6.31
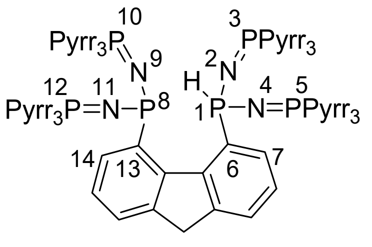<br>**2bb,H+**	LP(1)P8 → σ* P1-H<br>LP(1)N9→ σ* P1-H<br>LP(1)P8 → σ* C13-C14<br>LP(1)P8 → π* C13-C14<br>LP(1)P8 → σ* N11-P12<br>LP(1)P8 → σ* P1-N2	-<br>6.43<br>3.81<br>1.05<br>5.78<br>3.55
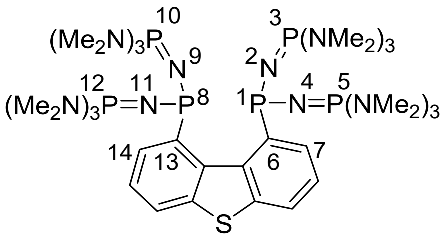<br>**3aa**	LP(1)P1 → σ* C6-C7<br>LP(1)P1 → π* C6-C7<br>LP(1)P1 → σ* N2-P3<br>LP(1)P1 → σ* P8-N11<br>LP(1)P8 → σ* C13-C14<br>LP(1)P8 → π* C13-C14<br>LP(1)P8 → σ* N11-P12<br>LP(1)P8 → σ* P1-N2	2.13<br>4.23<br>7.03<br>8.61<br>1.17<br>3.14<br>6.87<br>4.59
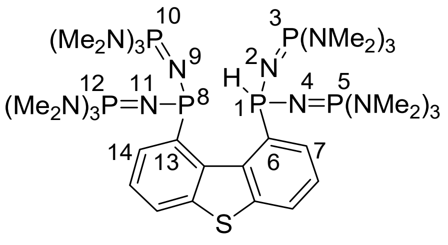<br>**3aa,H+**	LP(1)P8 → σ* P1-H<br>LP(1)P8 → σ* C13-C14<br>LP(1)P8 → π* C13-C14<br>LP(1)P8 → σ* N11-P12<br>LP(1)P8 → σ* P1-N2	8.16<br>1.82<br>4.28<br>6.63<br>2.34

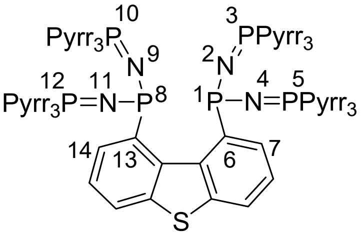<br>**3bb**	LP(1)P1 → σ* C6-C7<br>LP(1)P1 → π* C6-C7<br>LP(1)P1 → σ* N2-P3<br>LP(1)P1 → σ* P8-N11<br>LP(1)P8 → σ* C13-C14<br>LP(1)P8 → π* C13-C14<br>LP(1)P8 → σ* N11-P12<br>LP(1)P8 → σ* P1-N2	2.37<br>4.86<br>7.21<br>11.53<br>1.60<br>2.68<br>7.24<br>7.55
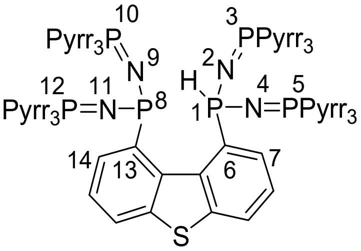<br>**3bb,H$^+$**	LP(1)P8 → σ* P1-H<br>LP(1)N9→ σ* P1-H<br>LP(1)P8 → σ* C13-C14<br>LP(1)P8 → π* C13-C14<br>LP(1)P8 → σ* N11-P12<br>LP(1)P8 → σ* P1-N2	-<br>6.68<br>3.80<br>0.64<br>5.50<br>4.05
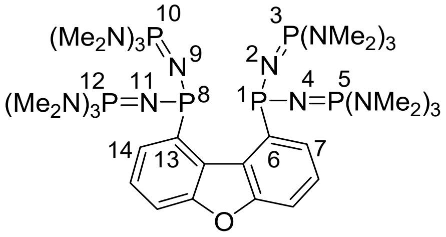<br>**4aa**	LP(1)P1 → σ* C6-C7<br>LP(1)P1 → π* C6-C7<br>LP(1)P1 → σ* N2-P3<br>LP(1)P1 → σ* P8-N11<br>LP(1)P8 → σ* C13-C14<br>LP(1)P8 → π* C13-C14<br>LP(1)P8 → σ* N11-P12<br>LP(1)P8 → σ* P1-N2	1.67<br>3.19<br>7.21<br>3.36<br>2.72<br>3.40<br>7.33<br>5.44
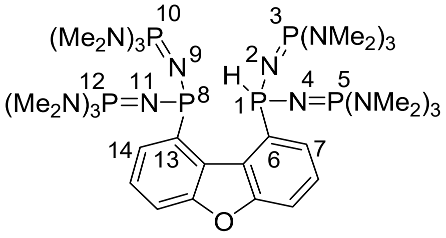<br>**4aa,H$^+$**	LP(1)P8 → σ* P1-H<br>LP(1)P8 → σ* C13-C14<br>LP(1)P8 → π* C13-C14<br>LP(1)P8 → σ* N11-P12<br>LP(1)P8 → σ* P1-N2	2.22<br>2.51<br>3.74<br>6.72<br>2.38
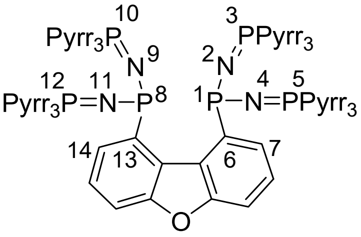<br>**4bb**	LP(1)P1 → σ* C6-C7<br>LP(1)P1 → π* C6-C7<br>LP(1)P1 → σ* N2-P3<br>LP(1)P1 → σ* P8-N11<br>LP(1)P8 → σ* C13-C14<br>LP(1)P8 → π* C13-C14<br>LP(1)P8 → σ* N11-P12<br>LP(1)P8 → σ* P1-N2	2.83<br>4.45<br>7.37<br>7.37<br>2.40<br>2.50<br>7.32<br>5.83

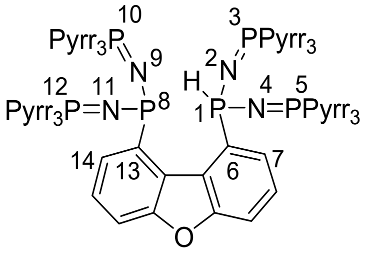 **4bb,H$^+$**	LP(1)P8 → σ* P1-H<br>LP(1)P8 → σ* C13-C14<br>LP(1)P8 → π* C13-C14<br>LP(1)P8 → σ* N11-P12	18.51<br>2.37<br>3.14<br>7.17
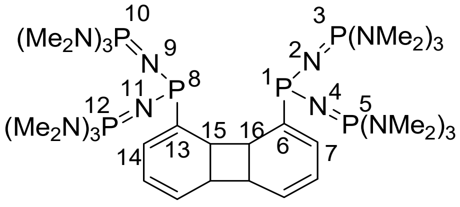 **5aa**	LP(1)P1 → σ* C6-C16<br>LP(1)P1 → σ* C6-C7<br>LP(1)P1 → σ* N2-P3<br>LP(1)P1 → σ* P8-N11<br>LP(1)P8 → σ* C13-C15<br>LP(1)P8 → σ* C13-C14<br>LP(1)P8 → σ* N11-P12<br>LP(1)P8 → σ* P1-N2	1.88<br>4.02<br>7.59<br>0.92<br>2.82<br>2.71<br>7.35<br>0.65
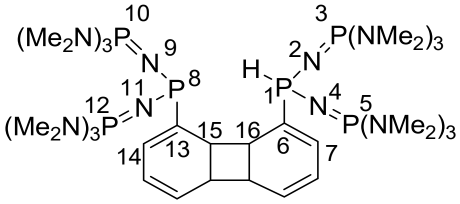 **5aa,H$^+$**	LP(1)P8 → σ* P1-H<br>LP(1)N9 → σ* P1-H<br>LP(1)P8 → π* C13-C15<br>LP(1)P8 → σ* C13-C14<br>LP(1)P8 → σ* N11-P12	-<br>2.80<br>3.34<br>3.10<br>7.14
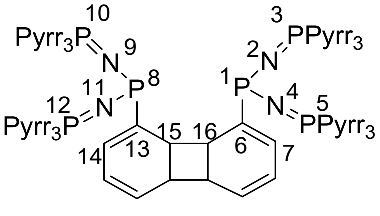 **5bb**	LP(1)P1 → σ* C6-C16<br>LP(1)P1 → σ* C6-C7<br>LP(1)P1 → σ* N2-P3<br>LP(1)P1 → σ* P8-N11<br>LP(1)P8 → σ* C13-C14<br>LP(1)P8 → σ* N11-P12<br>LP(1)P8 → σ* P1-N2	3.60<br>2.51<br>7.03<br>0.80<br>4.78<br>1.06<br>0.83
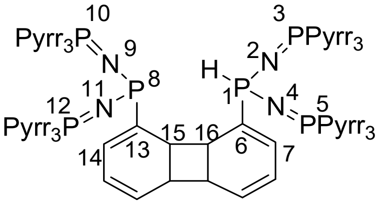 **5bb,H$^+$**	LP(1)P8 → σ* P1-H<br>LP(1)N9 → σ* P1-H<br>LP(1)P8 → π* C13-C15<br>LP(1)P8 → σ* C13-C14<br>LP(1)P8 → σ* N11-P12	-<br>5.59<br>2.73<br>3.89<br>6.54
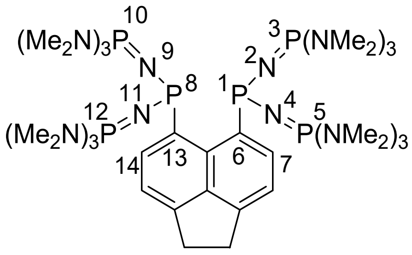	LP(1)P1 → σ* C6-C7<br>LP(1)P1 → π* C6-C7<br>LP(1)P1 → σ* N2-P3<br>LP(1)P1 → σ* P8-N11<br>LP(1)P8 → σ* C13-C14<br>LP(1)P8 → σ* N11-P12<br>LP(1)P8 → σ* P1-N2	2.10<br>2.99<br>6.68<br>2.67<br>3.39<br>1.75<br>6.04

**6aa**		
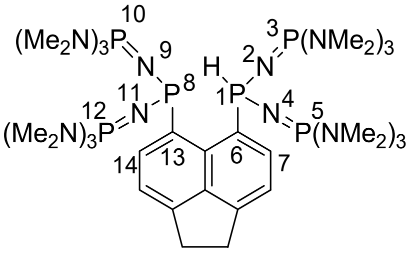<br>**6aa,H$^+$**	LP(1)P8 → σ* P1-H<br>LP(1)P8 → σ* C13-C14<br>LP(1)P8 → σ* N11-P12<br>LP(1)P8 → σ* P1-N2	1.12<br>3.76<br>1.56<br>3.05
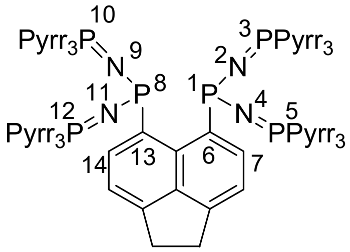<br>**6bb**	LP(1)P1 → σ* C6-C7<br>LP(1)P1 → π* C6-C7<br>LP(1)P1 → σ* N2-P3<br>LP(1)P1 → σ* P8-N11<br>LP(1)P8 → σ* C13-C14<br>LP(1)P8 → π* C13-C14<br>LP(1)P8 → σ* N11-P12<br>LP(1)P8 → σ* P1-N2	3.21<br>0.73<br>7.05<br>6.30<br>2.25<br>2.72<br>6.64<br>3.87
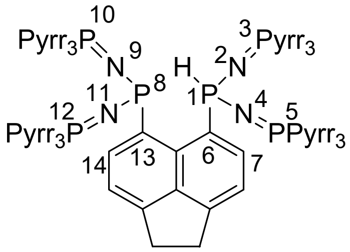<br>**6bb,H$^+$**	LP(1)P8 → σ* P1-H<br>LP(1)P8 → σ* C13-C14<br>LP(1)P8 → π* C13-C14<br>LP(1)P8 → σ* N11-P12<br>LP(1)P8 → σ* P1-N2	1.47<br>3.41<br>1.02<br>7.17<br>3.81
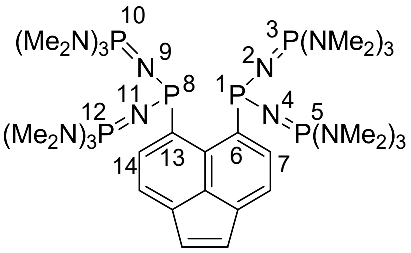<br>**7aa**	LP(1)P1 → σ* C6-C7<br>LP(1)P1 → π* C6-C7<br>LP(1)P1 → σ* N2-P3<br>LP(1)P1 → σ* P8-N11<br>LP(1)P8 → σ* C13-C14<br>LP(1)P8 → σ* N11-P12<br>LP(1)P8 → σ* P1-N2	1.99<br>3.39<br>6.56<br>0.97<br>3.65<br>1.20<br>4.63
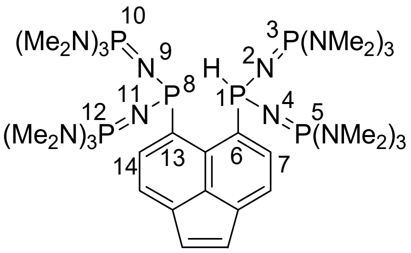<br>**7aa,H$^+$**	LP(1)P8 → σ* P1-H<br>LP(1)P8 → σ* C13-C14<br>LP(1)P8 → σ* N11-P12<br>LP(1)P8 → σ* P1-N2	1.19<br>3.88<br>1.55<br>2.88

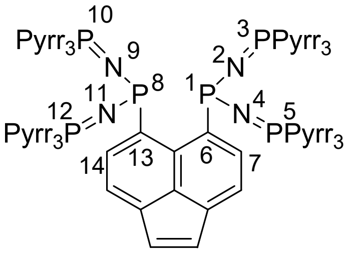<br>**7bb**	LP(1)P1 → σ* C6-C7<br>LP(1)P1 → π* C6-C7<br>LP(1)P1 → σ* N2-P3<br>LP(1)P1 → σ* P8-N11<br>LP(1)P8 → σ* C13-C14<br>LP(1)P8 → σ* N11-P12<br>LP(1)P8 → σ* P1-N2	2.31<br>2.99<br>6.64<br>3.52<br>3.19<br>7.06<br>5.73
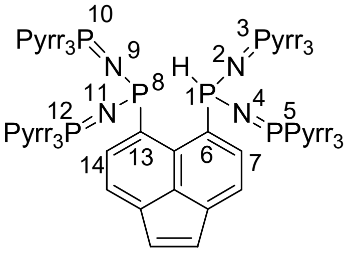<br>**7bb,H+**	LP(1)P8 → σ* P1-H<br>LP(1)P8 → σ* C13-C14<br>LP(1)P8 → π* C13-C14<br>LP(1)P8 → σ* N11-P12<br>LP(1)P8 → σ* P1-N2	1.24<br>3.52<br>1.35<br>7.16<br>3.71
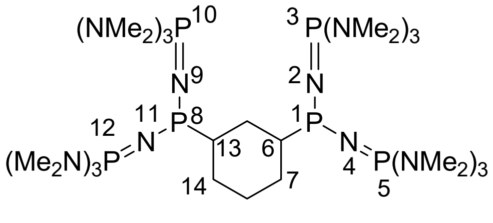<br>**8aa**	LP(1)P1 → σ* C6-C7<br>LP(1)P1 → σ* N2-P3<br>LP(1)P1 → σ* N4-P5<br>LP(1)P1 → σ* P8-N11<br>LP(1)P8 → σ* C13-H<br>LP(1)P8 → σ* N9-P10<br>LP(1)P8 → σ* N11-P12<br>LP(1)P8 → σ* P1-N2	1.46<br>1.25<br>1.61<br>1.05<br>1.62<br>1.63<br>1.11<br>1.25
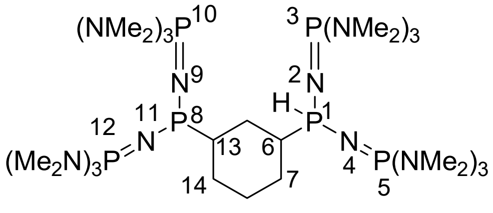<br>**8aa,H+**	LP(1)P8 → σ* P1-H<br>LP(1)P8 → σ* C13-H<br>LP(1)P8 → σ* N9-P10<br>LP(1)P8 → σ* N11-P12	4.86<br>1.60<br>7.40<br>0.72
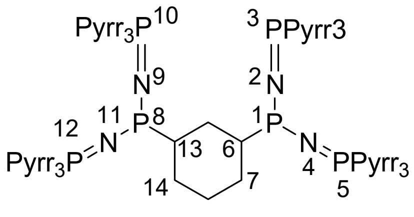<br>**8bb**	LP(1)P1 → σ* C6-C7<br>LP(1)P1 → σ* N2-P3<br>LP(1)P1 → σ* N4-P5<br>LP(1)P1 → σ* P8-N11<br>LP(1)P8 → σ* C13-H<br>LP(1)P8 → σ* N9-P10<br>LP(1)P8 → σ* N11-P12<br>LP(1)P8 → σ* P1-N2	1.18<br>1.35<br>4.58<br>2.50<br>1.52<br>0.91<br>6.64<br>2.14
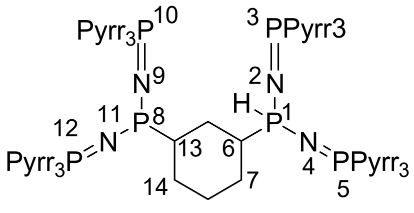<br>**8bb,H+**	LP(1)P8 → σ* P1-H<br>LP(1)P8 → σ* C13-H<br>LP(1)P8 → σ* N9-P10<br>LP(1)P8 → σ* N11-P12<br>LP(1)P8 → σ* P1-N2<br>LP(1)N9→ σ* P1-H	1.70<br>1.76<br>6.72<br>0.79<br>1.36<br>1.16

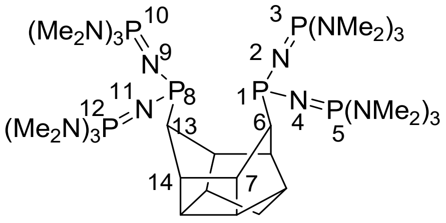<br>**9aa**	LP(1)P1 → σ* C6-H<br>LP(1)P1 → σ* N2-P3<br>LP(1)P1 → σ* N4-P5<br>LP(1)P1 → σ* P8-N11<br>LP(1)P8 → σ* C13-H<br>LP(1)P8 → σ* N9-P10<br>LP(1)P8 → σ* N11-P12<br>LP(1)P8 → σ* P1-N2	1.26<br>1.28<br>2.39<br>2.47<br>1.24<br>1.27<br>2.33<br>2.50
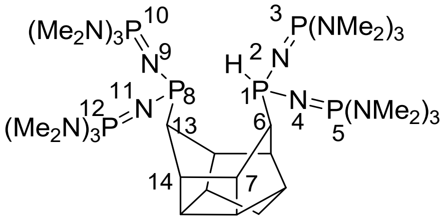<br>**9aa,H+**	LP(1)P8 → σ* P1-H<br>LP(1)P8 → σ* C13-H<br>LP(1)P8 → σ* N9-P10<br>LP(1)P8 → σ* N11-P12<br>LP(1)P8 → σ* P1-N2	4.81<br>1.19<br>2.87<br>1.32<br>1.17
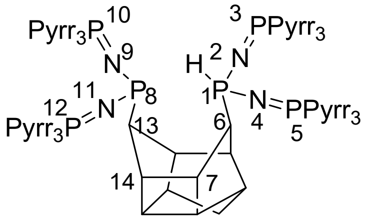<br>**9bb**	LP(1)P1 → σ* C6-H<br>LP(1)P1 → σ* N2-P3<br>LP(1)P1 → σ* N4-P5<br>LP(1)P1 → σ* P8-N11<br>LP(1)P8 → σ* C13-H<br>LP(1)P8 → σ* N9-P10<br>LP(1)P8 → σ* N11-P12<br>LP(1)P8 → σ* P1-N2	1.34<br>1.10<br>7.65<br>4.78<br>1.31<br>6.95<br>0.93<br>3.89
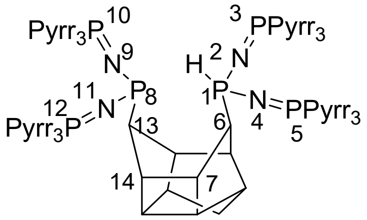<br>**9bb,H+**	LP(1)P8 → σ* P1-H<br>LP(1)P8 → σ* C13-H<br>LP(1)P8 → σ* N9-P10<br>LP(1)P8 → σ* N11-P12<br>LP(1)P8 → σ* P1-N2	2.17<br>1.57<br>7.68<br>0.90<br>1.99
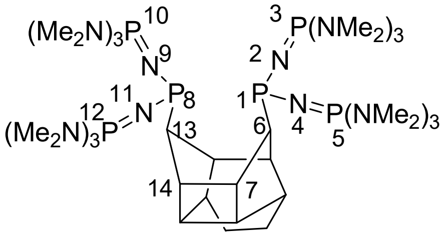<br>**10aa**	LP(1)P1 → σ* C6-H<br>LP(1)P1 → σ* N2-P3<br>LP(1)P1 → σ* N4-P5<br>LP(1)P1 → σ* P8-N11<br>LP(1)P8 → σ* C13-H<br>LP(1)P8 → σ* C13-C14<br>LP(1)P8 → σ* N9-P10<br>LP(1)P8 → σ* N11-P12<br>LP(1)P8 → σ* P1-N2	1.14<br>2.96<br>1.26<br>3.29<br>0.53<br>1.52<br>1.44<br>2.35<br>0.61
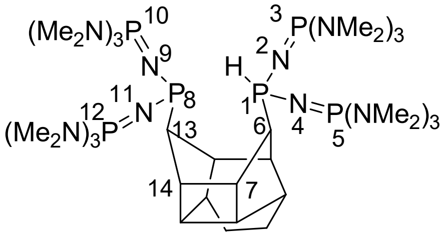<br>**10aa,H+**	LP(1)P8 → σ* P1-H<br>LP(1)P8 → σ* C13-H<br>LP(1)P8 → σ* N9-P10<br>LP(1)P8 → σ* N11-P12<br>LP(1)P8 → σ* P1-N2	3.84<br>0.97<br>2.87<br>1.36<br>1.64

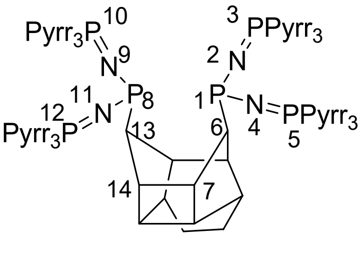 **10bb**	LP(1)P1 → σ* C6-H	1.07
	LP(1)P1 → σ* C6-C7	0.63
	LP(1)P1 → σ* N2-P3	0.90
	LP(1)P1 → σ* N4-P5	7.02
	LP(1)P1 → σ* P8-N11	5.71
	LP(1)P8 → σ* C13-H	1.02
	LP(1)P8 → σ* C13-C14	0.65
	LP(1)P8 → σ* N9-P10	6.95
	LP(1)P8 → σ* N11-P12	0.84
	LP(1)P8 → σ* P1-N2	5.68
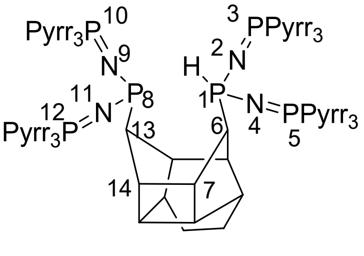 **10bb,H⁺**	LP(1)P8 → σ* P1-H	1.22
	LP(1)P8 → σ* C13-H	1.32
	LP(1)P8 → σ* N9-P10	7.52
	LP(1)P8 → σ* N11-P12	0.74
	LP(1)P8 → σ* P1-N2	3.14
	LP(1)N9 → σ* P1-H	0.99
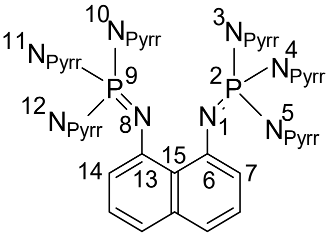 **XXbb,H⁺**	LP(1)N1 → σ* C6-C7	9.43
	LP(1)N1 → π* C6-C7	0.87
	LP(1)N1 → σ* C6-C15	3.58
	LP(1)N1 → σ* P2-N3	13.40
	LP(1)N1 → σ* P2-N4	3.61
	LP(1)N1 → σ* P2-N5	9.98
	LP(2)N1 → σ* C6-C7	0.88
	LP(2)N1 → π* C6-C7	47.88
	LP(2)N1 → σ* C6-C15	1.98
	LP(2)N1 → σ* P2-N3	0.98
	LP(2)N1 → σ* P2-N4	21.56
	LP(2)N1 → σ* P2-N5	15.58
	LP(1)N8 → σ* C13-C14	8.49
	LP(1)N8 → π* C13-C14	1.25
	LP(1)N8 → σ* C13-C15	2.82
	LP(1)N8 → σ* P9-N10	11.77
	LP(1)N8 → σ* P9-N11	5.05
	LP(1)N8 → σ* P9-N12	7.37
	LP(2)N8 → σ* C13-C14	1.26
	LP(2)N8 → π* C13-C14	43.28
	LP(2)N8 → σ* C13-C15	2.66
	LP(2)N8 → σ* P9-N10	0.66
	LP(2)N8 → σ* P9-N11	16.99
	LP(2)N8 → σ* P9-N12	13.54
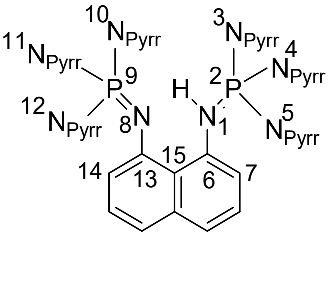	LP(1)N8 → σ* N1-H	35.55
	LP(1)N8 → σ* C13-C14	7.78
	LP(1)N8 → π* C13-C14	0.93
	LP(1)N8 → σ* C13-C15	2.63
	LP(1)N8 → σ* P9-N10	8.03
	LP(1)N1 → σ* P9-N11	4.26
	LP(1)N1 → σ* P9-N12	5.05
	LP(2)N8 → σ* C13-C14	1.05
	LP(2)N8 → π* C13-C14	38.52

	LP(2)N8 → σ* C13-C15<br>LP(2)N8 → σ* P9-N10<br>LP(2)N8 → σ* P9-N11<br>LP(2)N8 → σ* P9-N12	2.13<br>-<br>15.88<br>14.41

Cartesian coordinates of neutral and protonated proton sponges **1aa**-**10bb**

1aa

C 0.1167583899 -0.0967751493 -0.0324268597
C 0.0591293893 -0.0951986412 1.3894750482
C 1.2751390165 -0.0944057751 2.1692865744
C 2.5184326259 0.1121477852 1.4604740238
C 2.5140718549 0.1053962291 0.0752363674
C 1.3273198243 -0.0367649496 -0.6805141442
C -1.2092737557 -0.0831324843 2.0346377904
C -1.2941060581 -0.1240931259 3.4059138026
C -0.1126107196 -0.2666423718 4.169472462
C 1.1472349744 -0.2911074368 3.5956354327
P 2.6368167702 -0.6549177089 4.7021146704
N 1.8252262264 -0.8164849191 6.2176931055
P 2.536097576 -0.2670150151 7.5103764463
N 1.8182329812 1.1546055026 8.09059286
C 0.3956342574 1.3684838854 7.8427510104
P 4.1498493426 0.4619770621 2.3550627834
N 5.1577095073 0.700557216 0.9755700942
P 6.6894034572 0.3637767188 1.0717285582
N 7.6306997437 1.7664832961 0.9606968733
C 7.1206024988 2.8761955747 0.1599407762
N 3.8464487315 1.9886625287 3.0043714806
P 3.3331395783 3.4098062505 2.6435450482
N 2.5042813914 3.8098815533 1.2160986228
C 3.2293921923 3.7870307048 -0.0575866075
N 2.2538173487 3.9192685554 3.8462566392
C 1.8443466823 3.0367316563 4.9324234365
N 4.6557380152 4.4870181855 2.5271462765
C 4.4461664549 5.9199618619 2.3467780905
C 1.5291627259 5.1804449509 3.8029590886
N 3.0313955254 -2.2190678916 4.2123255441
P 2.4490796647 -3.628040626 3.9171107027
N 2.984004895 -4.1384604957 2.3953461002
C 2.6633868949 -5.437613844 1.8243851317
N 0.7935392128 -4.0023962576 3.864423886
C 0.0195578643 -3.8992878201 5.1067076908
N 2.959280763 -4.7150992859 5.137016393
C 2.6142087865 -6.13289397 5.0868647738
C 0.0145235313 -3.6065347343 2.6853941102
N 2.442288034 -1.4798964718 8.6912080168
C 2.2783236215 -2.8813490642 8.3162293744
N 4.1763301989 0.2058944872 7.5047493072
C 4.5849761663 1.4351580821 6.8054296584
C 2.959115784 -1.2953961435 10.0391577999
C 5.1581355813 -0.8672644449 7.3273744291
C 2.333853391 1.9594929616 9.1918134757
N 7.3687816086 -0.351200689 2.4642621417
C 7.4014135882 0.4135503452 3.7169328956
N 7.0933932072 -0.6848115213 -0.1989845544

C	8.4678867333	-1.0845095276	-0.4642589687
C	7.081580299	-1.7689098259	2.7086112484
C	6.102609977	-1.5896046484	-0.7734115215
C	9.0626716549	1.8440603404	1.2175574689
C	5.8311014727	4.1888085305	3.3414491522
C	1.109788325	3.3766179086	1.0726082641
C	4.2793214201	-4.4828202686	5.7189564437
C	3.8497288936	-3.3123599629	1.5639759924
H	-2.1064747791	-0.0408423031	1.4196028739
H	-0.8160372072	-0.138512578	-0.592058068
H	-0.1834359138	-0.4139232603	5.2429100743
H	3.4584588327	0.2660458414	-0.4369534353
H	4.4638286625	-3.4112734175	5.7894557704
H	4.2139453947	4.2414499335	0.0626927915
H	0.5830910247	-4.3236495276	5.939255359
H	2.4345097658	2.1230985626	4.9054959439
H	4.3110212385	-4.9198819306	6.7256989107
H	3.3596563375	2.7641549493	-0.4378354577
H	-0.2275065928	-2.8574922001	5.3523246511
H	1.9921164119	3.5322240829	5.9038414256
H	-0.9116706236	-4.4683238285	4.988248875
H	0.7810388453	2.7670233079	4.8405129359
H	1.6008346839	-6.26535576	4.7029615048
H	0.6415327381	3.2492997446	2.050054215
H	3.3103974862	-6.7156643413	4.4592715221
H	0.550602261	4.1392757076	0.5126865459
H	2.6529632945	-6.5472562407	6.1033914787
H	1.0365035059	2.4250735531	0.5363496752
H	-0.7920178749	-4.3364004119	2.529725991
H	0.4425315113	4.9998523028	3.8314949256
H	-0.4306658084	-2.6136634747	2.8055948431
H	1.7863990316	5.8158236623	4.6646950395
H	0.6459071824	-3.5902645712	1.795852397
H	1.7484457349	5.728279345	2.8861110054
H	1.8900145223	-5.9393727979	2.4077915547
H	3.623737863	6.1008723998	1.6513160807
H	3.5502549109	-6.0896945002	1.777637836
H	5.3558208199	6.3647225861	1.9213928996
H	2.2820301213	-5.3153192922	0.7988478644
H	4.2291253027	6.4391115494	3.2964477606
H	3.3676098213	-3.0969214849	0.5985759124
H	5.7363291319	4.5790587343	4.3703362498
H	4.8034346857	-3.8267654841	1.3670893893
H	6.7138437213	4.6517083518	2.8806796189
H	5.3011444316	-1.1207230206	6.266048524
H	6.1808880506	-1.5790751365	-1.8697535932
H	4.8445492643	-1.7684999112	7.8592228703
H	5.1012766495	-1.270300057	-0.4870533823
H	6.1215027034	-0.542583597	7.7424993461
H	6.2547091171	-2.6270466941	-0.4310147699
H	4.6906838029	1.2797157101	5.7238741545

H 8.6177234891 -1.1950774129 -1.5471819853
H 5.5442768184 1.7700641577 7.2241176837
H 8.7171455262 -2.0478283076 0.012674441
H 3.8469941041 2.2226694288 6.9591942085
H 9.1735735875 -0.3371873646 -0.1034116149
H 3.399541876 1.7757921215 9.3380592845
H 9.3953977538 0.987195803 1.8060265208
H 2.1986892399 3.0253998706 8.9574921107
H 9.6466988711 1.8853048823 0.2828636276
H 1.801532543 1.7554271916 10.1359634203
H 9.2831399656 2.7594379793 1.7863097324
H 0.2146785525 2.4381499507 7.6705442351
H 7.5583676074 2.8803763161 -0.8520497904
H 2.3044505744 -1.8047558774 10.7599848015
H 7.8805527363 -2.1892266438 3.334582376
H -0.2218653921 1.0456713739 8.6972901788
H 7.3723784879 3.8260545892 0.6500920958
H 3.9743378622 -1.7138886637 10.1494393313
H 6.1178420587 -1.9073683331 3.2188116346
H 0.0886476443 0.819106949 6.9536986648
H 6.0363463998 2.8013079226 0.086157674
H 1.5045899977 -3.3474952264 8.9431962107
H 8.2251215412 0.0387066557 4.3406890595
H 3.2160731259 -3.4443025744 8.4577681385
H 6.4600150208 0.3171070934 4.2753312647
H 1.9839426485 -2.9498734952 7.2699815975
H 7.5762953811 1.4712171233 3.5132843654
H 3.0005170027 -0.2411745744 10.3092498299
H 7.0609818376 -2.3249733541 1.7691916223
H -2.2620414499 -0.1013038997 3.9029412118
H 1.3752557968 -0.0456436545 -1.7677540645
H 4.0521430892 -2.3685310308 2.0685354217
H 5.9781713199 3.1110510688 3.3851341985
H 5.0846853442 -4.9439008316 5.1198466391
H 2.6618259515 4.3693406139 -0.7952505375

1aaH^+
C -0.9519255646 -0.6932970729 1.88805831
C 0.2847698392 -0.295677197 2.520928017
C 0.3882200654 -0.471827029 3.9516422767
C -0.612597128 -1.1879338279 4.6649265057
C -1.7129742451 -1.6815530756 4.0080501898
C -1.8822703897 -1.3970258275 2.6349598582
C 1.4366398247 0.2906970858 1.8686742401
C 2.4820694914 0.8097978223 2.6199849896
C 2.4971579392 0.7427772904 4.029458169
C 1.4846839342 0.0748175171 4.6741843734
P 1.7791394946 0.1931823636 0.0722068047
N 1.8954091739 -1.3320778178 -0.4236928638
P 2.603626269 -2.6616408552 0.0530355229
N 1.4286774254 -3.8197650903 0.3321986556

C 1.7563125315 -5.1805473808 0.7557358203
P -1.4108892126 -0.2275112435 0.1099969818
N -1.4172723446 1.4602132442 0.2363088277
P -2.2781374097 2.5432173434 0.988166929
N -3.0531377236 2.2555038395 2.4647671528
C -2.2446063801 2.2299226771 3.6874047156
N -3.0112168812 -0.7971816751 0.0391107888
P -3.5536961095 -1.5439706751 -1.2474365914
N -5.1332169817 -1.0113628723 -1.4810203883
C -6.0122093027 -1.6349814908 -2.4644771006
N -2.7760555787 -1.3721188255 -2.7541517877
C -2.9234366114 -0.0921956251 -3.4460446535
N -3.5398115849 -3.2272057662 -1.0962032533
C -3.7346705838 -4.1747821269 -2.1940521884
C -1.4687107613 -1.9895228602 -3.0046553817
N 3.0663624826 1.1537731902 -0.2017539081
P 3.2754283241 1.9407873888 -1.5716687299
N 1.9390872311 2.2538767253 -2.5473124779
C 1.0135381879 3.3331876226 -2.1738430458
N 3.9454853948 3.417668341 -1.1623352367
C 3.7830074246 4.0285820681 0.1575884181
N 4.2674248538 1.0636714947 -2.6027849068
C 4.4897287048 1.3948270545 -4.0121599961
C 4.3959719645 4.3515308829 -2.1931511584
N 3.7199297185 -3.0852947092 -1.1456163567
C 3.4159569239 -2.7334526574 -2.5363272765
N 3.5452122067 -2.7109988359 1.4375246262
C 2.9347389989 -2.8482278008 2.7668308145
C 4.4779491794 -4.3351183836 -1.0275101602
C 4.8378509093 -2.0170910399 1.4712091718
C 0.0228682793 -3.627313478 -0.0234724739
N -3.5776415378 3.0568957583 0.0333776921
C -3.3477533208 3.1825693003 -1.4048130345
N -1.2502845125 3.859923692 1.2673904288
C 0.1920742489 3.7010910501 1.4091296006
C -4.6950763398 3.8763846982 0.4998500937
C -1.7594929697 5.099847842 1.8451160949
C -4.2131579793 1.3607936919 2.5453856256
C -3.8209557262 -3.8167140317 0.2116711946
C -5.5701988118 0.3192998807 -1.0644046351
C 5.3657972677 0.2909917201 -2.0118828851
C 1.2812686599 1.18893054 -3.316967882
H -0.4900791631 -1.3284244575 5.7363756139
H 1.504297438 -0.0481354221 5.7548521172
H -2.8066730501 -1.6804447552 2.1436438422
H 3.3270353307 1.2505482795 2.1010327721
H -2.563696421 2.4915868024 -1.7142635448
H 5.2876196989 -2.0032278355 0.477704908
H -4.788205514 1.3879122696 1.6212966105
H -0.1424567151 -2.6072463436 -0.3654411282
H -4.2696829636 2.9336037644 -1.944771314

H	4.7257396659	-0.9825504879	1.8252139969
H	-3.911997063	0.3220034068	2.722339378
H	-0.2718320879	-4.3278101923	-0.8182119821
H	-4.8572338076	1.6915214202	3.3705690146
H	-0.6133769348	-3.8066958552	0.8527778847
H	-4.7768671971	3.8411051809	1.5865333051
H	1.9179982653	-3.2330454139	2.6884377025
H	-4.5852042776	4.9264617142	0.1865227691
H	3.5328688005	-3.5447748153	3.3679611524
H	-5.6310704462	3.4952626526	0.0683182668
H	2.896826869	-1.8829243897	3.2815284268
H	-2.8563179364	2.5829005104	4.527435125
H	1.0901515256	-5.4797009891	1.5759225272
H	-1.8864593285	1.219247421	3.9178644261
H	1.6284532538	-5.8969722928	-0.0683106789
H	-1.3794498846	2.8880724544	3.5962400884
H	2.7832892352	-5.2417221112	1.1187348602
H	-2.8290455307	5.2065156672	1.6687344914
H	4.7544053354	-4.51764669	0.0126236203
H	-1.2521389747	5.9565368854	1.3823213149
H	5.4000203386	-4.2467593294	-1.6137424081
H	-1.5872040819	5.1485396814	2.9323406618
H	3.9145986234	-5.2027026874	-1.4043874048
H	0.5077005021	3.6843498733	2.4652927144
H	2.722075914	-3.4496705779	-3.0051950431
H	0.7015177137	4.5399905673	0.9164650053
H	4.349835827	-2.7397782393	-3.1103203222
H	-2.1804819618	0.6465201807	-3.1075742724
H	4.7525233323	4.4018874385	0.5105390022
H	-3.9200079151	0.3230603258	-3.2852381956
H	3.4071685086	3.2918373026	0.8660174919
H	-2.7893155395	-0.2488498932	-4.5238241597
H	3.0812686584	4.8755058591	0.1167861356
H	-0.6411080399	-1.3425780517	-2.6844224353
H	5.3455627704	4.8069794215	-1.8866978499
H	-1.3734924806	-2.1942434598	-4.079713313
H	3.6628620336	5.1566426838	-2.3532908062
H	-1.3814191893	-2.9319914723	-2.4650075577
H	4.5551576426	3.8442214185	-3.1442876779
H	-3.6293856321	-3.6798293884	-3.1598706569
H	3.6771409476	2.011153742	-4.3993029366
H	-2.9829490396	-4.9739555797	-2.1266836489
H	5.4418528225	1.9265229435	-4.156961361
H	-4.7277851175	-4.6469135965	-2.1444861466
H	4.5285563964	0.4670373119	-4.5972677023
H	-3.1773217147	-4.6932385643	0.3643117276
H	6.2980666998	0.8744182288	-1.9812792586
H	-7.0467778574	-1.5949352444	-2.1017906216
H	0.5878210872	3.7667265089	-3.0870903571
H	-4.8702429702	-4.1402036218	0.290975424
H	5.5354515941	-0.6058557566	-2.6180333958

H -5.9708575269 -1.1206397711 -3.438355696
H 0.1951033483 2.9634043703 -1.540734547
H -3.6150663412 -3.0956765428 1.0005637038
H 5.1010327724 -0.0197885222 -1.0016146335
H -6.4957575001 0.2430850335 -0.4776964769
H 1.0097224607 1.570946761 -4.3096702337
H -5.7756897898 0.9566456415 -1.9395630402
H 0.3676277317 0.844149082 -2.8153872683
H -4.7987064601 0.7918535282 -0.4584412858
H 1.9480121537 0.334416193 -3.4409789052
H -5.7544563311 -2.682115385 -2.6189314779
H 1.5416987873 4.1231894755 -1.6372723229
H -2.4773841476 -2.2375750927 4.545522185
H 3.3248361392 1.1751919186 4.5847655656
H 0.5061656311 2.7717852107 0.9405489927
H 2.9822332938 -1.734805279 -2.5794686867
H -3.0539846213 4.2085040358 -1.679708049
H 5.5109048442 -2.5487277391 2.15447431
H 0.6888076028 0.7249858257 -0.6040458488

1bb
C 0.1710041285 0.7280169654 0.0953016705
N 0.1441807413 0.4378352679 1.533987353
C 1.552408499 0.4017312969 1.9679223572
C 2.2788337469 -0.292082552 0.7901485942
C 1.2925332761 -0.1980477269 -0.4078334783
P -1.0571052735 0.9666761532 2.6044666086
N -2.4410125868 0.6031470383 1.6897702078
C -2.6653833867 -0.8458747095 1.4216383495
C -4.1515263335 -1.1097893486 1.7531149521
C -4.5296532194 0.0405859806 2.7000051475
C -3.7191171006 1.2084723823 2.1266564637
N -1.0599056108 0.2691512895 4.0015044062
P 0.216044458 0.2161140193 5.1679198461
N 0.6224980993 -1.4177194353 5.2699005243
P -0.0616520723 -2.8182659018 5.354800201
N 0.8185629254 -3.753630591 6.4426688012
C 1.3795669125 -3.1995626502 7.6875325186
C 2.2617633986 -4.344659742 8.2508053858
C 2.0295886488 -5.5507122706 7.3068247719
C 0.7229900092 -5.2033460465 6.5757517583
C -0.8235304082 0.4278162273 6.7366797058
C -0.2731474501 0.298434861 8.0707680198
C -1.2105334906 0.2993329342 9.1742357349
C -2.5909572542 0.5670989039 8.9520668444
C -3.0574561782 0.8076188733 7.6835598927
C -2.1703002608 0.7109176198 6.5871859128
C -0.7660639272 0.0451834633 10.4995716763
C 0.5661387926 -0.1792447583 10.7545078373
C 1.4976020057 -0.0877439938 9.6999184512
C 1.1288690536 0.181767243 8.3920191453

P	2.5328519721	0.4654775533	7.1253928738
N	2.8101487183	2.1263687457	7.2767666354
P	2.7753320083	3.3774566324	8.194413658
N	1.345624493	4.2616692618	8.0958286194
C	0.0601176381	3.5835052829	7.8259607314
C	-0.9226125996	4.7305524562	7.5334191369
C	-0.0158176087	5.8698765782	7.0426202399
C	1.2268371765	5.7142834881	7.9303537627
N	3.7707471971	-0.3158253454	8.0268451149
P	5.0964062673	-0.9080518437	7.4393022518
N	5.0604459836	-1.4857296442	5.8688661336
C	6.2088818459	-1.5871502159	4.9450142644
C	5.8564476244	-2.7836279529	4.0490393468
C	4.3226076778	-2.7239752888	3.9905454095
C	3.936049209	-2.346103412	5.4240755385
N	5.5755841155	-2.14851846	8.465656549
C	6.7272072981	-3.0153296206	8.1754424746
C	7.0339709841	-3.6867240943	9.5266527446
C	5.6704450679	-3.6905958724	10.2382444848
C	5.0681396594	-2.3385218485	9.8331168986
N	6.4600550464	0.1062437873	7.3762209731
C	6.3753781842	1.3220242983	6.5233623646
C	6.8837304886	2.4639492474	7.4160828971
C	7.8416398589	1.7528159492	8.3821361972
C	7.1085280609	0.4361638262	8.6657774695
N	-1.0260289241	2.6456367803	2.7004867859
C	-1.2167524141	3.5567925804	1.5585257998
C	-1.8444027057	4.8047134268	2.1955080609
C	-1.1746920877	4.8429699419	3.5781836521
C	-1.1259359862	3.3635961526	3.9924985376
N	3.0184604826	3.148623917	9.8395685433
C	2.4081832319	3.8759182509	10.9473891957
C	2.3496134566	2.8077829498	12.0507103405
C	3.6271883167	1.9616943339	11.8215931915
C	4.0581669816	2.2542437622	10.3570142608
N	3.9950213198	4.4493401183	7.7396180504
C	4.6315746023	5.4168547387	8.6426132882
C	5.6834915498	6.0922736812	7.7495043405
C	5.0102991102	6.0956232402	6.3662367029
C	4.2779367895	4.7428216935	6.3236389994
N	-1.6785985227	-3.0394217432	5.8127255364
C	-2.0875094983	-2.9939487931	7.2293388915
C	-3.6084480682	-3.1533875541	7.1449294427
C	-3.9689337124	-2.3233355321	5.8993539022
C	-2.7635838985	-2.5041547747	4.9521761829
N	-0.0336123168	-3.592255731	3.8607920725
C	-0.7691130414	-4.7975405846	3.4673943842
C	-1.1476169221	-4.5166309216	2.0081929931
C	0.1070683882	-3.7959813284	1.4844539638
C	0.5854294057	-2.9475175741	2.6825865521
H	-1.6230791541	-4.9656245653	4.128891336

H	-2.5571278826	0.812279762	5.57739863
H	2.5528616536	-0.2312482721	9.9084333069
H	-4.1093385012	1.0301778504	7.5120477024
H	0.9093318497	-0.3956979624	11.764878783
H	-3.262326113	0.586926109	9.8088226797
H	-1.501085053	0.0279509847	11.3025846141
H	-4.1136785228	-2.8039337558	8.0517312478
H	-3.8643759023	-4.2100014792	6.9920713745
H	-1.6048202115	-3.7997102645	7.7912505756
H	-1.8284520868	-2.0387916237	7.7056734427
H	-4.9103641424	-2.6374278843	5.4348695223
H	-4.0669939815	-1.26995486	6.1775678667
H	-2.4670360369	-1.5603856052	4.4820457937
H	-2.9876048375	-3.2258113015	4.1548837504
H	-0.1541089503	-5.5102481279	7.1755713088
H	1.9689717315	-4.5838041442	9.2793254211
H	3.3148736734	-4.0499998507	8.2710804384
H	0.6438150844	-5.6833101027	5.5977785498
H	2.8407329592	-5.6184615101	6.5722187547
H	1.9736576186	-6.5079334606	7.8375993112
H	1.9464402903	-2.2970029808	7.4734556673
H	-0.1275525582	-5.6945432998	3.5165091398
H	0.5928024845	-2.9265651055	8.4056171862
H	-2.0165118402	-3.8479808463	1.9740794814
H	-1.3915578411	-5.4254009325	1.4462903659
H	0.8691441418	-4.5355195553	1.2068367279
H	1.6786281442	-2.9305596416	2.7606713686
H	-0.0890416593	-3.174395244	0.6040865582
H	0.2428990408	-1.915826426	2.5998077842
H	1.6574981637	-0.1312917467	2.9149483286
H	1.9406057597	1.4224189371	2.119358674
H	3.2339389685	0.2003211073	0.5784227593
H	2.4979068884	-1.3365729352	1.0335707428
H	1.762637725	0.1852885051	-1.3200338514
H	0.8764530485	-1.1853451111	-0.63684114
H	0.4339448879	1.7827299281	-0.0990033891
H	-0.8023217169	0.5331130689	-0.3596026724
H	-0.272853345	3.1343071417	4.6372835225
H	-2.4286284855	-1.0841232067	0.3772125467
H	-2.0121110126	-1.4484011626	2.0590481717
H	-4.2969998899	-2.0984801412	2.2012104679
H	-4.7621020701	-1.063127859	0.8423486365
H	-3.5456040181	2.0109655943	2.8486917023
H	-4.2309781089	1.647400671	1.2566124567
H	-5.6071087903	0.2391639199	2.7277698262
H	-4.1875052747	-0.1743111339	3.7180244915
H	-1.8533891554	3.0994539434	0.7931631129
H	-1.7119112779	5.4582126196	4.3069158946
H	-0.252979811	3.8075178875	1.0893466911
H	-0.1560739817	5.2420969112	3.4880877447
H	-1.6785827116	5.71128068	1.6035006871

H	-2.927720232	4.6629429086	2.3050266922
H	-2.0352568175	3.0725619118	4.5366551131
H	4.1212806581	1.3591663923	9.7327316521
H	5.0477315094	2.7446341605	10.3419661727
H	3.4305570901	0.8950343956	11.9722500191
H	4.4222225426	2.2486623042	12.5195494378
H	2.3016259555	3.2417574381	13.0559946028
H	1.4586563789	2.1912081344	11.8975234577
H	1.4269592774	4.2604712325	10.6577131534
H	3.0334183449	4.7321061455	11.2637587485
H	5.0755287231	4.9127584588	9.5084803609
H	6.5947740905	5.4830827879	7.7236689346
H	3.9162133252	6.1625868861	9.0290468654
H	-0.2636024523	2.9746053522	8.6763231662
H	0.1699449073	2.9132381835	6.9688538794
H	5.9532142746	7.0927783494	8.1042976938
H	-1.6847525429	4.4334809302	6.8056836714
H	-1.440502476	5.034232781	8.4523630778
H	-0.4759446787	6.861176479	7.1246077947
H	0.2628392612	5.7061354717	5.9943046557
H	2.1258151434	6.1362771307	7.4737099411
H	1.0768181177	6.220524164	8.9004558535
H	3.3560481365	4.7811165084	5.7302242836
H	4.2866891202	6.9194255587	6.3070533288
H	5.7191246774	6.2148776791	5.5397344847
H	4.9061548286	3.9530968486	5.8915487716
H	6.3390722011	-2.7254501784	3.0669005047
H	3.9987019402	-1.9356006685	3.2983394354
H	6.1756535632	-3.7185530783	4.5294239982
H	7.1532029443	-1.7158214563	5.4822250102
H	6.2935526788	-0.6700450909	4.3434941229
H	6.4652273776	-3.7589343369	7.4070482547
H	7.4698438834	-4.6847262806	9.4099833242
H	7.7462905298	-3.0728703688	10.0931550594
H	5.0462223288	-4.507811816	9.8552681529
H	5.7503579934	-3.8056336157	11.3245427102
H	3.9755364196	-2.3251379213	9.8419576136
H	5.4161756409	-1.5387169543	10.5075342371
H	3.8660169858	-3.2470144989	6.0518961453
H	3.8587865438	-3.663762854	3.6731807288
H	6.3696164274	0.5817503783	9.4679324579
H	5.3491670504	1.501514247	6.1843119423
H	8.039826288	2.3214102263	9.2976774707
H	6.0414122811	2.9001650019	7.9625378334
H	7.3638225517	3.2619887666	6.8388028392
H	8.803106789	1.5519568606	7.8913166011
H	7.0148275474	1.1942297842	5.6389243294
H	7.7843867463	-0.3696182284	8.9750603684
H	7.5828548413	-2.4407288406	7.7963670004
H	2.9821561658	-1.8174870853	5.4724240993

1bbH^+

C -0.0392393563 0.6027947358 0.2709163862
N 0.0356936848 0.3485741031 1.7232373897
C 1.4443361985 0.2642058468 2.1797642503
C 2.2693763884 0.5321612354 0.9082508826
C 1.3244063206 0.1121834281 -0.2278156959
P -1.1805845217 0.6080102797 2.8521753135
N -2.5658803806 0.5481065698 1.8876781791
C -2.9675506865 -0.800979857 1.3978974448
C -4.4520662279 -0.9835235369 1.8064329866
C -4.695997568 0.1119755975 2.8581619615
C -3.7854175088 1.2410283683 2.3616212469
N -1.2559177922 -0.4257531154 4.0409189666
P 0.0070664403 -0.8328485648 5.1012402359
N 0.3860157706 -2.4623568275 4.8645401005
P -0.4253134497 -3.7759705235 4.5405698241
N 0.4212908816 -5.0434382538 5.2510540339
C 0.9364352833 -4.9436238287 6.6375846794
C 0.9838584799 -6.4003914912 7.1256591054
C 1.1526651837 -7.2060549598 5.8288867996
C 0.2494856584 -6.4486406795 4.8472161349
C -0.9488223635 -0.9334954675 6.7226239552
C -0.3914763807 -1.4768756214 7.940380677
C -1.3040838451 -1.6930173816 9.0425508677
C -2.6435326002 -1.2170984139 8.978369511
C -3.1000578152 -0.5756536795 7.8539483353
C -2.2545754775 -0.4706538357 6.728057829
C -0.8878903997 -2.3882249561 10.2103674989
C 0.4004719918 -2.8451146373 10.3347167186
C 1.3365912949 -2.5349707527 9.3277020318
C 0.9909948025 -1.8431992056 8.1736352553
P 2.4770889454 -1.2843839681 7.2283786359
N 2.8128128492 0.2607041117 7.5464315334
P 2.9275291577 1.2283667831 8.7951686941
N 1.5884088216 2.210050671 8.8638160781
C 0.2636173746 1.8137811282 8.3315393913
C -0.6171488596 3.0561318633 8.5551500208
C 0.3851817117 4.2174602626 8.6437184898
C 1.5706629182 3.5864477206 9.3848582278
N 3.6194197008 -2.3818813064 7.5848927357
P 4.9084280937 -2.8065005242 6.7586183991
N 4.6974001295 -2.7831937024 5.1036543419
C 5.7760776237 -2.5688228897 4.1039145363
C 5.4105380617 -3.5101760105 2.9487925414
C 3.8777230716 -3.5531087396 3.0164273083
C 3.5963085639 -3.6025262604 4.5205309334
N 5.3045885923 -4.3297355116 7.2886414161
C 6.3027510587 -5.1501545431 6.5739918501
C 6.2994020782 -6.4824320366 7.3445425409
C 4.8742214784 -6.5561836455 7.9165159681
C 4.596664672 -5.1050997696 8.3258403744

N	6.2966560438	-1.8935135062	6.9782128044
C	6.3386433273	-0.4810197527	6.5094105753
C	7.0587888572	0.2778495208	7.6349425398
C	7.9381730212	-0.8004457826	8.2841011447
C	7.0282168483	-2.0328267696	8.2618133115
N	-1.0092056975	2.1873768172	3.3780980124
C	-0.5849609332	3.2891429945	2.4911470065
C	-0.6285764747	4.534886496	3.3970583926
C	-0.446317867	3.9604051939	4.8120319679
C	-1.2518591141	2.6580635286	4.7510849303
N	3.1224018544	0.5568582797	10.3087306257
C	2.028892485	0.4754433387	11.3103854984
C	2.5407525479	-0.5552017825	12.3372319378
C	4.0647741794	-0.5818533486	12.1309395273
C	4.2005370978	-0.401554038	10.6164404422
N	4.2817666155	2.1862238506	8.6111623705
C	5.005678121	2.8500417348	9.7185989452
C	5.9981723814	3.7707919857	8.9923956986
C	5.243635283	4.1657171796	7.7132832541
C	4.5295553574	2.865294068	7.3171364028
N	-2.0267410196	-3.9886928137	5.0250681009
C	-2.3942951385	-4.4004753356	6.3920268999
C	-3.9243006239	-4.4503451096	6.333022333
C	-4.2647964642	-3.245303929	5.4399223324
C	-3.1288888346	-3.2136990585	4.3994332033
N	-0.5275455327	-4.0603221977	2.895485387
C	-1.333806989	-5.0917358143	2.2296415853
C	-1.7463759391	-4.4127775157	0.9197175892
C	-0.4793896725	-3.6218452202	0.5428546722
C	0.14759516	-3.2056674535	1.8961814891
H	-2.1722617104	-5.3963033735	2.8604862476
H	-2.6471068497	-0.0627045556	5.8010137473
H	2.3684261661	-2.8425090098	9.458659845
H	-4.1185674241	-0.1970073885	7.8059956961
H	0.711906324	-3.4091269354	11.209937369
H	-3.292751268	-1.3727586517	9.8369907683
H	-1.6168362472	-2.5603234923	10.9993225711
H	-4.3817930636	-4.3925846443	7.3261062224
H	-4.249135264	-5.3854312621	5.8595797081
H	-1.9441471155	-5.3684283783	6.6347537674
H	-2.0654299379	-3.6691663425	7.1450121833
H	-5.2518274963	-3.3227362093	4.9721800245
H	-4.2465550311	-2.3284362822	6.0375388124
H	-2.8129781194	-2.1931052569	4.1622045448
H	-3.4337555381	-3.7027312957	3.4647523776
H	-0.7973455664	-6.7817389539	4.9444518972
H	0.036121382	-6.6686402645	7.6094759427
H	1.7852324294	-6.5704838969	7.851843499
H	0.5511146325	-6.5904890191	3.8050252171
H	2.194734308	-7.1580133399	5.4856586152
H	0.8755924868	-8.2603469272	5.9282872256

H	1.9297089904	-4.4824152668	6.6372867161
H	-0.7331473119	-5.9904452021	2.0152026839
H	0.299916765	-4.3215039377	7.2702759651
H	-2.5867940728	-3.7344493688	1.1059956457
H	-2.0503925653	-5.1270628687	0.1474104981
H	0.209118336	-4.2680305706	-0.0146501705
H	1.2326575673	-3.363024108	1.8977060671
H	-0.6940957535	-2.7512109739	-0.0856276332
H	-0.0381442743	-2.1560225971	2.1223228341
H	1.6500457008	-0.7309050907	2.5939352913
H	1.6525471966	0.9916373967	2.9739263524
H	2.4997809094	1.6017148217	0.8239060195
H	3.2185293277	-0.013829117	0.9087694845
H	1.593590611	0.5402506819	-1.1986445579
H	1.3112981012	-0.9807951726	-0.3267886233
H	-0.1753884032	1.6720045156	0.0477385596
H	-0.8739135139	0.0615457572	-0.179183643
H	-0.9285400541	1.9131625011	5.4790293436
H	-2.8424147796	-0.8622568111	0.3100278055
H	-2.3332822645	-1.5640129888	1.8548166907
H	-4.6473323394	-1.9904300935	2.1877663397
H	-5.1059490994	-0.8258191588	0.9406701078
H	-3.5430651221	1.984757989	3.1236320952
H	-4.2480439262	1.7687946088	1.5154579029
H	-5.747773818	0.4086961788	2.9317422702
H	-4.3555964095	-0.2228443238	3.8450523757
H	-1.2474319238	3.3869820913	1.6217389819
H	-0.7973742157	4.6366103862	5.5979901009
H	0.4300680726	3.0994961927	2.1156947583
H	0.6100139195	3.7305239149	5.0024176436
H	0.1317158281	5.2733563056	3.1239679799
H	-1.6088052549	5.0211357175	3.3190467294
H	-2.3221562722	2.8572425515	4.9290782773
H	4.058338908	-1.3501579932	10.0806000898
H	5.1739666403	0.0010285008	10.3181330565
H	4.529624103	-1.506844053	12.4870055114
H	4.538942244	0.2603216022	12.6499936735
H	2.2495024516	-0.2953165003	13.3594152722
H	2.1186443259	-1.5393762424	12.1095816219
H	1.0845015708	0.151314327	10.8597616894
H	1.8691400732	1.4605688361	11.7648596172
H	5.5095949186	2.1153088847	10.3540846267
H	6.9065748559	3.2128436032	8.7354934096
H	4.328784002	3.4284630413	10.3618947865
H	-0.1373894951	0.9419386422	8.8546557283
H	0.3592135777	1.5492185568	7.2746548284
H	6.291330926	4.627065709	9.6072866216
H	-1.3536035514	3.1797234056	7.7554900701
H	-1.1666068464	2.9630019426	9.4996530235
H	-0.013769215	5.0966924729	9.1592872234
H	0.7037453184	4.523137069	7.6395387741

H 2.5148469576 4.0948554403 9.1744400395
H 1.4093108824 3.6034191624 10.4746411657
H 3.591423286 3.0401098464 6.7794319961
H 4.5086267536 4.9496928628 7.9354212787
H 5.9004883307 4.5389751239 6.9213779455
H 5.1644119993 2.2385690872 6.6789882703
H 5.7986568094 -3.1557168433 1.9884455667
H 3.4512312736 -2.6355116658 2.5912286996
H 5.8233946074 -4.510997773 3.1299245373
H 6.7627393631 -2.7707175198 4.5299586468
H 5.7605009918 -1.5233994757 3.7682559416
H 5.9999047688 -5.2946108236 5.5269396149
H 6.5582189785 -7.3313776138 6.7046256344
H 7.0288260588 -6.4440599519 8.162994212
H 4.1672753367 -6.8626022002 7.1348858194
H 4.7802380104 -7.2550422587 8.7536113453
H 3.5360950578 -4.8485296444 8.3421068035
H 5.0138573055 -4.8912500451 9.3216681045
H 3.6518267387 -4.6380914578 4.8907821704
H 3.4387140769 -4.4054125989 2.4889989498
H 6.339534846 -2.0172008748 9.119871539
H 5.3313491677 -0.089434622 6.3298857376
H 8.2629896014 -0.5449296605 9.297913136
H 6.3209370661 0.6534588371 8.3508255542
H 7.6296410952 1.1325868119 7.2578832234
H 8.8333248378 -0.9828414824 7.6767971184
H 6.9018172014 -0.4253319744 5.5700180607
H 7.5800716008 -2.977242678 8.2871532954
H 7.2888308183 -4.6688997758 6.5703353659
H 2.6117941809 -3.2029109696 4.7700685072
H 2.2190559472 -1.3043986178 5.8652757365

2aa
C -0.0915333407 0.1695278152 0.0014464626
C -0.0816243774 0.1236755182 1.3889432422
C 1.1174826733 0.0610315854 2.150890434
C 2.3530953304 0.284139769 1.4821332864
C 2.3148707172 0.3018822291 0.0728186532
C 1.134881503 0.2102773322 -0.6661676265
C 0.7097098127 -0.1529975469 3.5761938469
C -0.6919985865 0.0469912612 3.6585819644
C -1.2887277177 0.2072247486 2.2864159209
C -1.3866062753 -0.011229382 4.8611488093
C -0.6848927891 -0.3528761988 6.0215251359
C 0.6678479923 -0.6869832893 5.9339420614
C 1.3874421128 -0.627012274 4.7269885713
P 3.1690143571 -1.1573579361 4.6840197981
N 3.101568116 -2.4723274451 3.6302646395
P 2.351564575 -3.8345387546 3.5108048621
N 1.962049737 -4.0813012026 1.884149127
C 1.2189740857 -5.231448605 1.3937338044

P	4.0429040777	0.5821915985	2.2895797724
N	3.7413417752	1.9090110864	3.300390644
P	3.1077971228	3.3280025001	3.116624529
N	2.3617490604	3.8608705134	1.6982568586
C	0.972542778	3.50674789	1.3978545139
N	4.830793818	1.2363476762	0.8877642324
P	6.3632628761	1.0965439437	0.6082024154
N	6.6746839059	1.7400658277	-0.9380601071
C	8.0084020786	1.5381278555	-1.4990156528
N	7.4876912883	1.9780964519	1.5498903094
C	7.3992953339	3.4425059084	1.4256349572
N	6.9773151405	-0.4785896122	0.7631239982
C	8.3886820423	-0.8272569253	0.8627891689
C	7.5579575454	1.5702376613	2.9608602947
N	3.327369212	-1.7661235563	6.2874444242
P	4.7134039571	-1.6140978335	7.0181551904
N	6.0271858789	-0.8257047005	6.2691235754
C	5.9569981309	0.6390344982	6.1761761419
N	4.4721720407	-0.7938759646	8.4833763179
C	3.4352071863	0.2264132694	8.611038511
N	5.3978511539	-3.1236439362	7.3509609196
C	6.7782670587	-3.3585167845	7.7516626717
C	5.5297869902	-0.6752466255	9.4771177007
N	3.3328627798	-5.0982106543	4.1173150922
C	4.7800846432	-4.9162903796	4.0439571294
N	0.8851243802	-4.2028956759	4.2812179584
C	-0.3448088572	-3.5631508602	3.8011392948
C	2.9322089695	-6.4919221093	3.9507795367
C	0.87122977	-4.4176988573	5.7318087145
C	2.484503756	-3.2115672665	0.8351732362
N	4.3308577436	4.5240147442	3.3023306458
C	5.2491482308	4.3187948229	4.4218926958
N	1.8962048989	3.5534229501	4.2763788164
C	1.8053890527	2.6798954725	5.4438965086
C	4.0586416428	5.9430413235	3.0820012262
C	1.223294302	4.8343239259	4.4441758017
C	3.1681879723	4.0431717266	0.4879651951
C	6.1067812379	-1.6343684275	0.5546455812
C	5.6142331745	1.6939218918	-1.942380928
C	4.5023998713	-4.2421969351	7.6310337059
C	6.5984900046	-1.4193806794	5.050843652
H	-1.0332734132	0.1903085067	-0.5438148345
H	3.2548261686	0.4387822112	-0.4491392231
H	1.1892348915	-1.055847771	6.8120462397
H	5.4206612886	3.2529736855	4.5680674983
H	1.7474540345	-4.9917728556	6.0351591634
H	4.2080101345	4.2468641165	0.7451700776
H	3.0414246177	-2.3938582261	1.2876467772
H	6.2093517864	4.8060509102	4.2055710992
H	0.8675795506	-3.4694031721	6.2856688301
H	3.1506534595	3.1403558823	-0.1335215656

H	3.1456964773	-3.7737173142	0.1558881972
H	2.7667865926	4.8904261297	-0.0866716343
H	1.6609949886	-2.7835879972	0.2460501033
H	3.3438988271	6.0775642168	2.2690577119
H	-0.2468235709	-3.2869477059	2.7496576113
H	3.6620282583	6.440722841	3.9830834693
H	-1.1810921964	-4.2692014924	3.9032339306
H	4.9952793073	6.4475603708	2.8066049269
H	-0.5777169452	-2.6585193765	4.3734181854
H	0.4718182952	4.3709895515	0.9373186317
H	0.4222576791	-4.8976392014	0.7114620832
H	0.9199997733	2.6604697772	0.7045775121
H	1.8666949818	-5.9284357338	0.83773529
H	0.4403071406	3.2355978752	2.3103365236
H	0.7494897659	-5.7711013986	2.2176963097
H	1.259477746	5.4155067031	3.5204052427
H	1.8558708001	-6.6004033304	4.1000957604
H	1.6717217833	5.4332546487	5.2543612314
H	3.4444889042	-7.1062514319	4.703466543
H	0.1675494323	4.663255403	4.6980466898
H	3.1928990571	-6.8903929952	2.9550926882
H	0.7661449813	2.3637686148	5.6011287357
H	5.1891574743	-5.2143803553	3.0620817273
H	2.1576601254	3.1993001299	6.3510181599
H	5.2600716533	-5.5339423286	4.8144775109
H	6.4846469124	3.8422705015	1.8884553197
H	2.9046842658	0.0988727481	9.5651185602
H	7.4142707212	3.7294117095	0.3727917025
H	2.7192663647	0.1323981309	7.7956899351
H	8.2711688434	3.8853878232	1.9231193236
H	3.8603838352	1.2440331465	8.5911508645
H	6.6607677521	1.8651355476	3.5190760872
H	5.0847964201	-0.6355859215	10.4805986422
H	8.4344125564	2.0431727843	3.4210976028
H	6.1341379893	0.2373820992	9.3370985868
H	7.6636133034	0.4885387826	3.0458079141
H	6.1987068888	-1.5350771946	9.4436558932
H	8.9949587641	0.0631955372	1.0331406622
H	7.3876457843	-2.4726230523	7.5653114044
H	8.5463336044	-1.5205034606	1.7036368818
H	6.8540276778	-3.626634827	8.818630999
H	8.7439975057	-1.3245260884	-0.0538570789
H	7.1942952242	-4.1946469353	7.1698650447
H	6.0936942842	-2.2746690325	1.4473609411
H	4.399434066	-4.4164003092	8.7147678266
H	8.1400197493	0.5278125541	-1.9234535995
H	6.9672115829	1.0238376513	5.98303487
H	6.4560285132	-2.2291665775	-0.3043430329
H	4.9026333524	-5.1556235129	7.1711894016
H	8.1750362166	2.2678857635	-2.3013803262
H	5.2871955807	0.9685662792	5.3693525684

H 5.0828032198 -1.3165511839 0.3618217315
H 3.5218200846 -4.0393519665 7.2006507277
H 5.5426767086 0.7091416973 -2.4365329608
H 7.6432069311 -1.0910065954 4.9576015373
H 5.823244222 2.4468888005 -2.7134881985
H 6.0460616855 -1.1189532241 4.1495250488
H 4.6579339597 1.9228585843 -1.4730330004
H 6.5885631575 -2.508569395 5.1223467717
H 8.7696999591 1.6969486475 -0.7316551357
H 5.6133931308 1.0656204392 7.1210166219
H 1.1727612508 0.2294102155 -1.7535562478
H -1.2006137137 -0.4172247343 6.9776612045
H 2.4145198905 1.7934601263 5.2820543958
H 5.0241324789 -3.8710555057 4.2237459286
H 4.8541670698 4.7450568577 5.3612673681
H -0.0310742265 -4.9863379101 5.9931381164
H -2.4592738602 0.1714237422 4.8870321795
H -1.8062500545 1.1688654214 2.1534241948
H -2.0299408516 -0.5791069369 2.0840595585

2aaH$^+$
C -0.0988657052 0.0517482467 0.036840089
C -0.0797600378 0.039710899 1.4250929025
C 1.1231273493 0.0089900691 2.1801471413
C 2.3359951987 0.2326450924 1.4729479831
C 2.3003107764 0.229943638 0.0637126001
C 1.1135702561 0.0945687641 -0.654820325
C 0.7422981456 -0.1895751367 3.6128361957
C -0.6608154196 0.0059973544 3.700891146
C -1.2763762153 0.1210129067 2.3332334267
C -1.3456649854 -0.0300338334 4.9098920666
C -0.6336310096 -0.3523105576 6.0672115581
C 0.7185769652 -0.6907362423 5.9750592061
C 1.4337329792 -0.6469469578 4.7658453961
P 3.2129175975 -1.2108911127 4.7538000091
N 3.16699805 -2.4143208436 3.5671956278
P 2.4083048661 -3.7960190949 3.4793659905
N 0.9496578007 -4.1235689185 4.2586255658
C -0.3045647395 -3.552981285 3.7568342548
P 3.9152422787 0.7933932305 2.2038072368
N 3.6781978275 2.0124742318 3.239571051
P 2.9933278135 3.431988805 3.0279800259
N 1.7432547797 3.5849318414 4.1276563279
C 1.6277819437 2.7132576198 5.3010840109
N 4.895696458 1.095755253 0.9348999132
P 6.4713206373 0.9217772148 0.8710205212
N 6.9397750221 -0.6315114508 1.3237776881
C 8.3295925084 -1.0151367728 1.5863557417
N 6.9374872851 1.3752893694 -0.6758247738
C 8.3262298284 1.1350464058 -1.0785153314
N 7.4410112907 1.887829771 1.8588050031

C	7.4511421925	3.3321775281	1.5670133342
C	5.9840722064	1.3670640696	-1.7879362596
N	3.3343785815	-1.8948614025	6.3031082392
P	4.6659573852	-1.7977368769	7.1464368712
N	5.2589921423	-3.3261375688	7.5217003814
C	6.6137417164	-3.6221160428	7.9799038149
N	6.054422479	-1.0619218978	6.4784276206
C	6.1045059453	0.4019482339	6.4515061604
N	4.3493128909	-0.9649421623	8.5791917399
C	3.3938328315	0.1396462006	8.6310305904
C	6.6819246017	-1.670090548	5.3024822694
N	1.980198443	-4.0407761733	1.8578291226
C	1.3870152459	-5.3031486851	1.4222684135
N	3.3937064108	-5.0353064278	4.0935865369
C	4.843023892	-4.8662565733	4.0944331903
C	2.6934808807	-3.33791707	0.7961696311
C	2.9831201927	-6.438073922	4.0981905288
C	0.9080952112	-4.3889852058	5.6992139664
N	2.3177225252	3.8442579442	1.554679886
C	0.9363748287	3.4877230125	1.2090533768
N	4.1910593854	4.6196567117	3.2301682822
C	5.0444359826	4.4752251604	4.4163669796
C	3.1791561621	4.0186744333	0.3776302111
C	3.9177276458	6.0303073414	2.9287287243
C	1.0201598366	4.8512559254	4.2557931772
C	7.3924031413	1.6278053507	3.3065307895
C	6.0823497623	-1.7802323901	1.0043874254
C	5.2833831825	-0.9803614475	9.7003346288
C	4.3013835239	-4.3990258835	7.785905272
H	-1.0463043825	0.042928784	-0.4972730238
H	3.2326805917	0.371707422	-0.4703183351
H	1.2385084274	-1.0589607195	6.8534639934
H	5.195424922	3.4207366188	4.6463216498
H	1.8136710641	-4.9038156844	6.0167267535
H	4.2026383591	4.2441437244	0.6794151629
H	3.0573113813	-2.3874372543	1.1776730291
H	6.0198616303	4.9363946181	4.2206673627
H	0.8159311615	-3.4612095537	6.2777110079
H	3.1958858892	3.1059619617	-0.2307487236
H	3.5431516143	-3.926768871	0.4100979944
H	2.796075992	4.8479394057	-0.2303414094
H	2.0049149888	-3.1412410862	-0.0355689944
H	3.2567898716	6.1230108703	2.0665168567
H	-0.1949255147	-3.2487717599	2.7150305867
H	3.459835862	6.554791824	3.7812035617
H	-1.09844921	-4.3095269009	3.8226788287
H	4.8658191336	6.5289341526	2.6935926878
H	-0.6042686448	-2.6805828642	4.3480265257
H	0.4599733741	4.3390781702	0.7060560551
H	0.7342583744	-5.1149134856	0.5601853244
H	0.9131389855	2.6225651123	0.5382347112

H	2.1484088794	-6.0386340126	1.1171156289
H	0.3680912216	3.2425833392	2.1062818295
H	0.7780881544	-5.7371923063	2.217243212
H	1.0805391216	5.4306107855	3.3321287426
H	1.8965827908	-6.5234200174	4.1438470588
H	1.4151422535	5.4608966419	5.0819097722
H	3.4014297793	-6.9357113006	4.983604313
H	-0.037693506	4.6441693166	4.459717587
H	3.341922156	-6.9756589953	3.2065580653
H	0.5949120529	2.3611324268	5.4031443535
H	5.3105981698	-5.3579253279	3.2248501209
H	1.9111741785	3.2573488471	6.2143795777
H	5.2661364353	-5.3093763147	5.0055816348
H	6.5369173648	3.8256646631	1.9291566294
H	2.724018556	0.0134258317	9.4921411966
H	7.5432940801	3.4994480941	0.4931412016
H	2.7928875138	0.1618997672	7.7230096119
H	8.3173247553	3.7801553846	2.0663661264
H	3.9064135395	1.109362734	8.7382518898
H	6.4774020529	2.0288223431	3.757754252
H	4.7217174251	-0.9054275494	10.6397934056
H	8.2585168535	2.1055710172	3.7778564767
H	5.9953391399	-0.1397271901	9.6601407957
H	7.4324841627	0.5593442584	3.5140704773
H	5.8500185773	-1.9108372399	9.7256457101
H	8.9419205731	-0.1390083453	1.80143882
H	7.2635123751	-2.7554709941	7.8506848906
H	8.362453458	-1.6858894207	2.4550228803
H	6.6238385101	-3.9179103902	9.0401131991
H	8.7650258055	-1.5454696487	0.727307677
H	7.0252728387	-4.4588853329	7.3975022212
H	5.9297896723	-2.3974677991	1.8976925662
H	4.183037016	-4.5730181361	8.8663480867
H	8.468896876	0.1224846997	-1.4861582899
H	7.1466193212	0.7156406117	6.3081916361
H	6.5382181344	-2.3970452774	0.2166817385
H	4.6553504519	-5.3298263786	7.3235659201
H	8.6050431014	1.8592123125	-1.8523761043
H	5.4911941458	0.8239098151	5.6414479275
H	5.1043393353	-1.4505091661	0.6571545066
H	3.3355494011	-4.1410777141	7.3537891358
H	5.9197211353	0.3797977176	-2.2712561128
H	7.7404774897	-1.3777362832	5.2710649591
H	6.3137270137	2.0958732668	-2.537678615
H	6.2001054964	-1.3565829849	4.3644791392
H	4.9962186506	1.6547794928	-1.429409677
H	6.6307161615	-2.7579519111	5.3677577618
H	8.9984734346	1.272941738	-0.2288389867
H	5.7624180657	0.8129905709	7.4029638477
H	1.1339214478	0.0837115585	-1.7411385539
H	-1.1416818661	-0.4053942988	7.0272660334

H 2.2748883266 1.8474541962 5.1851652462
H 5.0842052032 -3.8053280935 4.0753585566
H 4.601000929 4.9680057779 5.2963384035
H 0.0448352894 -5.0317856046 5.9139990718
H -2.4183114652 0.1467838216 4.9416125151
H -2.0009305776 -0.6865101309 2.1597827044
H -1.8171406911 1.065671933 2.1780886361
H 4.4383829888 -0.229842762 2.9959222433

2bb
C -0.7473230769 1.7431887395 0.8389074141
N -0.4653199881 1.3195795293 2.233989798
C 0.8738423946 1.7420943433 2.6669923606
C 1.607257577 2.0090636079 1.3457157799
C 0.4917865977 2.5655923365 0.4476782115
P -1.5664944362 0.4940682758 3.2008735987
N -0.6652099814 -0.8198323592 3.7940412871
C -1.3235180095 -1.6862454923 4.7938879843
C -2.0253080085 -2.799907192 3.9825329923
C -1.200266641 -2.8995551609 2.669989297
C -0.1516684981 -1.7597792057 2.7762433144
N -2.8679949335 0.0499996117 2.4300948712
P -3.9778023741 1.2266751125 1.8275144947
N -5.4615242383 0.8696825777 2.5117953531
P -6.5138079062 -0.2500307082 2.7373653752
N -6.2497604588 -1.086378075 4.166672978
C -5.1649519787 -0.6956483217 5.0941644533
C -5.0698493862 -1.8775937236 6.0877162897
C -5.7966316915 -3.038235363 5.3840632341
C -6.9101584207 -2.3121401587 4.6190175118
C -4.1624299178 0.6864460125 0.0618190744
C -4.9703382177 1.3796125696 -0.8777207189
C -5.0743361915 0.8437779853 -2.1884689894
C -4.3062370201 -0.2271054388 -2.629198259
C -3.4238161756 -0.8325553246 -1.7289054799
C -3.3821953241 -0.387778889 -0.408374434
C -5.8138569439 2.6253040368 -0.8279027943
C -6.5195829647 2.6906061047 -2.0635305949
C -6.1070218076 1.5814809132 -2.9924565744
C -7.4152764072 3.7037142083 -2.3764868234
C -7.6030988043 4.7319614888 -1.451094903
C -6.8622748956 4.7313241424 -0.2705621125
C -5.9431765986 3.7219366895 0.0760127916
P -4.9813966356 3.9774768111 1.6920641158
N -3.4728517847 4.3773528803 1.0725847375
P -2.5849237714 5.386623516 0.3114903043
N -3.2050211743 6.8265195589 -0.346549389
C -3.8562840826 7.7885257499 0.5774219542
C -5.1262323094 8.2484459277 -0.1552592089
C -4.7841044571 8.0330731705 -1.6374431134
C -3.981904868 6.7280761268 -1.605887772

N	-5.6930761639	5.4637646866	2.2150719771
P	-6.1705426583	5.8502163642	3.6541968766
N	-7.8284900013	6.1643477983	3.6604808698
C	-8.742750139	5.739355233	2.5816353877
C	-10.0517104448	5.4268739037	3.3182360415
C	-10.0350864537	6.4277787696	4.4840059297
C	-8.5596094384	6.4171775213	4.9131458389
N	-5.5247051707	7.2981423295	4.2601890689
C	-5.9944945808	8.5764742652	3.6883466743
C	-4.9859241758	9.5867807499	4.2421530631
C	-3.6582892836	8.8147209159	4.160009637
C	-4.0559289955	7.3563093671	4.4719640629
N	-5.8309380171	4.7812902324	4.9291615354
C	-5.6499574441	5.1860877723	6.3451386811
C	-6.3661024339	4.0986395871	7.1610716176
C	-6.2445425893	2.8594343894	6.2632047576
C	-6.4485924914	3.4309493502	4.8574271631
N	-1.8340934465	1.3582206105	4.6306846809
C	-0.7942231963	1.5980037813	5.6540487566
C	-0.8649371397	3.1066123198	5.9217794315
C	-2.3641372082	3.3978167256	5.7610468438
C	-2.7722170491	2.5157720773	4.5726138048
N	-1.8439085674	4.6374081281	-1.0021173493
C	-2.5330360125	3.5932165412	-1.7943433833
C	-1.9959253925	3.796738308	-3.2166112229
C	-0.5740867914	4.3295343251	-2.9784462417
C	-0.7481624111	5.2560440415	-1.7629145055
N	-1.3830718982	5.9607696818	1.3376527741
C	-0.5073025752	7.1219736812	1.1428151782
C	-0.073887849	7.4693967595	2.5729088657
C	0.0662306381	6.0797364605	3.2168003357
C	-1.1110924245	5.2857363027	2.6241989621
N	-6.7477891772	-1.5153413844	1.6345039545
C	-7.441500582	-1.2374975733	0.3520117669
C	-6.8029099848	-2.2086841841	-0.6603828039
C	-6.1718275923	-3.3044217764	0.2138751039
C	-5.6811965243	-2.5195572449	1.4338361508
N	-8.0140113125	0.5074962294	2.7977059067
C	-8.2887138793	1.7493600597	2.0382294081
C	-9.8065443892	1.7099354276	1.8132984346
C	-10.3225190694	0.9251849488	3.0300706696
C	-9.2530887497	-0.164400446	3.2073123819
H	1.3518175254	0.9620982592	3.2698896375
H	0.7157459294	2.4812540105	-0.6201752312
H	0.3175858803	3.6255794888	0.6616116836
H	0.8307779034	2.6605774198	3.2754411375
H	1.9891232718	1.0639103247	0.9381232597
H	2.4543089877	2.6938674027	1.4656676523
H	-0.8720278516	0.8665864342	0.1910198176
H	-1.660581934	2.3398215174	0.7833326244
H	-0.4668990001	3.382677943	6.9049947283

H	-2.6702944952	3.0645468453	3.630936919
H	-2.5896563433	4.4518441291	5.5726276638
H	-2.9032290981	3.097942754	6.6695282958
H	-3.807265297	2.1735492591	4.6296245813
H	-2.8023530756	-1.6691663427	-2.0432301808
H	-2.7378352936	-0.8862669924	0.3107946008
H	0.8108051908	-2.1518325146	3.1341699876
H	0.0282100978	-1.2421460947	1.8304723936
H	-0.7139774956	-3.8746970687	2.5516419994
H	-1.8496394147	-2.735775591	1.8055053751
H	-2.0038850618	-1.1040759711	5.4185965604
H	-0.5419018217	-2.108458274	5.4420871714
H	-4.7104037809	-2.0465992526	1.2335505351
H	-6.0208326421	-1.6905635343	-1.2210398023
H	-6.9270955866	-4.0402262359	0.520712686
H	-5.359450478	-3.8377355612	-0.2918702354
H	-7.5338452753	-2.5972933886	-1.3783062746
H	-5.5714405006	-3.1444764986	2.3259363353
H	-4.241096296	-0.5402510796	4.5325788831
H	-7.3084655901	-2.8744077584	3.7697858174
H	-7.7521429172	-2.0910695561	5.2978377174
H	-5.1238767199	-3.5337557351	4.6736946728
H	-6.1774582013	-3.7939505987	6.080406838
H	-5.5864327776	-1.6307138385	7.0240006279
H	-5.398523193	0.2409719089	5.6126925769
H	-9.1847886726	-0.5175869206	4.242730284
H	-9.476306183	-1.0378810656	2.5721128347
H	-11.3267675297	0.5095421983	2.8929094116
H	-10.3413860589	1.5751399725	3.9152654073
H	-10.2385374205	2.7124473533	1.725146913
H	-10.0359994958	1.1626104657	0.8898475773
H	-7.9914538489	2.6253736014	2.6253308443
H	-7.7248971799	1.7941662773	1.1044062139
H	0.1887443564	1.2583966577	5.3204554427
H	-1.0372686371	1.0351233082	6.56959184
H	-2.0631646578	-3.7449042176	4.5367423773
H	-4.0313886283	-2.1179108024	6.3382430647
H	-7.308922418	-0.1990370416	0.0274239014
H	-8.5184592968	-1.4181345781	0.4714019908
H	-3.048367407	-2.4923192993	3.7524473186
H	-0.2935441946	3.6488211192	5.157243548
H	-8.2436644982	7.3607948752	5.3766205911
H	-10.9312407005	5.52189795	2.6725605504
H	-10.023884584	4.3990438139	3.7044706514
H	-8.3787981505	5.6158996212	5.6445834526
H	-10.3166710907	7.4262909598	4.1251612241
H	-10.7101517333	6.1604262709	5.3041187526
H	-8.884578978	6.552461278	1.8532823229
H	-8.3457407938	4.8804781162	2.0389579502
H	-5.9196356231	3.9657567752	8.1529696617
H	-7.5280928613	3.5115583853	4.6440507099

H -6.9694269485 2.0726139491 6.4963065877
H -5.2403789457 2.4294669222 6.3526313097
H -5.995789055 2.8250670478 4.0682997906
H -8.2886649484 5.5508035032 -1.6626837904
H -6.9551442994 5.5668627572 0.4138476736
H -7.0233842438 8.7786880157 4.0005170389
H -5.970670801 8.5692986795 2.5879635183
H -5.2302415653 9.8222951583 5.2858591585
H -4.975442365 10.5230360154 3.6735774613
H -3.531607098 6.6476928979 3.8197269935
H -3.8223807135 7.0909927918 5.5100041391
H -4.1081126135 7.3101825328 1.5274550283
H -5.6687067285 7.9564532544 -2.2788684485
H -5.3968503733 9.283643164 0.0819429592
H -5.9582381033 7.5996060201 0.1379843624
H -4.1585783254 8.8545016112 -2.0115352031
H -3.1775507808 8.6327974428 0.7706703137
H -1.9956902859 5.3310775685 3.2689007118
H -1.0375875692 7.9322269085 0.6354360853
H 0.3757698964 6.8706168278 0.532116525
H -0.8672489951 8.0397910311 3.0725990158
H 0.8477255526 8.0608088391 2.6062584671
H 1.0191900536 5.6248053436 2.9149725078
H -0.875722793 4.2259278581 2.4894543435
H 0.1646441008 5.3153836401 -1.1580694416
H -1.0029403826 6.2803625221 -2.0799489831
H -0.1491168691 4.8517499333 -3.8426236859
H 0.0969526672 3.499892856 -2.7227366463
H -2.0212645717 2.8728858856 -3.804116706
H -2.5967459227 4.5499294717 -3.7430661963
H -2.2856578196 2.5944879526 -1.4153108208
H -3.6172343742 3.6964317016 -1.7361660026
H -6.0416266591 6.1901263751 6.5321181393
H -4.5794706697 5.1967356192 6.5919852769
H -2.8964074943 9.1920924707 4.8504617804
H 0.0398154271 6.1016434625 4.3115288081
H -4.6692492747 5.8702048329 -1.596662539
H -3.3082655812 6.6197225669 -2.4627341937
H -3.2504520859 8.8850294534 3.1464767822
H -7.4231520725 4.3588132664 7.3027412793
H -7.9368375103 3.7001749356 -3.3319075553
H -4.3991139989 -0.5844092705 -3.6531525102
H -5.7058498396 1.9776760274 -3.9362091826
H -6.9471610016 0.9232900201 -3.25974441

2bbH$^+$
C -0.2127125936 -0.1004522023 -0.0458484335
N -0.1651445736 -0.0723259026 1.4261439372
C 1.2210697798 -0.0928788711 1.9284790037
C 2.0677230917 -0.3219840845 0.6665626495
C 1.2118907237 0.3090297055 -0.4444012282

P	-1.4614072549	-0.325450575	2.4582464704
N	-1.4354763685	-1.9733939761	2.7839383009
C	-0.8576588541	-2.9828665027	1.8741715557
C	-1.1379803383	-4.3095565705	2.5926974085
C	-2.4886717133	-4.0480841375	3.277218458
C	-2.3467970545	-2.6018331132	3.7722560301
N	-1.4254400076	0.6400590921	3.6982279578
P	-1.136527575	0.2082928718	5.3094648905
C	-2.8974202962	-0.0536915927	5.9515501231
C	-3.281472344	-1.0305946819	6.9000460919
C	-4.6680438967	-1.2744798607	7.1038581976
C	-5.6556054713	-0.524316775	6.4807032702
C	-5.2707333551	0.4791898319	5.5860698464
C	-3.9175007453	0.6737842756	5.3168762663
C	-4.8898329513	-2.474831351	7.9816339758
C	-3.487792381	-2.9331608515	8.2755449162
C	-2.52972976	-2.0366558745	7.7214386177
C	-3.1359565494	-4.0646921732	8.9969881511
C	-1.7821071599	-4.3400403212	9.2101149647
C	-0.8305332542	-3.4277595393	8.7677906178
C	-1.1713877798	-2.2555615235	8.0597994118
P	0.2728941726	-1.1381895534	7.9371177805
N	0.8724777558	-0.8726736624	9.4279880148
P	0.3164510018	-0.2472922782	10.7743834214
N	1.6112311008	0.2954833963	11.6659498475
C	2.6919378673	1.087320883	11.0401884592
C	2.9994857078	2.1649179699	12.0830500839
C	2.8191106662	1.3974919923	13.4022483669
C	1.5985329824	0.4943241886	13.1368162181
N	-0.7690665596	1.6474163841	6.1055509901
P	-0.5358540244	3.1771817938	5.9368900284
N	-0.3711628256	3.871861543	4.4133869023
C	0.74238429	3.4434543508	3.5318845949
C	0.1658309998	3.5072099417	2.1044812313
C	-1.0211169541	4.4756600246	2.2330317801
C	-1.5897077756	4.1182175647	3.6088741682
N	-1.756601191	4.06166537	6.6840633569
C	-1.6748234138	5.5236991077	6.8889268826
C	-3.1271758307	5.9520958126	7.181449023
C	-3.7925459459	4.6600873991	7.6829866711
C	-3.1480027136	3.5926919238	6.7949200119
N	0.9174785123	3.6053065787	6.6710307684
C	1.226483297	3.015907107	7.993440893
C	2.3564963576	3.9006775627	8.546925116
C	2.0885671302	5.2686023747	7.9003505345
C	1.6266627539	4.8887516841	6.4877206051
N	-2.821740247	-0.0390938711	1.5274367486
C	-3.3682246798	1.318600277	1.3314061892
C	-4.4049441275	1.1386842339	0.2083915526
C	-4.8388978848	-0.3287586762	0.354882399
C	-3.5214509277	-1.0348160334	0.7005982484

N 1.3116298761 -1.8812371468 6.9714356733
P 2.8530662753 -1.7525010264 6.6509753711
N 3.9371758519 -2.4028824699 7.7520262259
C 3.8486623163 -3.8747196085 7.9669658288
C 4.2938506573 -4.0705859299 9.424607326
C 3.8192891302 -2.7788669685 10.106807028
C 4.13738629 -1.7118521242 9.0556778181
N 3.0602560202 -2.6372547086 5.2618991095
C 1.9689355686 -3.2800339587 4.4933565722
C 2.6485796752 -3.647182106 3.1645033275
C 4.1178239942 -3.8716721494 3.5567961128
C 4.3636733719 -2.762951875 4.5892629237
N 3.3887018668 -0.1767948614 6.4817928456
C 4.8060003535 0.2470081275 6.617881112
C 5.0305891711 1.2007612113 5.4377643341
C 3.6464896608 1.8354547016 5.2459387361
C 2.6887759799 0.6606425945 5.4683650583
N -0.4290612613 -1.3123508513 11.8338754438
C 0.244977564 -2.5531081257 12.2563637286
C -0.7316865412 -3.1105018772 13.294105627
C -2.1074566694 -2.8149883395 12.6631154459
C -1.8954729629 -1.5424771382 11.8034893928
N -0.7562637799 1.0174019909 10.5381472126
C -1.0318789327 2.0172665473 11.5954219108
C -2.1544470186 2.8737735106 10.9956452101
C -2.9584418467 1.8449984086 10.1902601538
C -1.8595567075 1.0029454471 9.5350944671
H -5.4267818788 -2.2273693781 8.9088637142
H -3.9072479705 -4.7259287883 9.3856277846
H -5.4844255528 -3.2451412962 7.4719159157
H -6.7063600547 -0.7380458953 6.6639364307
H -6.0221723706 1.0793883759 5.0773582517
H -3.623133609 1.3991792819 4.5638617399
H 0.2169446837 -3.6054236323 8.9966186327
H -1.4748521306 -5.2336270726 9.7470595566
H -0.131465715 2.595893538 11.824021324
H 2.2693217159 2.9812026257 12.0100861911
H 0.6718209417 0.9747309583 13.4673388518
H -2.7391050577 3.3884715552 11.764418359
H -1.7316773948 3.629132902 10.3208168292
H -1.3731298959 1.5368231081 12.5255055629
H 0.3648731493 -3.2501538334 11.4133038368
H -3.5611090869 1.2253972695 10.8666651292
H -3.6316925863 2.2884144496 9.4531016927
H -2.2031884812 -0.0100341604 9.3220031087
H -1.5238264895 1.4521478225 8.5948134113
H 2.7365748807 -2.8004974918 10.2748487092
H 1.2389284643 -2.3358650533 12.6570827711
H -0.5747704608 -4.175606674 13.492091166
H -0.6125735403 -2.5661300264 14.2382995646
H -2.891756272 -2.6766885519 13.413413116

H -2.4107103778 -3.6438694317 12.0156490237
H -2.4095288155 -0.6663215703 12.2119313397
H -2.2659726669 -1.7121274699 10.7850961945
H 1.6743499865 -0.4623522492 13.6634351259
H 2.6665433236 2.0473619719 14.2692257121
H 3.707144314 0.7839258359 13.5975494871
H 3.9997232296 2.5937901276 11.9646254687
H 2.3785249892 1.4843194461 10.0731193312
H 3.5742597772 0.4554633918 10.8744007878
H -0.158976442 0.0915074884 7.4429213697
H 5.1797385647 -1.3733790889 9.1307881545
H 5.4830754983 -0.6114722578 6.6149923354
H 4.9373072381 0.7716315835 7.5744292655
H 5.8230645132 1.9298591113 5.6357273256
H 5.311982494 0.6311547378 4.5424334229
H 5.1545802307 -3.0117051389 5.3058812296
H 1.7053836072 0.9748145267 5.8211436334
H 0.3480903969 3.0442911 8.6539871231
H 1.5217346487 1.9669210858 7.8936562754
H 3.33278876 3.5154388946 8.225702542
H 3.5100133221 2.2991389312 4.2647672453
H 2.3585554553 3.9339185686 9.6409231069
H 1.2805600027 5.7846806376 8.4344316876
H 2.9638076522 5.9265356658 7.8954819275
H 2.4942084447 4.7463754011 5.8264163563
H 0.9872964188 5.6421415511 6.0229925
H -4.8839952937 4.6750678678 7.5996723141
H -3.6493871236 3.5619249648 5.8148451513
H -3.1816444688 6.7785071825 7.8971018156
H 1.467102144 0.8639380101 2.404520295
H -1.0101974646 5.746126742 7.7345613048
H -3.6132598099 6.2823325061 6.2551106874
H -1.2719917343 6.0378535702 6.0066856882
H 3.0678476346 0.1160602962 0.7478825402
H 1.5932452158 4.1253192522 3.6589579067
H 1.3064859128 1.4016988157 -0.4181403128
H 1.481255077 -0.0329121718 -1.4484777993
H -0.1948531564 2.5162113016 1.8140648159
H -0.9765411959 0.5868539763 -0.4236261667
H -0.6760520557 5.5172749665 2.2264486209
H -1.761288684 4.3582110541 1.4344364031
H -2.5824469457 2.0342455911 1.0595146621
H -0.4451154349 -1.1060554666 -0.4309332991
H -2.1828841748 4.9246904428 4.052563522
H -2.2093517636 3.2134751265 3.5391691929
H 0.9105392885 3.8338165238 1.3706029582
H -3.8305999097 1.6758729813 2.2600549521
H -5.2347852735 1.84752855 0.2913085709
H -3.9315744921 1.2904683948 -0.7700667714
H -5.3090795264 -0.7343390275 -0.5464482676
H -5.544831283 -0.4346950644 1.1880551802

H -2.9592487385 -1.2746921509 -0.2170579283
H -3.6707585006 -1.9661388212 1.255071088
H 0.2103069571 -2.8036583175 1.7175556229
H -1.3461863383 -2.9723575053 0.8874995326
H -0.3657994698 -4.4962831448 3.348657468
H -3.3045548683 -2.0724343508 3.8266006532
H -1.153689862 -5.1600816293 1.9037294143
H -1.8941442627 -2.5712537605 4.7704611007
H -2.703689562 -4.7451751262 4.0932000263
H -3.3021457071 -4.1250884549 2.5443807704
H -3.5314501094 4.4839351049 8.7338362554
H 1.1167611582 -2.605208265 4.3749290424
H 1.6107870612 -4.1753362101 5.0193601305
H 2.5803442683 -2.8082134119 2.461377774
H 4.2372758897 -4.8539347371 4.0306683123
H 4.8086691869 -3.8173061253 2.7097203605
H 1.3617298144 -0.8761984738 2.6819385799
H 1.0765414257 2.4329779134 3.7848700843
H 2.5472547649 0.0937613664 4.5372801733
H 4.6439220405 -1.8193930037 4.0940908385
H 5.3868107099 -4.1491158737 9.4807505116
H 4.3140604222 -2.5868888591 11.0649066153
H 3.8696388053 -4.9795645164 9.8630271201
H 2.1857754822 -1.3972137735 0.4791136793
H 2.816788006 -4.2266505295 7.8295620735
H 4.4851903803 -4.4045070969 7.2503931242
H -3.1787880631 2.5822743967 7.2047665085
H 3.4673857272 2.6044251436 6.0043064988
H 3.4798509894 -0.845495686 9.1360995935
H 2.188132457 -4.5194681409 2.6904922511

3aa
C -0.0454796592 -0.0040968581 0.0045472933
C -0.038881901 -0.0159571776 1.4305465629
C 1.2406541467 0.0067894291 2.054241104
C 2.4350313688 0.2523779157 1.3720394458
C 2.3802962684 0.3928753761 -0.0107068695
C 1.1600889387 0.2241284725 -0.6730717236
S 1.1989351796 -0.4460675778 3.7528231403
C -0.5524482142 -0.6105349007 3.6809216183
C -1.1160766743 -0.2140159621 2.4314841944
C -2.5310835162 0.0022654326 2.4131507639
C -3.2688616476 -0.4357924702 3.5251866168
C -2.6868463868 -0.9774661205 4.6751122764
C -1.3023756512 -1.0222091528 4.7835851041
P -3.5352042406 0.8744711909 1.0523515906
N -4.9298837948 1.2354219656 2.021425222
P -6.3873026275 1.2877530162 1.4550410294
N -6.8209798635 -0.0225383609 0.4659180269
C -6.1915851536 -1.3293140769 0.6506581077
P -1.5966732281 -0.2744826196 -0.9887807765

N -0.919374378 -0.364386049 -2.5689830607
P -1.7649904939 0.1816978748 -3.780498782
N -2.1850860596 -1.0606626021 -4.84700423
C -1.2454453755 -2.1644267936 -5.0225985053
N -2.0373709886 -1.8309029125 -0.5126618691
P -1.3841741142 -3.2507730888 -0.5145064517
N 0.2834334318 -3.5440399548 -0.5653191957
C 1.02533056 -3.3290487026 -1.8110417731
N -1.8405945514 -4.044381633 0.9053881576
C -2.8412861781 -3.4838431317 1.8075187178
N -1.887605882 -4.122938864 -1.8986675355
C -1.5740343672 -5.5440079111 -2.0195584964
C -1.3520155776 -5.3574145664 1.2975149931
N -2.7070236071 2.3401008965 0.8763364506
P -2.2952071913 3.4902696763 1.8580496386
N -0.6560470221 3.8424792403 1.6420094501
C -0.0015316835 4.940019923 2.3410886041
N -2.4320458578 3.4021016654 3.5372018779
C -3.7559547239 3.3816264988 4.1639320052
N -3.2494792436 4.8837745854 1.5295762117
C -3.135674484 6.0941066911 2.3416547668
C -1.4208627958 2.7100560342 4.3406331915
N -7.4560228298 1.4598907467 2.770373383
C -7.0675361649 0.9073149835 4.0661777598
N -6.8731412814 2.606522448 0.4790927855
C -6.201494981 2.6830414118 -0.8263583777
C -8.8868284915 1.340381802 2.4994713924
C -6.8750991428 3.9172968786 1.1496294424
C -7.9769787601 -0.0603712818 -0.4210483802
N -3.2820976303 0.9203413482 -3.5358612264
C -4.3782878147 0.1113980647 -2.9812745988
N -0.8362122716 1.3227924289 -4.6234304091
C -1.2560786593 1.8459964904 -5.9158546445
C -3.2762731885 2.2802173769 -2.9803928621
C 0.1121532183 2.1868777081 -3.9259414272
C -3.1946311765 -0.9737946609 -5.8937536768
C -3.19873254 -3.7910479876 -2.4513670803
C 1.0941229577 -3.2612267432 0.624466485
C -3.4301627526 5.2025254965 0.1133806539
C 0.0637348836 3.3820673502 0.4578012789
H -0.8117315274 -1.3447848747 5.6982936521
H -4.3418071447 -0.2895414955 3.5007316485
H 1.1351128904 0.211876722 -1.7577952843
H -3.4827773585 4.282326201 -0.4677155221
H 0.4326088237 -3.6595913503 -2.664484638
H -4.4998917328 3.8382109959 3.511017105
H -3.1708107743 -2.5182007773 1.4299926995
H -4.3646644923 5.7633143079 -0.0222243061
H 1.2827747595 -2.2712940663 -1.9548412686
H -4.076412205 2.3523979011 4.3620742675
H -3.7073392036 -4.1596062991 1.8922325122

H	-3.7145517159	3.939562926	5.1108031785
H	-2.4157363427	-3.3340300163	2.8098474108
H	-2.9685542025	5.8424416966	3.3897720518
H	0.4752717916	-3.2560321319	1.523292825
H	-2.3167206492	6.7518276247	2.0045502978
H	1.8634216191	-4.0389041142	0.7322760444
H	-4.073075795	6.6626344971	2.2677672633
H	1.5895279651	-2.2878351823	0.5466960766
H	-1.2246518707	3.2904267951	5.2539054697
H	-0.9812203204	-5.325472069	2.3335576279
H	-1.7640423862	1.7106732844	4.6285030381
H	-2.1491661778	-6.1163026691	1.248485466
H	-0.488869762	2.6034465574	3.7848834655
H	-0.5276520312	-5.6738330718	0.6568599145
H	-0.5439445541	5.1982368506	3.2527315128
H	-0.5557118002	-5.7412158948	-1.6777436651
H	0.0764140998	5.8411905806	1.7104727945
H	-1.642333029	-5.8388226941	-3.0751782996
H	1.0168071343	4.6386507233	2.6248302257
H	-2.2688992369	-6.1802041419	-1.4445894448
H	1.02296619	2.9348022447	0.7480271939
H	-4.0189591947	-4.3054579295	-1.9194911891
H	0.2596595726	4.2198093051	-0.2323201126
H	-3.2318614702	-4.0970813009	-3.5050423399
H	-5.8560355893	4.2707150678	1.3680423504
H	1.0555934827	2.2334605277	-4.487878189
H	-7.4341967009	3.8580143107	2.0849448915
H	0.3146276816	1.7913787591	-2.9317249956
H	-7.3683655238	4.6424874618	0.4904883683
H	-0.2744005411	3.214738032	-3.8236570305
H	-5.1523291091	2.9884044469	-0.7304760704
H	-0.3690334653	2.0918868351	-6.5151159196
H	-6.7279932188	3.4145849654	-1.4519174117
H	-1.8646524774	2.7612731813	-5.8180376544
H	-6.2229849777	1.7165388933	-1.33002617
H	-1.8367476763	1.1078098153	-6.468758581
H	-8.4075488132	0.934904936	-0.5368160695
H	-3.8239229672	-0.0943379203	-5.7478733972
H	-7.675488879	-0.4262201817	-1.4148012204
H	-2.7392596821	-0.9242973523	-6.8967891792
H	-8.7557616315	-0.7375830447	-0.0355278592
H	-3.8346143414	-1.8682385871	-5.8643341981
H	-5.7324098716	-1.6714113824	-0.2869074287
H	-0.6169925716	-2.0228165607	-5.9172651268
H	-9.213315574	0.2899659356	2.4093314644
H	-4.254691568	2.739022011	-3.1744049014
H	-6.9364554768	-2.0741859075	0.9733437997
H	-1.8022371809	-3.1035815034	-5.1374385626
H	-9.4480876668	1.8010086744	3.3221892283
H	-3.0884092707	2.2826785202	-1.8972510788
H	-5.4044986131	-1.2787563348	1.4020995149

H -0.6104025244 -2.2453264793 -4.1407093763
H -7.2606018512 -0.1774655276 4.1378574242
H -5.3343341592 0.5395875505 -3.3144743103
H -7.6462197626 1.4080739051 4.8532678925
H -4.3573126728 0.0905887294 -1.8827541734
H -6.0067933454 1.0886810093 4.2366077505
H -4.3161881325 -0.9129172396 -3.3533941511
H -9.142682445 1.8664233787 1.576756522
H -2.5183084899 2.8912087383 -3.4751135095
H -3.3141735137 -1.3008924314 5.5030615107
H 3.2911938339 0.5694776964 -0.5789311962
H -0.5267463712 2.627027094 -0.0558036447
H -3.3585386623 -2.7163999054 -2.3883860207
H -2.6017695166 5.8196128364 -0.2777927825
H 1.950568521 -3.9194199345 -1.7739819186
H 3.3796276222 0.2892146306 1.908576712

3aaH^{+}
C -0.0751373817 0.0765211076 -0.0722326053
C -0.0316538934 0.0440668985 1.3569691991
C 1.2616282129 0.0816082506 1.9549813997
C 2.4351318192 0.3675168018 1.2527211743
C 2.3441331052 0.5301542571 -0.1234755128
C 1.1138128318 0.3403924417 -0.7641256031
S 1.2796951172 -0.4277476169 3.6371924174
C -0.4631909555 -0.6188007809 3.6113022783
C -1.0633927842 -0.1934286587 2.3905487615
C -2.4788826345 -0.0013711233 2.4402178224
C -3.1971046791 -0.4730710417 3.5488247713
C -2.5724691736 -1.0627129792 4.652747701
C -1.1858400052 -1.0861180083 4.7122943502
P -3.4350938006 1.0840859662 1.3180652587
N -4.9322493885 1.1648188216 1.9612871097
P -6.3088437115 1.2270121345 1.1742258363
N -6.6367462727 2.5800714223 0.2194834246
C -5.8227122326 2.7513637283 -0.994680357
P -1.6104259177 -0.2643530071 -1.0956714858
N -0.9183318661 -0.4623893836 -2.6316555301
P -1.6572444535 -0.012350879 -3.952855739
N -0.6943639266 1.1109827638 -4.7616319795
C -0.9263358414 1.4559957554 -6.1606252669
N -2.1073049023 -1.7469099533 -0.4610230881
P -1.4268878907 -3.1731689936 -0.4482088683
N -1.9172418552 -4.0561613193 -1.8143409154
C -1.4990878722 -5.4389597457 -2.0391405278
N 0.2387242097 -3.4195967264 -0.4762323291
C 1.0055439258 -3.2668232018 -1.7153280468
N -1.8525765685 -3.9291791506 1.0071673821
C -3.0707554667 -3.5284404963 1.7046169749
C 1.0515298789 -3.1764836874 0.7204473236
N -2.6652813036 2.4855621431 1.0842992392

P	-2.219745638	3.5998722337	2.1304865764
N	-3.1548799754	4.9873270081	1.8367160285
C	-3.069036216	6.151215876	2.7280761553
N	-0.579477704	3.8652887859	1.945550842
C	0.0901176879	4.9274448808	2.6988411275
N	-2.425797285	3.3644287253	3.7720890362
C	-3.7747779516	3.3335652683	4.3515015403
C	0.1410836603	3.4536879621	0.7369265687
N	-6.4425636672	-0.0322454185	0.0624785873
C	-5.8775028578	-1.3500100631	0.3814025185
N	-7.5189092157	1.2751024118	2.3341728467
C	-7.2837717344	0.7979970214	3.698999885
C	-7.4935119789	-0.0988300661	-0.9573013113
C	-8.911584111	1.1594280997	1.8919399337
C	-6.832595762	3.8565614717	0.9292071187
N	-1.9343833494	-1.3198448754	-4.9736120161
C	-0.9146737988	-2.3649508702	-5.0509362073
N	-3.2159150076	0.6792700164	-3.8716644337
C	-4.3265551185	-0.1257091573	-3.3570108337
C	-2.8882800062	-1.3476619803	-6.0794945137
C	-3.3159342412	2.0896598824	-3.4905849253
C	0.1081505219	2.0982265126	-4.0422529601
C	-1.4614719733	-5.3112864172	1.2783778301
C	-3.1912460924	-3.7380517855	-2.4513256362
C	-1.4343333482	2.6464218711	4.5831546226
C	-3.2928529666	5.390754294	0.4316523434
H	-0.6667383553	-1.4399428153	5.5989431982
H	-4.2713260675	-0.3311003226	3.5602179875
H	1.0769229778	0.3255181595	-1.8477123868
H	-3.3251585908	4.5109517727	-0.2103230007
H	0.4092204085	-3.5764569094	-2.5721019086
H	-4.4867854283	3.8383270883	3.6978229508
H	-3.2772445211	-2.4826896361	1.4923883439
H	-4.2250481534	5.9544108289	0.3066694552
H	1.3200249946	-2.226596797	-1.8685871354
H	-4.1127726906	2.2992402221	4.4914332292
H	-3.9376687149	-4.1416058048	1.4042623147
H	-3.7617748339	3.8402046854	5.3248319696
H	-2.9245850424	-3.6441353306	2.785926945
H	-2.9378105638	5.8383972578	3.76440129
H	0.4233964724	-3.1206548433	1.610116843
H	-2.2399942095	6.8216105213	2.4549568513
H	1.770112587	-3.9977993422	0.846725211
H	-4.0037745199	6.7198872178	2.6541015954
H	1.6092900538	-2.2386453558	0.6279022923
H	-1.27876368	3.1872141338	5.5254890159
H	-1.3417736033	-5.4441362357	2.3612606861
H	-1.7822671446	1.6337556791	4.8115035371
H	-2.2163835112	-6.0335070406	0.92891757
H	-0.4829946826	2.5715627399	4.0567729678
H	-0.5072731737	-5.5431185623	0.8024974041

H	-0.4528661177	5.1561390485	3.6180209621
H	-0.5285676796	-5.6298210411	-1.5789744814
H	0.1828849858	5.8482580897	2.1042482883
H	-1.406028385	-5.618655063	-3.1186943652
H	1.0984326579	4.5937262537	2.9729843217
H	-2.2274475081	-6.1606507761	-1.6373643273
H	1.0999862327	3.0034292977	1.0164740745
H	-3.9987817581	-4.4027310681	-2.1011297236
H	0.3314551208	4.3188660818	0.084286282
H	-3.1002180499	-3.8552372865	-3.5390030051
H	-5.8797083286	4.2738181984	1.2871009401
H	1.1348659951	2.1025415706	-4.4321889207
H	-7.4990486136	3.7165991916	1.7811530642
H	0.1393642019	1.8591342136	-2.9801237005
H	-7.294291266	4.5693170046	0.2372903315
H	-0.3052074016	3.1125061146	-4.1613652578
H	-4.8174565017	3.1233543411	-0.7599074398
H	0.0309219215	1.7073714471	-6.633895448
H	-6.320371481	3.4722643596	-1.6527011377
H	-1.5991866733	2.3222971387	-6.2694036598
H	-5.7185217106	1.8088211351	-1.5305909111
H	-1.35550743	0.6151529731	-6.7051116339
H	-7.8993690789	0.8919266016	-1.1635899651
H	-3.5471531658	-0.4791230639	-6.0441518968
H	-7.0695536011	-0.5027632082	-1.885620309
H	-2.3737464079	-1.3612782836	-7.0524004337
H	-8.3157692588	-0.7568285505	-0.6411337854
H	-3.5017262379	-2.2577055882	-6.0150075339
H	-5.2960646652	-1.7253301245	-0.4686841113
H	-0.2879202557	-2.2462119702	-5.9480514686
H	-9.2370427054	0.1101329559	1.8239338542
H	-4.3052999038	2.4637105127	-3.783378539
H	-6.6751214326	-2.0697998211	0.6156867946
H	-1.3981651948	-3.349376662	-5.0960204022
H	-9.559706991	1.6740102772	2.6104951727
H	-3.186809111	2.2390252621	-2.4082020867
H	-5.2085156035	-1.2887798498	1.2384954134
H	-0.284956153	-2.3274294429	-4.1633885126
H	-7.4776236806	-0.281323711	3.7981272577
H	-5.2685154445	0.2600383112	-3.7704294904
H	-7.9578985738	1.3321353616	4.378921703
H	-4.385316693	-0.1005928021	-2.2589724056
H	-6.2544858048	1.0052094276	3.9904077986
H	-4.2171864462	-1.1646126576	-3.6716857813
H	-9.042982333	1.634849202	0.9174740555
H	-2.5678001089	2.6825589033	-4.0194211628
H	-3.1669171042	-1.4326890747	5.4833770845
H	3.2373691328	0.7329140819	-0.7093067825
H	-0.438482192	2.7133839243	0.1913270433
H	-3.4592478364	-2.7069415499	-2.2301990848
H	-2.4574107355	6.0329271816	0.1097231552

H 1.8960617841 -3.9055665141 -1.6583073095
H 3.3915443259 0.4104560102 1.7664365828
H -3.4599048397 0.5005431805 0.0472165207

3bb

C -0.0234533688 -0.1114974422 0.0081272459
N -0.0151287352 -0.1000640573 1.4746565539
C 1.3635886483 -0.066926126 2.0160130528
C 2.2499840455 -0.1931152351 0.7674312361
C 1.3796131375 0.3993402404 -0.3525687807
P -1.3278795821 -0.5387193784 2.4280871936
N -1.6081822269 -2.1555012162 2.0117162385
C -0.45912535 -3.093159938 1.9304172767
C -1.0405127749 -4.4627313704 2.3327716772
C -2.5542700008 -4.3094779586 2.1147843821
C -2.805873783 -2.8551532762 2.5230921243
N -0.9831904608 -0.0979494126 3.8781480425
P -1.2059712671 -0.4477849357 5.4953767862
C -0.7309099851 -2.2377824143 5.6349012178
C 0.6157944722 -2.7022301567 5.5199822919
C 0.7928196169 -4.1151637302 5.6025688848
C -0.2183213126 -5.0186808434 5.9387540137
C -1.4972359757 -4.5184707522 6.1561441698
C -1.7366384872 -3.1545137294 5.9796999269
S 2.4183749026 -4.6503803283 5.2204191564
C 2.9439838983 -2.9823196138 5.0521978939
C 1.9205368737 -2.0135065662 5.3011366861
C 4.27595884 -2.6788227978 4.7678882965
C 4.6518626075 -1.3432216033 4.7805793989
C 3.7063061287 -0.3716067878 5.1137590733
C 2.3602793684 -0.6475060171 5.3987996525
P 1.3217673492 0.8745966037 5.8948380152
N 2.5388143252 2.098072519 5.7808326803
P 2.4229771001 3.5300805047 5.1615590475
N 0.9070516051 4.0342473092 4.5876159425
C 0.4707074139 5.4504629426 4.5005358749
C -0.2912646496 5.5471038158 3.169472035
C -0.8390992993 4.1253075623 2.9819616895
C 0.3124520107 3.2461736717 3.4766706467
N -2.9089331192 -0.5217695708 5.7509053886
P -3.6101150088 0.3805228255 6.8371570817
N -3.4705762858 2.0464236527 6.5858864722
C -4.3001733074 3.0564868201 7.2778401986
C -3.288383287 4.0783907136 7.8111248477
C -2.2089589625 4.0718282593 6.7191891034
C -2.1060008702 2.5906394719 6.3308941559
N -3.1610264473 0.0060555257 8.4144117984
C -2.1199407889 -0.9930932421 8.7638510284
C -2.0133244043 -0.8939924259 10.2945595536
C -3.3936873539 -0.3792628371 10.7309215981
C -3.7541297136 0.6116386021 9.6154940894

N	-5.3010289017	0.2055136706	6.8501892096
C	-6.021313599	0.5803017948	5.6152896232
C	-6.1087580081	-0.7144684403	4.7741127345
C	-6.0368535449	-1.8611392273	5.8196144537
C	-5.8082778791	-1.1431700303	7.1765907722
N	-2.7136778528	0.2119971289	1.8557188796
C	-3.4244077585	1.2279560371	2.6640140555
C	-4.7784542949	1.412741362	1.9384269269
C	-4.9083357269	0.1747801185	1.0322243215
C	-3.4525893773	-0.0897434137	0.6284101126
N	1.1171129858	0.5733059292	7.5325333992
P	1.7496415258	0.4687541404	8.9395531501
N	1.1161877185	1.668390629	9.9325553425
C	1.6115529047	2.1089246473	11.2442083865
C	0.8600396478	3.4274547352	11.4733079412
C	-0.5096157435	3.1404019994	10.8367079041
C	-0.1502058862	2.3421396828	9.574591844
N	3.4193937534	0.6467461665	9.2004275192
C	4.055816034	1.9253909304	8.795553364
C	5.3590453408	1.5178674398	8.0912392582
C	5.6809141681	0.1441946003	8.6991783903
C	4.2937925629	-0.4929730179	8.8296240896
N	1.3921792564	-1.0302029528	9.6201332713
C	1.3378259004	-2.264598932	8.8020432099
C	1.8450278203	-3.3585717331	9.7491471148
C	1.3932398158	-2.8579301508	11.1297854282
C	1.6183618497	-1.3389197268	11.0407310247
N	3.550660732	3.7284824147	3.9218582568
C	4.2375349293	2.603230635	3.2553378122
C	4.4648695504	3.1097862032	1.824017168
C	4.6246558202	4.6259888095	2.0142810255
C	3.5679087914	4.9382028447	3.0833606147
N	2.8102409467	4.8054506917	6.2119337665
C	4.2242693511	5.0208300808	6.5808887836
C	4.1249161871	5.9893006211	7.7633427001
C	2.8936169528	5.4688057132	8.5241373875
C	1.9473033485	4.9803416106	7.4078527203
H	-4.8354726026	0.7389506512	9.494143188
H	-1.7488266959	-1.8550765487	10.7465108632
H	-1.2307597486	-0.1787631725	10.5691517979
H	-3.3184227768	1.6025224047	9.823684986
H	-4.1205603517	-1.201879739	10.7427787705
H	-3.3927787051	0.0865639445	11.722757328
H	-2.4292245876	-1.9966477744	8.4473253474
H	-1.1706591393	-0.7597217009	8.2767911808
H	-3.7325940033	5.0652992297	7.9841111478
H	-1.354194475	2.0819676049	6.9430417295
H	-1.2442258609	4.4658861228	7.0521015828
H	-2.5389051924	4.6740345322	5.862314572
H	-1.8204747732	2.4415747655	5.2880258708
H	-2.3124939104	-5.1895677661	6.4196309417

H	-2.7448040736	-2.7655263413	6.0876156201
H	-6.7584587409	-1.0248035182	7.7162538857
H	-5.1118481302	-1.6671127087	7.8364663816
H	-6.9490582607	-2.4682955074	5.8420423874
H	-5.1945940782	-2.5213329368	5.5945729264
H	-5.509911244	1.399541609	5.1056829594
H	-7.0241412292	0.9292271205	5.9013022829
H	-2.8953027179	-2.7635297426	3.6138512245
H	-0.8392143438	-4.6489880891	3.3907063342
H	-2.8114644674	-4.4511143317	1.056602758
H	-3.1443153885	-5.0163655614	2.7083808887
H	-0.6005549173	-5.2837623486	1.7563990948
H	-3.713591944	-2.4379305796	2.0758384071
H	-3.5393632706	0.865921891	3.6881652407
H	-3.2582565355	-1.1145551367	0.3001586997
H	-3.1617212605	0.5854289062	-0.1950666629
H	-5.2928705717	-0.6778197475	1.6049559397
H	-5.5688373967	0.3369047037	0.1729043357
H	-4.760264556	2.3214375859	1.3233819269
H	-2.8652115009	2.1694369433	2.7063636241
H	-0.8175855299	0.5335212742	-0.3841299159
H	-0.185874532	-1.124921893	-0.3968312969
H	1.692213108	0.0975866921	-1.3582118215
H	1.3977816545	1.4964036736	-0.304144085
H	3.2108468365	0.3180869471	0.8899207838
H	2.4577669122	-1.2504706785	0.5589300586
H	1.5377856881	0.8749895945	2.5464472656
H	1.5445742878	-0.8677858273	2.7360546418
H	-4.9200395117	2.607491104	8.0569220001
H	-4.9824705871	3.5335396236	6.5562944245
H	-7.0218573823	-0.7449411029	4.1684714912
H	-5.6098321064	1.5096585205	2.6443349407
H	0.3566020484	-2.8074739071	2.6040579927
H	-0.0634756395	-3.0989862958	0.9059447062
H	-5.2474141483	-0.7719578724	4.1042751472
H	-2.8640341857	3.7235038201	8.7593132764
H	3.8089692926	5.8298404331	3.675834178
H	5.327000859	2.6356088653	1.3431756692
H	3.5782747725	2.9008317435	1.2103184602
H	2.5875634229	5.1100904155	2.6139703204
H	5.6275557604	4.853843299	2.3981492719
H	4.4738152646	5.2031570211	1.0956732857
H	5.1934328068	2.3859542703	3.75532272
H	3.637275276	1.6924989083	3.2874498214
H	-1.0707251816	6.3168902403	3.1949643936
H	1.0468736326	3.1060569964	2.6648953002
H	-1.1201429887	3.8933066975	1.9494540338
H	-1.7229980592	3.9773044084	3.6133902945
H	-0.0067793336	2.2568939289	3.8149009466
H	5.682364809	-1.0566493436	4.5788942675
H	4.0226648001	0.6598792927	5.2054671705

H 4.7812964523 5.4326312808 5.7338645165
H 4.7184344748 4.0861908238 6.8861043604
H 3.946915115 7.0080567413 7.3959433097
H 5.0351255755 6.0001353559 8.3727052924
H 1.454788279 4.0397709612 7.6808058946
H 1.1655247983 5.7190870904 7.1933143961
H 3.4071707785 2.4878630191 8.1196223527
H 6.3524136839 -0.4576336697 8.0774498091
H 6.1597117958 2.2530358494 8.2311283913
H 5.1656240623 1.4196214628 7.0179600708
H 6.1408025531 0.2594854938 9.6896842598
H 4.2586130769 2.5364696921 9.6878040792
H 0.0012312787 3.0039667121 8.7136993718
H 2.6987132346 2.2256177694 11.2363978805
H 1.3630585863 1.3896882717 12.0406088124
H 1.3622423944 4.2411940233 10.9344739274
H 0.8025206644 3.7020752159 12.5321719419
H -1.1143490987 2.5227559633 11.5144574384
H -0.9264436152 1.628493213 9.2879549298
H 0.9193165998 -0.7845221243 11.6782175393
H 2.639816795 -1.0705137337 11.3556446762
H 1.9399626707 -3.3115291475 11.9636812595
H 0.325714458 -3.0686356048 11.2722884945
H 1.446753 -4.3450847872 9.4904934718
H 2.9404232822 -3.4158710892 9.7079021594
H 0.3102835351 -2.4664597592 8.4772702616
H 1.9451772519 -2.17635381 7.9010139046
H 1.3172259188 6.1403994222 4.5604096834
H -0.2020420299 5.681174072 5.3372938692
H 2.426274739 6.2295267139 9.1586232979
H -1.0828558399 4.0449894859 10.6068193923
H 3.9990512389 -0.9379550561 7.8686006692
H 4.2509108871 -1.2727053265 9.5974456206
H 3.1793888166 4.6306868822 9.1677208246
H 0.3968559896 5.7954064308 2.3509446837
H 4.9921830079 -3.4722365908 4.569787888
H -0.0028207539 -6.0814448215 6.0149670914

3bbH^+
C 0.0737630323 0.0209613113 -0.0392205757
N 0.0721968486 0.2240065855 1.4343732598
C 1.4470961695 0.4488619968 1.9367136311
C 2.3345314506 0.0301303199 0.7569165996
C 1.4894837529 0.429338254 -0.4604361777
P -1.2693030675 0.6619525398 2.3309306266
N -1.2994462293 2.3373130838 2.3648090264
C -2.3833281433 3.0601173923 3.0547756241
C -1.9311114241 4.5161358289 2.9231441741
C -1.3614651657 4.5728076788 1.4907411654
C -0.8463331744 3.1398507142 1.1996978516
N -2.6054335361 -0.0560148432 1.8712240482

P -3.2913207295 -0.4808523466 0.4591150357
N -4.75148538 -1.0930542919 0.647132885
P -5.5824046528 -2.0451554178 1.5820634734
N -4.9200975217 -3.5754216409 1.7345514024
C -5.2297965707 -4.5248391564 2.8340402343
C -5.4385914461 -5.8714639339 2.1283229246
C -4.5172715678 -5.7650244209 0.9050181156
C -4.6897593043 -4.309406438 0.4608768139
C -3.5273169375 1.0134031185 -0.5827334628
C -2.8714193851 1.3961539906 -1.7876751513
C -2.9189971949 2.7891737353 -2.1027357988
C -3.702221624 3.7153369093 -1.4118148351
C -4.4583218122 3.272580985 -0.3326970522
C -4.3357823398 1.9473298239 0.0864047239
C -2.0992652131 0.6542635737 -2.8123206804
C -1.4583201865 1.5597459305 -3.71030288
S -1.8824022808 3.2459309876 -3.4427349341
C -0.6455309665 1.1494061451 -4.7670550281
C -0.5050654765 -0.2167431111 -4.9941580274
C -1.2102015371 -1.1226313924 -4.1985476334
C -2.0022075633 -0.7307828784 -3.1143702348
P -3.04312565 -2.1001174977 -2.3043837879
N -1.8063823516 -2.9009403457 -1.4959510543
P -0.9636050717 -4.1984881593 -1.3564504996
N -1.1230609322 -4.8217189901 0.1993716125
C -1.1410616659 -3.8701010075 1.3356049404
C -0.9280957594 -4.7619194634 2.5684150721
C -0.0620371787 -5.9104623841 2.0284391813
C -0.6599638048 -6.1567644039 0.6369713084
N -3.4136446865 -3.067823131 -3.6390950178
P -4.7167537899 -2.987370408 -4.5181351951
N -4.3202830976 -2.9625074962 -6.1409797585
C -3.1465478346 -3.6820996246 -6.677489207
C -3.3317259764 -3.5961662457 -8.2027030696
C -4.1458854833 -2.3058947715 -8.3921754503
C -5.1235543897 -2.3491178648 -7.2103452791
N -5.6922534825 -4.3345892335 -4.316001984
C -6.4399727131 -5.0298998772 -5.3776114934
C -7.0511860511 -6.2286197564 -4.6389752725
C -7.3496139764 -5.6507963785 -3.2452359998
C -6.1309850263 -4.7549525702 -2.9730282514
N -5.7340536014 -1.6798121452 -4.2435593016
C -7.2005544462 -1.7187690319 -4.4116222293
C -7.6320726878 -0.2837593069 -4.0799339569
C -6.4579869639 0.5565333018 -4.6060813192
C -5.2351258132 -0.2833175085 -4.2120715299
N -0.988196521 0.2611715714 3.9207908256
C -0.991299877 -1.1634691529 4.3268134913
C 0.1070159014 -1.2449866532 5.3937555344
C 0.0228324476 0.135326372 6.06291231
C -0.2169490707 1.0876200855 4.8804245876

N	-1.2805124507	-5.5515638914	-2.3065028502
C	-2.5832964252	-6.2512299299	-2.1792144434
C	-2.9456175483	-6.680942235	-3.6126236291
C	-1.5879786453	-6.7205588404	-4.3321453322
C	-0.8606878208	-5.5165207044	-3.7273686759
N	0.6660819287	-3.866406292	-1.6126590484
C	1.741962454	-4.8375162174	-1.3219510395
C	2.9799756605	-4.2693676762	-2.0478154475
C	2.6489322422	-2.7781589953	-2.2237450269
C	1.1470673682	-2.8089141861	-2.515477758
N	-5.8425756425	-1.586519582	3.1704575893
C	-6.6236498216	-0.3383935568	3.3879865202
C	-6.1315143588	0.1743087402	4.7487437351
C	-4.6576639431	-0.2577207108	4.7650248539
C	-4.7108959288	-1.6546203329	4.136844382
N	-7.0912874974	-2.0866129154	0.8841683042
C	-7.4058301508	-1.5398726989	-0.4587427602
C	-8.6631984537	-2.3203850265	-0.8712194576
C	-9.3461948813	-2.6374614458	0.4688848646
C	-8.1581787712	-2.9720922875	1.3814851345
H	-3.7175268957	4.7577474421	-1.7182571979
H	-0.1679727693	1.8803874823	-5.4137847701
H	0.1159690207	-0.5717352272	-5.8136088552
H	-1.1730308864	-2.1808017645	-4.4390304994
H	-4.8593498244	1.6281475272	0.9831619372
H	-5.1001376911	3.9616057061	0.2096587316
H	1.6314606839	-0.1469955043	2.8362202982
H	1.0880867619	-1.3790791572	4.9205279064
H	0.7330888982	1.4269086836	4.4501367005
H	3.3174534938	0.51023984	0.7863876214
H	2.4849624268	-1.0567441201	0.7707381327
H	1.6139201166	1.5065321112	2.1916626438
H	-3.3500891636	2.8992399355	2.5551949549
H	1.5375866159	1.5146253498	-0.6142557001
H	1.8004664119	-0.0541252039	-1.389876279
H	-0.6808713227	0.6323345134	-0.5359048821
H	-0.1308447674	-1.0286238952	-0.2782809458
H	-4.0527829688	0.397834054	4.1298896343
H	-2.4772555175	2.720593499	4.090250076
H	-2.7477996766	5.2271095249	3.0839545589
H	-1.1473177763	4.7239795743	3.6609677968
H	-0.5698658582	5.3205518279	1.3850487678
H	-2.1531206657	4.8258680365	0.7791857887
H	0.2446572717	3.0920003138	1.1257238003
H	-1.2663380642	2.7716631467	0.2559818525
H	-0.7805287622	1.9781320734	5.1731996335
H	0.9196315943	0.4022056364	6.6301737598
H	-0.8319621123	0.1658107158	6.7501073291
H	-0.0472580602	-2.0749794744	6.0905959786
H	-0.8085672312	-1.8162736313	3.4681166008
H	-1.9658838548	-1.4309719254	4.7515917102

H -2.405270279 -1.3443637158 -0.1820005485
H -4.9238111613 -2.4244107517 4.8903858913
H -6.1006137166 -4.2010179791 3.4107626277
H -4.3741720115 -4.5735765067 3.5214496976
H -5.2052124842 -6.7216615055 2.7774887315
H -6.4833052169 -5.9714522408 1.8069616547
H -8.3641019491 -2.7832966964 2.4413394637
H -3.820328918 -3.9135475687 -0.0660916188
H -0.3323345306 -3.1291150729 1.2416916156
H -2.0866641909 -3.3230414737 1.3754787791
H -1.8918170591 -5.1489569719 2.9231362831
H -4.7682446913 -6.4724405415 0.108918954
H -0.4624054648 -4.2210801808 3.3986244838
H 0.9814324926 -5.582147327 1.9368862925
H -0.0802800584 -6.8053332202 2.659121582
H -1.5132398983 -6.847553008 0.7023869339
H 0.050534 -6.5933255079 -0.0677603271
H 3.225361283 -2.2991248791 -3.0218293298
H 0.9725221103 -3.0621177874 -3.5737359547
H 3.9062072901 -4.4488442078 -1.4929426451
H -5.3311278512 -5.3099727333 -2.4682650356
H 1.9040707811 -4.9019589073 -0.2377734655
H 3.0863244114 -4.7422538985 -3.0317911405
H 1.4910424273 -5.844303333 -1.6815224425
H -7.4857767475 -6.4180540537 -2.4760925105
H -2.4709219381 -7.1151437936 -1.5112115325
H -6.3114490209 -7.0351122623 -4.5619197582
H -7.9371815951 -6.6252443541 -5.1442628904
H -3.5849319326 -5.919341153 -4.0688304891
H -5.7721735592 -5.3246769534 -6.1934762934
H -1.0489115605 -7.6465389115 -4.0953495554
H -1.6752950993 -6.6471234124 -5.4213998255
H -3.1120946357 -4.718939952 -6.3207509851
H -7.2338692196 -4.3964650825 -5.8041749832
H 0.22951963 -5.5872253048 -3.8041305794
H -1.1951196165 -4.5911197393 -4.2171623756
H -3.4749977377 -7.6396379115 -3.6372554337
H -2.2231053169 -3.1896408726 -6.3489388006
H -2.3766645136 -3.5890346373 -8.7373209035
H -3.9101090194 -4.4569383229 -8.561935535
H -4.6569990911 -2.2531406169 -9.3585598878
H -3.4934620271 -1.4285902363 -8.300496827
H -6.0070892357 -2.9590521502 -7.4606039906
H -5.4755081539 -1.3566840578 -6.912544145
H -7.6535627372 -2.4598002536 -3.7455742182
H -7.4875614805 -1.9764712388 -5.4430547244
H -7.7211416114 -0.1627096556 -2.99341019
H -4.3856888273 -0.1463324152 -4.8908270207
H -8.5942547411 -0.0237049601 -4.5325646366
H -4.8975357648 -0.0301220195 -3.20087043
H -6.4198641196 1.5646276959 -4.1817125104

H -6.5233319941 0.6518843269 -5.6975118708
H 2.8376137289 -2.2339701439 -1.2902499638
H -6.5675137636 -1.6709766794 -1.1480350796
H -7.6097249599 -0.4628658376 -0.387750238
H -8.3794724868 -3.2546603775 -1.3723014082
H -9.8679323149 -1.7494352597 0.846689969
H -10.0704011311 -3.4557128201 0.4080107587
H -6.374073172 -3.8906795671 -2.3441132279
H -3.344188451 -5.592011022 -1.7533059093
H -5.5624174498 -4.2081386573 -0.1996489446
H -7.8734821074 -4.0313633338 1.2785838422
H -6.6822205512 -0.3171475079 5.5606019196
H -4.2208903255 -0.2673092693 5.7693613435
H -6.2712573626 1.2549403201 4.8539322638
H -8.2640550454 -5.044294928 -3.2809913159
H -6.4109858131 0.3982688433 2.6003047141
H -7.6971810498 -0.5520725187 3.3699137372
H 0.6260349622 -1.8722016587 -2.3161707333
H -3.4756425319 -5.9391544243 1.190507143
H -3.7814443922 -1.9172296615 3.6266978694
H -9.2981945362 -1.7519460335 -1.5578686962

4aa
C 0.0474734694 -0.070423816 0.0383248597
C -0.015833509 -0.0523672148 1.4540175545
C 1.2052590443 -0.1387105542 2.1601306846
C 2.4655609327 -0.0590038346 1.5834033568
C 2.5015054576 0.0504201673 0.1918084399
C 1.3149980803 0.0160358396 -0.5558594413
O 1.0077271299 -0.3512462305 3.5039414931
C -0.3564228295 -0.3722369568 3.6873955761
C -1.0634687679 -0.1034787593 2.4891163569
C -2.4666449078 0.1136476196 2.589390654
C -3.057892583 -0.1724958658 3.8325049815
C -2.328741578 -0.5579929097 4.9661946715
C -0.9365683272 -0.6268858501 4.9209545441
P -3.5800760609 0.761706757 1.1989130119
N -4.970230897 1.0755157175 2.1830785178
P -6.4422608428 1.0382120959 1.6547951352
N -6.8737688509 -0.3952644264 0.8558881723
C -6.1845065672 -1.6457793005 1.165819997
P -1.4965451464 -0.2365984294 -0.9895770317
N -0.7821911582 -0.2863741558 -2.5534330902
P -1.5598443129 0.3462102678 -3.7667773128
N -1.9619535157 -0.8331463447 -4.9095041468
C -1.0702404073 -1.9790456861 -5.0653948421
N -1.978457388 -1.797348856 -0.5866826426
P -1.4509285571 -3.2621748844 -0.5832328577
N 0.1885783743 -3.703555786 -0.5994132469
C 0.9677081709 -3.4879989315 -1.823325768
N -1.9974596813 -4.0116409357 0.8307302341

C	-2.9564330599	-3.3602917575	1.7165627056
N	-1.9928167349	-4.0803058595	-1.9831806504
C	-1.7704736928	-5.5135703366	-2.1461177148
C	-1.6953103131	-5.3907493675	1.1813761154
N	-2.9189885614	2.284733004	0.8629838241
P	-2.4509807224	3.5307045067	1.683405538
N	-0.8478523413	3.897372105	1.2799889709
C	-0.1934998283	5.1049052929	1.7669075692
N	-2.4347317702	3.6035740163	3.3695565635
C	-3.7094790785	3.5382627124	4.0902572337
N	-3.4709925063	4.8670153727	1.3165387134
C	-3.3438866799	6.1434441379	2.0176902065
C	-1.3177886365	3.0592371835	4.1456589917
N	-7.4756014685	1.3563672372	2.9700004273
C	-7.0257504949	1.0148494303	4.3176960476
N	-6.9812910899	2.2093806834	0.5315776226
C	-6.3499341179	2.1284470933	-0.7941332501
C	-8.9078625427	1.1516086327	2.7689862117
C	-7.0100388436	3.5941884879	1.0303775846
C	-8.0560006993	-0.5743343712	0.0229015139
N	-3.0694248222	1.108482556	-3.5519029768
C	-4.2070266613	0.2918984037	-3.1032687751
N	-0.5707977445	1.5136808116	-4.4991482411
C	-0.9466016476	2.1658902075	-5.7457287961
C	-3.0632836381	2.4262721769	-2.9013935677
C	0.4019159586	2.2703374365	-3.7157855298
C	-2.8837251618	-0.6499644653	-6.0225536267
C	-3.244067301	-3.6306290973	-2.5871383539
C	0.985778241	-3.4849696591	0.6133692054
C	-3.7838859183	5.0564591788	-0.0991004827
C	-0.235553645	3.3389579063	0.0771753312
H	-0.3253932421	-0.8551267138	5.7884945499
H	-4.1305033141	-0.0390778032	3.9150990248
H	1.3645888115	0.0034334007	-1.6405356748
H	-3.9074623784	4.0886541446	-0.582467613
H	0.3764443787	-3.7599288153	-2.6986941123
H	-4.5241193007	3.9040133952	3.4638988842
H	-3.1347405512	-2.3439096995	1.3710394811
H	-4.7206706444	5.6210475756	-0.1954684678
H	1.2838835284	-2.441380103	-1.9283438516
H	-3.9448041453	2.5062427177	4.3762317648
H	-3.9106657405	-3.9113917744	1.7336742481
H	-3.6442913194	4.160288476	4.9944229499
H	-2.5617111162	-3.3173849814	2.7420607342
H	-3.0715365824	5.9832485972	3.06174627
H	0.3542396793	-3.5431348502	1.5014369353
H	-2.5913352627	6.8049392844	1.5564341604
H	1.7576250493	-4.2641310773	0.6799086696
H	-4.3106503712	6.6648575706	1.9877462251
H	1.4776041222	-2.506046717	0.6036469449
H	-1.1092051572	3.7265137231	4.9944764747

H -1.3841265315 -5.4477477524 2.2354303847
H -1.5535604359 2.0631378686 4.534520307
H -2.5720124402 -6.0463044499 1.0540463856
H -0.4217438307 2.9833030014 3.5289647001
H -0.877466993 -5.7763278646 0.5705001201
H -0.6447686563 5.4403824908 2.7029169518
H -0.7874371291 -5.7935912857 -1.7620299362
H -0.2485991352 5.9279971156 1.0350683669
H -1.8031698077 -5.7639550835 -3.2151385721
H 0.868227716 4.8946241658 1.9573985678
H -2.5365983414 -6.120588251 -1.6337821879
H 0.781050885 2.989197129 0.2985060058
H -4.1250561256 -4.1146081003 -2.1292593958
H -0.1804547298 4.0938946765 -0.7253048918
H -3.2370899227 -3.8779963819 -3.6570511967
H -6.0007280535 3.9931408526 1.2076090724
H 1.3431158118 2.3551923333 -4.2767935692
H -7.5741160454 3.6426001678 1.9634206928
H 0.5978420868 1.7584986133 -2.7744905908
H -7.513102079 4.2202144738 0.2830839713
H 0.0433758582 3.2901051337 -3.495377145
H -5.3029410089 2.4585520023 -0.7760087651
H -0.0425041899 2.3972654975 -6.3252614949
H -6.9108429837 2.7635614253 -1.4909993503
H -1.4905576577 3.1103181712 -5.5733689223
H -6.3692017207 1.103906861 -1.167024384
H -1.5765893695 1.5183237193 -6.3549115336
H -8.5197930555 0.3873276685 -0.1998158033
H -3.5057384453 0.2327542065 -5.8642945047
H -7.7761512773 -1.0527861682 -0.9283191753
H -2.3518794154 -0.5463171154 -6.9830201261
H -8.8007463354 -1.218453238 0.5170381645
H -3.5425492985 -1.5274115089 -6.1018384404
H -5.73706317 -2.0746746998 0.2586008983
H -0.3807878314 -1.8438475833 -5.9149513215
H -9.1959977185 0.0881384154 2.8322520482
H -4.0068150783 2.9363952705 -3.138397166
H -6.8869727112 -2.3783152919 1.5943439526
H -1.6669441136 -2.8832571457 -5.2457308848
H -9.46116619 1.7003340217 3.5414224472
H -2.963624569 2.3441502317 -1.8102202615
H -5.3830668346 -1.4742064345 1.8834715392
H -0.4957177778 -2.1197425983 -4.1499558825
H -7.1722931429 -0.0535413489 4.5539889052
H -5.1378096306 0.7884848815 -3.4107210539
H -7.5995590136 1.6059941927 5.0433447725
H -4.2142136992 0.1646257523 -2.0118682746
H -5.9681903939 1.2563518075 4.4197672046
H -4.1724289437 -0.6933774488 -3.5720106072
H -9.2125979549 1.5388459024 1.7937707433
H -2.2464537009 3.0402016885 -3.2871105386

H -2.8510733088 -0.7565697106 5.8995325754
H 3.4598573306 0.1097689021 -0.3196057935
H -0.824900194 2.4936068582 -0.271599804
H -3.3365654884 -2.5520338109 -2.4732564437
H -2.9922025224 5.6179126289 -0.6267510618
H 1.8599795536 -4.1267808595 -1.7882733723
H 3.3618363779 -0.1058576979 2.194020169

4aaH^+
C 0.0953007878 -0.1112789204 0.0722654353
C 0.0114786842 -0.0867368095 1.4896611274
C 1.2219767684 -0.1741200968 2.2142682671
C 2.4919006552 -0.1027515363 1.6578660802
C 2.5475925268 -0.0035603314 0.2683563966
C 1.3716918683 -0.0376021712 -0.4987560985
O 1.0050966847 -0.3825535329 3.5560009477
C -0.3527381161 -0.3975039742 3.7240031906
C -1.0433951021 -0.1294782123 2.5145057328
C -2.4489509974 0.068899746 2.6236111947
C -3.0621662061 -0.2065744709 3.858432724
C -2.3391823053 -0.5933797578 4.9943727125
C -0.9483772646 -0.6546798452 4.9500005237
P -3.5138275439 0.8946400993 1.3829979098
N -4.9964931721 0.9244972747 2.0558285634
P -6.4116944752 0.9670435104 1.3410345876
N -6.7816483918 2.2557359899 0.318169321
C -6.074506167 2.3230719577 -0.9707347871
P -1.4247662087 -0.2692807427 -0.9908840077
N -0.7119615652 -0.4334478124 -2.5221598283
P -1.3611705747 0.2259689662 -3.8024636002
N -0.3070465189 1.3909823647 -4.4196544166
C -0.5001659707 1.9789205163 -5.7415329481
N -2.0315934926 -1.7501130111 -0.4453787049
P -1.4892549605 -3.2315801067 -0.504308198
N -2.0410643638 -3.9924023838 -1.9166604621
C -1.724508312 -5.3790239889 -2.2522953279
N 0.1463706324 -3.641412159 -0.5332590237
C 0.9407892108 -3.47724045 -1.7535728737
N -2.000288567 -4.0148510605 0.9076264675
C -3.0669902205 -3.4603132532 1.7322393003
C 0.9563809348 -3.5335236553 0.6849880759
N -2.9087362033 2.3227813375 0.9311775276
P -2.4081659096 3.5783945972 1.7666384654
N -3.3949537662 4.8987954039 1.3537003719
C -3.3262232604 6.1608649578 2.1009409361
N -0.7981257563 3.8509885116 1.4054065084
C -0.1231708908 5.0482853961 1.9108136814
N -2.482357556 3.5732143975 3.4356520059
C -3.7917519582 3.5307615661 4.0998701025
C -0.1603005815 3.2641977814 0.2231836669
N -6.6244155451 -0.3601509679 0.3263938372

C	-6.0147967995	-1.6513235197	0.6653639397
N	-7.5544278732	1.1139106431	2.5615793636
C	-7.2503500777	0.7102079502	3.9366112891
C	-7.754172015	-0.4907801497	-0.5988320289
C	-8.9718678203	0.995058162	2.2065665579
C	-6.9192819673	3.5841230343	0.9408393332
N	-1.6638579443	-0.9324846917	-4.9827584629
C	-0.7459044011	-2.0641942055	-5.1051965356
N	-2.8829530522	0.9909451325	-3.6959962983
C	-4.051148151	0.180474281	-3.341798801
C	-2.5310804012	-0.7522787454	-6.1440060509
C	-2.9366547104	2.3350867055	-3.1147791788
C	0.5525072124	2.1873644618	-3.5469130599
C	-1.8246351731	-5.4565715629	1.0640617457
C	-3.2477036812	-3.5055714116	-2.5768282965
C	-1.3910682441	3.0542565781	4.2691655274
C	-3.5872991534	5.1301593652	-0.0832054989
H	-0.3425094083	-0.8863945522	5.8200554959
H	-4.1358340136	-0.0831397131	3.9323942582
H	1.4415126294	-0.0647412161	-1.5816385577
H	-3.6123378432	4.1817109139	-0.6184626599
H	0.3193266029	-3.6307850381	-2.6346387624
H	-4.5727320099	3.9034369928	3.4355758289
H	-3.090614986	-2.3799770583	1.6115247322
H	-4.5402451552	5.6481839301	-0.2435228719
H	1.3840017519	-2.4757119603	-1.8146742282
H	-4.0485141297	2.5039871083	4.3889757559
H	-4.0530475049	-3.8780719907	1.4673302154
H	-3.7620289464	4.1582989244	4.9993842698
H	-2.8694765312	-3.691983757	2.7872576936
H	-3.1402625263	5.976461272	3.159281029
H	0.3223233019	-3.5962383834	1.5700896694
H	-2.5392719467	6.8273508802	1.7165683803
H	1.6791265321	-4.3598427566	0.7102569278
H	-4.287623255	6.6794058064	2.0028136157
H	1.5089678142	-2.5875353927	0.7133915224
H	-1.2279441395	3.7350484795	5.1144171488
H	-1.7005259468	-5.691794454	2.1288290321
H	-1.6381545482	2.0615853958	4.6590227334
H	-2.690329216	-6.0252963059	0.6886357289
H	-0.4691403885	2.9781074478	3.6930561932
H	-0.928455515	-5.7935120375	0.5400181871
H	-0.5998565769	5.4081094079	2.824985572
H	-0.7864639845	-5.6859791322	-1.7880283348
H	-0.1276019276	5.8592083031	1.1674013599
H	-1.6119678341	-5.4726491449	-3.3410185412
H	0.920095979	4.8034445824	2.1444931335
H	-2.5192968055	-6.0710129092	-1.9330421506
H	0.8267887205	2.8704498079	0.4909986195
H	-4.1221034653	-4.1326696869	-2.3352154515
H	-0.0384940785	4.0206911148	-0.5666529403

H -3.106137483 -3.5209633237 -3.6659881119
H -5.9403562559 4.0287147029 1.1707913532
H 1.5650800379 2.2372580185 -3.9687237897
H -7.5001358297 3.5096494202 1.861092069
H 0.6096666727 1.73335672 -2.5587466749
H -7.4527362987 4.2397097175 0.2436281888
H 0.1774099758 3.2187094742 -3.4413047343
H -5.0411348977 2.6681098148 -0.8488597333
H 0.4775750296 2.216011965 -6.1792272568
H -6.6068547155 3.0221653443 -1.6251819461
H -1.0902938739 2.9090215954 -5.7000219564
H -6.0521181121 1.3473326223 -1.4546619058
H -1.0038974528 1.2823975808 -6.4111120864
H -8.1868765909 0.4839574832 -0.8258631323
H -3.142367201 0.1446282938 -6.0344292588
H -7.403868825 -0.9432037334 -1.5356958682
H -1.947796744 -0.6740842372 -7.0742657724
H -8.5402997397 -1.1355828172 -0.1803695694
H -3.1986408139 -1.6201885454 -6.2420461584
H -5.5090397832 -2.0681204018 -0.2139943403
H -0.0460283187 -1.9235577232 -5.9429257465
H -9.3136443804 -0.0512348111 2.2127964223
H -3.8734762908 2.8159095371 -3.4258282984
H -6.7775636531 -2.3655886465 1.0080446942
H -1.3192280218 -2.9833733689 -5.2850903961
H -9.5705069273 1.555783355 2.9333535205
H -2.894097993 2.3107235436 -2.0160231924
H -5.2749305815 -1.5343188575 1.4550664427
H -0.1829141135 -2.1799401866 -4.1800138545
H -7.4306050344 -0.3629993167 4.1035785598
H -4.9559575868 0.6718029256 -3.7243193357
H -7.8948017614 1.2774034568 4.6183368263
H -4.1508477075 0.053063533 -2.2535575293
H -6.2103132832 0.9381012212 4.167262973
H -3.9781346358 -0.8076160444 -3.7986929593
H -9.1543826179 1.4218484225 1.2180659704
H -2.1109522057 2.9451637992 -3.4856332452
H -2.864763319 -0.8015206686 5.9218891622
H 3.5130426376 0.0449804585 -0.2290698008
H -0.7676797065 2.4464075443 -0.158406339
H -3.4460445132 -2.4821617745 -2.2638549615
H -2.7821793887 5.7526264712 -0.5053923625
H 1.7450774677 -4.2240932796 -1.7553174481
H 3.3782325599 -0.1525051045 2.2816719612
H -3.5272688544 0.1196120717 0.2237062346

4bb

C -0.0342067791 0.0895393792 0.0433413223
N -0.0309666983 0.0328892813 1.5097534517
C 1.3461142167 -0.0180672772 2.0537129788
C 2.2248013331 -0.1822894189 0.8055165254

C	1.4049818764	0.5075970793	-0.2964826677
P	-1.3595265414	-0.4252452792	2.4329831003
N	-1.6623378068	-2.0179710819	1.9392844147
C	-0.5300635151	-2.9684890735	1.8085101343
C	-1.1408265645	-4.3544151838	2.1029802457
C	-2.65379492	-4.1516977998	1.9162273459
C	-2.8676258872	-2.722612942	2.4228070977
N	-1.0271331976	-0.0572209968	3.904753518
P	-1.2533357257	-0.4807633335	5.5066229883
C	-0.7326404365	-2.2618273108	5.5565037615
C	0.6329388511	-2.6330171273	5.4498285668
C	0.9363224631	-4.0135409137	5.4439425878
C	0.0180994454	-5.0317442961	5.6618356831
C	-1.3058145133	-4.6363965499	5.8700535795
C	-1.6637247052	-3.2832214508	5.8039450184
O	2.2748002888	-4.2469360703	5.2302998164
C	2.8694684123	-3.0076696942	5.1571179317
C	1.9371450147	-1.9536225043	5.3272409931
C	4.2356829283	-2.848949659	4.9786906427
C	4.7100866395	-1.5370800685	5.0087104452
C	3.8344693309	-0.4700784027	5.2510709173
C	2.4539306848	-0.6319540272	5.4475868705
P	1.4107726864	0.8739024441	5.9133292741
N	2.62990116	2.0896611208	5.8348054146
P	2.5128721572	3.5196922751	5.2073263504
N	0.9908660375	4.0349826497	4.6612596911
C	0.5482976731	5.4497922229	4.6021701158
C	-0.216607759	5.5682827937	3.2750868192
C	-0.7760388968	4.1529170496	3.0757837787
C	0.3691479111	3.2573145767	3.5563217259
N	-2.9566441333	-0.6239310456	5.7392162592
P	-3.676774608	0.2677317773	6.8228048401
N	-3.569634228	1.9383285455	6.5741132847
C	-4.4169443165	2.9300657012	7.2707511298
C	-3.4254903009	3.978277269	7.7909590195
C	-2.3584584435	3.9967756229	6.6867219768
C	-2.2233271487	2.5173655469	6.2999177096
N	-3.2194472071	-0.1042676225	8.3976630422
C	-2.1317757701	-1.0569948765	8.7363462577
C	-2.0159474069	-0.9531416673	10.2666940987
C	-3.4068737164	-0.4806703188	10.7168529729
C	-3.8070492694	0.4959444836	9.6029919688
N	-5.3641605291	0.0623491564	6.8364844053
C	-6.0952727165	0.4464976762	5.6105678596
C	-6.1509522107	-0.8304428307	4.7399221444
C	-6.048065727	-1.9981441275	5.7595680102
C	-5.8409078904	-1.3046534653	7.1327417371
N	-2.7229623044	0.3782649169	1.8754760447
C	-3.4554762421	1.3286781332	2.7431315878
C	-4.7853413878	1.577209613	1.9962015524
C	-4.9148330187	0.384581842	1.032308514

C -3.4563870044 0.1283335182 0.6319600918
N 1.1670620167 0.5354548852 7.5379700136
P 1.7377641795 0.453596341 8.9711531335
N 1.0425528026 1.650850816 9.924233376
C 1.4697564362 2.1163124066 11.2483637816
C 0.8305281367 3.5075231844 11.3434763239
C -0.5204192109 3.293288448 10.6399537597
C -0.1777992346 2.3517245515 9.4712040978
N 3.3923882569 0.6570763483 9.3055281031
C 4.0266672186 1.9446136534 8.9272635026
C 5.3566217101 1.5553512151 8.2643872666
C 5.6782774915 0.1869476393 8.8838815139
C 4.2969053245 -0.4703101751 8.9714871878
N 1.3770460941 -1.0461166723 9.6462521134
C 1.3401249794 -2.2817628106 8.8309218184
C 1.8475946062 -3.3679786403 9.7873042369
C 1.3715223443 -2.870055287 11.1611656722
C 1.5764095042 -1.3477889545 11.0721907008
N 3.6146064137 3.686680484 3.942010339
C 4.2821587478 2.5435506005 3.2876069786
C 4.423573442 2.9962317621 1.8284512191
C 4.6116679418 4.5157201603 1.9588645783
C 3.6271915893 4.8805859057 3.0811161448
N 2.941348559 4.7967214677 6.2387234984
C 4.3664756115 4.9864768421 6.5775267899
C 4.3099960716 5.9987492873 7.7250064124
C 3.0845875079 5.5290254204 8.5270709587
C 2.1074066596 5.0150762802 7.4482740918
H -4.8925120818 0.5987853096 9.4961596936
H -1.7166807325 -1.9039940594 10.7181693403
H -1.2536018495 -0.2124821192 10.5313790351
H -3.3898878714 1.4974838307 9.8003008728
H -4.1087575533 -1.3245484164 10.7369771811
H -3.4095739326 -0.0130596171 11.707876453
H -2.3997570916 -2.0717457167 8.4155922452
H -1.1960646125 -0.7816015872 8.2441056806
H -3.8925928115 4.9540141301 7.9670059051
H -1.4500263042 2.0294787205 6.9016561283
H -1.3996724014 4.4151714687 7.0083798978
H -2.713013174 4.5900379599 5.8332861968
H -1.9487135828 2.3720692506 5.2531499826
H -2.0702414386 -5.3890172021 6.0526858228
H -2.7036399851 -2.9930065494 5.9278654648
H -6.795237042 -1.2195894456 7.6713490253
H -5.1340238685 -1.8260593833 7.7832988296
H -6.9427560122 -2.6312306403 5.7665061812
H -5.186189897 -2.6277525714 5.5219621724
H -5.6065008316 1.2899646852 5.1190546614
H -7.1062570498 0.7624377661 5.9059041387
H -2.941053827 -2.6985691659 3.5183566127
H -0.9315188299 -4.6340709957 3.1388393461

H -2.9265099108 -4.2163488181 0.8545195633
H -3.2531901069 -4.8838344947 2.468509342
H -0.7274762087 -5.133888903 1.4536461912
H -3.7714231041 -2.2573519268 2.0169728947
H -3.6052641999 0.8861924141 3.7306658429
H -3.2657725302 -0.8843793911 0.2658103692
H -3.1539447515 0.8326247115 -0.1620540972
H -5.3129368709 -0.4898701841 1.5613788708
H -5.5642998682 0.591055298 0.1741155085
H -4.7320624554 2.5118760981 1.4232415188
H -2.8949277279 2.2599063549 2.8796618246
H -0.7760973874 0.8104449327 -0.3189024248
H -0.2728430808 -0.8889215656 -0.4066962431
H 1.6987869056 0.2172815401 -1.311063172
H 1.5046139253 1.5981804462 -0.2119071441
H 3.2225175357 0.2480620308 0.9419021077
H 2.3477377349 -1.2470540173 0.5688077169
H 1.5756965137 0.9088565742 2.5901674059
H 1.4774552396 -0.8323150165 2.7694217805
H -5.017891791 2.4688938794 8.0575310257
H -5.1182053347 3.3882103313 6.554956534
H -7.0642412674 -0.8702502992 4.1350552491
H -5.6316389423 1.6609757662 2.686110278
H 0.2794554792 -2.7478169594 2.513904828
H -0.116757246 -2.9072569724 0.7930230924
H -5.2893809445 -0.8502763082 4.0676434129
H -2.9819020888 3.636273599 8.7349759008
H 3.9320452502 5.7740384996 3.6413890517
H 5.2489277104 2.4963960949 1.3103517334
H 3.4971668402 2.7778451829 1.280465448
H 2.6276202017 5.0782553934 2.6669722139
H 5.6397005639 4.7408641673 2.2711171522
H 4.4115735364 5.0655664768 1.0329400435
H 5.268399469 2.3614050939 3.7413611262
H 3.6988525251 1.6278394646 3.3955261462
H -0.9901373333 6.3435818074 3.3122909341
H 1.092291218 3.1075957399 2.736823183
H -1.0645832932 3.9320122014 2.0429777757
H -1.6578254865 4.0076135356 3.7100721167
H 0.0385065214 2.2729231302 3.896821614
H 5.7733711682 -1.3431383282 4.8815105982
H 4.2336734322 0.5344281899 5.339650726
H 4.9200006632 5.351232001 5.7071968911
H 4.8389504419 4.04984477 6.9105352546
H 4.1442115033 7.0067063116 7.323806814
H 5.2318099945 6.0133641538 8.3165658311
H 1.6107574339 4.0894728134 7.7632429006
H 1.3279555417 5.7545419569 7.2296836417
H 3.3950883586 2.5031388386 8.2321042185
H 6.3780291191 -0.4058968539 8.284987376
H 6.1424043685 2.3016154155 8.4280292528

H 5.1966100113 1.454371696 7.185771076
H 6.1050952051 0.3098755124 9.8881859946
H 4.1920755703 2.5541227363 9.8283477997
H 0.0270726064 2.9062415722 8.5486998166
H 2.559988 2.127442945 11.3267258579
H 1.0858879366 1.4692833578 12.0546001457
H 1.4373525893 4.2327063841 10.7864416058
H 0.7332176606 3.8615451159 12.3756695766
H -1.2230292374 2.8028672062 11.3269014711
H -0.9909125709 1.6555032538 9.2442978095
H 0.8571597877 -0.8007277809 11.6936928219
H 2.5868909222 -1.0639827926 11.4074046602
H 1.9144865507 -3.3143006746 12.0024999362
H 0.3058585089 -3.0967763762 11.2912908433
H 1.4646101694 -4.3604748354 9.5277472416
H 2.9441276338 -3.4115004135 9.7606736711
H 0.3168028766 -2.4929484436 8.4969869194
H 1.9547291179 -2.1880306943 7.9348811879
H 1.3932836126 6.1406574597 4.6737457153
H -0.1260015959 5.661197163 5.4438787349
H 2.6423277219 6.3205487979 9.1414361311
H -0.9855539994 4.2238027665 10.2974087085
H 4.0407851892 -0.925519735 8.0042765088
H 4.2412324782 -1.2465081883 9.7421758121
H 3.3703484681 4.7113878017 9.1965997013
H 0.4709301024 5.8207941237 2.457406866
H 4.8830590455 -3.709576469 4.8423476704
H 0.331129479 -6.0711334076 5.6717820982

4bbH^+
C 0.024588657 -0.0292254563 0.0219375452
N 0.0291519369 -0.0087144153 1.5122771546
C 1.4226816494 -0.0144760724 2.0228875243
C 2.234913988 -0.5433184878 0.835938272
C 1.5069152317 0.0747649356 -0.3664700753
P -1.117270898 0.8721078522 2.3584609084
N -0.9198489431 0.4067872454 3.9447633401
C -0.4580401525 1.3229935335 5.0167485771
C -0.4057266122 0.4188720461 6.2563693972
C -0.0877474649 -0.9698144652 5.68150708
C -0.9116175709 -1.0003077747 4.3864242523
N -0.9828839513 2.4531770073 2.381317909
P -1.4943837342 3.5802353204 1.3510979518
N -1.0747108093 3.2807829299 -0.1848135905
P -1.5681877519 3.5288661478 -1.661698758
N -2.8227930516 4.6197912134 -1.7629197077
C -2.9592692738 5.8189227142 -0.8942536788
C -3.7438265149 6.8112734456 -1.7609521748
C -4.6156528183 5.8950543873 -2.6335570597
C -3.6675559366 4.7360325401 -2.972018203
C -0.7117422087 5.140810436 1.8598332421

C -1.340084146 6.3575989451 2.2354314693
C -0.4650199601 7.4634902364 2.4024390605
C 0.9157903349 7.4383274156 2.2789208228
C 1.4989223712 6.2159561558 1.9539271001
C 0.6874852864 5.0980063626 1.7416979379
C -2.6923539043 6.898719272 2.535435147
C -2.4609452533 8.2733689333 2.7958761192
O -1.1338829904 8.610122093 2.7216690482
C -3.4262479538 9.2198976601 3.1096976231
C -4.7342700534 8.7574392984 3.1857761538
C -5.0150080067 7.4002672139 2.9768256522
C -4.0389925112 6.4400664567 2.6749584855
P -4.6215036907 4.6593104901 2.4625957765
N -6.2803468071 4.8747841501 2.6680674672
P -7.5147596175 4.0884736895 2.1072983687
N -8.4150297091 3.2123979581 3.2322353439
C -7.7422513324 2.1031086966 3.9551705443
C -8.4479806945 2.0370908667 5.3261745101
C -9.7546764748 2.8204401052 5.119112363
C -9.3305181268 3.9245358092 4.1474642142
N -3.9427536794 3.909205397 3.8052693401
P -4.0002176161 4.0060561213 5.3756492622
N -4.6897400399 2.6225709251 6.0431661538
C -4.3178434522 1.2961923809 5.4977610668
C -4.4348356369 0.3458406348 6.6980534218
C -5.5189855061 1.0058476338 7.5613254984
C -5.1965891397 2.5005601707 7.4283630239
N -2.429557263 4.1109153064 5.9579604657
C -2.0562908472 3.8118441183 7.3524051172
C -0.5610568145 4.1601744475 7.4131078225
C -0.4340249404 5.3185881623 6.4122502535
C -1.3829954209 4.9096438515 5.2783406029
N -4.8471419039 5.2305656635 6.1643373119
C -4.1986420931 6.5451253288 6.4085762423
C -5.3693193975 7.506367473 6.7229288518
C -6.5825579536 6.587517061 6.9545279159
C -6.3010629147 5.42230707 6.0048724698
N -2.5378347449 0.3365782988 1.6570109321
C -2.8566706778 -1.0957055311 1.477300282
C -4.1932332379 -1.2624665529 2.2075725439
C -4.9175109963 0.0454322754 1.8542230709
C -3.8009893816 1.1089976274 1.8391461237
N -7.1884473577 2.897973067 0.9502110598
C -8.1036375015 1.7705045222 0.6346728333
C -8.125278345 1.7057795947 -0.8983750032
C -6.7186035266 2.1925482481 -1.2717386468
C -6.4737998302 3.3318394634 -0.2749046326
N -8.602791462 5.1839875293 1.4679516426
C -8.4639985121 6.6520116903 1.5406415939
C -9.5501812227 7.1558256032 0.5789931366
C -10.6491304157 6.0882667071 0.7001651589

C -9.8473303985 4.7812540246 0.7900014136
N -2.054082719 2.1125479712 -2.3866520029
C -1.9643596859 1.7897630583 -3.8269631599
C -2.5872732005 0.3907326863 -3.8937463429
C -3.7215535637 0.475416204 -2.8603907708
C -3.1097298087 1.3097791865 -1.7222647877
N -0.3318029521 4.0561298441 -2.6773242769
C 0.9578644534 3.3353472655 -2.6484584793
C 1.8181796759 4.1343292371 -3.6404299535
C 1.2632975749 5.5849273542 -3.5585247945
C 0.0237053445 5.492223859 -2.6399071711
H -10.3759209222 4.0000349534 1.3494260374
H -9.8951304932 8.1633970555 0.8311467711
H -9.1609227039 7.1789666804 -0.4471712743
H -9.6320700003 4.3814646477 -0.2126035809
H -11.2195268307 6.2400631528 1.6254023734
H -11.3556873671 6.0902099996 -0.1356930069
H -8.6437730265 7.0126402861 2.5647025925
H -7.4579876579 6.9644553431 1.2502797335
H -8.3515657681 0.7012149572 -1.2706220935
H -6.8999123778 4.2734579687 -0.6575286448
H -6.6263958891 2.522176014 -2.3121550545
H -5.9940108516 1.3861209723 -1.1087609005
H -5.4148307856 3.5086612869 -0.0639839924
H -5.5415747505 9.4450222494 3.4264996372
H -6.0363764958 7.0510058895 3.0760604603
H -10.1693806756 4.3559625546 3.5939946114
H -8.8237558859 4.74035299 4.6861038089
H -10.5151596206 2.185183219 4.6487844036
H -10.1703513064 3.2180273722 6.0505972546
H -6.669635294 2.2978531795 4.0689660392
H -7.8668985328 1.1709733684 3.3912367452
H -6.5634557712 5.6689542568 4.9683377497
H -5.5515720492 8.1520861537 5.8578928727
H -6.6056152357 6.2280843254 7.9908238013
H -7.5369533443 7.0816227031 6.7428754033
H -5.1522106253 8.1514415416 7.579978744
H -6.8385437052 4.5086648805 6.2718850702
H -5.0146999501 1.0057477542 4.7012810588
H -6.0745967857 3.1330859585 7.5838798304
H -4.4473220498 2.8015337399 8.1717601067
H -6.5113537459 0.7945064849 7.1464562826
H -5.5101118645 0.6717019898 8.6035784159
H -3.4885671267 0.315934633 7.2517599091
H -3.3135613092 1.3041733602 5.064460339
H -2.2432251527 2.7577068529 7.5861729205
H -2.6307592029 4.4212864114 8.0675643252
H -0.236233009 4.418131301 8.4260489212
H 0.0361985966 3.3047868122 7.0755727341
H 0.5896771968 5.4754226859 6.0577238099
H -0.7778863756 6.2530045196 6.8738670975

H -0.8774773251 4.3010501499 4.5214303996
H -1.8092397518 5.7747384261 4.7697504999
H -9.091876069 1.9210149556 1.0746601265
H -7.6944418535 0.8399036075 1.0507114695
H -8.6134096378 1.0074469564 5.658899126
H -4.6784713566 -0.6788029707 6.3976604348
H -3.6400792039 6.9007541488 5.5344728808
H -3.49680779 6.4560130793 7.2453492683
H -7.8321636622 2.5339005359 6.0820853598
H -8.88166917 2.3920791338 -1.2989326098
H -0.4747681358 -1.655512952 3.6245551171
H 0.3327114021 0.7621139557 6.9873158185
H -1.3840376064 0.4045438926 6.7502664583
H -1.9368302722 -1.3485345135 4.5817490209
H 0.981169245 -1.0430891599 5.4448861252
H -0.3408734291 -1.7917337761 6.3583803519
H 0.5339518751 1.7291746009 4.7774035006
H -1.1470774084 2.1596013347 5.130012093
H -4.740136924 -2.1592278712 1.8978055848
H -3.7725594226 1.7000209582 2.7569394245
H -5.7156861544 0.3088385328 2.552869773
H -5.3712407705 -0.0397479211 0.8592453741
H -3.9423463501 1.8108147317 1.0133223913
H 2.5772910849 6.1338340252 1.8512952305
H 1.1520018771 4.1572391943 1.4588183865
H 1.5060639918 -0.6456837042 2.9130116509
H 1.7514383094 1.0004967302 2.2934739404
H 2.1747073063 -1.6382875112 0.7972431788
H 3.2911749775 -0.2636018801 0.8988577727
H -0.554767397 0.8002701569 -0.3860557018
H -0.4177453727 -0.9722699173 -0.3261035105
H 1.38878292 3.3639855508 -1.6348938785
H 1.9958728963 6.2867685687 -3.1486394333
H 1.6924730663 3.734031821 -4.6515825561
H 2.8817298429 4.0726578877 -3.3899279085
H 0.9824934695 5.9487171284 -4.5514904944
H 0.8261408828 2.2858926475 -2.9265872795
H -2.6707558779 0.6842434926 -0.9390699137
H -0.9272125463 1.8135971798 -4.1681436559
H -2.5341180013 2.5010072066 -4.4421920259
H -1.848615506 -0.3607046805 -3.5878058423
H -2.9350079871 0.1372273825 -4.8998626279
H -4.58215295 1.0002361323 -3.2931249915
H -3.8514154978 1.9601155081 -1.2467643023
H -4.1990449806 3.7971468558 -3.1620579407
H -3.0621384174 4.9691693868 -3.8609681015
H -5.0051180047 6.3867396844 -3.5302527306
H -5.4666872382 5.5226930656 -2.0511083944
H -4.3224478975 7.5132407191 -1.1530057133
H -3.0589231602 7.3926151205 -2.3913843427
H -3.5254288642 5.5608054291 0.0063588312

H -1.9856227247 6.2092436936 -0.5856381267
H -2.0551642924 -1.7320341962 1.8568233051
H -2.9750055693 -1.3168473226 0.4053215382
H 1.7267451531 -0.4278885019 -1.3139619768
H -4.0693229094 -0.5033153708 -2.5150354362
H 0.2814364063 5.8075059176 -1.6154891829
H -0.8170109387 6.0999677149 -2.984961345
H 1.7844321434 1.1310377434 -0.4706194866
H -4.0175588874 -1.3236484388 3.2892868318
H 1.4948706309 8.3424888052 2.436700184
H -3.1488794972 10.2544302042 3.2830726599
H -2.8823312604 3.8102613851 1.4400096852

5aa

C -0.0624466312 0.0497555809 0.1026243293
N 0.2075449296 0.1775494409 1.5458790488
C 1.6006627094 0.5780650936 1.7774377442
H 2.260743241 -0.1873584013 1.3515510899
P -0.9497196569 1.1373094998 2.3751842405
N -0.4368302178 1.1677727013 3.9911032732
C 0.2120342063 2.3473829503 4.5551530718
H -0.1392710315 2.5008883672 5.5851388758
C -0.1793352459 -0.0641607919 4.7215365792
N -1.0945893383 2.6095118014 1.8993178553
P -2.1596663309 3.4037022906 0.8441238518
N -1.4649041852 3.1880609553 -0.7186432676
P -2.2742726055 2.8004404288 -2.0084172246
N -3.5461349146 3.8471338856 -2.355418598
C -3.4857528964 5.2622127567 -1.9958577794
H -4.2905567534 5.5113016428 -1.2953610904
C -4.7931310242 3.4564704385 -2.9951058609
C -1.5872572359 5.132270168 1.2128868577
C -2.4720748234 6.1691103221 1.4097087456
C -2.0581210316 7.5166764244 1.6469376677
C -0.7347256059 7.8712924316 1.7232015268
C 0.2000325933 6.8070271931 1.5483327243
C -0.2095529019 5.5023018111 1.303592597
C -3.9202469924 6.6111480105 1.4485689526
C -5.2468562638 6.2371391817 1.342246892
C -6.1673524081 7.325222123 1.4112237046
C -5.7658920684 8.6431231611 1.6154096678
C -4.3959194521 8.9983686193 1.7587609015
C -3.5014222136 7.9571731008 1.6666362903
P -5.7273108256 4.4575122826 1.0373088821
N -5.3272823738 3.7403711741 2.5057342867
P -5.8625125403 3.8307662183 3.9743581975
N -4.5911105323 3.340915181 4.9736858012
C -3.1965991513 3.6169003352 4.6313177549
H -2.7844965233 4.4390514906 5.2397325843
C -4.81139553 3.0589879489 6.3828204842
N -7.4465406089 4.6392643942 1.0314199495

P -8.4506864193 4.0409344504 -0.0061733394
N -8.1524441548 4.5584362202 -1.5949581334
C -7.5611872766 5.8657691046 -1.8743702475
H -8.3192930188 6.5902914402 -2.2150017097
C -8.8461201219 3.9781397323 -2.7371973177
N -10.0642820511 4.4534072039 0.3645833393
C -10.5010024597 5.7941230116 -0.0290968822
H -11.5900645475 5.855930212 0.0838105676
C -10.503508187 4.1094316411 1.7221492881
N -8.5675741182 2.351581514 -0.0799449444
C -9.7723224722 1.5828422195 -0.3694557957
H -9.709583135 1.0938774459 -1.3560220675
C -7.3345897024 1.5932101323 -0.2650694948
N -2.3163876433 0.1457711562 2.2538649192
C -2.2936519635 -1.3126742868 2.2326163304
H -2.6150378651 -1.7365110609 3.1989377956
C -3.604665575 0.6958337645 2.6839274906
N -6.4458469371 5.2675616923 4.6724220378
C -5.4773803263 6.2911217027 5.0720649645
H -5.9370552327 6.9344634243 5.8337460423
C -7.7178770201 5.8369295553 4.2200751672
N -7.1791602806 2.801683689 4.2340899834
C -8.1185992893 2.8643647612 5.3424766619
H -9.1508988969 2.9295495959 4.9639020124
C -7.3444437144 1.6286724868 3.3857232121
N -2.9101381011 1.2284954185 -2.0729289082
C -3.037179015 0.407797336 -3.2696775838
H -2.6300224801 -0.5971436034 -3.080296938
C -3.7185011825 0.7612807686 -0.9517830162
N -1.2465983921 2.7805943508 -3.3755651814
C -0.024913311 1.9830566334 -3.214650112
H 0.4390619954 1.8413223076 -4.1991434546
C -0.9726420229 4.0817160738 -3.9915166824
H -6.8057704006 5.7679077354 -2.6646450361
H -7.0759867463 6.2642466863 -0.9841737465
H -8.1686491639 3.9524568718 -3.6014368941
H -9.1574862705 2.9551769559 -2.5223704568
H -6.4615212972 2.1849814667 0.0212340783
H -7.3548747611 0.6878292471 0.3566853041
H -6.5224686405 9.4237382104 1.6694964613
H -7.2253262386 7.0959198762 1.3111406498
H -10.2613548071 5.9823720788 -1.0775694075
H -10.0432285575 6.5859159674 0.5870471112
H -10.1609930089 4.8472072433 2.4626062246
H -10.1106048108 3.1317774003 2.0108641704
H -7.5532075328 6.6922735206 3.5554229742
H -8.2888311219 5.0955808886 3.6613623971
H -6.6057613362 1.6463911823 2.5851368121
H -7.9254534563 3.740279508 5.963108897
H -8.0508409295 1.9627870805 5.9732690024
H -7.2225746609 0.7012517474 3.9680838374

H	-4.097362919	2.2923135602	6.7126286633
H	-5.8174090847	2.6716005168	6.5528116233
H	-2.5894923866	2.720417135	4.8032487645
H	-3.1151076681	3.8833252243	3.5796828961
H	-10.6501375518	2.2295357461	-0.3577502568
H	-9.9013535125	0.791759097	0.3843290465
H	-5.1664064548	6.9194036964	4.2247708239
H	-4.5894366798	5.8316077685	5.5097044722
H	0.8896465584	-0.3388888713	4.7164441363
H	-0.7424114713	-0.895519414	4.2960115639
H	1.309671501	2.2374024254	4.5815217472
H	-0.0429434614	3.2227494938	3.9566710224
H	-3.7989888092	0.4966800194	3.749965572
H	-3.6677750625	1.7696652878	2.5091496347
H	1.2642504499	7.0283639993	1.6018633317
H	0.5341167008	4.7200965599	1.1738563547
H	1.8103715685	0.6463510504	2.8468747822
H	1.8412850831	1.546978537	1.3103658868
H	-1.0872161433	-0.2878533535	-0.0651579853
H	0.6233258818	-0.6952877807	-0.3199864859
H	0.698549244	2.4667854779	-2.5405889147
H	-0.2697074601	0.99945631	-2.8057569017
H	-3.4014761287	-0.2456066526	-0.6452874901
H	-2.4928858087	0.8607274196	-4.0989500884
H	-4.0933431934	0.2865472666	-3.5663166326
H	-3.6186837105	1.4267454725	-0.0920892566
H	-4.7968393772	2.3906169192	-3.2243118429
H	-4.9422053992	4.0083921629	-3.9379998835
H	-2.5343426252	5.4945141822	-1.5160486299
H	-3.5905903452	5.8915174707	-2.8946365999
H	-1.291876682	-1.6779703418	2.0026627944
H	-2.9890118623	-1.6800618278	1.4629246323
H	-0.3211011785	4.7159397799	-3.3678451367
H	-1.9077526627	4.6132990953	-4.1800709944
H	-0.3957815655	8.8891740234	1.8993285838
H	-4.0979961022	10.0319066293	1.9152336622
H	0.0630006703	1.0084059067	-0.4189763819
H	-0.4903610785	0.0563679277	5.7685618633
H	-4.4037802761	0.2234335173	2.0971098283
H	-0.4750730357	3.9154213349	-4.9547230143
H	-4.7835710714	0.7169338871	-1.2283857317
H	-5.6392695759	3.6677675545	-2.3265736392
H	-9.7388275482	4.5601747465	-3.0226452759
H	-11.6001872731	4.067137393	1.7454819834
H	-7.2095738783	1.2869768024	-1.3167167487
H	-8.3447279122	1.625386191	2.9282307337
H	-4.6703143342	3.9494646474	7.0198388755
H	-8.3024113678	6.1726620546	5.089470865

5aaH^+

C 0.0543093223 0.3397125399 0.2648065488
C 0.2008165279 0.4077057299 1.680247438
C 1.4063446824 0.1827872476 2.3019508923
C 2.4889750873 -0.12287507 1.4318508698
C 2.3281409334 -0.2156307902 0.0504763354
C 1.0743915246 -0.0160953995 -0.5973623642
C -1.2561256887 0.7842896398 1.819512023
C -1.3913514777 0.7556732825 0.3985979402
C -2.5698927035 1.0929720993 -0.2340283985
C -3.6681294144 1.4169622792 0.6211052832
C -3.5384358209 1.4155106617 2.0033440014
C -2.3040884393 1.104591865 2.6470429995
P -2.6721095501 1.2312649943 -2.033541256
N -4.2311424856 1.135825387 -2.4754926513
P -4.8993366228 0.1859464313 -3.5599837906
N -3.8969037492 -0.0484433387 -4.8989643116
C -3.2682584234 1.1289230015 -5.5120565755
P 0.777671886 -0.1509865076 -2.4380517704
N 2.3460457264 -0.6281758164 -2.9356392674
P 2.9826501166 -0.1257576249 -4.2880338213
N 2.2195351879 -0.4299723005 -5.7796353645
C 2.1550445279 -1.8184470939 -6.2508976403
N -0.2289484681 -1.5144195702 -2.5335576986
P -0.0648494487 -3.0365492133 -2.1367952178
N 1.4339140109 -3.8236362259 -2.1317323426
C 2.4274596855 -3.4728969133 -1.1098829092
N -0.588997875 -3.3909963847 -0.5746288805
C -0.2967485723 -4.6148191364 0.1693613375
N -1.0303742479 -3.8870184077 -3.2417493071
C -1.2457909568 -5.3218997178 -3.0857151815
C -1.6408262625 -2.5896988067 0.0380881228
N -1.9488594695 2.5540396563 -2.5684923541
P -1.4021788255 3.9664818256 -2.1378529942
N -0.7158357425 4.2386732157 -0.6403149618
C 0.6617218377 3.8251631821 -0.343929491
N -0.148044472 4.3091588688 -3.2010806265
C 0.4824934327 5.6280180058 -3.205711148
N -2.6716261951 5.0777878458 -2.1572232624
C -2.4542791353 6.4943309642 -1.8503329766
C 0.009414396 3.5598973436 -4.4473641864
N -5.2435168873 -1.3981547812 -3.1056361643
C -4.0884909239 -2.2118113803 -2.6880166779
N -6.3868396505 0.8469272824 -3.940064969
C -7.1377245765 0.2857472679 -5.0649016344
C -6.4225070212 -1.6532212165 -2.2663728609
C -6.7585520566 2.2155367807 -3.5795439984
C -4.0893215621 -1.1384966703 -5.8610726628
N 4.4524064141 -0.9390820194 -4.4875511612
C 5.2935986315 -0.6627894581 -5.6545754882
N 3.1406391558 1.5682789855 -4.3582564321

C 3.3927746344 2.306359226 -3.1198152582
C 5.2315398409 -1.3149515342 -3.3060503889
C 3.6836148033 2.2474461861 -5.5360901271
C 0.9724548862 0.2886342324 -6.0649816019
C -3.8270121045 4.8507061776 -3.0297056924
C -1.5331440495 4.4183918776 0.5660418315
C -1.2551826528 -3.4041155153 -4.5997638527
C 2.0643510831 -4.15366061 -3.4101837996
H -7.2824300509 -1.0873024074 -2.6255788411
H -6.2400344525 -1.3895400049 -1.2132324916
H -6.6586989396 -2.7216687542 -2.3198758796
H -3.8900373489 -2.0868428236 -1.6156969923
H -4.3004707886 -3.2696302195 -2.8799785226
H -3.1861782943 -1.9375421844 -3.2340258522
H -4.5854239252 -1.986121955 -5.389191737
H -4.6887478345 -0.8125378732 -6.724419972
H -3.1090021815 -1.4659827399 -6.2274403063
H -6.9352100716 0.8276336998 -6.0009728794
H -6.8860797346 -0.7677525576 -5.2074924438
H -8.2119334517 0.3514919245 -4.8546449642
H -7.8286358514 2.2444408462 -3.3399768333
H -6.1908172035 2.5352360758 -2.7062310964
H -4.6231628964 1.6659394949 0.1671093494
H -4.4014092925 1.666438299 2.6143182322
H -4.7131909736 5.3037411824 -2.5682748658
H -3.6862557826 5.307640011 -4.0222044422
H -4.008306403 3.7830337034 -3.1493691674
H -2.2221423788 1.1295500614 3.7299332763
H 1.208988735 3.631930667 -1.2663363877
H 1.1672815814 4.6260403917 0.211049538
H -2.5585105432 4.6772223442 0.3017549618
H 0.6764198909 2.9136405317 0.2640518617
H -1.5490079067 3.5026325044 1.1680329151
H -1.1088518031 5.2294321596 1.1719551793
H -3.3567164618 6.9021846338 -1.378791094
H -1.624120551 6.6157428726 -1.1528439139
H -2.2451293538 7.0828933578 -2.7561512327
H 1.5413161903 5.5210841782 -3.4727567718
H 0.0137672038 6.3089858612 -3.9321070944
H 0.4327688613 6.0825028572 -2.2143000899
H 1.0727545065 3.5312959967 -4.7076034957
H -0.5414573611 4.0244637297 -5.280730811
H -0.3340756989 2.5373461566 -4.3017012705
H -6.5695085306 2.9163752927 -4.406997519
H 3.4528196867 1.6873266639 -6.4431533442
H 4.774372985 2.380615526 -5.4697027466
H 3.2304759121 3.2443959612 -5.6224413522
H 4.4701395104 2.4645552782 -2.9508770047
H 2.9097667538 3.2906771366 -3.1791435315
H 2.9809457455 1.7733316447 -2.26310611
H 1.0496092333 1.3268010158 -5.7463590891

H 0.7908764087 0.2636914769 -7.1466687455
H 0.1097021832 -0.1663870903 -5.5526053036
H 5.886263088 -1.5564682025 -5.8862719652
H 4.6804739942 -0.424591195 -6.5251977067
H 5.9914680215 0.1695679463 -5.4739824856
H 5.9333782306 -0.5191827973 -3.0081918892
H 4.5607453688 -1.5254909459 -2.4748453715
H 3.1831926033 -0.4611301957 -0.5724154225
H 3.4731243719 -0.2978215981 1.8603360551
H -0.5945497804 -3.8976018023 -5.3320267495
H -2.2925957932 -3.6133651305 -4.8940489065
H -1.0863543147 -2.3271067485 -4.6331641186
H 1.54881682 0.2390707024 3.3772845051
H 1.9411509033 -3.1468805104 -0.1897232404
H 3.0423447769 -4.3555657833 -0.8907600923
H 1.3144193398 -4.429433889 -4.1538147096
H 3.0708961113 -2.6592258493 -1.4606930846
H 2.6540156295 -3.3109338087 -3.7935225542
H 2.7295877521 -5.0144901673 -3.266204162
H -0.4987986686 -5.9196883337 -3.6323650326
H -1.2112417579 -5.6091066428 -2.0348573367
H -2.2389008235 -5.5839235196 -3.4729974233
H 0.0826105628 -4.3601087067 1.1689315857
H -1.205205768 -5.2229977281 0.2998881563
H 0.4561750358 -5.212228126 -0.3447764559
H -1.3402720134 -2.2792011425 1.0470892648
H -2.5786880212 -3.1610997335 0.1167508366
H -1.8181891187 -1.6938256181 -0.552284488
H 5.8132471703 -2.2172170314 -3.5326343412
H -2.2890516134 0.8418640918 -5.9140177497
H -3.8803826209 1.5249564163 -6.337139695
H -3.1063345148 1.9194359821 -4.77880686
H 1.3293581293 -2.3702507626 -5.7790837371
H 1.9950975983 -1.814754089 -7.3360389458
H 3.0880199728 -2.341058229 -6.0384404031
H -1.9446016326 0.1348927083 -2.5215554724

5bb

C 0.07227532 0.4636932838 1.2462506019
N 0.0420174891 -0.1057350313 2.6092021515
C 1.4059175525 -0.0710256885 3.2004634398
C 2.2797281984 0.6214971627 2.13709598
C 1.2681283111 1.4182575143 1.2984670725
P -0.908209452 -1.4801418227 2.8754480274
N -1.1688623327 -1.3820979853 4.5379081017
C -1.2065200598 -0.1385630088 5.3364727887
C -2.325099823 -0.3846076978 6.356319744
C -2.2140774949 -1.8948466739 6.6097821873
C -1.926891684 -2.4630261319 5.212630334
N -0.2710939335 -2.7936236398 2.3287132464
P -0.5075481456 -4.4354038876 2.7745318324

C	-0.1685620354	-5.2023599857	1.1042069458
C	-0.0547646522	-6.5704994271	0.9318168368
C	0.2383989597	-7.1744394566	-0.3276490705
C	0.3369200208	-6.4430613523	-1.4889606585
C	0.1524536825	-5.0399751866	-1.3462170575
C	-0.075760556	-4.4502343655	-0.1057845192
C	-0.101923305	-7.9461287	1.5740695879
C	-0.4141726751	-8.6911924248	2.6912489429
C	-0.2446370684	-10.1009716735	2.529217406
C	0.1480742133	-10.6767185956	1.3274832471
C	0.3878160428	-9.8925248516	0.1601833507
C	0.2474807185	-8.5366217993	0.3189990223
P	-1.1309941575	-7.9388357998	4.2375513046
N	-0.3229232166	-8.9292784806	5.3617743112
P	-0.164118601	-8.771692669	6.906451293
N	-0.3566856614	-7.2355994994	7.5607438494
C	-0.8192661746	-6.9358657878	8.9329538695
C	-0.349075788	-5.4915231883	9.1794695907
C	-0.2678891908	-4.8952248902	7.7663977124
C	0.2905701058	-6.0576006763	6.941007693
N	0.8558133081	-4.8990065214	3.6547708918
P	2.3936098928	-4.6086780594	3.5644381144
N	3.2166654023	-4.3074737104	2.1214080045
C	3.4613382974	-5.4128738699	1.1619794996
C	3.6528726402	-4.7155083186	-0.2016753103
C	3.9575891488	-3.2518215678	0.1579928726
C	3.097759567	-3.02291618	1.4036340769
N	2.8073423219	-3.2043533368	4.4267242196
C	4.196463253	-2.817949285	4.6982904072
C	4.0317181709	-1.4532838973	5.3812780499
C	2.7321600811	-1.6151769249	6.211493988
C	2.0334326677	-2.8758403072	5.6352788875
N	3.1972639961	-5.9274894092	4.2377035666
C	2.5716369279	-7.251325619	4.3840768279
C	3.4819303443	-8.1670966022	3.5588778638
C	4.8934219741	-7.5918904442	3.8110370219
C	4.6579246115	-6.1048836116	4.218328119
N	-2.3714896245	-1.1252026339	2.118771798
C	-3.1911736231	0.0469903922	2.480001757
C	-4.046747336	0.3132603182	1.2273935363
C	-4.12994431	-1.0663303707	0.5547144478
C	-2.7271944527	-1.640803595	0.7829135897
N	-2.7082774639	-8.6177073159	4.1255130293
P	-4.0647509537	-7.8339474753	4.2051285709
N	-4.4001024282	-7.0390288928	5.652341548
C	-5.7164555174	-6.8699592436	6.2873959233
C	-5.3808299639	-6.0855146212	7.5630112964
C	-4.2456482659	-5.158919647	7.1012154804
C	-3.4185338432	-6.0525562634	6.1619887996
N	-5.3939266353	-8.8891548356	4.0993654566
C	-5.3681126643	-10.1056269193	4.9307563463

C	-6.6389049238	-10.8627327705	4.4961469162
C	-6.9228642367	-10.3561353129	3.0520453399
C	-5.7885547942	-9.3538959732	2.7567576994
N	-4.2781205417	-6.7023047292	2.991215
C	-3.5256009292	-6.746738168	1.7185931509
C	-4.025470494	-5.5159493724	0.9429805166
C	-4.5113078738	-4.5626513221	2.0471945269
C	-5.1375818373	-5.5168301234	3.0711172067
N	1.3612988909	-9.3206774243	7.3412434375
C	2.1846239311	-10.2256193527	6.5331879591
C	3.594019329	-9.9645896301	7.0750456638
C	3.3400787954	-9.7635834245	8.580375563
C	1.9860431565	-9.0201834603	8.635759101
N	-1.2128715419	-9.6676715995	7.8990161853
C	-0.8914352548	-11.0932235803	8.1323476898
C	-2.2234011865	-11.6855762508	8.603806077
C	-3.2372680762	-10.9358985819	7.7278196196
C	-2.6748990139	-9.5056436978	7.6838314487
H	-2.5687047489	0.9129425402	2.739520295
H	-4.3942841431	-1.0161191757	-0.5067480019
H	-4.8754788675	-1.6888318894	1.0626897849
H	-3.817088424	-0.1835678324	3.3545210875
H	-3.5282840911	1.0186876216	0.5651524712
H	-5.0249506647	0.7413715133	1.4707956587
H	-2.0324634294	-1.2777689705	0.008481731
H	-2.6937836524	-2.7315112865	0.7682821424
H	-2.2094072389	0.2205649262	7.2621911885
H	-2.8621350556	-2.6792499827	4.6739842483
H	-3.1106052746	-2.333950955	7.0577562928
H	-1.3693266361	-2.1016760638	7.278589479
H	-1.3493740788	-3.3917653743	5.2423646706
H	0.2162839897	-4.4058389828	-2.2287851232
H	-0.1712845477	-3.3701748221	-0.032921622
H	-0.8726934871	0.9673934463	1.0172098176
H	0.2398792009	-0.3118816984	0.4809777138
H	0.9848199041	2.3453320469	1.8134689218
H	1.6399622029	1.6793720455	0.3018835832
H	1.76405938	-1.0785922275	3.4321365329
H	1.383921862	0.5087886019	4.1326904703
H	2.0537381512	-2.8006823261	1.1443632631
H	2.7237214503	-4.7721559474	-0.7735668776
H	5.0195054131	-3.1263603839	0.4085152396
H	3.7140924151	-2.5541443505	-0.6514125636
H	4.444131472	-5.1864405109	-0.7949877881
H	3.4663162955	-2.207643519	2.0313609944
H	0.9882789237	-2.7087522838	5.3723452006
H	4.779892287	-2.782684078	3.7741721928
H	4.6896489847	-3.5287791313	5.3844142396
H	3.904982749	-0.6765149112	4.6189649272
H	4.9002914126	-1.1843362877	5.992335028
H	2.9542053672	-1.7526145784	7.2755767398

H	2.076835295	-3.7019820624	6.3612340691
H	5.0792091627	-5.9113540287	5.2157091383
H	5.1211901962	-5.3957402486	3.5260242848
H	5.5405410068	-7.6756105486	2.9310534076
H	5.3887638582	-8.1302934525	4.627894001
H	3.3973617112	-9.2200266264	3.8439248939
H	3.2072179957	-8.0893267965	2.5015323297
H	2.5674001187	-7.5466630801	5.4419080372
H	1.5402349211	-7.2234746044	4.0463348192
H	-1.3745282432	0.7392770283	4.7081888748
H	-0.245060143	0.0049521929	5.8526096077
H	3.0637509334	1.243933742	2.5814158077
H	2.0925400528	-0.7314385729	6.1258809175
H	2.6259616142	-6.1190267025	1.1272254103
H	4.3592549466	-5.9675952983	1.4604076804
H	2.7665062873	-0.1299717113	1.5051287611
H	-3.2994258274	-0.1470235586	5.9091301117
H	1.3725483631	-9.3620493171	9.4829391288
H	4.2899952277	-10.7836223983	6.8634812256
H	3.9977034956	-9.0478389811	6.6276100571
H	2.1206415258	-7.934887149	8.7484104009
H	3.2490116796	-10.7403465604	9.0721120763
H	4.1380933482	-9.2104660876	9.0870669748
H	1.9010359409	-11.2810383691	6.6899045209
H	2.0563919572	-9.9993539467	5.473832285
H	-1.0232445815	-4.9456672954	9.8491180012
H	1.3875738128	-6.1079363773	7.0421361819
H	0.3555772694	-3.998903504	7.7105923765
H	-1.2671359505	-4.6302087519	7.4042110829
H	0.0599417608	-5.9851815831	5.8757648718
H	0.254514272	-11.7581959033	1.2661976879
H	-0.4437206078	-10.7333608796	3.3907428047
H	-0.0902854826	-11.1920588166	8.8733011057
H	-0.5632531105	-11.595364487	7.2084790198
H	-2.385858977	-11.4526069255	9.6645635354
H	-2.2616823693	-12.7737720919	8.48304587
H	-2.8890874233	-9.0196964367	6.7279810599
H	-3.1013640994	-8.887732417	8.4872580844
H	-4.4618022958	-10.6935364161	4.7188551395
H	-6.937079597	-11.1675064387	2.3166501708
H	-7.4765332726	-10.6206100818	5.1593600326
H	-6.4882619413	-11.9467730273	4.5398219565
H	-7.8947008421	-9.8529992709	3.0067608638
H	-5.3562101955	-9.8492047273	5.9941061218
H	-2.611880647	-6.5646302543	6.6967308323
H	-6.1811891497	-7.8376596378	6.4956874384
H	-6.4157124607	-6.3024404669	5.6522668076
H	-5.0128792625	-6.7732868249	8.3356131435
H	-6.2466544405	-5.5492774675	7.9665896784
H	-4.66173313	-4.3074644364	6.5464815791
H	-2.9519369815	-5.4878969514	5.3451025275

H -5.137179575 -5.1018265262 4.0835089968
H -6.1829040723 -5.7544920309 2.8068824161
H -5.2224534061 -3.8116305092 1.6877516627
H -3.6566470105 -4.0495992158 2.5024174578
H -3.2321267297 -5.0895727334 0.3206451518
H -4.8626195532 -5.7882301128 0.2866782001
H -2.4564015599 -6.6733772341 1.916319485
H -3.7030104795 -7.683143446 1.1757493426
H -0.4160176143 -7.6403660049 9.6674242505
H -1.9149582894 -7.003645389 8.985432552
H -4.2621422431 -10.9741361129 8.1131709041
H -3.6485321814 -4.7583170804 7.9271195461
H -4.948974829 -9.8593535881 2.2501952615
H -6.1034409071 -8.509122845 2.1383095875
H -3.239206779 -11.3589935904 6.7165237412
H 0.6488087664 -5.4929332133 9.637600422
H 0.6503134742 -10.361166212 -0.7851402328
H 0.5352982197 -6.8873876108 -2.4609796043

5bbH^+
C -0.0498551221 -0.0850496841 -0.0280817916
N -0.0153438797 0.1151897156 1.44073047
C 1.3588448854 0.1420171384 1.9692991996
C 2.1749288569 -0.500286302 0.8407964355
C 1.4367534835 -0.0251041008 -0.4206007837
P -1.3281514767 0.4135539853 2.4039644242
N -1.2537824949 -0.72823009 3.6345935385
C -2.3222114587 -0.6843335088 4.6733638816
C -2.9315836695 -2.0908564574 4.6628684214
C -1.7339686364 -2.9727205023 4.28196006
C -1.0321625016 -2.1387775631 3.2022213107
N -2.6017040264 0.3014513497 1.4587604823
P -4.0127620226 1.0664904342 1.4801324572
N -4.7505940983 1.075375332 2.9289179413
P -6.1760660896 1.5279893428 3.4566701364
N -5.9821816566 1.9879207366 5.0466097816
C -4.6697616876 2.1418291704 5.7138095432
C -5.0440479301 2.6824731944 7.0965050935
C -6.3702093305 1.9629871313 7.3897525913
C -7.0874456373 1.9921401405 6.0303755102
C -5.0894166217 0.3299830182 0.2242279934
C -5.3282152621 0.9374696388 -0.9885712089
C -6.3571208537 0.4921088298 -1.874723637
C -7.1405083024 -0.6003659034 -1.5979949742
C -6.8674006843 -1.2678410848 -0.3671258384
C -5.8920353171 -0.81757925 0.5102537382
C -6.0428644983 1.6371609554 -2.8065219857
C -5.0041923141 2.0719736122 -1.9305778452
C -4.1627529778 3.1310364885 -2.2036993078
C -4.4439028293 3.7827286087 -3.4407678526
C -5.4773696387 3.3892318458 -4.2867905675

C	-6.3154863163	2.2772974342	-3.9915443463
P	-2.6470015457	3.4878283991	-1.1494110096
N	-1.8436519477	4.5742083236	-2.1858744559
P	-0.5183198434	4.2239601573	-2.9633613661
N	-0.3785306496	5.1834908503	-4.3233285101
C	-0.7397238263	6.613803753	-4.3329814598
C	-0.1949727383	7.12425305	-5.6795660037
C	-0.1872320308	5.8674819482	-6.5645346097
C	0.2530225083	4.77030538	-5.5872181177
N	-3.2965909026	4.386280566	0.121730915
P	-3.513074798	5.858142207	0.6074294277
N	-5.0204673231	6.4524917566	0.1434533096
C	-5.7122060335	5.9744690097	-1.074115052
C	-6.9683898562	6.8506318241	-1.1347839243
C	-6.4780639621	8.1945940934	-0.5754443288
C	-5.5341103842	7.774027998	0.562907527
N	-2.4385018374	7.0899050315	0.2170505409
C	-2.5011555601	7.7403440601	-1.1129837044
C	-1.2137801386	8.5708485566	-1.1457190254
C	-0.2099150688	7.6472012446	-0.4382012637
C	-1.0415996062	7.0549150929	0.7110220268
N	-3.4144977082	5.8745548379	2.2881286264
C	-3.3228298457	7.1009520028	3.1080333158
C	-4.3836068178	6.9050261492	4.1957910905
C	-4.300591313	5.3939553971	4.4498095465
C	-4.1025552528	4.802568891	3.0427474842
N	-1.1553994676	1.8753881402	3.1981188876
C	-0.6518330669	2.060070638	4.5796360798
C	0.2138915461	3.3247501219	4.4953754268
C	-0.4686592778	4.1417668144	3.3886761113
C	-0.8696372888	3.075371913	2.363638041
N	-0.3753583114	2.6360887142	-3.482103877
C	-1.4914027872	1.9183235938	-4.1441977398
C	-0.7940000623	0.7924310953	-4.9230080233
C	0.4616622774	0.5168775024	-4.0814379315
C	0.903408229	1.9262222738	-3.6686429951
N	0.8741380016	4.5094807107	-2.0708231784
C	2.1602639472	5.0032947468	-2.586969247
C	2.9889367464	5.198023811	-1.3091991144
C	2.508433195	4.0424926244	-0.414308279
C	0.9993771444	3.9792306222	-0.7002770567
N	-7.2910727611	0.2851118353	3.4424345187
C	-8.6970567283	0.3624260283	2.9990357155
C	-9.166726269	-1.0965448134	3.0818112842
C	-8.3740661514	-1.6518175929	4.2757330056
C	-6.9935233966	-0.9982738309	4.1085219957
N	-6.9098871134	2.7856319235	2.6425025581
C	-7.065950353	2.8126415796	1.1665369439
C	-8.2079152302	3.8081981203	0.942110103
C	-8.0267758415	4.7960431747	2.1030738657
C	-7.6875885178	3.8705387933	3.2798204845

H	1.4287855961	-0.4054365507	2.9156262182
H	1.6574422032	-0.6324273671	-1.3032326647
H	1.7238947605	1.0083379146	-0.6452868047
H	1.6886665281	1.1767656456	2.1563813422
H	2.1244289839	-1.593189672	0.9218150175
H	3.2289167099	-0.2061289436	0.8648964071
H	-0.489111026	-1.0623236177	-0.2665489368
H	-0.6564549446	0.6841841939	-0.515805615
H	0.2748923521	3.8514518232	5.4533295212
H	-0.0371206436	2.8646058191	1.6783956937
H	0.1805222031	4.9063076472	2.9508543862
H	-1.3609534629	4.6484256718	3.7718176776
H	-1.7302681635	3.3708242036	1.758910132
H	-7.4497324492	-2.1472894641	-0.1037446242
H	-5.7424899521	-1.337921141	1.4508566207
H	0.0392604058	-2.3498220826	3.1267993052
H	-1.4885043671	-2.3259760632	2.2204777026
H	-1.0736868639	-3.1091930928	5.147642295
H	-2.0182686384	-3.9634925401	3.9140199506
H	-3.0624679843	0.0826601647	4.4359985426
H	-1.8727516595	-0.4516621799	5.6479694164
H	-6.3291940227	-1.6133634854	3.4862444591
H	-8.8854145831	-1.6248525529	2.1626823199
H	-8.8342536783	-1.3251131968	5.2169261462
H	-8.3194441836	-2.7445896527	4.2909641956
H	-10.2509996308	-1.1786390581	3.2040100849
H	-6.4829897202	-0.8365654455	5.0664974644
H	-4.1708923576	1.1702578179	5.8077620274
H	-7.7523937968	1.1340897084	5.8825433032
H	-7.6936423428	2.9027644555	5.9313692248
H	-6.174148809	0.9259341054	7.6898691679
H	-6.960175869	2.4363937442	8.1802456808
H	-5.2013732304	3.7674628349	7.0519671778
H	-4.0101149144	2.7991924565	5.1457574679
H	-7.0982388798	4.3659102621	4.0558657028
H	-8.6080552185	3.4846405597	3.74353467
H	-8.9119139394	5.4084051429	2.301346364
H	-7.1825278676	5.4637656715	1.8925379325
H	-8.1541962857	4.2728597262	-0.0456469332
H	-9.1767021172	3.2993704405	1.0254443985
H	-6.1415546723	3.1691366507	0.6954757851
H	-7.2899327503	1.8219219264	0.7633469557
H	-0.0960687892	1.1826596329	4.9209613512
H	-1.4995122672	2.2093165251	5.2635034997
H	-3.3841984867	-2.3605357439	5.6229253196
H	-4.2687364815	2.4827853733	7.8429980647
H	-8.7725319062	0.7630195795	1.9846136982
H	-9.2919083528	1.0059297201	3.6649240453
H	-3.7073011319	-2.1478360969	3.8889029973
H	1.2344703063	3.0584192586	4.1914791521
H	-4.7215673246	8.4942848306	0.7107184748

H	-7.3742721921	6.9229992937	-2.1486685686
H	-7.7517436375	6.4410667598	-0.4855356811
H	-6.0763851048	7.686254955	1.5160631799
H	-5.9155642516	8.736189334	-1.3463269056
H	-7.2867859957	8.8468286042	-0.2304681307
H	-5.0955953975	6.1202752898	-1.9716429382
H	-5.9264032358	4.9057536082	-1.0026493023
H	-4.1938474477	7.5091585656	5.0891978086
H	-5.0634116991	4.5545201396	2.5809227971
H	-5.1817546654	4.9826421774	4.9495793507
H	-3.4305057319	5.1729601106	5.0821490234
H	-3.5109511896	3.8831480147	3.0552643569
H	-5.6382977813	3.9359487681	-5.2135881699
H	-3.7996763229	4.610191102	-3.7282637155
H	-3.4036365269	8.3542361329	-1.1987631283
H	-2.4985285935	6.9905978591	-1.9143481011
H	-1.3466183055	9.4993742137	-0.5755039253
H	-0.9180603357	8.841724001	-2.1638897781
H	-0.7363622583	6.0359408538	0.9670700346
H	-0.9547528569	7.6656572098	1.6200874334
H	-1.8288806558	6.7273410867	-4.2591586219
H	-1.1992116596	5.6532281647	-6.9303053437
H	0.8298580715	7.4962999453	-5.5541900762
H	-0.7980161753	7.9403946903	-6.0895336058
H	0.4781946368	5.9506984853	-7.429513697
H	-0.2935167131	7.1436980122	-3.4842927562
H	0.4296742251	4.6034327945	-0.0015434028
H	2.0204105564	5.9310512816	-3.1516600461
H	2.6461368175	4.2736283457	-3.2547327351
H	2.7385900508	6.1612284338	-0.8476961032
H	4.0664212676	5.1806066144	-1.5001200164
H	2.9923261811	3.1068703908	-0.7236664732
H	0.5942928636	2.9635216066	-0.6245291104
H	1.4937050139	1.9372140447	-2.7480825577
H	1.5149487426	2.3830591555	-4.4632459206
H	1.2430052519	-0.0247304191	-4.6241615953
H	0.1950402537	-0.0649667481	-3.1906928263
H	-1.4360865673	-0.0850066929	-5.0473812255
H	-0.5043184996	1.1417344742	-5.9224720254
H	-2.1777926728	1.5138496242	-3.3910932814
H	-2.0718377536	2.5856863069	-4.7911453346
H	-3.4692140781	7.9999535461	2.5069183326
H	-2.3228690761	7.1697160291	3.5630437184
H	0.6837086098	8.1691167055	-0.0791982437
H	2.7267837847	4.1954764565	0.647799602
H	-0.0849083233	3.7735361595	-5.8857566282
H	1.3522951335	4.7433910963	-5.5057117361
H	0.1034142777	6.8457441405	-1.1156242208
H	-5.3749301035	7.1713141952	3.8065690744
H	-7.0952855606	1.9634569537	-4.6797539877
H	-7.9305524954	-0.9505639491	-2.2563294908

H -3.8691316908 2.3934176943 1.0396457541

6aa

C -0.1859471947 0.0795738728 -0.1862881724
C -0.1210990714 -0.0259533026 1.2222199227
C 1.0876756166 0.0190153236 1.9767388986
C 2.2777254247 0.3018598583 1.2197793849
C 2.1925659417 0.3921395418 -0.1656753844
C 0.9819928421 0.2621075811 -0.8920759211
C 0.9452378058 -0.212559126 3.3992139361
C -0.33479147 -0.3128208559 3.9279971032
C -1.5265153681 -0.2650852731 3.1537146039
C -1.4136399678 -0.1527705063 1.7889049908
P 2.43410338 -0.4009221793 4.5428954512
N 3.1205870323 -1.8135813986 3.9073778925
P 2.6817139161 -3.3111185314 3.8132955971
N 1.0708457744 -3.8286728225 3.6410443099
C 0.4228551349 -3.6469402191 2.3395991302
P 3.9058413447 0.7898184477 2.0344804092
N 3.8099338255 2.4848615452 1.8201067502
P 2.980911265 3.6389945326 2.4496378222
N 3.6043020858 5.0902831397 1.8122173825
C 2.9433152622 6.3798661986 1.9469588462
N 4.9625245638 0.3120400882 0.7819061347
P 6.4657253151 -0.0358609836 1.0575384214
N 6.8682738771 -1.3891608152 0.1134069396
C 8.2358737292 -1.8885645538 0.0528601809
N 7.4651788402 1.303608491 0.7387719321
C 8.917924466 1.2449817242 0.8493350692
N 7.0393834588 -0.5034464025 2.5855754401
C 7.1173029489 0.5095980682 3.6454436994
C 6.9873314118 2.3252057832 -0.1916621493
N 1.6484335001 -0.9294806075 5.9907189738
P 1.6053194869 -0.1691337088 7.3537156362
N 1.3222975642 -1.3046054588 8.5857492018
C 1.369171187 -0.8638436221 9.9767769857
N 2.9774455423 0.7876561627 7.7009291415
C 3.0201220255 1.799176252 8.753092516
N 0.3671176667 0.9738360869 7.6331295634
C 0.3742907326 2.123255435 6.7165692581
C 4.2931027948 0.2119233503 7.4244449745
N 3.4889414791 -3.9685303108 2.4737804923
C 3.3947761183 -5.3874538129 2.1629965804
N 3.1300083268 -4.1794878813 5.2051731246
C 4.3515894079 -3.7782884925 5.89492045
C 3.8474184302 -3.1436480049 1.3217647472
C 2.7151771676 -5.5385014951 5.5276898237
C 0.119987665 -3.7299491896 4.7508284199
N 1.3310446613 3.8324159576 2.1133036721
C 0.2694148191 3.2705436412 2.9481713891
N 2.9679347598 3.6730797661 4.1617397784

C	4.0747157119	3.024419179	4.866463324
C	0.9522151315	3.9252194485	0.7011409276
C	2.4948593463	4.8737473506	4.8456413468
C	5.0284416002	5.1903051289	1.5182699926
C	6.5944342475	-1.8024308333	3.1174383787
C	6.0713929704	-1.7047560225	-1.0690173519
C	-0.9931615958	0.4897930747	7.88008403
C	1.779616971	-2.6798147966	8.3964892764
C	-1.6295930625	-0.0385953463	-0.6400687958
C	-2.4574553649	-0.1082642292	0.6878601785
H	3.1113756839	0.5969876813	-0.710291488
H	-0.4201982734	-0.4866900851	4.9962654268
H	5.4434070162	4.1915399887	1.3878249794
H	0.6415456764	-3.7360248447	5.7064769237
H	1.7611277321	4.3739342932	0.1201857037
H	3.8987649736	-2.096714604	1.607848656
H	5.176887003	5.7590244688	0.5885182483
H	-0.4641680456	-2.8044765275	4.6975777919
H	0.7293882781	2.9350476465	0.2796344355
H	4.8316629481	-3.4496261932	0.9458287443
H	0.059708875	4.5598297365	0.6060014113
H	3.1197864194	-3.2472329641	0.4993412996
H	1.8739318582	6.2444794582	2.1172682319
H	1.1482746054	-3.7280630772	1.5289889385
H	3.3600263495	6.9790809483	2.7740606164
H	-0.3326373496	-4.4332042874	2.2041230413
H	3.0730271775	6.9567135239	1.0191545185
H	-0.070168592	-2.6697826382	2.2615441452
H	-0.536405276	4.0112476265	3.0648512406
H	2.578813678	-5.6069268779	1.4529375637
H	-0.1560345916	2.3649725119	2.5013245716
H	4.3361705728	-5.7234362974	1.7068738079
H	0.6580322751	3.0084646059	3.9304269571
H	3.2281802874	-5.9806369683	3.0618277883
H	1.628774962	5.2968652459	4.3320018933
H	1.8365535057	-5.8234024727	4.9473590006
H	3.2747613493	5.6513927287	4.9136277341
H	2.4590100031	-5.6026241451	6.5960529761
H	2.1892111052	4.6142705513	5.8679721107
H	3.5194340526	-6.2685200551	5.3348910147
H	3.7470901759	2.7751739473	5.8827190543
H	5.2114988803	-4.3992645548	5.5922841617
H	4.964672074	3.6755554264	4.9311221441
H	4.2144425316	-3.8857818113	6.979481171
H	6.1744640928	0.5700275869	4.2068021098
H	-0.1707686747	1.9012991422	5.7881010137
H	7.3308228486	1.4920217009	3.2209536093
H	1.3964395999	2.3937894778	6.4462590218
H	7.9260532547	0.2394364096	4.3392192022
H	-0.1016009635	2.9824202688	7.2071195818
H	5.5730575679	-1.7511107332	3.5180994627

H -1.5046019283 0.1908528752 6.9509231974
H 7.2849806713 -2.1019644052 3.9173486572
H -1.574957322 1.2968002948 8.3440433199
H 6.6242351312 -2.5611944234 2.3342842979
H -0.9790471456 -0.3670187662 8.5549201813
H 8.7471087913 -1.7144451685 1.0019523139
H 0.9665147986 0.1471080225 10.0735472287
H 8.2173523068 -2.9721768404 -0.1281849489
H 0.757794821 -1.5370042497 10.5923487807
H 8.820928015 -1.4216268424 -0.7572132015
H 2.3951031799 -0.8714430248 10.3821244486
H 6.0567934385 -2.7939915766 -1.2143649837
H 1.0858211528 -3.3690587457 8.8973879433
H 9.4011143804 0.9778867321 -0.1053252473
H 3.4373943361 1.3997406173 9.6923303385
H 6.4861570251 -1.2433957028 -1.9813719648
H 2.784646792 -2.8353605535 8.8244880101
H 9.2993574976 2.2298336868 1.1536016495
H 3.6598857732 2.6332125358 8.4300874134
H 5.0506482806 -1.3494579224 -0.9254486889
H 1.8169214919 -2.9085324716 7.3310628563
H 7.3099695479 2.121848634 -1.2273414971
H 4.7087337037 -0.2969526756 8.3112349978
H 7.3963477706 3.3006483545 0.1051488559
H 4.9872535943 1.0096137757 7.1295429026
H 5.8983140796 2.3714096678 -0.1563363783
H 4.2367050956 -0.4988738271 6.6004540987
H 9.2138369823 0.5183313974 1.6085114561
H 2.0204091506 2.1854547115 8.9539823415
H 0.9877548524 0.3421229631 -1.9780751875
H -2.4949258024 -0.3511457492 3.6444499034
H 4.3393105164 2.0902743952 4.3733647288
H 4.5737662441 -2.7346929758 5.6721098639
H 5.5763790659 5.7088107159 2.3243529768
H -0.5673704342 -4.5877408583 4.7112208438
H -3.1042231075 0.7721254656 0.7975016328
H -3.1163083394 -0.9850211697 0.7142781037
H -1.7773296241 -0.9420591955 -1.2462911594
H -1.9315552282 0.8101186804 -1.2661487099

6aaH^+
C -0.3803484868 0.2167081648 0.0205896969
C -0.2639910275 -0.0155429508 1.4101276607
C 0.9714916975 -0.0060024986 2.1246025611
C 2.1299805765 0.3518475399 1.3604604091
C 1.99116033 0.5603689523 -0.0105821285
C 0.7606959735 0.4838420232 -0.7039916194
C 0.8515144328 -0.3319752778 3.5335984674
C -0.4113316177 -0.5040450406 4.0938043799
C -1.6194487761 -0.4265084147 3.3572379661
C -1.5360832803 -0.2128075312 2.001078919

P 2.2530251475 -0.7862818925 4.6139439429
N 3.093378642 -1.9968371261 3.9639758446
P 2.7135155937 -3.5297851649 3.7793820191
N 1.1124451281 -3.9899424827 3.5464227728
C 0.5016518868 -3.8211636979 2.2230308631
P 3.7971326117 0.793835406 2.1238686132
N 3.8077999105 2.4730023569 1.8625477619
P 3.1255425063 3.7114863748 2.5161274405
N 3.991692596 5.0807092765 2.0475307834
C 3.5179004331 6.4473521278 2.2435370767
N 4.7824119566 0.1881371852 0.8937866279
P 6.3344358299 -0.0206279748 1.0062966847
N 6.7280640705 -1.3711231041 0.0603983754
C 8.1176041602 -1.8022247235 -0.0869336169
N 7.1838605021 1.3796810351 0.5611766055
C 8.6473683327 1.404280735 0.5197042097
N 7.0697735996 -0.4032289569 2.4885348457
C 7.2116340768 0.6484726749 3.5012238631
C 6.5665075693 2.2565114944 -0.4410722234
N 1.6944847464 -1.1146254237 6.109344719
P 1.5991588055 -0.2072197 7.4022026936
N 1.2521642963 -1.2302584404 8.6826997888
C 1.2622087057 -0.6939309136 10.0464738706
N 3.0059864773 0.7109602576 7.6197563095
C 3.0815513824 1.804228166 8.59619905
N 0.405265433 0.9730401076 7.4764323727
C 0.4788908698 2.0764882683 6.5063634628
C 4.3002041068 0.0418665996 7.4374259884
N 3.5794117982 -4.0549472595 2.4498018998
C 3.6168747597 -5.4776737785 2.1166826368
N 3.126696159 -4.3987449338 5.1544798783
C 4.3021067199 -3.9954409949 5.9278150877
C 3.9166324152 -3.1751413088 1.3269687479
C 2.6802113541 -5.7600777067 5.4516651905
C 0.1311404008 -3.9536750915 4.6370079422
N 1.5381495197 4.1391837784 2.0973837685
C 0.4002573194 3.4293008948 2.6903866753
N 2.9889965235 3.6178859561 4.2337314008
C 4.1584525699 3.0481884165 4.9118701085
C 1.3035982351 4.453587276 0.6816844999
C 2.4832052164 4.7937128675 4.952712937
C 5.417221783 4.9870724043 1.7435137692
C 6.7346147652 -1.700407237 3.0924934912
C 5.8951911248 -1.6948408723 -1.0994903219
C -0.9752931529 0.6336620546 7.8374294002
C 1.5262211058 -2.6673730078 8.6069745788
C -1.8351357567 0.1259996301 -0.3958877789
C -2.6127217544 -0.1136512808 0.9396804535
H 2.8890723591 0.8181525989 -0.5669406258
H -0.4708735489 -0.7508932634 5.1484228897
H 5.6989592366 3.9424380932 1.6174488624

H	0.6313587966	-3.8839629801	5.6027579089
H	2.1534129587	4.9961585866	0.2635387584
H	3.8779562388	-2.131314651	1.6259111187
H	5.6364191795	5.5278986041	0.8121269223
H	-0.5350681465	-3.089710833	4.5345221085
H	1.1441485406	3.541169391	0.0880166692
H	4.9359692617	-3.3940876656	0.9930378498
H	0.4115146866	5.0870425665	0.6016717222
H	3.2339864912	-3.3275661889	0.4769808198
H	2.4460321918	6.458516222	2.4447832556
H	1.245301247	-3.9402182536	1.4343745473
H	4.0400972255	6.9417425502	3.0770933271
H	-0.2676625727	-4.5907666692	2.086996389
H	3.7039077249	7.0362196734	1.3341408647
H	0.0354768331	-2.8325256054	2.1161604465
H	-0.4526125944	4.1169493858	2.765836307
H	2.8350130943	-5.7519054567	1.3915410747
H	0.0988405702	2.5692843489	2.078965966
H	4.591492438	-5.7149928047	1.6740399464
H	0.6504002854	3.066160047	3.68703095
H	3.4936338999	-6.0963661709	3.0059985271
H	1.6044664498	5.2042075716	4.4524337898
H	1.8511292136	-6.0482192851	4.8048174385
H	3.2415593107	5.5884341115	5.0365839557
H	2.3421495922	-5.8158846561	6.4953517153
H	2.1952101637	4.4979012099	5.9691080092
H	3.4981827051	-6.4841650258	5.3225836955
H	3.8661418704	2.738924472	5.921910492
H	5.1579788584	-4.6554636523	5.7236095672
H	4.9866618719	3.7722613852	4.9988007594
H	4.0699553673	-4.0479039529	6.9994660998
H	6.3262870369	0.6965895752	4.153180077
H	-0.1572070725	1.8741741129	5.6346821536
H	7.3430921726	1.622106137	3.0274467644
H	1.5000448894	2.218593432	6.150402863
H	8.0913731689	0.4341393089	4.1223724333
H	0.1400315562	3.0051125509	6.9814756523
H	5.744873081	-1.6856842991	3.5716929874
H	-1.5692953707	0.3597291804	6.9528669357
H	7.4955972009	-1.9414565681	3.8453070942
H	-1.4431036737	1.5075773176	8.3060198903
H	6.7397326231	-2.4842759416	2.3341231045
H	-0.9981295048	-0.1971939836	8.5426210306
H	8.6822645451	-1.5898958673	0.8228662256
H	0.9855514675	0.3625844047	10.0481739781
H	8.1396410253	-2.886507588	-0.2574138452
H	0.5303928753	-1.2384187519	10.6549181982
H	8.6192406309	-1.3166410068	-0.9384381382
H	2.2502977265	-0.8007652147	10.5187145543
H	5.9314135201	-2.7781370708	-1.2752915713
H	0.7431931614	-3.2123452437	9.1484656258

H 9.0415944497 1.0949657478 -0.4607746198
H 3.4153736306 1.4476042785 9.5819510843
H 6.2478394046 -1.1900398696 -2.0132576991
H 2.4977951204 -2.9139864437 9.0622251087
H 8.9946144785 2.4270011084 0.7149722139
H 3.804485495 2.5496062484 8.2434329771
H 4.8642066052 -1.3980654073 -0.9056180131
H 1.5339743407 -2.9865228672 7.5651529411
H 6.7563430679 1.9063948996 -1.4690618986
H 4.6857830216 -0.3549769021 8.3891676994
H 6.9971540838 3.2600210386 -0.3418061479
H 5.0275320535 0.7616939418 7.0438153168
H 5.491912068 2.3176373058 -0.2685522137
H 4.2173309023 -0.7825862009 6.7287355049
H 9.0645400571 0.7503672081 1.2876852
H 2.1111766866 2.2880723331 8.7079834164
H 0.7274755713 0.6643119839 -1.7763550945
H -2.5723658949 -0.5653962786 3.8621054542
H 4.5153366994 2.1685613221 4.3755764107
H 4.5802334567 -2.971083599 5.6804943049
H 6.0238738219 5.4325059299 2.5480096625
H -0.475166677 -4.8686765283 4.613132689
H -3.2996051901 0.7126666871 1.1600534124
H -3.2229336488 -1.0238382567 0.9034190889
H -1.9915919157 -0.6973116052 -1.1036564323
H -2.1673604934 1.0396813799 -0.9024680831
H 3.1243787825 0.3017978788 4.6707651413

6bb
C -0.0301834292 0.0669804502 -0.0148722843
N -0.0622140124 0.1162509008 1.4633335586
C 1.295407706 0.159437806 2.017742527
C 2.1486953875 -0.4698891099 0.9094511027
C 1.4721307006 0.068656189 -0.3626752535
P -1.4892414352 -0.1672259493 2.3082647609
N -0.9772265557 -1.3004627242 3.4641248204
C -1.663183662 -1.4619896021 4.7575923429
C -0.8771194037 -2.6005909342 5.4149920073
C -0.5886327748 -3.5415601149 4.2308842177
C -0.4110685405 -2.5981333632 3.0215931163
N -2.6793261246 -0.4254294378 1.332232536
P -3.6105728414 -1.7048117791 0.7428332913
C -4.2837910602 -2.4233668342 2.3506212384
C -5.2175328279 -1.6964238358 3.1840627896
C -5.5013752231 -2.2935164341 4.4469170994
C -5.0310559767 -3.5660977088 4.8558884907
C -4.2349058583 -4.2817554833 3.9954482105
C -3.8628030864 -3.6811797124 2.7619969688
C -6.3013983608 -1.6980322654 5.4497756665
C -6.8664091241 -0.4693699235 5.197433659
C -6.6664834542 0.1142465786 3.9211286026

C -5.9069405535 -0.4603010518 2.9082272695
P -5.8751077355 0.3795045357 1.2038034833
N -6.3346629751 1.9482840521 1.741587436
P -6.069963983 3.272888678 0.9454482778
N -7.2721879423 3.8329990719 -0.1141228807
C -7.6195418925 2.9680526372 -1.2740188894
C -9.1532146038 2.8892517438 -1.2609504047
C -9.5641774573 4.2125801935 -0.6001274664
C -8.5062814865 4.3791057936 0.4974976491
N -2.4622748293 -2.9410352485 0.365693935
P -2.0975679333 -3.5279701358 -1.0336950062
N -3.3368212846 -4.4444910465 -1.7078900649
C -3.2021252991 -5.2954810214 -2.9029676163
C -4.2150965679 -6.42281568 -2.6575055988
C -5.3404212799 -5.6973145795 -1.9030239011
C -4.5837619226 -4.7430486287 -0.9652602674
N -1.5379404952 -2.4025490458 -2.1690395858
C -0.8919878473 -2.7513661955 -3.4401443636
C -0.3644233562 -1.3900561431 -3.9244898734
C -1.4038107821 -0.3660600022 -3.3893456809
C -2.3100198289 -1.1729755787 -2.4267450098
N -0.7593309759 -4.5715305279 -0.9772753362
C 0.5041841597 -3.9657096645 -0.469144549
C 1.0850919744 -4.9897559446 0.5298740791
C -0.1388233361 -5.8093585963 0.9685968279
C -0.9552948357 -5.8869348644 -0.3263978723
N -1.8289555205 1.2119364382 3.2089622211
C -3.1697234942 1.4193585808 3.811987265
C -2.89222302 2.3159797553 5.0288141597
C -1.6170222971 3.0744933947 4.6300107782
C -0.8012628461 1.9949054906 3.9057222985
N -7.2458542916 -0.273071717 0.4518963371
P -8.6517091706 -0.9023845944 0.6364018113
N -9.6021006277 -0.5702091612 -0.7157291063
C -11.068192607 -0.4929071329 -0.7027872212
C -11.4131715664 -0.1106246287 -2.1502680218
C -10.3154463155 -0.8173163774 -2.9639224856
C -9.0630320482 -0.6579938811 -2.0841030775
N -8.6482617301 -2.5821585085 0.777372625
C -7.5118140252 -3.2753198934 1.4197420461
C -7.7444490374 -4.7623221414 1.1031966205
C -8.5720787093 -4.7297291557 -0.1909795543
C -9.4932728548 -3.5219721427 0.0322174633
N -9.5786007029 -0.3785177509 1.935452452
C -10.520224014 -1.1529012572 2.7364923481
C -10.4234982791 -0.4706868403 4.1101292913
C -10.1910753931 1.0230565725 3.7707022435
C -9.6741203815 1.0371345492 2.3045664154
N -4.7227285318 3.3039510404 -0.053623407
C -4.546265003 4.112823717 -1.2760447947
C -3.0321062415 4.3634599253 -1.3376666931

C	-2.4514323088	3.1001919376	-0.6840455499
C	-3.4191189296	2.8326317046	0.4730467116
N	-5.964226298	4.5207375657	2.0674533423
C	-5.4187987159	5.8357241831	1.7008541888
C	-5.2994987631	6.5742859002	3.046474618
C	-5.0787652429	5.4314865056	4.0514536316
C	-6.0167076288	4.336929902	3.5262435521
H	1.342495866	-0.3710995188	2.9726322164
H	-3.1726505105	-4.2102902715	2.1101569228
H	-7.1272684144	1.0746194966	3.7116635406
H	-3.8603741952	-5.2723451223	4.2508211687
H	-7.4748832284	0.0453633327	5.9401029629
C	-5.5676271425	-3.8875648677	6.2388995128
C	-6.3505958706	-2.600869606	6.6686938136
H	-1.4416811307	-3.0864699291	6.2184314253
H	0.0588887295	-2.212279449	5.8372086241
H	-1.6225123651	-0.5287877126	5.3287366645
H	-2.7174747109	-1.7476545095	4.6370969651
H	0.2899127316	-4.175788744	4.3938080493
H	-1.4495224367	-4.1964337183	4.0662801342
H	-0.9283284036	-2.9722768913	2.1317715879
H	0.6501032901	-2.4677479608	2.7681472572
H	-0.2329882312	1.3853058434	4.6303598342
H	-2.6958720003	1.7010960448	5.9171350944
H	-3.7367064689	2.9726753883	5.2601694116
H	-0.0857323029	2.4165774316	3.1918139437
H	-1.8626982461	3.8832369414	3.9290909029
H	-1.0798689567	3.5104041493	5.4795901302
H	-3.8382301106	1.9029081753	3.0936946977
H	1.6221013188	1.197425853	2.1985300617
H	-3.6451777381	0.478640913	4.0969721711
H	2.0641708204	-1.563105219	0.95248444
H	3.2094421819	-0.2045867324	0.983295258
H	1.8203927029	1.0904045916	-0.5624429733
H	-0.5550217075	0.9209900891	-0.4561796782
H	1.6814643578	-0.5345992403	-1.2527990097
H	-0.5177308348	-0.8414936771	-0.3720263985
H	-2.5278950731	-0.6460231999	-1.4957933641
H	-3.2742477794	-1.4098902328	-2.9053323972
H	-1.9955472715	0.0789342042	-4.1964116975
H	-0.9081060018	0.4536681533	-2.8596939009
H	-0.2510327018	-1.355927523	-5.0135636275
H	0.6192166934	-1.2014702565	-3.4800703031
H	-1.6192657175	-3.1570337892	-4.1654919085
H	-0.1122436343	-3.5007244273	-3.2881749684
H	-5.1442734349	-3.8282685061	-0.7523504736
H	1.1930279708	-3.7611822245	-1.2980031469
H	0.2901420393	-3.0160913031	0.0303437229
H	1.5937347018	-4.4969998167	1.3654229591
H	1.8146726985	-5.6411469062	0.031806249
H	-2.0168955626	-6.0853879579	-0.1554045622

H -0.5687974225 -6.6811088732 -0.9833251096
H 0.1233450064 -6.7952185197 1.3691559258
H -0.7107403515 -5.2651071358 1.7278136787
H -2.1742198522 -5.6580735006 -3.0127164678
H -6.006171412 -6.3722078817 -1.3556792955
H -3.4615652219 -4.7372117755 -3.815614527
H -5.9529067329 -5.1230547322 -2.6102014134
H -4.5478059565 -6.9026331503 -3.5843029506
H -3.7671689385 -7.1936242609 -2.0164979184
H -4.3632424915 -5.2228048902 -0.0014005681
H -8.699123853 1.5180372438 2.1885545642
H -10.3902954883 1.5699775409 1.654361575
H -9.4655565053 1.478551076 4.452781749
H -11.121391526 1.5967001874 3.8542024497
H -11.3154618293 -0.633078786 4.7260784736
H -9.5567490625 -0.8734072445 4.6432859828
H -10.2333043387 -2.2075469348 2.7493421274
H -11.5491007308 -1.0816340919 2.3358202798
H -11.4163400624 0.2505848038 0.0231837863
H -11.3344644885 0.9758405335 -2.275588538
H -11.5355218412 -1.4560123489 -0.4353131209
H -7.4785550031 -3.0853054406 2.4977482469
H -6.5701524896 -2.9135803815 0.9962630219
H -12.4272264313 -0.4131947089 -2.4328052793
H -6.7999334114 -5.3073728253 1.0060183485
H -8.323631143 -5.2387610289 1.904994632
H -9.1245606271 -5.6570269493 -0.3807917863
H -7.919007566 -4.5368265644 -1.0511405958
H -9.8459445923 -3.0823822167 -0.9045097317
H -10.3828727717 -3.8156059849 0.6173741302
H -8.3651801377 -1.4980535888 -2.1874498305
H -10.5643130051 -1.8806562597 -3.0789571376
H -10.180034001 -0.3964614762 -3.9660927014
H -8.5061544913 0.25505501 -2.3312030227
H -2.6793147777 4.5334686136 -2.3611940022
H -2.4791204729 2.2594720584 -1.3895052139
H -2.7720375675 5.2471199595 -0.7389579544
H -5.13549103 5.0345078181 -1.2402882935
H -4.8746308927 3.5434605403 -2.1582648736
H -4.433961545 5.7282173888 1.2205046897
H -4.4958528669 7.3185731257 3.0436665754
H -6.2391485756 7.0941438414 3.2743199578
H -4.0387256113 5.0838515369 4.0031462295
H -5.2949418876 5.7161273831 5.0870759949
H -5.7139467307 3.3233127938 3.796176401
H -7.0417053765 4.4899749933 3.9021792874
H -3.1245479206 3.4156345911 1.3616626087
H -1.4188075391 3.2193168737 -0.3396762796
H -8.7988141166 3.8123853091 1.3939482772
H -7.163621342 1.9760929999 -1.1837611254
H -10.5820854825 4.2002367865 -0.1944210797

H -9.4684956353 2.0383728724 -0.6485094439
H -9.5717144276 2.7573917401 -2.2650158975
H -9.4953266416 5.0382872896 -1.3207811952
H -7.2551524122 3.4345631891 -2.199689589
H -8.3569195883 5.4229285994 0.7949975974
H -6.0735764865 6.3601049892 0.9911913471
H -3.4553340687 1.7769488395 0.7497895882
H -5.876189388 -2.1189568077 7.5337989401
H -7.3815974392 -2.829798081 6.9661472083
H -4.7613902226 -4.1295434206 6.942672214
H -6.2297186532 -4.7629829457 6.2100939413

6bbH^+
C 0.1339994417 0.0590511948 0.0806259706
N 0.1212309939 -0.0460549388 1.5590798276
C 1.4895251379 -0.0053643518 2.0970526457
C 2.3137716768 -0.601664748 0.9515623401
C 1.6337101891 0.0041884061 -0.2891247716
P -1.2771310271 -0.2661405347 2.430207396
N -0.8417449597 -1.3957266625 3.5878799549
C -1.451360507 -1.457197816 4.9317233458
C -0.6501578477 -2.5729256131 5.6083985761
C -0.4498644162 -3.587938854 4.4690472951
C -0.2828741061 -2.7176658275 3.2056049386
N -2.4651738446 -0.5936764431 1.435826449
P -3.3515520781 -1.8934444853 1.1014592252
C -4.1993847074 -2.5087734448 2.5885503194
C -5.123819616 -1.7131225208 3.3706087771
C -5.4355461718 -2.264435499 4.6464961932
C -4.9870500862 -3.5253346239 5.1106668804
C -4.1839461783 -4.2934054809 4.2997457031
C -3.787637486 -3.7552660303 3.0501340973
C -6.260898602 -1.6324707468 5.6058578308
C -6.8092563279 -0.4087825835 5.295269951
C -6.5657631324 0.1327438411 4.0093324366
C -5.7885683174 -0.4828055868 3.0329654898
P -5.7206932596 0.276268364 1.2968084775
N -6.2544530065 1.8363790791 1.6981711394
P -5.9949287993 3.1280388406 0.8331306483
N -7.1710760342 3.5787866378 -0.2889768707
C -7.4423358372 2.6584839181 -1.4261264553
C -8.9732860862 2.5676115908 -1.4973967525
C -9.430711514 3.9170154565 -0.9251400727
C -8.4405871045 4.1503002135 0.2224893098
N -2.5400468662 -3.150193116 0.4513252807
P -2.2468574546 -3.4082949745 -1.0906230275
N -3.5826256092 -4.0724019994 -1.8224339502
C -3.7136175757 -4.1586555088 -3.2941037059
C -5.0487308851 -4.8957127471 -3.502344231
C -5.8468249887 -4.5561181077 -2.2334277228
C -4.7798263237 -4.6017678488 -1.134474094

N -1.803765879 -2.0672320105 -1.9834857576
C -0.7790048583 -2.0284274635 -3.0512304466
C -0.8419463827 -0.5747826647 -3.5365233181
C -2.3321166477 -0.2284573644 -3.4038292742
C -2.7364298249 -0.9131908276 -2.0897303881
N -0.9416073763 -4.4450511751 -1.2991926712
C 0.3172262326 -4.0759883165 -0.5917919677
C 0.6026471296 -5.2210818678 0.4218972214
C -0.6383525819 -6.1339569431 0.3507795907
C -1.1538792309 -5.8931974467 -1.0743127761
N -1.6705635416 1.128954005 3.2436927066
C -3.0198470675 1.3562443979 3.8283756342
C -2.7702052842 2.4111591185 4.9167523761
C -1.5345780446 3.1685946131 4.4069400671
C -0.6700967012 2.045005791 3.8206082268
N -6.9485236244 -0.5667048912 0.5027411025
P -8.3931378744 -1.1466837174 0.6655305768
N -9.2633658602 -0.9076867081 -0.7494595114
C -10.7337639479 -0.8479238699 -0.8170662758
C -11.0081188349 -0.5868076905 -2.305330043
C -9.8635551026 -1.3392911214 -3.0041432663
C -8.6583477202 -1.0872146837 -2.0825877612
N -8.4076009035 -2.8083854986 0.9102813445
C -7.5831165947 -3.4421500193 1.9572527881
C -8.1639240124 -4.8595451398 2.0828587718
C -8.7498754981 -5.1261501868 0.6872853373
C -9.349082904 -3.7649538644 0.3087989604
N -9.3427363545 -0.5232164817 1.8988141023
C -10.3519162048 -1.2386733859 2.681184001
C -10.3682726281 -0.4460871042 3.996790544
C -10.1097702553 1.0172520577 3.5580430532
C -9.4980167038 0.9189748732 2.1334382382
N -4.6159606408 3.1116399101 -0.1301723332
C -4.4167706071 3.8879429777 -1.3747695348
C -2.9308637542 4.2703096275 -1.3437723391
C -2.2927091279 3.0840031898 -0.6066737101
C -3.3161739239 2.7627119824 0.4892310817
N -5.936148749 4.4219473355 1.8909611651
C -5.4585691465 5.7486706448 1.4650599186
C -5.4401496797 6.5671976517 2.7677832079
C -5.1810836941 5.5013195788 3.8452855274
C -6.02488964 4.3163431844 3.3584852518
H 1.5552994651 -0.5623084939 3.034672402
H -3.1120953974 -4.3305942681 2.4239699913
H -7.0185322218 1.0854538081 3.7511117087
H -3.8346003069 -5.2779772882 4.6016098847
H -7.4392002929 0.1307752591 5.9999956535
C -5.5522741781 -3.7896743665 6.4921420608
C -6.3616404922 -2.4983968366 6.8465318022
H -1.1745463453 -2.9976298754 6.4702273617
H 0.3154114285 -2.1825207779 5.9527020892

H	-1.3582823976	-0.4909172823	5.4359554867
H	-2.5170326988	-1.7239742436	4.883312143
H	0.4082745247	-4.2479600486	4.6302147199
H	-1.3423343118	-4.2140002247	4.3738810997
H	-0.8068857776	-3.1442349058	2.3437074918
H	0.7734215894	-2.6044261123	2.9300844998
H	-0.0748920587	1.5584651854	4.6107036358
H	-2.5412765678	1.924726666	5.8736686111
H	-3.6411052311	3.0549817979	5.0704042688
H	0.0186332445	2.3988130329	3.0471442638
H	-1.8219718414	3.8675509235	3.6109591945
H	-1.0144536345	3.7334306416	5.1867460475
H	-3.7066978479	1.7193857371	3.0597924893
H	1.8077545925	1.0292459012	2.2989941474
H	-3.4506630661	0.4389814865	4.2362721691
H	2.2087058833	-1.6941404023	0.9536945616
H	3.3788018786	-0.3589504227	1.0205740351
H	2.0198559542	1.015007831	-0.4663944824
H	-0.3341526726	0.9893724256	-0.2590860159
H	1.8067961501	-0.5783097921	-1.1998947475
H	-0.4266787078	-0.7709884103	-0.3533643569
H	-2.6097889361	-0.2425659866	-1.2322149798
H	-3.7813182631	-1.2459474686	-2.1022537535
H	-2.893261272	-0.6557798564	-4.2440693477
H	-2.5290133041	0.8478920835	-3.3825314243
H	-0.4576391245	-0.4623930404	-4.5549213698
H	-0.2455375845	0.0656208592	-2.8749332195
H	-1.0180220816	-2.7291671194	-3.8634683373
H	0.2055593724	-2.294777491	-2.6589178906
H	-5.037202138	-3.9937132074	-0.265431314
H	1.1287912557	-3.9839031732	-1.3228730295
H	0.2006350623	-3.1085701067	-0.0948620198
H	0.77545686	-4.8348630569	1.4308557953
H	1.5015011215	-5.771807414	0.1235714577
H	-2.2025155988	-6.1609779264	-1.2231320981
H	-0.5560753401	-6.4508696104	-1.8080897363
H	-0.4057989814	-7.1853906964	0.5486944313
H	-1.3973645862	-5.8033869523	1.0686149495
H	-2.8666877634	-4.6933943118	-3.7408294916
H	-6.6711308863	-5.2505733482	-2.0454606904
H	-3.7393228142	-3.1500231491	-3.728466726
H	-6.2624720586	-3.5431510973	-2.2971197039
H	-5.548689872	-4.591317902	-4.4268490326
H	-4.875474393	-5.9775704849	-3.5559417848
H	-4.6131992273	-5.6333304501	-0.7877278269
H	-8.5309470038	1.4178052628	2.0378008621
H	-10.1841126871	1.3658214102	1.3945080496
H	-9.4296941718	1.525979845	4.2491826539
H	-11.0402877942	1.5945322239	3.5363682629
H	-11.3072413655	-0.5660805638	4.5473523208
H	-9.5497658216	-0.7992948574	4.6325354207

H -10.0726924069 -2.2869568377 2.8107908858
H -11.34143932 -1.2069601406 2.1914870213
H -11.1224802826 -0.0531571505 -0.1711710903
H -10.9378958153 0.486679124 -2.5149392661
H -11.2026674623 -1.7920170554 -0.4990447395
H -7.632295055 -2.8891200465 2.9019566802
H -6.5315759208 -3.4639318237 1.6498086851
H -12.0020369692 -0.927783224 -2.6115706099
H -7.4080543604 -5.5906567526 2.387178206
H -8.9660442249 -4.8756627519 2.8320720067
H -9.4917519819 -5.9309614687 0.6710068644
H -7.9475041286 -5.3866162888 -0.014411587
H -9.4207248897 -3.6172267488 -0.7735518515
H -10.3642331264 -3.6653783442 0.7279942379
H -7.9449086921 -1.9200313398 -2.0873208912
H -10.0916754287 -2.4124234221 -3.0416047978
H -9.6850752531 -0.9996177523 -4.0295843619
H -8.1069710246 -0.1832550515 -2.3724156929
H -2.5213115537 4.436936468 -2.3457391135
H -2.1953681048 2.227135593 -1.2863508684
H -2.7889260777 5.1917525991 -0.764381927
H -5.0845994435 4.7523734368 -1.4207479807
H -4.631151189 3.2554513998 -2.2486789616
H -4.450131034 5.6761810064 1.0300988181
H -4.6894143845 7.3635678865 2.7486473385
H -6.4200032015 7.032553644 2.9322885123
H -4.1181657787 5.2244375204 3.8532765648
H -5.4541918135 5.8295479017 4.8534849624
H -5.6606508598 3.3438446335 3.6965836353
H -7.0691972523 4.4162749848 3.6927550718
H -3.1300736007 3.3837780348 1.3810351326
H -1.301181214 3.30394679 -0.1971106116
H -8.7799432133 3.6320624383 1.1307479913
H -6.9833015607 1.6770189384 -1.2630321429
H -10.469758864 3.914008749 -0.5792611475
H -9.3190874604 1.7432888189 -0.8653316642
H -9.3309988687 2.3867490716 -2.5165639299
H -9.327157199 4.7069381482 -1.6798200739
H -7.0273977753 3.085108449 -2.3489925376
H -8.3122404277 5.2094923329 0.4681957857
H -6.1151216811 6.1865980789 0.7022900221
H -3.2935587992 1.7140044788 0.7912127911
H -5.9370423141 -1.9879282051 7.719836716
H -7.404393978 -2.7273838765 7.0962665373
H -4.7562131112 -3.9886295107 7.2194628879
H -6.1956109178 -4.6781596782 6.4848147499
H -4.3395194141 -1.4621842597 0.2154169923

7aa
C -0.0534913355 0.0539656732 0.0492264887
C -0.1386369803 0.0627339265 1.5142022498

C	1.1976749593	0.0806237172	2.0031595566
C	2.0916153416	0.083868813	0.9013839895
C	1.2662062026	0.0572684965	-0.3126767055
C	1.5747692685	0.10421571	3.3610310565
C	2.9889790887	0.2569467162	3.6003136092
C	3.8647809139	0.2273195251	2.5150326946
C	3.4496189177	0.1185124588	1.1658133412
C	-1.1608370977	0.0574925574	2.4407527757
C	-0.8217139043	0.0005348299	3.8194358506
C	0.4820203968	-0.019723133	4.3100309186
P	3.6916178694	0.8616169869	5.2420435343
N	5.1013496832	-0.1038567296	5.2974541685
P	5.873487143	-0.3927721672	6.6324868754
N	5.0940907776	-0.8959326873	8.0570572987
C	4.551420569	-2.2626159947	8.0973265523
P	0.7650353104	-0.2362049355	6.1624281242
N	-0.8221230608	-0.691678903	6.6702365842
P	-1.7771490534	0.1095237131	7.6127102218
N	-2.7523500078	1.3537616111	6.9687447897
C	-3.9057940621	0.9950220216	6.1398850399
N	1.6457121054	-1.685451611	6.150553304
P	1.2479782575	-3.1549445945	5.7585480662
N	0.4919057049	-3.9592683092	7.0514726197
C	-0.192798637	-5.2430241416	6.9706133541
N	0.1898376362	-3.5513815024	4.4932440031
C	-1.2558739446	-3.3479371416	4.5950148633
N	2.6927050607	-3.9591249818	5.3910554563
C	3.8337256383	-3.2591522379	4.80317289
C	0.6712545084	-3.470921948	3.1122525776
N	4.3312436746	2.3484660837	4.6528819718
P	3.6642572797	3.7593615899	4.7320060407
N	3.4530124958	4.3311310921	6.3225276941
C	2.7187600485	5.5378656794	6.6811329181
N	4.6235632982	4.8145327413	3.8060914808
C	6.0196656436	4.473417525	3.5468173916
N	2.1206357964	4.0670340615	4.0908955475
C	1.9596117814	3.957731671	2.6340621516
C	4.3739832084	6.2489167107	3.8883141407
N	6.9212671054	-1.6871125026	6.3234206552
C	7.2641231634	-2.0557239997	4.9529514532
N	6.7012976654	0.9830546182	7.2139691371
C	7.3038537759	1.868471824	6.214226989
C	7.9226358213	-2.0674676314	7.3112237419
C	7.4100710768	1.0065743221	8.491032684
C	4.1780324984	0.0567666349	8.7054779046
N	-2.9458601198	-0.9686648228	8.2139927066
C	-2.5889254689	-2.3768812329	8.3739875102
N	-1.006280072	0.9591460577	8.8733326519
C	0.1214923328	0.3262487968	9.5544939519
C	-3.8875437122	-0.495192819	9.2249233493
C	-1.6585704494	1.9761294546	9.6928618391

C	-2.0231612687	2.5044819689	6.4174481103
C	2.7018010555	-5.3933794399	5.1410538909
C	0.9098718324	-3.6224525535	8.4080845127
C	0.9516824269	3.5366965703	4.8050694904
C	4.3162382415	3.8410234454	7.3906588564
H	4.924844055	0.3458058743	2.725744799
H	-1.6245987625	-0.1044181317	4.5413431739
H	6.1117824853	3.3930912076	3.4339259943
H	-1.5533692478	-3.2012007933	5.631511076
H	2.8161146187	4.4049399103	2.1267152083
H	3.7683627465	-2.190708623	4.9908518951
H	6.3399721735	4.9636216042	2.6176485177
H	-1.5684130576	-2.460197738	4.0341656743
H	1.8603975809	2.9116742178	2.3092616677
H	4.7610940611	-3.6308040929	5.2576261628
H	1.0513689164	4.4996202543	2.3405745795
H	3.8964190774	-3.4232589421	3.7144722422
H	3.3001950496	6.4448344505	3.9355689559
H	1.7476285238	-3.6387922039	3.0632748488
H	4.8578527115	6.714322113	4.7645208643
H	0.1748836157	-4.2470085766	2.5133678389
H	4.7676781958	6.7355527748	2.9866629415
H	0.4584965905	-2.4930258032	2.6632316618
H	0.1072585848	4.229272075	4.6813267355
H	2.5703175599	-5.6331525833	4.0720182863
H	0.6552964016	2.5562981057	4.4159000508
H	3.6660033867	-5.8095157776	5.4629267171
H	1.1628059527	3.4215734183	5.8692499209
H	1.9163761175	-5.8998307964	5.7017779042
H	2.0660185661	5.8492560261	5.8648058556
H	-0.4105298843	-5.5027186285	5.9341224718
H	3.3981307552	6.3705767499	6.9261242665
H	-1.1443093851	-5.1915175763	7.5211730503
H	2.092751336	5.3431438275	7.5652194299
H	0.4068548884	-6.0527856974	7.4190234269
H	3.7113135226	3.5963606599	8.2760898191
H	1.6636921485	-4.331924728	8.7885851794
H	5.0612954646	4.5998862091	7.6845252487
H	0.0413457746	-3.651442416	9.0793351554
H	3.1889438162	0.0569497934	8.228460159
H	-1.6940453079	2.3202506876	5.3844566718
H	4.5816957745	1.0691834421	8.6569587718
H	-1.1400980316	2.7232394715	7.0207210534
H	4.0702277399	-0.2259135116	9.7620580425
H	-2.6801255157	3.3837949713	6.4242446611
H	3.5975181662	-2.3316543286	7.5630572787
H	-3.6197195384	0.7852368051	5.0971622246
H	4.3965072141	-2.5398257598	9.1488177068
H	-4.6130491364	1.8347813245	6.1350085917
H	5.2590204758	-2.9649829871	7.6544889724
H	-4.4068519808	0.1138490389	6.5425057545

H 7.5579852311 -1.8680170947 8.3212938985
H -4.1741960147 0.5404647747 9.028993533
H 8.1237070548 -3.1444574847 7.2300374024
H -4.7940711587 -1.1137541997 9.1930374842
H 8.877044298 -1.5334819387 7.1678291079
H -3.4705944348 -0.5560472225 10.2444017898
H 7.3928822262 -3.1457887681 4.8926181805
H -3.4745334718 -3.0019605486 8.1951234914
H 8.4710264764 0.7265070472 8.3826132005
H -2.0187112135 1.5623148359 10.649156556
H 8.2031287345 -1.5799198629 4.6238703601
H -2.2215282421 -2.5888157399 9.3926433492
H 7.3729572283 2.0238121053 8.905666995
H -0.9404114058 2.7766318742 9.922284972
H 6.4608305411 -1.7494129888 4.2829738509
H -1.8069231265 -2.638818221 7.6612536003
H 8.3254853121 1.5481831814 5.9460473733
H -0.1972176506 -0.1698872453 10.4872537628
H 7.3641488412 2.8847153718 6.6264542497
H 0.8733522287 1.0857049747 9.8067288392
H 6.6791368581 1.9003752548 5.3218951603
H 0.5970771011 -0.4101927225 8.908275366
H 6.9395484616 0.3262963651 9.2024163308
H -2.5055391145 2.4119924019 9.162148243
H 4.1909335197 0.1074327936 0.3682958082
H -2.208627083 0.0514847026 2.1432750801
H 4.8414740266 2.9400531321 7.0760408934
H 1.3308376194 -2.6177275987 8.4255227126
H 6.6896769646 4.8062219539 4.3587474808
H -1.7783827144 -4.2259369607 4.186240444
H -0.9037546067 0.0471522184 -0.6244149746
H 1.6520331955 0.0444082445 -1.32658719

7aaH^+
C -0.1988835564 -0.1846849731 0.1264350099
C -0.2503500493 -0.1936450059 1.594687359
C 1.0937916179 -0.0890191088 2.0584547175
C 1.9566344427 -0.0011735325 0.9378236819
C 1.1055819851 -0.0777161244 -0.2601612873
C 1.5018363966 -0.0374193667 3.4042065191
C 2.9103913711 0.1962028821 3.6183483864
C 3.7531304746 0.2539555982 2.5112019304
C 3.3103479375 0.14666367 1.167324246
C -1.2583700691 -0.2534991558 2.531835288
C -0.8918341737 -0.2723724342 3.9037457071
C 0.42289911 -0.1953896316 4.3613376675
P 3.648199522 0.6765115748 5.2886168304
N 5.1362833076 -0.0989418288 5.1137265846
P 6.1557203904 -0.3558500352 6.2809647246
N 5.6288336768 -0.5908905547 7.8774686045
C 4.8350363296 -1.795388726 8.1577815954

P	0.6582161387	-0.5663911036	6.139244227
N	-0.7970661458	-0.6918935514	6.8572946169
P	-1.6541415293	0.3688010378	7.6616332751
N	-2.4471308227	1.5802042873	6.8102107351
C	-3.728543912	1.3252905698	6.1432723399
N	1.5814853286	-1.8722027363	6.3123909878
P	1.2766421213	-3.4039698028	6.0070470641
N	0.5622091224	-4.1252042712	7.3431314293
C	-0.0947262247	-5.4329455739	7.3360616512
N	0.2203409695	-3.8319537138	4.7722381776
C	-1.2304347705	-3.6497540212	4.8964953891
N	2.7566723388	-4.0983750136	5.661521208
C	3.8495695073	-3.3587631546	5.0231702457
C	0.6851088453	-3.7987213351	3.3799268071
N	3.9973507765	2.3095570238	4.9870527814
P	3.1867932682	3.6389818644	4.9120954054
N	1.9063060973	3.7481730436	6.0632519008
C	1.1673326659	5.0136270617	6.156912306
N	4.2761870911	4.9128460415	5.0907104178
C	5.5137357486	4.7337173956	5.8451728326
N	2.3703630618	4.0667826594	3.4896028749
C	3.2000231347	4.2312914202	2.2880371855
C	3.9435193673	6.3086136954	4.822122717
N	6.9390893037	-1.8214051473	5.9490374299
C	7.0993895983	-2.2438070878	4.5560924613
N	7.2241217746	0.9493617633	6.466975084
C	7.562990175	1.7232544752	5.2665829081
C	7.9925082593	-2.3234945379	6.8306441618
C	8.3105655155	0.9115254302	7.4483456185
C	5.1555616559	0.5650858393	8.646452081
N	-2.8830703775	-0.4820874791	8.4172343956
C	-2.7768111383	-1.9238164409	8.6565350302
N	-0.6859040435	1.2668453019	8.7220291805
C	0.307179852	0.5453416859	9.5280579924
C	-3.752434365	0.2157464867	9.3689415007
C	-1.1831277449	2.4680537971	9.4039649243
C	-1.6207854527	2.5600602481	6.0881903053
C	2.8702008329	-5.5497967996	5.5272002434
C	0.9407729213	-3.6776394305	8.6845257522
C	1.0713952438	3.4630939531	3.170784251
C	2.234052299	3.2305466186	7.3962910029
H	4.8108398585	0.4194697908	2.6994348982
H	-1.6793427252	-0.3959284775	4.6392238881
H	5.6931295502	3.6723281849	6.0114699117
H	-1.5102572049	-3.4860886073	5.9371032122
H	4.154004534	4.6973912653	2.5410467618
H	3.7248443461	-2.2878419516	5.1573991977
H	6.3592157274	5.1514449039	5.2805761349
H	-1.5666275695	-2.7858494503	4.3123185546
H	3.399728708	3.2650759963	1.8017289027
H	4.7976429977	-3.6492564898	5.4870068191

H	2.6717351706	4.8795012162	1.5783039721
H	3.9093082535	-3.5783414507	3.9462998454
H	3.0273410241	6.3802722267	4.2348708966
H	1.7496184413	-4.0273894698	3.3211550521
H	3.8131036301	6.8798230569	5.7539800814
H	0.1399539004	-4.5574974702	2.8061284972
H	4.7564491795	6.7786178749	4.2506401048
H	0.5141030395	-2.817202012	2.9185555516
H	0.4800605267	4.1768409014	2.5824883466
H	2.768544847	-5.8756074891	4.4805320238
H	1.1860428539	2.5432349258	2.5835617939
H	3.85579606	-5.865466305	5.8892084366
H	0.5258098601	3.2203315978	4.0825897461
H	2.114861194	-6.0635288286	6.1224482707
H	0.9134724455	5.3872933741	5.1636023308
H	-0.2925411679	-5.7640659409	6.3162397159
H	1.7389889078	5.7911784453	6.6882336504
H	-1.0515727027	-5.3655089858	7.8711668773
H	0.2358114633	4.846193793	6.7115174313
H	0.5220702562	-6.1908486414	7.8410964239
H	1.3024353316	3.0512087341	7.9451445277
H	1.6477879567	-4.3780355376	9.1529447892
H	2.8482075829	3.9366715615	7.9810631349
H	0.0440338453	-3.6149597202	9.314490773
H	4.0764058184	0.7257787333	8.5016826335
H	-1.5059625971	2.2743042804	5.0343963423
H	5.6820520568	1.4704094422	8.3416554231
H	-0.6247769649	2.6322497442	6.5266755788
H	5.3420917379	0.3883543011	9.713970219
H	-2.0976837057	3.5465338469	6.13510577
H	3.7917441902	-1.6841477563	7.8283915314
H	-3.5837757156	0.9719954962	5.1115904918
H	4.8533508347	-1.9815256013	9.2389838195
H	-4.3008073475	2.2597860627	6.1073350464
H	5.2661557445	-2.660002874	7.6517273687
H	-4.3071813451	0.5795203034	6.6883185583
H	7.7997329836	-2.0279904619	7.8636670765
H	-3.8473419709	1.2709468442	9.1038302246
H	8.0040168489	-3.4202949075	6.7855351308
H	-4.7522777408	-0.2333165389	9.3417282517
H	8.9910004136	-1.9633439041	6.5380130284
H	-3.3701845614	0.1432631934	10.3981434835
H	7.1284558176	-3.3406799922	4.5156709509
H	-3.7688623393	-2.3797728906	8.551258643
H	9.2308982452	0.4717370434	7.0338105403
H	-1.6499915887	2.2241958093	10.3697313426
H	8.0343979155	-1.8623581829	4.1153393819
H	-2.4045357205	-2.1346030589	9.6708731542
H	8.5408084048	1.9359286326	7.7678565235
H	-0.3414868585	3.1443196328	9.5959806469
H	6.2563739836	-1.8863605705	3.9643683908

H -2.0960582382 -2.3689816536 7.9314221079
H 8.3697372634 1.2486904068 4.6842050562
H -0.1026196242 0.2571238966 10.508262303
H 7.9092824428 2.7166627049 5.5756369806
H 1.1767786303 1.1920193022 9.6942970696
H 6.6796102688 1.8389854733 4.6385695123
H 0.6447106569 -0.3569244593 9.0172190412
H 8.0135252034 0.3386038841 8.3286342772
H -1.9119645383 2.989324252 8.7833060742
H 4.0283826342 0.2136017995 0.3526531148
H -2.3078213489 -0.3139908677 2.2532195236
H 2.7690558523 2.2840000447 7.3143928845
H 1.4023804483 -2.6915726784 8.6347326244
H 5.4630037279 5.2503725926 6.8166862434
H -1.744517026 -4.5454059102 4.5240230588
H -1.0616352183 -0.2494103146 -0.5263051604
H 1.4699231439 -0.0498380892 -1.2810230453
H 1.3722129252 0.4920182726 6.7018102692

7bb
C 0.1114855956 0.1069387272 0.0736992869
C 0.0108385669 0.0571765777 1.5378074713
C 1.3384901522 0.0282997053 2.0401585818
C 2.2457769836 0.0736659349 0.945406574
C 1.4340698683 0.1260532001 -0.2766401277
C 3.5958489441 0.0732543119 1.2297941194
C 4.0007658782 -0.045237775 2.586045241
C 3.1195222709 -0.136061246 3.6608608958
C 1.6972576635 -0.0190598281 3.4018492732
C -1.0232037801 0.0397193133 2.4543404433
C -0.700243971 0.0582870835 3.8342244897
C 0.599095533 0.0702132114 4.3380761139
P 3.7994364981 -0.4325084803 5.393476357
N 5.4604380397 -0.6650982214 4.987151763
P 6.7247905329 -0.1702266635 5.7586974896
N 8.1672217593 -0.790421202 5.1146169897
C 8.2759788656 -2.2771096837 5.099744306
C 8.8092352608 -2.6420779417 3.6971852917
C 8.4163982014 -1.4361246639 2.8290365286
C 8.603069831 -0.2653020131 3.8005339957
P 0.8551084913 0.2948924972 6.2103660975
N 0.8346124876 1.9797454542 6.3616080663
P 0.3354241428 3.337765435 5.7998829544
N -1.1623816006 3.3645530742 5.0421909756
C -1.5825980466 4.1918467173 3.9159466128
C -2.6258900571 3.3009784702 3.2235391144
C -3.3093819285 2.5452500903 4.3910973239
C -2.3261366471 2.6606945629 5.589662298
N 3.204668743 -1.9807000881 5.6992035582
P 3.2681623094 -3.4197098869 5.0934685816
N 1.7327138488 -4.0982849824 5.1817917852

C	0.5312253356	-3.326264931	4.7758323487
C	-0.4760788926	-4.4067936888	4.352028019
C	-0.0475467292	-5.6351962271	5.1692044122
C	1.4838642546	-5.5440848779	5.126777601
N	4.3090970375	-4.3998010696	5.9795405158
C	5.0674071971	-3.8596955275	7.1297360474
C	6.1040329713	-4.9551651329	7.4490407578
C	6.2506670251	-5.7232550654	6.1245113639
C	4.8121254642	-5.7229097348	5.5924521941
N	3.7715870619	-3.7320764694	3.5045025648
C	2.8681550009	-3.5847527492	2.3491368649
C	3.7735985375	-3.9402049776	1.1658794193
C	5.1189344026	-3.3070679102	1.5651342365
C	5.1699724611	-3.4450108759	3.1016417411
N	-0.7030217667	-0.2505829759	6.6900127336
P	-1.0514458148	-0.7898192374	8.1216118845
N	-2.3465768693	-1.8479551117	7.9564312878
C	-2.7314256753	-2.7613049748	9.0423435791
C	-3.790635146	-3.6744253916	8.3986695832
C	-3.3777858117	-3.6941677211	6.9175119429
C	-2.9426571294	-2.2438433322	6.6705669581
N	-1.619613046	0.3110643472	9.2814627165
C	-0.6969292203	1.4005163978	9.7007844416
C	-1.5283888797	2.6860882246	9.5853959507
C	-2.9624766572	2.1934204806	9.8245880608
C	-2.983540602	0.854496484	9.0769713028
N	0.1838680752	-1.5417074497	8.9710541138
C	0.3248270961	-1.6240836735	10.438698151
C	1.0727362065	-2.9467808464	10.664322992
C	1.9391919717	-3.0681730765	9.4014549938
C	1.0060509541	-2.5725289798	8.29245986
N	6.9094037512	1.5008607251	5.7175254662
C	8.1103280315	2.2274210961	6.1658113594
C	8.1310771096	3.4737237487	5.2695898639
C	6.6348994172	3.7681838889	5.0780758216
C	6.0130466622	2.3713446408	4.9204422346
N	6.8588218986	-0.7227588582	7.3533964229
C	8.0712871449	-0.6159456338	8.1739864807
C	7.724363003	-1.4945205304	9.3882096311
C	6.1872570915	-1.3312406276	9.5509091416
C	5.7233531787	-0.5743568299	8.2819749075
N	0.1899391887	4.4502975034	7.0575470194
C	-0.7328619309	5.5924333687	7.05637016
C	-0.5115183663	6.2238751526	8.4391680286
C	0.9831029102	5.9683214128	8.6984882793
C	1.2107833398	4.5629079687	8.1141841328
N	1.3830282799	4.0504264774	4.690682511
C	2.2117774584	3.2128417987	3.797707208
C	3.1940727835	4.2037876653	3.1511152466
C	3.2875067513	5.3414999919	4.1796165101
C	1.8457182001	5.4429812608	4.6975461861

H	4.7364147267	-5.8666547301	4.5112108794
H	5.0613382047	-0.1221551068	2.8089525927
H	-1.5106582695	0.0646873527	4.5561666322
H	4.3491038647	0.1250669644	0.4443897954
H	-2.0675104464	0.0378265003	2.1441268825
H	3.3850231602	-3.5565106293	0.2165270091
H	3.8698821905	-5.0307806667	1.0845515331
H	2.0112447236	-4.2588468289	2.4479914961
H	2.4888425235	-2.5589128015	2.2439043767
H	5.9769726874	-3.7837805689	1.0781811253
H	5.1221447109	-2.2485487816	1.2880133118
H	5.5413500197	-2.5342439877	3.5831442966
H	5.8182650997	-4.2783988797	3.4060445309
H	1.8744654287	-5.9993532194	4.1996309225
H	-0.3751005383	-4.6202420604	3.2798224297
H	-1.5112894727	-4.1011861868	4.5331399604
H	1.9593321632	-6.0508466063	5.9732360106
H	-0.3939896485	-5.5383355141	6.2066794345
H	-0.4250502054	-6.5829997502	4.7699952098
H	0.1614280137	-2.7310469599	5.6158860663
H	4.2334031891	-6.5280349342	6.0754816292
H	0.7435281298	-2.6269428361	3.9646277869
H	6.9011266859	-5.1711872861	5.4343731781
H	6.6603585051	-6.7319304677	6.2495236456
H	5.7249871352	-5.6296264553	8.2278854
H	4.4106190823	-3.6532388424	7.9815242495
H	7.0463003122	-4.5271599352	7.8082571135
H	5.5553777053	-2.9235550877	6.8531044596
H	4.8074325753	-0.9801842925	7.8481387926
H	5.5392289527	0.4887806115	8.5074418878
H	5.9263625577	-0.769607919	10.4542139392
H	5.6961759429	-2.3069846134	9.6213419969
H	8.283413121	-1.1995906128	10.2829012276
H	7.9749174881	-2.5379414219	9.1666028368
H	8.2502831062	0.4249751617	8.4964151126
H	8.9487724196	-0.9534728895	7.618453641
H	4.9851571008	2.3240981272	5.291448935
H	8.9449860296	-2.6213617532	5.8979823235
H	7.2929335439	-2.7263960354	5.2697264541
H	8.3871506333	-3.5861180638	3.3362971832
H	9.9012012107	-2.750485939	3.7177188634
H	8.0276109961	0.6219263572	3.5225488243
H	9.6623205764	0.0282683639	3.8575942961
H	9.0246800777	-1.3411847046	1.9224159997
H	7.3629470261	-1.5030568913	2.5367082534
H	9.0067559415	1.6067745821	6.0596634463
H	6.4224296056	4.4134298608	4.2197620284
H	8.0238471846	2.516142382	7.224586822
H	6.2340676205	4.2596354357	5.974050463
H	8.6876031506	4.3058808483	5.7140904762
H	8.5943420147	3.2309809034	4.3041453504

H	5.9953167272	2.0589833498	3.867059502
H	-2.0179945071	1.6945906651	5.9983834073
H	-2.7910380764	3.2379808763	6.4084223761
H	-3.4984963018	1.4983280383	4.131537342
H	-4.2754702655	2.9973477485	4.6433883088
H	-3.3344455587	3.8756491946	2.6163082495
H	-2.1063163521	2.5941456801	2.5694595992
H	-0.7270020735	4.4371704069	3.2816414777
H	-2.0410985579	5.1391440501	4.2569323674
H	-1.7666472017	5.2621586058	6.9048640848
H	-1.1188639617	5.7005980517	9.1872578773
H	-0.4997537393	6.3177290461	6.258240195
H	1.6040483765	2.691580944	3.0509672398
H	2.7330156019	2.4508533271	4.3853722221
H	-0.7818925678	7.2849855008	8.4629523705
H	4.1574177551	3.7324844035	2.9306946871
H	2.7857601197	4.5854818787	2.2064577077
H	3.6547929343	6.2838974457	3.7576981056
H	3.9521170622	5.0518018637	5.0032190329
H	1.7876231882	5.8761195806	5.6996758297
H	1.2360762527	6.0735221887	4.0265181349
H	2.2225309682	4.4336157395	7.7101739198
H	1.5861546027	6.7066081153	8.1534743651
H	1.2562282816	6.031253516	9.7573440653
H	1.0628025377	3.7791007278	8.8681911335
H	1.6483015687	-2.9458446152	11.5968477068
H	2.8111540338	-2.4049307363	9.4726720865
H	0.3583869904	-3.7802018955	10.7085689276
H	-0.646499194	-1.586570649	10.9418620349
H	0.9230734236	-0.7788274384	10.8098707471
H	-1.8633099021	-3.3449432772	9.3858268963
H	-3.8205434191	-4.6674899264	8.8598219296
H	-4.7868667233	-3.2250481686	8.5018034963
H	-2.5230896307	-4.3679010284	6.7743986522
H	-4.1816521452	-4.0168587297	6.2468356983
H	-2.2200295774	-2.1278037902	5.8604459393
H	-3.8137509519	-1.6112777811	6.4335658308
H	0.3721800687	-3.3975529618	7.9266865486
H	2.3012176984	-4.0843137804	9.213177591
H	-3.1960114057	1.0203270577	8.0105433406
H	0.1947182928	1.4359615394	9.0653129808
H	-3.7287248427	2.8843259531	9.4553773487
H	-1.425242532	3.0970191249	8.5758715555
H	-1.2087167106	3.4540699018	10.2986316184
H	-3.1383965288	2.0320369619	10.8964306871
H	-0.3761006565	1.2284060852	10.7375061653
H	-3.7353657426	0.1580522853	9.4644410702
H	-3.1216091873	-2.2126293798	9.910542292
H	1.5532163423	-2.1560441074	7.4443702303
H	-0.7323773899	0.1275808572	-0.6079288798
H	1.829866109	0.1721397506	-1.2857581094

7bbH^{+}

C -0.0808879109 -0.0566872673 -0.0407673222
C -0.12237255 -0.0925516583 1.4288980505
C 1.2237115637 -0.1226419123 1.8747379655
C 2.0849751301 -0.0914765356 0.7394604003
C 1.2221580676 -0.0484627396 -0.4491266978
C 3.4466327201 -0.0860009151 0.9545260045
C 3.9121625988 -0.1780061129 2.2924840451
C 3.0680185677 -0.2398852967 3.3997462803
C 1.6332596948 -0.1524268533 3.2188302082
C -1.1191727988 -0.0988810332 2.3837035181
C -0.7420767996 -0.0786179844 3.7524315262
C 0.5741404974 -0.0589996187 4.2028768704
P 3.8659811147 -0.6539869521 4.9873204256
N 5.4696050228 -0.4623214404 4.7775149822
P 6.5523418461 -0.0546822551 5.8692429
N 8.0889750103 -0.5737444898 5.433448913
C 8.2294630312 -2.0213502896 5.1038820154
C 8.7009106444 -2.0885850484 3.6237238416
C 8.6005667379 -0.6388381851 3.1098791394
C 8.8130551639 0.1844377453 4.3866846955
P 0.9276459932 0.1586260225 6.0491499031
N 1.1772438134 1.8233313571 6.153029219
P 0.6506909655 3.2117202433 5.6525954498
N -0.8857351414 3.2628354383 4.9855839421
C -1.3433608712 4.1274400926 3.8960128507
C -2.4956393696 3.3108951589 3.2928028392
C -3.1351092979 2.6037667312 4.5138305328
C -2.0572478588 2.6539031204 5.6315238412
N 3.3937687305 -2.0997640558 5.5054464422
P 3.4030111452 -3.5725113842 4.9208850956
N 1.8701606538 -4.1939562684 5.0857673885
C 0.6533522399 -3.4259143638 4.7075410371
C -0.4020339994 -4.5148399015 4.4622400381
C 0.0615100325 -5.6733663753 5.358392042
C 1.5862087778 -5.6369435715 5.1934127057
N 4.4867517094 -4.4906157688 5.7832891646
C 5.1106773522 -4.0201720842 7.0428481016
C 6.197527559 -5.0773317705 7.3435101724
C 6.4213597641 -5.7920196099 5.9988082787
C 5.0101925447 -5.81147239 5.4028410336
N 3.8578575936 -3.816389585 3.3290520437
C 2.9089893962 -3.8063491805 2.1961772812
C 3.8011968259 -4.1923474473 1.0131813404
C 5.1187695699 -3.4606396285 1.3250811733
C 5.2354978153 -3.518254675 2.8627978378
N -0.6081527295 -0.247524913 6.6390506015
P -0.9026046016 -0.7485624746 8.1051783486
N -2.2125001923 -1.7835031031 8.0164488442
C -2.6081816509 -2.6279483902 9.1571881853

C	-3.729819694	-3.5133584084	8.586661461
C	-3.3628742579	-3.6244247204	7.0976676292
C	-2.8657106553	-2.2117209582	6.7659564373
N	-1.3717350695	0.40106675	9.2464721278
C	-0.3915516289	1.4598292228	9.6119827216
C	-1.1945232033	2.7678188493	9.5686333867
C	-2.6258799776	2.3104536716	9.8841102313
C	-2.7310097112	0.9830692492	9.1236970654
N	0.3729231839	-1.5013780118	8.9028169079
C	0.5657491343	-1.5618670947	10.369472898
C	1.1770907287	-2.9488411025	10.6094702132
C	1.9868498593	-3.1868264332	9.3266074555
C	1.0784083654	-2.6174195978	8.2303341326
N	6.5777122684	1.5961267121	6.0519767098
C	7.2896950279	2.2513457643	7.1716421349
C	7.1242387106	3.7532635772	6.8822783129
C	5.8095387634	3.8200235456	6.089039998
C	5.8846318701	2.5796609557	5.1914326792
N	6.3463473892	-0.7269488871	7.3851723161
C	7.4240472382	-1.2026064243	8.2823269493
C	6.6606006664	-1.6330033942	9.5412376876
C	5.5088286185	-0.619852363	9.6135680467
C	5.0968372865	-0.4511602792	8.1435993897
N	0.6336898151	4.3054781817	6.925165442
C	-0.2255523802	5.5018706992	6.9746512599
C	0.1422264484	6.1453945481	8.319672132
C	1.6344446125	5.8052249495	8.468645468
C	1.7292585439	4.3752176242	7.9108725326
N	1.6528220224	3.9000495803	4.4950767672
C	2.0877121118	3.1468845813	3.302294926
C	2.6403086866	4.2269632788	2.3591844116
C	3.1062467604	5.33129837	3.3210082214
C	2.0122132082	5.3233400588	4.3970519601
H	4.9822947846	-5.9432078335	4.318615883
H	4.9826456248	-0.2228718133	2.4682412599
H	-1.5224871967	-0.0578513249	4.5069447265
H	4.1621263065	-0.0357798749	0.1369572029
H	-2.173744321	-0.0911090255	2.1143909292
H	3.3667769343	-3.8979973716	0.052867656
H	3.956210373	-5.2782348585	1.0018374319
H	2.0969682414	-4.5171957791	2.3742100024
H	2.4715037016	-2.8099869121	2.0401515324
H	5.9852269897	-3.9101342004	0.8299383068
H	5.0477960396	-2.4191940091	0.9965509427
H	5.6089311927	-2.5767659025	3.2797085676
H	5.9148882748	-4.3169808888	3.1869410522
H	1.8934646654	-6.1845112872	4.2874386642
H	-0.3889066508	-4.827627276	3.4103979377
H	-1.4124572553	-4.1650692569	4.6930795011
H	2.116240136	-6.0700135513	6.0473426393
H	-0.2021138877	-5.4710325923	6.4044644884

H	-0.3664536617	-6.6410390503	5.0790319301
H	0.3642604527	-2.7558834093	5.5212218503
H	4.4155870773	-6.6210118664	5.853861158
H	0.8166598338	-2.8075387911	3.8220132782
H	7.0841146175	-5.2009848476	5.353831173
H	6.8519336601	-6.7921787489	6.1091718533
H	5.8350101271	-5.7954983971	8.0885376862
H	4.3758947744	-3.9510785463	7.8526695567
H	7.1090946463	-4.6211303838	7.7430484552
H	5.5347725267	-3.025131568	6.8949769219
H	4.3162242215	-1.1669156004	7.8612286033
H	4.7194037315	0.5569408053	7.933575436
H	5.8711796836	0.3328608937	10.019081148
H	4.6739188236	-0.95379058	10.2374377082
H	7.2978635024	-1.6320022807	10.4307863909
H	6.2631744018	-2.6468337345	9.4086589847
H	8.1423462395	-0.4020426662	8.5072870441
H	7.9757399887	-2.028779506	7.82696726
H	4.9014160137	2.2156852265	4.8879054561
H	8.9658436091	-2.477453904	5.7754067389
H	7.2752137146	-2.5355725075	5.2518266475
H	8.0925086722	-2.7821666965	3.0355524555
H	9.7375723915	-2.4398012287	3.5750603345
H	8.4448520271	1.2111588994	4.3246051035
H	9.877131485	0.2234153401	4.6566823533
H	9.3328472109	-0.4120374785	2.3282064436
H	7.5970669158	-0.4407432743	2.7165867282
H	8.3420852957	1.9453827238	7.2096627412
H	5.7000930188	4.7450235619	5.5151282638
H	6.8248628053	1.972844424	8.1275139534
H	4.9479373706	3.7336609023	6.7625063773
H	7.1126057664	4.3521169203	7.797972
H	7.9545651973	4.1086357094	6.2596932742
H	6.4656380856	2.7853442749	4.2794666854
H	-1.7948611916	1.6712737225	6.0301372246
H	-2.4102340498	3.2756173101	6.4711665357
H	-3.414905065	1.5721724197	4.2761173825
H	-4.0458311303	3.1192127841	4.8370498538
H	-3.207092611	3.9338093118	2.7407152461
H	-2.0799448284	2.5709751772	2.6010200489
H	-0.5347838933	4.3223348086	3.1877750796
H	-1.7068527028	5.0986390184	4.275951018
H	-1.2837782223	5.2252380923	6.9153688346
H	-0.4339906782	5.6787706607	9.1268150627
H	-0.0182048688	6.196457667	6.1461830443
H	1.2616235076	2.586890966	2.8505816694
H	2.8597416944	2.4190224943	3.5763713259
H	-0.0612775223	7.2206525594	8.3358719234
H	3.4368587542	3.8436830702	1.7135382373
H	1.8409596969	4.6091032923	1.7115339433
H	3.2175026836	6.3095694465	2.8425715989

H 4.0698338138 5.0586104499 3.7696830374
H 2.3602858614 5.7070068757 5.361390314
H 1.1587564381 5.9442293422 4.0776142483
H 2.699302548 4.1698274659 7.4423709969
H 2.235525263 6.4897279805 7.8561451597
H 1.9935936885 5.8772489084 9.5004220142
H 1.5796789309 3.6226615085 8.6960627978
H 1.7796586769 -2.9872699767 11.5231399577
H 2.9255034892 -2.6180531742 9.3627640521
H 0.3837965492 -3.7024598433 10.6971111819
H -0.3743458562 -1.4048626389 10.90508007
H 1.2652869056 -0.7750553078 10.687571776
H -1.7589992225 -3.2386681898 9.4994821656
H -3.796482814 -4.4801348618 9.0956002645
H -4.6980105844 -3.0088433954 8.6950136349
H -2.5475641592 -4.3481438481 6.9633645995
H -4.2016733095 -3.9367899384 6.4671350922
H -2.1618915179 -2.1740892533 5.9317274225
H -3.7090678886 -1.5475332737 6.521690343
H 0.3615774911 -3.3827900894 7.8898342513
H 2.2364009946 -4.2390318014 9.1540240975
H -2.9982333446 1.1654791024 8.0728086058
H 0.4542490698 1.4776700143 8.9154509345
H -3.3916313576 3.0275265378 9.5701046642
H -1.1390455665 3.1964187473 8.5627606473
H -0.8129327383 3.5118627543 10.275783983
H -2.7436299801 2.1388626096 10.9615492066
H -0.0040233551 1.2659538528 10.6208059545
H -3.4775172348 0.3038418225 9.5478014615
H -2.9423647916 -2.0217534356 10.0088577317
H 1.6389458383 -2.2625580045 7.3634739456
H -0.9527995894 -0.0380572504 -0.685242817
H 1.5759264553 -0.0133896787 -1.4731423298
H 3.4036049006 0.2655913437 5.9283415757

8aa
C -0.0129408973 0.0665435648 0.0029775483
C 0.0263357482 0.0875240851 1.5492218344
C 1.4342244658 0.0395279356 2.1796242404
C 2.2514259233 -1.10932323 1.5649985559
C 2.295192235 -1.0279982552 0.0326914329
C 0.8825691795 -1.0486620373 -0.5632464844
P 2.3528909964 1.7000949565 2.135710667
N 3.6721701002 1.2500892424 3.1561653598
P 5.19530415 1.2085664137 2.8176926051
N 5.7959811937 0.8395004216 1.2699733767
C 5.4855842442 1.7676281273 0.1784044281
P 0.2220320063 1.8195444529 -0.7516688718
N -1.2388953512 1.8650394368 -1.6828656736
P -2.6158319089 2.5507886878 -1.4383924098
N -3.1074196935 3.0006590775 0.1305131989

C -2.169208872 3.8581340389 0.8600700637
N 1.2757971627 2.494067227 3.1928555951
P 1.1781136513 3.9596774385 3.6945211888
N -0.4619637055 4.3129610659 3.9723487715
C -1.3779832247 3.2287389097 4.316009584
N 2.1367094935 4.2174994817 5.0831546086
C 2.3599346649 3.0721374959 5.969569304
N 1.6446716314 5.2918405787 2.7289929598
C 0.8530714751 5.4570049238 1.4980052438
C 2.1328705254 5.4968032446 5.7866374411
N 1.4072265225 1.4914984811 -1.9314021657
P 2.1430105477 2.4980937566 -2.8642593005
N 3.7065795612 1.9030130045 -3.1548878901
C 3.9510887995 0.4640930723 -3.1105820023
N 1.2747630714 2.7748491501 -4.3065820879
C 0.3655995114 1.7218520577 -4.7676598058
N 2.487343764 4.1004811465 -2.38165062
C 3.3588689655 4.2048294266 -1.1986052036
C 1.8743492381 3.5369378159 -5.3982740324
N -2.7810737319 4.0330670009 -2.2437261668
C -2.0109571688 4.2977903726 -3.4516673298
N -3.9027196681 1.5414576414 -1.9730764523
C -4.3721329426 1.7380984434 -3.3478083454
C -3.9158176946 4.9258279999 -2.0467051281
C -3.709475462 0.1119084701 -1.6895473128
C -3.8514344658 2.0940248568 0.998218636
N 5.9698570947 2.7172618534 3.0630356149
C 5.547203434 3.4835657496 4.2327126912
N 5.9068796284 0.0059180736 3.7777796075
C 7.3555259837 -0.0875187192 3.8972901278
C 7.3575961635 2.9815021483 2.6918813239
C 5.1723133365 -0.6197627255 4.872681716
C 5.8988891576 -0.5457736924 0.8119543795
C 3.0862533928 5.4534005642 2.4898177763
C -0.8929826546 5.6160383055 4.46301613
C 1.3685287233 5.0516529177 -2.3435409287
C 4.6991874596 2.6399028924 -3.925776547
H 3.2622437377 -1.1181286834 1.9895050254
H 2.7905187127 -0.0993903697 -0.2729500491
H 2.8856959461 -1.8614711925 -0.3761405847
H 0.9264433966 -0.9717573622 -1.6556409811
H 0.4182032386 -2.0206388769 -0.3244329841
H -1.0441751059 -0.1572747643 -0.2934267624
H -0.5212069326 0.9573829443 1.9344425276
H -0.5196287755 -0.8028648679 1.9054288862
H 1.322161524 -0.1390562457 3.2593644411
H 1.7858039346 -2.0661039019 1.8545598292
H 4.5332836699 1.5262656986 -0.3103304752
H 5.4208565202 2.7879085827 0.5598532141
H 6.2871539708 1.7241891453 -0.5731990584
H 3.2520782863 6.4274972538 2.0130583555

H	3.5011042781	4.6692827523	1.8404724168
H	3.6277759083	5.4357750663	3.4349653034
H	0.9377089778	4.5958178319	0.8214313513
H	1.2042170272	6.3560262802	0.9781107619
H	-0.1997927484	5.5954860837	1.7484860776
H	6.7508320241	-0.6306882458	0.1222970937
H	4.995013497	-0.8717096668	0.281261631
H	6.0651433529	-1.2143051856	1.6579606264
H	-1.009696898	2.292825713	3.8957683885
H	4.1022271929	-0.4962440507	4.7048256694
H	-1.486070851	3.113776222	5.407842104
H	-2.3697332573	3.4448136927	3.8955901131
H	5.4158818487	-1.691203134	4.9131449227
H	-0.9612020235	5.6503207735	5.56313872
H	-0.2061942673	6.3964113251	4.1294274222
H	-1.8900889494	5.8422445587	4.0593708411
H	2.4824357226	2.1650225923	5.376689827
H	7.7463482353	0.5063213676	4.7404625196
H	1.5319850255	2.9348735574	6.6859956551
H	7.6414697183	-1.1353713704	4.0621216169
H	3.2800420994	3.2440285928	6.5441899678
H	7.8406569206	0.2501693004	2.9787314211
H	1.3470726082	5.5497269727	6.5585560611
H	3.1016808839	5.6385813893	6.2855540054
H	1.9876903824	6.3192690031	5.0832197701
H	4.4763408343	3.3661064483	4.3886299143
H	8.0583750293	2.7765706144	3.5184162074
H	6.0834124683	3.1741321225	5.1475005053
H	5.7599663394	4.5473225399	4.0638178462
H	7.462558552	4.0412493619	2.4196921654
H	7.6454627706	2.3754403874	1.8316971222
H	5.4337184959	-0.1705252273	5.8453610401
H	-1.4083118314	3.2921892562	1.4116761586
H	-1.6537998143	4.5191618871	0.1598537887
H	-2.7300837368	4.4781258116	1.5731402175
H	1.7765779258	6.0631153044	-2.2229553933
H	0.6706430419	4.8541369026	-1.517540096
H	0.8112491671	5.012909084	-3.2788689111
H	2.9185008554	3.74239372	-0.3050971647
H	3.545711082	5.2660669279	-0.9976554498
H	4.316935852	3.7223308879	-1.3987110152
H	-4.3079778523	2.6812236314	1.8059660784
H	-3.2116275512	1.3253603324	1.463378943
H	-4.6529670129	1.6105981451	0.4365595767
H	3.1731899177	-0.0229264196	-2.5238205668
H	-3.3700889983	-0.0406925319	-0.6631976752
H	3.9681351509	0.0249210845	-4.1221177293
H	4.9246880969	0.2720045555	-2.6376343785
H	-4.6695030496	-0.4054865339	-1.8124255795
H	4.7205840692	2.3317651587	-4.9841719443
H	4.5036141047	3.7126170479	-3.8762189314

H 5.6971078874 2.4532861476 -3.5032575204
H -0.138171328 1.2689181948 -3.9134456009
H -3.6449089781 1.3812253809 -4.0976039629
H 0.8924969916 0.9418937842 -5.3436988349
H -5.3040951066 1.1758959063 -3.4835887361
H -0.3917417831 2.1741773361 -5.4222083935
H -4.5862892679 2.7894644902 -3.542631297
H 2.4903103991 2.9071191869 -6.0620682807
H 1.0778754009 3.9862258218 -6.0068596554
H 2.4972485015 4.3433553689 -5.0048548627
H -1.140630626 3.6428132202 -3.4878338047
H -4.6205567934 4.8903154419 -2.8948356316
H -2.6149815179 4.1452619106 -4.3625114126
H -1.6657113946 5.3411548109 -3.4465388605
H -3.5601467418 5.9623856398 -1.9536812034
H -4.4487488962 4.6563941208 -1.1335738155
H -2.9613222926 -0.3419053535 -2.3569794428

8aaH^+
C 0.2698582374 -0.0203788662 -0.1111664729
C 0.3934609926 -0.0037862358 1.4242551233
C 1.8746070374 -0.0571012487 1.8555219434
C 2.8190635273 0.9406325876 1.1352384778
C 2.6067997571 0.9066163097 -0.3881859628
C 1.1313970144 1.0616925213 -0.7798560442
P -0.6620738275 1.4040307042 2.1756127415
N -2.1801610375 0.7001455856 1.8507800457
P -3.4277414148 1.28075958 1.1028594067
N -3.9938545498 2.739814312 1.7402401315
C -4.0310043967 2.9219073012 3.1881151655
P 2.7259901863 2.6228430104 1.8874701787
N 3.6844456375 2.535375565 3.1893726911
P 3.7943775046 3.518971247 4.4322179648
N 2.433812756 4.2983464544 5.0317937572
C 1.800293427 5.3590005634 4.2361335761
N 3.0780879216 3.8069053033 0.8621593743
P 2.8655888667 4.7104936732 -0.397642293
N 3.3707737341 4.1620987513 -1.8960938349
C 2.5055485173 3.4908448027 -2.8686189586
N 1.2315738854 5.0667607292 -0.6612818358
C 0.9019344446 6.1082624155 -1.643727201
N 3.8304186257 6.0771532581 -0.1848359051
C 4.0557041083 7.0515376616 -1.2550693964
C 0.3001355678 5.0571672317 0.4736561624
N -0.3762932854 1.2234179506 3.8325588585
P -0.774143423 0.2091503549 4.9648093832
N -1.9583038694 -0.9824721508 4.7498135454
C -1.7187619689 -2.0558650975 3.7774114038
N 0.5389117876 -0.7379129857 5.4702026559
C 0.4412478346 -1.9647338438 6.260272237
N -1.2820393922 1.1341075835 6.2915897088

C	-1.5719453123	0.510644833	7.5788141302
C	1.8403043603	-0.0904393219	5.5969743672
N	4.3268793442	2.5912371669	5.7210434086
C	4.6492058895	3.228266425	6.9992415969
N	4.8100297869	4.8151441054	4.0444037801
C	5.0482906893	5.9062765543	4.9961744162
C	4.9785014091	1.2941766238	5.5225484467
C	5.9862227383	4.524922787	3.2122642365
C	1.4437828963	3.5262442946	5.8054492557
N	-3.2189497819	1.5908359003	-0.5501565165
C	-4.1869836472	1.2705228919	-1.5907675562
N	-4.6671860216	0.148187249	1.2143002199
C	-6.0777157723	0.4947801607	1.3252823454
C	-2.1794379941	2.5253260871	-0.9634819499
C	-4.4090851534	-1.2541583649	0.8959159326
C	-4.769784037	3.7250698488	0.9972565367
C	-1.8944675004	2.4480346266	6.1298225405
C	-3.3706226244	-0.5926668017	4.792952526
C	4.018481971	6.6383561582	1.1526629856
C	4.7991703584	3.921868145	-2.1244493589
H	3.2309172668	1.6651241998	-0.8705068154
H	0.7755871822	2.059922503	-0.4917726575
H	1.0289389909	1.0000174979	-1.8707869539
H	-0.7814555941	0.084385897	-0.4029558503
H	0.598938756	-1.0022831936	-0.4870420756
H	-0.0877441165	-0.9077049268	1.8167129268
H	1.9569339012	0.0484136736	2.9396140157
H	2.2565349917	-1.0618131602	1.6175025991
H	3.8562413214	0.6523207764	1.3555916955
H	2.9663157059	-0.0687873288	-0.747140657
H	0.3866173242	4.1410562754	1.0562425559
H	0.4344255947	5.9272496177	1.1357496573
H	-0.7201274267	5.0871335534	0.0773682
H	1.3240043564	6.0765801453	4.9150548155
H	1.0278081838	4.9518891909	3.5683159071
H	2.5398259185	5.8882359693	3.6357022677
H	0.7565769139	2.9785750434	5.150065955
H	0.8734823281	4.2273874484	6.4256906436
H	1.9430392865	2.8131896106	6.4609545612
H	0.9282801958	7.1154342238	-1.2008900795
H	-0.1082244341	5.9279355817	-2.0289761803
H	1.5936014169	6.0813397777	-2.4873668299
H	4.6633900588	0.8623979529	4.5735906197
H	1.4625681598	3.5468104525	-2.5597775419
H	6.0745280313	1.3896357014	5.5284798683
H	4.6852903912	0.6205635963	6.3370446093
H	2.6170092068	3.9677131791	-3.8516631194
H	5.7198934523	3.4682521856	7.0783662451
H	4.070827366	4.1448808665	7.1301410092
H	4.3906711871	2.5444539797	7.8170417112
H	5.7279763932	3.8067270787	2.4343474476

H	5.0360590476	2.8513895216	-2.0434077406
H	6.8239012068	4.1309680295	3.8084732192
H	5.0722927258	4.2617130199	-3.1314726733
H	6.3150491329	5.4537943602	2.7335022391
H	5.4015802966	4.4688673923	-1.3975277657
H	5.8954534321	5.6928646377	5.664885116
H	5.2794588341	6.8205605078	4.4358210967
H	4.1595546887	6.089615428	5.6016253575
H	3.9682890915	5.8494894717	1.9013111203
H	5.080997971	7.4351266523	-1.1799304369
H	5.0093921442	7.1063175433	1.207787873
H	3.2664766072	7.4104795555	1.3818848324
H	3.3658314704	7.905269882	-1.1842215526
H	3.9378233675	6.5856411113	-2.2341382326
H	2.7795696589	2.4328790622	-2.9673451867
H	2.6312721219	-0.7943668492	5.3079774932
H	1.9011010372	0.7800564248	4.9455324459
H	2.0327714075	0.2328044798	6.6336017492
H	-5.0533887286	2.8126734856	3.5851887457
H	-3.6673575464	3.9251302784	3.448528098
H	-3.3853675848	2.1878715062	3.6681005066
H	-4.369672387	4.7310765232	1.1853550171
H	-5.830171146	3.7249234004	1.2962471627
H	-4.7183592817	3.5309969829	-0.0745287023
H	0.7366468242	-1.7939197765	7.3079308027
H	1.1165884641	-2.724775697	5.8436348599
H	-0.5760641054	-2.3562302936	6.2439213059
H	-1.470088399	2.6877955586	-0.1489280716
H	-0.6581844463	-2.3144727779	3.7448293501
H	-1.6266599956	2.1222353526	-1.8228300694
H	-2.6023381587	3.501750758	-1.2538095227
H	-2.2811513314	-2.9470506898	4.0854239417
H	-3.6679869304	0.8413339896	-2.4586156389
H	-4.9103928979	0.537394564	-1.2337481675
H	-4.7375044195	2.1622639236	-1.9345244644
H	-3.3341059598	-1.427858984	0.8320185835
H	-3.725871497	-0.2656696478	3.8071952337
H	-4.874948233	-1.5435548411	-0.0593369323
H	-3.9650161035	-1.455856347	5.1191810512
H	-4.8187543964	-1.8990807006	1.6852753084
H	-3.5276890373	0.2129700091	5.5126388311
H	-6.6099358862	0.4053228138	0.3642696283
H	-6.5624928147	-0.1818416197	2.0414484078
H	-6.1983810604	1.5142698645	1.6937275391
H	-1.6221448531	2.8611699957	5.1587325454
H	-2.6469301231	0.3047199156	7.7061332098
H	-2.9936565616	2.4034299514	6.2068913852
H	-1.5308190324	3.121103902	6.9182724099
H	-1.2574036816	1.1799388752	8.3904075796
H	-1.0329940107	-0.4296125322	7.6893727064
H	-2.0414030216	-1.7524095316	2.7741080995

H 1.3911899338 2.7811488939 2.3073451189

8bb

C 0.0256763977 -0.0142258263 0.1833981858
N -0.0744718508 0.0252056039 1.6570629244
C 1.2630101771 0.2190695199 2.2640037978
C 2.2337979805 -0.1533296956 1.1387493646
C 1.4949944255 0.3353988301 -0.1172664522
P -1.3353304871 -0.7964254256 2.4354458649
N -1.5689229589 -2.2825435927 1.6705962009
C -0.5366619487 -3.3205592791 1.8394619298
C -1.0539812239 -4.4830243715 0.9845013483
C -1.7527449799 -3.758357999 -0.1772380167
C -2.4455757602 -2.5785205487 0.5226169371
N -1.1596898548 -1.0295743789 3.9610165347
P -0.4163164901 -0.239766049 5.3048366756
N -0.3226846992 -1.4656074577 6.4683366859
P -0.4350143079 -3.0150980894 6.5035320835
N 0.5672407037 -3.7608394034 5.3571603604
C 0.8410600434 -5.2041162431 5.3559277
C 1.8069361114 -5.3742732214 4.1784959459
C 2.6794102063 -4.1125990487 4.2935822513
C 1.6906633294 -3.0172253648 4.7386599558
C -1.9378043194 0.7671418172 5.955349085
C -1.6570256771 2.2855789602 5.9061131437
C -0.7848146926 2.8237452232 7.060941561
C -1.3433049677 2.3598436018 8.4190809125
C -1.5678520562 0.8424286787 8.4780061894
C -2.4785795486 0.3704969105 7.3382916498
P 1.0766978971 2.4493139301 6.8561604071
N 1.6120311834 3.4622903694 8.157734396
P 2.8650666061 3.305988005 9.0758569587
N 4.3650633772 3.1209391057 8.3111407176
C 4.545215944 1.9656351417 7.4063590039
C 6.0246041888 2.0501580797 7.0132543976
C 6.6842487229 2.5927821477 8.2908652399
C 5.6656065178 3.6290132388 8.7915335406
N 1.3962386665 3.3151378967 5.4262585538
P 1.3670806456 4.7982311942 4.9295710866
N 0.3175853496 5.9581574373 5.5905513956
C -1.1035454356 6.0177338057 5.1832948832
C -1.8352804265 6.6060483622 6.4065131889
C -0.7303386234 7.3407839549 7.1818442154
C 0.4842560913 6.4291024564 6.9797705582
N 0.9726077068 4.8338398574 3.2712016068
C 0.0145277957 3.8369454797 2.7442404753
C 0.3011487564 3.7814010443 1.2372883188
C 1.8003649221 4.0960997113 1.1624942073
C 1.9684906551 5.1808004495 2.2355589941
N 2.8420096258 5.5822290373 5.0979694817
C 4.0935186605 4.83927094 5.3595689628

C	5.1938277656	5.9148696721	5.2583399517
C	4.4504251557	7.2310831169	5.5428218556
C	3.1048866411	7.0037358382	4.8431738083
N	-2.6934077497	0.068713788	1.885282414
C	-4.0299285383	-0.4282982643	2.3095946442
C	-4.7360540341	0.7895230626	2.9232562144
C	-4.1102229132	1.9641889127	2.1575063957
C	-2.6402952987	1.5441427596	2.0432650896
N	2.7465585832	2.061560328	10.2009096378
C	1.6334730423	1.0961214838	10.2720882577
C	2.2706903777	-0.1281537797	10.9426648961
C	3.3157320906	0.4950706284	11.8816154019
C	3.8807454579	1.6530673421	11.0427159735
N	3.1566557988	4.674537611	10.0287561182
C	3.3955405974	5.9591200864	9.3190132944
C	2.4834621379	6.9763545015	10.0228870407
C	2.3551009645	6.3994258156	11.4407347558
C	2.2377481753	4.8951895167	11.1710073681
N	-0.0620337744	-3.5031982813	8.0695768246
C	-0.3650488924	-4.8110193215	8.6719546097
C	0.7181043284	-4.9707070567	9.7474426539
C	0.936147191	-3.5231763317	10.2165228611
C	0.8626958449	-2.7177083241	8.9097644556
N	-1.8771960447	-3.8769102916	6.2419448018
C	-3.0129301777	-3.6087391346	7.1566402629
C	-4.1355904557	-3.1059547024	6.242501793
C	-3.9205842562	-3.9569239019	4.9830224478
C	-2.3894127308	-3.9813091626	4.8458010072
H	-2.609654108	-0.7137553689	7.3920683137
H	-2.709506179	0.537804361	5.2094894237
H	-1.202281819	2.5582745786	4.9474666636
H	-2.6283787001	2.8103598855	5.948128548
H	-0.825961679	3.9189419694	7.0372338145
H	-0.6761605223	2.6924534131	9.2230923433
H	-2.3121175862	2.8613498623	8.5869377374
H	-0.6142170389	0.3121608236	8.3879548831
H	-2.0014270792	0.563691037	9.4499394137
H	-3.4765790897	0.8250826076	7.4670156199
H	2.142310118	-2.3036950108	5.4359106247
H	1.8824460426	-3.3730703122	10.7463425145
H	1.3171967336	-2.4415348199	3.8860491578
H	1.2476467079	-5.3698663002	3.2345881594
H	3.1911991358	-3.8527561307	3.3605242855
H	3.447883005	-4.2658598404	5.0623727266
H	2.3802832634	-6.3064976649	4.2316204444
H	-0.0860676226	-5.7769216194	5.24756166
H	1.3294736123	-5.5285095861	6.2916286019
H	-0.3583967887	-5.6146948519	7.930406711
H	1.6396606498	-5.3616175583	9.2952474308
H	0.4176835395	-5.6520465143	10.5512212918
H	0.1223282536	-3.222299611	10.8896860129

H	-2.7315719834	-2.8902176896	7.9320431234
H	0.4901859789	-1.7000550238	9.047969383
H	-3.3139610101	-4.5417562529	7.656939603
H	-5.1307759038	-3.220275657	6.687231266
H	-3.9760488262	-2.0460078221	6.0104031916
H	-4.3979512234	-3.5403470786	4.0887823329
H	-4.3195562829	-4.9685408333	5.1384506385
H	-2.0559219287	-3.1234086003	4.2561497581
H	-2.0279961247	-4.9022272685	4.3716172414
H	-1.7830718007	-5.0706266129	1.5577827165
H	-0.2530847276	-5.1567280787	0.6613465943
H	0.4412087893	-2.9714818799	1.4622372182
H	-1.0028452656	-3.3872906787	-0.8886715239
H	-0.4127133506	-3.578765502	2.8928902978
H	-2.4574733194	-4.3899749935	-0.7288719428
H	-3.4455601819	-2.876720417	0.8726079153
H	-2.5724454681	-1.7067700574	-0.1272317718
H	1.3796396766	-0.3824339565	3.1651438211
H	1.3980127276	1.2639826245	2.5683820147
H	-2.131062219	2.0019209922	1.1905322736
H	-2.0946436977	1.8303538169	2.9523390441
H	-4.2239900608	2.9264647189	2.6680762232
H	-4.5601937057	2.0521945033	1.1597253476
H	-4.4916779609	0.8677194664	3.9891362732
H	-5.8260214692	0.7350618564	2.8268554607
H	-3.9343539973	-1.2565311827	3.0206357673
H	-4.5792040781	-0.7957753931	1.4317069354
H	3.2206615622	0.3051034408	1.2662463887
H	1.6166540301	1.4199326472	-0.2218320191
H	1.8472571843	-0.1308850124	-1.0438677748
H	2.3662690249	-1.2434645205	1.1012841086
H	-0.6804800865	0.6848969312	-0.2836754035
H	-0.2128321738	-1.0198213718	-0.1924182922
H	1.8560360036	-2.6405599728	8.4411082158
H	-1.3644412863	-4.8054950738	9.1356046519
H	4.2434746102	4.0277067978	4.6361947478
H	0.0362153375	2.8092483848	0.8145355825
H	4.0559882924	4.3931345348	6.3552843852
H	4.2911812827	7.3528855802	6.6217200133
H	6.018458689	5.723910836	5.9536212472
H	5.613167418	5.9409377468	4.2440060926
H	4.9777122161	8.1169826771	5.172032046
H	2.2947063446	7.6262330924	5.2319759502
H	3.1942045181	7.220408697	3.7650048719
H	2.9818313515	5.2000910215	2.6453759999
H	2.3836312804	3.2089668372	1.4402696296
H	2.1306590485	4.4267516942	0.1711284409
H	-0.2723166476	4.5526445065	0.7062120397
H	-1.5058386497	5.0285252414	4.9347844839
H	-1.0209781833	4.1343497711	2.9485407638
H	-1.2059760317	6.6524870761	4.2929500754

H -2.6700819493 7.2544913635 6.1189486434
H -2.2392830629 5.7936683079 7.0227111809
H -0.9723227571 7.4850237924 8.240872508
H -0.5414619094 8.3270783727 6.7375461527
H 0.5013246106 5.600473479 7.7004147151
H 1.4328909712 6.9667196633 7.0842881961
H 1.4991549941 6.9913362822 9.5408498847
H 2.8898128808 7.9934419578 9.9976353427
H 4.4521959503 6.2434248078 9.4200186719
H 3.2614229564 6.6110688044 12.0233577249
H 3.1686068035 5.8673418821 8.2521287933
H 1.4948689699 6.7909832723 11.9942618455
H 1.2009769454 4.6360995543 10.907338738
H 2.5362483552 4.2784885808 12.0254312825
H 3.8664661546 2.0264005637 6.5500127866
H 4.3306914352 1.0179705727 7.9273797667
H 4.2397144646 2.487185621 11.6605045601
H 4.7267792199 1.3148228887 10.4235256087
H 4.0927385902 -0.2071222795 12.2029963864
H 2.8238354898 0.8891104965 12.7804921124
H 2.765442654 -0.7496017279 10.1853379938
H 1.5354646355 -0.75226061 11.4604018218
H 1.2427506049 0.8747759015 9.2786059325
H 0.8060413691 1.4927000738 10.8800353443
H 6.4270224542 1.0846563055 6.6891801693
H 6.7924542143 1.7850023136 9.0265815794
H 7.674991174 3.0289643902 8.1217463272
H 6.151240833 2.7648247767 6.1896303056
H 5.6775749456 3.7520698208 9.8789788765
H 5.8712980351 4.6150467091 8.3471080874
H 0.1833987552 2.8639290632 3.2186818394
H 1.7584695144 6.1780733401 1.8169750694

8bbH^{+}
C -0.5659770447 0.3407036693 -0.1634758986
N -0.3405743701 0.7471209677 1.2511055179
C 1.0303769637 1.3106632174 1.4029160936
C 1.766865847 0.781005625 0.1730275887
C 0.6824416498 0.8290608356 -0.9102118011
P -1.5409079296 1.3280040898 2.2722752148
N -2.9270977205 0.6103159528 1.6629819826
C -3.0150442535 -0.846892609 1.3744548099
C -4.4145653531 -1.2560563895 1.8522367995
C -5.2283027519 0.0399051229 1.7167967608
C -4.2319550237 1.1192997729 2.1561500086
N -1.2648708859 1.1506429848 3.8197696515
P -0.2594096694 0.3372439278 4.7951062979
N -0.688805773 -1.1309174703 5.2898751453
P -1.2075002904 -2.5110188505 4.7454219506
N -0.7616626638 -2.7935835748 3.1603380716
C -1.2497574587 -3.9524575118 2.3842947084

C	-0.442622295	-3.8684077899	1.0810063239
C	0.9132431096	-3.3189841313	1.552733195
C	0.5220038141	-2.2740893826	2.6054789886
C	-0.000138285	1.3848320907	6.3022538524
C	1.381894848	2.0901651029	6.2942394195
C	2.5659614144	1.2131037287	6.7581943787
C	2.2437725994	0.5598231376	8.1124694302
C	0.9306526083	-0.2366247835	8.069107493
C	-0.2444663217	0.6548833777	7.6418842466
P	3.1261146468	-0.0506678677	5.4464592089
N	4.6334978528	-0.4804237176	6.1227506074
P	5.444150266	-1.818452323	5.991950956
N	5.4483651656	-2.599226316	4.4944235128
C	4.1681249889	-3.0970954477	3.9464678953
C	4.5857173242	-3.9258675124	2.7242790489
C	5.9517675694	-4.4891532812	3.1431104536
C	6.5935255116	-3.3066882412	3.8799036752
N	3.389215764	1.0241622911	4.1617504795
P	4.4678203799	1.9789944892	3.5646684107
N	5.3279212588	3.0104877033	4.5841212095
C	5.9086616065	4.2959968813	4.1325581298
C	6.7739218572	4.7552125528	5.319415065
C	7.1877287453	3.431746649	5.9804996356
C	5.8994592477	2.6094998364	5.8871217823
N	3.7228648326	3.0507829851	2.4939482931
C	2.6930351274	3.9885533166	2.9816997492
C	2.6515823098	5.0952817069	1.9099631972
C	3.1898591235	4.4037562242	0.6456120179
C	4.2828139107	3.4849997721	1.205828833
N	5.603215399	1.146469195	2.6504153217
C	5.2483642387	-0.1073744887	1.9514924415
C	6.4171847836	-0.3274808877	0.9793649614
C	7.6052578074	0.3230229154	1.705433592
C	6.976241997	1.5724171109	2.3404590337
N	-1.765270449	2.9572753079	2.0375968616
C	-1.7314776165	3.9936210354	3.0920388965
C	-1.6757006264	5.3034173053	2.2920373925
C	-2.4481379745	4.9703298621	1.0054583462
C	-2.0055927067	3.5313198589	0.7013217778
N	4.9584046802	-2.9764491842	7.1079418498
C	4.2328673554	-2.6648562792	8.3567512138
C	4.1813769378	-4.0107204339	9.0975201219
C	5.4603230115	-4.726402866	8.6343306178
C	5.574682152	-4.3163228349	7.1579301625
N	7.0950605676	-1.6077046355	6.2708501487
C	7.874882587	-0.7035017583	5.3882667509
C	8.8040087049	0.0662259173	6.3412126019
C	8.9679233279	-0.899332092	7.5239988797
C	7.5548444806	-1.4711307662	7.6729714224
N	-0.6657275861	-3.8396852353	5.6213998826
C	0.6448535439	-4.4644404751	5.3492712166

C 1.5286887586 -3.9891258659 6.5034853461
C 0.566643149 -4.0540335562 7.7041740464
C -0.8400500772 -3.7803285234 7.1001472535
N -2.8631875138 -2.5691957062 4.9128494822
C -3.6726094208 -3.8055198181 4.9369684231
C -4.7834248502 -3.4878609269 5.9455204366
C -5.0275274847 -1.988533595 5.7108023463
C -3.614782077 -1.4239547032 5.4954612351
H -1.1711605946 0.0724344931 7.594180165
H -0.7737036788 2.1543131766 6.1805659271
H 1.5958798388 2.5163372476 5.3083912073
H 1.2994747169 2.9393524996 6.9898462071
H 3.4336760731 1.8712402841 6.8839509306
H 3.0796537132 -0.0743730472 8.4238535541
H 2.152009031 1.3497109711 8.8754727602
H 1.0210094184 -1.069221082 7.363086472
H 0.7173717012 -0.6712268597 9.0553655207
H -0.3944465074 1.4322571784 8.4058154131
H 1.2789724188 -2.1412144355 3.3845356166
H 0.8206802744 -3.3287343308 8.482313405
H 0.346204992 -1.2990188756 2.1416584782
H -0.91839596 -3.158910693 0.3923246114
H 1.5098814553 -2.8833098254 0.7447029237
H 1.5057795799 -4.1222854388 2.0071962595
H -0.3700568103 -4.8352171439 0.5739294247
H -2.3301924718 -3.8770310257 2.2192972364
H -1.0513079505 -4.8992173959 2.9082750614
H 1.0219509405 -4.1890094433 4.3642999335
H 1.852527862 -2.9609577292 6.3097669332
H 2.4230915171 -4.604342437 6.6373036145
H 0.5938505554 -5.0507830917 8.1596506881
H -3.0573411705 -4.6639714275 5.2202386786
H -1.5709629197 -4.5328591219 7.41987275
H -4.0989720126 -4.0038904201 3.9426121255
H -5.676570035 -4.1023365891 5.7958129276
H -4.4193010071 -3.6573214482 6.9666780076
H -5.5399273623 -1.4961974144 6.5430221557
H -5.6399706693 -1.8468051917 4.8113780243
H -3.1620296828 -1.117696389 6.4459528774
H -3.5924083902 -0.5554175734 4.8305998
H -4.3686772365 -1.5754827097 2.8965879416
H -4.824155514 -2.0846773762 1.2651568277
H -2.9147465775 -1.0104387928 0.2945355566
H -5.5183056789 0.1991902017 0.670914513
H -2.2244445832 -1.4031032521 1.8841238373
H -6.1386047016 0.0478751452 2.3244730654
H -4.228180983 1.2223825037 3.2514560918
H -4.4476766819 2.1022726594 1.7254115573
H 1.5077516631 1.0135738958 2.3386672715
H 0.991013701 2.4072724803 1.3785074745
H -2.764946159 2.9585899632 0.1558144986

H	-1.0821397366	3.5181292083	0.1013783928
H	-2.2372453282	5.6566933132	0.1796805547
H	-3.5280377238	4.9946771869	1.197816968
H	-0.63445883	5.5497467358	2.0498343264
H	-2.1000175518	6.1464368306	2.8457551183
H	-0.8681666839	3.8559419121	3.7484332512
H	-2.6358876579	3.941809283	3.7136807994
H	2.6527782754	1.3754578168	-0.062539791
H	0.5409250302	1.8629705526	-1.2506807854
H	0.905575052	0.2135287721	-1.7874171419
H	2.0900551686	-0.2536279281	0.346961772
H	-1.4937097247	0.7599563197	-0.5623016284
H	-0.641402185	-0.7534067926	-0.2171317281
H	-1.2177207962	-2.7960912368	7.3928614983
H	0.5261131734	-5.5577885245	5.367234124
H	4.2858021757	-0.0185590014	1.4361581998
H	1.6488221556	5.5140617786	1.7797382024
H	5.1702058454	-0.922563741	2.6771863105
H	7.9780979685	-0.3470596169	2.488436041
H	6.5768453339	-1.3874996775	0.7545432989
H	6.2278508809	0.1921293023	0.0311213523
H	8.441017798	0.5676013938	1.0422514734
H	7.4999026705	1.8944636545	3.2464938137
H	6.9932633137	2.4126942355	1.6278217464
H	4.4955053867	2.623682296	0.563922449
H	2.4019829157	3.8062188389	0.1709255138
H	3.5756255561	5.1067098021	-0.0995892084
H	3.3188811982	5.9184400963	2.1930775567
H	5.1273365914	5.0229961243	3.8838852599
H	2.9455580643	4.3848616007	3.9737274918
H	6.530649169	4.1652178067	3.236227365
H	7.6201295858	5.3715116698	4.9994346238
H	6.1713120997	5.3476638543	6.0190803731
H	7.5388494348	3.5523431466	7.0107883072
H	7.9896353297	2.9550852621	5.4019011018
H	5.2184499178	2.8936612372	6.7030546982
H	6.0317275597	1.5281632984	5.948979776
H	8.3138737836	0.9833871087	6.6847108924
H	9.7510974328	0.3451614167	5.8679411207
H	8.457140214	-1.3006660129	4.6740293823
H	9.6744440385	-1.7002938278	7.2720940894
H	7.2162880815	-0.0419910468	4.8147019816
H	9.3179969495	-0.4103282393	8.4385560914
H	6.9195667815	-0.7746000215	8.2419959656
H	7.536922902	-2.4410076993	8.1795091594
H	3.5023459743	-2.2657420564	3.6946264811
H	3.647804099	-3.7233177077	4.6854590652
H	6.6144052626	-4.2893264362	6.8128792118
H	5.0270305239	-5.0144459906	6.505674724
H	5.4236808774	-5.8123580278	8.7679110337
H	6.3250596754	-4.3489492251	9.1940277004

H 3.2971149188 -4.5797302186 8.7882352415
H 4.1293922483 -3.8810775876 10.183143211
H 3.2375164721 -2.271385204 8.1377948852
H 4.7659958644 -1.9086529245 8.949760454
H 3.8535765315 -4.7014486973 2.4772250798
H 5.814617038 -5.3317654435 3.8328785161
H 6.5580359757 -4.8366097287 2.3002428474
H 4.6965303637 -3.2755006162 1.8470483243
H 7.3258219186 -3.6127798597 4.6312395967
H 7.1050946404 -2.6427062273 3.1673834921
H 1.7276219198 3.4763961529 3.0797060393
H 5.2221703759 4.0489513628 1.3225909269
H 0.9841710568 0.2464481405 4.1594923239

9aa
C 0.1970974416 0.4131622887 -0.6946250418
C -0.004079991 0.5648547048 0.8166647231
C 1.4169043602 0.3505476522 1.441669574
C 2.0635163505 -0.697450422 0.4371660537
C 0.9157734584 -0.9359160794 -0.5924521192
C 1.0287273504 -0.1930094711 2.8357162565
C 0.1227342095 -1.3575357715 2.429158321
C 0.7939635421 -2.4020689224 1.4607486428
C 2.23701345 -2.1100270547 1.0231890919
C -0.0472584842 -1.8133186388 0.2668565246
C -0.6970356149 -0.7706783508 1.2312920066
P 3.7803665516 -2.4448349443 2.0514811461
N 3.3484868635 -4.0446275689 2.4705969016
P 4.2104076433 -5.2637554269 2.8992735794
N 5.5481350811 -5.0869807231 3.9508115635
C 5.2201392347 -4.433480668 5.2251877339
P 2.2237474329 -0.4808978462 4.2630307753
N 2.9916030165 1.0419453008 4.139962729
P 3.7920868698 1.8665910914 5.1858283843
N 4.8682424526 1.1133359803 6.2813604539
C 5.8898115775 0.2828306345 5.6286762438
N 1.0675978181 -0.2501472089 5.535025356
P 0.4603995077 -1.3679817057 6.4399418013
N 0.5229945316 -0.8565895525 8.0648353386
C 0.6609814359 0.5667399428 8.3582508417
N 4.8639144895 -2.65545691 0.7131655379
P 5.9687012869 -1.6555504769 0.2490220661
N 7.4045861018 -2.5238482126 -0.0519525725
C 7.3271681304 -3.9620522129 -0.2911557693
N 5.476965646 -0.7718520497 -1.1239647459
C 4.5367694744 -1.3868009624 -2.0569843551
N 6.5397668339 -0.3937209544 1.2356416712
C 7.3486838832 -0.7630428837 2.3995145527
N 4.9292754546 -6.0759801043 1.5833521133
C 4.2818648576 -5.9729087882 0.2739065175
N 3.213799398 -6.3049288095 3.8114282696

C	1.8311089768	-6.4699742389	3.3651615102
C	6.2578147616	0.3059441072	-1.7205115536
C	5.6226622508	0.71600308	1.5446573174
C	8.513609222	-1.8749679271	-0.7413507271
C	5.6226242092	-7.3453618902	1.7832336311
C	3.7405885112	-7.4843665808	4.4901020884
C	6.8000512787	-4.5658910031	3.3874743753
N	-1.1325010132	-1.7540592754	5.9697865596
C	-1.951478956	-0.7205652173	5.342181572
N	1.132117553	-2.9253644124	6.5517543278
C	2.4195310635	-3.0579770407	7.2372891692
N	2.7959633264	2.7494773828	6.2513331486
C	1.4536574364	3.115501718	5.7942863899
N	4.8583112353	2.8874538275	4.3316137172
C	4.3661215939	3.4900573154	3.0937530901
C	-1.9187453568	-2.8255421052	6.5707170317
C	1.0554127164	-3.804184202	5.3730018424
C	-0.1994598586	-1.5986930559	9.0910813242
C	3.3742850304	3.722085913	7.174153559
C	5.8601232045	3.7037701688	5.0086728741
C	4.2961891508	0.4387727545	7.4535915918
H	-0.7463463251	0.3646250247	-1.2554145213
H	0.823964339	1.2031830851	-1.1297112124
H	0.6255849376	-3.4486861092	1.7253410446
H	0.3869301366	0.5832419334	3.2825712201
H	2.4246616264	-2.7925181556	0.1783288221
H	-0.5051985354	1.4742706553	1.1634086138
H	2.0071219311	1.2686262789	1.5034745149
H	-1.7882973937	-0.7306258252	1.304446939
H	-0.7014606865	-2.4854225439	-0.2968442924
H	1.252986924	-1.3935542593	-1.5280569617
H	2.9722496081	-0.2982892004	-0.0214733704
H	-0.445028697	-1.8012053005	3.2504417153
H	3.9625918759	-2.1586224364	-1.54408419
H	3.8407904981	-0.6252267858	-2.4350076591
H	5.5778884074	1.0888021368	-2.0864730438
H	6.9234012317	0.7521127101	-0.9800529114
H	5.0158844282	0.9622539615	0.6717143741
H	4.9496714095	0.4844612934	2.3794271308
H	6.2204925135	1.5963237618	1.8128878677
H	7.9079646727	0.1206374592	2.7348163858
H	6.7251299441	-1.1146673495	3.2337160964
H	8.0594575224	-1.5475439253	2.1333436834
H	8.5950726088	-0.8303949897	-0.4322669285
H	9.4507874982	-2.3815587254	-0.4747265748
H	6.7398447177	-3.4924344559	3.1617818107
H	7.6026823255	-4.7264793564	4.1186901749
H	8.2651011957	-4.4323845856	0.0367845136
H	7.1830326007	-4.1981938431	-1.3598867376
H	6.4981104934	-4.3808560904	0.2773902404
H	6.0344335493	-4.6134822362	5.9386302288

H	4.2984149886	-4.8497964635	5.6349577169
H	1.2048699193	-6.7463001169	4.2235326369
H	1.4663842426	-5.5307059916	2.9481774842
H	1.733434765	-7.2638266285	2.6044790156
H	3.1288705045	-7.6880916133	5.380176459
H	3.4573362025	-6.6971286297	0.1599409133
H	5.0274596281	-6.1806666021	-0.5049354863
H	4.7685001849	-7.3098488223	4.812878023
H	6.1018814072	-7.3709098519	2.764153221
H	6.4032043634	-7.4579673365	1.0179814878
H	4.9438775559	-8.2106645566	1.7012657837
H	5.0859161807	-3.3468788615	5.1063809732
H	3.7168318963	-8.3852825852	3.854438527
H	5.0550776767	-1.8380441176	-2.9200633519
H	7.0553621569	-5.0905751762	2.4675457509
H	3.9060723006	-4.9613642842	0.1271673181
H	6.8613573153	-0.0415027887	-2.5750391633
H	8.4098898471	-1.9124281332	-1.8391857815
H	-1.3073478136	0.0288141894	4.88161209
H	-2.5796049297	-1.1718352239	4.5615151279
H	-2.5398136522	-3.2991671799	5.7967936794
H	-1.2650466868	-3.5886873188	6.995821205
H	0.1074713416	-3.6600160487	4.8520717358
H	1.8721632764	-3.6234183513	4.663244388
H	1.1124159532	-4.8464863048	5.7120874751
H	2.5588770096	-4.108685819	7.5250432523
H	3.2568644488	-2.7627873512	6.5891405081
H	2.4340674299	-2.4429163993	8.1389548698
H	-0.1665814187	-2.6700353272	8.8803721839
H	0.2803590052	-1.4297934706	10.0644633957
H	3.7880033262	-0.4985163519	7.1886514176
H	5.1101501278	0.2110758218	8.1538011067
H	1.1916609833	0.6878698689	9.3134172893
H	-0.317956664	1.0692187435	8.446595036
H	1.2343167455	1.0471465827	7.5667667983
H	6.6891729765	0.0680397642	6.3492883645
H	6.3199798684	0.8153456094	4.7788958413
H	5.216477575	3.7051179412	2.4333515049
H	3.6939344149	2.7944110563	2.5903323427
H	3.8315688702	4.4380323857	3.2778992243
H	6.7210818648	3.8419858687	4.3395740487
H	1.4602036643	4.0295423335	5.1763297596
H	0.8216918094	3.3026121464	6.6725259579
H	6.2070554224	3.2045745828	5.9151597636
H	4.3649318783	3.3994977953	7.5016257535
H	2.7320218424	3.8079999413	8.0616362125
H	3.4625518863	4.7251100648	6.7242676586
H	5.4730315059	-0.6693654604	5.2648216313
H	5.4812232433	4.7040389318	5.2773094331
H	-2.6141848465	-0.2253677508	6.0722446464
H	3.5752470755	1.0840578363	7.9541540275

H 1.0195646648 2.2920861233 5.2289439138
H -2.5916258006 -2.4551651147 7.3615019688
H -1.2542573442 -1.2863749451 9.1760680165

9aaH$^+$
C 0.017987708 0.1711947256 -0.1345562985
C -0.1538505361 0.1848159928 1.417172632
C 1.2799013135 0.3121553511 1.9387709559
C 1.8131892749 -0.8799052517 1.1405678868
C 1.3732677043 -0.579651877 -0.3262571476
C 0.8409729125 -2.0496913625 1.4921940537
C -0.5546876601 -1.3093365513 1.6703165156
C -0.7613152864 -1.0775178708 -0.67108601
C -1.5931760372 -1.5074469012 0.5405513405
C 0.601766271 -1.8422923713 -0.8601722569
C 0.9530303019 -2.8930293969 0.2058623648
P -2.7879521308 -2.9641406354 0.3551445871
N -4.0262901351 -2.1300586162 -0.4691184584
P -4.427178349 -2.1022770994 -1.9793840435
N -4.0200684744 -3.3745649707 -3.0312529511
C -2.5969897676 -3.5828687245 -3.3296092778
P 0.3470903511 -4.6075610007 0.217894531
N 1.3161808713 -5.4822332597 1.1841785157
P 1.2893651836 -5.8452606626 2.7247828243
N -0.138762135 -5.7889653403 3.5975272374
C -0.7333467252 -4.4967022032 3.9834313799
N 0.3817071856 -5.0880931203 -1.3236226317
P 0.2543577044 -6.5185324635 -1.9910414058
N -0.1509257359 -6.2423259975 -3.599042636
C -0.3858720354 -7.366419471 -4.5100401657
N -0.9480714297 -7.5786940738 -1.4715757885
C -0.7731745844 -8.2868112474 -0.1957085042
N 1.6635621057 -7.4225975167 -1.7698441873
C 1.781503994 -8.7597167302 -2.3650111228
C -2.3390765845 -7.1417623568 -1.6696864059
N -3.2811547839 -2.9984480014 1.9838633356
P -4.6454663102 -3.3864916557 2.6402200643
N -4.3349674498 -3.7190205616 4.284533513
C -5.3681031579 -4.2946849451 5.1496870756
N -5.4665434523 -4.8024389964 2.1564686045
C -6.2382158214 -4.7700501348 0.9071499557
N -5.8503024869 -2.2064065336 2.4525779556
C -7.1446307282 -2.3547901292 3.121218146
C -4.6865739273 -6.0378558159 2.2770447171
N -6.1173554108 -2.0775465685 -2.0890699971
C -6.7543488654 -1.8328466266 -3.3823729694
N -3.7163215345 -0.7846532183 -2.7939241137
C -3.8216736991 -0.5907022463 -4.2415390179
C -6.9125680865 -1.6190332888 -0.9510008998
N 1.7481560655 -7.4611838826 2.8478099162
C 1.9831816708 -8.0437756928 4.1718297169

N	2.2736708191	-4.7749339762	3.5766615008
C	2.4087524342	-4.8349065824	5.0354662213
C	2.5258667629	-8.0969871862	1.7816595251
C	3.4369981136	-4.1713712152	2.9200454663
C	-1.1547770075	-6.8287821212	3.4061653262
C	2.9515200005	-6.7204526629	-1.698113683
C	0.3588108822	-5.0430101927	-4.2739150209
C	-3.6106742931	0.483045408	-2.0675662028
C	-4.7464706919	-4.6375451008	-2.88012298
C	-5.4437826431	-0.806412472	2.3009021802
C	-3.5019684853	-2.7443941151	4.995473363
H	1.7578782352	1.2617252657	1.6680375189
H	1.3610436909	0.1760452167	3.0247026891
H	0.8131462464	-2.168673446	-1.8808142693
H	-2.335754856	-0.7125692391	0.7065592818
H	2.0245304242	-3.1154263475	0.0598937502
H	-0.8854295748	0.9033810758	1.7965906634
H	-0.976868664	-1.4856912791	2.6625105321
H	-0.1211217278	1.1345003508	-0.6316483715
H	2.1386853442	-0.1307128076	-0.9639191261
H	2.8703537733	-1.1337244726	1.2679419997
H	1.1342333645	-2.5869005704	2.3971858408
H	-1.3314671448	-0.9541673396	-1.5933733783
H	3.2849793292	-4.138231699	1.841724491
H	3.5665381701	-3.1459394769	3.2873795446
H	2.5399942385	-3.8175100096	5.424405468
H	1.5130388777	-5.2604278138	5.4896348001
H	0.0405603168	-3.7378134412	4.0985098575
H	-1.4701477479	-4.1501684155	3.248362837
H	-1.2421399695	-4.622711398	4.9457472002
H	-1.7265875688	-6.9399941797	4.3353441966
H	-1.8561543728	-6.5578482999	2.6061799395
H	-0.6863739401	-7.7836093607	3.1649580237
H	1.3110734802	-7.6022702457	4.9103679437
H	1.7803234843	-9.120440767	4.1294723617
H	-1.0700569377	-7.6624276638	0.657699282
H	-1.4026019141	-9.1839927785	-0.2043019028
H	2.2893782944	-9.1684437869	1.762787782
H	3.6096320192	-7.9885451954	1.9444975785
H	2.2630212631	-7.6575677617	0.8206522443
H	-2.9948309633	-8.0176160448	-1.6102309769
H	-2.4568120056	-6.6829007535	-2.651090951
H	-0.3270067162	-4.7786806475	-5.086569788
H	0.4071059893	-4.2133785186	-3.5689313373
H	1.3565836184	-5.2101576764	-4.707877582
H	-1.1094359984	-7.057364956	-5.2740010543
H	3.355950612	-6.4984752845	-2.6975590314
H	3.6696931167	-7.3618584815	-1.1744046106
H	-0.8030933861	-8.2178168868	-3.9696137045
H	0.8086622781	-9.2522465705	-2.4050785882
H	2.449595915	-9.3682250115	-1.7433465319

H 2.2009808171 -8.7213364317 -3.3811103419
H -2.6484681441 -6.4129794991 -0.9051595008
H 0.5347823918 -7.6845299644 -5.0213588474
H 4.3579819765 -4.734827372 3.1329313717
H 0.2644311607 -8.5856019025 -0.0559915271
H 2.84729408 -5.7943204357 -1.1353263507
H 3.2799186698 -5.4330038566 5.3399682193
H 3.0218961845 -7.9039547761 4.5071802122
H -3.5088893286 0.2930848727 -0.9991041134
H -2.7227745242 1.0280785209 -2.4130813655
H -2.911340973 -0.0945588748 -4.6038157962
H -3.9182492078 -1.5493643814 -4.7526961776
H -2.08927806 -2.6247331021 -3.453098782
H -2.0812739354 -4.1440633246 -2.5399114263
H -2.5138324529 -4.1430316852 -4.2693580631
H -4.6088302535 -5.2346863208 -3.7905096734
H -4.3850148281 -5.2232240647 -2.0223529776
H -5.8122288998 -4.4487224052 -2.7447518887
H -6.1684897461 -2.2781500037 -4.1893169759
H -7.7477666961 -2.29867075 -3.3866451337
H -5.5914277929 -4.7655157345 0.0180873102
H -6.878838078 -5.659409128 0.8732104333
H -7.8790265299 -2.140847315 -0.9553201142
H -7.1099882786 -0.5354777626 -0.9989931998
H -6.3874659872 -1.8437825098 -0.0243052317
H -5.3688948371 -6.896270747 2.2558106674
H -4.1410266638 -6.0507542742 3.2221662303
H -3.0368769558 -3.2316827248 5.8617244841
H -2.7188603381 -2.3707187461 4.3351058956
H -4.0918867611 -1.8899098225 5.3667710993
H -4.8806083802 -4.8455594483 5.9645903194
H -5.2553269898 -0.3229937362 3.273085082
H -6.2500841308 -0.2579152504 1.7985517355
H -5.9952134073 -4.989118122 4.5890452351
H -7.405499327 -3.4093108581 3.2281108679
H -7.9215472068 -1.8680429274 2.5170279354
H -7.1501712049 -1.8881489674 4.1186131207
H -3.9626214624 -6.1474480164 1.4519494094
H -6.013051293 -3.5255715399 5.6027006552
H -4.4918988588 1.1238285093 -2.2335463105
H -6.8708707013 -3.8852530457 0.8680745861
H -4.5522862071 -0.7441260963 1.6800266809
H -4.680954737 0.041227484 -4.5136511884
H -6.8829136929 -0.7583979646 -3.5885337437
H -0.9710223788 -4.6391144364 0.7101630191

9bb

N 0.239478725 0.4148213164 0.0850002137
C 0.4248790015 0.6914325602 1.5269536046
C 1.9385760765 0.8647263132 1.7167555679
C 2.5245497101 -0.0160806687 0.6025544174

C	1.5556527145	0.2170007707	-0.5654602662
P	-0.9247656548	1.3578569439	-0.728346536
N	-2.322757525	1.0079391155	0.1530865848
C	-2.6507443092	-0.2872023736	0.7555206171
C	-3.1019708825	0.089560014	2.1735160947
C	-3.8682405842	1.4068101315	1.9503298745
C	-3.2104240431	2.0533744182	0.7002189612
N	-0.7062146319	2.8804746008	-0.7797503452
P	-0.2948088893	4.3203825667	-1.563835586
N	0.67941864	3.8768570268	-2.9243674458
P	2.2430462674	3.835700188	-2.9636704202
N	2.8576083909	2.2782848127	-3.1135206966
C	2.1668656675	1.310008547	-3.9937848618
C	3.2580097155	0.2890570404	-4.354452707
C	4.2046888679	0.3429449838	-3.1444001029
C	4.2250373135	1.8372528836	-2.7904789605
C	-1.8369307936	4.6626092879	-2.602890426
C	-3.2713078053	4.2933918366	-2.174092031
C	-3.9218133721	5.2344210937	-1.075276094
C	-3.0088378654	6.3793526584	-0.591007318
C	-2.6805484254	7.0468715548	-1.9344960192
C	-2.055241612	6.1162137745	-3.0410346226
C	-4.0594136901	6.9961564302	-2.6899231703
C	-4.9879811537	6.0249310815	-1.8975492992
C	-3.4350473475	6.0686734819	-3.7806247195
C	-4.0726513164	4.6818144698	-3.4607898915
C	-5.4772754856	5.0344923884	-2.9597925702
P	-1.7450075159	5.9839531081	0.7627085967
N	-0.8160821507	7.4026739857	0.849535683
P	-0.6189058674	8.9304240162	0.7241367538
N	0.5331126199	9.5635813442	1.8119546549
C	1.9670761877	9.3293452669	1.5522348993
C	2.4044533259	8.3478531818	2.6461383054
C	1.6083551708	8.8558074706	3.8582003946
C	0.2561419873	9.285014723	3.2473470649
N	-2.9115421681	6.1298816076	2.033618467
P	-2.6579553457	5.822472612	3.5466554946
N	-2.4181577818	7.2135067508	4.4765393174
C	-3.2558763065	8.4003038935	4.209215527
C	-3.3793381525	9.0865849085	5.5757201348
C	-2.0392040995	8.7460936228	6.2450036554
C	-1.7860120747	7.2936113296	5.8072710595
N	-1.3482400465	4.8919796454	4.0868636804
C	0.0410981361	5.3311358846	3.7742478258
C	0.7860571396	4.0307735263	3.4396687544
C	0.0292813938	2.9917611245	4.2786911576
C	-1.4245939312	3.4153851795	4.0547103516
N	-4.0118803752	5.0017950276	4.1219060255
C	-5.1412572236	4.6360583561	3.2468954193
C	-6.0140764914	3.7782347911	4.1674654734
C	-5.8668615177	4.4838279053	5.5256848361

C -4.3941696116 4.9466723686 5.5415113309
N -1.9128180144 9.9496980533 1.095587732
C -3.3127546586 9.5599220247 0.8476813805
C -4.1566161315 10.7001473702 1.4797765568
C -3.1399450026 11.6097032715 2.2027169015
C -1.8459944541 11.3805014671 1.4134807591
N -0.0533774231 9.3579368345 -0.8028787724
C -0.1359412847 10.7108337279 -1.375211079
C 0.5781962458 10.5848614856 -2.7359495997
C 0.4198054558 9.0960104982 -3.0880866572
C 0.595705989 8.4176540694 -1.7266498052
N 3.1749399942 4.423456542 -1.6687960657
C 2.9178279816 3.8977274616 -0.3029061408
C 2.7612763633 5.1477025583 0.5740696256
C 3.6896396592 6.1539781838 -0.1192059941
C 3.4323828402 5.8814148361 -1.6062307001
N 2.760191249 4.713138898 -4.3004344362
C 1.9999678674 5.8566310098 -4.8385369759
C 3.0946128128 6.7880049947 -5.3726830874
C 4.1568580599 5.7996905268 -5.8831777398
C 4.1315148701 4.6748948396 -4.8294013973
N -0.9247433167 0.5381126079 -2.2108954033
C -1.399682938 1.2043418042 -3.4381714833
C -1.28654365 0.1099182786 -4.5343813552
C -0.9003827476 -1.1900905024 -3.7835837577
C -1.2561935334 -0.8853222533 -2.3224317614
H -6.1104043877 5.4964752329 -3.7297652756
H -6.0129834553 4.1756479591 -2.5335645219
H -2.2086939366 8.0274308955 -1.8386519602
H -1.6288879002 4.090507177 -3.5167404726
H -3.6702105403 7.0740271459 -0.0612833063
H -4.0001640681 3.9420451734 -4.2647580941
H -3.3727818117 3.2382065759 -1.9118745461
H -3.4869807774 6.380776205 -4.8281837772
H -4.5202653309 7.9381534813 -3.0037386259
H -5.7494501946 6.5099545866 -1.2784649038
H -4.357875668 4.6600562012 -0.2526979871
H -1.2136743523 6.5509974654 -3.5886404724
H 1.6701077388 8.3142966612 -1.4991657007
H 0.1481274958 7.4245599442 -1.6516658148
H 1.1469179747 8.7496253316 -3.8300759382
H -0.5867010435 8.8965976432 -3.4760400641
H 1.6431548681 10.8266172924 -2.623778416
H 0.1594532883 11.2612462006 -3.4888120333
H 0.3385302489 11.4610733511 -0.7280985844
H -1.187038968 11.0045281942 -1.5080083041
H -1.8449710226 12.0049606201 0.5036911042
H -0.9357439687 11.6053785764 1.973180217
H -2.9981974324 11.2862581699 3.2400807509
H -4.6777804168 11.2637183114 0.6972338826
H -3.4451097125 12.6621165698 2.2162770818

H -4.9199347827 10.3076583205 2.1600755216
H -3.5054845246 8.5889615369 1.3099155754
H -0.4815643381 8.4817003266 3.3418126623
H 1.4798994738 8.0989306321 4.6392683253
H 2.1198381527 9.716566945 4.3084853748
H 3.4881791053 8.3382765453 2.8105377701
H 2.5195750249 10.2778381613 1.6439088725
H 2.127164915 8.954186122 0.5402851301
H 2.0833883416 7.3364345225 2.3739042795
H -0.716978013 7.0690858901 5.7518265882
H -2.2295168203 6.5834540741 6.5193181643
H -1.2499220914 9.3975587545 5.8498451046
H -2.7599237726 9.0500971928 3.4812853007
H -4.2261431458 8.1145686271 3.7883280076
H -2.0520863628 8.855102939 7.3348593784
H -3.5612049877 10.1636952283 5.4897324509
H -4.2087554793 8.6493798128 6.1472557061
H -5.687495686 5.5272273863 2.9015256091
H -4.7879198109 4.1121208413 2.3573279867
H -5.6123522405 2.7584297554 4.2220086561
H -7.0526944295 3.7163422955 3.8247754019
H -3.7515168079 4.2397654538 6.0883522312
H -4.2911615841 5.9249892338 6.0269922516
H -6.530882063 5.3568882652 5.5616994961
H -6.1125494425 3.8444500589 6.3802859391
H -2.1104942938 3.0463033769 4.8240509948
H -1.772448646 3.0478791073 3.0795051228
H 0.2923067895 3.0927325808 5.3402917973
H 0.2177361774 1.9566874798 3.9806663108
H 0.6749649501 3.8089660273 2.3718311445
H 1.8547839909 4.0896540818 3.671680871
H 0.0667386785 6.0500427014 2.9507051321
H 0.4856214569 5.8100199164 4.658655382
H -0.1515310195 10.1771930326 3.7408348842
H -3.5261125375 9.4692787274 -0.2271259584
H -2.6314045292 2.9534163519 0.924621497
H -3.9792773654 2.3384163686 -0.0280832978
H -3.8201827878 2.064734155 2.8216633812
H -4.927840195 1.2007647874 1.756077762
H -2.2207221738 0.2644876893 2.8018118028
H -3.7117387474 -0.6864127219 2.6506348239
H -1.787282976 -0.9563885858 0.7310092966
H -3.4749275658 -0.7780849017 0.2106822282
H -2.3306412446 -1.0677019733 -2.1382124847
H -0.6856885356 -1.4742138011 -1.5984982439
H 0.1782608978 -1.3708127138 -3.8614979045
H -2.2412001685 -0.0063935903 -5.0589424344
H -1.4177015012 -2.0767281419 -4.1668313851
H -0.5353473933 0.3756691886 -5.2855623054
H -0.7781469668 2.0784405668 -3.637928207
H 1.8536673792 1.0991860019 -1.1435446942

H 3.5559770127 0.244903591 0.3405676948
H 2.510025072 -1.0708852696 0.9072295217
H 2.2720739643 0.5764192598 2.719703865
H 0.053118393 -0.1552789317 2.1231657753
H -0.1358995772 1.5811027503 1.8244976361
H 2.216980367 1.9118029263 1.5590357813
H 4.4552109559 2.0165357269 -1.7362193574
H 4.984602998 2.3653628623 -3.3869546992
H 3.7841332351 -0.2325422173 -2.3110205278
H 1.3383924334 0.8429485583 -3.4522460471
H 1.7488059123 1.8055494116 -4.8780072576
H 5.2057201244 -0.0486812791 -3.3554068673
H 2.8474017638 -0.7096265034 -4.5399636649
H 3.7918986871 0.6055755542 -5.2600511146
H 1.3284345175 5.5282019972 -5.6455719934
H 1.3753461574 6.3130801475 -4.0675554358
H 3.5030787267 7.3924948177 -4.5518781641
H 2.7303485445 7.4697718964 -6.1490364339
H 4.8717486386 4.8595787388 -4.035396974
H 4.3600120563 3.6956106553 -5.2688114546
H 3.8546601601 5.4034789808 -6.8609928209
H 5.1520136988 6.2433628707 -5.994490207
H 4.2855601866 6.1529317107 -2.2389269742
H 2.5625174604 6.45404152 -1.9475949122
H 4.7385652804 5.9431495636 0.1293951814
H 3.4820322029 7.1933131431 0.1502150874
H 1.722754399 5.4965863144 0.5344776427
H 3.0290278404 4.9621675396 1.619407689
H 2.0244049748 3.2689201351 -0.2802407195
H 3.7730408616 3.2873754662 0.0280133246
H 1.5184621171 -0.6325148516 -1.2568183589
H -2.4446720957 1.5371660908 -3.3372494675

9bbH^{+}
C -0.45413298 -0.2339698593 -0.0065548911
N -0.1937138773 0.1133943187 1.4071143949
C 1.2590060945 0.0624120142 1.6996065924
C 1.9073841534 -0.3808308633 0.373301842
C 0.7682032453 -1.0748343827 -0.3894970434
P -1.2598393837 0.8699212123 2.4624763102
N -1.0397602538 0.7417769731 4.0020536522
P -0.0477614536 0.2893018365 5.2949524969
C 1.5564202359 1.2350967124 4.9639285298
C 1.6201222391 2.6521818765 4.3530326298
C 1.3059896206 3.8549913525 5.341520393
C 0.9811820088 3.4335772409 6.7925936906
C 2.2150554598 2.5760621891 7.1217573235
C 2.5425069781 1.401933109 6.1254289599
C 3.6931680861 2.2610097197 5.498607456
C 3.1626049898 2.8044364481 4.1364595347
C 3.3718083341 3.4285764378 6.4837804675

C	2.714141607	4.4970848423	5.5553651115
C	3.4295883705	4.3101424664	4.2149440924
P	-0.7353614431	3.0270525427	7.2831985458
N	-1.4669849429	4.4826605614	7.23792699
P	-3.0059361997	4.8270870586	7.4525569104
N	-3.2683663071	6.2163810986	6.5820247026
C	-2.5192484631	6.5577599786	5.3506643884
C	-3.4552427306	7.5347956209	4.6276028511
C	-4.1736026614	8.2496065221	5.7834672527
C	-4.4146873716	7.1188175214	6.796609722
N	-2.7323325412	0.2168761908	1.9273827297
C	-3.977684579	0.9293281779	2.3096948878
C	-4.8737440365	-0.1481687592	2.940995393
C	-4.4377529426	-1.4252547211	2.2121756913
C	-2.919261071	-1.2443746466	2.1117124077
N	-1.3308051014	2.4995225673	2.0368886321
C	-1.1286484071	2.9963811463	0.6628872336
C	-1.2724990776	4.5265088917	0.7893370159
C	-0.9409318569	4.8011733318	2.2655274759
C	-1.5859651267	3.6020302561	2.9676863959
N	0.6167402603	-1.2440989863	4.9601267052
P	-0.0509745357	-2.6434702395	5.2250751332
N	1.1502491977	-3.6602978784	5.7878488413
C	2.3617248684	-3.1789063961	6.4782895409
C	2.8610953122	-4.4244097008	7.2229816769
C	2.4505052648	-5.5728973614	6.286944845
C	1.0696441175	-5.1291596707	5.774539101
N	-0.7285316604	-3.3389834245	3.8595292749
C	0.0190464587	-3.2917943331	2.5841207018
C	-0.426366939	-4.5603386003	1.8419905237
C	-1.855662151	-4.7856421721	2.3601821118
C	-1.7595580182	-4.3974490201	3.8439204371
N	-1.3284143314	-2.809136509	6.3268487876
C	-2.5680693203	-2.0095767357	6.1696479553
C	-2.803150639	-1.4030090998	7.5583772281
C	-2.2718191458	-2.5032897061	8.4883117956
C	-1.009340581	-2.9870048458	7.7612673325
N	-0.8299718404	2.2276091918	8.67782191
P	-0.3250716019	2.1099349129	10.1646553969
N	0.9217698269	1.012963282	10.2423808229
C	1.8464017797	0.9209516733	11.3937295898
C	2.7187282706	-0.3098413798	11.0732364603
C	2.6200905906	-0.445094508	9.5442369265
C	1.1619333325	-0.0712283329	9.2700795617
N	-1.5287607512	1.6004658349	11.21585914
C	-1.8513952987	0.1632671696	11.3734468145
C	-3.322012634	0.0619886635	10.9550582422
C	-3.9068236653	1.3728538143	11.500493779
C	-2.7835690002	2.3991159982	11.2538761373
N	0.157720995	3.5407351319	10.8787990266
C	1.005695386	4.5158258379	10.1512791358

C	1.5801863515	5.4233687048	11.254627981
C	0.5659834397	5.2917463904	12.4007295218
C	0.1740955507	3.8124346374	12.3347780124
N	-3.384754984	5.0733659568	9.0557750101
C	-2.4633819399	5.8475246507	9.9217189572
C	-3.4005705997	6.6186342307	10.8591273315
C	-4.5918651511	5.6608943731	11.0177458294
C	-4.759177533	5.0583522376	9.6128588393
N	-4.1746945599	3.7255962623	6.9734406099
C	-4.2538398662	2.3486595332	7.5320967015
C	-4.5843720863	1.4782997946	6.3128266462
C	-5.454815063	2.4195781439	5.469251613
C	-4.7149294722	3.7555213259	5.5907369237
H	4.4967020987	4.5620246837	4.2554100651
H	2.9665433459	4.8767669095	3.3969717564
H	2.310713207	2.3206187361	8.1790935097
H	2.0555884892	0.5689442109	4.2504844172
H	1.0838276982	4.3524821953	7.3876904249
H	3.5344788023	2.278600744	3.2523707576
H	1.0464336161	2.7466542559	3.4293927041
H	4.707686589	1.855146279	5.4820366156
H	4.1653522084	3.7993952719	7.1380241465
H	2.6765661726	5.5117477661	5.9637722358
H	0.5788059495	4.5559635022	4.9237034431
H	2.8281685926	0.4637509323	6.6079230539
H	0.5072055989	-0.9331650514	9.4704738544
H	0.9603603742	0.2649115682	8.2519987522
H	2.8702321507	-1.448751738	9.1879322959
H	3.2884538025	0.2687104912	9.047286089
H	2.2957798028	-1.2017704837	11.5513986055
H	3.7445014437	-0.1932652512	11.4355764078
H	1.3073557299	0.8051532252	12.3426320882
H	2.449047601	1.8362470379	11.4597736894
H	0.9193469825	3.1936801204	12.854638456
H	-0.7995420053	3.6039195326	12.7812199434
H	-0.3117761585	5.9215863461	12.2103744909
H	2.5585003778	5.0486477715	11.5793894589
H	0.9776066766	5.5692830925	13.3759287499
H	1.7164333353	6.4529141811	10.9100447954
H	0.3898695963	5.0806276309	9.4420013399
H	-2.9254167767	2.9251500659	10.3045834318
H	-4.8409410752	1.6661028148	11.010190636
H	-4.110035362	1.2774132838	12.5739690078
H	-3.8166955099	-0.8313448954	11.3497941492
H	-1.7237667185	-0.1278450293	12.4255886894
H	-1.1868962661	-0.4603296471	10.7734615932
H	-3.3907493792	0.0404572355	9.8620364682
H	-5.1537886401	4.0385777411	9.6455363965
H	-5.4467014002	5.659072489	9.0048394664
H	-4.3462197472	4.8725228319	11.7385686022
H	-1.816753131	5.1582435434	10.4755643843

H	-1.8227639731	6.5043339874	9.3247688046
H	-5.5047573019	6.1555198671	11.3630676824
H	-2.9231284557	6.8746854434	11.8106048753
H	-3.7256108054	7.552905677	10.3844121003
H	-1.5612088688	7.0266013315	5.6094518648
H	-2.2997561216	5.6646463859	4.7620961384
H	-4.1806279527	6.9791501715	4.0192819519
H	-2.9143577613	8.2193104466	3.9667886593
H	-5.3704112704	6.609267405	6.5982138422
H	-4.4377610527	7.4826191421	7.8295851212
H	-3.5152042464	9.0093920247	6.2222355841
H	-5.103132826	8.7417289332	5.482065298
H	-5.3696774076	4.6206497458	5.4476178097
H	-3.9089254075	3.8109605294	4.8477847867
H	-6.4574584656	2.5014072598	5.9078279495
H	-5.5670033122	2.1030285633	4.4300586878
H	-3.6646089446	1.2190128793	5.7727938568
H	-5.0925713196	0.5497933245	6.5884377892
H	-3.3253941262	2.0543718817	8.0282501601
H	-5.0615594911	2.2956882888	8.2764094486
H	-2.7373504456	3.1518589586	12.0491996883
H	1.8018190009	4.0163455573	9.5889830962
H	-2.6684890348	3.7814816604	3.0926651939
H	-1.1724051351	3.3762798559	3.9515065859
H	-1.3255416851	5.7636642941	2.6177970525
H	0.1443728907	4.7868878562	2.4275962639
H	-2.3077395301	4.8253309228	0.5824523794
H	-0.6250729948	5.0630227228	0.0887114978
H	-1.8591778142	2.5716987771	-0.0380415152
H	-0.1264350838	2.7221648151	0.3076756514
H	-0.5124907597	0.6590799807	-0.6453209604
H	-1.3984898823	-0.7706463445	-0.1114530521
H	0.642784438	-2.1060331037	-0.0400525826
H	2.2505079124	0.4948606087	-0.1916645614
H	0.9294288462	-1.1024470506	-1.4720694804
H	2.7745669433	-1.028206692	0.5378933614
H	1.4416912172	-0.6481709513	2.5120435028
H	-2.4204568974	-1.5959346232	3.0235289105
H	-4.7107749124	-2.3437207868	2.7425993204
H	-4.8864738409	-1.4658747086	1.211596342
H	-5.9411107491	0.0733792963	2.835133036
H	-4.4471394355	1.3613994968	1.415048959
H	-3.7721048078	1.7482511673	3.0051663998
H	-4.6470454644	-0.2387305951	4.0101266276
H	-2.7105282618	-4.0228892933	4.2342679884
H	-1.4700103834	-5.2608923181	4.4586186331
H	-2.5544261529	-4.1182701268	1.8422955344
H	-0.2467674156	-2.3830203294	2.0364496041
H	1.1004550734	-3.2675255901	2.7609698305
H	-2.210481405	-5.8122541723	2.2242439135
H	-0.373403065	-4.4489272615	0.7537257147

H	0.212664824	-5.4073112947	2.122323582
H	3.1018376593	-2.8171326612	5.7512195646
H	2.1308693544	-2.3438263302	7.1456330082
H	2.340556277	-4.5206273097	8.1852941825
H	3.9373890037	-4.3925917714	7.4216545827
H	0.2662102462	-5.4928338626	6.4349478215
H	0.8630054372	-5.5035896887	4.7654715045
H	3.1551325366	-5.6408175539	5.4487089025
H	2.4189474156	-6.5493912083	6.7802911114
H	-0.772014151	-4.0338448662	7.981598953
H	-0.1433522443	-2.3798541744	8.055083273
H	-3.0021437951	-3.3188621644	8.5638969631
H	-2.0554938299	-2.1560317721	9.5028492845
H	-2.2074544858	-0.4876287473	7.6630670509
H	-3.8556704531	-1.1608900118	7.7374155098
H	-2.4607452262	-1.2478579765	5.3920174398
H	-3.4050202947	-2.6669132012	5.8877976185
H	-2.4923206334	-1.7964213891	1.2684860416
H	1.6401688654	1.0403517837	2.0142898868
H	-1.3225868076	2.1824298878	6.3352183669

10aa

C	-0.6586995128	0.2556834273	0.5711785072
C	-0.4864473294	0.4847504674	2.1131234529
C	0.9408566974	0.2799541258	2.6459536222
C	1.1717177646	-1.1933078282	2.2132438435
C	1.0060567943	-1.4140855546	0.6462820111
C	0.6507196582	-0.1247072175	-0.138466042
C	-0.0556695873	-1.8291674841	2.933964729
C	-1.175427754	-0.9052629857	2.394132787
C	-0.321461343	-2.1941199647	0.3891895398
C	-1.343695928	-1.1338024641	0.8567730468
C	-0.437170409	-3.5331894406	1.1198898948
C	-0.2897322722	-3.3129856959	2.644849059
P	2.3435166065	1.550116349	2.4743475511
N	2.9540475019	1.3661740795	4.0915972091
P	4.4195651235	0.9542547164	4.4392636194
N	5.7161478383	1.0930026363	3.3431607324
C	5.7643343516	0.1721099719	2.1996354673
P	1.8780121828	1.259895973	-0.5283357511
N	1.1777367596	1.6563189262	-2.053348061
P	0.6347736598	2.8971926508	-2.8035436922
N	-0.7750931457	2.6054093643	-3.728398180
C	-0.6180911359	1.7139956031	-4.882762088
N	3.2699173672	0.373129231	-0.9192974475
P	3.8059501505	-0.6143352836	-1.991121647
N	5.2477349848	0.0259390103	-2.6347084805
C	6.115106741	-0.7825850351	-3.4816935064
N	2.9773416209	-1.0535873861	-3.408721052
C	1.7244582762	-1.8004083548	-3.258213638
N	4.0675638373	-2.1628581709	-1.313908914

C	4.5165722538	-3.3121004561	-2.092545945
C	2.9163745055	-0.1192230031	-4.536109297
N	1.3748364271	2.9401499938	2.5548972184
P	1.1723702388	4.2671532683	3.3086950266
N	-0.1548256504	5.0726817834	2.5977454856
C	-0.7095339939	6.2390744701	3.2754343664
N	2.3583114096	5.5064594243	3.2707115356
C	3.6451757836	5.1543000948	3.8834366623
N	0.9798355871	4.1088167182	4.9958658189
C	0.9869404714	5.2235774734	5.9365172463
C	2.5568462396	6.1944109091	1.9915530702
N	0.1154836733	4.2171937057	-1.8498045649
C	-0.6476147872	5.328388059	-2.4187383065
N	1.7347899567	3.4096783241	-4.0059575403
C	1.4117754995	4.5169222654	-4.899124326
C	1.045967957	4.6635592795	-0.806822392
N	4.9672911838	1.9419696472	5.7186807158
C	6.248140531	1.6767127156	6.3644096392
N	4.5244636763	-0.7027775448	4.8268234722
C	5.7707903594	-1.3672573747	5.188619055
C	3.9724694988	2.5163368891	6.6225440461
C	3.1644477613	3.2799577817	-3.7344503465
C	-1.9442739555	2.2200275056	-2.923603111
C	4.4574594613	-2.2366818544	0.09060331
C	5.9460749089	1.0999233373	-1.9357785206
C	3.334888967	-1.4093360444	5.2921183108
C	6.1539086681	2.4377082786	2.9631109253
C	0.2480630654	2.9467393202	5.493161329
C	-1.154764113	4.2795249657	1.8847525544
H	-0.9742702225	1.3761969977	2.5109328343
H	0.3850208574	-0.4444772718	-1.153728210
H	0.8478492966	0.2690068078	3.7408211513
H	-0.4216113855	-2.3679623512	-0.69045613
H	1.8675905619	-1.9348241022	0.227113685
H	-2.3647984999	-1.2946142325	0.493728936
H	-2.0932077676	-0.9129659405	2.992446885
H	0.0608970214	-1.6915689981	4.0170826845
H	2.1254058221	-1.6040925894	2.5645780735
H	-1.2506608125	1.0135842876	0.0488576568
H	-1.1877998274	-3.6499028924	3.180615042
H	0.5486521041	-3.8973342501	3.0482294027
H	-1.4031725473	-3.9982689716	0.879717387
H	0.337225017	-4.2161756851	0.7443547019
H	3.3449930355	2.4331493311	-3.0715816256
H	3.7002466248	3.1049877398	-4.6778357812
H	1.9242587434	4.3722207337	-5.8608950129
H	0.3365510557	4.5533332039	-5.0870132827
H	0.2454214315	2.0110026449	-5.480237643
H	-0.4858657668	0.6626683063	-4.578934144
H	-1.5182670929	1.7850978103	-5.505698222
H	-2.8431395719	2.2913507657	-3.548216018

H	-1.8569481481	1.1893350102	-2.543743146
H	-2.0562124932	2.8898035517	-2.068851022
H	-1.3001089453	4.9780811609	-3.220178972
H	-1.2693540108	5.7726558157	-1.629067597
H	0.8658320312	-1.1192070018	-3.176167204
H	1.5808957689	-2.4452864956	-4.136143119
H	0.4952292344	5.2514870993	-0.0670177183
H	1.8512768949	5.2986823783	-1.217284754
H	1.486992439	3.8117009013	-0.2828347935
H	2.7070556463	-0.6895543802	-5.451348255
H	3.8709754264	0.3943867625	-4.6558412269
H	6.3969985913	1.7820950155	-2.6709168252
H	5.2379111278	1.6504759235	-1.3157280534
H	6.7519334873	0.7122242288	-1.2893425521
H	6.6480552037	-0.1307678956	-4.187926089
H	5.5539229667	-2.2187217102	0.2175568032
H	4.0798569036	-3.171715009	0.5269119321
H	5.5273212837	-1.4972053172	-4.061626445
H	4.1754083867	-3.2302555676	-3.125787458
H	4.0907233424	-4.2290913046	-1.661264325
H	5.6143532269	-3.4180842998	-2.089404255
H	2.1296000144	0.6315374072	-4.3936281408
H	6.8702212178	-1.3375999227	-2.899857494
H	3.5801747084	4.1893984865	-3.2688814634
H	1.7566632175	-2.4280751041	-2.365500837
H	4.0251931539	-1.3983452388	0.6341944673
H	1.7304098231	5.4910112716	-4.4920320009
H	0.0043357755	6.1228775107	-2.8186573759
H	2.4411763796	-0.8648649748	4.9886537955
H	3.3034975961	-2.4148846946	4.8486876249
H	5.7725800818	-2.3888281886	4.7813776967
H	6.6254376008	-0.8342821621	4.768703714
H	5.4160403457	-0.8169596987	2.4986050845
H	5.1411318708	0.5241901304	1.3663427133
H	6.8057481353	0.0852997656	1.8582293079
H	7.1916426324	2.3856572521	2.6065000213
H	5.5308208728	2.8527938592	2.155940588
H	6.1128802708	3.1074004096	3.8236516082
H	6.981864929	1.3376653283	5.6298493934
H	6.6225106406	2.6053350538	6.8158505242
H	4.2613169135	4.543056199	3.2092920901
H	4.194284391	6.0752888685	4.1193333022
H	4.3602291714	3.4596839697	7.0321584762
H	3.7456317008	1.8454219023	7.4694981759
H	3.052788734	2.7132911826	6.0712841396
H	3.2023842228	7.065321459	2.1609499958
H	1.602579721	6.5393966977	1.5926771611
H	-1.6701717604	4.924196725	1.1598044124
H	-0.6638419711	3.4674313214	1.3493700846
H	-1.9134611756	3.8531837979	2.5632256738
H	-1.2509808725	6.8582157184	2.5476547787

H -0.8210650545 3.1733091182 5.6470249572
H 0.67174674 2.6293390436 6.4554198518
H 0.0907506971 6.8452454404 3.7067321898
H 1.5308019452 6.0722986453 5.5201670122
H 1.4862075296 4.9130075182 6.8664332445
H -0.0329910946 5.5517011727 6.1970426037
H 3.0371372257 5.5479889714 1.2386928918
H -1.4155586939 5.9668030454 4.0788567141
H 3.3303654666 -1.5189143901 6.389788112
H 3.4941121413 4.5900414032 4.8030066028
H 0.3440460231 2.1166648964 4.7965159354
H 5.9009970585 -1.4414840773 6.2808288097
H 6.1713001503 0.9207140712 7.164323261

10aaH$^+$
C 0.1342302788 0.3642656374 -0.2230667874
C 0.1041634789 0.1763329147 1.2930418585
C 1.4986563945 0.215394856 1.990059901
C 2.2195054518 1.5979617269 1.6669040503
C 1.275790228 2.404170362 0.7138316678
C 0.8669004034 1.6811860293 -0.570375314
C 2.2469878355 2.6318759476 2.8346267022
C 0.7443949668 2.7435544975 3.113610713
C 0.0691547848 1.3783581089 3.4865843247
C 0.9915312559 0.1425682031 3.4504974563
C 0.0981208098 2.6781493684 1.6823181162
C -0.5946648726 1.3275367337 2.0526186485
P 2.1455403828 -0.408472266 4.7416226688
N 1.3917303053 -0.1408225284 6.1439252597
P 1.7732649893 -0.5130358919 7.6347593983
N 1.5859287253 -2.1674819487 7.9188820846
C 0.4557176703 -2.8494693379 7.2733341024
P 3.4235938713 2.7388932376 4.3213520846
N 4.8557655252 2.3969466267 3.4561455619
P 6.3166064626 2.9103829831 3.6865913171
N 6.5407640337 4.5342577169 3.2478865869
C 5.7443223318 5.0564486321 2.1331347242
N 2.4540456584 -1.9765538633 4.4550744456
P 3.610243177 -2.7812260473 3.7351750804
N 2.8984527938 -3.7530274772 2.5739791224
C 1.5183393746 -3.5956893216 2.1198276728
N 4.8616655125 -1.9773396369 2.9612578565
C 5.8068959099 -1.1586946755 3.7363049482
N 4.4443292794 -3.6862861699 4.8891112666
C 3.6500318081 -4.3343165601 5.9375893033
C 4.7458654555 -1.5487210929 1.5659944044
N 3.4007665498 4.4356373975 4.4909455774
P 2.6085323083 5.392847751 5.4385618556
N 1.1297741061 5.8882986431 4.7497104031
C 1.1267502846 6.2079647284 3.320592799
N 2.0770559266 4.8928757706 6.9739035128

C	3.0471255041	4.8264015126	8.0681086851
N	3.5912120658	6.7193834268	5.8122228416
C	4.7575176625	7.0288302187	4.9884271876
C	1.0891366234	3.8079396436	7.0266636063
N	7.3567927337	1.8660957225	2.8324839644
C	6.9201593949	1.4125454534	1.5115863765
N	7.0187539253	2.8759359887	5.2415563655
C	7.1448706682	1.5497468342	5.8552818557
C	8.80995074	2.0282424927	2.9138517039
C	6.5821343818	3.8803757208	6.2201004511
C	7.8508183966	5.1792759953	3.3588753811
N	0.8241000043	0.4657944611	8.6155785753
C	-0.4786215583	0.9424609935	8.1388258704
N	3.3399020505	-0.2233721715	8.1849601077
C	3.7488692135	1.186000822	8.3040680538
C	0.8959772801	0.3157630723	10.0716949824
C	4.4193121305	-1.0631934676	7.6439870137
C	1.9411714805	-2.7602885985	9.2136464219
C	2.9877639916	7.8938247167	6.4402581734
C	0.0942273903	6.6159724779	5.4852098755
C	5.6781498487	-4.4154474667	4.5811955933
C	3.6668949999	-4.7502191352	1.8299282681
H	-0.5944193277	1.3982581386	4.354233985
H	2.5562875956	3.5686334428	2.3539259995
H	0.3144025853	-0.7254123245	3.5129269219
H	1.77060087	3.3452433717	0.4463100471
H	3.2119308084	1.4341794479	1.2375035952
H	-0.5592606907	3.4963442271	1.3735145815
H	-1.6869989057	1.3058596961	2.0005274929
H	-0.375860713	-0.7840499044	1.5233007098
H	2.1214183761	-0.6357335879	1.702085826
H	0.4614696136	3.5800456986	3.7543741782
H	-0.894696896	0.3697543354	-0.6044804058
H	0.6300241703	-0.4990451105	-0.686163446
H	0.2271402779	2.3412974559	-1.1694637639
H	1.7621374592	1.4867319154	-1.1751780146
H	1.8997189697	5.6355601725	2.8079939182
H	0.1530725123	5.9449458817	2.8874223823
H	-0.8926158659	6.3161625224	5.1073875304
H	0.1425112369	6.3833792536	6.5494786883
H	0.3709710067	3.9073212908	6.2105183888
H	1.5539082233	2.8166138969	6.9536963161
H	0.5422492298	3.8732074992	7.9759795671
H	2.5021889942	4.8087916643	9.0203893318
H	3.6783472377	3.9279672027	8.0153492036
H	3.6972593444	5.7017980811	8.0538643806
H	2.197756564	7.5966021526	7.1335446253
H	3.7554723556	8.4306065904	7.0111900318
H	5.597976245	3.6394570931	6.6476050147
H	7.3179692488	3.9201399048	7.0325326664
H	5.532738286	7.4859434105	5.6183449136

H	4.5114310662	7.7386773884	4.181618757
H	5.1449341247	6.1125929354	4.546055611
H	7.8702054522	1.6052718973	6.6760629941
H	7.5015542942	0.8216751198	5.1249762803
H	7.3676614499	0.4327624425	1.2989684984
H	5.8349176617	1.3200754287	1.4964067284
H	7.2300631952	2.1073443902	0.7137986803
H	9.287058714	1.0583917422	2.7213052631
H	6.1989309155	4.8222118716	1.1568228437
H	5.6787877172	6.1471891004	2.2248170952
H	9.1043776318	2.3650161977	3.9089256398
H	8.4275850348	4.7436160118	4.1762539917
H	7.708727964	6.2479212687	3.568009927
H	8.4347207636	5.0916342081	2.4296386739
H	6.1857543198	1.1943198081	6.2632248456
H	9.1934002309	2.7449082409	2.1708486046
H	1.3022324944	7.2814926653	3.1435668021
H	6.5162742251	4.8648103683	5.7606645628
H	4.7356384565	4.650054931	2.1755635946
H	0.186689313	7.7057010095	5.359042702
H	2.5640132233	8.5913192631	5.7002162125
H	0.9825940231	-2.9229762616	2.7880430052
H	1.4834766422	-3.1895901711	1.0975803381
H	3.5823094022	-4.5690769031	0.7490880184
H	4.7250258223	-4.7055425674	2.0904125541
H	4.020838689	-2.1632299938	1.030911684
H	4.4397758291	-0.4985070716	1.4944284165
H	5.721161863	-1.6564774934	1.0757912021
H	6.8066497648	-1.2432256006	3.2921848314
H	5.5189602693	-0.101255064	3.7288550078
H	5.8569128272	-1.5179782606	4.7655875371
H	6.2336721775	-3.9150945108	3.7868020444
H	6.3081943044	-4.4404571724	5.4787710295
H	4.7693793102	-0.6941771547	6.6700312487
H	5.2635671492	-1.0461070991	8.3426014381
H	4.2894357492	-4.5033122021	6.8125764039
H	3.2548075813	-5.3084275402	5.609204757
H	2.8245501778	-3.6866011055	6.2295887416
H	4.6757106235	1.2298672913	8.8865748525
H	2.9824559743	1.7588048866	8.8256063845
H	-0.7117363936	1.8882655275	8.6408697285
H	-0.4395461102	1.1117926158	7.0631375477
H	-1.283199402	0.2257445011	8.3635617082
H	0.6735871751	1.2818495447	10.5403139501
H	-0.4797778662	-2.7208111394	7.839371746
H	0.6758974967	-3.9219473294	7.22154742
H	1.8991995623	0.0123177777	10.3774654376
H	2.7691306978	-2.217457988	9.6717136787
H	2.2550989378	-3.7992084393	9.0532470653
H	1.0910135972	-2.7646225875	9.9114983093
H	3.9209452134	1.644537265	7.3191834506

H 0.170857961 -0.4215193238 10.4476230153
H 1.0145263167 -4.5708454083 2.1224681802
H 4.0851404494 -2.0918254 7.5197310087
H 0.3211476697 -2.4788677093 6.2579184923
H 3.2953300766 -5.7626640006 2.0373890262
H 5.4775247531 -5.4544343624 4.2785143489
H 3.3420975263 0.3214426345 4.6662599284

10bb

N 0 0.0758923248 0.4151600463 0.4058661001
C 0 -0.1213973857 0.6235670051 1.8580373078
C 0 1.2909193809 0.7463608485 2.448446519
C 0 2.1401502367 -0.0919819318 1.4806318746
C 0 1.5163843508 0.2244608575 0.1142484841
P 0 -0.8167905425 1.425456628 -0.6388982189
N 0 -2.4053012784 1.082856908 -0.1757950212
C 0 -2.9099733742 -0.2183818778 0.269465695
C 0 -3.714863127 0.1313599898 1.5292061459
C 0 -4.365733421 1.4773909656 1.1573644197
C 0 -3.3883790919 2.1361838219 0.1446148778
N 0 -0.5618832267 2.9393983713 -0.5543667144
P 0 0.0418226408 4.4153260529 -1.1224711697
N 0 1.3356192645 4.0145770767 -2.2032187116
P 0 2.8554804817 3.9335767015 -1.8447037062
N 0 3.4693326911 2.3689862612 -1.9481188793
C 0 2.9948557081 1.4646872732 -3.0177905647
C 0 4.1271061533 0.4365416563 -3.1657477187
C 0 4.7411179978 0.3900205583 -1.7573830539
C 0 4.6966853431 1.8598145201 -1.3130839696
C 0 -1.1715080374 4.8082948079 -2.5209369206
C 0 -1.2479918012 6.2677696567 -3.0439286911
C 0 -2.0137006276 5.9840115597 -4.3717864971
C 0 -3.2079510168 5.1766459461 -3.8090067634
C 0 -2.6667298906 4.4596271256 -2.5121174534
C 0 -2.4292254468 7.2230201498 -5.1668763735
C 0 -3.28809982 8.1530233119 -4.2765350313
C 0 -3.4686679651 7.5257709851 -2.892896016
C 0 -4.094426014 6.1113754817 -2.9236645946
C 0 -2.1447664653 7.2095731196 -2.128843559
C 0 -2.7757009666 6.4976049839 -0.9038766731
C 0 -3.5616652198 5.3925653069 -1.6272462438
P 0 -1.8517415218 6.0589160799 0.6923835421
N 0 -0.773113132 7.3601276046 0.8103632501
P 0 -0.4284644817 8.8252229121 1.1315921097
N 0 0.3991032776 9.1454271467 2.5889266567
C 0 1.748752055 8.5575113783 2.734748895
C 0 1.7258111611 7.812205913 4.0763433842
C 0 0.7069684182 8.6202238936 4.8947985127
C 0 -0.3592754894 8.9885212599 3.8526364079
N 0 -3.1895464885 6.4618898673 1.7157723622
P 0 -3.4481386586 5.9428764655 3.1676133538

N 0 -3.4764546231 7.2104392469 4.2806225461
C 0 -3.9978472547 8.5343083292 3.8746484966
C 0 -4.2385557237 9.267124776 5.2057793918
C 0 -3.23883645 8.6022288811 6.1660180508
C 0 -3.2846528079 7.1292165902 5.7368212906
N 0 -2.3792123294 4.8806281989 3.9405450263
C 0 -0.9412134061 5.2466832492 4.010618264
C 0 -0.197761826 3.9045734053 3.9150328502
C 0 -1.22329572 2.9072835182 4.4732609106
C 0 -2.523389249 3.4145375574 3.8430233395
N 0 -4.9299550876 5.1434901324 3.2020688951
C 0 -5.6793767444 4.8525456489 1.9621510095
C 0 -6.9050350712 4.0851735746 2.4703865302
C 0 -7.1889776429 4.752310072 3.8253308338
C 0 -5.7811350452 5.0097603978 4.3940762127
N 0 -1.7241779434 9.8951639696 1.345879555
C 0 -2.9879479204 9.7077990502 0.6092633504
C 0 -3.8633204212 10.918761823 1.0336450581
C 0 -2.9466760435 11.8017359374 1.9166107045
C 0 -1.5330439963 11.3345096758 1.547875732
N 0 0.6534304734 9.4195288607 -0.0232457454
C 0 1.5134580606 10.5956476735 0.1369843963
C 0 2.8809199424 10.1010973429 -0.3543332594
C 0 2.5055471422 9.1875052957 -1.5358278135
C 0 1.1069640606 8.6164585916 -1.1769673972
N 0 3.4307569301 4.3863071729 -0.3141939483
C 0 2.8266660674 3.7735318743 0.896574325
C 0 2.7203741836 4.9321200531 1.8988213414
C 0 3.8974279586 5.8290542106 1.4885455893
C 0 3.8035326713 5.7897787926 -0.0397528205
N 0 3.7067853549 4.8960709347 -2.9242467713
C 0 3.0727693211 5.9980483918 -3.6727171009
C 0 4.272598873 6.7813803817 -4.2206924317
C 0 5.3172684655 5.6812951214 -4.4696713671
C 0 5.1244171146 4.7373389339 -3.2690338379
N 0 -0.4397252695 0.6633314179 -2.1050759091
C 0 -0.5635614173 1.3860133406 -3.3841516348
C 0 -0.1796545432 0.330894445 -4.4572529963
C 0 -0.0306246557 -1.0065502141 -3.6876934268
C 0 -0.7550070598 -0.745082079 -2.3604381948
H 0 -2.8660586308 3.3906565393 -2.4160128382
H 0 -3.5246251363 7.1890524981 -0.5036059984
H 0 -0.7263585277 4.2216603581 -3.3321861798
H 0 -4.0790511531 8.1974934659 -2.2751010445
H 0 -1.603683621 8.1159234589 -1.8480682753
H 0 -5.1722084338 6.0959328154 -3.1200749718
H 0 -3.7323074654 4.5665238246 -4.5529461133
H 0 -1.3874477555 5.3447252617 -5.0073096944
H 0 -0.261141323 6.7110969591 -3.2178318458
H 0 -4.2976226447 4.8806938067 -1.0003185315
H 0 -2.9844004399 6.9111994636 -6.0624078014

H	0	-1.529497466	7.74154048	-5.5256111303
H	0	-4.2741503832	8.3314268062	-4.7273413165
H	0	-2.8155364548	9.1388076462	-4.1658016666
H	0	1.1169575212	7.5559850478	-0.9079536089
H	0	0.4234544361	8.723071297	-2.0277961675
H	0	3.2429357577	8.3965056358	-1.7004272636
H	0	2.4476101304	9.7732770821	-2.4613032898
H	0	3.3649482662	9.519475454	0.4390803138
H	0	3.5558936367	10.9162476001	-0.6395878703
H	0	1.5157982175	10.9363733803	1.1752775072
H	0	1.1615222609	11.430895347	-0.491777561
H	0	-1.1885946727	11.8330351263	0.6234412435
H	0	-0.7897018223	11.5204240881	2.3281560518
H	0	-3.127053698	11.5980500782	2.9787772003
H	0	-4.2070174012	11.4697622812	0.1513250382
H	0	-3.0941567814	12.8746016031	1.749764692
H	0	-4.7552160317	10.5940934539	1.5799771325
H	0	-3.4245505711	8.7474188589	0.8860033438
H	0	-1.1100911023	8.1936316965	3.7721415895
H	0	0.2839885477	8.0597812253	5.7359539729
H	0	1.1786008791	9.5266908026	5.296603153
H	0	2.7149273124	7.7578076332	4.5444870369
H	0	2.5068063041	9.3552336683	2.7463097669
H	0	1.9738371413	7.8955729564	1.8951627479
H	0	1.3677774251	6.7886682219	3.9263850187
H	0	-2.3685061733	6.5868744641	5.9841625451
H	0	-4.1152829806	6.6099635516	6.2412715536
H	0	-2.2324974965	9.0063561474	6.0044473673
H	0	-3.2584491228	9.0506095726	3.2554886929
H	0	-4.9161158289	8.4396939209	3.2817001785
H	0	-3.49388523	8.737941014	7.2227480411
H	0	-4.0968501368	10.349571262	5.1131160206
H	0	-5.2638491113	9.0937817136	5.5580107225
H	0	-5.9707283129	5.7801789593	1.4496682013
H	0	-5.0710236132	4.2762002469	1.2617494787
H	0	-6.6547824751	3.0265075837	2.6154639956
H	0	-7.7493536227	4.1405591142	1.774852066
H	0	-5.446295669	4.1708175045	5.0252127998
H	0	-5.7532843116	5.9157290728	5.0091671217
H	0	-7.705809955	5.7077157498	3.667480037
H	0	-7.806523427	4.1430586329	4.4940403127
H	0	-3.4227037488	3.0775656691	4.3680359708
H	0	-2.5865423373	3.0785123813	2.7990778245
H	0	-1.279710445	2.9867589128	5.5671337249
H	0	-1.0089682694	1.8651263099	4.2194757846
H	0	0.0102485808	3.6804007876	2.8622993438
H	0	0.7521641656	3.9112179539	4.4599095113
H	0	-0.656696999	5.9229166674	3.2000248057
H	0	-0.7274358138	5.7524481542	4.9641947133
H	0	-0.8873962752	9.9160451704	4.1013261942
H	0	-2.8259553612	9.7143495104	-0.4798024751

H 0 -2.87142308 3.0167481308 0.5370159675
H 0 -3.9310051316 2.4571336167 -0.7529087118
H 0 -4.5341013639 2.1099672258 2.0329794799
H 0 -5.341402067 1.309265861 0.6855603178
H 0 -3.0289461202 0.2613062491 2.3745732021
H 0 -4.4463970334 -0.6389472944 1.7995189672
H 0 -2.0862184591 -0.9129847352 0.4509248551
H 0 -3.5696578732 -0.667774876 -0.4922020723
H 0 -1.8430754901 -0.9033522349 -2.4738199247
H 0 -0.4069238262 -1.3754067303 -1.5371401819
H 0 1.0266210765 -1.2156873788 -3.4858182291
H 0 -0.9608006669 0.2614774428 -5.222111574
H 0 -0.4442134216 -1.8630387399 -4.2317378475
H 0 0.7496761301 0.6031303979 -4.9685814492
H 0 0.0999006722 2.2520321034 -3.3748614039
H 0 1.9624297736 1.1304485735 -0.3132329413
H 0 3.2070622199 0.1556747379 1.5137527913
H 0 2.0338552471 -1.1601550799 1.7115562398
H 0 1.3439367798 0.3993459994 3.4862853354
H 0 -0.650694837 -0.2375909747 2.2924302325
H 0 -0.7271091841 1.514783261 2.0416410157
H 0 1.614485853 1.7919608684 2.427519136
H 0 4.6504045139 1.9655966599 -0.2255212637
H 0 5.5952329883 2.3962639613 -1.6548036524
H 0 4.1143537354 -0.2186986476 -1.094332779
H 0 2.0570540294 0.9892057983 -2.7137432212
H 0 2.8024444916 2.0142148554 -3.9468366377
H 0 5.756127272 -0.0213836405 -1.7360055296
H 0 3.7623546791 -0.5364805276 -3.5130275242
H 0 4.8741771976 0.7920643282 -3.8876090748
H 0 2.442032958 5.6056425382 -4.4828682541
H 0 2.427639015 6.5986111969 -3.0261813783
H 0 4.6412768296 7.4843879739 -3.4621532377
H 0 4.0239635483 7.3554332179 -5.1197146265
H 0 5.7777788806 5.0255992772 -2.4286379995
H 0 5.3572351545 3.6966956231 -3.5238470604
H 0 5.0831855148 5.1499706552 -5.4011234292
H 0 6.3425681876 6.0588840379 -4.5457775352
H 0 4.7448079866 6.0461621062 -0.5377179945
H 0 3.0307927812 6.4916262022 -0.3827110127
H 0 4.8471599765 5.3873435864 1.8192090248
H 0 3.8422858599 6.8462784212 1.8861053429
H 0 1.7702013402 5.4581080356 1.7447715577
H 0 2.7665701917 4.5942385937 2.9393372808
H 0 1.850953108 3.3299119361 0.6814922625
H 0 3.4844247368 2.9786545406 1.2815170731
H 0 1.6568427462 -0.5855474785 -0.6102172072
H 0 -1.5930072731 1.7406365942 -3.5490391334

10bbH^+

C 0 0.0114228145 0.450628593 -0.2287807265
N 0 0.1443941849 0.2342471971 1.2358418943
C 0 1.5702750802 0.3553807564 1.6447266585
C 0 2.2698165985 0.936421983 0.4091075349
C 0 1.4480764061 0.3647067602 -0.7538575584
P 0 -1.117937213 0.7063104929 2.2330270351
N 0 -0.8870350563 -0.2419056748 3.582010299
C 0 -1.5968807411 0.0953074477 4.8412196947
C 0 -1.5809637661 -1.2158847759 5.6465492279
C 0 -0.3629737776 -1.9713761174 5.0912850737
C 0 -0.4127759405 -1.6421585501 3.5947249285
N 0 -1.2943743305 2.2436208658 2.6177038858
P 0 -0.1151370685 3.3710265797 2.6816033502
N 0 0.9131284784 3.2599357553 3.9202055401
P 0 0.9950456013 3.4268619557 5.4813980655
N 0 -0.3962294022 3.0683467815 6.3365440679
C 0 -1.7087309964 3.665692464 6.0044203292
C 0 -2.5275005985 3.4846954656 7.2910274911
C 0 -1.9406354726 2.2019531024 7.9015793349
C 0 -0.437391282 2.3402135042 7.621063162
C 0 -1.0640580614 4.9376983808 2.6852815111
C 0 -1.737879154 5.4756437023 1.4103885279
C 0 -0.7970868147 6.3440086579 0.5042035472
C 0 0.6403565341 6.523176524 1.019986304
C 0 0.3387653318 7.1711890089 2.3968296026
C 0 -0.5936333556 6.2762041472 3.3277025961
C 0 -1.5245322002 7.6483634525 1.0126533332
C 0 -0.4884296091 8.3911988582 1.8892137689
C 0 -2.0027274609 6.9457893413 3.4021139306
C 0 -2.4475434763 6.7831000115 1.9287043343
C 0 -2.0030707183 8.3920251674 3.8974476582
C 0 -1.1123639402 9.2621908305 2.9799274709
P 0 1.9882337379 5.2035451913 0.9116863917
N 0 3.2688557413 6.2758801518 0.5769131362
P 0 4.7954268983 5.9759441163 0.8303744542
N 0 5.298121207 4.646046025 1.7513832076
C 0 4.97184567 3.2660191959 1.3229011582
C 0 4.3152034697 2.6308444486 2.5542547217
C 0 5.0513633919 3.3068239667 3.7197407772
C 0 5.249174807 4.7508764446 3.2268936546
N 0 1.5778447994 4.378198485 -0.514180873
P 0 1.3573937181 4.5279970031 -2.051325421
N 0 -0.2601083357 4.4102442834 -2.5107284356
C 0 -0.8688082504 5.1031637868 -3.662732078
C 0 -2.3392582492 4.6368598993 -3.6460199128
C 0 -2.5825731601 4.2339959702 -2.1819004845
C 0 -1.2451517312 3.5960799461 -1.7935169258
N 0 1.9019488048 5.9249609563 -2.8161315917
C 0 2.4539044344 5.9792247934 -4.1833308203
C 0 2.867050816 7.4478097302 -4.3496656336

C	0	1.8141593446	8.1962503794	-3.5173844073
C	0	1.6046494818	7.2809427296	-2.3008922605
N	0	2.1275076522	3.2673810299	-2.8488815684
C	0	1.6728944754	2.6469389897	-4.1051827465
C	0	2.7733783495	1.6180684556	-4.4117451414
C	0	4.0400558576	2.287556679	-3.8519253648
C	0	3.5400489171	2.9475321778	-2.5586924693
N	0	-2.5230503863	0.2923740807	1.4432610413
C	0	-3.3190509978	1.2487965073	0.6372882275
C	0	-3.9067871848	0.3717522871	-0.4743353011
C	0	-4.1189905282	-0.9784270131	0.2286818284
C	0	-2.8790917228	-1.1069821396	1.1312209928
N	0	5.4430687767	7.3074650339	1.6156746601
C	0	4.6490713636	8.1682047658	2.5160055318
C	0	5.6531489006	8.5654796371	3.6043881736
C	0	6.9729721693	8.6713175826	2.8231781842
C	0	6.8905953349	7.5039726894	1.8203441156
N	0	5.6307604998	5.6884310882	-0.5937044101
C	0	5.2421965538	6.3766951162	-1.8379115182
C	0	6.1969246996	5.7967949229	-2.918058188
C	0	7.0223004155	4.7125706029	-2.1900154513
C	0	7.0009094406	5.1796165115	-0.7301727375
N	0	1.4049762956	4.9695598408	5.955394368
C	0	0.9606198708	5.6309136022	7.2016344039
C	0	1.8338762092	6.8986952058	7.2745941035
C	0	2.1962781944	7.1807347937	5.8066134262
C	0	2.4129530556	5.7738498466	5.2428293399
N	0	2.1820828831	2.4039660003	6.0295328141
C	0	3.0118057466	2.6342490474	7.227344666
C	0	3.985127417	1.444771507	7.2131117868
C	0	3.1499790797	0.3185781615	6.5806804166
C	0	2.3728137993	1.0462198319	5.4758543639
H	0	-0.8586993105	6.1170802115	-0.560262068
H	0	-1.8862757325	4.5752020116	3.3181118737
H	0	1.0892727743	7.3147449378	0.4102424792
H	0	-2.6471331186	6.3481125551	4.0609831429
H	0	-0.1498955075	6.1338880326	4.3146143896
H	0	-3.5261661089	6.8742091185	1.7698745149
H	0	-2.0271831578	8.2782608382	0.2721780624
H	0	0.162164999	9.0135860499	1.2634738032
H	0	1.2429482758	7.47794169	2.9319141935
H	0	-2.3602064116	4.7432785225	0.8902100833
H	0	-1.694905895	10.0672654386	2.5140807314
H	0	-0.314223576	9.7511080232	3.553677212
H	0	-3.0346983218	8.7655996302	3.9194767766
H	0	-1.6444747532	8.4161365501	4.9355407746
H	0	3.4293575027	5.42135016	5.4745734771
H	0	2.2639727991	5.6949236915	4.1624576473
H	0	3.0801091161	7.8163275674	5.6941470629
H	0	1.3599745975	7.6686408592	5.2920725731
H	0	2.7451792464	6.6918307537	7.8491553949

H	0	1.3135683227	7.7287502094	7.7619470518
H	0	1.0998408763	4.9911113365	8.0808277979
H	0	-0.1063026188	5.8828788069	7.138196498
H	0	0.0511519561	2.9098721939	8.4258784628
H	0	0.0727655252	1.3744450409	7.5385185131
H	0	-2.3414634231	1.3214279759	7.3860125258
H	0	-2.3599993286	4.3326032242	7.9668114786
H	0	-2.153209296	2.093680951	8.9694354651
H	0	-3.6016626802	3.4180164301	7.0936305941
H	0	-2.1602235538	3.1302997252	5.159434088
H	0	2.9544398772	1.100599313	4.5476301813
H	0	3.7584544054	-0.503598662	6.1911758588
H	0	2.4549785146	-0.0969870875	7.3220488565
H	0	4.358263479	1.2021408798	8.2125551336
H	0	2.4076181439	2.6281828681	8.1468210069
H	0	3.522265607	3.6017624092	7.1687254325
H	0	4.8470417979	1.6746275794	6.5752915941
H	0	0.5599066502	-1.728282549	3.106366667
H	0	-1.1095795202	-2.3158418121	3.0759406809
H	0	0.5635882756	-1.5808847885	5.5302201544
H	0	-1.0611750828	0.9007293603	5.3495604799
H	0	-2.616524118	0.4469450602	4.6445923051
H	0	-0.3982455413	-3.0485323751	5.2812560611
H	0	-1.5241985721	-1.0381256286	6.7254104415
H	0	-2.494819696	-1.7906698718	5.4529755443
H	0	-4.1082063017	1.6962215677	1.2560002248
H	0	-2.6972798785	2.0629779822	0.2589253466
H	0	-3.1800447012	0.265053193	-1.2900775165
H	0	-4.8280874025	0.7885142673	-0.8936093659
H	0	-2.0572446719	-1.6184683871	0.6084977851
H	0	-3.0937267999	-1.6688647597	2.0470460313
H	0	-5.026738454	-0.9410207545	0.8432154382
H	0	-4.2161010303	-1.8210364316	-0.4625813157
H	0	-0.6519572927	-0.2993717399	-0.6717912006
H	0	-0.4004453248	1.4412705443	-0.4398618861
H	0	1.7226511949	-0.6795853031	-0.9501429105
H	0	1.568458891	0.9404056062	-1.6747608481
H	0	2.1987766989	2.0288100537	0.3992630237
H	0	3.3283434654	0.6610751023	0.3679039261
H	0	1.6809202411	0.9839702239	2.5316254193
H	0	1.969788805	-0.6397578766	1.8870956767
H	0	1.4146490935	0.5743297871	5.2372441247
H	0	-1.6073296777	4.7201251532	5.7227616988
H	0	-1.2165748655	2.5440212244	-2.1250788286
H	0	-1.0393617972	3.6205012148	-0.720595241
H	0	-3.4295471448	3.5507893397	-2.0584018665
H	0	-2.7665921006	5.1212338433	-1.563869297
H	0	-2.4608521499	3.7608934932	-4.2952705785
H	0	-3.0230572411	5.4129180133	-4.0036940953
H	0	-0.3798508153	4.8414543546	-4.609581278
H	0	-0.7891947692	6.1915129365	-3.5415661525

H	0	1.7017376673	5.7106701106	-4.9413131977
H	0	3.2946031821	5.2871240543	-4.2921915586
H	0	3.8668767194	7.6099116003	-3.9318520814
H	0	0.8821999466	8.2866082318	-4.0900737696
H	0	2.8871487027	7.7543337951	-5.4003404884
H	0	2.1287573904	9.2038871758	-3.2278672549
H	0	2.2896626416	7.5216481495	-1.4796788893
H	0	3.6064166592	2.2563124655	-1.70804284
H	0	4.8594055064	1.5834128172	-3.674555942
H	0	4.4014791423	3.0520791916	-4.5517230055
H	0	2.8391539157	1.385166178	-5.479119912
H	0	1.5965403864	3.3769939061	-4.9252196684
H	0	0.6852086115	2.1887816691	-3.9780264665
H	0	2.5754560547	0.6820838582	-3.8747907616
H	0	7.1937627569	4.3850816028	-0.0071202088
H	0	7.7519659777	5.9716471695	-0.5730773605
H	0	6.5294451308	3.7362555953	-2.2652687655
H	0	4.1897007292	6.1787077569	-2.0379656105
H	0	5.3665174214	7.4668710563	-1.7506264697
H	0	8.0374589609	4.6117348272	-2.5876595058
H	0	5.6471974709	5.3896200686	-3.7729700292
H	0	6.855659184	6.5825630426	-3.3034645448
H	0	4.2786031615	9.0506821815	1.9749696709
H	0	3.775863401	7.6361391372	2.901970346
H	0	5.7215814003	7.7692625201	4.357748769
H	0	5.3790064196	9.4950610815	4.1145361584
H	0	7.3602851669	6.5956387144	2.2257342576
H	0	7.3939838925	7.7428583836	0.8764875573
H	0	7.0092911377	9.6260658827	2.2843331034
H	0	7.8633570463	8.6114180604	3.4570118421
H	0	6.1703749956	5.1993400937	3.6179949429
H	0	4.4136815415	5.3820313949	3.540662695
H	0	6.0237849775	2.8279351753	3.8918879482
H	0	4.4830984009	3.2693084808	4.6547370082
H	0	3.2542541926	2.8961106587	2.5856415316
H	0	4.4085316725	1.5392188085	2.5624818213
H	0	4.3165397513	3.2706475196	0.4484515396
H	0	5.8943969965	2.7303656434	1.0490961245
H	0	4.1074027353	3.8425931451	-2.2894512331
H	0	0.580740536	7.3448318166	-1.9146645898
H	0	0.6644664194	3.2568662906	1.5323480272

Cartesian coordinates of mono phosphazenyl phosphines and their protonated forms **1a-10b**

1a
C 0.0790117949 -0.0199031057 -0.338622622
C -0.0196207783 -0.0138230995 1.0803972562
C 1.1692813741 0.1584139553 1.8700381856
C 2.4351232036 0.3436570526 1.2120633905
C 2.4716551811 0.3284632377 -0.171365811
C 1.3012250002 0.1482987753 -0.9497165653
C -1.2766000127 -0.1612970994 1.7306619605
C -1.3728162188 -0.1430143021 3.1052028274
C -0.2036253003 0.0155511101 3.8893896
C 1.0290825334 0.1602412468 3.2880810401
P 4.013971042 0.4958911919 2.223688159
N 5.0960004413 0.8040609081 0.9370914911
P 6.634771694 0.5113697114 0.9373101617
N 7.5338256006 1.9425726123 0.8789495123
C 6.9946474768 3.0546832543 0.1007175355
N 3.775534423 1.9507348359 3.0532860599
P 3.2745954496 3.3975868906 2.7288571658
N 2.4149005553 3.8396975735 1.3407298642
C 3.1007166979 3.9639460969 0.0532264651
N 2.1987929142 3.8666850411 3.9513999775
C 2.1425817756 3.1564439843 5.2243000232
N 4.6085146419 4.4598156439 2.6144212026
C 4.3981125363 5.9022844099 2.529382313
C 1.5104934338 5.150273658 3.9665346388
N 7.3902456104 -0.282707901 2.2449453021
C 7.3933538407 0.3783779609 3.5569844837
N 7.0041312596 -0.4159478823 -0.4307754227
C 8.3796874742 -0.6905104952 -0.8224996342
C 7.1751551865 -1.7288793278 2.3596841032
C 6.0351448976 -1.350077058 -0.9975298247
C 8.9612181006 2.0591746242 1.150350584
C 5.8264855394 4.1080751726 3.340091763
C 1.0021725944 3.4750179711 1.1913320004
H -2.1676032737 -0.2879137144 1.1183105694
H -0.8253267429 -0.154949508 -0.9287857413
H -0.2790815016 0.022188687 4.9743098181
H 3.4275316097 0.4846043699 -0.6632522374
H 4.1132934205 4.340853158 0.1984583992
H 2.6989120804 2.222839246 5.1460508469
H 3.1546275937 2.9993891176 -0.4698069947
H 2.5733457363 3.7640858345 6.0368079684
H 1.0970303586 2.9305863962 5.4796723772
H 0.5697465533 3.1989118413 2.1537261045
H 0.446219496 4.3323980544 0.7850936263
H 0.8841354999 2.6270629594 0.508099406
H 0.4638083857 5.0039190469 4.2711193225
H 1.9731286296 5.8555195744 4.675787786
H 1.5107629844 5.6023928015 2.9737675327

H 3.527742559 6.1267934843 1.9092965152
H 5.276746324 6.367821938 2.0631329004
H 4.2552556134 6.3665314133 3.5198689902
H 5.7953715527 4.4387744949 4.3929527383
H 6.6850328107 4.5924668927 2.8568344081
H 6.0308910606 -1.2594286107 -2.0926999534
H 5.0379838258 -1.122789885 -0.6215716913
H 6.2810109338 -2.394801512 -0.7434900472
H 8.4452161615 -0.7429529728 -1.9176446682
H 8.7451618401 -1.648162324 -0.4144943653
H 9.0501144797 0.0997074619 -0.4853777138
H 9.3295047148 1.1703403834 1.6649590002
H 9.5419803234 2.2004683071 0.2238327105
H 9.1434937564 2.9339527914 1.7918708423
H 7.435325961 3.0918948801 -0.9091186938
H 7.9631337916 -2.1533787751 2.9949644372
H 7.2177226633 4.0009512938 0.6102590757
H 6.1973343308 -1.9676450237 2.8037804236
H 5.9130915609 2.9517158813 0.0203684293
H 8.2152031113 -0.0329103726 4.1582801168
H 6.4479666228 0.2280835215 4.0972510933
H 7.5577933783 1.4508243036 3.4404411799
H 7.2405978913 -2.2052734594 1.3795168488
H -2.3403467938 -0.2546920052 3.5887443042
H 1.3744364286 0.150674667 -2.0354832761
H 5.9667312774 3.0296864146 3.3121883192
H 2.5472130283 4.6748430632 -0.5750099926
H 1.9127775911 0.2893310921 3.9015673031

1aH$^+$
C -0.0298450783 -0.0983058214 -0.2759227695
C -0.1138342561 0.0269313759 1.1377586057
C 1.0859411407 0.2249159777 1.9028305838
C 2.3372425894 0.2847087493 1.2005617277
C 2.3720538933 0.1518101533 -0.177406449
C 1.1843270449 -0.0340035946 -0.922674417
C -1.3681363103 -0.024735507 1.8058302134
C -1.4486279373 0.1154392885 3.1738830899
C -0.2684905705 0.3102106293 3.9314578034
C 0.9635147301 0.3643648701 3.3136455251
P 3.9004317076 0.5597875976 2.089326779
N 5.0685481554 0.2655777425 1.0249653373
P 6.6488726634 0.1369280859 1.1577017112
N 7.3767607829 1.6443953011 1.1428185162
C 6.8907491774 2.6214990578 0.1617128597
N 3.9304408182 1.9492327722 2.9027591533
P 3.366084985 3.4121032992 2.6273839851
N 2.4670880216 3.7538090108 1.2631448397
C 3.1066727672 3.8816818689 -0.0512639025
N 2.3392616777 3.8036712539 3.8912241475
C 2.4073200359 3.1239470069 5.1872763129

N	4.692899954	4.4453380275	2.4593193314
C	4.4565759779	5.8890762869	2.3243101104
C	1.6336123406	5.0873024842	3.9474916157
N	7.2814653304	-0.5665167515	2.5501980308
C	7.0948160729	0.0922976572	3.8507878218
N	7.134259105	-0.7722954299	-0.1529061043
C	8.5555985671	-0.9088169188	-0.4749537319
C	7.3144500411	-2.0304405113	2.6549701839
C	6.2733329895	-1.7657297685	-0.8011877542
C	8.7365406094	1.9226767333	1.6132251679
C	5.9170270776	4.1405262421	3.2089254205
C	1.0260211156	3.4805300863	1.1748838585
H	-2.2677933123	-0.177389427	1.2145101042
H	-0.9463171601	-0.2468878058	-0.8420776173
H	-0.3345451578	0.4166628074	5.011027031
H	3.3297397139	0.1868744755	-0.6876600201
H	4.1573732117	4.1498348082	0.0589714309
H	2.9862445418	2.2052671158	5.1006675272
H	3.0323484602	2.941158704	-0.6145010353
H	2.8776499156	3.7684058554	5.943648833
H	1.3921956912	2.8775054494	5.5233249098
H	0.6207953764	3.2059648379	2.1484717168
H	0.5069369589	4.3758016339	0.8094778348
H	0.8365609867	2.6563830681	0.4790024962
H	0.6120390861	4.9220978552	4.3119663087
H	2.1333455504	5.7901423012	4.6287356111
H	1.5702666121	5.5427587259	2.958603584
H	3.5700002627	6.0784631594	1.7164662978
H	5.3172097434	6.3424689444	1.8196840704
H	4.3312887966	6.3824707747	3.2996369324
H	5.8392405173	4.4203745806	4.2710872247
H	6.7439306658	4.7096687435	2.7695335061
H	6.3763788121	-1.6757109436	-1.889272245
H	5.2325332921	-1.5907646695	-0.5296704855
H	6.5544810243	-2.7889882822	-0.5109753538
H	8.6883441486	-0.8591902738	-1.5620763366
H	8.9616893767	-1.8680191043	-0.1214893271
H	9.1386279269	-0.1046046794	-0.0248571342
H	9.1055422777	1.1078112558	2.2364627171
H	9.4277073132	2.0648603331	0.7700032724
H	8.7322784484	2.8458490107	2.2066319307
H	7.5120712763	2.6151624484	-0.7455109837
H	8.1147319503	-2.316811556	3.3462932035
H	6.9209824645	3.6234720642	0.6020947657
H	6.365450675	-2.4375610756	3.0349229189
H	5.8593950652	2.3984406409	-0.1113605582
H	7.9553951423	-0.1283931577	4.493132516
H	6.1850850984	-0.2606410423	4.357388619
H	7.0220709563	1.1732366637	3.7285821781
H	7.5251848526	-2.4834628668	1.6855714917
H	-2.41210443	0.0734534725	3.6736938587

H 1.2386252955 -0.1325547941 -2.0030941023
H 6.1425814821 3.0786806026 3.1285790291
H 2.600706936 4.6695794581 -0.6221959569
H 3.9259323066 -0.4215669239 3.1001534475
H 1.8476594951 0.528950759 3.9180568399

1b
C -0.0876691848 0.0016787703 -0.0981674135
N -0.0398708846 -0.1896301129 1.3639726831
C 1.3385763033 -0.0657866109 1.8411912785
C 2.145667118 -0.6615659255 0.6828263887
C 1.3878127918 -0.1399727811 -0.5555252639
P -1.4359713154 -0.2029208777 2.3002551035
N -0.9929503277 -1.3474438393 3.4665723825
C -1.7274452459 -1.4585900246 4.7397893202
C -0.9879046667 -2.5948273482 5.4517007906
C -0.6953579821 -3.5786586263 4.3035395487
C -0.4788536964 -2.6800380958 3.0651225355
N -2.7317640968 -0.3193347774 1.4167959148
P -3.6332621725 -1.5991167975 0.7834575085
C -4.2615232378 -2.4378459135 2.3525937333
C -5.1924710335 -1.7539198013 3.2095489893
C -5.660745336 -2.3996688302 4.4058874652
C -5.2205739217 -3.7191748477 4.7040999316
C -4.3613808404 -4.3722466916 3.8494941739
C -3.8880152044 -3.7280339626 2.6799643915
C -6.5590721442 -1.7100606971 5.2674158918
C -6.9979580754 -0.4389393 4.9661797538
C -6.5573806748 0.1943736903 3.7779729778
C -5.6797260488 -0.4445896515 2.9268884722
N -2.5120297775 -2.7921832546 0.295729223
P -2.1647676805 -3.4674567303 -1.0664433058
N -3.395144425 -4.4502161767 -1.6468912481
C -3.2820433261 -5.2929395137 -2.852738762
C -4.2919201081 -6.4232360987 -2.6025483577
C -5.3830230792 -5.7229805132 -1.7770098053
C -4.578166717 -4.8160854292 -0.8354488266
N -1.6579389063 -2.4109264198 -2.2821947301
C -0.9715477129 -2.7952971127 -3.5212392116
C -0.4792295852 -1.4409970963 -4.0697525465
C -1.4981506737 -0.401195975 -3.5203179475
C -2.4449632885 -1.2119736558 -2.6058393598
N -0.8004558535 -4.4666094701 -0.9645545671
C 0.4438911869 -3.8096987773 -0.4792780244
C 1.163419967 -4.8846931817 0.353988301
C 0.0122298451 -5.7571795108 0.8775147621
C -0.9443670553 -5.7937983756 -0.3209661216
N -1.5643673169 1.2235437616 3.1789648357
C -2.8632842245 1.8539773626 3.4968936854
C -2.6153475646 2.5165035104 4.8576814668
C -1.1389464385 2.9322271962 4.7728652134

C -0.4837123016 1.7557876202 4.0263169603
H 1.4694518885 -0.586545102 2.7935691323
H -3.1874391496 -4.2424367334 2.0282218349
H -6.9139686887 1.1927724171 3.5354735701
H -4.0303710767 -5.3843977806 4.075657387
H -7.6861184953 0.0756756937 5.632558236
H -5.580193737 -4.2026548746 5.6103775878
H -6.8996135281 -2.2098839842 6.1725029432
H -1.5830208765 -3.0413868641 6.255362346
H -0.0528264784 -2.2157498899 5.8839406474
H -1.6921193367 -0.5114239656 5.2869000572
H -2.7823297651 -1.7246647196 4.5867973415
H 0.1690502765 -4.2213982341 4.5036630215
H -1.5640542477 -4.2252837715 4.1456682591
H -1.0116027136 -3.058395383 2.1871948814
H 0.5843762755 -2.6012789106 2.8039362439
H -0.1156658991 0.9926444197 4.7295241248
H -2.7576619974 1.7826412411 5.6618266448
H -3.2918904709 3.3572717463 5.0454464828
H 0.3705615837 2.085683056 3.4251116851
H -1.0440252257 3.8505136693 4.1791743056
H -0.6756074113 3.1137471276 5.7486804756
H -3.1236936661 2.6006605934 2.7323682317
H 1.6278103368 0.988542265 1.9921214862
H -3.664950658 1.115601612 3.522907095
H 2.1033174 -1.7555784556 0.7321082567
H 3.1991346559 -0.3602376485 0.698285647
H 1.7901310514 0.8348707848 -0.8558156704
H -0.4974552608 0.9850385815 -0.3683746416
H 1.4779977604 -0.8113656458 -1.4158330447
H -0.7256366161 -0.7614049792 -0.540948776
H -2.7369664416 -0.6769101798 -1.6992719005
H -3.3684744859 -1.4853300575 -3.143872835
H -2.0551227236 0.099933167 -4.3187809768
H -0.9848020301 0.3738828706 -2.9425483402
H -0.4236946173 -1.4445202192 -5.1636260203
H 0.525535692 -1.2290643507 -3.6886190157
H -1.6689908087 -3.2632188032 -4.2369981887
H -0.1706396031 -3.5086128627 -3.3167828585
H -5.1314754777 -3.9267200842 -0.5228953283
H 1.0514171248 -3.458132796 -1.3216328996
H 0.1937026635 -2.9426244266 0.1406472019
H 1.7689734254 -4.4429564746 1.1527897687
H 1.8300383217 -5.4818749776 -0.2815401195
H -1.9809021675 -5.9854437274 -0.0299377348
H -0.6485183037 -6.5802042837 -1.0315430226
H 0.3341282064 -6.7557560987 1.1932025146
H -0.4777165854 -5.2685205132 1.7270525497
H -2.2577707803 -5.6599889976 -2.9859548447
H -6.0290018494 -6.4174373209 -1.2301255673
H -3.5542454993 -4.7219763199 -3.7527785584

H -6.0182436209 -5.114508112 -2.433474228
H -4.6626529118 -6.8693624862 -3.5314810965
H -3.8231697984 -7.2180704426 -2.0074920358
H -4.2864062974 -5.3582797024 0.0756872804
H -5.344741274 0.0562855784 2.0249905152

1bH^+
C -0.6147875585 -0.9427082254 -0.0365528774
N -0.3571031205 -0.3224335591 1.2793082979
C 1.0832697094 -0.3111279783 1.6143452025
C 1.7181932551 -1.1602715988 0.5011146611
C 0.7722795439 -0.9449439073 -0.6915171107
P -1.5100359404 0.2185163541 2.3374775029
N -1.0040914846 1.5982769098 3.1192908707
C -0.1593942627 1.6277975596 4.3421974164
C 0.7988715314 2.7998669679 4.0999278975
C -0.059000912 3.7765498491 3.2815867244
C -0.8348710413 2.8508265575 2.3335770773
N -2.8225798244 0.4858218304 1.4694131653
P -4.1845108174 1.2575145152 1.8631345697
C -4.7252518881 2.1305124497 0.3598673639
C -5.7074308432 3.1769734611 0.4176958581
C -6.1082886627 3.8013891663 -0.8126718202
C -5.5414322494 3.3595880486 -2.0395907941
C -4.6024668347 2.3524755654 -2.0647144075
C -4.1865802914 1.7466646188 -0.8565560168
C -7.0722359302 4.8468475853 -0.7837197773
C -7.6259847973 5.2666639691 0.4055299943
C -7.2401900166 4.6476299152 1.6193799282
C -6.3112660737 3.6282247523 1.6258417096
N -5.3849662718 0.4325433993 2.5421907054
P -6.1522153752 -0.9301286021 2.2190166667
N -7.7061870349 -0.5479920634 1.7767288083
C -8.0995789297 0.7072098598 1.0906324607
C -9.4059657808 0.3322172124 0.380838206
C -10.0247408494 -0.7161422936 1.3169511384
C -8.8082686952 -1.5347978301 1.7752779801
N -6.1573549643 -1.8773790429 3.5880715703
C -6.4581454081 -1.23355634 4.8952291985
C -7.2723742059 -2.2918711785 5.6492584606
C -6.6941901329 -3.6112158283 5.1179740925
C -6.4807629479 -3.3263565894 3.6235526775
N -5.552830144 -1.9465485147 1.0348035686
C -5.897656331 -1.6919498984 -0.3939677565
C -4.6895318874 -2.2319579449 -1.1788250809
C -4.0784069758 -3.2782654905 -0.2351942445
C -4.2321010514 -2.610364905 1.1332648998
N -1.5987394259 -0.9166860182 3.5552536751
C -2.5538218476 -0.7769565663 4.6821552225
C -2.9249187921 -2.2255488357 5.0296280198
C -1.6575997701 -3.0089282328 4.6582305849

C	-1.2005706036	-2.3318495593	3.3593131303
H	-7.6862755476	4.9750525962	2.5547429331
H	-3.4233134571	0.9750857167	-0.8744493103
H	-8.3588512588	6.0685016477	0.4161653249
H	-4.1688363852	2.0271003848	-3.0062803624
H	-7.3646832586	5.3123361682	-1.7219984106
H	-5.8593063813	3.8377960041	-2.9631665205
H	-3.4406241445	-1.870755184	1.2902681093
H	-4.2150599813	-3.3224158619	1.9624517991
H	-3.0341002717	-3.5103528328	-0.4681667333
H	-4.651850802	-4.2126006231	-0.2698466777
H	-4.9786761581	-2.6376462359	-2.1527998631
H	-3.9681723655	-1.4236726997	-1.3490371994
H	-6.0576878276	-0.6275446447	-0.5960111816
H	-6.8206754674	-2.2268712962	-0.6476945638
H	-5.6631408924	-3.9195567296	3.2044069382
H	-5.7323142392	-3.8218817097	5.6013725055
H	-7.3826903292	-3.5572109979	3.0449149744
H	-7.3301939595	1.0451233665	0.393786909
H	-8.2517444879	1.506513869	1.8249108013
H	-7.3482286792	-4.4728242905	5.2815439605
H	-10.0500982901	1.2011723629	0.2162080275
H	-9.1871437881	-0.1167296205	-0.5964557707
H	-10.7842543159	-1.3387663619	0.8348974827
H	-10.488859757	-0.2234068245	2.1800821019
H	-8.9508378099	-1.9592135128	2.7742214726
H	-8.6000255319	-2.3617754165	1.0797966445
H	-7.003126703	-0.2953001162	4.7560105568
H	-8.3339684664	-2.20976041	5.3840917492
H	-7.1881028519	-2.1873780856	6.735244194
H	-5.5215979378	-1.0017230121	5.4173877975
H	1.1747354856	3.2296721111	5.033333349
H	-0.7556875391	4.309069385	3.9406403705
H	1.6614265046	2.4659886118	3.5092589411
H	0.3511512242	0.6737700567	4.495517644
H	-0.7921767864	1.8160477292	5.2194176276
H	1.4665400234	0.718607348	1.6033596163
H	2.7519774044	-0.8677895462	0.2957289051
H	1.7187416519	-2.2179855387	0.7926119705
H	0.9657084345	0.0286056944	-1.1586261229
H	0.8681492817	-1.7161233315	-1.4616987044
H	-1.3583120901	-0.3660495862	-0.5914818109
H	-0.9961503604	-1.9685830657	0.0804448436
H	-0.2628033043	2.6532141752	1.4156384721
H	0.5274567273	4.5233805446	2.7384301228
H	-1.7055562834	-2.7766686715	2.4914462838
H	-3.4314030574	-0.1899275179	4.390979784
H	-1.8322727104	-4.080869238	4.5234319503
H	-3.7674968895	-2.5417644836	4.405192862
H	-3.2135681289	-2.3412857688	6.0789024965
H	-0.8931161736	-2.8860875951	5.4350756286

H -0.1217589785 -2.4162704422 3.19840046
H 1.273291487 -0.725115663 2.6121325426
H -1.8034757084 3.2649385957 2.0377943486
H -3.9087230028 2.2765539662 2.7955824733
H -6.0619690545 3.1496675187 2.5669996132
H -2.0663528709 -0.2731467817 5.5270898857

2a
C -0.2772417643 0.2509298942 0.0191603054
C -0.076457675 -0.0386269642 1.3750773885
C 1.2163903098 -0.3866708817 1.8272314637
C 2.3034168448 -0.3996352254 0.9593064275
C 2.0997726378 -0.0876959311 -0.391260854
C 0.8145441419 0.2215956651 -0.8537736182
C 1.1749595853 -0.753641929 3.2923992065
C -0.2929208924 -0.6107850582 3.6294573157
C -1.0180578099 -0.1334744995 2.5120737405
C -2.4014654213 0.1354871371 2.6207494415
C -3.0228591771 -0.2117548184 3.8319701935
C -2.3092691289 -0.7280186756 4.9195672644
C -0.9246021 -0.9069385307 4.8326704741
P -3.3911295846 0.8753906769 1.2040455443
N -4.8576578791 1.1292169191 2.0624175358
P -6.2991552996 1.1184508693 1.4504428931
N -6.6766572059 -0.2870014867 0.5768083399
C -6.1122854545 -1.5658762423 1.0051808486
N -2.6466908804 2.3881714893 1.012350038
P -2.3681542212 3.6075714852 1.9609560053
N -0.7654288547 4.1148353201 1.7636169082
C -0.2992366724 5.3579418373 2.3656521748
N -2.5227992486 3.5511446498 3.641422129
C -3.8470072144 3.3926225386 4.2463669804
N -3.4373509481 4.8945220985 1.5720454083
C -3.533519148 6.1085361207 2.380773242
C -1.4482955915 3.0087282857 4.4776593722
N -7.3983974488 1.3945313519 2.7173621216
C -7.0333957706 1.0232746212 4.0820947181
N -6.7650831998 2.3228097431 0.3356385753
C -6.0393577724 2.2973065691 -0.9455260533
C -8.8242531207 1.2427310384 2.4378063082
C -6.8624961939 3.6881927034 0.8773661412
C -7.8540915283 -0.4368175716 -0.2731753593
C -3.5658644102 5.184378463 0.1425698419
C 0.044883385 3.666586373 0.635727522
H -0.3565669003 -1.2846099146 5.6805510983
H -4.0916588301 -0.0455578872 3.9177133881
H -3.5132824111 4.257608051 -0.4290690839
H -4.6306802121 3.6770425444 3.5437721588
H -4.5335452276 5.6656484976 -0.0498097654
H -4.0201691403 2.348987383 4.5318925999
H -3.9162581832 4.025567075 5.1433082879

H -3.368726802 5.886639633 3.4356132587
H -2.8093568395 6.8792581081 2.0686227357
H -4.5415338835 6.532007599 2.2699855997
H -1.4080537141 3.57603613 5.4184180447
H -1.6163427431 1.9511739846 4.7117151732
H -0.4875617993 3.1012573936 3.9701531252
H -0.876348268 5.5951180863 3.2618766248
H -0.3679985086 6.2077626849 1.6664878485
H 0.7538745527 5.2486197734 2.6596515434
H 1.0599138357 3.4221175034 0.9780015539
H 0.1174269076 4.4492221315 -0.137589439
H -5.8830098584 4.0862985616 1.1785721338
H -7.5264793687 3.702410561 1.7434193953
H -7.2870244907 4.3362857022 0.1012542522
H -5.0115609384 2.6727683709 -0.8456691036
H -6.577700362 2.9245000101 -1.6665034897
H -5.989240893 1.2798121179 -1.3369426971
H -8.2403265591 0.5391765006 -0.569382044
H -7.5821781895 -0.9935669786 -1.1814681593
H -8.6566275272 -0.9937891291 0.2368493859
H -5.8032380884 -2.1464153214 0.1258198626
H -9.1553682891 0.1922270886 2.4972480417
H -6.8500388607 -2.1595939844 1.5702205154
H -9.3987106012 1.8211199114 3.172689983
H -5.235526972 -1.4083272119 1.6336130681
H -7.2399634056 -0.0390722736 4.2977005152
H -7.6156813913 1.6334548304 4.78519193
H -5.9732249315 1.2154560985 4.2396421428
H -9.0604259687 1.6280317477 1.4433407903
H -2.833444794 -0.9732269145 5.8410853557
H 2.9386047669 -0.098319896 -1.0831162864
H -0.3970611151 2.775157347 0.1986230591
H -2.7713779565 5.8649991138 -0.2105352972
H 3.295908412 -0.6627850489 1.3201523089
H 1.797027117 -0.0844803968 3.9046491531
H 1.5437408321 -1.7737539644 3.4682366092
H -1.2647051499 0.4996841079 -0.3520156104
H 0.6607041529 0.4473911302 -1.9064379249

2aH^+
C -0.1264648712 0.3451498672 0.0332367482
C 0.0296764296 -0.0852205657 1.3583184418
C 1.2926094567 -0.5526757125 1.7895596903
C 2.3891693833 -0.558232078 0.9335025478
C 2.2267427029 -0.1116791955 -0.3825701078
C 0.9734731649 0.3271157293 -0.8275227651
C 1.2162831696 -1.0411075926 3.2148276937
C -0.2371616728 -0.8254932742 3.5621895412
C -0.9240479742 -0.2244552982 2.479437983
C -2.29555003 0.0838999 2.6261182887
C -2.9468202229 -0.2933116325 3.8145516865

C -2.2587755684 -0.9092241143 4.8614937486
C -0.887596421 -1.1601025249 4.74393379
P -3.2398599009 1.0430449036 1.4046015638
N -4.770112882 1.0288917475 1.9193058119
P -6.1531786872 1.006360144 1.1363254953
N -6.4765633659 2.2299127063 0.0277004133
C -5.6500620553 2.2716338405 -1.1912895482
N -2.5501277643 2.470795054 1.0957831919
P -2.2974275341 3.6933120324 2.0972041471
N -3.3684094953 4.9395825286 1.6794553101
C -3.5007045478 6.1392403646 2.5189286407
N -0.6959666728 4.148976425 1.9740464842
C -0.2264192455 5.3535580623 2.6650651505
N -2.5569573712 3.5031926955 3.7362753483
C -3.914520597 3.3753270949 4.2794088129
C 0.185757466 3.7069293084 0.8911482796
N -6.2711844369 -0.3817546532 0.1888944824
C -5.7805650193 -1.6534698362 0.7400374724
N -7.3549681116 1.1813891774 2.2869198442
C -7.1236498152 0.9018713324 3.7058870336
C -7.368045582 -0.5773502992 -0.7707292668
C -8.7556642522 1.0443079444 1.8725256018
C -6.742810845 3.5740812515 0.5703945592
C -1.5209384646 2.9654670604 4.6283825738
C -3.4258853377 5.2744304255 0.2482758661
H -0.3404022754 -1.6261489242 5.5598357314
H -4.0071466303 -0.0887250298 3.9130358383
H 0.8527741344 0.6599750257 -1.8550124141
H -3.3588423701 4.3688946479 -0.3544205162
H -4.6569016391 3.6938568166 3.5470825109
H -4.3789737367 5.7704825541 0.0338161679
H -4.1225597484 2.3330441357 4.548261507
H -4.0091352933 3.9964387733 5.1793130452
H -3.3956299942 5.891343922 3.5752781935
H -2.7553743892 6.9061826187 2.2618321637
H -4.4976881659 6.56751595 2.3622413111
H -1.5226147956 3.5377728377 5.5643925823
H -1.710521074 1.9103519518 4.8573771613
H -0.5372197857 3.0484946996 4.1665578259
H -0.8617377021 5.5848210084 3.5222588979
H -0.2074224998 6.2227011019 1.9920824896
H 0.792113051 5.18321383 3.0342137105
H 1.1583562777 3.4138419116 1.3051147997
H 0.3429632432 4.5152715893 0.1626929036
H -5.8290642614 4.0485375375 0.954132853
H -7.4756116154 3.519403852 1.3761910036
H -7.1559772238 4.192509422 -0.2331812265
H -4.6648540879 2.7185420037 -0.9989252396
H -6.1665842536 2.8743845234 -1.9457004675
H -5.5032610179 1.2682176139 -1.5931388505
H -7.727535351 0.3800666835 -1.1487005412

H -6.9988368215 -1.1699179084 -1.6160004799
H -8.2108965512 -1.1164334664 -0.3148717328
H -5.4174009352 -2.2815180902 -0.0813582987
H -9.1122831799 0.0088131428 1.9732924092
H -6.5788279531 -2.1994653613 1.2650117578
H -9.3807418574 1.6862566056 2.503882437
H -4.9590872882 -1.4856374214 1.4375296957
H -7.3660795945 -0.1403140842 3.9618781676
H -7.762171333 1.561918795 4.3050635406
H -6.0826096495 1.0988403737 3.9571808077
H -8.882433337 1.3633087403 0.8359823899
H -2.7904017788 -1.1868640421 5.7673568555
H 3.0723296751 -0.1163590439 -1.0647783291
H -0.2449493988 2.848247902 0.3843999956
H -2.6116296409 5.9564643211 -0.0425672384
H 3.3560729275 -0.9174118707 1.2774674767
H 1.5070126348 -2.096651426 3.3019797038
H 1.8797660737 -0.4768894531 3.8850743724
H -3.1317128837 0.3480470264 0.185840164
H -1.0764585975 0.7063124976 -0.3396225026

2b
C -0.2051287639 -0.6400976671 0.0329454365
N -0.1287281368 -0.2179488976 1.4430362175
C 1.2588943098 -0.1307654891 1.918779683
C 2.058075739 -0.8618915582 0.8297632146
C 1.2534087689 -0.55651981 -0.4440802826
P -1.4095854597 0.40187392 2.3320394029
N -1.4035382608 -0.5723969686 3.7156778202
C -2.4247719045 -0.3042086621 4.7610995538
C -2.933649352 -1.6950359898 5.1698624589
C -1.736639073 -2.6089965742 4.8742636479
C -1.1932048384 -2.0292884226 3.5629315525
N -2.6794216772 0.3974509229 1.4133227027
P -4.0170941679 1.4591665523 1.471921088
N -5.3095317496 0.6681426298 2.2174128212
P -6.0882965102 -0.6892249981 2.1931568586
N -6.0821605264 -1.4069587821 3.7193981378
C -6.3369470443 -0.5528733258 4.8999651114
C -7.1342356815 -1.4580667151 5.8495345176
C -6.5823412968 -2.8560881331 5.5306968243
C -6.3942579305 -2.8220110583 4.0043289387
C -4.5088664981 1.3560663506 -0.3428933733
C -5.4813659953 2.2142082895 -0.9042037151
C -5.8172359025 2.1167256223 -2.2761746694
C -5.1990351011 1.1920705053 -3.1103135044
C -4.2196647666 0.3534195018 -2.5661554288
C -3.8847560473 0.4463280718 -1.211805503
C -6.3136672075 3.2872134834 -0.3143810755
C -7.1420727132 3.8247798404 -1.3247905255
C -6.8841427282 3.1258014362 -2.6393134298

C -8.0456969773 4.8457682791 -1.0479060453
C -8.1278678668 5.3459840685 0.2580054938
C -7.3105443403 4.8166520498 1.2649712585
C -6.4057884599 3.7859356458 0.9920815572
N -0.9712161911 1.8873799273 2.9855058553
C -0.1889438491 2.1041191566 4.2211647273
C 0.6575132473 3.347324245 3.9131348641
C -0.2466316352 4.1454896219 2.9611749372
C -0.865234876 3.0533106067 2.0783869991
N -5.6308640453 -1.9951302308 1.2191878559
C -6.1344034854 -2.0895222593 -0.1750324685
C -5.1110289371 -2.9919716443 -0.8999029849
C -4.3241945862 -3.6739456301 0.2328957208
C -4.2796774012 -2.5818856996 1.3035594239
N -7.695978338 -0.4142307627 1.7923678851
C -8.1258092882 0.664999155 0.87784147
C -9.5002638806 0.1941771358 0.3850629575
C -10.0444684046 -0.6003644708 1.58186779
C -8.7983440152 -1.3362441124 2.1003636389
H 1.3736117589 -0.5918040479 2.9082425612
H 1.4653302991 -1.2467789083 -1.267408567
H 1.4747144172 0.4613207643 -0.7908413125
H 1.5814844336 0.9192468699 2.0066699769
H 2.054749153 -1.9419260151 1.0267161056
H 3.1005843257 -0.5300355257 0.7801232367
H -0.5886310925 -1.6694918777 -0.0434187025
H -0.8776509672 0.0075754157 -0.5338675282
H 0.9356759681 3.8985596105 4.8180292647
H -0.2067438821 2.8235695414 1.2243414681
H 0.2906919172 4.8994311681 2.3763883434
H -1.0347651314 4.6553461751 3.5297995339
H -1.8494353111 3.3254961886 1.6868902873
H -3.7169660882 -0.3764840203 -3.1982143059
H -3.1243460057 -0.2066984987 -0.7951685642
H -0.1323009871 -2.2521972571 3.4069850109
H -1.749574672 -2.4343159837 2.7055142707
H -0.9814770512 -2.5224299332 5.666495355
H -2.008709413 -3.6660227538 4.7785763151
H -3.2336268583 0.3270522299 4.3752578118
H -1.9580577667 0.2163552479 5.6084044012
H -3.5038037924 -1.8347040707 1.0887995607
H -4.4365802667 -2.3690798937 -1.4965647448
H -4.8680721515 -4.5495814254 0.6105874792
H -3.3244941003 -3.9990185822 -0.0768568101
H -5.5955302964 -3.7033041648 -1.5772238268
H -4.0971235587 -2.9757886598 2.3087923779
H -5.3881413996 -0.2386908546 5.3551133207
H -5.580425052 -3.478957548 3.6781103499
H -7.3023248033 -3.1583759293 3.4877445577
H -5.6132175377 -2.9961607068 6.0252362728
H -7.2423710538 -3.6707092888 5.8474569529

H -8.2037861576 -1.4102731209 5.6068101763
H -6.879030532 0.3547449677 4.617304431
H -8.8575726593 -1.5340765995 3.1764725907
H -8.6737479148 -2.3059066475 1.5898296939
H -10.8569642062 -1.2874450085 1.3218503536
H -10.4159722542 0.0890469235 2.3509231901
H -10.1428005777 1.0294312194 0.0876142478
H -9.3819299251 -0.469030601 -0.4822283342
H -8.1922993738 1.6193882793 1.4130951634
H -7.4179180152 0.8011407659 0.058713746
H 0.4110322231 1.2257948733 4.4761453821
H -0.8663189744 2.3001596206 5.0652179149
H -3.2625733857 -1.7308561249 6.2141426079
H -7.0153132477 -1.1740522955 6.900775744
H -6.1946486136 -1.1105037882 -0.6639183248
H -7.1397072181 -2.5288200218 -0.1710430449
H -3.7824963782 -1.9651590195 4.5348120512
H 1.5818340735 3.0552528705 3.3967970903
H -8.8291233543 6.1440444321 0.4902617477
H -7.3838819732 5.205377624 2.2780594086
H -8.6807788283 5.2501181133 -1.8339375815
H -5.4652222481 1.1282331064 -4.1636422201
H -6.5404331384 3.8237624445 -3.4160004468
H -7.7903370276 2.6417447676 -3.0311530189
H -5.795186597 3.3710662372 1.7853412933

2bH^+
C -0.6637662712 -0.0749057629 -0.1988261163
N -0.3340988816 -0.0554500212 1.2420854379
C 1.1271490865 -0.0264370421 1.4726352906
C 1.6926684154 -0.4916704569 0.1258292776
C 0.7009570285 0.1011032298 -0.8876421487
P -1.4950051689 -0.0802523007 2.4247801727
N -2.1988949308 1.4337017737 2.4055107373
C -1.4821017573 2.6483865881 1.947739024
C -2.4110161822 3.7925671373 2.3805190754
C -3.050838035 3.2581091146 3.6718854384
C -3.288183254 1.776507883 3.3499521825
N -0.7850748001 -0.5604429271 3.7480544049
P -0.8288898213 -0.8251136715 5.3180187863
C -0.0942044002 0.6176901535 6.170630855
C 0.3287520416 0.6215081762 7.5181894937
C 0.7911547486 1.8305375749 8.0942376601
C 0.8411655459 3.0137652704 7.3671826078
C 0.4426294424 3.0027410681 6.0265929064
C -0.0079897955 1.8172004723 5.4443689994
C 1.1975832681 1.6147323305 9.5325876786
C 0.9000921532 0.1508388467 9.7446331102
C 0.4076904036 -0.4296962596 8.5529164023
C 1.072507536 -0.6087790812 10.8968430888
C 0.7625996093 -1.9734163608 10.8645829055

C	0.2916533257	-2.5602995638	9.6838044928
C	0.1098791106	-1.7981246196	8.5263954165
N	-2.1973529439	-1.3177336859	5.9941957314
P	-3.4892309503	-0.8443365017	6.7895646367
N	-4.6931626242	-0.3397609456	5.7476190353
C	-5.01650775	-1.218102293	4.5910744555
C	-6.5042085996	-0.9513814274	4.3114607551
C	-6.7191798325	0.4718994631	4.8473940044
C	-5.8605127034	0.4950000706	6.1176598796
N	-4.0269522103	-2.1760530303	7.6219018817
C	-3.1683346021	-3.287610815	8.09185091
C	-4.0765550045	-4.0550090043	9.0634054414
C	-5.4846434449	-3.8182328115	8.4987621513
C	-5.4319224054	-2.3535754073	8.0413299556
N	-3.3671987729	0.4004490371	7.8875162685
C	-3.0689213595	1.8095136252	7.5527259965
C	-3.2957162558	2.5225179653	8.8878996744
C	-2.7190611728	1.515613333	9.8961875403
C	-3.1227537662	0.1395472619	9.3335005935
N	-2.7593274544	-1.0807631139	2.0189225604
C	-3.8174777286	-0.7594337198	1.031036781
C	-4.1659724571	-2.1209022674	0.415145372
C	-3.9290134732	-3.0957992071	1.5791838776
C	-2.660103269	-2.5448969574	2.2444258611
H	0.6408790393	2.2637867497	10.222254367
H	1.1962764218	3.9309058798	7.8309974606
H	2.2621599424	1.8381355761	9.6880268998
H	1.4496885463	-0.1515848723	11.8084476178
H	0.8956352825	-2.5807230291	11.7555840057
H	0.0639209451	-3.622959331	9.6644063703
H	-0.3063474958	1.8165777393	4.4038574506
H	0.4889046451	3.912499606	5.4339711857
H	-6.115698603	-2.1574274519	7.2099380953
H	-7.1307460875	-1.6593739701	4.8676654515
H	-6.4118567992	0.0780241742	6.9709349695
H	-6.2819089371	-3.9974811281	9.226171898
H	-5.6939892381	-1.6706448716	8.8638743494
H	-2.0324711196	1.9347589762	7.2250461017
H	-3.9975348476	-3.6225226519	10.0687541149
H	-3.8096478772	-5.1139827375	9.1307022358
H	-2.2703029086	-2.9128658246	8.5850557094
H	-2.8578084815	-3.9086577756	7.2417265194
H	-2.3501933387	3.3505778419	4.5105422779
H	-3.7188838543	2.1689023394	6.7503937855
H	-2.802743044	3.4988170423	8.9256612098
H	-4.3690127599	2.670979892	9.0597429151
H	-3.0850290144	1.664414291	10.9162933884
H	-1.6275816695	1.5959684556	9.9157262935
H	-4.0411737457	-0.2368150246	9.7984171355
H	-2.33208478	-0.6014197532	9.4878373047
H	-5.549061125	1.5056234877	6.3905410863

H -7.7680670821 0.705568049 5.053028617
H -6.3422132076 1.210048803 4.1287584547
H -6.7499428881 -1.0555776531 3.249878801
H -4.8220405478 -2.270327082 4.8302734174
H -4.3820738893 -0.9500207182 3.744376619
H 0.0860235198 -1.8667302464 5.5397305995
H -4.2635498064 1.6281241909 2.8665980401
H -3.4772030923 -0.0296737008 0.2926824808
H -4.6890182685 -0.3276796902 1.5434268821
H -5.1867319742 -2.1509442759 0.0217789605
H -3.478877053 -2.3457915916 -0.4103167717
H -1.3638663096 0.7264491047 -0.4677450788
H -2.6082266405 -2.7600496884 3.3157311567
H -3.8100328079 -4.1348847123 1.2586083641
H 1.3936233061 -0.6757879886 2.3088754528
H 1.4648450139 0.9938866528 1.7092346182
H 1.680043893 -1.5875640946 0.075036415
H 0.9094644448 1.1681147165 -1.0342635763
H 0.7343438591 -0.3844878718 -1.8673731053
H -1.7566849478 -2.9523575223 1.7688938858
H -1.1314579463 -1.0318841133 -0.4689526241
H -3.1820164952 3.9575111486 1.618007862
H -3.9742816631 3.7791912246 3.9438418395
H -1.869210409 4.7329017906 2.5198658226
H -0.4963084553 2.7434958124 2.4249073961
H -1.3226139272 2.6216753195 0.8655827134
H -4.7708486293 -3.0548050288 2.281691999
H -3.2643561322 1.1426840604 4.2417392393
H 2.7216700085 -0.1554807015 -0.032953301
H -0.270390401 -2.2759489194 7.6331383835
H -5.6604043454 -4.4712090018 7.6348470487

3a
C 0.0197164816 0.0700756707 -0.0665129948
C 0.0013326183 0.0813629829 1.3310545734
C 1.1829362505 0.0249137579 2.1156397897
C 2.4402328685 0.0711213926 1.4520412347
C 2.4396337612 0.0183463243 0.0534895291
C 1.2565096646 0.0056326611 -0.700208487
C 0.8573814909 -0.086828713 3.540078175
C -0.5371057809 -0.0089813 3.7814848181
S -1.4755317672 0.1435962108 2.2978174827
C -1.0783310477 -0.0965331715 5.0680748926
C -0.2160573418 -0.3059362658 6.142406058
C 1.1647444417 -0.4375863307 5.9234221634
C 1.6987284635 -0.3269065876 4.6426492672
P 4.0750871653 0.1847291948 2.3883887059
N 3.8986641532 1.6562444238 3.2090974639
P 3.5871921849 3.1346667026 2.7811408338
N 5.0143227874 4.0802821588 2.9221714722
C 5.7642740473 3.8909031105 4.1656550571

N	5.0885874754	0.4687393873	1.0340065876
P	6.5997131852	0.0712553312	0.9168386434
N	7.757458796	0.8003239153	1.9340948691
C	7.9858013066	2.2382401406	1.7154697663
N	6.9119933117	-1.5716371112	1.2037916644
C	8.2462219682	-2.1378914597	1.3791322898
N	7.1429883685	0.5655187434	-0.6155164236
C	8.4727028086	0.1387751941	-1.0453903574
C	5.9108418729	-2.567883333	0.8261225932
N	2.3775247277	3.7650820199	3.7803482552
C	1.9855779802	3.1051826544	5.0216184948
N	3.0536298352	3.5768471727	1.2424607234
C	1.6379720335	3.4777392746	0.8760111244
C	2.0160857097	5.1763675042	3.7243771435
C	3.9488353734	3.4329570939	0.0926223422
C	5.0789012531	5.4657813312	2.4590677443
C	6.1889436379	0.6817784363	-1.7156734907
C	7.6182682545	0.4832527333	3.3653350929
H	-0.9054935011	0.0970849171	-0.6358885138
H	3.3994131104	0.0214185437	-0.4516135424
H	5.7129237833	2.8464267657	4.4734400815
H	4.9895469044	3.3897033091	0.4149323931
H	6.8156483157	4.1628204591	4.005497564
H	3.7315047898	2.5084011919	-0.4541693141
H	3.8141817602	4.2898979903	-0.5834858493
H	4.4668007197	5.6056622349	1.5673934847
H	4.7449246366	6.1817056404	3.2283739304
H	6.1200983055	5.7084513143	2.2053818414
H	1.3782131187	4.3215839684	0.2211156547
H	1.4271030311	2.5433132983	0.3442944528
H	1.0079241867	3.5164157365	1.7649940312
H	2.2461542814	5.5978523435	2.7437732059
H	2.5404296769	5.7665429842	4.4937965137
H	0.9358822205	5.2841583707	3.8947847834
H	0.8902127808	3.0684356018	5.0996284788
H	2.3797629343	3.6467223599	5.8971188554
H	7.1101645673	2.8441301845	1.9886438898
H	8.2266217922	2.4251093627	0.6675574227
H	8.83917464	2.5480022648	2.3305972284
H	6.7711887579	1.0113266125	3.8245360469
H	8.5407253861	0.776266788	3.8812145828
H	7.4641908895	-0.5878240061	3.5069879012
H	8.9576107689	-1.3627416624	1.6664723787
H	8.2206757557	-2.8978406394	2.1732835013
H	8.6065356812	-2.6236223057	0.4580209732
H	5.8310898573	-3.3289531849	1.6137095965
H	8.4711126718	-0.8791307954	-1.4701869287
H	6.1837572793	-3.071428257	-0.1160141464
H	8.8395223838	0.8275231246	-1.8170521259
H	4.9326736148	-2.1027123849	0.702989458
H	6.0102673399	-0.2831967772	-2.2202239599

H 6.5860758336 1.3863953944 -2.4581602144
H 5.2417511932 1.0629195142 -1.3370597486
H 9.1713878559 0.1664238154 -0.2061878827
H 1.3070619574 -0.0266283976 -1.7864399282
H -0.6189115134 -0.3829870214 7.1491874285
H 2.367680566 2.0878726905 5.0324620048
H 5.3702421388 4.5212173712 4.9820639146
H -2.1515467903 -0.0214113638 5.2224110743
H 2.7675631979 -0.4233023072 4.4939701979
H 1.8295818405 -0.622312192 6.7633232571

3aH⁺

C 0.0797618219 0.1117014913 -0.1044206091
C 0.0363115969 0.1486178382 1.2927808348
C 1.2013900518 0.0581176905 2.1023873724
C 2.457361783 0.0189101988 1.4346305473
C 2.4867941203 -0.0494239737 0.0360442046
C 1.313903164 -0.0176071122 -0.729886902
C 0.8490649923 0.002479363 3.5215377661
C -0.5483014548 0.1428811332 3.7239786897
S -1.4495633069 0.2885597193 2.2210408602
C -1.1263856645 0.1111440752 4.9975965143
C -0.3027879169 -0.0998224385 6.0991575253
C 1.0783470412 -0.2860979569 5.9239613871
C 1.6484857772 -0.2337140148 4.6570915385
P 4.0612807481 0.2312501436 2.2792849682
N 4.0715602088 1.5474815864 3.2124122898
P 3.8653011463 3.0749151209 2.7766391878
N 3.3691234535 3.4420114866 1.2258453563
C 1.9495246874 3.4777708165 0.849297629
N 5.1814255449 0.1031646437 1.1258102858
P 6.6551502895 -0.4929406318 1.1296680451
N 7.3131913853 -0.142036834 -0.3666459807
C 8.616075735 -0.7170047094 -0.7185903492
N 7.7741858463 0.1272105093 2.2215687972
C 8.276679325 1.489204677 1.967619628
N 6.6281317869 -2.1294218505 1.5279436334
C 7.8611775118 -2.889727566 1.7818611895
C 7.530939978 -0.1239537746 3.6527779233
N 5.366108184 3.8548619244 2.8974344774
C 6.1020120475 3.6231667177 4.1500743475
N 2.7014069857 3.7469144037 3.7652485738
C 2.2462734978 3.1244420165 5.0107431065
C 5.5285066692 5.2392155879 2.428128813
C 2.3841651881 5.1748526539 3.6673357463
C 4.2713286213 3.2755710468 0.0805234894
C 5.5624540345 -2.9705867479 0.963376096
C 6.4900163819 0.2278815963 -1.5199468827
H -0.8362313321 0.1694237998 -0.6856353132
H 3.4492618601 -0.1070132169 -0.4589689706
H 1.7111502468 -0.47218025 6.7871572897

H 5.9991108991 2.5839549939 4.4615180908
H 5.3025950564 3.1579258509 0.4144152154
H 7.1635688333 3.8388537954 3.9865126695
H 3.9924384458 2.3868418606 -0.498102194
H 4.20341491 4.1555376421 -0.5718609602
H 4.941417523 5.4182397527 1.527086122
H 5.231813725 5.9718212729 3.193202411
H 6.5854094778 5.4051525302 2.1895960643
H 1.7625236011 4.3657276619 0.2324807332
H 1.6775205761 2.5850849005 0.2756506288
H 1.3166278012 3.5243444824 1.7352581803
H 2.666504594 5.5725962501 2.6907849851
H 2.8971986119 5.7539552448 4.4481009369
H 1.3032053881 5.3136683566 3.7908485452
H 1.1498251328 3.1161366149 5.0433170166
H 2.6213671362 3.6836839995 5.8794282234
H 7.5153721025 2.2527023692 2.1787998638
H 8.5952528423 1.5884189276 0.9293593946
H 9.1431462781 1.6609607771 2.614496379
H 6.7710261105 0.5566590384 4.0614490412
H 8.4669636089 0.0271953359 4.2006414175
H 7.1995293744 -1.1507479474 3.813670448
H 8.6420005713 -2.240321115 2.1784197848
H 7.6497282614 -3.6724493719 2.5197144686
H 8.2331891711 -3.3713778048 0.866195005
H 5.3382621156 -3.7812135333 1.6658743659
H 8.5103453425 -1.6780737647 -1.2425681128
H 5.8673853265 -3.4185974572 0.0056676465
H 9.1489329264 -0.0222327249 -1.3779608661
H 4.6525730582 -2.3913999327 0.8011575898
H 6.218893894 -0.6492178407 -2.1262269409
H 7.0571973058 0.921923405 -2.1516436709
H 5.5816000511 0.7245827711 -1.1823667468
H 9.2241071573 -0.8663965125 0.1760577214
H 1.3729130633 -0.0741683497 -1.8129784096
H -0.7347547547 -0.1379394765 7.0951009594
H 2.5995207888 2.0988276289 5.0674737158
H 5.7410185292 4.2750946976 4.9610226734
H -2.1992565074 0.2283090452 5.1203280658
H 4.1897660413 -0.8343893192 3.1885448068
H 2.7172004776 -0.3684216895 4.5658882042

3b

C -0.1490742 0.3043948291 0.0731378161
N 0.0966875448 0.1040643212 1.5210862288
C 1.5147468775 -0.1884945438 1.7831402375
C 1.9777908958 -0.7924740225 0.4525347384
C 1.2255973045 0.0605396149 -0.5816343614
P -1.0479250093 0.6637669683 2.615189133
N -0.9179161284 2.3337936151 2.6214242971
C -2.0250651665 3.2324299176 3.0238964896

C	-1.3031375365	4.51480626	3.4624729164
C	-0.0494412423	4.5308186687	2.5730810821
C	0.3646004015	3.0516168564	2.5394158748
N	-2.5125025658	0.14473688	2.4195114223
P	-3.6296374466	0.5573170219	1.2002901966
N	-4.2025613444	-0.9069170183	0.5899384755
P	-3.7305592074	-1.9913591052	-0.417113682
N	-2.6255863572	-1.4986607705	-1.5944463645
C	-1.6797723232	-2.3676195978	-2.3252757615
C	-1.5548042228	-1.7063099701	-3.7062330792
C	-1.7819902758	-0.2176215644	-3.3986349107
C	-2.8936972965	-0.2524953228	-2.34167132
C	-5.1383871867	0.9505639098	2.2423523809
C	-6.4011704426	1.2684979439	1.669579739
C	-7.4940125331	1.5105103809	2.5426536076
C	-7.3712470576	1.4921622628	3.9343460433
C	-6.1204668853	1.2122892725	4.4748562339
C	-5.0299796311	0.9428582062	3.6355458525
S	-9.0129547008	1.8206778147	1.6950257884
C	-8.2054557732	1.6569329626	0.139461868
C	-6.8198014939	1.3841195045	0.2683945651
C	-8.8303389778	1.7864712044	-1.1054829961
C	-8.059700436	1.659394491	-2.2591937497
C	-6.6803089135	1.4168202652	-2.1594936289
C	-6.0670702055	1.2818134302	-0.9172482846
H	-5.0047984287	1.0947598065	-0.860976537
N	-0.3866207241	0.1254594398	4.0846053035
C	-0.5246514154	-1.3042814581	4.419025823
C	0.0998828656	-1.3967637476	5.8232927703
C	-0.1017452059	0.0206038113	6.4337430105
C	-0.7738027112	0.8427617743	5.3126705955
N	-5.0771959134	-2.6205873093	-1.1828337264
C	-6.4711742543	-2.3680327328	-0.7809417121
C	-7.2386950647	-2.5739409069	-2.09040001
C	-6.4683049478	-3.7282783095	-2.7549512975
C	-4.9950367454	-3.4307097769	-2.4056002446
N	-2.9316358761	-3.3526359259	0.1990897415
C	-3.7658523063	-4.3808147378	0.8664761026
C	-2.7709352469	-5.1208237645	1.767539241
C	-1.8550118602	-3.9837482445	2.2425676158
C	-1.6882291341	-3.120793674	0.9824178454
H	-8.5293685403	1.7554263002	-3.2350134756
H	-6.0789793134	1.3301397792	-3.0614298766
H	-4.4146464591	-4.3519049329	-2.2495580238
H	-8.2978582456	-2.8004476162	-1.928501344
H	-7.1729788286	-1.6625186676	-2.6978077836
H	-4.4998635035	-2.8646470586	-3.2088667912
H	-6.7717850811	-4.6829897453	-2.3065067732
H	-6.6311700775	-3.8010763368	-3.8356492805
H	-6.7978547003	-3.0925302047	-0.0168065845
H	-6.5717151759	-1.3663072414	-0.363258779

H -0.5902871419 -1.9116124437 -4.1833675642
H -3.8869437736 -0.2842658108 -2.8179823948
H -2.0591748191 0.3732741464 -4.2777756578
H -0.8725766855 0.2219438702 -2.9693467284
H -2.8690982143 0.6128592399 -1.6711642897
H -5.992420245 1.1853796957 5.5550744017
H -4.0669400653 0.685206794 4.0661609982
H -4.2396334106 -5.030801103 0.1227580798
H -4.5601641446 -3.9235403187 1.475790566
H -2.2001872935 -5.8531761024 1.181155452
H -3.26811809 -5.6523380653 2.5861263544
H -1.5639547969 -2.0649582257 1.2377454937
H -0.8166960907 -3.4394793354 0.3924505759
H -1.5871770563 -1.5917356636 4.4356379253
H -0.7227159782 0.0038164922 7.3353742003
H 1.1669012196 -1.6332651895 5.7505440435
H -0.3727656672 -2.1845320866 6.4192372946
H 0.8618689628 0.4631053085 6.706856375
H -0.0242696358 -1.9297996438 3.674023536
H -0.9028621202 -0.4043688479 -0.2826416329
H 1.6242562361 -0.8730818018 2.6271820758
H 2.0920290287 0.7205888234 2.0189969014
H 1.6526144277 -1.8389498351 0.3904024073
H 3.0659268391 -0.7623985152 0.331262408
H 1.7513521166 1.0123343691 -0.7302261637
H -0.5277061459 1.3142873211 -0.132982052
H 0.9018336337 2.7863104504 1.6216560136
H 1.0190646599 2.8059144721 3.3918372667
H 0.747535154 5.1797696811 2.9511741186
H -0.3116663506 4.8673205819 1.5620410805
H -1.9291880796 5.4055830836 3.3477188403
H -1.0110830028 4.4411368239 4.518651407
H -2.690789601 3.416364214 2.1709316832
H -2.6315369217 2.7927560168 3.8211576559
H -2.034998303 -3.4005717535 -2.3741644787
H -0.7064795862 -2.3800372592 -1.8112033318
H -0.8972391214 -4.3310403375 2.6460169131
H 1.1360860491 -0.4245839691 -1.5591193397
H -1.8694423882 0.8447586917 5.4434797415
H -0.4341301308 1.8814704362 5.2733868089
H -2.360669366 -3.3974284835 3.0185583501
H -2.345843084 -2.0751497093 -4.3722267949
H -8.2298166928 1.6880819157 4.5712173131
H -9.8967237478 1.9869503991 -1.1686809964

3bH^+

C -0.2159546431 0.4294498985 0.0929254512
N 0.0382499428 0.2239004697 1.5413736741
C 1.4876884981 0.1133238072 1.8155115062
C 2.0347791093 -0.3855191361 0.4747787236
C 1.1864648208 0.3890949828 -0.5464683309

P -1.146653182 0.6261922396 2.6334801499
N -1.2900313716 2.2827180019 2.6560376013
C -2.4808502369 3.0085342645 3.1753038775
C -1.9065253428 4.3461532134 3.6614322353
C -0.7093228326 4.5756862794 2.72602785
C -0.1052396854 3.1716565047 2.5925333274
N -2.5386763106 -0.07812033 2.3218679083
P -3.7574651955 0.2113664888 1.3088803515
N -4.226769828 -0.9885339168 0.3692474547
P -3.6274177607 -2.1388362659 -0.5422301345
N -2.4724114744 -1.5685505925 -1.6083299292
C -1.4812511031 -2.4121926149 -2.3233767114
C -1.4382728462 -1.8192749553 -3.7370530611
C -1.7128289487 -0.3281439064 -3.4894683362
C -2.7888421519 -0.3500953164 -2.3945797608
C -5.2132915692 0.753746155 2.2521493148
C -6.3674352245 1.3406809543 1.6645315511
C -7.399168051 1.762007127 2.5477049279
C -7.3320198209 1.5844199289 3.9326907519
C -6.2042436703 0.9800924381 4.4751954171
C -5.1536988004 0.5818137889 3.6390871888
S -8.7538315369 2.525563232 1.7280238112
C -7.9760164988 2.2835560857 0.1708055419
C -6.7187792978 1.632522908 0.2715555872
C -8.5234647744 2.669986974 -1.0573848632
C -7.8111275971 2.4019769843 -2.2221014742
C -6.5719961318 1.7447016857 -2.1539838476
C -6.03285586 1.3619085394 -0.9303661696
H -5.1024244708 0.8148356563 -0.9205247353
N -0.511765348 0.1173701747 4.0952188424
C -0.4306962298 -1.323904145 4.4030500895
C 0.2660508406 -1.3385282995 5.7726381918
C -0.160455655 -0.0018485805 6.4435671397
C -0.9283585301 0.7777186142 5.3499708792
N -4.9267589802 -2.7742845642 -1.3428685466
C -6.3532312904 -2.5238935765 -1.0254637284
C -7.0693547053 -3.0333151984 -2.2809026039
C -6.2032436467 -4.2266135854 -2.7161863447
C -4.7682534106 -3.7394278693 -2.4508756713
N -2.8487906903 -3.4130178656 0.196415212
C -3.6693881425 -4.4382665645 0.8950234807
C -2.6432713463 -5.1841915773 1.7549772613
C -1.6794356638 -4.063832149 2.1710264699
C -1.5450350506 -3.2252658838 0.8922827991
H -8.2212125235 2.6951007539 -3.1844234034
H -6.0278753485 1.523159077 -3.0684799779
H -4.0916830886 -4.5588763881 -2.1776440529
H -8.1100784884 -3.3066741399 -2.0830517706
H -7.0635930797 -2.2550253444 -3.0545706004
H -4.3467542944 -3.2381291664 -3.334047283
H -6.4233864063 -5.0975978894 -2.0866200977

H -6.354317845 -4.5200351021 -3.7592275054
H -6.6576174379 -3.0949679598 -0.1367410328
H -6.5229263438 -1.464923512 -0.8246056524
H -0.4848659809 -2.0074270579 -4.2401425841
H -3.7954709838 -0.4200403388 -2.8322937434
H -2.0433527752 0.2097217765 -4.3827999574
H -0.8070062743 0.165540175 -3.1159248439
H -2.7596925685 0.5421535999 -1.7599224245
H -6.1324075134 0.8254377373 5.5480596943
H -4.265376879 0.131343007 4.0701698909
H -4.1739731244 -5.0836347448 0.1697398295
H -4.4355668735 -3.9695704383 1.5293321641
H -2.1189168821 -5.9353267554 1.1512885358
H -3.1095143127 -5.6948945705 2.6028232095
H -1.3532332254 -2.1721530025 1.1122328467
H -0.7345068508 -3.6002126849 0.2539134264
H -1.4407795683 -1.7572571305 4.4645514161
H -0.7960964076 -0.1634505851 7.3191093772
H 1.3520403727 -1.3678708021 5.6381796144
H -0.0188635106 -2.2167725361 6.3595698385
H 0.7159193568 0.5616534797 6.7766747821
H 0.1171114269 -1.8605599789 3.6233867076
H -0.8638391787 -0.3655921078 -0.2882089925
H 1.6753744527 -0.574302957 2.6421933704
H 1.9275137309 1.0859940485 2.0805402627
H 1.8570296523 -1.4642173872 0.380909784
H 3.1085983651 -0.2052972855 0.3683208982
H 1.579800304 1.4059550506 -0.6630099836
H -0.7168502161 1.3895403303 -0.0916169834
H 0.4328627024 3.0270329944 1.650121795
H 0.5919476609 2.9632685625 3.4176428429
H 0.0117854533 5.3016325276 3.1127516781
H -1.0558893884 4.9294819343 1.7472201822
H -2.6466239554 5.1509075854 3.6256120156
H -1.5607219466 4.252896581 4.6987803206
H -3.2085365279 3.1598971704 2.3680476609
H -2.9829721961 2.4488287815 3.9688362667
H -1.7656164818 -3.4670399195 -2.3016199094
H -0.502066656 -2.3204035493 -1.8326738595
H -0.7137651814 -4.4311710782 2.5329714768
H 1.1728957385 -0.0756273836 -1.5370612842
H -2.0156515243 0.683349564 5.5034593484
H -0.6793942656 1.8415042462 5.321259336
H -2.1331257158 -3.4553191022 2.9618457054
H -2.236371409 -2.2530427018 -4.3523967113
H -8.1476574552 1.9146285971 4.5698214411
H -9.4882916663 3.1673221348 -1.0986633809
H -3.4331261853 1.3351914106 0.5074011432

4a

C	-0.017177742	-0.1149080013	0.0874062171
C	0.0608973054	-0.1061997286	1.4738619936
C	1.2713299681	-0.0376676716	2.1931611701
C	2.4918962918	0.0814650349	1.492258121
C	2.4208130844	0.0324611911	0.0932285495
C	1.1997798843	-0.0613787169	-0.597046994
C	0.8838086953	-0.1440022822	3.5937146211
C	-0.5231384336	-0.2303006387	3.5926739525
O	-1.0316017623	-0.1967525644	2.3127984589
C	-1.2889441563	-0.3588890543	4.7437893953
C	-0.5938863437	-0.4275552853	5.9553181829
C	0.8112705441	-0.3757002514	5.9892658371
C	1.559431429	-0.2337900412	4.8192753747
P	4.114235583	0.2201410921	2.4210608335
N	3.9036502782	1.6807502311	3.2580204035
P	3.6243653757	3.1611809141	2.8090050711
N	5.0729096644	4.0784987634	2.9044076388
C	5.8410341951	3.8944121779	4.1377259745
N	5.1205471549	0.5321490356	1.0646766834
P	6.6205972553	0.1077087931	0.9085859924
N	7.8145953332	0.8122354177	1.9026375615
C	8.0615208766	2.2464983234	1.6806176209
N	6.9116464711	-1.5419302296	1.1784898597
C	8.2393324681	-2.1304370419	1.3288588876
N	7.1381741643	0.6015730206	-0.6330565652
C	8.4508957682	0.1542405223	-1.093330657
C	5.8910512351	-2.5185985981	0.8007349025
N	2.4497074185	3.834189556	3.8244313586
C	2.0801106297	3.2084833852	5.0897016281
N	3.0626374572	3.5914503462	1.2765025554
C	1.6381849006	3.4929865955	0.9448587386
C	2.1398847951	5.25763407	3.7611130583
C	3.9290292562	3.436856385	0.1060218113
C	5.165316305	5.4521085251	2.4112970764
C	6.1623959785	0.7350839745	-1.7119531478
C	7.6940859562	0.4957333354	3.3355817777
H	-0.9729650704	-0.1793477454	-0.4227785526
H	3.3494677472	0.0912480716	-0.4646831684
H	5.7727649517	2.8567781339	4.4650612224
H	4.9749178994	3.3628470997	0.4042125844
H	6.8948642903	4.1406824782	3.9548324576
H	3.6757867724	2.523268955	-0.4432168235
H	3.800076076	4.302363638	-0.5604361087
H	4.5306782765	5.5946263988	1.5361542374
H	4.8768014107	6.1921012858	3.1759828168
H	6.2050961189	5.6578840484	2.121475651
H	1.3736488641	4.3138385827	0.263512961
H	1.4053628122	2.5405436324	0.4537754017
H	1.029636541	3.5755941069	1.8459852254
H	2.345311031	5.6550449193	2.7650458462

H 2.7163637232 5.839413782 4.4991414461
H 1.0723577465 5.4082713391 3.9728858236
H 0.9897454986 3.2436719036 5.2196531888
H 2.5489931507 3.7275778757 5.9420266254
H 7.193176119 2.864254396 1.9509912976
H 8.3056019136 2.4282289005 0.6325682284
H 8.9180612698 2.5468875444 2.2957844389
H 6.8661380669 1.0395425202 3.8112088586
H 8.6308823101 0.7691724788 3.8360795469
H 7.520767514 -0.5722488398 3.4785262543
H 8.9670506483 -1.3691349325 1.612273371
H 8.2133809943 -2.8972881945 2.1162999109
H 8.578307272 -2.6131387884 0.3980687763
H 5.8072752472 -3.286672863 1.5809672426
H 8.4222372811 -0.861286623 -1.5228584942
H 6.1468618091 -3.015942951 -0.1494891838
H 8.8131793222 0.8404274486 -1.8694426213
H 4.9187802001 -2.0376408774 0.6920556528
H 5.963170201 -0.2247577681 -2.2186005742
H 6.5507359809 1.4405751466 -2.4583095234
H 5.2275443962 1.1226886083 -1.3102798801
H 9.1677079686 0.1649335403 -0.2691565606
H 1.2009934419 -0.0812098615 -1.6846412087
H -1.1505948492 -0.5325325393 6.882983152
H 2.3965174927 2.168940913 5.0898668003
H 5.4753403118 4.5474347361 4.949480395
H -2.3717872834 -0.4159304231 4.6932661393
H 2.6422603829 -0.1877217938 4.8510238754
H 1.3243239246 -0.4444947593 6.9450903985

4aH^+
C -0.4257381555 -0.3120200551 0.7672497324
C -0.1050198703 -0.2912825433 2.1175436071
C 1.2025480691 -0.0995765282 2.6146072352
C 2.2492689798 0.0730449519 1.6830655924
C 1.9440641631 0.0315805494 0.3157848356
C 0.6275108966 -0.1481765183 -0.1352017922
C 1.0754476004 -0.1738581127 4.0631240363
C -0.2956994446 -0.4000667257 4.3034959673
O -1.0123866334 -0.4672641878 3.1299440988
C -0.846501649 -0.5357763078 5.5706581366
C 0.0347425224 -0.4387839881 6.6491120476
C 1.4090304247 -0.2099661723 6.4485024921
C 1.9399305067 -0.07334795 5.1668649609
P 3.9501186011 0.3470831188 2.2350379652
N 4.1333803759 1.7028692017 3.0879759992
P 4.0870056006 3.2330001988 2.6246208051
N 4.4158046313 3.6529818841 1.037015989
C 3.4440626963 3.3240015758 -0.0155022922
N 4.9037156712 0.1496952753 0.943425598
P 6.388323307 -0.4359310368 0.9297524985

N	7.1059580643	0.1295878822	-0.4772912449
C	8.4668668013	-0.3108642915	-0.8126383416
N	7.4722130721	0.0123934121	2.132091573
C	8.0304003057	1.3729356169	2.1357866287
N	6.3348298806	-2.1084061649	1.0999423654
C	7.5647569049	-2.9091712821	1.1833535532
C	7.2888151501	-0.5093246178	3.496128704
N	5.2941653122	4.0145641576	3.4959426763
C	5.7772558386	3.4703712912	4.7702338957
N	2.5421321228	3.8498333589	2.8410024774
C	1.7086495484	3.373812522	3.951319207
C	5.3615043812	5.4801575054	3.4335013479
C	2.1624962975	5.1920450501	2.3827733452
C	5.7921951816	3.6996628106	0.5307844657
C	5.2059554688	-2.8656973905	0.5398631338
C	6.2886150033	0.4216686852	-1.6614145704
H	-1.4493106329	-0.4633266445	0.4404165101
H	2.7493807131	0.1310928034	-0.4043486775
H	2.0669164838	-0.1338720093	7.3095163716
H	5.723077191	2.38251232	4.754665953
H	6.4804771237	4.0012968056	1.3198305995
H	6.8206905747	3.7763440413	4.9115189453
H	6.10455442	2.7230154625	0.139907679
H	5.8421338358	4.4372391488	-0.2788429433
H	5.0659885253	5.838070424	2.4452491439
H	4.7179540456	5.9530928958	4.1898333483
H	6.3947293878	5.7970349294	3.6151070645
H	3.4364722332	4.1264117441	-0.7627769672
H	3.7146012949	2.3828097225	-0.5101987233
H	2.4407887088	3.2198961279	0.3988087638
H	2.7882992576	5.5121467875	1.5492221547
H	2.2440526142	5.9312889211	3.1922294222
H	1.1198834762	5.1694286688	2.043537757
H	0.6678007265	3.2980131391	3.6154916873
H	1.7504117922	4.0676857181	4.8036086149
H	7.3605660285	2.0796006016	2.6444801272
H	8.2006298585	1.7208677828	1.1171675629
H	8.9899530473	1.3546510742	2.6645905113
H	6.6319956518	0.1455557486	4.0869935583
H	8.2649505381	-0.5577876582	3.9916020116
H	6.8596895315	-1.5114201796	3.4744366692
H	8.3791192086	-2.3251353416	1.6137255609
H	7.3823086152	-3.7762372385	1.828947982
H	7.8748185243	-3.2762687509	0.1947046275
H	5.0143469125	-3.7387661015	1.17417936
H	8.4632837744	-1.2412888267	-1.3987982121
H	5.4261375514	-3.2201204443	-0.4780366636
H	8.9540698751	0.4685786293	-1.4093199076
H	4.3057419412	-2.2517624517	0.5094743439
H	6.1750972685	-0.4631733663	-2.3052340995
H	6.7796471032	1.2107792751	-2.2426615647

H 5.3018901938 0.7688707586 -1.3576864373
H 9.0563584307 -0.4655889491 0.0928071654
H 0.4266989998 -0.1712255921 -1.2022864913
H -0.3491111536 -0.5399294219 7.6600864356
H 2.0346956307 2.3902825374 4.2803982742
H 5.1889578635 3.8436973984 5.6218064558
H -1.9090820585 -0.7096457467 5.7039655377
H 4.1664679043 -0.6680455728 3.1874592304
H 2.9964862463 0.1330271841 5.0371811073

4b
C -0.0970877171 0.2663417081 0.0674307481
N 0.1272486657 0.0852424132 1.5209236225
C 1.5388174482 -0.214904756 1.8056378146
C 2.0081717002 -0.8579297706 0.4957740205
C 1.2791564141 -0.0206756225 -0.5677444026
P -1.0300043836 0.6689689375 2.589764243
N -0.8881776637 2.3382074972 2.5666581813
C -1.9896978264 3.2537799069 2.9457916732
C -1.2595903034 4.5381296268 3.3648148666
C 0.0008336728 4.5259591384 2.4850458469
C 0.401614427 3.0428585063 2.483321886
N -2.4933579414 0.1510780173 2.3814905284
P -3.6047595662 0.5711948588 1.1559651127
N -4.2005184262 -0.8817195388 0.5369550005
P -3.7047239278 -1.9932785978 -0.4301481786
N -2.5901931724 -1.5243750859 -1.6050649052
C -1.6259778552 -2.401418637 -2.3015352333
C -1.4466564006 -1.7408370323 -3.677597374
C -1.6919678731 -0.2518389907 -3.3845030929
C -2.8476044255 -0.2912766806 -2.3770016169
C -5.0938021593 0.9950165225 2.192182676
C -6.3287855605 1.3084271911 1.5826778518
C -7.4236108664 1.676798384 2.3899636094
C -7.3713716617 1.7654991547 3.7744136618
C -6.1426220281 1.4588306549 4.364848867
C -5.0327453937 1.085137692 3.5876675002
O -8.5528833334 1.9398299774 1.638115872
C -8.1868230803 1.7443834511 0.3263967665
C -6.8348541292 1.3586713212 0.2163043043
C -9.0360789431 1.900685646 -0.7615023934
C -8.4901090986 1.6624505025 -2.0267135721
C -7.1437198758 1.2834343937 -2.1731739349
C -6.3100820226 1.1278990437 -1.0642229708
H -5.2818635197 0.8134351313 -1.1826492956
N -0.3898614923 0.1545007347 4.077325802
C -0.5428453204 -1.2667872655 4.4392508815
C 0.0670680002 -1.336037458 5.8514162954
C -0.1257144716 0.095847565 6.4300326109
C -0.7823780549 0.8996236736 5.2868733055
N -5.0382116195 -2.6401485785 -1.2050080155

C -6.4323342429 -2.4783952417 -0.7640337763
C -7.2177405399 -2.7448525782 -2.0513768692
C -6.3988083675 -3.8632056761 -2.7204336845
C -4.9346655257 -3.4933642906 -2.3966204215
N -2.9117876108 -3.3393334076 0.2267666786
C -3.7556046747 -4.3575234006 0.8969009147
C -2.771452803 -5.0920276041 1.8138792772
C -1.8561937338 -3.9531971153 2.2857967364
C -1.6756025456 -3.102377387 1.0192740007
H -9.1179415297 1.772228597 -2.9073099023
H -6.7446259564 1.1037733826 -3.1685835018
H -4.3190220537 -4.386306356 -2.2125014037
H -8.2580288826 -3.0296496052 -1.8614666173
H -7.2189095575 -1.8391489381 -2.6706427153
H -4.46578272 -2.9386690407 -3.2228046191
H -6.6484008665 -4.8288244307 -2.2624695163
H -6.5752106436 -3.9512576425 -3.797794009
H -6.6922656556 -3.2173022231 0.0126137834
H -6.5858675855 -1.4817590247 -0.3495661689
H -0.4627956808 -1.9444083555 -4.114207062
H -3.816480161 -0.3482482102 -2.8999607692
H -1.9342181879 0.3347498938 -4.2767075379
H -0.8043579044 0.1933364932 -2.9173781925
H -2.8710865442 0.5808907974 -1.7153668772
H -6.046765185 1.5031873848 5.4476593776
H -4.0950787438 0.8308221077 4.0734450986
H -4.2255093929 -5.0137146818 0.1560171571
H -4.5535381916 -3.8915185615 1.4950366269
H -2.1981776237 -5.8318687434 1.2395251662
H -3.2776234673 -5.6138709711 2.6331658099
H -1.5515133033 -2.0449071946 1.2660081483
H -0.7991930242 -3.4290896554 0.4410076601
H -1.6078208882 -1.5451833592 4.4510919729
H -0.7527830157 0.1041140717 7.3275564427
H 1.1321907252 -1.5851005 5.7945141004
H -0.4197484334 -2.1061403759 6.4589472086
H 0.840133207 0.5354129249 6.7000254677
H -0.0401892522 -1.9112849837 3.7121391974
H -0.864422275 -0.4293676884 -0.2849041456
H 1.6319860158 -0.8780705221 2.6684241448
H 2.1237550365 0.6939687474 2.0235227662
H 1.6693528803 -1.9011447637 0.4562596559
H 3.09784564 -0.8454231261 0.3855638149
H 1.8230303727 0.9171001293 -0.7382383102
H -0.4488066755 1.2814262343 -0.1608192943
H 0.9449940129 2.7553248944 1.5759749385
H 1.045915452 2.8077871595 3.3464685401
H 0.800752314 5.1749794218 2.8567894974
H -0.2506212217 4.8450034844 1.4656485603
H -1.8769468308 5.4318632081 3.2280424404
H -0.9760682201 4.4826791409 4.4244440261

H -2.6489571024 3.4269420372 2.0857206912
H -2.6044044658 2.8349061845 3.7477355107
H -1.9868867105 -3.4317887645 -2.3654815264
H -0.672568831 -2.4222914949 -1.7525816079
H -0.9030527925 -4.2996510933 2.7006867556
H 1.1895727606 -0.5310807465 -1.5322257717
H -1.8790309738 0.9118503334 5.4082391374
H -0.4348742036 1.9348861421 5.22948628
H -2.366556026 -3.3582391266 3.0520016397
H -2.2083604348 -2.1135888802 -4.3749240646
H -8.2421400076 2.056599337 4.3533349975
H -10.0719352185 2.1952015511 -0.6245369059

4bH^+
C -0.1556893141 0.3628007696 0.1116839903
N 0.0793066724 0.1417551666 1.5612700648
C 1.5181041238 -0.0599485826 1.842286483
C 2.04421617 -0.5736405766 0.4987243935
C 1.2472845266 0.2588090555 -0.5180081772
P -1.079637065 0.6237253364 2.6495476024
N -1.1283883073 2.2854881875 2.6491282816
C -2.279863447 3.0832624366 3.1501474109
C -1.6366173519 4.3996808135 3.6080055444
C -0.4252899598 4.5439587489 2.6737148344
C 0.1031678107 3.106980259 2.5750083506
N -2.5138904556 0.0022284544 2.3561355384
P -3.7021486544 0.3023398157 1.3123233426
N -4.1850159781 -0.8793081398 0.358138989
P -3.6335455995 -2.0687224332 -0.5310301491
N -2.4659540217 -1.5640064009 -1.6138164739
C -1.4941179426 -2.4498742933 -2.3029998546
C -1.4018189029 -1.8687195478 -3.7194840155
C -1.6310771973 -0.3671315814 -3.4887163416
C -2.7305929313 -0.3455432567 -2.4178883648
C -5.1645930064 0.8664240003 2.2170606792
C -6.3142032821 1.330210911 1.5430628679
C -7.412660217 1.7574960503 2.319526893
C -7.4362144508 1.7378710335 3.7075098995
C -6.2907848398 1.2660458678 4.3514984472
C -5.1720135874 0.842098691 3.6168765963
O -8.452363732 2.1903617595 1.5356267156
C -8.034962174 2.0460300496 0.2323629386
C -6.7279916353 1.5208886954 0.1596222898
C -8.8010517259 2.3704219814 -0.8795848862
C -8.2146481478 2.155974694 -2.1284308192
C -6.9121340248 1.6342019146 -2.2398316303
C -6.1623058739 1.3130426392 -1.1094305148
H -5.1733059074 0.8854325301 -1.2121891316
N -0.4729838141 0.0985788005 4.1175391911
C -0.4840043032 -1.3406887419 4.4460953426
C 0.2033912073 -1.3818120221 5.8205685124

C -0.1215894593 -0.0036361055 6.4646056236
C -0.850363506 0.7995894991 5.3626431251
N -4.9539633073 -2.6708553328 -1.3234365115
C -6.3702421854 -2.4448028462 -0.9538194195
C -7.1153214746 -2.8885332501 -2.2161931322
C -6.2719137358 -4.073176718 -2.7159041218
C -4.8233204441 -3.6261312068 -2.4442406663
N -2.89877018 -3.3551663299 0.2319686771
C -3.7534802216 -4.3577540482 0.9215587409
C -2.7548140339 -5.1346320865 1.7861275759
C -1.7649927723 -4.0420054178 2.2150796049
C -1.5991356128 -3.1997873769 0.941979939
H -8.7753771302 2.3953649872 -3.0274336946
H -6.4849031115 1.4761840296 -3.2264569353
H -4.1706879953 -4.4694570292 -2.1839906127
H -8.1559394291 -3.1581821572 -2.0126235997
H -7.1118072585 -2.0753846379 -2.9530279464
H -4.3865385982 -3.1233976418 -3.3185428612
H -6.5020162038 -4.9689562918 -2.1261741589
H -6.4382956026 -4.315270115 -3.7697244063
H -6.651633966 -3.066400225 -0.090917059
H -6.5347058987 -1.398996813 -0.6919612428
H -0.4451485398 -2.093029975 -4.2010609848
H -3.728611659 -0.3923002484 -2.87777643
H -1.9240936221 0.1741511035 -4.3929922235
H -0.7176515434 0.0990885812 -3.0988622474
H -2.691171183 0.5512831901 -1.790112694
H -6.2646109974 1.2271295724 5.4366878913
H -4.2920195154 0.478265117 4.1380356786
H -4.2710439823 -4.9864164289 0.1907298866
H -4.5105115564 -3.8695639229 1.5524170639
H -2.2459174578 -5.896638586 1.1828593735
H -3.2410273131 -5.6364281209 2.6280418477
H -1.3855310811 -2.152551042 1.1695935635
H -0.7899495331 -3.5889630742 0.3103431527
H -1.5198165825 -1.7081855589 4.5067678817
H -0.7522304234 -0.0997338953 7.3532679627
H 1.2844966222 -1.4983863946 5.6950097096
H -0.1524648974 -2.2240307625 6.4213883866
H 0.7965195037 0.5069123271 6.7695987182
H 0.0330059793 -1.9215432007 3.6772733186
H -0.8359056651 -0.4009971591 -0.2775960914
H 1.6587551996 -0.7689129321 2.6603382551
H 2.0132606328 0.8807713954 2.1236219875
H 1.806598399 -1.6394627189 0.3908002556
H 3.1271550101 -0.4532326414 0.4006817536
H 1.6941001802 1.2553704157 -0.616084833
H -0.6109369263 1.3452368999 -0.0726649868
H 0.6384540849 2.912454672 1.6399954421
H 0.7831486021 2.8798802271 3.4095440778
H 0.3319074555 5.2390416912 3.0479427544

H -0.7484436227 4.8933891658 1.6853644456
H -2.3325342312 5.2417318206 3.5505940812
H -1.2999355663 4.3119046881 4.6487965492
H -2.9986041189 3.2536189728 2.3386298876
H -2.8097897602 2.5682452474 3.9559024339
H -1.8185987941 -3.4930758164 -2.280892931
H -0.5216023156 -2.3920491133 -1.7942371986
H -0.8119503078 -4.4365827502 2.5817620716
H 1.2178921188 -0.1903116677 -1.5153709208
H -1.9407906498 0.7644726751 5.5196035864
H -0.5454000586 1.8481144926 5.3177238933
H -2.208253456 -3.4268756255 3.0066830267
H -2.2011108308 -2.2799462261 -4.3485790279
H -8.3105642055 2.0753707006 4.2543796252
H -9.8037924025 2.7703710946 -0.7712452227
H -3.3514325377 1.4165358683 0.5056966179

5a
C -0.1623867868 0.0237796293 -0.0438892515
C -0.135567352 0.1399370677 1.3748545449
C 0.9659721473 -0.1563161024 2.1514790944
C 2.0910938238 -0.6118729905 1.4035023898
C 2.0754353492 -0.7360380786 0.015043421
C 0.9262921548 -0.4159745949 -0.7603118871
C -1.5494708519 0.6597034979 1.3639022556
C -1.5809575562 0.5485466045 -0.0583402318
C -2.6842385223 0.9033061803 -0.796971699
C -3.7996935684 1.3852950385 -0.0537057832
C -3.7688777704 1.4876976143 1.3348011585
C -2.6198660958 1.1209011308 2.0902783316
P 0.8668861499 -0.039224143 4.0102029103
N 2.5243839642 -0.3097740088 4.3765127628
P 3.0151132258 -0.9741355133 5.7123440855
N 4.0302999848 0.1121594541 6.5440579836
C 4.78412694 1.0866945291 5.7567458815
N 0.5319089243 1.6056420094 4.2190484049
P 1.1636413874 2.9580818085 3.7362447604
N 2.062399446 3.1484735159 2.3163396739
C 3.452829036 2.6948307186 2.2423563592
N -0.1056450874 4.0514523427 3.4796127142
C -1.3965236926 3.8590390266 4.1335264841
N 2.2798677436 3.5387085016 4.8928321222
C 2.8773887815 4.8642351634 4.750879713
C 0.1088280362 5.4134649562 3.0072189523
N 1.9486284438 -1.4306163188 6.9535080798
C 1.2805934779 -0.3804867336 7.7305117197
N 3.7920850011 -2.4501814077 5.3944553975
C 4.4100462901 -3.2520818479 6.4447182391
C 1.0749150993 -2.5904107932 6.7315013944
C 4.2830146332 -2.7610305867 4.0565063423
C 4.7104337558 -0.2775667653 7.7761786872

C 2.059907289 3.1721600418 6.2905166781
C 1.3957933924 3.1226022628 1.0102192705
H -2.7279862521 0.828689956 -1.8804333917
H -4.6477257181 1.8584210277 1.8572095584
H 3.0010412928 -0.8422054215 1.9484686041
H 3.9424997583 2.8034074111 3.2096538668
H -1.4720705437 2.8331030857 4.4921627054
H 3.5178994749 1.6435895801 1.9327910522
H -1.5181901533 4.5484795213 4.9855006957
H -2.2071902755 4.0488939403 3.4163575976
H 0.3234438117 3.2899584343 1.1203625474
H 1.8137319935 3.9154630801 0.3731848229
H 1.5442571016 2.1585844083 0.5109919392
H -0.6965104929 5.689532812 2.3117740744
H 0.1056289109 6.1423820955 3.8340835763
H 1.0573602387 5.4940880402 2.4741227005
H 3.0685976857 5.090598405 3.7001652368
H 3.8369700952 4.885794729 5.2850090955
H 2.2387833008 5.6589669135 5.171604833
H 1.3587400841 3.8606108922 6.7936721415
H 3.0164102365 3.2062083889 6.8266480084
H 5.3573398525 -2.5359948383 3.9504986592
H 3.7260020845 -2.1853951789 3.3197754979
H 4.1359832804 -3.8308326288 3.8527021865
H 5.4905505039 -3.0564547875 6.5381628288
H 4.2807482443 -4.3180971329 6.2108536856
H 3.9320482095 -3.0590466997 7.4065234308
H 4.0821109176 -0.9490488887 8.36450629
H 5.6779210118 -0.7712347523 7.5872357598
H 4.903719867 0.620949196 8.3773605008
H 5.7309730323 0.6698418879 5.372500556
H 0.8140675912 -3.0284448137 7.7042508884
H 5.0266682751 1.9474614641 6.3933003162
H 0.1517830481 -2.3043462244 6.2083304775
H 4.1744949866 1.4269641205 4.9217140475
H 0.959871979 -0.8034252103 8.6915493846
H 0.3960193442 0.0146058905 7.2091241881
H 1.9696267874 0.4422366774 7.9261200472
H 1.5914529476 -3.3448150232 6.1357763319
H -4.7007221321 1.6789090429 -0.5869188517
H 2.9739549273 -1.0804974666 -0.4928358106
H 1.6634594803 2.1611212843 6.3458113071
H 3.9867816653 3.3120749312 1.5069532642
H 0.9350237008 -0.5134808302 -1.8426312226
H -2.6005382378 1.2068636859 3.171356432

5aH^+
C 0.0605565647 0.0201612483 -0.1740053762
C 0.0804940892 0.1706229873 1.2436277079
C 1.1854416953 -0.1371269133 2.0066792333
C 2.3187975884 -0.6251029491 1.2869746058

C	2.2967838012	-0.7693924688	-0.09458326
C	1.1478021689	-0.4446631555	-0.8725224627
C	-1.3252363332	0.7067092104	1.2149580702
C	-1.3511778191	0.5611128402	-0.2022308328
C	-2.4430364754	0.919884531	-0.9561057644
C	-3.54798443	1.4415371156	-0.2286283837
C	-3.5222095637	1.580978253	1.1582755475
C	-2.3863072179	1.2114299269	1.9276420381
P	1.1625641611	0.078297982	3.8024444711
N	2.572318249	-0.4782566764	4.343487093
P	3.0224054148	-1.0627550563	5.7535125131
N	4.0747682631	0.0426447081	6.4647481131
C	4.878260742	0.9176224674	5.6004887681
N	0.6740034992	1.5547090518	4.2217418693
P	1.11755243	3.0148819495	3.7538165162
N	1.7338431225	3.2766681068	2.2265903107
C	3.1147509534	2.9158010247	1.8818081304
N	-0.2599197591	3.9618766054	3.8133088348
C	-1.4363320543	3.5878822211	4.6021592709
N	2.3766990604	3.5486969388	4.7495019036
C	2.9617561272	4.8811956165	4.5439083622
C	-0.2013761136	5.3912249006	3.4932823069
N	1.8882775943	-1.3341356552	6.9587967135
C	1.3432618626	-0.2240732863	7.7517112247
N	3.6613184989	-2.5894715681	5.490987514
C	4.1942293224	-3.3920741917	6.5974239609
C	0.9479411725	-2.4581610822	6.8348205187
C	4.1583584921	-3.0044202914	4.175355451
C	4.754961126	-0.3038230266	7.719781292
C	2.3213255294	3.1843768778	6.1701778277
C	0.8750255369	3.4636088113	1.0506334082
H	-2.484320351	0.8200435393	-2.0367228131
H	-4.3963213502	1.9809542549	1.6650442907
H	3.2187088533	-0.8715407882	1.8407088405
H	3.7096118123	2.7633730582	2.7830116548
H	-1.4072408462	2.5239217459	4.8337416247
H	3.1343420703	1.9953910295	1.2851936523
H	-1.477213104	4.1563430396	5.5426365871
H	-2.3434639485	3.8064685157	4.025099415
H	-0.1695710637	3.5666391009	1.3436812099
H	1.1865800934	4.3662287396	0.5093736857
H	0.9609524686	2.6042784485	0.3764784757
H	-1.1064311234	5.6748345075	2.9424574901
H	-0.1441588026	6.0056745509	4.4030211664
H	0.6610991796	5.6152130093	2.8636260203
H	2.995679134	5.1314388734	3.4827649753
H	3.9884403532	4.878347337	4.9277904249
H	2.3991693759	5.6635768223	5.0744583021
H	1.6489654935	3.8452813266	6.7397718724
H	3.3266837202	3.2713797592	6.5949889876
H	5.2442743874	-2.8536695879	4.0872313

H 3.6531752737 -2.4429429561 3.3914340336
H 3.9452608739 -4.0708905967 4.0356751459
H 5.2838958604 -3.2831890237 6.6914403712
H 3.9702986441 -4.4493650566 6.4118779477
H 3.726493923 -3.1085174113 7.5414342161
H 4.1069782901 -0.910371533 8.3549672643
H 5.6929438015 -0.8503171361 7.5441038345
H 4.9923342684 0.6196357613 8.2601840687
H 5.7702097976 0.4063339866 5.2076529682
H 0.7459943997 -2.869730861 7.8305233345
H 5.2115148588 1.7763238138 6.1933744668
H -0.0060999287 -2.1324765836 6.3959110334
H 4.2745489533 1.2807868894 4.7696900142
H 1.0198310217 -0.6131092673 8.7236854179
H 0.4780163766 0.2414867664 7.2575471694
H 2.1045621215 0.5371343027 7.9194699351
H 1.3710491754 -3.2454403433 6.2100944143
H -4.4411068262 1.7378362227 -0.7718331072
H 3.184956546 -1.1404781281 -0.5986648823
H 1.9894001937 2.1548308269 6.2787756711
H 3.5676938538 3.7249558909 1.2949800263
H 1.1574487261 -0.5633333506 -1.9519450884
H -2.3742093818 1.3295916154 3.0057604022
H 0.1112802765 -0.7417611419 4.257877518

6a

C 0.997073496 -0.0829887572 -0.3463644372
C 0.1967451058 0.1591650647 0.9817732687
C 1.0799241751 0.138890175 2.2420831778
C 1.649280277 -1.3030825003 2.1421245732
C 2.4612191405 -1.5463065285 0.7953612332
C 2.4978846208 -0.2691508381 -0.0735447124
C 0.3044223848 -2.0828310052 2.052921572
C -0.3722603424 -1.3078428995 0.8988336218
C 1.6310790334 -2.4694077897 -0.1417282143
C 0.4269752571 -1.5446015265 -0.4259603685
C 1.2383152185 -3.8155919168 0.4714593123
C 0.4473637301 -3.5835580891 1.7840043836
P 2.2321466904 1.5773813074 2.6437897561
N 2.4981732238 1.2092675146 4.3049460929
P 3.7734120604 0.5965611957 4.9602953674
N 5.2420185065 0.413530113 4.1175077922
C 5.2965811905 -0.5769779514 3.0348812482
H 2.9628524057 0.5935923765 0.408510538
N 1.0069706532 2.7584433136 2.6771351256
P 1.0605637915 4.2202927448 3.2156601549
N -0.0799338651 5.1304549078 2.35043363
C -0.3500049262 6.5219557261 2.6902354448
N 2.4540510027 5.1955388114 3.0665745043
C 3.6149345731 4.8764822694 3.9068507159
N 0.8087418652 4.294526457 4.9004092427

C 0.7854219945 5.5586118803 5.6313585723
C 2.8592798186 5.53601157 1.6970694873
N 4.2613698675 1.573805662 6.2660204901
C 5.313576512 1.1319409535 7.1747015384
N 3.4894660553 -1.0127780669 5.4431724049
C 4.5094459338 -1.8560832076 6.0578552766
C 3.2837945261 2.4614657299 6.8900569641
C 2.1260355879 -1.4187646733 5.7774929298
C 5.9848823322 1.6303952534 3.7730544281
C -0.0518117387 3.270589138 5.4973499668
C -1.1889783145 4.4596591552 1.6765734783
H -0.51822054 0.9845907386 0.9612644493
H 3.0448640613 -0.4675455231 -1.0060273491
H 0.4069419259 0.1693469519 3.1124737075
H 2.2006502542 -2.6408260437 -1.0656957442
H 3.4551222653 -1.9562696792 1.0087317336
H -0.1599571501 -1.8254142051 -1.3066455673
H -1.4583465683 -1.4369927903 0.8431658095
H -0.2525907768 -1.9354568929 2.987603398
H 2.2439957961 -1.5923343844 3.0131075606
H 0.7616822664 0.5998061483 -1.1683481751
H -0.5506063326 -4.0387910486 1.7270178552
H 0.9544249778 -4.0564946877 2.6361806708
H 0.6402586063 -4.3852009379 -0.2525019694
H 2.1442082463 -4.4080670047 0.6582951238
H 1.4148505944 -0.7717355644 5.2631849952
H 1.9621648259 -2.4551849147 5.4514354948
H 4.3566270085 -2.8979065936 5.7425453878
H 5.5060550527 -1.5474617571 5.7377910192
H 4.712975933 -1.4608903211 3.2953642807
H 4.9052442583 -0.1687955711 2.0919716736
H 6.3411529388 -0.8786391646 2.8807216275
H 7.0378218402 1.3681807642 3.6052633475
H 5.5905803594 2.0993346395 2.8584840838
H 5.929034675 2.3509606499 4.5908072613
H 6.071507298 0.5626610189 6.6323603931
H 5.8013343216 2.0113625484 7.6161394303
H 4.2042450568 4.0459178936 3.4924000038
H 4.2589693628 5.7635131873 3.9720394721
H 3.8020455926 3.3452283027 7.2882313277
H 2.7546490011 1.9719975829 7.7257639672
H 2.5538838642 2.7797776571 6.14678492
H 3.5300214629 6.4040855103 1.7319468341
H 1.9845261978 5.7929784623 1.0969952231
H -1.4318706058 5.0018902807 0.7520954997
H -0.898558411 3.4387455345 1.4283711767
H -2.0943108109 4.4315881691 2.3060416592
H -0.6507979726 7.063585147 1.7829713698
H -1.1210262898 3.5325373255 5.4222872801
H 0.1996382129 3.172583305 6.5614610262
H 0.5485642666 6.999998238 3.085737366

H 1.4503119505 6.2858531566 5.1620194691
H 1.1355095982 5.3875910388 6.6590270365
H -0.2268190571 5.9914093029 5.6884728342
H 3.3884424064 4.7032543014 1.2077636795
H -1.1625385158 6.6253005512 3.4286549852
H 1.9387157387 -1.3666125999 6.8632456413
H 3.2979380931 4.5985955803 4.9112812368
H 0.1255100137 2.3120408013 5.0109908614
H 4.4666157719 -1.8284149836 7.1588462989
H 4.9259845808 0.5115232028 8.0000791508

6aH^+
C 0.8479069575 -0.0464542323 -0.2653924194
C 0.086074032 0.0759535935 1.0977062058
C 1.0115707148 0.0376229597 2.3311493873
C 1.6869460489 -1.3497782971 2.1380555744
C 2.4587153063 -1.4564708679 0.7514529964
C 2.3660483085 -0.1445432519 -0.0621315857
C 0.3891169518 -2.2082856725 2.0554822511
C -0.3798424012 -1.4235317144 0.9678510327
C 1.6553768739 -2.3903137344 -0.2006838458
C 0.3800004154 -1.5400862006 -0.3949304869
C 1.3819330107 -3.7885529402 0.3568132923
C 0.6219017151 -3.6787773564 1.7021821732
P 1.9764698731 1.48370242 2.8871585857
N 2.4728975406 1.2266583684 4.4034944274
P 3.7735478653 0.5844105789 5.0404467528
N 5.1843117971 0.4617556572 4.137979126
C 5.3066877006 -0.5477474402 3.0776564436
H 2.8065969728 0.7368341272 0.4069189333
N 1.0041675342 2.7484271187 2.7037966899
P 1.0715702798 4.2541154044 3.208477975
N -0.0534913647 5.0939749033 2.3044380704
C -0.3289612517 6.5027645601 2.6003110695
N 2.4913904746 5.1333419765 3.010319319
C 3.6638271267 4.7997744002 3.8298133647
N 0.8384151123 4.3308515736 4.8781679922
C 0.9283525041 5.6070811981 5.6005438709
C 2.8633852946 5.5937486047 1.6650698458
N 4.2235941988 1.5692697412 6.3245754899
C 5.3168701263 1.156248616 7.2111896173
N 3.4652623999 -1.0199205764 5.4455059401
C 4.4985906562 -1.877932795 6.0381847868
C 3.2431993641 2.4477249689 6.9713498128
C 2.1045702006 -1.4331587194 5.8084177204
C 6.0090267577 1.6483230446 3.8829732304
C -0.1431414114 3.4166230534 5.4789088863
C -1.1105893654 4.4237017749 1.540525228
H -0.6841502648 0.8477492993 1.1506128625
H 2.8787579913 -0.2717767122 -1.0233923328
H 0.3569331333 -0.0422138788 3.2145380131

H	2.1987989699	-2.4753247834	-1.1502196621
H	3.486193384	-1.8023625814	0.9061211853
H	-0.2211438841	-1.8179671126	-1.2644272862
H	-1.455312043	-1.6188266192	0.9469914338
H	-0.1394656323	-2.1481709668	3.016213073
H	2.3264278568	-1.640226275	2.9763490671
H	0.5326796261	0.6566222471	-1.0404321381
H	-0.3467055475	-4.1910077577	1.6511131342
H	1.1830591241	-4.1616937967	2.5127605218
H	0.802444159	-4.3634733127	-0.3755750224
H	2.3326904883	-4.3212322452	0.4846670711
H	1.37309495	-0.7808344291	5.332536547
H	1.938032993	-2.4600499373	5.4616876037
H	4.3369479586	-2.9104806899	5.7061736944
H	5.4928121995	-1.5674931133	5.7138810878
H	4.6866613752	-1.4163461784	3.2975702805
H	5.0058549752	-0.1413539045	2.1014863396
H	6.3518876663	-0.8710870008	3.0089726263
H	7.0634112115	1.3509723049	3.8397724614
H	5.7458244328	2.1178154329	2.9233023381
H	5.8827490727	2.3807215251	4.6805999853
H	6.065665861	0.5849206835	6.6591335972
H	5.8042342306	2.0505981545	7.6162688243
H	4.2908245418	4.0411183463	3.3394676739
H	4.2687558776	5.7023791494	3.9752373972
H	3.7644939778	3.3320811409	7.3582455696
H	2.7429554869	1.948055528	7.8148240126
H	2.4936490987	2.7649230482	6.2471717158
H	3.5021876177	6.4789917436	1.7595147151
H	1.9753452777	5.861930702	1.0923128968
H	-1.2608894971	4.9582782964	0.5946379381
H	-0.8187899746	3.3964429845	1.3257130438
H	-2.0610446855	4.4207142432	2.0935623917
H	-0.5409588279	7.0322678044	1.6638422768
H	-1.1684132871	3.8099496586	5.404687781
H	0.0986725188	3.2910246034	6.5402470769
H	0.5369809849	6.9767546016	3.0666065497
H	1.6609748735	6.2658010749	5.1328239751
H	1.2506403716	5.4077940723	6.6296446109
H	-0.040734839	6.1253215633	5.6397843003
H	3.4188704863	4.821393931	1.1118031994
H	-1.198188523	6.616593794	3.2639839421
H	1.9512605168	-1.405810285	6.8973556776
H	3.3610343117	4.4176483169	4.803956514
H	-0.09606659	2.4395999803	5.0001674989
H	4.4618056149	-1.8612213088	7.1367162859
H	4.956194648	0.5497654236	8.0547744798
H	3.1284697993	1.6442876315	2.0846053222

6b

C 0.407663994 0.4643490369 0.5282675723
N 0.3356435178 0.232388297 1.9885716231
C 1.6801610259 0.1766710397 2.5860080032
C 2.5904033744 -0.1197353988 1.3874071525
C 1.9094860812 0.6737072583 0.2607013301
P -1.0922686117 -0.2637820597 2.7309021715
N -1.51363197 0.7856888565 3.993711992
C -2.3057796192 1.9944523725 3.6886860485
C -2.4016072527 2.7399531492 5.0466374498
C -1.4246710638 1.9983942646 5.9963891034
C -0.5609149583 1.1537143941 5.0498405723
N -2.1607318889 -0.4849625302 1.637071014
P -3.7898348143 -0.5177779574 1.1930632166
N -4.719266246 -0.0428622694 2.5625443645
P -5.6886545196 -0.9905148671 3.3546448104
N -7.2056324247 -0.2701705929 3.4320329687
C -7.7217729078 0.6061222321 2.3598168865
C -9.2279762105 0.3151580983 2.3464449058
C -9.5304168373 0.013654957 3.8225858018
C -8.296688116 -0.786645913 4.2766784952
C -3.820766228 1.1492414972 0.296765783
C -3.1816362465 1.2334816389 -1.1156646756
C -4.095137415 0.5579507942 -2.2316099773
C -5.3916600322 -0.0247881556 -1.622364484
C -6.0325230246 1.2566304314 -1.0689693566
C -5.1325413611 1.9127330066 0.036290099
C -5.6103366849 2.3784237654 -2.0838639566
C -4.7132930465 1.6719632259 -3.1245422498
C -3.2253759425 2.7771177996 -1.3097193033
C -4.7137648544 3.0385947714 -0.9840527357
C -2.8081856459 3.2670087378 -2.6993798702
C -3.6937198697 2.603349584 -3.7846716496
H -5.2217844436 -0.7948272388 -0.866395954
N -0.5963243649 -1.6105298654 3.6530329934
C 0.0219918823 -2.7267181162 2.8965693204
C -0.9317286934 -3.9110811471 3.0897948517
C -1.4386320647 -3.6880183649 4.5222584829
C -1.6027122974 -2.1598028801 4.6008467338
N -5.1598575558 -1.28760869 4.9222193529
C -4.4572829104 -0.2296766731 5.6764700983
C -4.1325843331 -0.8813168028 7.0473395215
C -4.5374921573 -2.3622727358 6.8933454374
C -5.6631496476 -2.300420003 5.855126278
N -5.9911402545 -2.5696874599 2.8124792815
C -4.8906895721 -3.5654319076 2.7666183412
C -5.0204082064 -4.2304820186 1.3897774873
C -6.5273017874 -4.1328838999 1.1064325104
C -6.8784858494 -2.7363454274 1.6360253132
H -7.0972093092 1.1727800056 -0.8269088348
H -3.2128379364 1.7744841065 0.9643977028

H -6.0119145004 -0.4640505107 -2.4165385786
H -2.5827272338 3.246783514 -0.5530383552
H -2.163937876 0.8322102124 -1.1476735196
H -4.9451361009 4.0777121863 -0.726429506
H -6.3953192616 3.0075018547 -2.516574632
H -5.334504911 1.202276179 -3.8999351784
H -3.5230255867 -0.1757894312 -2.8105392075
H -5.6459163119 2.22093637 0.9490210899
H -4.2229320729 3.3613116174 -4.3778736354
H -3.0820778564 2.0279648436 -4.492546853
H -2.8929688453 4.3615551894 -2.7390206633
H -1.7474507235 3.0328490466 -2.8620606025
H 0.0138579153 -0.4031994306 -0.0156691084
H -0.1932550142 1.3307049537 0.2324468983
H 2.1987697228 0.3430373271 -0.7424044404
H 2.1669508809 1.7372273306 0.3498968523
H 2.5702467256 -1.1941094353 1.161862387
H 3.6315150826 0.1740293543 1.5609782826
H 1.7303072094 -0.5830691381 3.3709365831
H 1.9522505042 1.1432307078 3.0390486248
H 0.2704363141 1.7606944883 4.6494532159
H -0.1344145964 0.2613759926 5.5155366301
H -1.9769781155 1.3377840405 6.6736064942
H -2.1215217541 3.7921546104 4.9279400588
H -0.8245478267 2.679097048 6.6102121179
H -3.4249200003 2.7190027129 5.4352614707
H -3.2788797191 1.699553034 3.2925560677
H -2.617141568 -1.8701647929 4.3068819331
H -2.3771093094 -4.2107425159 4.7372140708
H -0.6886584522 -4.0337463506 5.2456067614
H -0.4426199781 -4.8811789638 2.9471456541
H 1.0139208857 -2.953010433 3.3167422344
H 0.1571524027 -2.4605837035 1.8439104477
H -1.7593495782 -3.8324668029 2.3747574936
H -5.8464884079 -3.2466175841 5.3429195909
H -6.6056284291 -1.9914723789 6.3388185036
H -3.7040675196 -2.9488261086 6.4902948541
H -3.5633712907 0.0705158004 5.1298488712
H -5.0907069304 0.6614174017 5.8036492449
H -4.8541850208 -2.8201301966 7.8369329227
H -3.0763600306 -0.7651378772 7.3118382296
H -4.7212014183 -0.4096061786 7.8427835294
H -7.5224986238 1.6601109805 2.6004583744
H -7.2359324426 0.3943685189 1.4058622002
H -9.4329982643 -0.5701279103 1.7303995991
H -9.8140913602 1.1489972302 1.9454434077
H -8.4441169226 -1.8669650943 4.1214377007
H -8.0795085897 -0.6321594899 5.3397715441
H -9.5966104182 0.9515539335 4.3887287033
H -10.4638474327 -0.5382639063 3.9761901561
H -7.92982497 -2.6456341996 1.9317885949

H -6.6730268087 -1.9798366906 0.8654692019
H -7.0739265073 -4.9003034221 1.6706551125
H -6.7785830782 -4.2467494668 0.0467544385
H -4.4621990216 -3.6433760598 0.652111334
H -4.6422873278 -5.2588377346 1.3836233507
H -3.9168371905 -3.0901111553 2.904672503
H -5.0213674491 -4.302387933 3.5739916857
H -1.4328442811 -1.7717100307 5.612070587
H -1.7951421796 2.6245907546 2.9413463399

6bH^+
C 0.0122635058 -0.3003578894 0.1002719405
N 0.0180749781 -0.081767218 1.5681745398
C 1.3999318862 0.0519595255 2.0854282141
C 2.2689639601 -0.460679453 0.9299208423
C 1.4673342016 -0.0280409914 -0.3073235357
P -1.298760114 -0.3567552122 2.5468427317
N -1.5398251369 0.8404919455 3.6851405956
C -2.3504780277 2.047401113 3.36317364
C -1.9078480364 3.0998144012 4.400569866
C -1.2699872764 2.2721512651 5.5280559121
C -0.5648601213 1.1563732411 4.754660478
N -2.5359922433 -0.5668562562 1.5864026187
P -4.0741459918 -0.2191486408 1.302061665
N -5.0943804334 -0.0393804464 2.5450676243
P -5.8414593979 -1.1264647228 3.4404829905
N -7.3997511977 -0.5885741589 3.6089410043
C -8.104779435 0.2304501543 2.5924071045
C -9.5863145123 0.0296265415 2.9410922966
C -9.5662825431 -0.1821627541 4.4627729964
C -8.3129140936 -1.0440366836 4.6767283617
C -4.2881053274 1.335436395 0.3539886682
C -3.1858584126 1.9782061703 -0.5373892369
C -2.9691820065 1.2206173238 -1.9152257068
C -3.883998262 -0.0155817381 -2.0119860116
C -5.2686609007 0.655172469 -1.9472384441
C -5.499452958 1.4248247446 -0.5973926771
C -5.0622543252 2.0276945274 -2.6860559241
C -3.5680174359 2.072202865 -3.0707348564
C -3.9133092692 3.3268767839 -0.8271098952
C -5.2724092159 2.7927580894 -1.3369306935
C -3.1953097993 4.2327367733 -1.8304929535
C -3.0054945128 3.4909810696 -3.1771522602
H -3.6968698866 -0.7739417695 -1.24951554
N -0.8654425817 -1.6627775459 3.4999376295
C -0.2305373009 -2.8478847775 2.8622983489
C -0.9450501512 -4.0508252159 3.4930764764
C -1.3082819868 -3.5401855643 4.8950630016
C -1.7168354905 -2.0827292422 4.6401584014
N -5.1099025689 -1.2813130159 4.9238149394
C -4.5984840957 -0.0691591337 5.6162097677

C	-4.6546370878	-0.4432714587	7.1046562496
C	-4.4779388085	-1.9697664331	7.0955746587
C	-5.2884532415	-2.4087624314	5.8682570969
N	-5.9174895203	-2.7017421627	2.8757387201
C	-4.6944116284	-3.5161140081	2.6486542534
C	-5.0116520433	-4.3347415695	1.3887464353
C	-6.5388019538	-4.4890332033	1.4529067941
C	-6.999028217	-3.1113811077	1.9430151936
H	-6.099522413	0.0237407753	-2.2733777284
H	-4.4729956701	2.028108064	1.1864501541
H	-3.7449550666	-0.5099039244	-2.9813856597
H	-4.0496988343	3.8717500122	0.1164542058
H	-2.2388853385	2.1163332817	-0.0064243871
H	-6.0830422428	3.5260730619	-1.3094831687
H	-5.7482485035	2.2804419081	-3.4988115365
H	-3.4062614486	1.5418051704	-4.0178343682
H	-1.9109472097	0.9918083639	-2.0746895134
H	-6.4659347939	1.2665164488	-0.1118094626
H	-3.5120826319	4.0214787527	-3.9927592528
H	-1.9445646942	3.442106386	-3.4526455275
H	-3.7801998577	5.1509220016	-1.9653764202
H	-2.2293091103	4.5415752765	-1.4106969149
H	-0.281016829	-1.3313380206	-0.1404929852
H	-0.6994009932	0.3662497666	-0.3877138339
H	1.7468349284	-0.5683866399	-1.2167832479
H	1.6133295844	1.0435119236	-0.4929215505
H	2.3407347767	-1.5549413918	0.9713396635
H	3.2838013476	-0.0526793572	0.9569258118
H	1.5338168594	-0.5178214409	3.0105451837
H	1.6246292992	1.1058398688	2.3003360058
H	0.3854805585	1.5236418648	4.3406810186
H	-0.3487071135	0.2715699947	5.3572380283
H	-2.042017152	1.8449289226	6.1799371554
H	-1.1573535169	3.7690818568	3.963677212
H	-0.5802793714	2.8505351486	6.1500908639
H	-2.7453474475	3.7173700512	4.7386704973
H	-3.4123258519	1.801119422	3.4574842527
H	-2.780178993	-2.0091023933	4.3879718462
H	-2.1091993149	-4.1143187937	5.3721299869
H	-0.4303762513	-3.5798093737	5.5513615998
H	-0.3181691502	-4.9475718412	3.5070166571
H	0.8429814237	-2.8595530138	3.0930258057
H	-0.3385542536	-2.8251552646	1.7737930807
H	-1.8551140804	-4.2838111625	2.9271489405
H	-4.9194889682	-3.3415199601	5.4342843927
H	-6.344265407	-2.5577122275	6.1295325258
H	-3.4214017328	-2.229132047	6.9599783056
H	-3.5754848848	0.1357593428	5.2826829707
H	-5.2103543177	0.8083179132	5.3810340577
H	-4.8271108085	-2.4524353222	8.0131525257
H	-3.8908280237	0.0764392678	7.6916896948

H -5.6337085669 -0.1813421174 7.5242534563
H -7.8012229113 1.2807321151 2.6824878554
H -7.8648955545 -0.0978069916 1.5763580236
H -9.9714942635 -0.868457736 2.441722461
H -10.203790125 0.877907276 2.6308900603
H -8.545549246 -2.1152211699 4.5742656299
H -7.8646438585 -0.8884614571 5.6633119449
H -9.4486354388 0.7799115643 4.9763272214
H -10.4707584595 -0.6624444015 4.8474134739
H -7.9627262929 -3.1387497703 2.4598098687
H -7.0867642529 -2.4141141467 1.0963910548
H -6.8120657586 -5.258876457 2.1849548833
H -6.9893074208 -4.758378061 0.4930748221
H -4.7262868192 -3.7707677742 0.491642357
H -4.4785937625 -5.2901861268 1.3685695007
H -3.8070141795 -2.8886699282 2.5246292406
H -4.5193055893 -4.1747965318 3.5097676595
H -1.5402650504 -1.44334585 5.5106436508
H -2.1700439815 2.3952164464 2.3391154433
H -4.5373703037 -1.2848860945 0.5068841467

7a
C 0.6273395251 -0.0117322459 0.2303490239
C 0.9530944681 -0.3846229223 1.7070944842
C 2.3797790235 -0.9449679097 1.6353760208
C 2.06550521 -1.9630988134 0.530956404
C 1.398193297 -1.1038499451 -0.5831484826
C 0.8270781356 -2.748029578 1.0635013459
C 0.0429335746 -1.6401507224 1.8896346352
C -0.7381875267 -0.6957183627 -0.1315953649
C -1.2721673903 -1.1809948776 1.2255977746
C 0.0407871111 -1.7933198156 -0.9498530942
C 0.0918660035 -3.1445690038 -0.2285450536
P -2.7854491785 -2.3022761361 1.2614686895
N -3.9665660839 -1.1294556002 0.8202893563
P -4.7032384323 -0.9848543849 -0.5469616455
N -4.552731322 -2.1300580032 -1.7976031811
C -3.2548308346 -2.2431587801 -2.4745764692
H -0.8915787939 -3.5910095918 -0.0582040651
N -2.892994976 -2.3761484143 2.9595509874
P -4.0430940086 -2.944223533 3.8455568434
N -3.3572974638 -3.4837380851 5.3003397967
C -4.184901419 -3.9953060737 6.3856776847
N -4.9627508225 -4.3057117222 3.3817326892
C -5.9355126594 -4.1484588108 2.2934665053
N -5.2832033122 -1.8010468351 4.0968094884
C -6.4460735767 -2.0877825868 4.9325756018
C -4.2255133009 -5.5702017546 3.2652682825
N -6.3867617929 -0.9945246618 -0.2924030972
C -7.3079913038 -0.6812172796 -1.3792515082
N -4.1922939455 0.4262381486 -1.3534820554

C	-4.6802099008	0.8136201852	-2.6729328557
C	-6.9060412858	-0.6568116261	1.0299511875
C	-3.7097863827	1.5553002117	-0.5611634446
C	-5.1781424718	-3.4422755562	-1.6041803261
C	-4.8999831466	-0.3871343104	4.1127065226
C	-2.0662793752	-2.9590255237	5.7380317623
H	3.1262973203	-0.1970430082	1.3360081145
H	2.7080764259	-1.4115368207	2.573500031
H	-0.219725342	-1.8471965017	-2.0113902819
H	-1.6288051025	-0.2996689468	1.7830075626
H	0.6982577502	-3.8619625444	-0.8008181567
H	0.7659279751	0.4079015637	2.4383626914
H	-0.0912906696	-1.9316007521	2.9343385202
H	0.7762678139	1.0393057492	-0.0346740192
H	2.0625996631	-0.7816113373	-1.3901264688
H	2.8889672017	-2.6007657895	0.1928906691
H	1.0952234602	-3.6022102363	1.6942489048
H	-1.4523722922	-0.1094109918	-0.7140584489
H	-3.3289595747	1.1948977767	0.395016335
H	-2.8964290862	2.0598950195	-1.1008613055
H	-3.874974808	1.3217440857	-3.2220258513
H	-4.9777641278	-0.066720204	-3.2451684453
H	-5.3435272624	-3.9049543061	-2.5861535704
H	-4.5399266543	-4.1097833033	-1.0050963715
H	-6.1408749096	-3.3313048433	-1.1023842017
H	-6.9155260218	-1.0485442467	-2.3298313307
H	-8.268447174	-1.1804387317	-1.1932223149
H	-5.4543276119	-4.1846648325	1.3055204075
H	-6.6690603524	-4.9632981822	2.3548339115
H	-7.8483485605	-1.1978092242	1.1962963377
H	-7.1113885604	0.4228594274	1.1305015955
H	-6.1856693091	-0.9528740853	1.7912642283
H	-4.940712446	-6.4019869875	3.3021866767
H	-3.5227379285	-5.6754980963	4.0938020313
H	-1.5077264069	-3.7533589049	6.2527019479
H	-1.4972824601	-2.6245193244	4.870568954
H	-2.1835501095	-2.1139134801	6.4370394964
H	-3.6194521195	-4.7534030893	6.9449382227
H	-4.5244863498	-0.0702130173	5.1006294515
H	-5.7822159682	0.2210936395	3.8742236269
H	-5.0843816489	-4.470369742	5.9889188273
H	-6.7066258105	-3.1463113353	4.8776435567
H	-7.3038462121	-1.5046358794	4.5694306527
H	-6.2799813167	-1.819761251	5.9889220461
H	-3.6648111748	-5.6309395947	2.3193854329
H	-4.4827338843	-3.2062001801	7.0960567995
H	-4.5070848689	2.2945446295	-0.3746409453
H	-6.459917872	-3.19769917	2.3809516339
H	-4.1384997432	-0.2039113311	3.3553062034
H	-5.5364683395	1.5053286121	-2.6158109514
H	-7.5022615915	0.4006700972	-1.4693059193

H -3.4115781781 -2.6574520262 -3.4795043274
H -2.7863844663 -1.2626868398 -2.5693132079
H -2.5678732393 -2.9024305336 -1.9252735168

7aH+
C 0.6362341832 -0.0042205781 0.0701607278
C 0.9712843908 -0.2393651262 1.5730747655
C 2.3913183535 -0.8170603132 1.5412788265
C 2.0599931301 -1.9308739182 0.5402552275
C 1.388796176 -1.1747416143 -0.6440789141
C 0.8224065721 -2.6540223783 1.1553926125
C 0.0590916016 -1.4688112181 1.8835613754
C -0.7378363072 -0.7013204017 -0.2254883439
C -1.2526653527 -1.0417292474 1.1850600167
C 0.02215795 -1.8827050037 -0.9360337763
C 0.0820514089 -3.1619736084 -0.0931671117
P -2.8008227291 -1.9871538328 1.3871558068
N -4.0378148666 -1.1002766887 0.8459723397
P -4.75812016 -0.962429525 -0.5588192106
N -4.5672412297 -2.1536771202 -1.7258266936
C -3.3066144282 -2.2705681642 -2.4714171132
H -0.8868408318 -3.6256970873 0.1099790022
N -2.8776411459 -2.3715571692 2.9452453192
P -4.0328713831 -2.9742668868 3.8560591673
N -3.2931895372 -3.4869533479 5.2625739824
C -4.1071865229 -3.9772922765 6.3784140688
N -4.8856590823 -4.3242573588 3.3278024532
C -5.8202559339 -4.1871898719 2.2029607371
N -5.2644991645 -1.8425014096 4.0792446885
C -6.4934183455 -2.1837124257 4.8083471556
C -4.2273063861 -5.6380929394 3.3496278019
N -6.4124283203 -0.9744059684 -0.2650868108
C -7.3502076247 -0.7207721205 -1.3644115875
N -4.1839297739 0.4101596897 -1.3454263276
C -4.661870306 0.7859970531 -2.6818439137
C -6.9387127321 -0.6000961697 1.0516623895
C -3.7257829633 1.5616746513 -0.5587901303
C -5.2722465199 -3.4348852089 -1.6066094736
C -4.8790240037 -0.4357731366 4.2605930514
C -1.9422929947 -3.0647874367 5.6450913655
H 3.1364639304 -0.1050377458 1.1667586101
H 2.7268476616 -1.1942685994 2.5149219186
H -0.2474829278 -2.0334366357 -1.9846807193
H -1.5710104708 -0.096307418 1.6563503428
H 0.6927876101 -3.9182828499 -0.6031535923
H 0.7978129694 0.6191929495 2.2285059004
H -0.0710655043 -1.6565837927 2.9515831178
H 0.7882605804 1.0151574914 -0.2920697128
H 2.0447573373 -0.9350307454 -1.483532722
H 2.8738863131 -2.604067753 0.2579581618

H 1.0890924781 -3.4468548997 1.8603913119
H -1.4513528363 -0.1583476557 -0.8490432589
H -3.3535807911 1.2311879919 0.4105647836
H -2.9117037889 2.0608042477 -1.0976893251
H -3.8498001399 1.2873967924 -3.2216887899
H -4.9545183458 -0.097536901 -3.2509419924
H -5.522686547 -3.8021764716 -2.608812762
H -4.6440360325 -4.1907139093 -1.1119225517
H -6.1934311258 -3.3135667378 -1.0363773201
H -6.9478247098 -1.0941786653 -2.3080030715
H -8.2880233857 -1.251040315 -1.1622089587
H -5.3170069358 -4.3656133481 1.2416154395
H -6.6234161751 -4.9254219429 2.3112159465
H -7.8717698003 -1.1498218959 1.2272747102
H -7.1588730726 0.4766513991 1.1107856964
H -6.2200486605 -0.8623698159 1.8271785522
H -4.9958985487 -6.4160329012 3.4180287792
H -3.5666534565 -5.7215250977 4.2128696491
H -1.4226836625 -3.9106136762 6.111636301
H -1.3879458881 -2.7505302692 4.7617634356
H -1.9728029122 -2.2345519771 6.3654898355
H -3.5662873621 -4.780373073 6.8928604546
H -4.6121904486 -0.2161479619 5.3055259323
H -5.7279997456 0.1993848267 3.9840057431
H -5.0527480925 -4.3861527836 6.0168390159
H -6.750475304 -3.2336298695 4.6637145889
H -7.3154673199 -1.5700445667 4.4205334698
H -6.3984882488 -1.9870489509 5.8860687417
H -3.6374265533 -5.8112198964 2.4367546765
H -4.3182853745 -3.1822901249 7.1080948677
H -4.5339813379 2.2912128457 -0.4008644493
H -6.2588684813 -3.1902881303 2.1814855351
H -4.0396623329 -0.1846771462 3.6136450905
H -5.5167110273 1.4753039114 -2.6308978676
H -7.5774153047 0.3497929852 -1.4726306905
H -3.5209656428 -2.6288073463 -3.4850683745
H -2.8075556621 -1.3044027748 -2.5400898911
H -2.6223435787 -2.9833302724 -1.9898442328
H -2.739471604 -3.1640343517 0.607040189

7b

C -0.12223274 0.0380603923 -0.0588850951
N -0.0327567491 -0.0151432101 1.4064715906
C 1.3605868845 -0.1394865701 1.8625872089
C 2.1752438702 -0.2154679566 0.5553517635
C 1.2787643656 0.5036708095 -0.4673212758
P -1.3631708349 0.0372976069 2.4339396426
N -0.8924835751 0.7676440693 3.8754449707
C -1.2072686542 2.1731164506 4.1865518569
C -0.8905815292 2.3173200758 5.7013681548
C -0.5268915798 0.8947474163 6.1807526813

C	-0.0035414022	0.2235142454	4.9052596579
N	-2.6040579216	0.6037007121	1.7104655885
P	-3.6827749484	1.8035999649	1.210187311
N	-4.5623859325	2.26960008	2.6123709625
P	-5.7526736157	1.4468131438	3.2211270988
N	-6.9107897025	2.5520676157	3.7259155522
C	-7.08049921	3.8802068006	3.1017030772
C	-8.5811346069	4.1595999697	3.2601005058
C	-8.9184660157	3.469817825	4.591554777
C	-8.0785466129	2.1811427737	4.5395339936
C	-2.6246948617	3.3730411326	1.1710037335
C	-3.26956408	4.631668904	0.5493193722
C	-3.325821201	4.600021958	-1.0390978896
C	-2.7330949522	3.304525443	-1.6222427374
C	-1.3003195016	3.3830624775	-1.0859946468
C	-1.2492824276	3.4052007492	0.4869323417
C	-0.9508798839	4.9085603965	-1.1209255891
C	-2.2679647398	5.6756796073	-1.4376197331
C	-0.8963124237	4.9354393078	0.4424325288
C	-2.186380677	5.721519801	0.8216956177
C	-2.3502621019	6.7288783179	-0.3242551627
H	-3.2686546165	2.4005325653	-1.3191481188
N	-1.5430686525	-1.5677658761	2.9676292357
C	-1.5712134989	-2.6432813665	1.9447978661
C	-2.8531994889	-3.4378158792	2.2388391672
C	-3.0204426441	-3.2682685999	3.7557600288
C	-2.6032884055	-1.8083953083	3.9773631323
N	-5.3209755808	0.5036758433	4.5454559007
C	-4.3575688568	1.0514018028	5.5238580225
C	-4.7546085362	0.3882131301	6.8495318698
C	-5.3100701203	-0.9713510343	6.3981491164
C	-6.0819533886	-0.6253070031	5.1143638312
N	-6.6049641584	0.3106899887	2.289031153
C	-5.9178700337	-0.9109866866	1.787694735
C	-6.2378216321	-0.9541061841	0.2879145858
C	-7.6192937373	-0.2856929665	0.2198545863
C	-7.4836608799	0.8637787828	1.2272137393
H	-1.5423554976	7.4715380398	-0.3697612923
H	-3.3118581831	7.2583208189	-0.2991766359
H	-0.5107472788	2.7369865727	0.9372089671
H	-2.7357085716	3.352159952	-2.7211067262
H	-2.4688698119	3.5979943482	2.2331356786
H	-2.3585961426	6.0331130332	-2.4686629117
H	-4.3312675349	4.8259917361	-1.4098424217
H	-0.0691941446	5.2363013225	-1.6793729815
H	0.0236048766	5.2767163349	0.9268752155
H	-2.2057692567	6.1194308337	1.8410731681
H	-4.2425724467	4.8724338909	0.988117111
H	-0.5909462481	2.7014230252	-1.5646420255
H	-0.3330962127	-0.9562016323	-0.48966245
H	-0.9236711707	0.7094804545	-0.3692226628

H 1.522341352 0.2552617748 -1.5058658072
H 1.3564017998 1.5919222749 -0.3471828005
H 2.3088710762 -1.2633347574 0.2573622328
H 3.1694087035 0.2321834512 0.6588829636
H 1.5082588362 -1.0261789806 2.494273009
H 1.6473493937 0.7411577742 2.4546481175
H 1.0472576715 0.5108285223 4.7290335592
H -0.0528025131 -0.8675766918 4.9191126671
H -1.4205345801 0.363906408 6.5296671179
H -0.0419420873 2.9951514692 5.8484092137
H 0.2070877739 0.8936537902 6.9943541967
H -1.7396927888 2.7353833543 6.252152489
H -2.2558026052 2.3738262822 3.9544455052
H -3.455116068 -1.1347366285 3.8204642318
H -4.0400776771 -3.4638499366 4.1059711182
H -2.3428672398 -3.9464854194 4.2910243176
H -2.7836060363 -4.4839720677 1.9210182136
H -0.6793976301 -3.2778136737 2.0511944023
H -1.5716228653 -2.2358978044 0.9298698162
H -3.7007506699 -2.9781199376 1.7164150457
H -6.1157222808 -1.4681719481 4.417997242
H -7.1203463709 -0.3490244422 5.3445408128
H -4.4840249655 -1.6543277256 6.165425435
H -3.3379221717 0.7784070911 5.2333180058
H -4.4109148608 2.1445889127 5.5632997877
H -5.9466481498 -1.4552367738 7.1466155759
H -3.9110562588 0.303951059 7.5436942905
H -5.5419513991 0.9714585243 7.3446022028
H -6.476620199 4.6309538305 3.631267996
H -6.7478122245 3.8733971611 2.0607992174
H -9.1385327611 3.6876182836 2.4402122437
H -8.8138008501 5.2299572787 3.2558461228
H -8.6426096997 1.3569509411 4.0741500423
H -7.7743127035 1.8500462819 5.5399935868
H -8.5895382883 4.0969145585 5.4302072918
H -9.986519205 3.265904492 4.7227444017
H -8.4450025778 1.1775749748 1.65038718
H -7.0217249544 1.7351185024 0.7409373615
H -8.3996675516 -0.9861201882 0.5464735687
H -7.881745823 0.0727285742 -0.7811117252
H -6.227992209 -1.9740962021 -0.1126955222
H -4.8423642112 -0.871171022 1.9785611105
H -6.32745238 -1.7968111525 2.2960341881
H -2.2198882852 -1.6310062493 4.9890772705
H -0.5926931101 2.8658182197 3.5933253724
H -5.5008507142 -0.3541700843 -0.2577485505

7bH^+
C 0.1409245349 0.1269566525 0.2072313192
N 0.047701786 0.0050384014 1.6742575269
C 1.3852042944 -0.1622809928 2.2869900713

C	2.3553079848	-0.0904029409	1.0888017053
C	1.5598031143	0.6650006548	0.0097557784
P	-1.3608196366	-0.0683651376	2.5502246982
N	-1.1378878459	0.655763771	4.0330347125
C	-1.072444626	2.1343693711	4.1424837032
C	-0.2115637149	2.379798086	5.3903984879
C	-0.4878954261	1.1411461154	6.2547583484
C	-0.5410529754	0.0045445009	5.2260233717
N	-2.5259024879	0.556016868	1.6742404578
P	-3.4825854993	1.8362383973	1.5223523809
N	-4.4588383444	2.2308288913	2.7530664822
P	-5.7264291496	1.4272160902	3.3008340705
N	-6.7813858942	2.5921241684	3.8262438532
C	-6.8342514907	3.9744017581	3.2883819862
C	-8.2595566514	4.4314850775	3.6278393724
C	-8.5726387438	3.6755491103	4.9283544968
C	-7.9441775563	2.294508495	4.6873774192
C	-2.6197262571	3.4156781201	1.1621676095
C	-3.4180453504	4.484194479	0.3773502806
C	-3.4593358323	4.2165951477	-1.1892129383
C	-2.704261274	2.9392228872	-1.6065522944
C	-1.2967322432	3.2583042464	-1.0989291319
C	-1.2551417467	3.4958311371	0.4534605442
C	-1.1364428571	4.7948876274	-1.3415235963
C	-2.5352223287	5.3469945651	-1.7428796721
C	-1.0923737025	5.0392432371	0.2026825173
C	-2.466954031	5.715006263	0.4875957507
C	-2.7462949217	6.5253087817	-0.7839962528
H	-3.1130690793	2.0030961975	-1.2157376627
N	-1.6160256403	-1.6702673188	2.9689746375
C	-1.3450047581	-2.7521941005	1.9931983807
C	-2.7362752036	-3.2890469622	1.6415313901
C	-3.4507116406	-3.2518474272	3.0030311845
C	-2.8611296382	-2.0070737959	3.7122866329
N	-5.3159950903	0.3961500725	4.5400520477
C	-4.3915869998	0.8867278339	5.5963564871
C	-4.8908257512	0.1976096739	6.8722480001
C	-5.4535571567	-1.1324998153	6.3494978028
C	-6.139060347	-0.7362814925	5.0328798541
N	-6.5682381985	0.4404177908	2.2400561217
C	-5.9941653109	-0.8076900753	1.6668130541
C	-6.4230195834	-0.7820890171	0.1940460932
C	-7.7651340788	-0.0365605027	0.2446842514
C	-7.5023260285	1.0732700102	1.2702348658
H	-2.0311422545	7.3419699709	-0.9395763307
H	-3.7617618542	6.9385782459	-0.8188186253
H	-0.4456686241	2.9845553777	0.9801137785
H	-2.7049688726	2.8531772162	-2.7009019182
H	-2.4850584113	3.8054218808	2.1811340883
H	-2.6655056869	5.5448201214	-2.8102726485
H	-4.4819221564	4.2679522345	-1.5753479646

H	-0.2989524946	5.1467978553	-1.9476942968
H	-0.2221232136	5.5443849868	0.6282097105
H	-2.537878009	6.2445753117	1.442100378
H	-4.4129154783	4.6699212974	0.7923413342
H	-0.5068440613	2.6103132719	-1.4867645478
H	0.0299844992	-0.8524028091	-0.2852251897
H	-0.6451535571	0.780999269	-0.1674049607
H	1.9389789546	0.4909379088	-1.0017888687
H	1.5775804315	1.7455505607	0.2010628866
H	2.5887588456	-1.101943497	0.735921703
H	3.2994389169	0.3949496726	1.3526908621
H	1.4696837189	-1.1140353853	2.8266993944
H	1.5672181011	0.6482213909	3.0042672598
H	0.465217497	-0.3793735065	5.0135356746
H	-1.1546353889	-0.8355663795	5.5587892854
H	-1.4576812032	1.2399398941	6.7581320484
H	0.8497953707	2.4160329796	5.1154247745
H	0.2733922593	0.967513345	7.0210454977
H	-0.4618471588	3.3219266928	5.8872109532
H	-2.0841117979	2.5412913774	4.2613206904
H	-3.5543574049	-1.1617922156	3.691302231
H	-4.5397100632	-3.1920401894	2.9132426376
H	-3.2179751546	-4.158480086	3.5731219205
H	-2.709771017	-4.2898725151	1.1989697463
H	-0.738520525	-3.5287426125	2.4793392325
H	-0.7844941589	-2.3808078236	1.1340053593
H	-3.2201073553	-2.6075117967	0.930994989
H	-6.1434897508	-1.5557753666	4.3096385385
H	-7.1803012199	-0.4359344615	5.2028395583
H	-4.6354274961	-1.8347174353	6.149562413
H	-3.3663168751	0.5925331823	5.3476904137
H	-4.4191441762	1.9789084562	5.6663545709
H	-6.1472424235	-1.6154716597	7.0439766868
H	-4.0966334368	0.0688270013	7.6144479261
H	-5.6895875013	0.7907351519	7.3346363891
H	-6.0776582924	4.5968116023	3.7820232336
H	-6.6277275937	3.9925112643	2.2144097497
H	-8.9527453751	4.1204122999	2.8359121845
H	-8.3331417774	5.5182219788	3.7315993543
H	-8.6505113568	1.6181517704	4.1820604953
H	-7.6257915076	1.8148626929	5.6189399951
H	-8.0808175928	4.1659087052	5.7774035513
H	-9.6422499314	3.6115012761	5.1486361904
H	-8.4107195997	1.4130571748	1.7768773002
H	-7.0407571635	1.9444028976	0.7817494835
H	-8.5553200991	-0.7050892965	0.6077419657
H	-8.0790480863	0.3630411282	-0.7241262798
H	-6.5001101381	-1.7866768039	-0.2330497409
H	-4.908618665	-0.8482252014	1.7909348929
H	-6.4278789113	-1.6778613234	2.1762084696
H	-2.6277989802	-2.2210931677	4.7617878337

H -0.6281488448 2.5793944931 3.2455421126
H -5.6961412365 -0.2169768838 -0.4029663378
H -4.2774717127 1.525559936 0.4038644251

8a
C -0.0089974056 -0.2010919304 0.2932022502
C 0.1592402797 -0.471660634 1.797520541
C 1.6442083372 -0.5346184971 2.1929182492
C 2.4186722803 -1.5576519546 1.3406795591
C 2.24624227 -1.3162240701 -0.1740667862
C 0.755486798 -1.2453711426 -0.5359014662
H 2.0921764854 0.4596514001 2.0596662201
P 3.2458304941 0.2041344054 -0.7182416806
N 2.8056458772 0.3047928472 -2.3615832146
P 1.9798356252 1.1017760326 -3.3984317047
N 2.8839208677 1.2123141078 -4.8392893154
C 4.3285938679 1.383564307 -4.7434223993
N 4.7443081546 -0.6027240312 -0.7135716269
P 6.1928554796 -0.0843428386 -0.9476846242
N 6.6524958282 -0.2660230874 -2.5801589448
C 6.0338393037 -1.36056716 -3.333530973
N 6.6489868404 1.5356499684 -0.6635821075
C 6.5402192408 1.9957983314 0.7274832427
N 7.2301673467 -0.9088315321 0.1117482182
C 6.8337971109 -2.2026806699 0.6624708882
C 6.1992984296 2.56373118 -1.6088061056
N 0.5346452556 0.4420619534 -4.009250147
C 0.6237490658 -0.8460281455 -4.6989017799
N 1.4217887948 2.638236304 -2.8921377316
C 2.202985337 3.3696342573 -1.8950872544
C -0.7480820021 0.6249113678 -3.3286462978
C 0.7628830782 3.5314183663 -3.8446303833
C 2.3292969777 1.7015194121 -6.0959387536
C 8.6705989972 -0.6847249973 0.1138434224
C 8.0060545458 0.0454885196 -3.0321559957
H 0.6521971246 -1.0495169687 -1.6067854674
H 0.3691412739 0.8044128858 0.0579741203
H -1.0743552215 -0.20798904 0.0223397876
H -0.3593178169 0.2973290401 2.3867885984
H -0.3206690904 -1.4330192358 2.0415370372
H 1.7407963859 -0.7862837729 3.2583152283
H 3.4836573956 -1.5441547615 1.6055769143
H 2.0486275968 -2.5683839875 1.5787098931
H 2.7102671251 -2.1440938801 -0.7283306245
H 0.3038142344 -2.2345488249 -0.3527588015
H 2.6244606057 2.6831210648 -1.1578790548
H 3.0206745002 3.9519658658 -2.3548777951
H 1.5423729519 4.0728686899 -1.3705507671
H 6.8202359959 3.4592972996 -1.4763593164
H 5.1476507568 2.8426793719 -1.4461330079
H 6.3073683193 2.2142158739 -2.6354763209

H 5.5025490724 2.2501786473 0.992914251
H 7.165668219 2.8885735262 0.8558720761
H 6.8934552424 1.2204185101 1.4098211975
H 1.4860089707 4.1449551055 -4.4074022335
H 0.0955081584 4.2122505513 -3.2990674598
H 0.1612758131 2.9644529398 -4.5573000524
H 5.7468796544 -2.2857526814 0.6531721249
H -0.7222682479 1.509466867 -2.6918547825
H 7.2622088317 -3.0395267469 0.0857651598
H 7.1931146102 -2.2824684032 1.698119693
H -1.5433944694 0.7474105539 -4.077738599
H 9.2076051501 -1.3984649916 -0.5325295795
H 8.9000862545 0.3307649869 -0.2146749636
H 9.0540874565 -0.8056158292 1.1366023763
H 4.9890319131 -1.4653241983 -3.040063469
H 0.5269325409 -1.6899987502 -3.9983247463
H 6.5558283456 -2.3192988869 -3.1741782583
H -0.1835879378 -0.9169353685 -5.4405318735
H 6.077859536 -1.121359227 -4.4036165497
H 1.5814683797 -0.935948763 -5.2149689203
H 8.6900654441 -0.8135096631 -2.9334521643
H 7.9754847828 0.3298581671 -4.092820723
H 8.4156719695 0.8848973057 -2.4667681226
H 4.6941935925 0.9429560403 -3.8175982897
H 2.7985950482 1.1650624588 -6.933172635
H 4.8152971732 0.8756739437 -5.5879617864
H 4.6200339015 2.4480736241 -4.7765243688
H 2.5139513646 2.7785407366 -6.2431465044
H 1.2537326545 1.5231171404 -6.1362834535
H -1.002566025 -0.2405683799 -2.7004383424

8aH^+
C 0.083353284 -0.0158300127 0.1880747826
C 0.2349933131 -0.1837288227 1.7083204523
C 1.7092054143 -0.338654131 2.1154027829
C 2.4039142566 -1.4773895137 1.3451904905
C 2.2179478931 -1.3748433776 -0.1899539201
C 0.7352298684 -1.1895162888 -0.5585141441
H 2.2323959544 0.6123123681 1.941019729
P 3.334133895 -0.1000625438 -0.9097158453
N 2.8463053516 0.4501743702 -2.3216967606
P 1.9401662402 1.1006613442 -3.4222151931
N 2.8449704152 1.1388961902 -4.8395224781
C 4.2965967295 1.3196576862 -4.7824331779
N 4.7967013893 -0.7721341533 -0.8692925434
P 6.2534070937 -0.1462097265 -0.9799090261
N 6.7502679401 -0.1662249844 -2.5925995631
C 6.3314229426 -1.3047568476 -3.4242001645
N 6.5130863763 1.4542864831 -0.5399282332
C 6.508373538 1.8214257201 0.882927014
N 7.2272815506 -1.0120797293 0.0671131486

C	6.84042913	-2.3275489448	0.588808858
C	6.0608914547	2.5495173111	-1.4071036039
N	0.5424546001	0.3266735076	-3.9095722641
C	0.6253088405	-0.9400250024	-4.6459389652
N	1.3739742708	2.606135663	-2.9117516595
C	2.0850178002	3.3576028189	-1.8761374901
C	-0.7817311125	0.5774696511	-3.3309732028
C	0.6922218497	3.4788993129	-3.8770219637
C	2.261763596	1.5591729924	-6.1178227425
C	8.667289481	-0.7457181338	0.1367856207
C	8.0688659781	0.3553247825	-2.9780355485
H	0.6325827018	-1.0780139633	-1.6411622244
H	0.5427160892	0.9328721902	-0.1306696452
H	-0.9775252713	0.0520982252	-0.0824994128
H	-0.2137905852	0.6678034544	2.2340991402
H	-0.3230467287	-1.0775525841	2.0238180633
H	1.7859926608	-0.5332044248	3.1920386465
H	3.469824748	-1.5250424124	1.599906172
H	1.9671604158	-2.4351579462	1.6604829153
H	2.5933784242	-2.2976557068	-0.6539378203
H	0.2096191418	-2.1164006499	-0.2880356104
H	2.4684177079	2.6879299856	-1.1074594233
H	2.9218052344	3.9437832147	-2.2870517194
H	1.3828226566	4.0515115691	-1.4002871431
H	6.7388648032	3.4029288334	-1.2892014336
H	5.0449899161	2.8772515279	-1.1429256937
H	6.0642902284	2.2413639836	-2.4521022933
H	5.5005803092	2.1047217294	1.2215395985
H	7.1765153884	2.6772377355	1.0326349563
H	6.8616025644	0.9902606008	1.4939264125
H	1.4012371259	4.1240039505	-4.416476796
H	-0.0189336204	4.1187953865	-3.3421970128
H	0.1328964194	2.8894784061	-4.6051376143
H	5.7593488562	-2.4482859946	0.5306348954
H	-0.7545974756	1.4363989819	-2.6613234876
H	7.3229235799	-3.1374945205	0.0230433822
H	7.1539614549	-2.4025368052	1.6372571969
H	-1.5038524788	0.7717179476	-4.1345307135
H	9.2396593211	-1.4322528502	-0.5032336106
H	8.8856824177	0.281612534	-0.160762138
H	9.0068495287	-0.8781586398	1.1709497122
H	5.3103998819	-1.5955264575	-3.1774495552
H	0.4139390081	-1.7918606171	-3.9844884363
H	6.9957343658	-2.1726226138	-3.2953138367
H	-0.1125435585	-0.9415255388	-5.4577741973
H	6.3653489423	-1.0000272864	-4.475833582
H	1.6186536211	-1.069897786	-5.0776268287
H	8.8520086805	-0.4131627808	-2.9058141174
H	8.0220195312	0.6985514605	-4.0181484208
H	8.348513382	1.2028730499	-2.3505043169
H	4.6951399965	0.8941550245	-3.8631347588

H 2.7285518693 0.9865711653 -6.9283050484
H 4.753403551 0.799317115 -5.6329658456
H 4.5776292548 2.383020352 -4.8431976728
H 2.4298887717 2.6283145359 -6.3121159324
H 1.1890316585 1.3627838946 -6.1378389286
H -1.1283077392 -0.2948384691 -2.7625456838
H 3.3212346656 0.9867649812 -0.0021297296

8b
C 0.1635063753 -0.034851883 0.1257917141
N 0.0284207891 -0.0714596661 1.5984601238
C 1.3733090936 -0.1062755662 2.2320640558
C 2.3623030143 -0.1229933345 1.0534521807
C 1.5684911866 0.5392366501 -0.0831718745
P -1.183439037 -1.0575873009 2.2469135122
N -2.5454284883 -0.6793350739 1.3395790194
C -3.24305335 -1.5998871292 0.4285622071
C -4.5863045393 -0.8976532438 0.1915846301
C -4.2022358566 0.5910332538 0.212475573
C -3.142103362 0.6658298436 1.3262174544
N -0.8196764747 -2.5760440098 2.2138683219
P -1.4314412694 -3.9271749861 3.0892642665
N -0.2893164319 -4.2862322648 4.2892306371
P 1.2656946516 -4.2723028973 4.4534383285
N 1.8401140442 -2.7601837052 4.9428005619
C 0.9514351227 -1.8702162927 5.7270049695
C 1.8981882413 -0.7962486362 6.3217041994
C 3.2406901921 -1.0094284538 5.5960616936
C 3.2340140279 -2.5181238438 5.3352736192
C -0.9698772377 -5.2392814114 1.7837849215
C -1.1380908703 -6.6772909347 2.3122726184
C -2.6119310866 -7.1096536813 2.4078929742
C -3.3376013119 -6.931511449 1.0649543308
C -3.2081202027 -5.4863837153 0.558016682
C -1.7319017923 -5.0655791907 0.4564877762
N -1.4116673207 -0.3356193307 3.7558378724
C -2.4245472891 -0.9066964844 4.6698047065
C -2.4316170937 0.0726575261 5.8494831662
C -2.1619361507 1.4237393752 5.1695433862
C -1.1120011403 1.0690075763 4.1057836569
N 2.3648907857 -4.5912066508 3.199333294
C 2.516314922 -5.9675766856 2.6700176452
C 2.8005233696 -5.7866560432 1.1679945952
C 3.3977743111 -4.3735843018 1.0827040957
C 2.5868096362 -3.5971974822 2.1256301231
N 1.6579739096 -5.4393781414 5.6058144329
C 0.7167862393 -6.4264364848 6.1389168151
C 1.4293692112 -7.7640175918 5.8775663067
C 2.9323025329 -7.4277206688 6.0543334387
C 3.0131445725 -5.8732098804 5.9578480832
H -4.3957863701 -7.2109414928 1.1620592898

H	-2.6736955517	-8.1560525776	2.7378801096
H	-0.6561649248	-6.7772521658	3.2923533845
H	-0.6138634419	-7.3673518804	1.6292886074
H	0.0920589881	-5.0473924986	1.5927056711
H	-1.6398313348	-4.0289913581	0.1132731128
H	-1.2473476596	-5.6932714456	-0.310752803
H	-3.7355656652	-4.8200904016	1.2551455266
H	-3.6986889423	-5.3779573931	-0.4196591639
H	-2.8967054825	-7.6149623936	0.3214002752
H	0.4248334368	-2.4183874919	6.5202499354
H	1.092427869	-8.5620813822	6.5486346632
H	0.2019451738	-1.4419917802	5.0618083486
H	3.252063013	-0.4734044485	4.6391875668
H	1.503022186	0.2166424634	6.1903693956
H	2.0290250714	-0.9588450404	7.3983930653
H	4.1054299281	-0.6814038734	6.1834917481
H	3.9211146064	-2.8333394191	4.5448247848
H	3.5073039097	-3.0634611407	6.2556489142
H	3.3089513842	-5.4497120835	6.9300198066
H	3.3045817712	-7.768273806	7.027206723
H	3.5460225791	-7.9107097701	5.286497369
H	1.2265303453	-8.0833874224	4.8487091968
H	1.6211170288	-6.5726822813	2.8428342611
H	-0.2533333464	-6.3234587805	5.6519270689
H	3.3558816442	-6.4694836446	3.1707077087
H	3.4652231867	-6.5641652292	0.7762857479
H	1.863896767	-5.8298277629	0.6003117062
H	3.3194313706	-3.9324840946	0.0828558632
H	4.4590261694	-4.3901931835	1.3643431838
H	1.6344995952	-3.2361699658	1.7125220906
H	3.1293201602	-2.730591306	2.5152240393
H	2.6014704562	-1.1564770593	0.7780293165
H	3.3019291947	0.389136265	1.287551396
H	1.5117576605	0.7942207413	2.8461492299
H	1.5525696326	1.6295391673	0.0441551198
H	1.4851855682	-0.9801740364	2.881958926
H	1.9667168855	0.3171685198	-1.0790523042
H	0.0944714104	-1.0426270596	-0.314069515
H	-0.6248285897	0.5834644533	-0.3169011291
H	-2.1595242173	-1.9277182689	4.9611821154
H	-3.4135682342	-0.9472269371	4.1852369539
H	-2.388192762	1.4400170687	1.1288974935
H	-3.5988488456	0.8932714892	2.3014446129
H	-5.0496629353	1.2601732642	0.3955147878
H	-3.7536220449	0.8725445118	-0.7490772072
H	-5.2732418922	-1.1233259696	1.0174232316
H	-5.0640318924	-1.2088862694	-0.7433248686
H	-3.3509439481	-2.5854573029	0.8825685543
H	-2.6927017709	-1.7232981659	-0.5185091668
H	-3.3713940179	0.0441278289	6.4104736234
H	-3.0781818597	1.7889931813	4.6871566683

H -1.8114923776 2.1995099116 5.8590942316
H -1.6150449473 -0.1716105262 6.5412756
H -1.1498909372 1.7236086342 3.2302397958
H -0.0983988695 1.1422568143 4.5269601727
H 0.5776573925 -6.2669689539 7.2211844878
H 3.7353400346 -5.5236583928 5.2153402761
H -3.1171374838 -6.5041711539 3.1730420364

8bH+
C 0.1295969285 0.4761191742 0.2474282629
N -0.0011559339 0.3078952133 1.7170331712
C 1.3262113669 0.2359597236 2.3694242981
C 2.2932735887 0.0287056027 1.1947625001
C 1.6169196753 0.7961505657 0.0484526458
P -1.3903823224 0.6935134814 2.5637657436
N -2.6862411291 0.5389240152 1.6649297303
P -3.2529605282 -0.4716091787 0.5498896035
N -3.1117963653 -2.0527327893 0.7956686263
P -2.2894732594 -3.3221300427 0.3286347397
N -1.1329402644 -3.897134237 1.3882309757
C 0.1127745639 -3.1401443792 1.6719596843
C 0.3314108832 -3.321286499 3.1798433437
C -0.3138639483 -4.6847826666 3.467644303
C -1.5669868707 -4.6641579796 2.5836575032
N -1.5092439357 -0.2254535913 3.9418596819
C -2.6623241318 -1.1335519913 4.1864710826
C -2.3976100269 -1.6775417744 5.5979278063
C -1.6269737385 -0.5381351613 6.2833684529
C -0.6990537733 -0.043713377 5.1671340046
N -1.169534601 2.2507790599 3.1187061114
C -0.5559245456 3.2903357208 2.2483549594
C -1.153010307 4.6205232508 2.7533472432
C -1.6385741522 4.2980347645 4.1757628556
C -2.1633109641 2.8684346929 4.0271957259
C -5.0441577534 -0.1093710439 0.3587911614
C -5.6335590766 -0.7809500571 -0.9053948876
C -5.3038782714 -0.022959551 -2.2042243647
C -5.6821390521 1.4630453298 -2.1092443013
C -5.022961544 2.1288812345 -0.8914423618
C -5.3865541153 1.3951785091 0.4090689751
N -3.3852409118 -4.5544019275 0.1488583247
C -4.7494577464 -4.5874009953 0.7206247923
C -5.3573314608 -5.8367185814 0.0729479851
C -4.1582296521 -6.7941167293 -0.0206126192
C -2.9938920987 -5.8674375098 -0.4100794297
N -1.4308220546 -3.0898186607 -1.0889935057
C -0.2293541594 -3.8793085239 -1.4785775796
C -0.4619657852 -4.221939679 -2.9541504596
C -1.2431617009 -3.004147022 -3.4663697131
C -2.1918074887 -2.6831733851 -2.3028079991
H -4.2308182783 -0.1045956884 -2.4290008084

H	-4.8959235013	1.8602102934	1.2700675773
H	-5.4651174336	-0.601019744	1.2481853334
H	-5.3033087304	-1.8247413938	-0.9746475564
H	-6.7253299276	-0.8079818867	-0.7825568638
H	-5.8278623312	-0.4981014354	-3.0429830223
H	-5.3993072339	1.984699085	-3.0319288469
H	-6.7749038617	1.552601971	-2.0191112963
H	-3.9300728995	2.1365907748	-1.0225146248
H	-5.3315424268	3.1790158534	-0.8184824657
H	-6.4708951648	1.4798539518	0.5710050819
H	-0.5267445605	1.2659140919	-0.1305581703
H	-2.4051884945	4.9907421341	4.5354980363
H	-0.1445011007	-0.458103173	-0.26386687
H	2.3593332093	-1.0382001394	0.9473026032
H	1.9763221525	0.5012220175	-0.9421948741
H	1.7876326965	1.8734842951	0.1637658323
H	3.3023426613	0.3849070182	1.4213413263
H	1.3573066916	-0.5903929482	3.0860364171
H	1.5527017424	1.1652583273	2.9111671445
H	-0.7899584867	3.1307767709	1.1893154636
H	-2.0063392783	4.9069000192	2.1273594733
H	-0.4262138773	5.4374230571	2.7189110803
H	-0.8009687862	4.3161880463	4.883914533
H	-0.3892070301	0.9984940501	5.2820813476
H	-2.2107804749	2.3219144422	4.9737158804
H	0.2108963439	-0.6587992078	5.1243912875
H	-1.0765462484	-0.8583109415	7.1731028593
H	-2.3179268668	0.2609594175	6.5798896539
H	-3.324844811	-1.945559929	6.1131358753
H	-1.770254581	-2.5767571797	5.5489523384
H	-3.6020776853	-0.5687699173	4.1478657094
H	-2.7160563433	-1.9148606877	3.4253298998
H	-0.1977654754	-2.5292207882	3.7187433652
H	1.3895541437	-3.2762897524	3.4566426281
H	0.9437259569	-3.5674299909	1.0960031884
H	0.354435295	-5.4961322492	3.1548549539
H	0.0076043668	-2.0873917875	1.401081337
H	-0.5578474709	-4.8368635063	4.523621347
H	-2.3971688814	-4.1634599561	3.1025055555
H	-1.90429065	-5.6653884886	2.2986679788
H	-3.1252792557	-3.2591087311	-2.3821629476
H	-2.0336640799	-6.2088707005	-0.0043427186
H	-2.8878431171	-5.7944681773	-1.5012969464
H	-4.3018474844	-7.604848699	-0.7408248497
H	-3.9640732507	-7.2464565961	0.9598825585
H	-5.7298438459	-5.5945077702	-0.9303261095
H	-6.189505746	-6.2438889425	0.6549156854
H	-5.2868427473	-3.6665245022	0.4864813133
H	-4.71477389	-4.6903837161	1.8154618646
H	-1.7848950306	-3.1918794658	-4.398049878
H	-1.0736272556	-5.1286298053	-3.039785885

H 0.4742760241 -4.394857783 -3.49351321
H -0.1120355279 -4.7605782711 -0.843764308
H 0.6667966395 -3.2557690398 -1.3599450591
H -3.1679989934 2.8629181135 3.5780828439
H 0.5344573211 3.2615691042 2.3609077066
H -2.6425917334 -0.1461141279 -0.6827662112
H -0.5601621158 -2.1627651756 -3.6376704239
H -2.4546568687 -1.6232018936 -2.265150154

9a
C 0.6273395251 -0.0117322459 0.2303490239
C 0.9530944681 -0.3846229223 1.7070944842
C 2.3797790235 -0.9449679097 1.6353760208
C 2.06550521 -1.9630988134 0.530956404
C 1.398193297 -1.1038499451 -0.5831484826
C 0.8270781356 -2.748029578 1.0635013459
C 0.0429335746 -1.6401507224 1.8896346352
C -0.7381875267 -0.6957183627 -0.1315953649
C -1.2721673903 -1.1809948776 1.2255977746
C 0.0407871111 -1.7933198156 -0.9498530942
C 0.0918660035 -3.1445690038 -0.2285450536
P -2.7854491785 -2.3022761361 1.2614686895
N -3.9665660839 -1.1294556002 0.8202893563
P -4.7032384323 -0.9848543849 -0.5469616455
N -4.552731322 -2.1300580032 -1.7976031811
C -3.2548308346 -2.2431587801 -2.4745764692
H -0.8915787939 -3.5910095918 -0.0582040651
N -2.892994976 -2.3761484143 2.9595509874
P -4.0430940086 -2.944223533 3.8455568434
N -3.3572974638 -3.4837380851 5.3003397967
C -4.184901419 -3.9953060737 6.3856776847
N -4.9627508225 -4.3057117222 3.3817326892
C -5.9355126594 -4.1484588108 2.2934665053
N -5.2832033122 -1.8010468351 4.0968094884
C -6.4460735767 -2.0877825868 4.9325756018
C -4.2255133009 -5.5702017546 3.2652682825
N -6.3867617929 -0.9945246618 -0.2924030972
C -7.3079913038 -0.6812172796 -1.3792515082
N -4.1922939455 0.4262381486 -1.3534820554
C -4.6802099008 0.8136201852 -2.6729328557
C -6.9060412858 -0.6568116261 1.0299511875
C -3.7097863827 1.5553002117 -0.5611634446
C -5.1781424718 -3.4422755562 -1.6041803261
C -4.8999831466 -0.3871343104 4.1127065226
C -2.0662793752 -2.9590255237 5.7380317623
H 3.1262973203 -0.1970430082 1.3360081145
H 2.7080764259 -1.4115368207 2.573500031
H -0.219725342 -1.8471965017 -2.0113902819
H -1.6288051025 -0.2996689468 1.7830075626
H 0.6982577502 -3.8619625444 -0.8008181567
H 0.7659279751 0.4079015637 2.4383626914

H -0.0912906696 -1.9316007521 2.9343385202
H 0.7762678139 1.0393057492 -0.0346740192
H 2.0625996631 -0.7816113373 -1.3901264688
H 2.8889672017 -2.6007657895 0.1928906691
H 1.0952234602 -3.6022102363 1.6942489048
H -1.4523722922 -0.1094109918 -0.7140584489
H -3.3289595747 1.1948977767 0.395016335
H -2.8964290862 2.0598950195 -1.1008613055
H -3.874974808 1.3217440857 -3.2220258513
H -4.9777641278 -0.066720204 -3.2451684453
H -5.3435272624 -3.9049543061 -2.5861535704
H -4.5399266543 -4.1097833033 -1.0050963715
H -6.1408749096 -3.3313048433 -1.1023842017
H -6.9155260218 -1.0485442467 -2.3298313307
H -8.268447174 -1.1804387317 -1.1932223149
H -5.4543276119 -4.1846648325 1.3055204075
H -6.6690603524 -4.9632981822 2.3548339115
H -7.8483485605 -1.1978092242 1.1962963377
H -7.1113885604 0.4228594274 1.1305015955
H -6.1856693091 -0.9528740853 1.7912642283
H -4.940712446 -6.4019869875 3.3021866767
H -3.5227379285 -5.6754980963 4.0938020313
H -1.5077264069 -3.7533589049 6.2527019479
H -1.4972824601 -2.6245193244 4.870568954
H -2.1835501095 -2.1139134801 6.4370394964
H -3.6194521195 -4.7534030893 6.9449382227
H -4.5244863498 -0.0702130173 5.1006294515
H -5.7822159682 0.2210936395 3.8742236269
H -5.0843816489 -4.470369742 5.9889188273
H -6.7066258105 -3.1463113353 4.8776435567
H -7.3038462121 -1.5046358794 4.5694306527
H -6.2799813167 -1.819761251 5.9889220461
H -3.6648111748 -5.6309395947 2.3193854329
H -4.4827338843 -3.2062001801 7.0960567995
H -4.5070848689 2.2945446295 -0.3746409453
H -6.459917872 -3.19769917 2.3809516339
H -4.1384997432 -0.2039113311 3.3553062034
H -5.5364683395 1.5053286121 -2.6158109514
H -7.5022615915 0.4006700972 -1.4693059193
H -3.4115781781 -2.6574520262 -3.4795043274
H -2.7863844663 -1.2626868398 -2.5693132079
H -2.5678732393 -2.9024305336 -1.9252735168

9aH^+
C 0.6362341832 -0.0042205781 0.0701607278
C 0.9712843908 -0.2393651262 1.5730747655
C 2.3913183535 -0.8170603132 1.5412788265
C 2.0599931301 -1.9308739182 0.5402552275
C 1.388796176 -1.1747416143 -0.6440789141
C 0.8224065721 -2.6540223783 1.1553926125
C 0.0590916016 -1.4688112181 1.8835613754

C	-0.7378363072	-0.7013204017	-0.2254883439
C	-1.2526653527	-1.0417292474	1.1850600167
C	0.02215795	-1.8827050037	-0.9360337763
C	0.0820514089	-3.1619736084	-0.0931671117
P	-2.8008227291	-1.9871538328	1.3871558068
N	-4.0378148666	-1.1002766887	0.8459723397
P	-4.75812016	-0.962429525	-0.5588192106
N	-4.5672412297	-2.1536771202	-1.7258266936
C	-3.3066144282	-2.2705681642	-2.4714171132
H	-0.8868408318	-3.6256970873	0.1099790022
N	-2.8776411459	-2.3715571692	2.9452453192
P	-4.0328713831	-2.9742668868	3.8560591673
N	-3.2931895372	-3.4869533479	5.2625739824
C	-4.1071865229	-3.9772922765	6.3784140688
N	-4.8856590823	-4.3242573588	3.3278024532
C	-5.8202559339	-4.1871898719	2.2029607371
N	-5.2644991645	-1.8425014096	4.0792446885
C	-6.4934183455	-2.1837124257	4.8083471556
C	-4.2273063861	-5.6380929394	3.3496278019
N	-6.4124283203	-0.9744059684	-0.2650868108
C	-7.3502076247	-0.7207721205	-1.3644115875
N	-4.1839297739	0.4101596897	-1.3454263276
C	-4.661870306	0.7859970531	-2.6818439137
C	-6.9387127321	-0.6000961697	1.0516623895
C	-3.7257829633	1.5616746513	-0.5587901303
C	-5.2722465199	-3.4348852089	-1.6066094736
C	-4.8790240037	-0.4357731366	4.2605930514
C	-1.9422929947	-3.0647874367	5.6450913655
H	3.1364639304	-0.1050377458	1.1667586101
H	2.7268476616	-1.1942685994	2.5149219186
H	-0.2474829278	-2.0334366357	-1.9846807193
H	-1.5710104708	-0.096307418	1.6563503428
H	0.6927876101	-3.9182828499	-0.6031535923
H	0.7978129694	0.6191929495	2.2285059004
H	-0.0710655043	-1.6565837927	2.9515831178
H	0.7882605804	1.0151574914	-0.2920697128
H	2.0447573373	-0.9350307454	-1.483532722
H	2.8738863131	-2.604067753	0.2579581618
H	1.0890924781	-3.4468548997	1.8603913119
H	-1.4513528363	-0.1583476557	-0.8490432589
H	-3.3535807911	1.2311879919	0.4105647836
H	-2.9117037889	2.0608042477	-1.0976893251
H	-3.8498001399	1.2873967924	-3.2216887899
H	-4.9545183458	-0.097536901	-3.2509419924
H	-5.522686547	-3.8021764716	-2.608812762
H	-4.6440360325	-4.1907139093	-1.1119225517
H	-6.1934311258	-3.3135667378	-1.0363773201
H	-6.9478247098	-1.0941786653	-2.3080030715
H	-8.2880233857	-1.251040315	-1.1622089587
H	-5.3170069358	-4.3656133481	1.2416154395
H	-6.6234161751	-4.9254219429	2.3112159465

H -7.8717698003 -1.1498218959 1.2272747102
H -7.1588730726 0.4766513991 1.1107856964
H -6.2200486605 -0.8623698159 1.8271785522
H -4.9958985487 -6.4160329012 3.4180287792
H -3.5666534565 -5.7215250977 4.2128696491
H -1.4226836625 -3.9106136762 6.111636301
H -1.3879458881 -2.7505302692 4.7617634356
H -1.9728029122 -2.2345519771 6.3654898355
H -3.5662873621 -4.780373073 6.8928604546
H -4.6121904486 -0.2161479619 5.3055259323
H -5.7279997456 0.1993848267 3.9840057431
H -5.0527480925 -4.3861527836 6.0168390159
H -6.750475304 -3.2336298695 4.6637145889
H -7.3154673199 -1.5700445667 4.4205334698
H -6.3984882488 -1.9870489509 5.8860687417
H -3.6374265533 -5.8112198964 2.4367546765
H -4.3182853745 -3.1822901249 7.1080948677
H -4.5339813379 2.2912128457 -0.4008644493
H -6.2588684813 -3.1902881303 2.1814855351
H -4.0396623329 -0.1846771462 3.6136450905
H -5.5167110273 1.4753039114 -2.6308978676
H -7.5774153047 0.3497929852 -1.4726306905
H -3.5209656428 -2.6288073463 -3.4850683745
H -2.8075556621 -1.3044027748 -2.5400898911
H -2.6223435787 -2.9833302724 -1.9898442328
H -2.739471604 -3.1640343517 0.607040189

9b
C -0.12223274 0.0380603923 -0.0588850951
N -0.0327567491 -0.0151432101 1.4064715906
C 1.3605868845 -0.1394865701 1.8625872089
C 2.1752438702 -0.2154679566 0.5553517635
C 1.2787643656 0.5036708095 -0.4673212758
P -1.3631708349 0.0372976069 2.4339396426
N -0.8924835751 0.7676440693 3.8754449707
C -1.2072686542 2.1731164506 4.1865518569
C -0.8905815292 2.3173200758 5.7013681548
C -0.5268915798 0.8947474163 6.1807526813
C -0.0035414022 0.2235142454 4.9052596579
N -2.6040579216 0.6037007121 1.7104655885
P -3.6827749484 1.8035999649 1.210187311
N -4.5623859325 2.26960008 2.6123709625
P -5.7526736157 1.4468131438 3.2211270988
N -6.9107897025 2.5520676157 3.7259155522
C -7.08049921 3.8802068006 3.1017030772
C -8.5811346069 4.1595999697 3.2601005058
C -8.9184660157 3.469817825 4.591554777
C -8.0785466129 2.1811427737 4.5395339936
C -2.6246948617 3.3730411326 1.1710037335
C -3.26956408 4.631668904 0.5493193722
C -3.325821201 4.600021958 -1.0390978896

C	-2.7330949522	3.304525443	-1.6222427374
C	-1.3003195016	3.3830624775	-1.0859946468
C	-1.2492824276	3.4052007492	0.4869323417
C	-0.9508798839	4.9085603965	-1.1209255891
C	-2.2679647398	5.6756796073	-1.4376197331
C	-0.8963124237	4.9354393078	0.4424325288
C	-2.186380677	5.721519801	0.8216956177
C	-2.3502621019	6.7288783179	-0.3242551627
H	-3.2686546165	2.4005325653	-1.3191481188
N	-1.5430686525	-1.5677658761	2.9676292357
C	-1.5712134989	-2.6432813665	1.9447978661
C	-2.8531994889	-3.4378158792	2.2388391672
C	-3.0204426441	-3.2682685999	3.7557600288
C	-2.6032884055	-1.8083953083	3.9773631323
N	-5.3209755808	0.5036758433	4.5454559007
C	-4.3575688568	1.0514018028	5.5238580225
C	-4.7546085362	0.3882131301	6.8495318698
C	-5.3100701203	-0.9713510343	6.3981491164
C	-6.0819533886	-0.6253070031	5.1143638312
N	-6.6049641584	0.3106899887	2.289031153
C	-5.9178700337	-0.9109866866	1.787694735
C	-6.2378216321	-0.9541061841	0.2879145858
C	-7.6192937373	-0.2856929665	0.2198545863
C	-7.4836608799	0.8637787828	1.2272137393
H	-1.5423554976	7.4715380398	-0.3697612923
H	-3.3118581831	7.2583208189	-0.2991766359
H	-0.5107472788	2.7369865727	0.9372089671
H	-2.7357085716	3.352159952	-2.7211067262
H	-2.4688698119	3.5979943482	2.2331356786
H	-2.3585961426	6.0331130332	-2.4686629117
H	-4.3312675349	4.8259917361	-1.4098424217
H	-0.0691941446	5.2363013225	-1.6793729815
H	0.0236048766	5.2767163349	0.9268752155
H	-2.2057692567	6.1194308337	1.8410731681
H	-4.2425724467	4.8724338909	0.988117111
H	-0.5909462481	2.7014230252	-1.5646420255
H	-0.3330962127	-0.9562016323	-0.48966245
H	-0.9236711707	0.7094804545	-0.3692226628
H	1.522341352	0.2552617748	-1.5058658072
H	1.3564017998	1.5919222749	-0.3471828005
H	2.3088710762	-1.2633347574	0.2573622328
H	3.1694087035	0.2321834512	0.6588829636
H	1.5082588362	-1.0261789806	2.494273009
H	1.6473493937	0.7411577742	2.4546481175
H	1.0472576715	0.5108285223	4.7290335592
H	-0.0528025131	-0.8675766918	4.9191126671
H	-1.4205345801	0.363906408	6.5296671179
H	-0.0419420873	2.9951514692	5.8484092137
H	0.2070877739	0.8936537902	6.9943541967
H	-1.7396927888	2.7353833543	6.252152489
H	-2.2558026052	2.3738262822	3.9544455052

H -3.455116068 -1.1347366285 3.8204642318
H -4.0400776771 -3.4638499366 4.1059711182
H -2.3428672398 -3.9464854194 4.2910243176
H -2.7836060363 -4.4839720677 1.9210182136
H -0.6793976301 -3.2778136737 2.0511944023
H -1.5716228653 -2.2358978044 0.9298698162
H -3.7007506699 -2.9781199376 1.7164150457
H -6.1157222808 -1.4681719481 4.417997242
H -7.1203463709 -0.3490244422 5.3445408128
H -4.4840249655 -1.6543277256 6.165425435
H -3.3379221717 0.7784070911 5.2333180058
H -4.4109148608 2.1445889127 5.5632997877
H -5.9466481498 -1.4552367738 7.1466155759
H -3.9110562588 0.303951059 7.5436942905
H -5.5419513991 0.9714585243 7.3446022028
H -6.476620199 4.6309538305 3.631267996
H -6.7478122245 3.8733971611 2.0607992174
H -9.1385327611 3.6876182836 2.4402122437
H -8.8138008501 5.2299572787 3.2558461228
H -8.6426096997 1.3569509411 4.0741500423
H -7.7743127035 1.8500462819 5.5399935868
H -8.5895382883 4.0969145585 5.4302072918
H -9.986519205 3.265904492 4.7227444017
H -8.4450025778 1.1775749748 1.65038718
H -7.0217249544 1.7351185024 0.7409373615
H -8.3996675516 -0.9861201882 0.5464735687
H -7.881745823 0.0727285742 -0.7811117252
H -6.227992209 -1.9740962021 -0.1126955222
H -4.8423642112 -0.871171022 1.9785611105
H -6.32745238 -1.7968111525 2.2960341881
H -2.2198882852 -1.6310062493 4.9890772705
H -0.5926931101 2.8658182197 3.5933253724
H -5.5008507142 -0.3541700843 -0.2577485505

9bH$^+$
C 0.1409245349 0.1269566525 0.2072313192
N 0.047701786 0.0050384014 1.6742575269
C 1.3852042944 -0.1622809928 2.2869900713
C 2.3553079848 -0.0904029409 1.0888017053
C 1.5598031143 0.6650006548 0.0097557784
P -1.3608196366 -0.0683651376 2.5502246982
N -1.1378878459 0.655763771 4.0330347125
C -1.072444626 2.1343693711 4.1424837032
C -0.2115637149 2.379798086 5.3903984879
C -0.4878954261 1.1411461154 6.2547583484
C -0.5410529754 0.0045445009 5.2260233717
N -2.5259024879 0.556016868 1.6742404578
P -3.4825854993 1.8362383973 1.5223523809
N -4.4588383444 2.2308288913 2.7530664822
P -5.7264291496 1.4272160902 3.3008340705
N -6.7813858942 2.5921241684 3.8262438532

C	-6.8342514907	3.9744017581	3.2883819862
C	-8.2595566514	4.4314850775	3.6278393724
C	-8.5726387438	3.6755491103	4.9283544968
C	-7.9441775563	2.294508495	4.6873774192
C	-2.6197262571	3.4156781201	1.1621676095
C	-3.4180453504	4.484194479	0.3773502806
C	-3.4593358323	4.2165951477	-1.1892129383
C	-2.704261274	2.9392228872	-1.6065522944
C	-1.2967322432	3.2583042464	-1.0989291319
C	-1.2551417467	3.4958311371	0.4534605442
C	-1.1364428571	4.7948876274	-1.3415235963
C	-2.5352223287	5.3469945651	-1.7428796721
C	-1.0923737025	5.0392432371	0.2026825173
C	-2.466954031	5.715006263	0.4875957507
C	-2.7462949217	6.5253087817	-0.7839962528
H	-3.1130690793	2.0030961975	-1.2157376627
N	-1.6160256403	-1.6702673188	2.9689746375
C	-1.3450047581	-2.7521941005	1.9931983807
C	-2.7362752036	-3.2890469622	1.6415313901
C	-3.4507116406	-3.2518474272	3.0030311845
C	-2.8611296382	-2.0070737959	3.7122866329
N	-5.3159950903	0.3961500725	4.5400520477
C	-4.3915869998	0.8867278339	5.5963564871
C	-4.8908257512	0.1976096739	6.8722480001
C	-5.4535571567	-1.1324998153	6.3494978028
C	-6.139060347	-0.7362814925	5.0328798541
N	-6.5682381985	0.4404177908	2.2400561217
C	-5.9941653109	-0.8076900753	1.6668130541
C	-6.4230195834	-0.7820890171	0.1940460932
C	-7.7651340788	-0.0365605027	0.2446842514
C	-7.5023260285	1.0732700102	1.2702348658
H	-2.0311422545	7.3419699709	-0.9395763307
H	-3.7617618542	6.9385782459	-0.8188186253
H	-0.4456686241	2.9845553777	0.9801137785
H	-2.7049688726	2.8531772162	-2.7009019182
H	-2.4850584113	3.8054218808	2.1811340883
H	-2.6655056869	5.5448201214	-2.8102726485
H	-4.4819221564	4.2679522345	-1.5753479646
H	-0.2989524946	5.1467978553	-1.9476942968
H	-0.2221232136	5.5443849868	0.6282097105
H	-2.537878009	6.2445753117	1.442100378
H	-4.4129154783	4.6699212974	0.7923413342
H	-0.5068440613	2.6103132719	-1.4867645478
H	0.0299844992	-0.8524028091	-0.2852251897
H	-0.6451535571	0.780999269	-0.1674049607
H	1.9389789546	0.4909379088	-1.0017888687
H	1.5775804315	1.7455505607	0.2010628866
H	2.5887588456	-1.101943497	0.735921703
H	3.2994389169	0.3949496726	1.3526908621
H	1.4696837189	-1.1140353853	2.8266993944
H	1.5672181011	0.6482213909	3.0042672598

H 0.465217497 -0.3793735065 5.0135356746
H -1.1546353889 -0.8355663795 5.5587892854
H -1.4576812032 1.2399398941 6.7581320484
H 0.8497953707 2.4160329796 5.1154247745
H 0.2733922593 0.967513345 7.0210454977
H -0.4618471588 3.3219266928 5.8872109532
H -2.0841117979 2.5412913774 4.2613206904
H -3.5543574049 -1.1617922156 3.691302231
H -4.5397100632 -3.1920401894 2.9132426376
H -3.2179751546 -4.158480086 3.5731219205
H -2.709771017 -4.2898725151 1.1989697463
H -0.738520525 -3.5287426125 2.4793392325
H -0.7844941589 -2.3808078236 1.1340053593
H -3.2201073553 -2.6075117967 0.930994989
H -6.1434897508 -1.5557753666 4.3096385385
H -7.1803012199 -0.4359344615 5.2028395583
H -4.6354274961 -1.8347174353 6.149562413
H -3.3663168751 0.5925331823 5.3476904137
H -4.4191441762 1.9789084562 5.6663545709
H -6.1472424235 -1.6154716597 7.0439766868
H -4.0966334368 0.0688270013 7.6144479261
H -5.6895875013 0.7907351519 7.3346363891
H -6.0776582924 4.5968116023 3.7820232336
H -6.6277275937 3.9925112643 2.2144097497
H -8.9527453751 4.1204122999 2.8359121845
H -8.3331417774 5.5182219788 3.7315993543
H -8.6505113568 1.6181517704 4.1820604953
H -7.6257915076 1.8148626929 5.6189399951
H -8.0808175928 4.1659087052 5.7774035513
H -9.6422499314 3.6115012761 5.1486361904
H -8.4107195997 1.4130571748 1.7768773002
H -7.0407571635 1.9444028976 0.7817494835
H -8.5553200991 -0.7050892965 0.6077419657
H -8.0790480863 0.3630411282 -0.7241262798
H -6.5001101381 -1.7866768039 -0.2330497409
H -4.908618665 -0.8482252014 1.7909348929
H -6.4278789113 -1.6778613234 2.1762084696
H -2.6277989802 -2.2210931677 4.7617878337
H -0.6281488448 2.5793944931 3.2455421126
H -5.6961412365 -0.2169768838 -0.4029663378
H -4.2774717127 1.525559936 0.4038644251

10a
C 0.997073496 -0.0829887572 -0.3463644372
C 0.1967451058 0.1591650647 0.9817732687
C 1.0799241751 0.138890175 2.2420831778
C 1.649280277 -1.3030825003 2.1421245732
C 2.4612191405 -1.5463065285 0.7953612332
C 2.4978846208 -0.2691508381 -0.0735447124
C 0.3044223848 -2.0828310052 2.052921572
C -0.3722603424 -1.3078428995 0.8988336218

C	1.6310790334	-2.4694077897	-0.1417282143
C	0.4269752571	-1.5446015265	-0.4259603685
C	1.2383152185	-3.8155919168	0.4714593123
C	0.4473637301	-3.5835580891	1.7840043836
P	2.2321466904	1.5773813074	2.6437897561
N	2.4981732238	1.2092675146	4.3049460929
P	3.7734120604	0.5965611957	4.9602953674
N	5.2420185065	0.413530113	4.1175077922
C	5.2965811905	-0.5769779514	3.0348812482
H	2.9628524057	0.5935923765	0.408510538
N	1.0069706532	2.7584433136	2.6771351256
P	1.0605637915	4.2202927448	3.2156601549
N	-0.0799338651	5.1304549078	2.35043363
C	-0.3500049262	6.5219557261	2.6902354448
N	2.4540510027	5.1955388114	3.0665745043
C	3.6149345731	4.8764822694	3.9068507159
N	0.8087418652	4.294526457	4.9004092427
C	0.7854219945	5.5586118803	5.6313585723
C	2.8592798186	5.53601157	1.6970694873
N	4.2613698675	1.573805662	6.2660204901
C	5.313576512	1.1319409535	7.1747015384
N	3.4894660553	-1.0127780669	5.4431724049
C	4.5094459338	-1.8560832076	6.0578552766
C	3.2837945261	2.4614657299	6.8900569641
C	2.1260355879	-1.4187646733	5.7774929298
C	5.9848823322	1.6303952534	3.7730544281
C	-0.0518117387	3.270589138	5.4973499668
C	-1.1889783145	4.4596591552	1.6765734783
H	-0.51822054	0.9845907386	0.9612644493
H	3.0448640613	-0.4675455231	-1.0060273491
H	0.4069419259	0.1693469519	3.1124737075
H	2.2006502542	-2.6408260437	-1.0656957442
H	3.4551222653	-1.9562696792	1.0087317336
H	-0.1599571501	-1.8254142051	-1.306645567
H	-1.4583465683	-1.4369927903	0.8431658095
H	-0.2525907768	-1.9354568929	2.987603398
H	2.2439957961	-1.5923343844	3.0131075606
H	0.7616822664	0.5998061483	-1.1683481751
H	-0.5506063326	-4.0387910486	1.7270178552
H	0.9544249778	-4.0564946877	2.6361806708
H	0.6402586063	-4.3852009379	-0.2525019694
H	2.1442082463	-4.4080670047	0.6582951238
H	1.4148505944	-0.7717355644	5.2631849952
H	1.9621648259	-2.4551849147	5.4514354948
H	4.3566270085	-2.8979065936	5.7425453878
H	5.5060550527	-1.5474617571	5.7377910192
H	4.712975933	-1.4608903211	3.2953642807
H	4.9052442583	-0.1687955711	2.0919716736
H	6.3411529388	-0.8786391646	2.8807216275
H	7.0378218402	1.3681807642	3.6052633475
H	5.5905803594	2.0993346395	2.8584840838

H 5.929034675 2.3509606499 4.5908072613
H 6.071507298 0.5626610189 6.6323603931
H 5.8013343216 2.0113625484 7.6161394303
H 4.2042450568 4.0459178936 3.4924000038
H 4.2589693628 5.7635131873 3.9720394721
H 3.8020455926 3.3452283027 7.2882313277
H 2.7546490011 1.9719975829 7.7257639672
H 2.5538838642 2.7797776571 6.14678492
H 3.5300214629 6.4040855103 1.7319468341
H 1.9845261978 5.7929784623 1.0969952231
H -1.4318706058 5.0018902807 0.7520954997
H -0.898558411 3.4387455345 1.4283711767
H -2.0943108109 4.4315881691 2.3060416592
H -0.6507979726 7.063585147 1.7829713698
H -1.1210262898 3.5325373255 5.4222872801
H 0.1996382129 3.172583305 6.5614610262
H 0.5485642666 6.999998238 3.085737366
H 1.4503119505 6.2858531566 5.1620194691
H 1.1355095982 5.3875910388 6.6590270365
H -0.2268190571 5.9914093029 5.6884728342
H 3.3884424064 4.7032543014 1.2077636795
H -1.1625385158 6.6253005512 3.4286549852
H 1.9387157387 -1.3666125999 6.8632456413
H 3.2979380931 4.5985955803 4.9112812368
H 0.1255100137 2.3120408013 5.0109908614
H 4.4666157719 -1.8284149836 7.1588462989
H 4.9259845808 0.5115232028 8.0000791508

10aH+
C 0.8479069575 -0.0464542323 -0.2653924194
C 0.086074032 0.0759535935 1.0977062058
C 1.0115707148 0.0376229597 2.3311493873
C 1.6869460489 -1.3497782971 2.1380555744
C 2.4587153063 -1.4564708679 0.7514529964
C 2.3660483085 -0.1445432519 -0.0621315857
C 0.3891169518 -2.2082856725 2.0554822511
C -0.3798424012 -1.4235317144 0.9678510327
C 1.6553768739 -2.3903137344 -0.2006838458
C 0.3800004154 -1.5400862006 -0.3949304869
C 1.3819330107 -3.7885529402 0.3568132923
C 0.6219017151 -3.6787773564 1.7021821732
P 1.9764698731 1.48370242 2.8871585857
N 2.4728975406 1.2266583684 4.4034944274
P 3.7735478653 0.5844105789 5.0404467528
N 5.1843117971 0.4617556572 4.137979126
C 5.3066877006 -0.5477474402 3.0776564436
H 2.8065969728 0.7368341272 0.4069189333
N 1.0041675342 2.7484271187 2.7037966899
P 1.0715702798 4.2541154044 3.208477975
N -0.0534913647 5.0939749033 2.3044380704
C -0.3289612517 6.5027645601 2.6003110695

N	2.4913904746	5.1333419765	3.010319319
C	3.6638271267	4.7997744002	3.8298133647
N	0.8384151123	4.3308515736	4.8781679922
C	0.9283525041	5.6070811981	5.6005438709
C	2.8633852946	5.5937486047	1.6650698458
N	4.2235941988	1.5692697412	6.3245754899
C	5.3168701263	1.156248616	7.2111896173
N	3.4652623999	-1.0199205764	5.4455059401
C	4.4985906562	-1.877932795	6.0381847868
C	3.2431993641	2.4477249689	6.9713498128
C	2.1045702006	-1.4331587194	5.8084177204
C	6.0090267577	1.6483230446	3.8829732304
C	-0.1431414114	3.4166230534	5.4789088863
C	-1.1105893654	4.4237017749	1.540525228
H	-0.6841502648	0.8477492993	1.1506128625
H	2.8787579913	-0.2717767122	-1.0233923328
H	0.3569331333	-0.0422138788	3.2145380131
H	2.1987989699	-2.4753247834	-1.1502196621
H	3.486193384	-1.8023625814	0.9061211853
H	-0.2211438841	-1.8179671126	-1.264427286
H	-1.455312043	-1.6188266192	0.9469914338
H	-0.1394656323	-2.1481709668	3.016213073
H	2.3264278568	-1.640226275	2.9763490671
H	0.5326796261	0.6566222471	-1.0404321381
H	-0.3467055475	-4.1910077577	1.6511131342
H	1.1830591241	-4.1616937967	2.5127605218
H	0.802444159	-4.3634733127	-0.3755750224
H	2.3326904883	-4.3212322452	0.4846670711
H	1.37309495	-0.7808344291	5.332536547
H	1.938032993	-2.4600499373	5.4616876037
H	4.3369479586	-2.9104806899	5.7061736944
H	5.4928121995	-1.5674931133	5.7138810878
H	4.6866613752	-1.4163461784	3.2975702805
H	5.0058549752	-0.1413539045	2.1014863396
H	6.3518876663	-0.8710870008	3.0089726263
H	7.0634112115	1.3509723049	3.8397724614
H	5.7458244328	2.1178154329	2.9233023381
H	5.8827490727	2.3807215251	4.6805999853
H	6.065665861	0.5849206835	6.6591335972
H	5.8042342306	2.0505981545	7.6162688243
H	4.2908245418	4.0411183463	3.3394676739
H	4.2687558776	5.7023791494	3.9752373972
H	3.7644939778	3.3320811409	7.3582455696
H	2.7429554869	1.948055528	7.8148240126
H	2.4936490987	2.7649230482	6.2471717158
H	3.5021876177	6.4789917436	1.7595147151
H	1.9753452777	5.861930702	1.0923128968
H	-1.2608894971	4.9582782964	0.5946379381
H	-0.8187899746	3.3964429845	1.3257130438
H	-2.0610446855	4.4207142432	2.0935623917
H	-0.5409588279	7.0322678044	1.6638422768

H -1.1684132871 3.8099496586 5.404687781
H 0.0986725188 3.2910246034 6.5402470769
H 0.5369809849 6.9767546016 3.0666065497
H 1.6609748735 6.2658010749 5.1328239751
H 1.2506403716 5.4077940723 6.6296446109
H -0.040734839 6.1253215633 5.6397843003
H 3.4188704863 4.821393931 1.1118031994
H -1.198188523 6.616593794 3.2639839421
H 1.9512605168 -1.405810285 6.8973556776
H 3.3610343117 4.4176483169 4.803956514
H -0.09606659 2.4395999803 5.0001674989
H 4.4618056149 -1.8612213088 7.1367162859
H 4.956194648 0.5497654236 8.0547744798
H 3.1284697993 1.6442876315 2.0846053222

10b

C 0.407663994 0.4643490369 0.5282675723
N 0.3356435178 0.232388297 1.9885716231
C 1.6801610259 0.1766710397 2.5860080032
C 2.5904033744 -0.1197353988 1.3874071525
C 1.9094860812 0.6737072583 0.2607013301
P -1.0922686117 -0.2637820597 2.7309021715
N -1.51363197 0.7856888565 3.993711992
C -2.3057796192 1.9944523725 3.6886860485
C -2.4016072527 2.7399531492 5.0466374498
C -1.4246710638 1.9983942646 5.9963891034
C -0.5609149583 1.1537143941 5.0498405723
N -2.1607318889 -0.4849625302 1.637071014
P -3.7898348143 -0.5177779574 1.1930632166
N -4.719266246 -0.0428622694 2.5625443645
P -5.6886545196 -0.9905148671 3.3546448104
N -7.2056324247 -0.2701705929 3.4320329687
C -7.7217729078 0.6061222321 2.3598168865
C -9.2279762105 0.3151580983 2.3464449058
C -9.5304168373 0.013654957 3.8225858018
C -8.296688116 -0.786645913 4.2766784952
C -3.820766228 1.1492414972 0.296765783
C -3.1816362465 1.2334816389 -1.1156646756
C -4.095137415 0.5579507942 -2.2316099773
C -5.3916600322 -0.0247881556 -1.622364484
C -6.0325230246 1.2566304314 -1.0689693566
C -5.1325413611 1.9127330066 0.036290099
C -5.6103366849 2.3784237654 -2.0838639566
C -4.7132930465 1.6719632259 -3.1245422498
C -3.2253759425 2.7771177996 -1.3097193033
C -4.7137648544 3.0385947714 -0.9840527357
C -2.8081856459 3.2670087378 -2.6993798702
C -3.6937198697 2.603349584 -3.7846716496
H -5.2217844436 -0.7948272388 -0.866395954
N -0.5963243649 -1.6105298654 3.6530329934
C 0.0219918823 -2.7267181162 2.8965693204

C	-0.9317286934	-3.9110811471	3.0897948517
C	-1.4386320647	-3.6880183649	4.5222584829
C	-1.6027122974	-2.1598028801	4.6008467338
N	-5.1598575558	-1.28760869	4.9222193529
C	-4.4572829104	-0.2296766731	5.6764700983
C	-4.1325843331	-0.8813168028	7.0473395215
C	-4.5374921573	-2.3622727358	6.8933454374
C	-5.6631496476	-2.300420003	5.855126278
N	-5.9911402545	-2.5696874599	2.8124792815
C	-4.8906895721	-3.5654319076	2.7666183412
C	-5.0204082064	-4.2304820186	1.3897774873
C	-6.5273017874	-4.1328838999	1.1064325104
C	-6.8784858494	-2.7363454274	1.6360253132
H	-7.0972093092	1.1727800056	-0.8269088348
H	-3.2128379364	1.7744841065	0.9643977028
H	-6.0119145004	-0.4640505107	-2.416538578
H	-2.5827272338	3.246783514	-0.5530383552
H	-2.163937876	0.8322102124	-1.1476735196
H	-4.9451361009	4.0777121863	-0.726429506
H	-6.3953192616	3.0075018547	-2.516574632
H	-5.334504911	1.202276179	-3.8999351784
H	-3.5230255867	-0.1757894312	-2.810539207
H	-5.6459163119	2.22093637	0.9490210899
H	-4.2229320729	3.3613116174	-4.3778736354
H	-3.0820778564	2.0279648436	-4.492546853
H	-2.8929688453	4.3615551894	-2.7390206633
H	-1.7474507235	3.0328490466	-2.8620606025
H	0.0138579153	-0.4031994306	-0.0156691084
H	-0.1932550142	1.3307049537	0.2324468983
H	2.1987697228	0.3430373271	-0.7424044404
H	2.1669508809	1.7372273306	0.3498968523
H	2.5702467256	-1.1941094353	1.161862387
H	3.6315150826	0.1740293543	1.5609782826
H	1.7303072094	-0.5830691381	3.3709365831
H	1.9522505042	1.1432307078	3.0390486248
H	0.2704363141	1.7606944883	4.6494532159
H	-0.1344145964	0.2613759926	5.5155366301
H	-1.9769781155	1.3377840405	6.6736064942
H	-2.1215217541	3.7921546104	4.9279400588
H	-0.8245478267	2.679097048	6.6102121179
H	-3.4249200003	2.7190027129	5.4352614707
H	-3.2788797191	1.699553034	3.2925560677
H	-2.617141568	-1.8701647929	4.3068819331
H	-2.3771093094	-4.2107425159	4.7372140708
H	-0.6886584522	-4.0337463506	5.2456067614
H	-0.4426199781	-4.8811789638	2.9471456541
H	1.0139208857	-2.953010433	3.3167422344
H	0.1571524027	-2.4605837035	1.8439104477
H	-1.7593495782	-3.8324668029	2.3747574936
H	-5.8464884079	-3.2466175841	5.3429195909
H	-6.6056284291	-1.9914723789	6.3388185036

H -3.7040675196 -2.9488261086 6.4902948541
H -3.5633712907 0.0705158004 5.1298488712
H -5.0907069304 0.6614174017 5.8036492449
H -4.8541850208 -2.8201301966 7.8369329227
H -3.0763600306 -0.7651378772 7.3118382296
H -4.7212014183 -0.4096061786 7.8427835294
H -7.5224986238 1.6601109805 2.6004583744
H -7.2359324426 0.3943685189 1.4058622002
H -9.4329982643 -0.5701279103 1.7303995991
H -9.8140913602 1.1489972302 1.9454434077
H -8.4441169226 -1.8669650943 4.1214377007
H -8.0795085897 -0.6321594899 5.3397715441
H -9.5966104182 0.9515539335 4.3887287033
H -10.4638474327 -0.5382639063 3.976190156
H -7.92982497 -2.6456341996 1.9317885949
H -6.6730268087 -1.9798366906 0.8654692019
H -7.0739265073 -4.9003034221 1.6706551125
H -6.7785830782 -4.2467494668 0.0467544385
H -4.4621990216 -3.6433760598 0.652111334
H -4.6422873278 -5.2588377346 1.3836233507
H -3.9168371905 -3.0901111553 2.904672503
H -5.0213674491 -4.302387933 3.5739916857
H -1.4328442811 -1.7717100307 5.612070587
H -1.7951421796 2.6245907546 2.9413463399

10bH^+
C 0.0122635058 -0.3003578894 0.1002719405
N 0.0180749781 -0.081767218 1.5681745398
C 1.3999318862 0.0519595255 2.0854282141
C 2.2689639601 -0.460679453 0.9299208423
C 1.4673342016 -0.0280409914 -0.3073235357
P -1.298760114 -0.3567552122 2.5468427317
N -1.5398251369 0.8404919455 3.6851405956
C -2.3504780277 2.047401113 3.36317364
C -1.9078480364 3.0998144012 4.400569866
C -1.2699872764 2.2721512651 5.5280559121
C -0.5648601213 1.1563732411 4.754660478
N -2.5359922433 -0.5668562562 1.5864026187
P -4.0741459918 -0.2191486408 1.302061665
N -5.0943804334 -0.0393804464 2.5450676243
P -5.8414593979 -1.1264647228 3.4404829905
N -7.3997511977 -0.5885741589 3.6089410043
C -8.104779435 0.2304501543 2.5924071045
C -9.5863145123 0.0296265415 2.9410922966
C -9.5662825431 -0.1821627541 4.4627729964
C -8.3129140936 -1.0440366836 4.6767283617
C -4.2881053274 1.335436395 0.3539886682
C -3.1858584126 1.9782061703 -0.5373892369
C -2.9691820065 1.2206173238 -1.9152257068
C -3.883998262 -0.0155817381 -2.0119860116
C -5.2686609007 0.655172469 -1.9472384441

C	-5.499452958	1.4248247446	-0.5973926771
C	-5.0622543252	2.0276945274	-2.6860559241
C	-3.5680174359	2.072202865	-3.0707348564
C	-3.9133092692	3.3268767839	-0.8271098952
C	-5.2724092159	2.7927580894	-1.3369306935
C	-3.1953097993	4.2327367733	-1.8304929535
C	-3.0054945128	3.4909810696	-3.1771522602
H	-3.6968698866	-0.7739417695	-1.24951554
N	-0.8654425817	-1.6627775459	3.4999376295
C	-0.2305373009	-2.8478847775	2.8622983489
C	-0.9450501512	-4.0508252159	3.4930764764
C	-1.3082819868	-3.5401855643	4.8950630016
C	-1.7168354905	-2.0827292422	4.6401584014
N	-5.1099025689	-1.2813130159	4.9238149394
C	-4.5984840957	-0.0691591337	5.6162097677
C	-4.6546370878	-0.4432714587	7.1046562496
C	-4.4779388085	-1.9697664331	7.0955746587
C	-5.2884532415	-2.4087624314	5.8682570969
N	-5.9174895203	-2.7017421627	2.8757387201
C	-4.6944116284	-3.5161140081	2.6486542534
C	-5.0116520433	-4.3347415695	1.3887464353
C	-6.5388019538	-4.4890332033	1.4529067941
C	-6.999028217	-3.1113811077	1.9430151936
H	-6.099522413	0.0237407753	-2.2733777284
H	-4.4729956701	2.028108064	1.1864501541
H	-3.7449550666	-0.5099039244	-2.981385659
H	-4.0496988343	3.8717500122	0.1164542058
H	-2.2388853385	2.1163332817	-0.0064243871
H	-6.0830422428	3.5260730619	-1.3094831687
H	-5.7482485035	2.2804419081	-3.4988115365
H	-3.4062614486	1.5418051704	-4.0178343682
H	-1.9109472097	0.9918083639	-2.0746895134
H	-6.4659347939	1.2665164488	-0.1118094626
H	-3.5120826319	4.0214787527	-3.9927592528
H	-1.9445646942	3.442106386	-3.4526455275
H	-3.7801998577	5.1509220016	-1.9653764202
H	-2.2293091103	4.5415752765	-1.4106969149
H	-0.281016829	-1.3313380206	-0.1404929852
H	-0.6994009932	0.3662497666	-0.3877138339
H	1.7468349284	-0.5683866399	-1.2167832479
H	1.6133295844	1.0435119236	-0.4929215505
H	2.3407347767	-1.5549413918	0.9713396635
H	3.2838013476	-0.0526793572	0.9569258118
H	1.5338168594	-0.5178214409	3.0105451837
H	1.6246292992	1.1058398688	2.3003360058
H	0.3854805585	1.5236418648	4.3406810186
H	-0.3487071135	0.2715699947	5.3572380283
H	-2.042017152	1.8449289226	6.1799371554
H	-1.1573535169	3.7690818568	3.963677212
H	-0.5802793714	2.8505351486	6.1500908639
H	-2.7453474475	3.7173700512	4.7386704973

H -3.4123258519 1.801119422 3.4574842527
H -2.780178993 -2.0091023933 4.3879718462
H -2.1091993149 -4.1143187937 5.3721299869
H -0.4303762513 -3.5798093737 5.5513615998
H -0.3181691502 -4.9475718412 3.5070166571
H 0.8429814237 -2.8595530138 3.0930258057
H -0.3385542536 -2.8251552646 1.7737930807
H -1.8551140804 -4.2838111625 2.9271489405
H -4.9194889682 -3.3415199601 5.4342843927
H -6.344265407 -2.5577122275 6.1295325258
H -3.4214017328 -2.229132047 6.9599783056
H -3.5754848848 0.1357593428 5.2826829707
H -5.2103543177 0.8083179132 5.3810340577
H -4.8271108085 -2.4524353222 8.0131525257
H -3.8908280237 0.0764392678 7.6916896948
H -5.6337085669 -0.1813421174 7.5242534563
H -7.8012229113 1.2807321151 2.6824878554
H -7.8648955545 -0.0978069916 1.5763580236
H -9.9714942635 -0.868457736 2.441722461
H -10.203790125 0.877907276 2.6308900603
H -8.545549246 -2.1152211699 4.5742656299
H -7.8646438585 -0.8884614571 5.6633119449
H -9.4486354388 0.7799115643 4.9763272214
H -10.4707584595 -0.6624444015 4.847413473
H -7.9627262929 -3.1387497703 2.4598098687
H -7.0867642529 -2.4141141467 1.0963910548
H -6.8120657586 -5.258876457 2.1849548833
H -6.9893074208 -4.758378061 0.4930748221
H -4.7262868192 -3.7707677742 0.491642357
H -4.4785937625 -5.2901861268 1.3685695007
H -3.8070141795 -2.8886699282 2.5246292406
H -4.5193055893 -4.1747965318 3.5097676595
H -1.5402650504 -1.44334585 5.5106436508
H -2.1700439815 2.3952164464 2.3391154433
H -4.5373703037 -1.2848860945 0.5068841467

Cartesian coordinates of neutral and protonated forms molecules **Iaa**-**IXaa**

Iaa

C	0.2289867361	0.1347656286	0.0797839131
C	0.2519611873	0.2377070856	1.498219249
C	1.5108503762	0.1140929422	2.1727683365
C	2.6814642978	-0.1134098492	1.4018706184
C	2.6439432546	-0.1886788672	0.0219203536
C	1.3899595195	-0.0678153734	-0.6374035371
C	1.5434845862	0.2286907003	3.5900137718
C	0.3965586498	0.4366572529	4.3302123405
C	-0.8481230677	0.5591863524	3.6508282783
C	-0.9178476099	0.4632426901	2.2778923238
H	-1.8751576865	0.560854024	1.767135009
H	-0.7266099644	0.2248996385	-0.4354940331
H	-1.7422007421	0.7431811485	4.2413779876
H	1.3698429067	-0.1431993692	-1.7215372162
H	3.6329234916	-0.2133244004	1.9256881371
H	2.5060603938	0.1390490022	4.0951325865
P	4.2657366485	-0.4030182884	-0.8816663001
N	3.6412441431	-0.8205525995	-2.4101526273
P	4.4414560126	-1.0196045528	-3.7367815737
N	3.5294815379	-2.0482491557	-4.7309407786
P	4.7302549614	2.6328839469	-0.4797662404
N	4.820448415	0.4536619597	-4.4975416574
N	5.3366074104	2.7120259476	1.1062608964
N	4.777368037	1.2045742346	-1.0733001525
N	5.585483792	3.6402256283	-1.5599382746
C	3.9240263298	-2.2621092131	-6.1181330472
C	2.0985814641	-2.2168458105	-4.4888319064
C	2.3737245368	3.2548694779	0.8028773882
C	5.4020015987	3.9445492394	1.8852315868
C	6.2527217906	1.6805430214	1.5825333171
C	7.1078839776	-1.0575024465	-3.1846425512
C	5.8310703751	5.0380123171	-1.2227237319
C	6.6287183899	3.0514235128	-2.3947564387
C	5.6122625059	0.5475388455	-5.7199690196
C	3.9035027387	1.5789221524	-4.3188325856
C	2.5664669365	3.8590120885	-1.5383483102
N	3.2932234192	3.5270970492	-0.3098151676
H	6.026747406	1.446858336	2.6325644021
H	6.142101284	0.769470196	0.9927783277
H	7.3032005062	2.0117708771	1.5254188955
H	8.036576353	-1.4733673839	-3.5972455254
H	7.1366389061	-1.1482240596	-2.0887115357
H	7.056757296	0.0016123656	-3.436932653
H	5.9501497963	-3.3529847343	-2.3132557988
H	6.9297492642	-3.6441220626	-3.7764130443

H	5.1543199289	-3.7263167041	-3.8628549726
H	6.4072667768	4.3953573346	1.8540302204
H	5.1600540119	3.7297226712	2.9358432619
H	4.6812170122	4.674407773	1.5145604049
H	1.8915558024	-2.0886161323	-3.4262646472
H	2.9125381865	2.8825248577	1.6730492893
H	1.4947178751	-1.4923368588	-5.0612970549
H	1.7996606191	-3.2286210834	-4.7953448843
H	1.8598409213	4.1863238262	1.0764771832
H	3.5021157331	-1.5037603943	-6.7990734208
H	5.012296204	-2.2516441294	-6.210904739
H	3.5659885235	-3.2465369018	-6.4475928187
H	3.4027683962	1.4961996066	-3.3562703369
H	1.9215083248	3.0282897804	-1.8656906443
H	3.1520961019	1.6246443031	-5.1257141673
H	1.9304259729	4.7339784774	-1.3509285361
H	4.4749563224	2.5156557673	-4.3249948044
H	3.2659158104	4.098611877	-2.3411395066
H	4.9805518002	0.5793504881	-6.6227027333
H	6.2083683681	1.4708586103	-5.6950900026
H	6.2965886596	-0.2984547654	-5.8020513868
H	6.3394510108	2.0381332692	-2.6722377185
H	5.9781826756	5.6117884161	-2.1475901701
H	6.7418545785	3.6561739284	-3.3053384117
H	7.6070127279	3.019646371	-1.8840671395
H	6.7298412307	5.1717633235	-0.5967361905
H	4.9712717614	5.4583712433	-0.696848062
C	5.9957088509	-3.2046632375	-3.4034194163
N	5.9671423038	-1.7815734851	-3.7627047582
H	1.6197636285	2.5084667968	0.527057827
P	0.5410887709	0.4714510328	6.1915932816
N	-0.8457610304	1.3979455565	6.522071721
P	-1.528797339	1.6783070471	7.8962317712
N	-2.4176202961	3.1138728248	7.7353100185
P	0.2454620981	-2.5979831641	6.2223640288
N	-2.4834987026	0.3753778674	8.4341015814
N	1.8754861637	-3.0738812236	6.1051674633
N	0.0605022476	-1.1080320738	6.5996961707
N	-0.6208634635	-3.5067000444	7.3760690475
C	-3.2891708837	3.5704513457	8.8110141386
C	-2.8201661764	3.5769696926	6.4092294968
C	0.3178309803	-3.0296035475	3.5077071774
C	2.2852160036	-4.4348923849	5.769587472
C	2.8801848708	-2.3478698878	6.8784145474
C	0.12743736	0.879227294	9.901967719
C	-0.5852578199	-4.9645002926	7.3508176191
C	-0.8197927718	-2.9501732802	8.7108137216
C	-3.2037037294	0.3930425864	9.7042358032
C	-3.1268106788	-0.4642940447	7.4224631472
C	-1.8325390295	-3.2155294882	4.6163340415
N	-0.376222482	-3.2567448852	4.7832187599

H 3.8251228076 -2.3312473932 6.3182860075
H 2.5630619894 -1.3176670151 7.0474360576
H 3.0709895856 -2.8258927989 7.8544252614
H 0.3741000972 1.1361298831 10.9403674863
H 1.0619515274 0.671029151 9.3594887703
H -0.4764129685 -0.0279574155 9.9005257727
H 1.1110573368 3.1073952667 8.7084446115
H 0.4510575461 3.4859015648 10.323084805
H -0.3933359041 4.0482695991 8.8613210211
H 2.4829801853 -5.0391913565 6.6700748324
H 3.2088651805 -4.4028767835 5.1748354666
H 1.5171481497 -4.9337899044 5.1769867275
H -2.0994027413 3.2309480912 5.6680158503
H 1.3907186409 -2.919301684 3.6628035767
H -3.8232704299 3.2102547124 6.1327272617
H -2.8448372683 4.6754349323 6.4040646352
H 0.1420179809 -3.8903415065 2.8484604934
H -4.3097422682 3.1600848283 8.7301778582
H -2.8739875373 3.2929170297 9.7822481815
H -3.3639344014 4.6656834927 8.774995031
H -2.4884500534 -0.537909902 6.5438386244
H -2.1715408621 -2.2319445497 4.2556038312
H -4.1173996717 -0.0728976427 7.1337820197
H -2.1271086984 -3.9736299738 3.8791608077
H -3.2614174266 -1.4743374646 7.8296266763
H -2.3298395797 -3.4344054105 5.563076824
H -4.2345672683 0.7675934917 9.5940392473
H -3.2594061766 -0.6300900904 10.101918969
H -2.6822212087 1.0150586843 10.4337329467
H -0.9180000566 -1.8671461871 8.6401674537
H -1.5064410506 -5.3566948734 7.8029055644
H -1.7418527611 -3.3673598743 9.139482555
H 0.0124800893 -3.1940815141 9.3940964445
H 0.2684020919 -5.3767171858 7.9152294945
H -0.5327042154 -5.3253958473 6.3216344404
C 0.1839294397 3.2274287104 9.2904825918
N -0.616708193 1.9964206607 9.3038263283
H -0.0480834746 -2.1263446672 3.0063179224

IaaH^+
C -0.0093254619 -0.316342745 0.0241055898
C 0.0950836543 -0.1488975067 1.4319384966
C 1.398654187 -0.104709135 2.030375701
C 2.5345432303 -0.2108985336 1.1929044664
C 2.4037420477 -0.3331217372 -0.180305705
C 1.1137610697 -0.4024135603 -0.7721253238
C 1.5129071032 0.0830358429 3.4358695777
C 0.3998221168 0.2227893141 4.236525542
C -0.8900786269 0.1783490814 3.6330274287
C -1.0417218952 -0.0078118401 2.2772617585
H -2.0341412964 -0.0417025983 1.8313266006

H	-0.9989067365	-0.3660369213	-0.4250036249
H	-1.7585332001	0.3062646744	4.2740385142
H	1.0281735964	-0.5180909458	-1.8483424838
H	3.5187960176	-0.1541430896	1.6535180556
H	2.5078328093	0.1125381897	3.8816959523
P	3.9025217232	-0.2544547405	-1.1800995029
N	3.507938056	-0.7108586878	-2.6713519184
P	4.3710094199	-0.8171053178	-4.0042820716
N	3.429528997	-1.683273291	-5.0733532216
P	4.5598278262	2.564624738	-0.3535130397
N	4.7466775564	0.696428602	-4.6177136805
N	4.7854582943	2.456210275	1.300537304
N	4.6822532942	1.1461585959	-1.0521549056
N	5.7518597406	3.4950446959	-1.0730781658
C	3.8294239092	-1.8160527134	-6.4743844604
C	2.4065041929	-2.6414040042	-4.6448527123
C	1.9166049421	3.0351234234	0.2573990363
C	4.4277813405	3.4984924065	2.2697381459
C	5.7804537608	1.5137487161	1.8189865075
C	6.9391689549	-0.9410953079	-3.1060371177
C	6.0538720054	4.8301828337	-0.5600006536
C	6.282210262	3.2300601435	-2.4085357242
C	5.80736715	0.963792611	-5.5925621821
C	3.6904318713	1.7134775399	-4.6173794078
C	2.823223738	4.1179432611	-1.7308454005
N	3.1317506564	3.4446012777	-0.4658267822
H	5.4070876745	1.0689382785	2.7496993861
H	5.9660272004	0.7157174162	1.0982392244
H	6.7330008523	2.0186395668	2.0353183393
H	7.9152351523	-1.1603150746	-3.5547402862
H	6.9347043767	-1.3326738686	-2.0783132354
H	6.809535461	0.1404970408	-3.0591226809
H	5.7994955807	-3.4399716121	-2.9092002589
H	6.9361398258	-3.3329180939	-4.2765734679
H	5.1963653002	-3.4476789997	-4.5838430851
H	5.3280079391	3.9953952832	2.6588475311
H	3.8938533467	3.0437013978	3.1135372604
H	3.7824018393	4.2493081397	1.8142589543
H	2.1368804267	-2.4571977738	-3.605218335
H	2.1654292849	2.5151931808	1.180755317
H	1.5144427735	-2.5177363517	-5.2706623152
H	2.761516053	-3.6777018989	-4.748971001
H	1.3371032258	3.9316663473	0.5066513157
H	2.947381111	-1.7095921556	-7.1170349436
H	4.5476741944	-1.0440416806	-6.7526856321
H	4.285005128	-2.7971228189	-6.6749467084
H	2.9711830982	1.5099771341	-3.8238017513
H	2.2734183845	3.4588675185	-2.4190402618
H	3.162398433	1.7417862759	-5.581715432
H	2.1994621336	4.9947920205	-1.5233856336
H	4.137613995	2.6970728395	-4.435845152

H	3.7344659404	4.4571758359	-2.2258453845
H	5.3927493116	1.1306723493	-6.5974081041
H	6.3512084722	1.8702126417	-5.2967055881
H	6.5113692154	0.1324994525	-5.6351303173
H	5.9607381842	2.2447886961	-2.7448572021
H	5.5646426635	5.6163541338	-1.1551011239
H	5.9339513868	3.9867329882	-3.1294688046
H	7.3791606955	3.2634780575	-2.384733534
H	7.137263793	4.99667194	-0.5941727689
H	5.7291900027	4.9342740164	0.4757748288
C	5.94835436	-3.0237992319	-3.9170962804
N	5.8821495454	-1.5575901185	-3.92139753
H	1.2923357859	2.3681177998	-0.3503561984
P	0.6215738048	0.3746198918	6.093334228
N	-0.6968393405	1.3928003405	6.3814691693
P	-1.2965303782	1.837212244	7.7594033266
N	-2.0822887148	3.3138426323	7.5094676576
P	0.3429836758	-2.6699075606	6.4445970598
N	-2.3101789487	0.6483194954	8.4305014521
N	2.0005535814	-3.0729929669	6.4766511113
N	0.0621080613	-1.1500157601	6.5759411213
N	-0.5509029004	-3.4528851862	7.6528496234
C	-2.8306112149	3.9582559824	8.5850909554
C	-2.5195465251	3.7012789237	6.1698803242
C	0.5951245563	-3.3679840256	3.8099673115
C	2.4530586985	-4.4621052452	6.3786666387
C	2.8751321987	-2.2587462905	7.3237933
C	0.3698617857	1.0763925001	9.7782224764
C	-0.5164147965	-4.9050685785	7.8044916302
C	-0.8758695238	-2.7406294468	8.8868697028
C	-3.0025088724	0.85304446	9.7035846149
C	-3.0577419898	-0.209791301	7.5071761863
C	-1.6018438673	-3.5948505847	4.8136491802
N	-0.1571973202	-3.5245973039	5.0601795208
H	3.9007695093	-2.3171863507	6.9367554755
H	2.5621448243	-1.2135660692	7.3062814505
H	2.8863835779	-2.6122416242	8.368541798
H	0.6532757299	1.4036545572	10.7863778909
H	1.2781211843	0.7522278959	9.2474634201
H	-0.3006184774	0.221724624	9.8637302246
H	1.493621089	3.1174480628	8.3816275507
H	0.8895346129	3.68280048	9.9625058848
H	0.0626670124	4.1750732576	8.4672015541
H	2.5090498386	-4.9509182782	7.3642935729
H	3.4573989352	-4.4836492786	5.9346771652
H	1.7835605775	-5.0410059674	5.7406268092
H	-1.8864635242	3.2183036252	5.4251776468
H	1.6556839673	-3.2191317353	4.0157174407
H	-3.5700034282	3.4217424877	5.9855245331
H	-2.4335132883	4.791367422	6.0643811901
H	0.4759246891	-4.2771306055	3.2057651521

H -3.8917186151 3.6619700034 8.5924391658
H -2.3903732048 3.7182592758 9.5548005891
H -2.7856158427 5.0473017739 8.4529142058
H -2.4290273301 -0.4740520994 6.6583203497
H -1.97660851 -2.6798766493 4.3286767929
H -3.9854104537 0.2708074416 7.1537165343
H -1.8086831666 -4.4465804405 4.1536109707
H -3.3308300375 -1.1344090293 8.0300831079
H -2.1415824291 -3.7391729207 5.7508698453
H -3.9919181746 1.3181776043 9.5699948889
H -3.1505105742 -0.1194220173 10.1915986126
H -2.4072836527 1.4809170594 10.3685568607
H -0.9548368027 -1.6730312364 8.6832577693
H -1.483985226 -5.2513945737 8.1915742935
H -1.8400769235 -3.1036406205 9.2679239623
H -0.1170750035 -2.9062967783 9.6698177049
H 0.2650490181 -5.232540273 8.5088280787
H -0.3450525285 -5.3867543883 6.8401138694
C 0.5872064385 3.3549425272 8.9603133022
N -0.2966622759 2.1897735077 9.08963328
H 0.2334369625 -2.5123405323 3.2235146701
H 4.7657177379 -1.2135911877 -0.6022630754

Ibb
C -0.6574723502 1.1106184855 -0.1171284636
C -0.5929631007 0.4519697073 1.1412996391
C 0.6861297982 0.3239480199 1.7741933414
C 1.8303769514 0.8604736179 1.1293610732
C 1.75785365 1.4766613587 -0.1061591347
C 0.4802667441 1.6034729902 -0.7228949582
C 0.7713719814 -0.3363377786 3.0270062971
C -0.3441628263 -0.8349979793 3.6738572321
C -1.6095198983 -0.7064506019 3.0328534872
C -1.7288138009 -0.0866383498 1.8056782361
H -2.7050089662 0.0044315238 1.3304129228
H -1.626515255 1.2147971042 -0.6042024064
H -2.4822835445 -1.0991856145 3.5459040124
H 0.4291726939 2.0882068772 -1.6932249445
H 2.7878348332 0.7437902009 1.6329755772
H 1.7556902165 -0.4148648617 3.4839410884
P 3.3314462367 2.1432037973 -0.8822192687
N 2.6724710273 2.6083117933 -2.366137725
N 4.2199201574 0.7095264067 -1.2293107543
P 3.322018936 2.8636235068 -3.7612442419
P 4.5310659524 -0.6050163887 -0.4511610998
N 4.6906611014 3.8425865995 -3.7403171625
N 2.1180246044 3.5570222713 -4.7040395358
N 3.8650812593 1.5815278387 -4.7231879249
N 5.6665471578 -1.5327698584 -1.283396772
N 5.1892332997 -0.3517308272 1.0970069649
N 3.2650953497 -1.676736922 -0.2262886412

C	5.5148379496	-2.946431343	-1.6621621088
C	6.8215840996	-3.2619552528	-2.4129860931
C	7.8395875785	-2.2937956367	-1.7874537624
H	7.3794128867	-0.389765311	-0.7744462654
C	5.9428192991	-1.396961479	1.8140705348
C	7.1420302979	-0.6577271967	2.4254929901
H	7.3386570841	1.5131956632	2.8062931418
C	5.6559298568	0.9995836717	1.4925987198
C	2.9644462127	-2.4632613492	0.9821993658
C	2.2115468983	-1.8177069579	-1.2499186251
C	1.4691136768	-3.0861370105	-0.8120449847
C	1.5543284876	-3.0211737355	0.7209841991
C	0.7079641386	3.6780461368	-4.2949231717
C	0.9008605454	4.1614488455	-6.6417808885
C	0.1035457981	4.5664834233	-5.3912599565
C	2.3272221179	3.9747029228	-6.0970255479
C	4.6631289885	5.0302255245	-2.8526799959
C	5.8515117022	5.8753045505	-3.3342397884
C	5.9104943517	5.5608009491	-4.8373314379
C	5.621525357	4.0542790326	-4.8687604324
C	3.6500562747	-0.6354240123	-5.5353474423
C	4.823973974	-0.6040046782	-4.5464057176
C	5.1479349957	0.8918464588	-4.4249030658
C	2.8343669345	0.5922783389	-5.1157856439
H	6.9985005867	-0.4062254117	-2.503850488
H	6.7018558	-3.0347995385	-3.4794322462
H	8.7241360984	-2.1308324039	-2.4125528045
H	8.1763253858	-2.6753707809	-0.8144116652
H	7.1083271525	-4.3150610686	-2.3232751176
H	4.6334429451	-3.108117213	-2.2941248305
H	5.400999178	-3.5882015077	-0.7755413614
H	6.2470244984	-2.1966994362	1.1296140931
H	7.9577274731	-0.5955856101	1.6936089159
H	7.5299262534	-1.1532148636	3.3226097568
H	5.9788852443	0.7293647863	3.6231252696
H	2.9945058415	-1.8362010994	1.8789459369
H	4.8161347421	1.6564067873	1.7417951788
H	3.6952388115	-3.2771793945	1.1133599121
H	1.3986257655	-3.9893430482	1.2080055182
H	0.8046748771	-2.3258851419	1.1097517071
H	0.4391782625	-3.1124523801	-1.1831380707
H	1.9917392627	-3.976457076	-1.1875231124
H	1.5501182436	-0.9425782933	-1.2478912423
H	2.6419970983	-1.9053290515	-2.2548817266
H	4.5085488044	-0.9766198483	-3.5682156016
H	5.6865440586	-1.1928364665	-4.8783518251
H	5.9030025586	1.1944206521	-5.1640871357
H	4.0106366061	-0.5168409596	-6.5658553524
H	5.50678674	1.1455280749	-3.4243571956
H	3.0626832219	-1.5590750123	-5.4854873665
H	2.1793384423	0.3456430058	-4.2663398269

H	2.2091132717	0.9888385955	-5.924326017
H	4.7410110514	4.729403455	-1.8034786932
H	3.7198734431	5.5853673381	-2.9733597902
H	2.8961500865	3.2225502595	-6.6585595191
H	2.892344754	4.9196584919	-6.1432075274
H	0.8530803819	4.8978639298	-7.4511667254
H	0.5234092559	3.2068454999	-7.0311184229
H	0.2824708742	5.6242885089	-5.1575244722
H	-0.9766257648	4.4213910484	-5.4998972848
H	0.6298626657	4.108555964	-3.2936645753
H	0.2169324653	2.6924720674	-4.2714932521
H	5.7204645903	6.9400771338	-3.1142358298
H	5.1179566381	6.1064690175	-5.3670130294
H	6.8691760682	5.8166472451	-5.3020819189
H	6.7759884227	5.54033318	-2.8457809745
H	5.1920296176	3.7137228546	-5.8152246039
H	6.5475855512	3.4840000886	-4.7027809686
H	6.2011857036	1.4832620294	0.6718076813
H	5.3229263811	-1.8527234117	2.599911602
C	6.5693728511	0.7409773809	2.6998200183
C	7.0062616263	-1.0196358322	-1.590938662
P	-0.1269030223	-1.6866199688	5.3318643947
N	-1.7629352099	-1.9220187336	5.6807518145
N	0.3578452311	-0.3952659843	6.3627066854
P	-2.5085177182	-2.196248128	7.0235035063
P	1.364842962	0.7885849787	6.2393546068
N	-1.8260682729	-3.397037699	7.9849373309
N	-4.0793386665	-2.6074531778	6.597020595
N	-2.7347451628	-0.9674498557	8.1649176529
N	1.5356401584	1.5787364336	7.7186945154
N	2.935234585	0.315706013	5.7872759435
N	0.9568858947	2.0318477511	5.1953973677
C	1.362445531	3.0209938861	7.9525146141
C	1.6008656987	3.1735414857	9.4662094484
C	2.5505297159	2.0111766129	9.8010813482
H	2.7780762359	0.1390011059	8.6579594917
C	4.1144906892	1.1679286203	6.0265695615
C	5.1914421284	0.2012649512	6.5404131196
H	5.2638403329	-1.9958992875	6.2879234142
C	3.3108875405	-1.1192409722	5.7989474517
C	1.8527528745	2.7597862113	4.2804083797
C	-0.4546306174	2.4178213131	5.0050663988
C	-0.3520662008	3.7501601777	4.2525618299
C	0.8941358491	3.5531624685	3.375160041
C	-4.6228094164	-2.510122845	5.2311585555
C	-6.4244930104	-2.8650555951	6.7828744563
C	-5.9858674833	-3.2064830799	5.3494830169
C	-5.1121460409	-2.9642926333	7.5790669242
C	-1.3416627568	-4.6206262236	7.3013688126
C	-1.1391257843	-5.62418849	8.4460239026
C	-2.2271434344	-5.2209031515	9.45360984

C	-2.1822467678	-3.6885497173	9.3895532162
C	-3.1447464239	1.3050686919	8.70450719
C	-1.6683411856	1.0055225652	8.9998737734
C	-1.6181402275	-0.5282760076	9.0430627283
C	-3.5073685764	0.207687166	7.6979237556
H	1.1908393245	0.3416780234	9.4167804629
H	0.6547907558	3.0457197666	10.0064284081
H	2.5603952289	1.7519483347	10.865246157
H	3.5769007551	2.2628290116	9.5026982329
H	2.0062868794	4.1571904761	9.7257626418
H	0.3627714348	3.3654750053	7.6620543493
H	2.0899711366	3.6098612873	7.3738928538
H	3.8823156132	1.9601285219	6.7470220425
H	5.0879377536	0.0656470452	7.6247178343
H	6.2081421837	0.5575462474	6.3399269468
H	5.2289209406	-1.0692384031	4.7812563613
H	2.476898225	2.0683539343	3.7054299168
H	2.9076791491	-1.6461745467	4.9278566753
H	2.5208407007	3.4344659262	4.8390487028
H	1.3377132358	4.4928415382	3.0301563378
H	0.6421384461	2.9575162293	2.4929085484
H	-1.2545488308	3.9687604893	3.6721199646
H	-0.193942948	4.5725133559	4.9635458685
H	-0.9865359202	1.6616533707	4.4147503869
H	-0.9743177532	2.5137684574	5.9661888714
H	-1.0365684005	1.3556325409	8.1790910795
H	-1.3109439256	1.4617470949	9.9299510208
H	-1.790088959	-0.9012987877	10.0625818379
H	-3.7494885762	1.1992597211	9.615082044
H	-0.6600597052	-0.9095097859	8.680829628
H	-3.3134009026	2.3093098982	8.2999454082
H	-3.2022332323	0.5029829007	6.6825184167
H	-4.5796571423	-0.0199285913	7.6790368947
H	-0.426041477	-4.41168234	6.7398077092
H	-2.0945564182	-4.993201079	6.5893759931
H	-5.0928732147	-2.2875758415	8.4430096448
H	-4.9578908375	-3.9879471057	7.9569013226
H	-7.2043159532	-3.5276271615	7.1734691478
H	-6.8054669801	-1.8361033192	6.8207001794
H	-5.8626360262	-4.2922885166	5.2425683827
H	-6.6960272614	-2.869344322	4.5868420827
H	-3.9521667215	-2.9861175007	4.5116724105
H	-4.7426096995	-1.4583315609	4.9273680327
H	-1.2189517296	-6.6625522237	8.1072743947
H	-3.2070377839	-5.582556509	9.1137941385
H	-2.0538598998	-5.6064188816	10.4644858493
H	-0.1460385837	-5.4902679545	8.8946752458
H	-3.1259878789	-3.213985241	9.6730674837
H	-1.402806791	-3.3003676151	10.0619636819
H	2.9076663408	-1.6236046754	6.6864957837
H	4.4384878328	1.6535516616	5.0944666715

C 4.8468188136 -1.1042083422 5.8078839542
C 2.0106574778 0.8782764216 8.917101909

IbbH⁺

C -0.7429762488 1.0044853004 -0.1699128998
C -0.6558219624 0.3922109743 1.1108142374
C 0.6244987181 0.3194039129 1.7541229192
C 1.7557754896 0.848425595 1.0860246221
C 1.6495950882 1.4151891885 -0.1710756727
C 0.3758488147 1.5023293894 -0.80232946
C 0.7327130803 -0.2979595024 3.0250581277
C -0.3677895262 -0.8196326594 3.6792825739
C -1.6342530233 -0.7408205797 3.0312951814
C -1.7764043703 -0.1567640646 1.7891173583
H -2.7549191739 -0.1053533143 1.3147203116
H -1.7155819768 1.0714889434 -0.6530496621
H -2.4928667122 -1.1472360931 3.5579535675
H 0.3050330081 1.9611356124 -1.7829169765
H 2.7177250834 0.7644266097 1.5846192579
H 1.7167563648 -0.330630505 3.4876614743
P 3.152272695 1.9321602738 -1.0397030529
N 2.6869702204 2.6255908436 -2.4044187438
N 4.2420797776 0.7439452074 -1.1918896618
P 3.3297712329 2.8037434299 -3.8473139668
P 4.6153015985 -0.5763038778 -0.3893552286
N 4.7111149387 3.7367454483 -3.7989093
N 2.1133642311 3.4801104685 -4.752210108
N 3.8235846613 1.4613681106 -4.7046488105
N 5.7199676689 -1.4652679147 -1.2649823817
N 5.3197367668 -0.2021169018 1.0829455516
N 3.3480468191 -1.603477675 -0.1051740699
C 5.6260787625 -2.9177543074 -1.5324277481
C 6.9216947911 -3.2220314164 -2.3015527439
C 7.9189356983 -2.1928610355 -1.7460646664
H 7.4138382291 -0.2587348442 -0.8127765836
C 6.1805717862 -1.1563798678 1.8142256384
C 7.2784872383 -0.2733399547 2.4197454059
H 7.1991460122 1.9008414549 2.8134681873
C 5.5657336687 1.1907262399 1.5307814199
C 3.1333022885 -2.3895303756 1.1311998739
C 2.3216446549 -1.8987441601 -1.1299509473
C 1.6864112507 -3.2000527424 -0.6245162963
C 1.766101994 -3.0530865471 0.903015819
C 0.7068276432 3.6456873088 -4.3194054707
C 0.871562062 4.0611619326 -6.6840805016
C 0.1024922628 4.5119319605 -5.4323107196
C 2.3050077129 3.876356403 -6.1624032091
C 4.7093144301 4.9409821079 -2.9245110221
C 5.8663660526 5.7945105286 -3.4661165113

C 5.8878300605 5.4457422834 -4.9618825375
C 5.6266712265 3.9357350707 -4.9517895519
C 3.6008531267 -0.7434848776 -5.5420941472
C 4.7869262812 -0.7167620758 -4.5668167599
C 5.1205127915 0.7769333139 -4.4557140425
C 2.7857825439 0.4828282031 -5.1157529917
H 7.0210333239 -0.3638217663 -2.5377199485
H 6.7675715483 -3.052213879 -3.3739576714
H 8.7853635275 -2.0348768545 -2.3953904822
H 8.2874061667 -2.5140087748 -0.7634732382
H 7.2461123407 -4.2583142685 -2.1681474792
H 4.7327587983 -3.1607989024 -2.1167528577
H 5.577484258 -3.4913412994 -0.5965220627
H 6.5742482033 -1.9195869085 1.1354830639
H 8.0729739856 -0.1039823372 1.6822849694
H 7.732130194 -0.7207042487 3.3096084016
H 5.970110358 0.9442569014 3.6497045271
H 3.1378340958 -1.7400442617 2.0116446265
H 4.6375137377 1.6873708264 1.8348319723
H 3.923716096 -3.1434532137 1.2576563977
H 1.6806338796 -4.0045231229 1.4357973764
H 0.9703662899 -2.3942947028 1.2629976537
H 0.6630178546 -3.3282304493 -0.9898698143
H 2.2786745279 -4.0618211669 -0.9581275303
H 1.5870068474 -1.0854229869 -1.1727890212
H 2.7679886114 -2.0025914821 -2.1256537875
H 4.4856275065 -1.0927743973 -3.5843885363
H 5.6417676793 -1.3079252992 -4.9110066409
H 5.8427700693 1.0786928808 -5.2249466088
H 3.9515731692 -0.6223474605 -6.5744609548
H 5.5186935405 1.0421550629 -3.4741592021
H 3.0143599225 -1.6662650502 -5.490693737
H 2.124508573 0.2331210933 -4.2724194657
H 2.1689014896 0.8916002909 -5.9225834026
H 4.8459584006 4.6565990238 -1.8763030995
H 3.7545440225 5.4792140093 -3.0060782694
H 2.8608753079 3.1110737918 -6.7171873688
H 2.8723907667 4.8164642614 -6.224681294
H 0.8183903554 4.7759393545 -7.510690555
H 0.4777020733 3.1014362432 -7.0417896132
H 0.2987685625 5.5723948258 -5.2300283137
H -0.9802070131 4.3790444892 -5.5191006271
H 0.6560561349 4.1103992905 -3.3323203112
H 0.2031143509 2.6697907086 -4.2614362506
H 5.7186037505 6.8603224004 -3.2680393727
H 5.0742515881 5.9655278121 -5.4842443656
H 6.8292942949 5.7053657865 -5.4558887973
H 6.8113107604 5.4922592639 -2.9975069034
H 5.187472308 3.5627444731 -5.8806806274
H 6.559108502 3.3824200143 -4.7769186563
H 6.009163586 1.7811495999 0.7193958921

H	5.6072422231	-1.668730262	2.5981158988
C	6.5341334298	1.0373445912	2.7159707075
C	7.0552280402	-0.9327112714	-1.5995332897
P	-0.1384717443	-1.6738237499	5.3419705063
N	-1.7666787956	-1.9136919311	5.6811433354
N	0.3564254182	-0.3829748047	6.3585086768
P	-2.4984090979	-2.240741675	7.0290681263
P	1.3343354698	0.8231621	6.2412118932
N	-1.7811776989	-3.4576876354	7.9377908012
N	-4.0650913393	-2.6486315444	6.6028268929
N	-2.713136172	-1.0433149683	8.1980139042
N	1.5150551606	1.6097994534	7.7130706643
N	2.9077767469	0.386968448	5.7447862384
N	0.8774709395	2.0487940564	5.1924118576
C	1.2898714203	3.0437221067	7.9667811375
C	1.5170683283	3.1833424633	9.4830763335
C	2.5006450464	2.0472888178	9.809180426
H	2.7964381624	0.1971588962	8.6472332648
C	4.0712445524	1.2598935884	6.0014583721
C	5.1740293828	0.3091130917	6.490193159
H	5.2915752445	-1.8833066888	6.2128116047
C	3.3107608242	-1.0419775932	5.7782614043
C	1.7428763931	2.8237334524	4.2927336452
C	-0.5446130052	2.4162880388	5.0454364936
C	-0.4854324531	3.7523155262	4.2941812496
C	0.7518673817	3.5877341729	3.3972845665
C	-4.619307193	-2.5564867027	5.240272896
C	-6.4018097615	-2.9549132793	6.8070341378
C	-5.974493493	-3.2683454037	5.364173376
C	-5.0810085328	-3.0558741176	7.5865020703
C	-1.3222667901	-4.6688334795	7.2124693275
C	-1.1058129474	-5.7046125476	8.3253076322
C	-2.1689238394	-5.3199244697	9.3655535797
C	-2.1122862856	-3.787412357	9.3434700358
C	-3.1543686537	1.1996793945	8.8281677946
C	-1.6676223934	0.9153779679	9.0844111162
C	-1.5956783707	-0.6178964241	9.0811751439
C	-3.5217632163	0.1305849108	7.792923503
H	1.1964169869	0.3409053368	9.3925703775
H	0.5729775841	3.0208912673	10.0163646745
H	2.5107858312	1.7749394487	10.8694127732
H	3.5208771637	2.3348190231	9.5227849559
H	1.8910823157	4.1750978953	9.7564759688
H	0.2800719094	3.3566699661	7.6776040442
H	2.0002779198	3.6630712282	7.3989563748
H	3.8255464362	2.0310335404	6.7395081696
H	5.0888960542	0.1600484732	7.5736315006
H	6.1811497377	0.6875680046	6.2827919252
H	5.2021135639	-0.9424406384	4.7161915522
H	2.3978262994	2.1623597804	3.7132029078
H	2.8937545799	-1.5942479928	4.9292099761

H 2.3843034693 3.5200102467 4.8562075587
H 1.161890007 4.5399253014 3.0442013248
H 0.4997290693 2.9804835203 2.5214620715
H -1.4019614407 3.9535618752 3.7303614903
H -0.3330130772 4.5765327278 5.0029488159
H -1.0833222218 1.6522619332 4.4709595798
H -1.0388136549 2.5022847679 6.019948845
H -1.0578496176 1.3013718457 8.2622063858
H -1.2995457745 1.3482632077 10.0210356835
H -1.7553906747 -1.0224070312 10.0899475961
H -3.7371106624 1.0533134207 9.7467256578
H -0.6360661883 -0.9790453476 8.7027349935
H -3.3487567835 2.2138709491 8.4625007103
H -3.2501576001 0.4665344488 6.7803998885
H -4.5890514266 -0.1177637728 7.7937992693
H -0.4153884226 -4.4539673275 6.639046838
H -2.0932768778 -5.015019458 6.5069163242
H -5.0619967421 -2.4060489897 8.4700272496
H -4.9037959764 -4.0876510193 7.9285333852
H -7.1701047489 -3.6323709388 7.1933396372
H -6.7925767213 -1.930931613 6.8676200589
H -5.8420075801 -4.3507956264 5.2385800633
H -6.6961503325 -2.9269198767 4.6147394662
H -3.9501360762 -3.0265297734 4.5148124356
H -4.7511950636 -1.505020167 4.940830869
H -1.2026943361 -6.7321759127 7.9601170141
H -3.1586447026 -5.6648527189 9.0379533598
H -1.9774352072 -5.7339315535 10.3611710407
H -0.1024783383 -5.5922401397 8.7563043309
H -3.0460872852 -3.3136790925 9.6583594096
H -1.3153505643 -3.4231081938 10.0074962477
H 2.9415803322 -1.5372527598 6.6852538093
H 4.3777879388 1.7748929467 5.0785174901
C 4.8444008889 -0.9966055209 5.7519952248
C 2.003757244 0.9082117701 8.9089943442
H 3.7283712184 2.922291625 -0.2085821503

IIaa
C 0.9446103824 1.1196904911 1.4369541773
C 1.1561208015 0.6624565521 2.7327938114
C 2.4206812691 0.7784576804 3.3462815672
C 3.4856192761 1.3104864301 2.6256690057
C 3.2824525883 1.7161141336 1.2949215172
C 2.0184565753 1.6244341291 0.6896447752
C 0.2478130704 -0.030320864 3.6474013454
C 0.9271628651 -0.2828819958 4.8578241571
C 2.3571165212 0.2241215609 4.7601937912
C 0.2652387538 -0.9337370713 5.8938014043
C -1.0670657348 -1.3399999516 5.7107146378
C -1.7384086256 -1.1163354066 4.4980708582
C -1.060369035 -0.4678640334 3.4565058773

H	-1.5572608776	-0.3014798011	2.5022499532
H	-0.0394349136	1.0296355612	0.9869218618
H	4.1136584215	2.0920953832	0.7050480231
H	-1.6016452287	-1.8365086981	6.5147193706
H	0.7691843541	-1.1328525523	6.8384993689
H	4.4743527247	1.3954416664	3.0743451406
H	3.0877657919	-0.5808231566	4.9220813488
H	2.5720790824	0.9979082902	5.5113069344
P	-3.4666435231	-1.700756605	4.1292850258
N	-3.8880957018	-2.2699325438	5.671958772
P	-5.2618375307	-2.8777387969	6.1015906277
N	-4.975131532	-3.9596292704	7.3746654953
P	-4.554144652	0.9901807751	3.0573109259
N	-6.3885834668	-1.6682049878	6.5078056825
N	-4.4021789994	0.6490371177	1.3827524281
N	-4.2923439301	-0.2201813885	3.9834410398
N	-6.0952283442	1.6053078435	3.4336420503
C	-6.0753269863	-4.6257674662	8.0618039504
C	-3.759299079	-3.847952915	8.1762920258
C	-2.3140404885	2.5269144262	2.5351627525
C	-4.7303418371	1.676878185	0.3914357734
C	-4.8554553807	-0.6794282197	0.9587377145
C	-6.9046472897	-3.1948852252	3.9385437241
C	-6.6138665356	2.8408511503	2.8595186353
C	-7.132322534	0.7059102812	3.9227238337
C	-7.7292401998	-1.9866366311	6.9926855583
C	-5.8638861201	-0.4120814451	7.0528459419
C	-3.6282454356	3.0524602699	4.5041869946
N	-3.624343662	2.4081461999	3.1856911407
H	-4.4328666634	-0.8986806432	-0.0291885746
H	-4.5078979163	-1.4442578709	1.6564409545
H	-5.9550023358	-0.7379686582	0.8782599352
H	-7.689617854	-3.8740960446	3.5811490888
H	-6.2340876573	-2.9615490216	3.0977785451
H	-7.3721407534	-2.2688235274	4.2711539649
H	-4.8458029616	-4.9542998586	3.81385193
H	-6.3630689614	-5.7825103549	4.2636808175
H	-5.0679620553	-5.5801840354	5.466193487
H	-5.8113190575	1.7172804154	0.1789182165
H	-4.2089761191	1.4503305917	-0.5473538324
H	-4.4073489862	2.6618236877	0.7319833228
H	-2.9659744875	-3.4056550909	7.5748197887
H	-2.2859941323	1.9538765365	1.6093610741
H	-3.9144505952	-3.2300382588	9.0763852348
H	-3.4473692824	-4.8508366602	8.4988989118
H	-2.1292651559	3.5821198151	2.2955364627
H	-6.4300424072	-4.0577547223	8.9376604166
H	-6.9129669339	-4.7820076372	7.3792124963
H	-5.7375371139	-5.6098335649	8.414334623
H	-4.9367596772	-0.1477147365	6.5455557839
H	-2.9039060284	2.5823451021	5.1868854578

H	-5.6888265641	-0.4761792475	8.1399759156
H	-3.3593920599	4.1097518293	4.3839385628
H	-6.5973125829	0.3832784299	6.8705618188
H	-4.6211013765	2.9931929535	4.9546713265
H	-7.7653527226	-2.0838636486	8.0900276259
H	-8.4172810363	-1.1800539242	6.7042236769
H	-8.0890885134	-2.9162975383	6.5478860071
H	-6.6725202132	-0.1470829456	4.4199665145
H	-7.2138167233	3.370976999	3.6134946376
H	-7.7648445783	1.2377291473	4.6482355131
H	-7.7841810073	0.3455543194	3.1080101892
H	-7.2615817499	2.6542697166	1.9870763061
H	-5.7944969801	3.4938246222	2.5552120083
C	-5.5758145217	-5.1077023271	4.6234563146
N	-6.1921733058	-3.843062348	5.0465080997
H	-1.5032340621	2.1716253072	3.183671651
P	1.6353907615	2.1549417482	-1.0564652847
N	3.2294621444	2.4348422082	-1.6008683101
P	3.5958545223	2.9558172034	-3.0328144827
N	5.1152105009	3.6900580899	-2.91528821
P	0.8325888991	-0.8055154752	-1.6049952841
N	3.6437843387	1.7274000706	-4.1943542978
N	-0.6455101819	-1.0407324707	-0.7959701247
N	1.1619648586	0.6977454707	-1.7804239319
N	0.828080696	-1.4973379302	-3.1665086442
C	5.7909350831	4.2267512926	-4.0873204842
C	5.6136692561	4.2181217505	-1.6495353188
C	1.8775499118	-1.9313908352	0.6826851598
C	-1.1867871519	-2.3442961847	-0.4176481906
C	-1.673474074	-0.0087526772	-0.8868786354
C	1.2733463582	3.6860569545	-4.2890074678
C	0.3155118395	-2.8513107698	-3.3468222744
C	0.6126439987	-0.6343681774	-4.3241191746
C	3.7305367323	1.9242222184	-5.6361044822
C	4.087439768	0.4039185017	-3.7619290452
C	3.1356827959	-2.2149286106	-1.3657519336
N	1.8222021366	-1.9239309528	-0.7847810928
H	-2.1906643888	0.0842451517	0.0764635326
H	-1.2249632968	0.9523247623	-1.1437971017
H	-2.4282024366	-0.2567730434	-1.6532444306
H	0.973033234	4.3145031031	-5.1388879541
H	0.5273289592	3.787662674	-3.4881897741
H	1.281839081	2.6438933297	-4.6106886098
H	1.9580290855	5.5203954803	-2.3851338134
H	2.2265542339	6.1479516605	-4.0341373492
H	3.6117874922	5.7587243529	-2.9999777419
H	-1.9522180985	-2.6880848082	-1.1324784295
H	-1.6547202668	-2.2755820188	0.5740414779
H	-0.3960526382	-3.0935583498	-0.3710582811
H	5.0519133809	3.7783892415	-0.8256656362
H	0.9092886411	-1.6858478577	1.1150507582

H 6.6765048729 3.9624178667 -1.5372501279
H 5.5198151286 5.3166898387 -1.6022631567
H 2.1638653423 -2.9374063585 1.0170503301
H 6.8716246732 4.046558118 -4.0058874791
H 5.4380389038 3.7478470632 -5.0002940899
H 5.6353866564 5.3141880726 -4.1922173136
H 3.9248154539 0.2977192479 -2.6908835999
H 3.8901055224 -1.473692684 -1.0581574207
H 5.1540501065 0.241969185 -3.9899000278
H 3.4660415204 -3.2030124477 -1.0193712204
H 3.4983303426 -0.3654572509 -4.2760842088
H 3.0760673502 -2.2262235974 -2.4548878083
H 4.7532779469 1.7514326013 -6.0107212696
H 3.0660091427 1.209779522 -6.1435094726
H 3.4210013971 2.9346818698 -5.9077882967
H 1.0758814087 0.3353330095 -4.1428991247
H 0.7489579595 -3.2843388867 -4.2581381798
H 1.0750428582 -1.0981387461 -5.206677569
H -0.45911809 -0.4827958741 -4.5414367694
H -0.7829675609 -2.8779273008 -3.4490834062
H 0.6035242017 -3.4814110222 -2.502516988
C 2.6015984144 5.4486711997 -3.2759120494
N 2.6116259923 4.0966729292 -3.8408060555
H 2.6122600239 -1.2100484759 1.0654038311

IIaaH^+
C 0.6464138171 0.312380841 1.2407519502
C 0.9385169552 -0.0795051564 2.5428298437
C 2.2114364511 0.1336466385 3.1106277231
C 3.2102590966 0.7149664867 2.3354753245
C 2.9128334913 1.1077679556 1.0193936224
C 1.6354355627 0.9257471938 0.4592234957
C 0.0689222947 -0.6656829428 3.5576518544
C 0.7775122289 -0.770628007 4.774013689
C 2.2135324287 -0.3224339214 4.5611413886
C 0.1199325205 -1.1866388235 5.9284858125
C -1.2527809017 -1.4774153546 5.8717001318
C -1.9470245782 -1.3816203531 4.6545176403
C -1.2758147738 -1.0035042777 3.483269198
H -1.8066582124 -0.9022198716 2.5427329234
H -0.3384408393 0.1286832545 0.8169443742
H 3.6877719281 1.5499441854 0.3997463956
H -1.7909584183 -1.7587884724 6.771445286
H 0.6494022355 -1.2621724321 6.8753852383
H 4.2088905994 0.8747676564 2.7376752732
H 2.9193116736 -1.1464714207 4.7378375653
H 2.4958819857 0.4837554877 5.2514944503
P -3.7323888511 -1.6159157997 4.5191814762
N -4.2883111234 -1.9289311847 5.9995385399
P -5.7424574793 -2.3665766184 6.4795695897
N -5.5272562261 -3.0664075956 7.9780544363

P	-4.258103185	1.0352832208	3.1413188703
N	-6.7703792538	-1.0461972555	6.5748011858
N	-3.406463546	1.0435645388	1.7016530783
N	-4.4449937229	-0.4422950267	3.6879121677
N	-5.8147602071	1.6213557207	2.9375430479
C	-6.6805381977	-3.399443906	8.8143880465
C	-4.2813886116	-3.7217525847	8.3845136221
C	-1.9771987027	2.1188601828	4.2409081937
C	-2.8358463002	2.244451666	1.0714555516
C	-3.6337121169	-0.0413420033	0.7400798725
C	-7.0729310062	-3.0390796568	4.1858259234
C	-6.0407058533	2.9087196951	2.2823154229
C	-6.9703427923	1.1190442878	3.6755873942
C	-8.2325815637	-1.1247006746	6.6259533754
C	-6.2359518752	0.1877928599	7.1606682438
C	-4.1161820274	2.7511220762	5.2256909458
N	-3.436161217	2.1821261701	4.0583052748
H	-2.6933967307	-0.2683521394	0.2257673428
H	-3.9826856436	-0.9400097453	1.2515279445
H	-4.3788104003	0.2453322671	-0.0162678635
H	-8.0368888417	-3.5161799901	3.9717627929
H	-6.3557958768	-3.3431931001	3.4103260179
H	-7.1983520686	-1.9578540564	4.1283630392
H	-5.5008546094	-5.16990858	4.9512104244
H	-7.2205549444	-5.4399064065	5.3280540846
H	-6.065269157	-5.154254599	6.6389322578
H	-3.4536791508	2.5704618513	0.2224944962
H	-1.8325027217	2.0185648645	0.692422895
H	-2.7627199406	3.0620391857	1.7886132538
H	-3.469196881	-3.4165949334	7.7255548035
H	-1.4870085457	1.7334349059	3.3486272125
H	-4.0360387789	-3.4250669472	9.4114292861
H	-4.3785672462	-4.8174030539	8.3544833049
H	-1.60643166	3.1330028285	4.4293405491
H	-6.4565058204	-3.151666058	9.8587310461
H	-7.5614758059	-2.8319191149	8.5127249665
H	-6.9260591843	-4.4704802639	8.7594324311
H	-5.1552409746	0.2310341213	7.0235704646
H	-3.941458137	2.1511291155	6.1312168662
H	-6.4647011257	0.2520192612	8.2344456281
H	-3.7315767758	3.7617412055	5.4038497164
H	-6.6861591909	1.0501247068	6.656702694
H	-5.1917105373	2.820476539	5.0552414921
H	-8.6062678239	-0.9386018285	7.6433013698
H	-8.6593627081	-0.3602137328	5.9638147798
H	-8.5798607756	-2.1044607558	6.2973057936
H	-6.6997834608	0.217177746	4.2231528229
H	-6.2189970469	3.7104361675	3.015266947
H	-7.3367928924	1.8731942663	4.3899601279
H	-7.7866902541	0.8878515383	2.9785995087
H	-6.9197086933	2.8367624089	1.6302919755

H	-5.1869199227	3.1928390767	1.6675935154
C	-6.3262633865	-4.8763292009	5.6170249475
N	-6.6207557391	-3.4417444551	5.525465383
H	-1.7025430635	1.4771271084	5.0891592747
P	1.1072584761	1.5808942647	-1.2134555618
N	2.6488560147	1.8785757999	-1.8733742301
P	3.0036836692	2.9815423073	-2.9384879574
N	4.1556014499	4.0266999746	-2.2779478957
P	1.0165813335	-1.2549324319	-2.196153793
N	3.6545966623	2.2947691734	-4.3347746356
N	-0.2656455049	-2.3560566272	-1.9695817959
N	0.4420997191	0.180625365	-1.8988149245
N	1.6625997939	-1.3327952831	-3.762241667
C	4.8855824099	4.9807064407	-3.1050828938
C	4.1665198316	4.3569609393	-0.8556426619
C	1.9901889392	-2.5558650765	0.0274222456
C	-0.0921582795	-3.7574609996	-2.3477204068
C	-1.6379861919	-1.8871162881	-2.1517766941
C	0.715250035	3.3577175607	-4.3785202442
C	2.2840054275	-2.530043338	-4.3208631874
C	1.1938671521	-0.3983965434	-4.7813745624
C	3.753259485	2.9452494733	-5.6375932448
C	4.5523014384	1.1528949697	-4.1825126261
C	3.6360396137	-1.5665437423	-1.467427726
N	2.2451757317	-1.9971449629	-1.3023928118
H	-2.3081653803	-2.4673735029	-1.5016620325
H	-1.6975087341	-0.8302158259	-1.8911497461
H	-1.9823912692	-2.0128946444	-3.1923086354
H	0.2807217642	4.1104469782	-5.0491167098
H	-0.0809754005	2.9554088339	-3.7351912571
H	1.1118498733	2.5453652812	-4.9890050912
H	0.5859610982	4.7907714648	-2.0310302865
H	0.8377037921	5.8386001818	-3.4547467517
H	2.1508499652	5.6015639236	-2.2933546327
H	-0.2944186393	-3.9311820178	-3.4172297483
H	-0.7899690947	-4.3753847706	-1.7677288145
H	0.9222330296	-4.0933361676	-2.1242885018
H	3.5420682862	3.6542775581	-0.3051102304
H	0.9262044174	-2.754191826	0.1611554785
H	5.1926472302	4.2964392239	-0.4677193513
H	3.7943307744	5.3785148029	-0.6739227959
H	2.5424499109	-3.5002497802	0.1336479613
H	5.8961381383	5.116043186	-2.6985853218
H	4.9833836247	4.6178184478	-4.1281053313
H	4.3947768867	5.9675083975	-3.1334758576
H	4.3949249752	0.692121203	-3.2082229742
H	3.8859378721	-0.7444437805	-0.7843798706
H	5.6068870915	1.4574391903	-4.2734374882
H	4.2978617359	-2.4176299106	-1.259380667
H	4.3417921225	0.4075160124	-4.9614341611
H	3.8111120498	-1.2301632379	-2.4879906724

H 4.7966363399 3.1995683958 -5.8821941805
H 3.3853814938 2.264984138 -6.4194587392
H 3.1556618803 3.8572810457 -5.6603084983
H 0.691912831 0.4395900861 -4.3021570085
H 3.1427748249 -2.2397345964 -4.9423710073
H 2.0459359792 -0.0158371906 -5.3585230357
H 0.4933612243 -0.8858230735 -5.4788102493
H 1.5846919053 -3.0964994772 -4.955678745
H 2.6455280458 -3.1859508133 -3.5277192469
C 1.3157657391 5.1064114304 -2.7919094561
N 1.7920815396 3.9805507567 -3.5998805953
H 2.3188365658 -1.8685613484 0.8168987099
H -3.8968847257 -2.7666825624 3.7158751128

IIbb

C 0.5893534355 -0.4556260876 1.8744405033
C 0.6374568086 -1.3567214618 2.9368756353
C 1.865391767 -1.7732384459 3.4901885031
C 3.0537031016 -1.2801291787 2.9597515998
C 3.0031450813 -0.3959844588 1.8701206847
C 1.780131109 0.0176811123 1.306753429
C 1.6152303751 -2.7700796352 4.6113097292
C 0.0990584881 -2.8745998681 4.6322128222
C -0.4489892976 -2.0385290257 3.6410254203
C -0.7587933259 -3.6011282207 5.4550640147
C -2.1463765523 -3.4689329106 5.2884904305
C -2.7028671022 -2.6174663554 4.3146163475
C -1.8249028442 -1.9226931779 3.4704478082
H -0.365922062 -0.1499571077 1.4542155435
H -2.2359903625 -1.2693205397 2.7092660024
H -2.8006277722 -4.0254960778 5.9517283625
H 3.9330187318 -0.0510593765 1.4277032408
H 4.0169818615 -1.5927807779 3.3608943602
H -0.3669396998 -4.2536552939 6.2339956869
H 2.0132537735 -2.4159411063 5.5732224072
H 2.0950114161 -3.7389400425 4.4111623798
P -4.5626583519 -2.471510358 4.0118748089
N -4.4631885045 -1.0119093081 3.1472811056
N -5.237326036 -2.0494871586 5.5120187744
P -5.3055913448 -0.301264932 2.0514343468
P -4.7488514046 -1.50832252 6.8990164239
N -5.7891645711 -1.2491219038 0.7578137472
N -4.3804072357 0.9764547475 1.47585968
N -6.783448747 0.4156129837 2.4897865781
N -5.9643643461 -0.6379010146 7.7083961623
N -4.4251466298 -2.7702806491 7.9743109172
N -3.414657806 -0.4853982146 6.9613365381
C -6.1707338468 0.8122604852 7.5317900863
C -7.6043764478 1.0708543581 8.0565602878
C -7.9795287861 -0.2141682993 8.8169228173
H -7.0620657023 -2.2049774096 8.606226707

C	-4.2465747724	-2.5617724107	9.4282571426
C	-4.8455716288	-3.8216089055	10.075403273
H	-5.2773221214	-5.7714727308	9.1188609603
C	-4.9530627387	-4.1268941643	7.7014566955
C	-2.1484089846	-0.7884540924	7.662122613
C	-3.2066889757	0.534379346	5.9010858906
C	-1.8145658981	1.1018641226	6.2072839244
C	-1.0741844188	-0.0988153903	6.8104731483
C	-3.2719431094	1.6120525141	2.2049263693
C	-3.7336265539	2.9176572779	0.2429898643
C	-2.5631497452	2.4120560674	1.1080871109
C	-4.7996022596	1.7978618743	0.3295544177
C	-4.9183603633	-2.3267221787	0.2474973945
C	-5.807572172	-3.0338899055	-0.7827819815
C	-6.6427926006	-1.8817134873	-1.3640720599
C	-6.9411476831	-1.0086577862	-0.1339279592
C	-7.9382847574	1.5971883144	4.2094315789
C	-8.1501704307	0.0920997532	4.4266877548
C	-7.8337296233	-0.4833160953	3.0416139934
C	-6.6738982787	1.6281097918	3.3409757894
H	-7.7987413418	-1.5795465866	7.1174703967
H	-8.2901673813	1.2087843674	7.2131274248
H	-9.0608172579	-0.3840781742	8.8612977292
H	-7.5942698467	-0.1821952683	9.8437298752
H	-7.6619022948	1.9701574463	8.6787208333
H	-6.0857660033	1.1227324188	6.4816621929
H	-5.4088733351	1.3607746862	8.0986350668
H	-4.7402387459	-1.6390596103	9.7535733059
H	-5.9185175361	-3.6807619633	10.2600534173
H	-4.3708604536	-4.0671117802	11.0315317855
H	-3.5928908402	-5.2260423611	8.9869518847
H	-1.9848215723	-1.8675414228	7.7259326697
H	-4.4852697102	-4.565769358	6.8173826263
H	-2.1692811454	-0.3792805122	8.6841935789
H	-0.1957163798	0.1849998265	7.4008793455
H	-0.7499772747	-0.7736050712	6.0141401963
H	-1.3297266809	1.4967107153	5.308152209
H	-1.8862176687	1.9153843982	6.9417932303
H	-3.2516442997	0.0817161004	4.907279756
H	-3.9775599974	1.3123477639	5.9431254388
H	-7.4228113077	-0.3012357376	5.1446265431
H	-9.1582233019	-0.1636399373	4.7721920766
H	-8.7185390628	-0.4551387509	2.389860148
H	-8.78853068	2.0270143186	3.6634453689
H	-7.4824430945	-1.5195127419	3.0942735931
H	-7.8124877857	2.1664412092	5.1364063603
H	-5.7818015194	1.5836432131	3.9827708369
H	-6.6000342579	2.5293613587	2.7211972523
H	-4.6013636853	-2.9854216955	1.0604583808
H	-4.0144084887	-1.9158519816	-0.2291121815
H	-5.8095396423	2.2118279254	0.4682360306

H	-4.8234393997	1.1841590275	-0.5780741326
H	-3.4392007462	3.1257964886	-0.790973255
H	-4.1367986466	3.8469353629	0.6642783882
H	-1.9010691924	1.7582122298	0.5309845981
H	-1.9532543323	3.2268898575	1.512105222
H	-2.6417292424	0.856659846	2.6741928686
H	-3.6413040007	2.2813025483	3.0014049716
H	-5.2273599576	-3.575857097	-1.5373742561
H	-6.0466799647	-1.3165365086	-2.0906283182
H	-7.5564459072	-2.2156876785	-1.8675691202
H	-6.4600108374	-3.7555399188	-0.2752155595
H	-7.0492949736	0.0516817358	-0.384650627
H	-7.87353581	-1.3276878068	0.3517084742
H	-6.0360360201	-4.1088511536	7.5117341854
H	-3.178572498	-2.4738865285	9.6712871362
C	-4.6385623377	-4.8909330268	8.9920142385
C	-7.2392739545	-1.2952112473	8.0234694961
P	1.697379202	1.2570647959	-0.1023046414
N	0.4040613977	0.6587203573	-1.0142375196
N	3.1278429583	0.7999482486	-0.9004015917
P	0.2531440229	-0.6245722344	-1.9195118751
P	4.5257803118	1.2875093378	-1.3415123737
N	-1.3754656146	-0.7228057858	-2.3231321985
N	0.5869378189	-2.1924743023	-1.4003793736
N	1.1938955736	-0.5586181357	-3.3143095164
N	4.4785521539	2.3693470254	-2.652473889
N	5.4758451238	1.839722059	-0.0492376285
N	5.5308313418	0.0871021569	-1.977465746
C	5.6903719471	2.8726427852	-3.3234299377
C	5.7419139713	4.404800346	-3.0453299547
C	4.4012233352	4.724720862	-2.3355975553
H	2.7335176184	3.3253844893	-1.8959019626
C	6.8922228699	2.2075914636	-0.1367005029
C	7.3251155468	2.3106682526	1.3448474313
H	6.0366478841	3.4649748229	2.7402201047
C	4.9161945165	2.6513354353	1.0374401217
C	5.9640568102	-0.9988620603	-1.0539469914
C	5.1220948006	-0.501754563	-3.2762170407
C	5.8652861252	-1.8391251321	-3.3152689599
C	5.7858680747	-2.3019717855	-1.8531602104
C	1.9851132873	-2.6214376736	-1.1472739922
C	0.4714068103	-4.0653325827	0.0512747617
C	1.8969473607	-3.4945759618	0.1121295489
C	-0.35048401	-2.8865162455	-0.4864007123
C	-2.4074641709	0.241542999	-1.8973371086
C	-3.4724852444	0.133010662	-2.9987906804
C	-3.3602788301	-1.3317652314	-3.4479398233
C	-1.8452737328	-1.5733321625	-3.4251881635
C	1.4349693733	-1.0160865759	-5.6284383222
C	1.9888083344	0.3904521683	-5.34719698
C	1.3806210791	0.7477747253	-3.9811187334

C 1.4664142845 -1.6710546024 -4.2409892005
H 3.0585960134 3.5807073201 -3.6192505509
H 5.8397710011 4.9611042077 -3.9843755497
H 3.9447181799 5.6570580387 -2.6857200797
H 4.5515119975 4.8146189509 -1.2536982714
H 6.601062544 4.6739963672 -2.4214205664
H 5.5959157865 2.6882059621 -4.4038054744
H 6.5747154526 2.3333751774 -2.9780754547
H 7.0181929096 3.1852015144 -0.633949797
H 8.0547180338 3.1149081546 1.4888632884
H 7.7970848491 1.3772828362 1.6689811142
H 5.7845771215 1.7163979119 2.7926519766
H 5.3707814451 -1.0035778461 -0.1347355868
H 3.9423625181 2.2750708762 1.3547982883
H 7.0146828341 -0.841087432 -0.7753605902
H 6.5362108221 -3.0559523561 -1.592415446
H 4.7964318439 -2.7281087037 -1.6536601188
H 5.4111582573 -2.5450334407 -4.0193026191
H 6.910229679 -1.6837378741 -3.6140350047
H 4.0364429975 -0.6622274658 -3.3054423852
H 5.3927310514 0.1644201434 -4.1008360658
H 3.082256507 0.3570128264 -5.2723632275
H 1.7294204268 1.1214402161 -6.1205685182
H 0.4164677371 1.2646727037 -4.1033637404
H 0.399956219 -0.9491797269 -5.9891238165
H 2.034342245 1.3842616539 -3.3845276144
H 2.0165436172 -1.5743759374 -6.3707474949
H 2.4630297499 -2.0970653025 -4.0469145081
H 0.7422353734 -2.4817083341 -4.1247018833
H -1.9849398729 1.24376241 -1.8005630177
H -2.8189680245 -0.0404513554 -0.9186863166
H -1.2486526116 -3.2067510765 -1.0258501749
H -0.6678393219 -2.2367175632 0.3380018871
H 0.1025646155 -4.4069965368 1.0238659006
H 0.4249128317 -4.9094195913 -0.6498521627
H 2.0127678631 -2.8725119518 1.0022385251
H 2.6716796389 -4.2695906451 0.1351636016
H 2.645583286 -1.758359948 -1.0166272924
H 2.3499782798 -3.2067124424 -2.0044178595
H -4.4730928498 0.4024709167 -2.6460201318
H -3.8506313885 -1.9865878368 -2.7185215048
H -3.8030640441 -1.5267722106 -4.4306363106
H -3.2196715832 0.8008093583 -3.8329335907
H -1.5875031738 -2.626590703 -3.2550873749
H -1.3915895296 -1.2712302313 -4.3825752252
H 4.7822406583 3.7002911518 0.7194697854
H 7.4531684865 1.4643148828 -0.7081240373
C 6.0020302537 2.5582734881 2.127141369
C 3.5282173395 3.4914108619 -2.6271793541

IIbbH+
C -0.5236412709 0.4338202404 -0.163456806
N -0.2915215657 0.1263745639 1.2731279809
C 0.9275098771 -0.7003925785 1.4492926967
C 1.1530790214 -1.2833661545 0.0494383468
C 0.7130911759 -0.1381968428 -0.8748815075
P -0.9282300658 1.1549350876 2.4202945357
N -0.557248953 0.384902508 3.8605652362
C -0.5298121281 1.1730170924 5.1178051968
C -1.7446824855 0.6667592002 5.9008493239
C -1.7146179354 -0.8395492587 5.5978355662
C -1.2237318786 -0.921289526 4.1328784708
N -2.4680390457 1.462168731 2.224222956
P -3.671356925 1.3111919084 1.173969033
N -4.1379251968 -0.1441500417 0.6633405202
P -4.7569885315 -1.4101242241 1.4200588549
N -3.5412551745 -2.4160585678 1.9600398624
C -2.3982263617 -2.7155189075 1.0590740489
C -1.70245084 -3.9108147428 1.7277925185
C -2.8519746939 -4.6410547924 2.4396759182
C -3.7075362036 -3.4855100502 2.9730576165
C -5.0628440839 2.2303705904 1.8859060423
C -6.3338638855 2.0353717681 1.3259937801
C -7.4319706029 2.6614349097 1.908749962
C -7.2717701551 3.4992908894 3.0349687072
C -6.005718004 3.7211239387 3.567421996
C -4.8980677202 3.0792699974 2.9931850825
C -8.6297360196 4.0002926188 3.4920900677
C -9.5784188774 3.3364614023 2.5103689209
C -8.8530628671 2.5693204744 1.5793485466
C -9.4864964638 1.8837497698 0.5440058086
C -10.880164648 1.9493025297 0.412777096
C -11.5998536819 2.691455199 1.371779416
C -10.9660885897 3.392130507 2.4064370815
P -11.7542520887 0.9303726618 -0.9181442568
N -12.6373607921 2.0325071661 -1.8658822677
P -12.8875880441 3.5812936621 -1.8434494245
N -11.5253492205 4.5427826739 -1.6378062743
C -11.353987163 5.613065603 -0.6382139538
C -9.8336051349 5.6851602202 -0.4351580788
C -9.2739066164 5.2617561341 -1.8004108534
C -10.2307307171 4.1441994685 -2.2374150402
N -10.3438383888 0.587265595 -1.8193975267
P -10.1577146096 -0.39363527 -3.0246167441
N -11.0051590931 -0.175999756 -4.4697097658
C -12.4757254268 -0.4172581733 -4.4921768474
C -13.0553443161 0.8122833296 -5.2025396448
C -11.9215688613 1.2242356531 -6.1506270722
C -10.6807410251 1.0266833495 -5.274215411
N -10.5320248937 -1.9868851052 -2.6689141814
C -10.5724993992 -2.4644724603 -1.2690100704

C -10.8963127458 -3.9606762417 -1.4050165829
C -10.2893303398 -4.3401155817 -2.7650465171
C -10.59697149 -3.1078256021 -3.6271444792
N -8.5424796104 -0.2429237243 -3.4972729172
C -7.7130054237 0.913750476 -3.0977247909
C -6.4171480248 0.7260922224 -3.9004285774
C -6.8988517321 0.0454895735 -5.1904679064
C -7.9817233916 -0.9191476018 -4.6826200104
N -13.5981512442 4.1493778585 -3.2671231778
C -12.8211852804 4.5243333035 -4.4649301744
C -13.8652933521 4.5782404957 -5.6039779074
C -15.220864808 4.6491283302 -4.8800350356
C -14.9768723228 3.7926962525 -3.6336465227
N -13.9983294222 4.0354317089 -0.6545053149
C -14.5990381605 5.3894615513 -0.5775873564
C -16.0253848954 5.1544543893 -0.0509304322
C -15.8857188377 3.8526636956 0.7525911423
C -14.9410841216 3.0216370245 -0.1197952808
N -5.6880525803 -2.203916081 0.3033029985
C -6.4720720675 -1.5605856759 -0.7706155013
C -6.8529439145 -2.7428922983 -1.6643830464
C -7.077485964 -3.8825127949 -0.6566427791
C -6.0195023424 -3.6382005827 0.4369566032
N -5.7067425626 -1.1955007771 2.7700116906
C -5.1961810809 -0.6405960567 4.0459672315
C -6.4139035459 0.0705509805 4.6516562868
C -7.6007685535 -0.7490522753 4.1214781767
C -7.1859155586 -1.0757612603 2.6814092305
N -0.1215137609 2.6064017452 2.4227126473
C 1.3499050957 2.7002784326 2.5597018722
C 1.5806646269 4.1558861244 2.9945877799
C 0.4076597578 4.9046454993 2.3423913727
C -0.7680131169 3.9354171882 2.5221798774
H -6.4661166035 1.374833847 0.473064507
H -8.9232687679 1.314589638 -0.189500079
H -12.6801024832 2.7500239206 1.3033474135
H -3.9077373968 3.2100279207 3.4178571246
H -5.87058526 4.3637767155 4.4345631262
H -11.5581615998 3.9734621782 3.1105923094
H -8.691660152 5.096833706 3.4477079962
H -8.8343254146 3.7182003107 4.5342465793
H -15.6631176734 4.0304526986 -2.8158810421
H -16.8395375514 3.3458240886 0.9308131172
H -15.0853165312 2.7205152288 -3.8612374649
H -13.8171285874 3.6588828909 -6.1986504201
H -16.0520122162 4.2817300747 -5.4907187179
H -15.4447211473 5.6805325407 -4.5807775687
H -13.6947748398 5.4201519463 -6.2824181069
H -12.0344514745 3.7936878793 -4.6941698678
H -12.3339699238 5.4929295486 -4.2979920041
H -14.5821716631 5.8843613886 -1.5555286987

H -16.7221413881 5.0041581346 -0.8852153642
H -16.3938542275 5.9980371197 0.5417215401
H -15.4202139271 4.055466971 1.7262143471
H -11.8729865176 5.3642743393 0.2915695614
H -14.4141014806 2.2404964418 0.4339738147
H -11.7524784025 6.568329109 -1.0129893821
H -9.5033650856 6.6808772844 -0.1196531578
H -9.5340815345 4.9626678819 0.3306175221
H -8.235604393 4.9156957903 -1.7519941142
H -9.3201120383 6.1009031926 -2.5066573947
H -9.9082376494 3.1662058953 -1.8667549941
H -10.3013806374 4.0735820206 -3.3289660116
H -13.2255835107 1.5944740262 -4.4571781658
H -13.999126228 0.5962663783 -5.7146689181
H -12.6847814404 -1.3385716484 -5.0521898181
H -11.8819568897 0.5545826018 -7.0195674395
H -12.8800407903 -0.5298116666 -3.4813568093
H -12.0051529429 2.2507410061 -6.5213942508
H -10.5242363422 1.9048169804 -4.6302001176
H -9.7701372022 0.8684933093 -5.8612935576
H -11.3262243991 -1.9197649713 -0.6939153736
H -9.6042410409 -2.3062972214 -0.7733228795
H -8.7551907352 -1.1157661918 -5.4326479905
H -7.5371447663 -1.8869726781 -4.4052404854
H -6.1018780143 -0.4679628151 -5.7383991659
H -7.3440314965 0.7901272847 -5.8618977727
H -5.727221331 0.0625539208 -3.3633405639
H -5.8998060282 1.6749987743 -4.077011914
H -7.5449485409 0.9186302557 -2.0170262895
H -8.2037669537 1.8632452755 -3.3520576922
H -10.4990846176 -4.5505274378 -0.5719356366
H -9.203919877 -4.4720312266 -2.6719865993
H -10.7066676234 -5.2605415344 -3.1862581795
H -11.9833664851 -4.1061449539 -1.4272444105
H -9.8879593691 -2.9790070847 -4.4497031761
H -11.6020949754 -3.1828566547 -4.0659027474
H -15.4961526044 2.5196517662 -0.9253009961
H -14.0296743946 6.0154873089 0.1214941807
H -0.6288491364 1.5135091646 -0.3269620967
H 0.6049469305 5.0606068015 1.274428816
H -1.4471926583 -0.0522399654 -0.5025213611
H 0.5113800284 -2.1606244519 -0.0996251483
H 0.4895003537 -0.4638000069 -1.895198197
H 1.5017848719 0.6221590585 -0.9296935497
H 2.1908286341 -1.5929821307 -0.1065696777
H 0.7650845872 -1.4699092007 2.2074146197
H 1.7876471046 -0.0934620361 1.7628441283
H 1.8343110714 2.4913590042 1.595697666
H 2.5617484595 4.5309972374 2.6886584645
H 1.5155961796 4.2344002688 4.0871364522
H 0.2118192778 5.88067901 2.7962963745

H -0.5518608926 2.245243675 4.9124776799
H -1.2441816866 4.0718148842 3.5047107205
H 0.4017718727 0.9529689315 5.6572321065
H -1.6878820228 0.8914255604 6.9707568721
H -2.6565605623 1.1231461748 5.4964220639
H -2.6874968801 -1.3223288918 5.7285999329
H -1.0040536076 -1.3413948558 6.265057125
H -2.0555349798 -1.0730856117 3.4386257941
H -0.514012049 -1.7441822699 3.9937847624
H -0.9706124236 -3.5565039414 2.4638644122
H -1.1719618831 -4.537521035 1.0043864043
H -2.7591821001 -2.9799316643 0.054860418
H -3.4267886566 -5.2356621067 1.719164931
H -1.7502780822 -1.8419185435 0.9737517226
H -2.513843539 -5.3112213795 3.2361611367
H -3.3225231958 -3.1378458524 3.9418699945
H -4.7584490013 -3.7498077952 3.1116421184
H -5.8652012846 -0.8059979353 -1.2730942223
H -7.3755478387 -1.0725234599 -0.3754644354
H -7.6271393921 -2.0070982279 2.3133376746
H -7.4836062582 -0.2670396717 2.0047828569
H -8.5489222024 -0.2043147237 4.1546385383
H -7.7166955171 -1.6734520277 4.7009997386
H -6.4676119489 1.09604339 4.2755017265
H -6.368923542 0.1086707225 5.7447800985
H -4.3531308168 0.0388032826 3.8825246425
H -4.8520179145 -1.4579494882 4.6942860781
H -7.7360105455 -2.5207495624 -2.2651370506
H -8.0810434371 -3.8041796602 -0.2251947986
H -6.9865233124 -4.8782371101 -1.1010838134
H -6.0221599446 -2.9839501065 -2.339292869
H -6.4087264001 -3.8800822006 1.4349232019
H -5.1184017033 -4.2439144749 0.2758990548
H -1.5423583753 4.0495991935 1.7600196041
H 1.7282579219 1.9777005496 3.29184
H -3.3413261092 1.9717047752 -0.026818916

IIIaa
C -0.0083589708 0.4510878861 -0.5230775473
C 0.0624388134 0.5853795913 0.8670421972
C 1.3217811811 0.5762521304 1.5043686889
C 2.4944884083 0.4149830197 0.758990913
C 2.3921209387 0.2421130817 -0.6235360949
C 1.1437489889 0.2404495363 -1.2813531588
S 1.1802574481 0.7467475465 3.2647491473
C -0.5890694142 0.7913511374 3.1482828747
C -1.0355316263 0.6833052107 1.8111690609
C -1.5048495294 0.9173527603 4.1965216171
C -2.8718174686 0.9216271329 3.8997656787
C -3.3405399285 0.7982280391 2.5771829852
C -2.4071395922 0.6714272921 1.5448582585

P -5.142775096 0.7002605635 2.0972137352
N -5.2560603742 1.9600258754 0.9733763007
P -4.814360779 3.465163719 0.9117328772
N -3.7173858788 4.2052158392 1.9668170389
C -4.1111385205 4.4630190525 3.3541569826
P 0.992829441 0.0105306314 -3.1330357511
N -0.4263152717 -0.8961726564 -3.2484157245
P -0.9800577248 -2.2342979605 -2.643468155
N -0.1419871141 -3.2324278572 -1.5594556879
C 1.01620669 -3.9898261301 -2.0488435047
N 2.3034793098 -1.0824942171 -3.3298224729
P 3.4014303135 -1.0005008505 -4.4420786205
N 3.7781532344 0.4501059161 -5.2433002657
C 4.3229798122 1.5406978634 -4.4252841733
N 4.9019400054 -1.3742299697 -3.7309891712
C 4.9365191012 -2.2617883798 -2.5699275092
N 3.0400967573 -2.0187334078 -5.7528306061
C 2.0669633821 -3.0944392868 -5.5876570134
C 6.1369275683 -1.3937449475 -4.5084938726
N -2.4012477615 -1.9124427259 -1.7666476429
C -3.2395460244 -0.7831968444 -2.1675279874
N -1.2545595376 -3.3223595375 -3.9290219362
C -1.4974290363 -2.7960204642 -5.270425307
C 3.9796694628 -2.2383755626 -6.8466070644
C -3.1636411849 -2.9628082854 -1.0932415835
C -1.9097399696 -4.6053475578 -3.6926972269
C 0.0273445813 -2.8215073442 -0.1591224101
C 2.8662945617 0.9483679989 -6.2810688823
N -5.83007447 1.2164639846 3.5815240322
P -7.290685714 0.8752407444 4.044385715
N -8.2776847935 -0.1935748327 3.1521835907
C -7.9841586805 -1.6254041344 3.2578563042
N -7.2160350103 0.2288403304 5.6074872976
C -6.0629879253 -0.5467467022 6.0548714564
N -8.2793421916 2.2463791799 4.0855627362
C -7.6748012157 3.5126222777 4.4868669106
C -8.4167263749 0.0073869029 6.4009478287
N -4.0674594821 3.7307153067 -0.5857809322
C -4.2223800803 2.7813784855 -1.6834099503
N -6.1776308863 4.4660376775 1.142741851
C -7.494585939 3.9458668514 0.7886204744
C -9.7352355571 2.2491807749 4.1459499651
C -3.6133872797 5.0488051613 -1.0118725367
C -6.0897123717 5.9179069034 1.0260385926
C -2.2710793035 4.0025010369 1.8283927958
C -8.6799233444 0.188407923 1.7921356398
H -0.9802164456 0.4431783746 -1.0049286872
H -2.7480949985 0.5908185128 0.5159729154
H -3.5966228051 1.0489885141 4.698813852
H 3.2926878642 0.0710729312 -1.2070359892
H 3.4666568703 0.3997375546 1.2460516204

H -1.1645829667 1.0170865619 5.2246212229
H -3.7578283465 -0.3886877886 -1.2853366698
H -2.6236378988 0.0012759951 -2.6081161746
H -4.0059555248 -1.0826654971 -2.9028341292
H 3.4280733957 1.606743394 -6.9572489143
H 2.0296733371 1.5136408447 -5.8462920306
H 2.45906767 0.1176018941 -6.8591446186
H 3.5250606027 2.1091329898 -3.924189386
H 4.8861241013 2.224984256 -5.073440042
H 4.9992937557 1.1432254303 -3.6661653473
H -3.9610623394 -3.3709943905 -1.7356938774
H -3.6341254222 -2.5429000199 -0.1945857827
H -2.5085418012 -3.7802209553 -0.7884612022
H 4.0159112192 -2.1513992603 -1.9969003957
H -0.8052208912 -2.1988491208 0.1663360156
H 5.0531578285 -3.3196507218 -2.8616805923
H 5.7890025642 -1.9891133807 -1.9330400483
H 0.0616209243 -3.7214866533 0.4695233329
H 6.3434398705 -2.3835579966 -4.9481753022
H 6.094497904 -0.6536679035 -5.3098844398
H 6.978678132 -1.1365487007 -3.8513400748
H 1.3584612028 -2.8391366247 -4.8019728931
H 1.910264915 -3.3564587151 -2.1185798242
H 2.5518322168 -4.0522447173 -5.333216174
H 1.2067937744 -4.818241719 -1.3545753679
H 1.5150637488 -3.2309550219 -6.5276088936
H 0.8110878505 -4.4049879804 -3.0366183134
H 4.6211881689 -3.1184217997 -6.6755428961
H 3.4216925931 -2.4051597544 -7.7784312709
H 4.6156762633 -1.3623648712 -6.987665724
H -0.9740155351 -1.8476219749 -5.3919034311
H -1.5625188063 -5.3325424442 -4.4396007833
H -1.1235933716 -3.5140810181 -6.0133030765
H -2.5724305428 -2.6358846662 -5.4605516897
H -3.0071478071 -4.5358431013 -3.7744173796
H -1.6563000163 -4.9875042187 -2.7020015029
H 0.9548864314 -2.2527666089 -0.0143592142
H -3.2516628697 2.6166166572 -2.1719017007
H -4.5933841997 1.8345189953 -1.2931470213
H -4.9294629467 3.1566673128 -2.4414903976
H -9.6127108627 -0.3332717849 1.5376466718
H -7.9126621826 -0.0694014209 1.0488698249
H -8.8620635942 1.2629056538 1.7392539968
H -7.1350404007 -1.921108428 2.6236344171
H -8.8710449892 -2.1914895398 2.9446365153
H -7.7607923782 -1.8961225223 4.2917223647
H -4.3493740368 5.548230834 -1.6630101597
H -2.6764499692 4.9475745098 -1.5770534656
H -3.4176195132 5.6884139327 -0.1496764225
H -5.2212205519 -0.3742036624 5.3848801742
H -2.0241249775 3.6417224201 0.8300598494

H -5.7786675667 -0.236032679 7.0698941332
H -6.2833153782 -1.6272315193 6.078836748
H -1.7559996267 4.9589097471 1.9970915041
H -8.187071151 0.1629154838 7.463660444
H -9.2049310262 0.7075398539 6.1239777265
H -8.8099181624 -1.0169555195 6.285080305
H -6.5930487082 3.4482223627 4.380471114
H -3.9816074663 3.5725765317 3.9844863548
H -7.9240141408 3.7621684532 5.5311921682
H -3.4866054413 5.2738115213 3.7515063785
H -8.0405766279 4.3214773626 3.8402520133
H -5.1547374239 4.7744552852 3.3990367511
H -10.0985557435 2.5609133781 5.1391772732
H -10.1351836071 2.9611451454 3.4086755583
H -10.1326829046 1.2583051172 3.9216809667
H -7.5009381455 2.8642648063 0.9100795687
H -6.8621533524 6.3788105427 1.6572720242
H -8.2513950719 4.3859591467 1.4524109473
H -7.7687981066 4.1910051162 -0.2520926858
H -6.2482083808 6.2662395936 -0.0083575338
H -5.1157700614 6.2719190665 1.3692276454
H -1.9014094491 3.2731290579 2.5571936668

IIIaaH^+
C 0.2169879232 0.251357466 -0.0022485599
C 0.2142559433 0.5532184818 1.3580788781
C 1.4347265542 0.8562517907 2.0071084238
C 2.6460042842 0.8249032869 1.3026564774
C 2.6357005822 0.4717941312 -0.0469274995
C 1.4217793169 0.1780303397 -0.7036173525
C -0.9137814578 0.5343180658 2.2627337806
C -0.5426412377 0.8717371271 3.5811588421
S 1.1990678118 1.1949245748 3.7206899142
C -1.493210039 0.8867681445 4.6080408127
C -2.8109243952 0.5422139823 4.3009504252
C -3.1993243772 0.1818898164 2.9939179943
C -2.2394225703 0.1777761652 1.9863780175
P -4.9348998404 -0.3177151344 2.5070115943
N -5.5691333123 -0.5115937251 4.0529631262
P -7.0420850494 -0.7664885777 4.5153591971
N -7.9421442133 0.6742991363 4.5667514839
C -9.3470462916 0.6991228659 4.9768312247
P 1.3308890722 -0.3163933576 -2.4384995991
N 2.8112948996 -0.7822339859 -2.8845646966
P 3.3695166025 -1.0681043412 -4.351788186
N 2.7681129943 -2.5076900904 -4.9661277782
C 2.8175632271 -2.8978007606 -6.3779422893
N 0.0987216417 -1.3062569599 -2.7115119885
P -0.7731297704 -2.4651303716 -2.0726142949
N -0.9495622773 -3.6319405995 -3.2770020882
C -1.8464200036 -4.7697138974 -3.0464652881

N -0.2376070071 -3.3676666495 -0.7683291112
C -0.3153400799 -2.8829210537 0.6187936523
N -2.2130732701 -1.8037053704 -1.5044312375
C -3.2447212869 -2.6323423649 -0.8589536275
C 0.8445997204 -4.3423099581 -0.9519332774
N -5.5501533544 1.1740625649 1.9710455974
P -5.5547945894 2.1117552896 0.737280546
N -6.9694298384 3.0330737144 0.779940699
C -7.2414678198 4.1154852655 -0.1603039754
N -4.3710609779 3.3245490094 0.5758123464
C -3.0094757704 2.9651599733 0.1666517942
N -5.3796946768 1.2941032597 -0.7695211164
C -5.3991277685 2.0588398716 -2.0213830227
C -4.3561732855 4.3489853956 1.6291951155
N -8.0929901688 -1.770946981 3.6300739721
C -8.6093952711 -1.2877972873 2.3444982349
N -6.9716646155 -1.5616413492 6.004113128
C -8.1881322103 -1.8267633991 6.7677954961
C -7.7550428002 -3.1984828466 3.5772950485
C -5.7574849499 -1.5185016369 6.8172503037
C -7.2233716356 1.9035154568 4.9203171387
C -6.1676419882 0.0604294976 -0.884034532
C -8.1462180809 2.5627562977 1.5036053057
N 3.0017153085 0.0236778595 -5.5803098119
C 1.6239866907 0.1532399705 -6.0752873373
N 5.0291615149 -1.1146642705 -4.2006619077
C 5.8651716339 -1.4955038249 -5.3397369541
C 3.7541534163 1.2807201199 -5.6608677842
C 5.7618879478 -0.4784586099 -3.1040832074
C 2.7073235708 -3.6571832656 -4.0568559295
C -2.7834713143 -0.6236795831 -2.1674807292
C -0.8221361837 -3.2686436683 -4.6904545031
H -2.522525353 -0.1000635795 0.9719448034
H -0.72282473 0.020789833 -0.4890966657
H 3.5701941379 0.4062027901 -0.5940838718
H -3.5668014406 0.5337287813 5.081587035
H -1.2125809517 1.1478274952 5.6251197664
H 3.58487015 1.0475021 1.8015932368
H -5.8068474112 -0.5112647334 -1.7480403059
H -6.0430116089 -0.5540159207 0.009493233
H -7.2410346337 0.2620123949 -1.0387249139
H -9.5210381562 -1.8456929293 2.0962123416
H -7.881600817 -1.4315037184 1.5306857087
H -8.8541342726 -0.2278088331 2.4014952753
H -6.9770327663 -3.4063955111 2.8256012468
H -8.655650887 -3.7662754002 3.3131279301
H -7.3990958405 -3.5395499966 4.5506675626
H -6.4230843549 2.2994920071 -2.3482947741
H -4.9263001343 1.4613390204 -2.8124694903
H -4.8420535117 2.9906651005 -1.9135480085
H -4.8891681715 -1.389169766 6.1713358426

H	-3.0244093208	2.1358091216	-0.5414829903
H	-5.7853097172	-0.6980884866	7.5526860107
H	-5.6587952154	-2.4652508493	7.3647548872
H	-2.543072822	3.8320909182	-0.3193835403
H	-8.4230355532	-1.0152855152	7.474703672
H	-9.0380533506	-1.9680662451	6.0972782923
H	-8.0565707649	-2.7505594778	7.3459597171
H	-6.243395538	1.9099204123	4.4447244715
H	-3.8416899233	3.9931801922	2.535072799
H	-7.1097558017	2.0166259809	6.0110494144
H	-3.8304425992	5.2351525884	1.2534174456
H	-7.7934261537	2.7627090051	4.5467305986
H	-5.3743357819	4.6357335011	1.8974379195
H	-9.4615389637	0.8421021161	6.0626139373
H	-9.8526487863	1.5330130282	4.4720458915
H	-9.848062667	-0.227180221	4.6910606221
H	-7.8528381379	1.8102092158	2.2345609359
H	-7.7055228135	4.9572171762	0.3727317921
H	-8.6126334925	3.4062831647	2.031885394
H	-8.8967716592	2.1342450262	0.8195677396
H	-7.9324243835	3.8010036467	-0.9580895901
H	-6.3160615678	4.4714986854	-0.6153886834
H	-2.3894119645	2.6735064675	1.0252857477
H	-3.3674096612	-0.0522427397	-1.4380450187
H	-1.9909877203	0.0094701123	-2.5679054894
H	-3.4524570708	-0.9100170262	-2.9939391772
H	1.641508473	0.3393157656	-7.156125365
H	1.1013582403	0.9893448812	-5.5897115692
H	1.0586436174	-0.7608795992	-5.8889092663
H	3.3034527778	2.0678016613	-5.0380452113
H	3.7617865399	1.6242999513	-6.7015395747
H	4.7873325275	1.1347909664	-5.3426177264
H	-3.9957407739	-2.9726737541	-1.5866479522
H	-3.7518995259	-2.0421489162	-0.0863627929
H	-2.7995830552	-3.5057394448	-0.3817091727
H	5.0875451058	-0.2872467901	-2.2708324026
H	-1.1120795246	-2.152556819	0.7409375161
H	6.5589322175	-1.1515321388	-2.7661445238
H	6.2204732407	0.468748615	-3.4258132224
H	-0.5227497413	-3.7350453031	1.2766364394
H	6.6424448597	-2.1944022032	-5.0080827288
H	5.2750491521	-1.984366986	-6.1153397399
H	6.357193078	-0.6192398016	-5.7869201779
H	2.6441184888	-3.3162057921	-3.0245122718
H	1.8267298838	-3.889207155	-0.7519812228
H	3.593083667	-4.2995619141	-4.1685441488
H	0.69894495	-5.1700089927	-0.2481863708
H	1.8126089577	-4.2478063886	-4.2803134437
H	0.8348186751	-4.7406864661	-1.9661924761
H	3.6289380408	-3.6162238223	-6.5652083278
H	1.8697920101	-3.3785650422	-6.651657716

H 2.9635098101 -2.0274921856 -7.0181428915
H -0.0409605966 -2.5190694347 -4.8091373397
H -1.4898602972 -5.6291590303 -3.6263463551
H -0.542818418 -4.1649900208 -5.2576221076
H -1.7646371173 -2.8815767688 -5.1075238644
H -2.879098439 -4.5507281481 -3.3559369267
H -1.8465429578 -5.0493387842 -1.9914664198
H 0.6292878512 -2.4187031673 0.9284155507
H 0.9876392638 0.8511339258 -3.1542927385

IIIbb
C -0.1546962715 -0.043405386 -0.1622182743
N -0.0405889055 0.0692903931 1.3002817725
C 1.3797729412 0.3495974175 1.5574182109
C 1.8358299144 1.2809561973 0.4060238954
C 0.8135705513 1.0189420965 -0.7369055781
P -1.2663186487 0.8400452128 2.1931506159
N -0.9208960485 0.2797660925 3.7490356567
C -1.788896312 0.7802366913 4.8521800722
C -2.0689257777 -0.4515463467 5.7324100604
C -0.8911871776 -1.3874710884 5.4242269391
C -0.6925066439 -1.1744909099 3.922163279
N -2.6877636921 0.5062279066 1.6840175806
P -3.8052062972 0.8476509229 0.4468743454
N -4.6304469203 -0.5912032138 0.1200900797
P -5.0219075082 -1.8077340544 1.0483535597
N -3.6842254691 -2.6705608353 1.5957291469
C -2.5304634973 -2.8420012472 0.6872827678
C -1.6968333117 -3.9647030208 1.3319640948
C -2.7287398593 -4.7687137502 2.1404081563
C -3.6631189653 -3.6738558634 2.6716758555
C -5.0632593907 1.7916780703 1.4757112647
C -6.4129115453 1.7485567475 1.1230004697
C -7.3704132465 2.4621606667 1.8557119864
C -6.9593123135 3.270046962 2.9391354697
C -5.6078574925 3.3594213322 3.2856333857
C -4.6774257986 2.6170321219 2.5552817159
S -8.3297159651 4.0495190145 3.7518870014
C -9.4667844116 3.2912453109 2.6226059433
C -8.8104590905 2.4782754557 1.6749108829
C -9.5555410043 1.8392868289 0.6793218365
C -10.9364451772 2.0175608188 0.5805735755
C -11.5712392191 2.8127889825 1.5594743873
C -10.8559194312 3.4528275382 2.5733911381
P -11.8879682896 1.1116350428 -0.778629257
N -12.7235016276 2.3226661508 -1.63393539
P -12.7412687794 3.888848568 -1.6839565447
N -11.2891960918 4.7128296599 -1.5008846501
C -11.0045165943 5.734418791 -0.4722824375
C -9.4891929892 5.6277985095 -0.2499301299
C -8.9604544353 5.1550625236 -1.6107886727

C	-10.0363074192	4.1670001896	-2.0795706611
N	-10.5171855273	0.7740122418	-1.7228319389
P	-10.1739248483	-0.2548101825	-2.8387799375
N	-10.9275126053	-0.1184284666	-4.3553097742
C	-12.4096232012	-0.2477680325	-4.4030726758
C	-12.8902494771	1.037112563	-5.087811197
C	-11.7326827128	1.3514560476	-6.0460035788
C	-10.5036256802	1.0375510626	-5.1846691477
N	-10.4946526344	-1.8565050908	-2.4698758355
C	-10.4289760636	-2.3407347781	-1.0762603203
C	-11.0207426719	-3.7524383455	-1.1701459855
C	-10.5625116659	-4.2278909622	-2.5581880196
C	-10.6929525873	-2.9642207831	-3.4255796615
N	-8.5383128708	-0.0748467273	-3.1754936702
C	-7.7633425985	1.1492989075	-2.9130366177
C	-6.3173590796	0.6783826475	-3.0897462452
C	-6.4233211765	-0.3173995566	-4.2604072813
C	-7.8184947324	-0.968372641	-4.0977589604
N	-13.365624959	4.4751059101	-3.1510635641
C	-12.5294089589	4.7014918664	-4.3456658446
C	-13.5353181572	4.7708342367	-5.5197809682
C	-14.9072678325	4.9497943371	-4.8456494197
C	-14.7428201274	4.1534581754	-3.5473748574
N	-13.8091563958	4.539103265	-0.5459715119
C	-14.2490409658	5.952679012	-0.5659850199
C	-15.7025977593	5.9140679776	-0.0642575343
C	-15.7197577546	4.668859146	0.8352447442
C	-14.8569664066	3.6770824765	0.0488856189
N	-6.0162984379	-2.7971115962	0.1227800401
C	-6.3359621976	-2.5687549855	-1.3002830611
C	-6.6077259102	-3.9819058044	-1.8380087666
C	-7.1720402536	-4.7206816651	-0.614990359
C	-6.3054833504	-4.179533857	0.5296876543
N	-5.9031980734	-1.6587771899	2.4756402323
C	-5.2923459217	-1.0602640209	3.6879634555
C	-6.4075098996	-0.1999422216	4.2970013132
C	-7.6923785017	-0.912685213	3.847101219
C	-7.3565015437	-1.371594024	2.4215763456
N	-0.9942415415	2.5009078481	2.3928657343
C	0.0540426523	3.1468239292	3.1867760914
C	-0.497746186	4.5754191188	3.3827193703
C	-1.4218261283	4.811344338	2.1518325203
C	-1.3892557104	3.4775419845	1.3727642467
H	-6.7281879608	1.1181502031	0.2953406234
H	-9.0594818635	1.2227616699	-0.0616945539
H	-12.6451097348	2.9616182464	1.5201701947
H	-3.6335782422	2.6536267957	2.850669945
H	-5.2862439519	3.9725742352	4.1244986571
H	-11.3712351778	4.0776941909	3.2989058946
H	-15.4444268039	4.4639403651	-2.7666384256
H	-16.7262019056	4.2821558146	1.0273778

H	-14.8881090775	3.074766689	-3.7205063906
H	-13.5235219826	3.8267943244	-6.0761228926
H	-15.7374369662	4.5900365614	-5.4631635342
H	-15.0870241252	6.0061357841	-4.6096046126
H	-13.2945298034	5.5729748388	-6.22524489
H	-11.8047566463	3.8925849019	-4.5085130732
H	-11.9610692258	5.6315015936	-4.2229938523
H	-14.1567022386	6.3771487123	-1.5723257007
H	-16.3931939234	5.7748538042	-0.9061193062
H	-15.9898414687	6.8348875662	0.4546409188
H	-15.2501364529	4.8938348202	1.8019583294
H	-11.5630914087	5.5309822322	0.445524458
H	-14.421503486	2.8941993799	0.6738077351
H	-11.2847024742	6.7357254624	-0.8346901091
H	-9.0510317428	6.5755526482	0.0812577392
H	-9.2827961394	4.8727389731	0.5128340965
H	-7.9742569424	4.6831911203	-1.546795787
H	-8.8898848901	6.0009091741	-2.3078778221
H	-9.8444816833	3.1543949685	-1.7162152495
H	-10.0919825448	4.1167514932	-3.1729195991
H	-12.9870800403	1.8211458992	-4.3298624407
H	-13.8555520391	0.9127026765	-5.5914474568
H	-12.6808735626	-1.1352570987	-4.9922316315
H	-11.7673478696	0.6866533276	-6.9192877333
H	-12.8331461366	-0.3621512504	-3.3994341
H	-11.7254760629	2.3842226546	-6.4100580966
H	-10.2563297581	1.907656304	-4.5588377098
H	-9.6167268891	0.7840110573	-5.7767604585
H	-10.9979120794	-1.6847879041	-0.4124601414
H	-9.3898832467	-2.371064876	-0.7140570759
H	-8.3353587397	-1.0638954254	-5.0640491006
H	-7.7511930072	-1.9735559866	-3.6667189274
H	-5.6202777643	-1.0620874581	-4.259641101
H	-6.3739172963	0.2197202712	-5.2159008248
H	-5.9675952699	0.183316009	-2.1781742923
H	-5.6321543101	1.5063992316	-3.2999238889
H	-7.9803971515	1.5305256129	-1.9153042042
H	-8.0080169981	1.9471545912	-3.6352113256
H	-10.6857188179	-4.4034567532	-0.3553823293
H	-9.515477419	-4.5501254627	-2.5156416931
H	-11.1537491636	-5.0622953553	-2.9506042149
H	-12.1159690027	-3.6974529075	-1.1309571744
H	-9.9624925592	-2.9368533452	-4.2410194561
H	-11.6918210717	-2.9058066841	-3.8783167408
H	-15.4523435276	3.1701308488	-0.7244372624
H	-13.6249229223	6.5538800836	0.1088061218
H	-1.1876011511	0.0864568392	-0.4903214865
H	-1.0832629313	5.6446660228	1.5272130331
H	0.1683070441	-1.053610341	-0.4548858368
H	2.8684428365	1.0722477045	0.1051604872
H	1.2933529151	0.6695335709	-1.6576164421

H 0.2640401848 1.9337848264 -0.9833496936
H 1.7953026995 2.3282351341 0.7242315377
H 1.9328825915 -0.6012261075 1.5188219926
H 1.5218506508 0.7730514205 2.5544825834
H 1.0080990907 3.1856282375 2.6328868191
H 0.3092718432 5.3126052444 3.454436045
H -1.0761559772 4.6263904521 4.3113586826
H -2.4449167848 5.0333153254 2.4714439586
H -2.7139619452 1.2190205404 4.4645667193
H -2.3464873779 3.2127615374 0.9179950988
H -1.2553532913 1.5584460098 5.4139308831
H -2.1575163732 -0.1933995569 6.7929308843
H -3.0064909036 -0.925750098 5.4219213214
H -1.0934539898 -2.4353292657 5.6717916861
H 0.0060799989 -1.0733073091 5.973183296
H -1.4281750427 -1.7587374618 3.3533524634
H 0.3067553325 -1.4493850399 3.5727902684
H -0.9445443299 -3.5354253508 2.0043306922
H -1.1677927745 -4.5670809598 0.5857161787
H -2.8690733177 -3.1302538155 -0.3189838127
H -3.2834512049 -5.4471974363 1.4791632533
H -1.9754227813 -1.9068234259 0.6019169098
H -2.282193447 -5.367829121 2.9420940212
H -3.248437672 -3.2382553321 3.5947079445
H -4.6690640664 -4.0327478433 2.9058554538
H -5.5098590446 -2.0681435362 -1.8094386808
H -7.2205472169 -1.9261482482 -1.3967250333
H -7.9150237889 -2.2649083946 2.1213897973
H -7.5848032506 -0.5773589967 1.7009646471
H -8.5731364131 -0.2628702882 3.8678406638
H -7.89247696 -1.7827458873 4.4866516614
H -6.3717954642 0.807085075 3.8771393391
H -6.3198999307 -0.1162215747 5.3860923912
H -4.3996352942 -0.480303995 3.4335707589
H -4.9962024588 -1.8610752316 4.3814998901
H -7.2866894176 -3.9819185265 -2.6964527947
H -8.219081196 -4.4391038874 -0.4543850812
H -7.1234224992 -5.8117426977 -0.6989556433
H -5.666685019 -4.4503403452 -2.1555318585
H -6.8158824178 -4.2106308024 1.5005924986
H -5.379786089 -4.769229709 0.6229834799
H -0.6376412481 3.523266846 0.5652568106
H 0.2299065129 2.6075975065 4.1191499741

IIIbbH^+
C 0.4375365608 -0.9737630738 0.0277918946
N 0.2318339296 -0.5088969877 1.4282384154
C 1.5292348398 -0.3445192605 2.1201835014
C 2.5224914041 -0.1905810136 0.964455818
C 1.9594404365 -1.1635896522 -0.0820984052
P -1.206766396 -0.7433118243 2.2481645187

N -2.3109487224 -1.1089672836 1.1881204796
P -3.5330243281 -0.7775825665 0.2177503797
C -3.4954337879 0.9618463678 -0.3189301884
C -4.6760195809 1.533768183 -0.7998471926
C -4.7210093925 2.8961952602 -1.1063800206
C -3.56445115 3.6918211015 -0.9163072908
C -2.3604294456 3.1157887303 -0.4954255415
C -2.3339645581 1.7528438483 -0.205288226
S -3.8780506429 5.4007081851 -1.2172296653
C -5.577594898 5.0539983992 -1.5961975615
C -5.8674078586 3.6770077404 -1.5239334557
C -7.1517647475 3.2187341171 -1.8335296024
C -8.1624387473 4.1030993942 -2.2087670557
C -7.8564144195 5.483194485 -2.2251800318
C -6.5791064159 5.9683935901 -1.9401086126
P -9.9013755775 3.4281016239 -2.5553995841
N -9.4038109774 1.8077207668 -2.6729284938
P -10.1449346708 0.4561041237 -2.9062263533
N -8.9379338615 -0.7216634286 -2.993024311
C -7.6174857123 -0.4923398255 -3.5949144167
C -6.8313045246 -1.7275089178 -3.1454529335
C -7.8807212522 -2.8587074758 -3.2152701854
C -9.2507244554 -2.1589904252 -3.0007672504
N -1.1933004096 -1.9882050022 3.3791616369
C -1.1978145488 -3.3955003838 2.9305833669
C 0.2404385723 -3.8674057075 3.1835232695
C 0.6170666879 -3.1582184582 4.5050197132
C -0.3487309153 -1.9396647451 4.5961733121
N -1.3408060187 0.5916890596 3.2319258315
C -0.7961729933 1.9389363515 2.9521651121
C -1.9531501085 2.8774955208 3.301207343
C -2.5789583563 2.1834782339 4.5209817202
C -2.479416925 0.6830549899 4.1853865181
N -5.0248932175 -1.1765131094 0.6634412929
P -6.0175439561 -0.8341263918 1.8634850567
N -6.5481840353 0.7279703706 2.0911585361
C -5.6718983205 1.8203747489 2.5768365612
C -6.3561837353 3.1135998451 2.0956063026
C -7.8235730249 2.7081749227 1.880897474
C -7.7245708749 1.269059647 1.3664884347
N -5.3374001097 -1.2281203665 3.3382284647
C -4.6731307158 -2.5540320914 3.448115686
C -4.4184687186 -2.7018646272 4.949785396
C -5.64171026 -2.0105199079 5.5719289368
C -5.8473762718 -0.7898682298 4.6627670531
N -7.3794713212 -1.7378278668 1.5882649103
C -7.7591299759 -2.3902548054 0.3180964028
C -8.7968399417 -3.4267645344 0.7651154299
C -9.5070392198 -2.7279627955 1.9351698453
C -8.3704501942 -1.9831479141 2.6548708987
N -10.2729200278 3.9015909147 -4.1459013801

P -9.5764572196 4.7712631271 -5.2503216988
N -9.7028727758 6.4257511755 -4.9286414919
C -9.384396914 7.474005801 -5.9282479068
C -10.3905107603 8.6021831354 -5.6447987385
C -10.6882895259 8.4271948559 -4.148117543
C -10.7531893941 6.9049023255 -3.9950015516
N -7.9525164741 4.4455321685 -5.521723728
C -6.8625444687 5.4317896849 -5.6264686616
C -5.5999414628 4.6127555429 -5.320914344
C -5.9561439152 3.2108552646 -5.8348806199
C -7.4335290283 3.0619602853 -5.4472046158
N -10.3006654763 4.6115143297 -6.7654495089
C -9.8794555465 3.6335210926 -7.7875890436
C -11.0456810542 3.610219742 -8.7993232857
C -11.798045279 4.926978303 -8.5429946795
C -11.6645163923 5.0963008585 -7.0257706106
N -11.0971457518 0.2127698603 -4.2789735037
C -12.3262827163 1.0345129078 -4.465373613
C -12.2640979021 1.4891595617 -5.9281073666
C -11.4921234463 0.3517404023 -6.6108030754
C -10.405954089 0.0361696249 -5.5774055545
N -11.2158557018 0.0137011411 -1.6986400129
C -10.9393584206 0.3725337163 -0.2952944049
C -12.1630035989 -0.1644827817 0.4585786432
C -12.5380643105 -1.41790378 -0.3485560874
C -12.3199763948 -0.9645767306 -1.7991599846
H -5.5736573558 0.9310230959 -0.8941059493
H -7.3808497085 2.1583639981 -1.8200315488
H -8.620338304 6.2016336609 -2.4999443878
H -1.4093256838 1.3032708748 0.1373188147
H -1.4661127244 3.7207177478 -0.3723199581
H -6.3711973467 7.0338039185 -1.9930044888
H -11.76792157 6.1372518038 -6.7049739992
H -11.6139556135 8.915652106 -3.8273645621
H -12.4240046208 4.5051492878 -6.4908685322
H -11.7075100099 2.7629459484 -8.5851492921
H -12.8407078255 4.8967977541 -8.8753520104
H -11.2997412576 5.7601817316 -9.0541046451
H -10.6960993633 3.5059010068 -9.8313225843
H -9.7120500827 2.634873932 -7.3644174764
H -8.936936395 3.9586211668 -8.246208271
H -9.4613641955 7.0804587223 -6.9484001514
H -11.3081645255 8.4522602506 -6.2274286502
H -9.9918005249 9.5893056953 -5.90005291
H -9.8635109338 8.8332244105 -3.5475089945
H -7.0024680978 6.2430906106 -4.9064767146
H -10.5641426702 6.5653414021 -2.9730212653
H -6.8197749747 5.8693483596 -6.6357545736
H -4.7059890066 5.0353039336 -5.7915243172
H -5.4361629295 4.5833557011 -4.2389367348
H -5.3357272309 2.4205501952 -5.3985514915

H	-5.8438493738	3.1668274856	-6.9259214738
H	-7.5509442149	2.6534360623	-4.4388173478
H	-7.9672856299	2.3950273059	-6.1329644551
H	-11.6889927042	2.4172393913	-5.9769404192
H	-13.2565101994	1.6642719026	-6.3568996246
H	-13.2115530843	0.4127889888	-4.2782489429
H	-12.1404237078	-0.52126258	-6.7595720336
H	-12.3493046143	1.8872037376	-3.7789091773
H	-11.0694587016	0.6277057762	-7.5825901386
H	-9.5691375094	0.7417056904	-5.6858577756
H	-10.0083266054	-0.9810095257	-5.668063499
H	-10.8041590089	1.4529243425	-0.1885109846
H	-10.0194389357	-0.1192867929	0.0560956645
H	-9.9589753408	-2.4254463416	-3.7997345175
H	-9.7187287111	-2.4386069839	-2.0498194883
H	-7.700884582	-3.6430972238	-2.472607922
H	-7.8563946008	-3.3360912702	-4.2009976565
H	-6.4760558163	-1.5838566593	-2.1193249701
H	-5.960291474	-1.9236172904	-3.7800207835
H	-7.2026580353	0.4526230758	-3.2420306229
H	-7.6674041905	-0.4462247148	-4.6961321832
H	-11.9517928109	-0.3684097776	1.5140732781
H	-11.8550956505	-2.2432064509	-0.1066675263
H	-13.5606098469	-1.7644909382	-0.1678475921
H	-12.978548279	0.5677883148	0.4164081577
H	-12.0722897365	-1.789011482	-2.4738031246
H	-13.2243251212	-0.4788648365	-2.1889170245
H	-11.7462625921	6.5264383281	-4.2759619448
H	-8.3561906057	7.8315990733	-5.7866150206
H	-1.5261990235	-3.4745313347	1.8929065612
H	2.2305735148	-2.1930293061	0.1838884982
H	-1.906141448	-3.9602483618	3.553805168
H	0.4648096467	-3.8243160879	5.3606884826
H	0.3272894105	-4.9568765676	3.2443652718
H	0.8826474055	-3.5247881783	2.3628431363
H	1.6673587645	-2.8501426546	4.5221495749
H	-0.997638526	-2.0295398457	5.4769163293
H	0.1674845672	-0.9803893011	4.669621644
H	1.7772215634	-1.232773483	2.7192063612
H	3.5496814898	-0.4191764663	1.264048695
H	2.4985426867	0.8383299961	0.5841509605
H	2.3259834421	-0.9726145514	-1.0950260582
H	-0.4548653332	2.0242346736	1.9200097856
H	0.0788362819	-0.2101672801	-0.6737688975
H	0.0646190451	2.132017517	3.609063834
H	-1.6169842817	3.8976306016	3.5118789016
H	-2.661371518	2.9154138453	2.4659245443
H	-3.6107028489	2.4924424463	4.7114301747
H	-1.993830548	2.4091486849	5.4205718441
H	-3.3919834301	0.3087245974	3.7114924769
H	-2.2908861367	0.0728371501	5.0751962494

H -3.4971292734 -2.1717356486 5.2196150947
H -4.3116306898 -3.7480955568 5.2527530983
H -5.339727825 -3.3509608316 3.084088139
H -6.5138583434 -2.6742982608 5.5196585319
H -3.7624713861 -2.5655918821 2.8518340873
H -5.4992643424 -1.7259931906 6.6191237354
H -5.2507423872 0.0582251956 5.0238566797
H -6.8882498161 -0.4601212589 4.6101400518
H -6.8812680346 -2.8324117088 -0.1566326202
H -8.1964617646 -1.6630030644 -0.378312332
H -8.6149418436 0.6790231299 1.5931067206
H -7.5809325502 1.2515546928 0.2792818368
H -8.3420247971 3.3564744228 1.1686701424
H -8.372517198 2.7277159277 2.8305077758
H -5.9195317567 3.4403326878 1.1496871098
H -6.239257007 3.9287715573 2.8164293024
H -4.6567225083 1.7266066069 2.176740929
H -5.6110832075 1.7848669983 3.6704652533
H -9.4798284467 -3.706921676 -0.0427406535
H -10.2352407248 -2.0066702239 1.5512781466
H -10.0363583966 -3.4175702798 2.5996137211
H -8.2923942875 -4.3367007005 1.1132676731
H -8.7160189337 -1.0464920211 3.1112729697
H -7.92827213 -2.5982883868 3.4512356847
H -0.1160711125 -1.8945518371 -0.1761676886
H 1.5116195151 0.5205846294 2.7892198736
H -3.3206108843 -1.5460856035 -0.9390948334

IVaa
C 0.018227451 0.2547771361 -0.3719425124
C -0.0747093082 0.4891613515 1.0012085634
C 1.089870751 0.768716587 1.7393717025
C 2.3505698737 0.7999629898 1.1593827088
C 2.4218621293 0.5364294415 -0.2146930795
C 1.2749475211 0.2567936966 -0.9878030719
O 0.7997945871 1.0039527895 3.0722434847
C -0.5726847462 0.8773402719 3.188982952
C -1.1629574372 0.5401363313 1.9560694137
C -1.3176930156 1.0773387714 4.3431567518
C -2.7095219638 0.9427885646 4.2319242396
C -3.3328248408 0.6009293181 3.0148993456
C -2.5444569481 0.3726481067 1.8802397175
P -5.1767344354 0.4084549247 2.7960964954
N -5.5498395982 1.6853899041 1.7450941141
P -5.0053111336 2.9290608569 0.9932174932
N -3.880994906 4.0435293245 1.6154511457
C -4.292303271 4.8875913571 2.7394981842
P 1.359410583 0.0087289431 -2.8324122167
N 0.3493561916 -1.33750265 -3.0107592124
P -1.0263824226 -1.9951711247 -3.2094159034
N -1.5976127067 -3.0777684828 -2.0216103875

C	-0.7361143019	-4.2439064171	-1.7825412193
N	2.9370336157	-0.6158941087	-2.9155730069
P	3.7829871796	-0.8922388099	-4.2001548886
N	3.7487538144	0.1561969631	-5.544567975
C	4.1997267922	1.5316383801	-5.2914494104
N	5.4193975836	-0.818990171	-3.7629010383
C	5.8184070283	-1.1556981578	-2.3972162498
N	3.3648594371	-2.3732534209	-4.9299033147
C	2.8532612929	-3.438753554	-4.0615244716
C	6.485659127	-1.0010220576	-4.7418378119
N	-2.3369676462	-0.8913254589	-3.2968407554
C	-2.0762725822	0.3632328643	-4.0077235367
N	-0.9827075616	-3.0227204364	-4.5654696977
C	-0.1351175973	-2.6697459316	-5.6977225176
C	4.1092157271	-2.8852906593	-6.0785243002
C	-3.684687013	-1.4018166631	-3.5624614521
C	-2.0572726864	-3.9490885916	-4.9038475452
C	-2.0971223633	-2.5242441556	-0.7545722149
C	2.5808410779	0.1276346852	-6.434134029
N	-5.6733756005	0.9242647033	4.3475326029
P	-7.1592277052	0.9010011849	4.8478829249
N	-8.2524983408	-0.3040909846	4.3271892375
C	-8.0094889708	-1.6586624061	4.8297360856
N	-7.1099221632	0.7600842493	6.532673108
C	-5.9682545013	0.1602329798	7.2161879187
N	-8.0222158105	2.2911943602	4.4204708829
C	-7.2879271809	3.5523510617	4.3582374722
C	-8.3255920492	0.796691814	7.3323463219
N	-4.2284031317	2.4825352398	-0.4524266201
C	-4.5246271414	1.1979522718	-1.0739078257
N	-6.3338705504	3.9655801526	0.7151708489
C	-7.6802250457	3.3994459908	0.7119505226
C	-9.4654186595	2.4650072353	4.5275381824
C	-3.5620840395	3.4130140836	-1.3573947909
C	-6.1812060247	5.100421596	-0.1892355479
C	-2.4544202243	3.706675688	1.6919364489
C	-8.6847257049	-0.31342623	2.9224101884
H	-0.8794687037	0.058477666	-0.9512395951
H	-3.005822318	0.1050128366	0.9336182416
H	-3.3401850648	1.1240813631	5.0975948073
H	3.3900576876	0.5313402501	-0.7061114307
H	3.2379656074	1.0122664455	1.7485460031
H	-0.8414667637	1.3437303442	5.2824242757
H	-2.8240446869	1.1061357416	-3.7043687931
H	-1.0850531833	0.7505062655	-3.7601998656
H	-2.1416485663	0.2426269774	-5.1031060083
H	2.8544840082	0.6002764351	-7.3864951019
H	1.7249697428	0.6707460129	-6.0056167197
H	2.2752986241	-0.8997492682	-6.6326399006
H	3.4084236045	2.1405363664	-4.8282503917
H	4.4849131122	1.9929274346	-6.2456693288

H 5.0686374735 1.5294055822 -4.6307244068
H -3.8498130753 -1.6046105759 -4.6334608459
H -4.4214268771 -0.6549450948 -3.2447063464
H -3.8642521299 -2.320747324 -3.001426902
H 4.9811345285 -0.9924856263 -1.718913664
H -2.7242795819 -1.6511924478 -0.9365529946
H 6.1437409283 -2.2063565211 -2.3164079683
H 6.6578180953 -0.5141421102 -2.0953846035
H -2.7037202127 -3.2875494242 -0.2528024647
H 6.8170131336 -2.0502461321 -4.809972144
H 6.1592156562 -0.6693293007 -5.7291932085
H 7.3528774697 -0.3943509064 -4.4467005138
H 2.2232181166 -3.0127898147 -3.2814576941
H 0.1440670467 -3.9828723548 -1.1742702691
H 3.6697363449 -4.0213503466 -3.6025180568
H -1.31578087 -5.0075118585 -1.2496525689
H 2.2429076568 -4.1216654264 -4.6658221787
H -0.3908964506 -4.6642422 -2.7291455929
H 4.9784368273 -3.49185959 -5.7755889637
H 3.4482893042 -3.5230450008 -6.6811519253
H 4.4589619257 -2.0649667695 -6.7084483468
H 0.71413465 -2.0796820034 -5.3551486969
H -1.6267716954 -4.8831768389 -5.293876402
H 0.2492246804 -3.5872107907 -6.1656416813
H -0.6862526131 -2.1032468668 -6.4676442649
H -2.7282634237 -3.5412757599 -5.6772142616
H -2.6473110371 -4.1881869596 -4.0181138935
H -1.2792011547 -2.2309313178 -0.0804509791
H -3.5927378589 0.7145446885 -1.3987429366
H -5.0350986619 0.5450914575 -0.3643823702
H -5.1698811434 1.3232678005 -1.9599802865
H -9.6766417882 -0.7818039581 2.8566825478
H -7.9833090849 -0.8703343314 2.2855969149
H -8.7527078087 0.7050048841 2.537478012
H -7.2007449661 -2.1648937483 4.2800006277
H -8.9284034995 -2.2487141426 4.7198435349
H -7.7476161167 -1.6352423707 5.8897188736
H -4.1793351216 3.6267305572 -2.2452696867
H -2.6122960497 2.9790161373 -1.7012519191
H -3.3417893059 4.3544999412 -0.8535381471
H -5.1062290464 0.1553727935 6.5493241238
H -2.1675443711 3.0281968342 0.890569834
H -5.7260198573 0.747800628 8.1126120662
H -6.182077346 -0.8746663882 7.5338455035
H -1.8675736862 4.6303000822 1.5978331706
H -8.1285902616 1.3169745128 8.279584684
H -9.1220026211 1.3312375056 6.8150770708
H -8.6943557528 -0.2154338092 7.5715100233
H -6.2279385638 3.3477414449 4.2194682441
H -4.1368351559 4.3815919421 3.7051117491
H -7.4289585187 4.1462745466 5.27642601

H -3.6902139952 5.805814017 2.7335944322
H -7.639502735 4.1412768656 3.5020029777
H -5.3450173736 5.1577229132 2.6470065931
H -9.7378537016 3.081231776 5.4005454851
H -9.8406409056 2.9773612246 3.6295782866
H -9.9664486146 1.4989998351 4.6072966719
H -7.739680597 2.6044087626 1.4548989857
H -6.9464041158 5.8527270134 0.0429304236
H -8.400081439 4.189486289 0.9668888816
H -7.960323886 2.9881874982 -0.2735235066
H -6.2967753851 4.8161920904 -1.2493270391
H -5.2008931863 5.5630725819 -0.055496077
H -2.2047304818 3.2274514851 2.646914729

IVaaH^+
C -0.1314212811 -0.6286424886 0.044516341
C -0.3482951361 -0.9197948509 1.3870908294
C 0.7341773133 -1.3641693464 2.1810925536
C 2.0211436812 -1.527826913 1.6827537497
C 2.2204161386 -1.2426476989 0.3276538706
C 1.1560864692 -0.8042078357 -0.4862462568
C -1.5064564678 -0.9358810657 2.2536821102
C -1.0314870391 -1.3712054174 3.5014655953
O 0.3354192621 -1.62938513 3.4610068619
C -1.8539828368 -1.5131801869 4.6100002718
C -3.2047424145 -1.2022504326 4.4248502169
C -3.7228276139 -0.7628684806 3.1843868075
C -2.8632502568 -0.6363299044 2.0905916218
P -5.5565157687 -0.4681747703 2.9886338311
N -5.800200997 0.3293192144 4.4553071615
P -7.1122972536 0.5642752352 5.2767685139
N -7.9873457141 1.8925769218 4.6770262801
C -9.1934532825 2.3729751681 5.3517617919
P 1.3535236622 -0.5341145055 -2.2582901786
N 2.9115600928 -0.7543733628 -2.6102999738
P 3.7015318761 -0.4485942341 -3.9615096022
N 3.4212071902 -1.6263227067 -5.1164111618
C 3.6570156096 -1.4718170972 -6.5535990065
N 0.2525284404 -1.3440497378 -3.1119924191
P -0.4957405785 -2.7393195821 -2.9600129491
N -0.3280647029 -3.5261461402 -4.4433648645
C -1.0332991537 -4.7922193544 -4.6713988151
N 0.0049574453 -3.9119934488 -1.8772785206
C -0.3719033455 -3.8977913453 -0.4558851831
N -2.0768518568 -2.4409270246 -2.4850930123
C -3.0459541429 -3.524079554 -2.2910918447
C 1.245322148 -4.658452473 -2.1174345938
N -5.4909585931 0.7436331992 1.8061254834
P -5.8876418306 1.2859325432 0.4210683035
N -7.4493400197 1.9421563633 0.3720271214
C -7.9606898647 2.7375536461 -0.741751687

N	-4.9527783402	2.599442837	-0.1366691387
C	-3.544481551	2.3021040692	-0.4281620925
N	-5.6478092869	0.1553237314	-0.8578375583
C	-5.9614770622	0.5692948608	-2.2290450141
C	-5.0729811209	3.8274880873	0.6662747767
N	-8.3323611086	-0.6152386855	5.3848839443
C	-9.2238097621	-0.8317530105	4.2388497415
N	-6.6626237746	0.7345533266	6.8977543781
C	-7.6545546234	0.9733457842	7.9438089635
C	-7.9637294362	-1.8812535354	6.0327247596
C	-5.3249068663	1.2193239038	7.2401269911
C	-7.2413301732	2.9650515438	4.0119347486
C	-6.1244265594	-1.2029654479	-0.579959804
C	-8.4950642786	1.3636525211	1.2092849058
N	3.3383562432	0.9669216719	-4.7983731975
C	2.0167008186	1.1401946917	-5.416955218
N	5.3121256381	-0.3997180196	-3.5330541579
C	6.3507370775	-0.3640906918	-4.5629808191
C	3.9368921252	2.2336890639	-4.3645570209
C	5.7737824872	-0.007761004	-2.1983953185
C	3.3844836387	-3.0249986011	-4.6793375368
C	-2.7102568948	-1.1347563806	-2.6827480312
C	-0.1385020363	-2.7430680396	-5.6679983331
H	-3.243752392	-0.2835277196	1.1364404333
H	-0.9591134115	-0.3084201841	-0.5822213808
H	3.2035102585	-1.3717447938	-0.1137743509
H	-3.8829169178	-1.2759957994	5.2703944014
H	-1.463988626	-1.8398809225	5.5688840589
H	2.8300515743	-1.8730311948	2.3185438264
H	-5.6597626023	-1.8961650953	-1.2927349459
H	-5.8519666686	-1.5096974473	0.4327517222
H	-7.2183373837	-1.2953888341	-0.6881020849
H	-10.1246873694	-1.3514074893	4.5884067071
H	-8.7472076779	-1.4450920214	3.4589565726
H	-9.5233620559	0.1202669111	3.8001746276
H	-7.4501029369	-2.5605180424	5.334987594
H	-8.8747055296	-2.3749397063	6.392987921
H	-7.3082204789	-1.6941229306	6.8849019088
H	-7.0433480403	0.5584142364	-2.4363123111
H	-5.4824963478	-0.1249935229	-2.930461542
H	-5.5785083198	1.5737629378	-2.42037908
H	-4.6323499705	0.9911696186	6.4298694176
H	-3.4669957775	1.4082079265	-1.0494745569
H	-5.3207069914	2.3067934393	7.4196100407
H	-4.9852282193	0.7208586266	8.1579909638
H	-3.1136005886	3.1483086347	-0.9765208494
H	-7.8082353996	2.0473178971	8.1344706208
H	-8.6111686632	0.5216302948	7.675388725
H	-7.3096059943	0.5115182616	8.8782565048
H	-6.3914619753	2.5507812605	3.4702584268
H	-4.5448989439	3.7395193486	1.6283615903

H	-6.8824016257	3.7205124217	4.730164683
H	-4.6399500868	4.6603484393	0.1003485922
H	-7.9045423032	3.4645756664	3.2941602011
H	-6.121749638	4.0515533289	0.8669048449
H	-8.9651615974	3.121400143	6.1270018792
H	-9.8559066367	2.8438150336	4.6133492151
H	-9.7328970798	1.5443412206	5.8140892488
H	-8.0576955737	0.8893004715	2.0860268076
H	-8.5555735712	3.5748851048	-0.3500509263
H	-9.1660147896	2.1595813387	1.558780577
H	-9.0995021987	0.6230611152	0.6613406839
H	-8.6100611625	2.1458778453	-1.4053254482
H	-7.1380680217	3.1480886484	-1.3288272475
H	-2.9569441796	2.1448873137	0.4907863281
H	-3.3325224491	-0.8934346451	-1.8122717005
H	-1.9518549668	-0.3619404444	-2.8072493079
H	-3.3520942403	-1.137854034	-3.5761947429
H	2.1260130388	1.6791219317	-6.3660215269
H	1.3426759285	1.716638102	-4.7678216122
H	1.5548294582	0.172369705	-5.6170877787
H	3.3421370444	2.7211572456	-3.5775880213
H	3.9942891583	2.9110040086	-5.224215269
H	4.9486696608	2.0756721502	-3.9891804642
H	-3.7122294643	-3.6253855411	-3.1599045247
H	-3.6591964123	-3.3061246505	-1.4085173634
H	-2.5405447174	-4.4758704751	-2.1245488592
H	4.9501812708	-0.0680661115	-1.4875595219
H	-1.24243988	-3.2671686591	-0.2870665264
H	6.5701060751	-0.6901483693	-1.8776544286
H	6.1754268485	1.016544859	-2.199210737
H	-0.6079458469	-4.9216929573	-0.1411074496
H	7.1943439768	-0.9897542786	-4.2486940922
H	5.975939611	-0.7528307322	-5.5106004133
H	6.7231103497	0.6573565659	-4.7314373
H	3.200774859	-3.0773740165	-3.606955989
H	2.101104928	-4.1721233584	-1.6271796952
H	4.3329150162	-3.533573783	-4.9048261018
H	1.1386710719	-5.6678089956	-1.7031399717
H	2.571178714	-3.5479746742	-5.1945179721
H	1.4432049213	-4.739248656	-3.1860068167
H	4.5498428585	-2.0300921344	-6.8704065366
H	2.7961589471	-1.8670346007	-7.1082659362
H	3.7888327293	-0.4216295206	-6.8147228712
H	0.5055851012	-1.8893485232	-5.4621758009
H	-0.4922097887	-5.3684963763	-5.4307105649
H	0.344397156	-3.3800891078	-6.4186648094
H	-1.0922061407	-2.3856675643	-6.0863344741
H	-2.0624607796	-4.6361296527	-5.0281338545
H	-1.0623223281	-5.3847403166	-3.7551175691
H	0.4510252257	-3.5190611666	0.1609850086
H	1.0097603221	0.8163719461	-2.4775949266

IVbb

C 0.4554282669 -0.1689831486 0.6026158872
N 0.1498418589 -0.1437715385 2.0403827118
C 1.4483808083 -0.1242788445 2.7377102499
C 2.4560881036 0.569944764 1.7747624758
C 1.6742302011 0.7586945399 0.4496842183
P -1.2080181906 0.7221439913 2.5844690598
N -1.2557495271 0.2281799902 4.2012978199
C -2.2604827974 0.8865148807 5.0824718893
C -2.7450996354 -0.2209115845 6.0396901231
C -1.6346334864 -1.2805114587 5.9730254326
C -1.2082960102 -1.2189194739 4.5049430962
N -2.4718535593 0.4314507419 1.7485101296
P -3.4089966754 0.8477832572 0.3898314652
N -4.1838833147 -0.5683001874 -0.1129121969
P -4.5992289598 -1.8729202044 0.6697649969
N -3.2902250234 -2.7683330129 1.2144708614
C -2.0440247739 -2.7900877898 0.4297117355
C -1.0799426173 -3.5823953925 1.3177960443
C -2.0079758939 -4.6081161101 1.9896502407
C -3.2865362542 -3.7985591806 2.2677230843
C -4.8115709848 1.7390050264 1.2717264299
C -6.1173740986 1.6377912403 0.7778130163
C -7.1525146041 2.3248429832 1.4181206382
C -6.8683936261 3.1343612714 2.533516632
C -5.5822657655 3.2868370131 3.0324571613
C -4.5646202655 2.5769500836 2.3829903527
O -8.0153490879 3.7114640127 3.0485906886
C -9.0532192381 3.2622074648 2.2502954222
C -8.5878187444 2.4084818861 1.2350658525
C -9.4887895569 1.8759397986 0.3113761461
C -10.8465221562 2.2091434266 0.3913763579
C -11.2757632041 3.0549950562 1.4379525134
C -10.3946243424 3.6000963745 2.379742149
P -12.0731036134 1.4044735649 -0.7893475546
N -13.071965595 2.6715187847 -1.2680583475
P -13.0058810362 4.1897003583 -1.6578030588
N -11.5369841507 5.0029110029 -1.839788307
C -10.9912801091 5.9073468861 -0.7990947481
C -9.5069220918 5.5386762504 -0.7122416267
C -9.1664612892 5.1286761028 -2.1497282289
C -10.4279834927 4.38686447 -2.621255181
N -10.9644571193 1.1686898434 -2.0711675482
P -10.6590297348 -0.0903234951 -2.9419658665
N -11.6225453144 -0.397714644 -4.305071436
C -13.0702314094 -0.6346367252 -4.0569713618
C -13.7847423807 0.4543479893 -4.8659797858
C -12.8526866571 0.6288019605 -6.0739278484
C -11.4580880035 0.5516044801 -5.4357664546
N -10.7363931174 -1.570785914 -2.1553369603

C	-10.0395172875	-1.7194304784	-0.8590748417
C	-10.0698571771	-3.2331726293	-0.591159854
C	-10.1022947498	-3.8452115534	-2.0009131533
C	-11.0142012059	-2.8820562106	-2.7700171105
N	-9.121218605	0.1035618793	-3.5786048578
C	-8.293367927	1.3153218561	-3.4888183571
C	-6.8676779398	0.7611086696	-3.5803550567
C	-7.0195050353	-0.4025281387	-4.5760980428
C	-8.4116955569	-0.9905701689	-4.2577298415
N	-13.8303240237	4.5090178185	-3.1202431818
C	-13.1847018962	4.366589174	-4.4402159028
C	-14.3597121502	4.2577981232	-5.4489628477
C	-15.6289316027	4.5144839799	-4.6120196849
C	-15.2167691482	4.0288008158	-3.2202764792
N	-13.8702165604	5.1075897024	-0.5454688953
C	-14.2431914933	6.5146814417	-0.7954656346
C	-15.6178116885	6.6626259943	-0.1264094416
C	-15.5281279201	5.6726170106	1.0450193728
C	-14.7829374556	4.4777387567	0.4332488593
N	-5.4942883354	-2.8095996885	-0.4016175215
C	-5.9264314229	-2.3819763759	-1.7400954388
C	-5.962927152	-3.6919688593	-2.5391825539
C	-6.3596944551	-4.7344181146	-1.4802817447
C	-5.6101042818	-4.2638008221	-0.223252007
N	-5.5802278961	-1.8651635881	2.0461485533
C	-5.1046914566	-1.2675012866	3.3183924437
C	-6.2924438743	-0.4413005028	3.8281512395
C	-7.5081243576	-1.2011373896	3.2744243828
C	-7.0376738472	-1.6461260279	1.8822485261
N	-0.9065060742	2.3810253402	2.7858497231
C	0.1231105077	2.9548726464	3.6582030572
C	-0.2577871534	4.4522423234	3.7158304561
C	-1.0852529894	4.698601821	2.4201938999
C	-1.0773708193	3.3414923303	1.6891619086
H	-6.3249182853	0.996716589	-0.0750337113
H	-9.1395288472	1.2313842461	-0.4876836711
H	-12.3286320616	3.3116617575	1.5057589585
H	-3.5552869298	2.6545670648	2.7733975628
H	-5.3816692007	3.9111870445	3.8985848707
H	-10.7368430789	4.2670049312	3.1658191292
H	-15.8239797826	4.4593076516	-2.4173296074
H	-16.5036086867	5.3863109266	1.4522590705
H	-15.2827849192	2.9310317151	-3.1412960157
H	-14.3947736967	3.2500896075	-5.8759921915
H	-16.5087849043	3.9906468483	-5.0014679471
H	-15.8569793281	5.5865508065	-4.5738541554
H	-14.2539886036	4.9612769556	-6.2814430479
H	-12.546549951	3.4721642947	-4.4975154717
H	-12.5520489869	5.240353124	-4.6289299384
H	-14.262379955	6.7237617007	-1.8699455273
H	-16.4101308062	6.352718894	-0.8203636009

H	-15.8263630486	7.6918933893	0.1850037748
H	-14.9361633224	6.1099227177	1.8598890231
H	-11.5091551604	5.7698739342	0.1530187805
H	-14.2306525503	3.899912385	1.1786007695
H	-11.1195915303	6.9554148808	-1.111099876
H	-8.8930676466	6.3667500336	-0.3405252703
H	-9.3779931975	4.6877778832	-0.039785116
H	-8.2758355821	4.494224874	-2.2143844571
H	-8.9919330396	6.0191377508	-2.7684203077
H	-10.3671273465	3.3126218703	-2.4230249595
H	-10.5841617785	4.5218177205	-3.6971833455
H	-13.8201355879	1.3762119845	-4.2745707397
H	-14.809428061	0.1788945533	-5.1389072934
H	-13.3426980947	-1.6381779983	-4.4136743507
H	-12.9948243647	-0.1965170897	-6.7839055201
H	-13.3028108059	-0.5820730471	-2.9877683649
H	-13.0030340995	1.5679619324	-6.616580111
H	-11.1524928695	1.5456968222	-5.0783494229
H	-10.688942945	0.193004403	-6.1296001897
H	-10.5401745444	-1.1425584215	-0.0751272929
H	-9.0032066338	-1.3549071949	-0.9312401018
H	-8.9387037195	-1.3164946465	-5.1660534993
H	-8.3380116946	-1.8620556794	-3.5937222247
H	-6.2259836709	-1.1516207037	-4.4885941065
H	-7.0027699903	-0.0179472527	-5.6035906655
H	-6.5472596939	0.3904856246	-2.5999153685
H	-6.1410957878	1.5118804672	-3.9077962833
H	-8.4990945681	1.8518623816	-2.5621584272
H	-8.5028158863	2.0020021631	-4.3264514829
H	-9.2139169013	-3.5611372105	0.0079337589
H	-9.0986990259	-3.8316695986	-2.4428635513
H	-10.4657380914	-4.8785744172	-2.0187177036
H	-10.9838929236	-3.4996562529	-0.0461471825
H	-10.8174205175	-2.8652712251	-3.8454889209
H	-12.0720029403	-3.1514651264	-2.6290124719
H	-15.4787709702	3.7840681124	-0.0584151528
H	-13.5108631902	7.1921219534	-0.3316615117
H	-0.4087450023	0.138689707	0.0094814315
H	-0.6659485054	5.4942654203	1.7955962921
H	0.7153934336	-1.1986966201	0.3130449896
H	3.3429102334	-0.0574583993	1.6313935064
H	2.2733552201	0.5228172949	-0.4366259701
H	1.3313242757	1.7948700847	0.3516119926
H	2.8022731096	1.5286340986	2.1758384145
H	1.7620832903	-1.1618679526	2.9257133862
H	1.3582615924	0.369596137	3.7073814369
H	1.1274996957	2.8390475158	3.2145324388
H	0.6331341876	5.0868328214	3.7733669558
H	-0.863760361	4.6549007254	4.6050813836
H	-2.1143512192	4.9792600581	2.6670959276
H	-3.0912411905	1.3028087854	4.5039952311

H -1.9876185621 3.1422430351 1.1202259981
H -1.7837262047 1.712054197 5.6259383685
H -2.924528081 0.1563874226 7.0517997725
H -3.6854057323 -0.6458262673 5.6720236708
H -1.972207443 -2.2802422765 6.2672836525
H -0.7930803984 -0.999690677 6.6193452354
H -1.9099512088 -1.7821841018 3.8745978103
H -0.2045761853 -1.6168626175 4.3334871238
H -0.6389037697 -2.9091763052 2.062304865
H -0.2668543103 -4.0446830317 0.7474331139
H -2.1927764044 -3.296314281 -0.5391974845
H -2.2257496271 -5.4266964278 1.2908519602
H -1.7079106639 -1.7731313604 0.2336319752
H -1.589928125 -5.0480313444 2.9018787268
H -3.2373382273 -3.3339134645 3.2614645014
H -4.1909366956 -4.4157098378 2.2455961336
H -5.2361313249 -1.6380949225 -2.1430782633
H -6.9267510893 -1.9246020308 -1.6984279226
H -7.5275065657 -2.5654017508 1.5421895258
H -7.2465585339 -0.8618569988 1.1429118804
H -8.4123653428 -0.5862262055 3.2211538954
H -7.726858461 -2.0784603507 3.8975298364
H -6.2538099462 0.5662696711 3.4083417722
H -6.3011305087 -0.3537638265 4.9205704172
H -4.2042106778 -0.6671054638 3.1579314929
H -4.8617277225 -2.07024752 4.0307196686
H -6.6580159824 -3.6511869573 -3.3850245281
H -7.4401642136 -4.6992480756 -1.3038464744
H -6.1003794109 -5.7611603957 -1.7597191272
H -4.9631864248 -3.9153273348 -2.9335062936
H -6.1491033482 -4.5140671746 0.7011542003
H -4.6145644012 -4.729576129 -0.1608484921
H -0.2281905245 3.2916632847 0.9850527082
H 0.1276868587 2.4627788561 4.6333944577

IVbbH+
C 1.1827560275 1.3857356326 1.6754091483
N 0.7992643409 0.2124129264 2.4989143481
C 1.9252775232 -0.2479819358 3.3434428587
C 2.9288915801 0.9102933201 3.2661239031
C 2.7072012805 1.454237884 1.8462986511
P -0.7643880171 -0.3144287893 2.6983652214
N -1.6494994003 0.5135455219 1.678422297
P -2.7404311354 0.3376472418 0.5153876301
C -3.2671464556 2.0105536248 0.0581327244
C -4.4922982006 2.1457341623 -0.6069993123
C -4.9804044241 3.4213402344 -0.8674696216
C -4.2142910146 4.5480171622 -0.5006566266
C -2.9772938926 4.4427301999 0.1259814872
C -2.5169820361 3.1525839369 0.4121005132
O -4.8721171354 5.7125874491 -0.7978911098

C	-6.0949706227	5.3352483499	-1.344755608
C	-6.215624447	3.9385272821	-1.4105165555
C	-7.38475765	3.356964369	-1.911003444
C	-8.4169204274	4.1827942329	-2.3585340996
C	-8.2618025683	5.5866284381	-2.2659243364
C	-7.1037077728	6.1918922559	-1.7680185916
P	-10.0634227546	3.4882795667	-2.9357321834
N	-9.5839007686	1.8316366871	-3.0063229689
P	-10.5930060747	0.664435557	-2.6869582716
N	-9.8917987442	-0.7385221564	-3.3100594379
C	-8.8427249006	-0.6743984183	-4.3540825744
C	-8.7600685272	-2.1110592051	-4.8959202531
C	-10.1777666767	-2.6631944553	-4.6798877586
C	-10.5940198203	-2.0360716225	-3.3400222977
N	-0.9202878722	-1.9675934518	2.5212303529
C	-0.9444317779	-2.5498764648	1.1497020971
C	-0.413903104	-3.9841383113	1.3307006394
C	-0.6983674022	-4.2935574452	2.8083571025
C	-0.3923047153	-2.9579421104	3.4908484282
N	-1.1202044608	-0.0893642314	4.3173540245
C	-0.7307805471	1.1868621828	4.9751333646
C	-2.0314124216	1.7077690783	5.5958721353
C	-2.7482307245	0.4120521938	6.0011513448
C	-2.4345304791	-0.5496713195	4.8422818386
N	-4.0143277371	-0.6221006748	0.7276502559
P	-5.3541413582	-0.5568634181	1.5930821335
N	-5.8400820628	0.8915973222	2.2464895937
C	-4.984907771	1.6774419049	3.1615660286
C	-5.4136672998	3.1265154445	2.9029687935
C	-6.9064452187	2.9928100921	2.5633359705
C	-6.9832902702	1.6939880974	1.7450943089
N	-5.1922682985	-1.5482189369	2.9227260203
C	-4.4930066587	-2.851375015	2.8168657418
C	-4.7786858711	-3.5294363403	4.1654546273
C	-6.1549365162	-2.9712425445	4.5612515792
C	-6.0734748598	-1.5062112277	4.1123750249
N	-6.5962377484	-1.0767930515	0.6202456258
C	-6.5792709453	-1.0914545681	-0.8577485353
C	-7.4170614543	-2.3266186695	-1.1940695313
C	-8.5142025759	-2.285385516	-0.1213145521
C	-7.783243548	-1.787940374	1.1437104076
N	-10.194310742	4.027681116	-4.5157291644
P	-9.3839579839	4.2157220991	-5.8492628509
N	-9.4378151937	5.8134112482	-6.3330162543
C	-9.2763595907	6.3011798529	-7.7097467056
C	-10.1646267181	7.5500912678	-7.7320805304
C	-9.9250414888	8.1506504735	-6.3363278359
C	-9.7556996029	6.9226906451	-5.4113188036
N	-7.764135743	3.7498621337	-5.9080544228
C	-6.6418693792	4.6745279194	-6.1295138871
C	-5.6578731864	4.3045258066	-5.0140212234

C -5.7949302362 2.7747151292 -4.9233667592
C -7.2597010443 2.480425974 -5.3429277145
N -10.0964251312 3.4265347553 -7.1669485513
C -9.7090404866 2.0613068354 -7.5781867427
C -10.9361967549 1.5179274339 -8.3481219836
C -11.7885101374 2.7639166316 -8.6481895693
C -11.5283067258 3.6478575184 -7.4258152832
N -12.1556119159 0.660541746 -3.3353778781
C -13.1991587534 1.5907446791 -2.8182045602
C -13.6925019507 2.3405332172 -4.0604948172
C -13.5839664364 1.2641087832 -5.1497247053
C -12.2441543247 0.5934509822 -4.8191934825
N -10.9165830027 0.4846515475 -1.0504827898
C -10.0915594967 1.1423347538 -0.0218049312
C -10.9188923957 0.9642930004 1.2574477976
C -11.5978084264 -0.3989744586 1.0505327724
C -11.9312146049 -0.4072091894 -0.4523313453
H -5.076928592 1.2749142861 -0.8817721513
H -7.5023359319 2.2786098883 -1.9800631499
H -9.0809396811 6.222994888 -2.5906239142
H -1.5785149368 3.0222545183 0.9413830084
H -2.4109026539 5.3253071658 0.4055252038
H -6.9982241766 7.270547919 -1.7058670294
H -11.72011637 4.7101769918 -7.6107546001
H -10.7363216228 8.8058561095 -6.0037855248
H -12.1475143413 3.3438314174 -6.5693485162
H -11.5017129306 0.8289118739 -7.7094197781
H -12.8502619994 2.533396956 -8.7859814163
H -11.4291994863 3.2673424358 -9.5539640107
H -10.6483828577 0.9677479458 -9.2496396474
H -9.4813863061 1.4164046625 -6.719751317
H -8.8085733404 2.106712246 -8.2023574569
H -9.5723257733 5.5304393367 -8.4252207741
H -11.2146032285 7.2529743788 -7.8475752985
H -9.912430633 8.238360815 -8.5455467535
H -9.0030339238 8.7452013403 -6.3446258181
H -6.976427663 5.7122970223 -6.0768808639
H -8.948425061 7.0854487328 -4.6865041987
H -6.1917419668 4.5106740624 -7.1225399613
H -4.6331295339 4.6301162948 -5.2254291143
H -5.9840060388 4.7717418591 -4.0800066096
H -5.5773601043 2.3990269145 -3.9183703513
H -5.0974749249 2.2897221927 -5.6171978181
H -7.8844256073 2.162615002 -4.5045872658
H -7.2836275944 1.6815237692 -6.0976814907
H -13.0139834424 3.1733361403 -4.2814491216
H -14.7065252351 2.7351695668 -3.9371054839
H -14.0155019 1.0060145107 -2.3704394197
H -14.4047015751 0.541267074 -5.0552603772
H -12.7926848571 2.2586275944 -2.0547043854
H -13.604304609 1.6629853197 -6.1686355964

H -11.4189219135 1.1523203812 -5.2803291983
H -12.194165042 -0.4445001824 -5.1650662854
H -9.9160208001 2.1917046823 -0.2698497339
H -9.1092079577 0.6568188669 0.0685734284
H -11.6772712032 -1.8980754559 -3.2591495038
H -10.2728183441 -2.6679088462 -2.4976530034
H -10.2200539222 -3.7572198969 -4.6636242347
H -10.8421134732 -2.3136447174 -5.4791775579
H -8.0341949673 -2.7015746842 -4.323430389
H -8.4447833139 -2.1329370605 -5.9441667855
H -7.895311729 -0.3209944852 -3.9339209194
H -9.1210548821 0.0288877976 -5.1467654121
H -10.3068657719 1.0082938649 2.1652609687
H -10.89629719 -1.2083949712 1.2872381787
H -12.4880208104 -0.539574429 1.6717249539
H -11.6724225497 1.7583422435 1.32286181
H -11.8863161196 -1.4166846398 -0.8760775417
H -12.941057102 -0.0187238273 -0.6322973722
H -10.6653597809 6.6853142769 -4.8495326443
H -8.2274229695 6.5697732264 -7.9201870542
H -0.3213229171 -1.9723721466 0.4555398386
H 3.1980515904 0.8023785493 1.1129806807
H -1.9726418575 -2.5431736435 0.7703091299
H -1.7546268676 -4.5532974577 2.9514558863
H -0.8884478159 -4.6873871395 0.6398755969
H 0.666833004 -4.0132993045 1.1469476332
H -0.0895952951 -5.1120459294 3.2041690057
H -0.8789636488 -2.8467237991 4.4617829243
H 0.6907306778 -2.8439795611 3.6403556324
H 2.3601590228 -1.1687753701 2.9301961207
H 3.9568181985 0.5856650956 3.4532055359
H 2.6741157956 1.6765963898 4.0092350771
H 3.0913520032 2.4689625656 1.70544921
H -0.2918200871 1.8870225495 4.2596924906
H 0.7016661488 2.2996552117 2.0519319742
H 0.0205445211 0.9830332155 5.7501765923
H -1.8532672054 2.384748552 6.4371685928
H -2.6139850848 2.2471426836 4.8396747162
H -3.8255983072 0.5395829669 6.1461979119
H -2.3285969569 0.0277295296 6.9387989119
H -3.2034909528 -0.501345582 4.0644150539
H -2.3712628035 -1.5888299683 5.1801094323
H -4.0260561741 -3.2292019327 4.9045569826
H -4.7585287693 -4.6211027989 4.0928323903
H -4.8974412454 -3.443643936 1.9829556555
H -6.9464356267 -3.4914220939 4.0073920543
H -3.427376108 -2.6912633974 2.638146524
H -6.3729739345 -3.0683017067 5.6292818081
H -5.6150332049 -0.8837801424 4.8937230751
H -7.0508447824 -1.0751681104 3.8788720767
H -5.5513090906 -1.1461482709 -1.2212695565

H -7.0551633229 -0.1856319329 -1.2628675621
H -7.9251629192 1.1641833511 1.906205762
H -6.890029143 1.897403138 0.6741066536
H -7.2964580514 3.8456961709 2.0001784097
H -7.4969413471 2.8951736248 3.4827209864
H -4.8625898253 3.5251875952 2.0464871275
H -5.2248435756 3.7764404015 3.7632614678
H -3.9237720696 1.5142037586 2.9519217359
H -5.1724635778 1.3851655458 4.205023719
H -7.8258406419 -2.2836337145 -2.2042116512
H -9.2812526701 -1.5688812793 -0.4279088577
H -9.0013198752 -3.2514667101 0.0440386606
H -6.8038205617 -3.2313134668 -1.09729578
H -8.4196309192 -1.1276108889 1.7476958449
H -7.4688032789 -2.6235085449 1.7816230598
H 0.873822186 1.2524586613 0.6356264196
H 1.593389863 -0.4588632915 4.3654432801
H -2.1121621188 -0.2126443099 -0.6193270826

Vaa
C 0.1232232663 0.1260598671 -0.2578049867
C -0.0176616577 0.1473862326 1.1634872205
C 1.0240096986 -0.1188827103 2.0132153747
C 2.2920048863 -0.4006915484 1.4141533995
C 2.4144632351 -0.4371478267 0.0233500019
C 1.3256447509 -0.1698558112 -0.8551504564
C -1.4716129826 0.5270472655 1.0127631913
C -1.3313950174 0.4974656794 -0.4082642259
C -2.3936070826 0.7641173001 -1.2395301981
C -3.6338144385 1.0518586325 -0.6012061893
C -3.7865154915 1.0632402142 0.78717751
C -2.6597279573 0.8146970371 1.6322135625
H -2.3209732575 0.7483408979 -2.3247548126
H -4.5109692835 1.2452496973 -1.2118930694
H 3.3921975751 -0.6555626881 -0.39637373
H 1.4667680103 -0.192903024 -1.9334797531
H -2.7732036358 0.8216718689 2.7122681381
H 0.9242442602 -0.0882236952 3.0944491194
P -5.4120193355 1.4916243069 1.6042385993
N -6.4638063532 1.0750144506 0.3129619224
P -7.6864454201 1.9356347872 -0.1543366674
N -7.7451482037 1.8999150953 -1.8556265938
P -5.4405201215 -1.2940350386 2.7595617394
N -9.1377010682 1.3908181392 0.5418690733
N -4.2465566558 -1.7750082662 3.8625023745
N -5.5415648783 0.2715604603 2.7678355926
N -6.9706651391 -1.9398410404 3.1667838614
C -8.7486159466 2.644808703 -2.6097568779
C -7.2742990094 0.7054580582 -2.553560362
C -3.6736065612 -2.3618652286 0.9426281062
C -3.9656686595 -3.1754937535 4.1543141865

C	-3.7377809465	-0.8522527888	4.8712911069
C	-8.1385187616	4.080972482	1.4796309663
C	-7.1707339343	-3.3808644329	3.2987843044
C	-7.8054164478	-1.1721545711	4.0910573309
C	-10.4313319396	1.9262807606	0.1311564035
C	-9.21071831	0.0344669717	1.0786778521
C	-6.0177912274	-2.2969994617	0.2940303956
N	-5.0610930759	-2.2346203957	1.4044861769
H	-2.6404784758	-0.9144282451	4.9140991358
H	-4.0324246088	0.1663748976	4.617545474
H	-4.134083998	-1.095982858	5.8705986675
H	-8.5595188396	5.0918929196	1.3947482024
H	-7.2629417	4.1132356181	2.1434084204
H	-8.8870285888	3.4253736372	1.9267106285
H	-5.8259191849	4.4646425216	0.128948719
H	-7.1231370971	5.4678366913	-0.5785132772
H	-6.4919150412	4.0658273911	-1.4732939546
H	-4.4575254484	-3.5088153305	5.082638161
H	-2.8822859196	-3.3153842915	4.2769078607
H	-4.2962876074	-3.8147735281	3.3340982704
H	-6.5016919633	0.2148023613	-1.9618822814
H	-2.9760642433	-2.2007838931	1.7640422317
H	-8.0919324099	-0.0122733306	-2.7389949509
H	-6.8502789782	0.9957635632	-3.524534107
H	-3.5243666638	-3.3746103236	0.5434037676
H	-9.6604432941	2.0535215728	-2.7958421591
H	-9.0234080066	3.5587353783	-2.0800835228
H	-8.3277368665	2.9274745453	-3.5843775807
H	-8.2261109293	-0.2915373359	1.4091286838
H	-5.8913496152	-1.4472860031	-0.3885758175
H	-9.5923312146	-0.6826432885	0.3316513159
H	-5.8584660677	-3.2347783047	-0.2547926017
H	-9.8921752881	0.0230870139	1.9399222831
H	-7.0415455459	-2.2807533504	0.6700714103
H	-10.8794345607	1.3480984699	-0.6937946982
H	-11.125760492	1.891907811	0.9818167918
H	-10.3339351095	2.9674339471	-0.1828650346
H	-7.6496466135	-0.1056706731	3.9270757778
H	-8.2276383678	-3.6148168928	3.1124796682
H	-8.8606190112	-1.4145368655	3.9081814702
H	-7.5782281891	-1.4083290039	5.1451572415
H	-6.9166028615	-3.7502150854	4.3065231849
H	-6.5675306113	-3.9197687844	2.5659090952
C	-6.7420803794	4.4428456665	-0.4798224393
N	-7.7797017225	3.6063730808	0.1370038344
H	-3.4446305072	-1.6340043936	0.1549397877
P	3.7179894276	-0.8144012288	2.5493121976
N	5.0116162078	-0.4669667221	1.4757864402
P	6.2736731587	-1.3727345782	1.2753196219
N	6.6481251149	-1.4108841774	-0.3857171747
P	3.5814075416	2.0166584304	3.5648060751

N	7.5857962239	-0.8271784568	2.206578415
N	2.2293956826	2.5692117125	4.424073221
N	3.6413196727	0.4518955911	3.667513801
N	5.0333717702	2.6635662329	4.1954231573
C	7.7438259993	-2.2177975641	-0.91406946
C	6.3675199629	-0.227559302	-1.1960974119
C	2.171535326	3.0354577411	1.4315835348
C	1.9569955486	3.9877537113	4.6201353737
C	1.517015574	1.6992299312	5.3532678889
C	6.3608497907	-3.4574145606	3.0429004666
C	5.2600943424	4.1052041361	4.2609344658
C	5.6642372003	1.9475819648	5.3045046695
C	8.9190240512	-1.400318744	2.0569572474
C	7.5909243273	0.5451109867	2.7060523039
C	4.5860584925	2.8742454821	1.1813942525
N	3.4590818791	2.8882833345	2.1197120107
H	0.4324145969	1.7983892423	5.1998793531
H	1.8134418386	0.6636762788	5.184568469
H	1.7411457636	1.9644623744	6.399449487
H	6.7587209514	-4.4807439042	3.0727924829
H	5.3796853051	-3.4414897504	3.5383286876
H	7.0371564051	-2.8018922578	3.5932867552
H	4.321408248	-3.8503413358	1.3114151382
H	5.6972752076	-4.905834572	0.8836645088
H	5.2733264476	-3.5272898737	-0.1578211265
H	2.3013071028	4.340725454	5.6059405124
H	0.8743526319	4.1665396241	4.5563102758
H	2.4417729069	4.5847842573	3.84577348
H	5.5199478162	0.3131268526	-0.7754064002
H	1.3443977955	2.9349482468	2.133759914
H	7.2354647989	0.4523737954	-1.2462577326
H	6.1200845187	-0.5386764832	-2.2201922986
H	2.1272171966	4.0321232729	0.9708503984
H	8.6968679688	-1.6643185456	-0.9467071743
H	7.8798108204	-3.1179359169	-0.3119844534
H	7.4997416599	-2.5255333009	-1.9400549351
H	6.5699098554	0.9013333478	2.8324292646
H	4.5242387898	2.01609324	0.5006784429
H	8.1253731701	1.2279883903	2.023359466
H	4.5718163347	3.8050312379	0.5981417085
H	8.0972528978	0.5734630739	3.6803380558
H	5.5324174815	2.8111492831	1.719780539
H	9.524068102	-0.8600619532	1.310143813
H	9.4474417445	-1.3487435473	3.0188241473
H	8.8549117632	-2.4499755487	1.7632271312
H	5.5070410304	0.8751660535	5.1863311394
H	6.3410877892	4.2994382428	4.240802234
H	6.7423213967	2.1555125178	5.2999930063
H	5.2596722133	2.262811823	6.2819951383
H	4.8547263936	4.5530726471	5.1837249595
H	4.8075742558	4.6036719721	3.4018761116

C 5.3318690469 -3.8704305076 0.876860245
N 6.2639491437 -3.0312017563 1.6410584644
H 2.0471610709 2.2783627891 0.6480523433

VaaH^+
C 0.0983100446 -0.1374371887 -0.3958644003
C 0.0400534956 -0.0425878673 1.0329603074
C 1.1385266638 -0.2412731869 1.8244177128
C 2.3602282693 -0.5433915748 1.1383922205
C 2.4107102889 -0.6555432419 -0.2503235149
C 1.2608586783 -0.4505635695 -1.0617478178
C -1.4240887446 0.3057915534 0.9457068841
C -1.3620109093 0.2063531262 -0.4766438439
C -2.4694000795 0.4220715256 -1.2648381316
C -3.6653950329 0.7533074322 -0.5744252496
C -3.7390576635 0.850949862 0.8200651609
C -2.5760570813 0.6244292762 1.6174730052
H -2.4573499614 0.3409356739 -2.3489424646
H -4.580725089 0.9119011424 -1.1377738113
H 3.3605493195 -0.8973565853 -0.7188544667
H 1.3252760915 -0.5375464164 -2.1424991123
H -2.6395391128 0.6863070959 2.7013238779
H 1.1081023255 -0.1579780981 2.9072006031
P -5.2873334885 1.3798191775 1.731472338
N -6.3932027957 1.2896664312 0.4392058617
P -7.6807289582 2.1778191763 0.2632949454
N -7.553814038 3.0360407646 -1.1877444537
P -5.710244118 -1.4877720149 2.5525389247
N -9.0709862451 1.2255389276 0.1951652282
N -4.9317854576 -2.2714195868 3.8454798058
N -5.5018920897 0.0565526214 2.7596047337
N -7.362575725 -1.8644084421 2.4634804055
C -8.6954928783 3.7394017133 -1.7610994743
C -6.2655357486 3.5027205696 -1.692079817
C -3.6842002324 -2.6769037272 1.1289549675
C -5.1192440278 -3.7063072217 4.0461348253
C -4.696067883 -1.5431619965 5.0917506844
C -8.4562571095 2.8427774117 2.7929677768
C -7.8717214021 -3.2181747048 2.2641426273
C -8.3352244536 -0.9728610385 3.0892049681
C -10.4370238435 1.6984898444 0.3957311078
C -8.992332824 -0.0562262407 -0.4998897467
C -5.7552156308 -2.256922467 -0.077875327
N -5.1116263156 -2.3650488388 1.2331159992
H -3.7946959308 -1.9436048794 5.5757440331
H -4.5535174903 -0.4845226937 4.8744795278
H -5.5375771565 -1.6499358319 5.7966175057
H -9.0959857264 3.5864634878 3.2855081868
H -7.5582921051 2.6865029022 3.4081412678
H -9.006668285 1.9027247781 2.7355009889
H -6.4342378343 4.5183770338 1.9729283987

H	-8.0209564425	5.3359146322	2.0015112276
H	-7.2488562393	4.9676614882	0.4529230493
H	-6.0210057247	-3.9333286075	4.6380645341
H	-4.252321584	-4.110655003	4.5847405397
H	-5.1905760371	-4.2222735298	3.0864517375
H	-5.4550075341	2.9909790558	-1.1740794983
H	-3.2209137183	-2.6673660934	2.1162172844
H	-6.1883980808	3.2893194778	-2.76719419
H	-6.1442835858	4.5894477726	-1.5521193431
H	-3.5655737627	-3.6781979717	0.6917948258
H	-8.628795874	3.7101779568	-2.8564254804
H	-9.6332581193	3.265805281	-1.4711046137
H	-8.7315968852	4.7964771442	-1.4500256135
H	-7.9511876702	-0.3672548004	-0.5746275063
H	-5.3377506438	-1.4290340268	-0.665783884
H	-9.4254263848	0.0089323885	-1.5106253008
H	-5.60243607	-3.195477695	-0.6271758711
H	-9.5467427271	-0.8191812515	0.0631973448
H	-6.8251430165	-2.0902237109	0.0363089571
H	-11.0021211961	1.7093757807	-0.5497443869
H	-10.9627887808	1.0261783189	1.0891597729
H	-10.4394383218	2.7042066952	0.8176911071
H	-7.8791991697	-0.0002543292	3.2623852172
H	-8.7659161179	-3.1810323648	1.6260008547
H	-9.2022845569	-0.8440974558	2.4277417719
H	-8.6900641711	-1.3784656355	4.0505781293
H	-8.1564078116	-3.694022706	3.2156949675
H	-7.1278707546	-3.8443683942	1.7695947504
C	-7.4082956272	4.5987277464	1.4676297422
N	-8.1245803497	3.3200608184	1.4447615138
H	-3.1618021435	-1.9539914384	0.489111743
P	3.8597029479	-0.6656978961	2.1284930405
N	5.0129782061	-1.2815394646	1.1897560698
P	6.5341571088	-1.6395500146	1.4841989627
N	7.0194199454	-2.598392054	0.2090672313
P	3.8004437537	2.2138244781	3.0126777654
N	7.480197465	-0.25990685	1.5960676785
N	2.351398697	2.3853671442	3.830255115
N	4.2280793285	0.6910090474	2.9106448916
N	5.0518027353	2.9572933001	3.839689834
C	8.4268674094	-2.9639047992	0.0488219062
C	6.090871181	-3.4199056508	-0.572510023
C	2.3772152284	2.8562251927	0.7515866098
C	1.5063978174	3.5837129998	3.8054593048
C	2.0752537286	1.5122741986	4.9740367194
C	6.6647432488	-1.779819586	4.2027131129
C	4.912574677	4.3344856809	4.309395347
C	6.4253438365	2.4592308131	3.8336602768
C	8.812589256	-0.2064405218	2.2033371088
C	7.2491102595	0.803601659	0.6135367958
C	4.6854317755	3.6364328997	0.8748005687

N 3.5356926317 3.1172949548 1.6199744295
H 1.0127359987 1.2390855322 4.9738943172
H 2.6721849941 0.6008497159 4.9129516999
H 2.301724331 2.0173524592 5.9241122366
H 7.4077222414 -2.1234756842 4.9321298862
H 5.6630290115 -2.0095125025 4.5939358998
H 6.7499042963 -0.6978866797 4.0987810807
H 5.6481418528 -4.1473801799 3.2352145408
H 7.3436022852 -4.3167297909 3.7545051249
H 6.9399667316 -4.3709599948 2.0314497192
H 1.5516601739 4.1145999359 4.7672022924
H 0.4641524581 3.292103894 3.6239813559
H 1.8201840369 4.2642064905 3.0140877577
H 5.0697737204 -3.0689227204 -0.4252469406
H 1.5494433887 2.4352038429 1.320490926
H 6.3456303669 -3.336939802 -1.6359913528
H 6.1545109923 -4.4796014744 -0.2829820596
H 2.0504685027 3.8015318885 0.3023947087
H 8.7014664612 -2.9022222461 -1.0109811747
H 9.0744114754 -2.2873638221 0.607564322
H 8.6214415338 -3.9896535527 0.3956439877
H 6.2211906299 0.7655287974 0.2521587103
H 5.0304987741 2.9241779487 0.1108411014
H 7.9337711219 0.7133788886 -0.2424983715
H 4.392695776 4.5646409796 0.370651096
H 7.4138157299 1.7763302284 1.0900547969
H 5.5171356195 3.8596416237 1.5445290152
H 9.5996953968 -0.1693296146 1.4361209233
H 8.8951861677 0.700620285 2.8160279784
H 8.9820577061 -1.074700758 2.8404743368
H 6.4528301257 1.45460295 3.4126231352
H 5.4046107667 5.048974724 3.6319510147
H 7.0811724653 3.1152329848 3.2395693319
H 6.8135754528 2.4306306625 4.8600633088
H 5.3716976748 4.4288456601 5.300860095
H 3.8620759394 4.6139554455 4.3964609973
C 6.6895476386 -3.8976978905 2.9817210999
N 6.9061116866 -2.447550734 2.9150964634
H 2.6294835513 2.1511483313 -0.0504830923
H 3.5367684257 -1.5914901268 3.146705834

Vbb

C -0.0088922438 0.5631888768 -1.2757104569
C -0.0658206584 0.5165064702 0.1504561352
C 0.8961546785 1.0617390769 0.9545792811
C 2.0348210729 1.6333575722 0.3090477909
C 2.0946069507 1.6621882899 -1.0870417801
C 1.0636215868 1.1353561953 -1.9200381424
C -1.3661222445 -0.231254823 0.081629549
C -2.3724440162 -0.8375061103 0.7810194234
C -3.4728226838 -1.3469537218 0.0263346325

C	-3.4524389117	-1.2577310736	-1.3684534885
C	-2.3755158298	-0.6680581741	-2.0942612287
C	-1.3412543029	-0.1575695163	-1.3442691963
H	-4.310284605	-1.6466068997	-1.9093561364
H	2.9799937616	2.0955723851	-1.5433188502
H	1.1526383851	1.1854540478	-3.0031412299
H	-2.4030430711	-0.6260153961	-3.1810143124
H	0.8205443808	1.0241255814	2.0358550872
H	-2.3585123055	-0.8906012884	1.8640242506
P	3.353946824	2.3963383263	1.4002463498
N	4.514438915	2.6701774515	0.204221372
N	3.9511055736	1.0644146713	2.3075318741
P	6.0417929387	2.9781250334	0.2986982758
P	3.3677898331	-0.2448739552	2.9166700757
N	6.4652346234	4.1486611441	1.4313501573
N	6.508421361	3.4535345713	-1.2417931642
N	7.153795715	1.7586119729	0.6716030399
N	4.5590617246	-1.0811770485	3.7683302028
N	2.1045876568	0.0242430249	4.0242136036
N	2.7905758011	-1.3994607562	1.8488609091
C	4.8438470423	-2.5201236738	3.6542214635
C	5.9988574675	-2.7357610301	4.6470283306
C	5.743051081	-1.6713078499	5.7265350283
H	4.5888901755	0.1884617234	5.471874864
C	1.6805240684	-0.9918817361	5.011050924
C	1.1343368693	-0.1798540643	6.2011276273
H	0.6292332771	1.9736562757	6.2748793906
C	1.8375231583	1.3863900467	4.5367072656
C	1.5282885648	-2.1556425723	1.9225011235
C	3.4679171072	-1.5902984622	0.5498768113
C	2.7968668619	-2.8495389747	-0.0115106678
C	1.3551297303	-2.7262757261	0.5048364803
C	5.6362150264	3.4053804054	-2.4284201665
C	7.9044803894	3.8169332323	-3.1158550255
C	6.4524989987	4.1544880364	-3.4917530891
C	7.8801091507	3.8569316365	-1.5788856161
C	5.6085532661	5.357433184	1.508399201
C	6.4513686119	6.3540067601	2.3174907717
C	7.8906653017	5.9847679191	1.9257638376
C	7.8376292636	4.4518671538	1.890128179
C	7.9333707331	-0.4716065843	0.5160792039
C	7.3852976176	-0.2627063982	1.9348582807
C	7.3018550348	1.2657465911	2.0672572932
C	7.2131064708	0.6232772005	-0.2774076925
H	6.1056413527	0.1459495372	4.5598485408
H	6.9570451245	-2.5392717777	4.1505874128
H	6.6278358343	-1.4436154937	6.3306112641
H	4.9472537012	-2.0066297795	6.404712439
H	6.024705188	-3.7578994767	5.0393074561
H	5.1257218801	-2.7971168104	2.6318183725
H	3.9684279063	-3.1294377261	3.9270415208

H 2.5185536659 -1.6375220259 5.303162889
H 1.9299147379 -0.003874885 6.9364348227
H 0.3130911963 -0.6930771652 6.7124959686
H -0.2391846589 1.0421771737 5.0371789296
H 0.693323007 -1.5067779798 2.205634391
H 1.5505027383 2.0676668864 3.7313858973
H 1.6003468155 -2.9645610877 2.6665514015
H 0.8081814533 -3.6746375678 0.5062247137
H 0.79739291 -2.0167598555 -0.1132132804
H 2.8589730068 -2.9000331 -1.103610396
H 3.2754232149 -3.74822151 0.4007112658
H 3.3067423406 -0.722491913 -0.1019277076
H 4.5504895916 -1.7111864746 0.6804857247
H 6.3790753416 -0.681974161 2.0208449971
H 8.0119847425 -0.7144537354 2.7121317673
H 8.2242651814 1.676340104 2.5005849703
H 9.0183681357 -0.3038985846 0.4924502841
H 6.4522988903 1.5691636991 2.6849294907
H 7.7353821614 -1.4721272136 0.1154965068
H 6.2031569365 0.2900675304 -0.5618694197
H 7.7454089297 0.9132078663 -1.1906655934
H 4.6483910664 5.1204849033 1.9764236349
H 5.403093542 5.7535283203 0.5016769273
H 8.617301704 3.1802035332 -1.1283301119
H 8.0941549684 4.8728698824 -1.2090749863
H 8.6405853489 4.5063717875 -3.5430412677
H 8.1523103351 2.8034003138 -3.4574514799
H 6.2831884832 5.2356081475 -3.4017211553
H 6.1873914927 3.8542559548 -4.5111304366
H 4.6689330021 3.8689515781 -2.2192607521
H 5.4456408315 2.365402625 -2.7355302808
H 6.1902368631 7.3943148281 2.0964790826
H 8.1205270057 6.3762577759 0.9255825927
H 8.646409036 6.3648113733 2.6221307364
H 6.3025039719 6.1890322494 3.3926462692
H 8.5897633479 4.0057239981 1.2333188267
H 7.9942217334 4.0410676174 2.8985604418
H 2.7254842969 1.8218114458 5.020311147
H 0.9005104293 -1.6376556568 4.586690925
C 0.7212975098 1.151999618 5.5564748293
C 5.2603699796 -0.4671242949 4.9036998765
P -4.852727675 -2.1958289346 0.9699390844
N -5.9426266399 -2.353432651 -0.3107818317
N -5.499642036 -0.9489056281 1.9597883446
P -7.4731791012 -2.6569080009 -0.3392083595
P -4.9596699704 0.3140361754 2.6939203357
N -7.9663660725 -3.928717541 0.6467698234
N -7.8457009028 -2.9774897826 -1.9443020061
N -8.603462564 -1.4708868806 0.083110569
N -6.2000644407 1.0760958095 3.545695312
N -3.7503604712 -0.032511097 3.8390687518

N -4.3421225659 1.5514925901 1.748250454
C -6.4865938441 2.5193549804 3.5348231793
C -7.6903601616 2.6520702552 4.4832987926
C -7.4826980761 1.5035132358 5.4840433308
H -6.3093602116 -0.3288073905 5.1363499635
C -3.3810051138 0.9098072648 4.9167535638
C -2.9040995544 0.013679822 6.0754575721
H -2.3937490951 -2.1393162724 6.021986185
C -3.5034450769 -1.4282897832 4.2644909034
C -3.0840660344 2.2964985379 1.9317562275
C -4.9617173199 1.8384071117 0.4379178711
C -4.2630684774 3.1301502039 -0.0028915821
C -2.8459557199 2.9616837031 0.5660107574
C -6.9019028649 -2.818396113 -3.0648004301
C -9.1270305394 -3.123731465 -3.9268372288
C -7.6573997829 -3.4398884412 -4.2485346586
C -9.196802888 -3.3256920402 -2.4041086805
C -7.1157182778 -5.1442588801 0.6569359661
C -8.0080340275 -6.2120944256 1.3062952258
C -9.4195580185 -5.7972160865 0.8625579172
C -9.3651677713 -4.2687039228 0.9839238982
C -9.3825108164 0.7635328924 0.0974379735
C -8.9313635094 0.4150564666 1.5229680414
C -8.8402360717 -1.1190392606 1.5089380132
C -8.6004714213 -0.246005052 -0.7486020949
H -7.7786587521 -0.2122311841 4.1560283782
H -8.6218699998 2.4955464192 3.9256464171
H -8.3949638052 1.2266185184 6.0232165967
H -6.7227114811 1.7830749484 6.2256395865
H -7.7407710567 3.6388519424 4.9555621066
H -6.7192023781 2.878223739 2.5255150875
H -5.6291502229 3.1059774841 3.8988761677
H -4.2331299886 1.5379047329 5.2057569648
H -3.7417799127 -0.2149545133 6.7467887279
H -2.1169348292 0.4877965097 6.6711716072
H -1.4600779431 -1.1196712804 4.9074930713
H -2.263287538 1.627157781 2.2078689984
H -3.1665435441 -2.0476585518 3.4290103367
H -3.1904731359 3.0530113459 2.7251494789
H -2.2953190375 3.9035674211 0.656262131
H -2.2653360006 2.2911978745 -0.0741476046
H -4.2769766608 3.2579682428 -1.0903531252
H -4.7565696743 4.0004035533 0.4506402894
H -4.774778527 1.0183198519 -0.2666976084
H -6.0486376055 1.9561862326 0.530084334
H -7.9383049796 0.828003926 1.7198313531
H -9.614892913 0.7843089465 2.2959021812
H -9.7824577861 -1.5746894451 1.8427613073
H -10.4618152888 0.5974797189 -0.0181960604
H -8.0262998582 -1.4781428734 2.1447445154
H -9.1654992165 1.7992980553 -0.1869020803

H -7.5745735509 0.1127956763 -0.9242347214
H -9.0639247442 -0.4415431908 -1.7224198026
H -6.1877545866 -4.9608392858 1.2067389215
H -6.8457975944 -5.4387391857 -0.3691868448
H -9.9557987129 -2.6907629596 -1.9296998829
H -9.4394686184 -4.3721179426 -2.1575321203
H -9.8407462608 -3.7540453086 -4.4681178256
H -9.3459712503 -2.0764880208 -4.1732788739
H -7.5020404464 -4.526717237 -4.2638522199
H -7.328136519 -3.0375599665 -5.2125220289
H -5.9533201913 -3.3160344941 -2.848212925
H -6.6836622446 -1.7550450982 -3.2483380916
H -7.7325414077 -7.2264156371 0.9988248943
H -9.5849980099 -6.0857686279 -0.184268572
H -10.2180116326 -6.2404027807 1.4680136321
H -7.9285337204 -6.1569539538 2.3998973639
H -10.0739155561 -3.7549227974 0.3282520923
H -9.5864303373 -3.9600064881 2.0163443034
H -4.4143670355 -1.9005448101 4.6630401768
H -2.5769177098 1.5790413009 4.5831934025
C -2.4483898762 -1.2676436512 5.3610161657
C -6.9543247538 0.3709347596 4.5907807084

VbbH^+
C 0.0039288341 -0.0228179368 0.0213934282
N -0.0213000995 -0.0138455893 1.4980932878
C 1.3421650708 0.0616443157 2.0691248413
C 2.248239014 -0.1955561378 0.8551136983
C 1.4444875365 0.3842431441 -0.3195416589
P -1.427764767 0.2995414603 2.337538934
N -0.9757939271 0.3594490248 3.9305194676
C -0.7923043211 1.5744133482 4.7563051909
C -0.6432076367 1.0209193324 6.1813776254
C -0.0172575281 -0.3651567644 5.9639481975
C -0.7422959557 -0.8756188693 4.7129299186
N -2.5128862518 -0.8044860245 1.9804238459
P -3.9299854697 -1.2976551923 2.5916413976
N -4.1712121961 -2.8798306852 2.6403022145
P -3.7045252971 -4.1064617518 1.7429984745
N -2.077217955 -4.4060832999 1.5380391755
C -1.2339404318 -3.6534092265 0.5701382039
C 0.1117582942 -3.486899028 1.2909430053
C 0.1638340232 -4.6953005486 2.2370646992
C -1.2837548603 -4.7982706069 2.7286602681
C -4.2525924392 -0.6620935808 4.2565438634
C -4.0762629483 0.7382525586 4.4911101612
C -4.2161256485 1.1560657745 5.7826857731
C -4.5650020749 0.2734557378 6.8545155382
C -4.7618869951 -1.0717013815 6.6383623406
C -4.5879109667 -1.5268577508 5.2995416475
C -4.116036991 2.2970572885 6.7481208259

C	-4.4943248093	1.4347041155	7.8191655834
C	-4.6240746608	1.9160961953	9.1034741293
C	-4.363604601	3.3028254627	9.2791667906
C	-3.9748148559	4.1511528174	8.2321296624
C	-3.8216179193	3.622024453	6.917065449
P	-3.5116465027	5.9494024241	8.5139124296
N	-4.6001611866	6.8173546067	7.5164143657
P	-5.3736338355	6.5804964933	6.186458578
N	-6.4682688224	5.3116550679	6.1639074706
C	-6.6550147426	4.2885012477	5.1244937186
C	-7.4711758498	3.194239684	5.8319923299
C	-8.2791276567	3.9757350116	6.8796173613
C	-7.2762369629	5.0279259989	7.3690122756
N	-4.1049473263	6.0882512612	10.080150288
P	-4.370405793	7.3537192689	10.9676887987
N	-5.7262239372	8.3191026206	10.6905779621
C	-5.769003911	9.2729547971	9.5502104812
C	-7.1774457871	9.1080164016	8.959687324
C	-8.0118189842	8.623997373	10.1540056423
C	-7.0460802588	7.6702605566	10.8648208473
N	-3.1430111047	8.5005231614	10.9413826053
C	-1.7473462484	8.0047375864	11.0462287037
C	-0.9357651784	9.2594567915	11.3995733363
C	-1.9277028148	10.0894383161	12.2287126296
C	-3.2453455556	9.8796048645	11.472345162
N	-4.6238893894	6.7737393248	12.5173603266
C	-4.6698074029	5.347600754	12.8876338028
C	-4.6521317357	5.3819707385	14.4233434758
C	-5.3874091278	6.6910066492	14.752840692
C	-4.8751933869	7.6529794862	13.6696617135
N	-6.310453339	7.9153439398	5.7828995028
C	-7.729705417	7.8890493832	5.3882729246
C	-8.0722491845	9.3745624222	5.1829116487
C	-6.7355042943	9.9933963335	4.742986371
C	-5.7154962409	9.2508883323	5.6182271455
N	-4.3405875775	6.303468951	4.8567515572
C	-4.8019087955	6.460173946	3.4600336775
C	-3.5737005237	6.9857711245	2.6949295743
C	-2.3956935772	6.4671228135	3.5331258764
C	-2.9029079694	6.6475038246	4.9659776279
N	-4.254466275	-3.9718096006	0.1733176305
C	-5.6582254383	-3.5187622031	-0.0319976686
C	-5.9963420467	-3.985701995	-1.4560723815
C	-5.1527075152	-5.2590252333	-1.6162633131
C	-3.8364350792	-4.8693738518	-0.9352769811
N	-4.2991472136	-5.4384279984	2.5355901212
C	-4.9436676425	-5.4303620255	3.8691782518
C	-5.4749413948	-6.8638562082	4.0076568482
C	-4.4491854937	-7.6979856056	3.2243743477
C	-4.1374800212	-6.8095326051	2.0108489614
N	-1.9717658095	1.8231005285	1.8957407326

C	-1.0466426509	2.9724742361	1.7395059616
C	-1.7242954664	3.8685126895	0.6906500208
C	-3.2152935783	3.5513092415	0.8743801491
C	-3.2171950469	2.0391792707	1.1192702835
H	-4.7062389706	-2.5827753848	5.0765158649
H	-4.4784397292	3.7487837399	10.2628878979
H	-4.9184993933	1.2979226916	9.9481157335
H	-5.0214746355	-1.772992647	7.4263661753
H	-3.5261357199	4.2535730004	6.0866291789
H	-3.7986600355	1.4215592423	3.6978753326
H	-4.7268959533	9.1847788005	5.1478807661
H	-1.4589696026	7.0037645065	3.3496887577
H	-5.5861783743	9.7450787465	6.5908332906
H	-8.3919098198	9.8165482719	6.1339978756
H	-6.6954928238	11.0790948873	4.8773670119
H	-6.5498803678	9.7766980135	3.6827049672
H	-8.8785762968	9.5177595333	4.4565012287
H	-8.3569672504	7.4283912144	6.1596103014
H	-7.8782405683	7.3155836046	4.4605038209
H	-5.6528638741	7.1489584874	3.3972148839
H	-3.5703332744	8.082761847	2.6923337209
H	-3.5553828961	6.6508223247	1.6520311075
H	-2.2244672517	5.4012916839	3.3296864259
H	-5.6909314936	3.9138651973	4.7633351227
H	-2.3921503805	6.0046101983	5.6887943897
H	-7.2064011431	4.6956629359	4.2619687664
H	-8.0957152159	2.6257978042	5.1348663969
H	-6.7933930901	2.4947996464	6.3326118367
H	-8.650551535	3.3463990669	7.6946809618
H	-9.1405859109	4.4652071543	6.4069224522
H	-6.642715802	4.6389350464	8.1757928674
H	-7.7665512083	5.934449021	7.7433397656
H	-7.1502797201	8.3425054684	8.1786722237
H	-7.5540171107	10.0365932064	8.5168171699
H	-5.6152833198	10.2936944896	9.924788911
H	-8.2652038632	9.4647947682	10.8124684087
H	-4.9923891343	9.0447235846	8.8151055007
H	-8.944667734	8.1287786777	9.8628163535
H	-7.0661581625	6.677169782	10.3895316145
H	-7.2747242517	7.5417590463	11.9285145509
H	-1.4334190257	7.536410219	10.1085713966
H	-1.6614900618	7.2517828655	11.8449063388
H	-5.6022525566	8.4357222104	13.4210088144
H	-3.9479340989	8.1515650413	13.9937150568
H	-5.1949698399	7.0583515132	15.7660409616
H	-6.4706104938	6.5503591152	14.6438594513
H	-3.6169765614	5.4324945333	14.7852245624
H	-5.1218475008	4.4976773227	14.8665162291
H	-3.8216940254	4.8057637336	12.4606371251
H	-5.5903607024	4.8756570383	12.5113787294
H	-0.0118689166	9.0184340582	11.9352764884

H -2.0063971392 9.6786363458 13.2440873707
H -1.6532524252 11.1465136802 12.3104709179
H -0.6644473244 9.8036359117 10.4856679026
H -4.1317663909 10.0035948397 12.1004367071
H -3.3262988499 10.5924672029 10.6392888603
H -2.7552096964 7.68294486 5.3050217542
H -5.1322981511 5.492924168 3.0553312173
H -0.7402478719 0.6715088274 -0.3867500147
H -3.8291021247 3.8315473379 0.0128595097
H -0.2289339615 -1.0250451591 -0.3609926941
H 2.3889005307 -1.2742255587 0.7166231914
H 1.7675378456 0.0063321754 -1.294261938
H 1.533537835 1.4778967302 -0.3354664158
H 3.2354909998 0.2607008601 0.973846064
H 1.4855663345 -0.6839361149 2.8579489571
H 1.5384175084 1.050101358 2.5072962569
H -0.0529686813 2.636363534 1.4225860868
H -1.4008259303 3.5833071354 -0.3180780302
H -1.4849555266 4.9262615291 0.8353096312
H -3.6080296393 4.072167703 1.756059599
H -1.6497912638 2.2466933596 4.6610890208
H -4.1003307758 1.7107764662 1.6746375123
H 0.1110966836 2.1197888284 4.4482024
H -0.0384722922 1.6772569061 6.8136273676
H -1.6292332236 0.9219351242 6.6455023207
H -0.1485465368 -1.0352558294 6.8189170144
H 1.0579914294 -0.2724204027 5.7647017357
H -1.6990356124 -1.3463167349 4.9727553351
H -0.1509209559 -1.6030049074 4.1446823365
H 0.1028374704 -2.5532694225 1.8614531633
H 0.9532610263 -3.4501616015 0.5915232776
H -1.1207513284 -4.243989536 -0.3474271639
H 0.4391021782 -5.6029771145 1.6859189103
H -1.6899184161 -2.6932626784 0.317185605
H 0.8725287881 -4.5725990836 3.0622800483
H -1.4584301767 -4.10506311 3.5655378901
H -1.5565653698 -5.8055606559 3.0589369497
H -5.7351017218 -2.4336098673 0.0871539088
H -6.3283771739 -3.9872487835 0.7023718636
H -3.1254913345 -6.9670140627 1.619566735
H -4.847935979 -6.996211615 1.1921983003
H -4.819864001 -8.6854829281 2.9337657197
H -3.5421293705 -7.8406505533 3.8253194943
H -6.4632665988 -6.944355499 3.5378892219
H -5.5726273683 -7.1698022013 5.0537136981
H -5.7301981085 -4.6736445394 3.9166850312
H -4.2062911843 -5.2023812678 4.6520810499
H -7.0693460994 -4.1525855704 -1.5895937122
H -5.6215743286 -6.0947971221 -1.0809076881
H -5.0122550755 -5.5596794945 -2.6591846686
H -5.6789763269 -3.2315301264 -2.1869952422

H -3.2673304938 -5.7253824184 -0.5635514368
H -3.1914791537 -4.3140294564 -1.6291836648
H -3.1959957466 1.482047036 0.172289952
H -0.9328938602 3.5012396378 2.6936283699
H -4.9312611816 -0.7142773815 1.7790995345

VIaa
C 0.2954357704 -0.1412680861 0.1807559011
C 0.4341126804 -0.1621547225 1.5860001972
C 1.6806096941 -0.0909246928 2.247289088
C 2.8291713018 0.007919397 1.4121199284
C 2.7068065586 0.0428862093 0.0297993219
C 1.4254084105 -0.040202049 -0.5994615995
C 1.6390334718 -0.1259788914 3.6699365853
C 0.4322302811 -0.2351280393 4.3470470283
C -0.8083814715 -0.2960923041 3.6383061486
C -0.8019773418 -0.2609331659 2.2619953063
P 0.4970625588 -0.3943524911 6.2079571089
N 0.17167877 -2.0534747299 6.4130489242
P 0.5833955871 -3.457536577 5.906719437
N 0.1993881877 -4.0091098948 4.3445689045
C -1.2247539633 -4.1142579076 4.0126471417
P 4.2666062925 0.2968141838 -0.9682850774
N 4.1199145022 1.935693139 -1.4062623563
P 3.8039101147 3.3397467709 -0.8357468184
N 2.2471779477 3.7802402862 -0.3114269912
C 1.1805495912 3.7511627775 -1.3168749019
N -0.9985038518 0.3322364565 6.5647300316
P -1.781545399 0.3714831229 7.9119157676
N -0.9890778383 0.6070653593 9.4059615833
C -0.3245966598 1.9043783155 9.585453764
N -2.8128811779 1.7171566662 7.8574394572
C -3.1933926756 2.2913617571 6.5690569327
N -2.6176973519 -1.078000994 8.2320022055
C -3.1122284032 -1.8445371936 7.0872107633
C -3.7774989055 1.9614170845 8.922470577
N 2.2640449256 -3.7278323245 5.9291254125
C 3.0854001143 -2.98121231 6.8794811812
N -0.2729810125 -4.5898041351 6.8522436624
C -0.6502142468 -4.2269485428 8.2152011034
C -3.4146569555 -1.276769894 9.4395055381
C 2.8694752556 -4.9917003093 5.5185841254
C -0.0667515396 -6.0218498681 6.6677751811
C 0.9913090013 -3.5656870528 3.189041271
C -0.1618203734 -0.4943671866 9.9180553716
N 3.7855946829 -0.5187986266 -2.380579445
P 4.4558891062 -0.5476136761 -3.7872734795
N 6.1501203769 -0.6519555942 -3.9744299039
C 6.776594216 -1.8866446911 -3.4842720489
N 3.9408698927 -1.9658951402 -4.5630149028
C 2.7107237623 -2.625081059 -4.1308198425

N	4.1299386038	0.8512251023	-4.7030237627
C	2.8492694889	1.5255282509	-4.4850240876
C	4.2938667732	-2.2250383038	-5.9532861849
N	4.7227031069	3.7412292715	0.5398872723
C	6.0258005127	3.1072814335	0.7275817399
N	4.0220797907	4.4480057008	-2.1140348006
C	4.9785303755	4.1258462685	-3.1689625335
C	4.6597455676	1.0534395832	-6.0487297364
C	4.6078583399	5.0267518705	1.2231605552
C	3.8607110849	5.8805848857	-1.8928608203
C	1.7592570146	3.347080357	1.005014273
C	6.947613907	0.53062453	-3.6204253462
C	-1.1845328655	-0.2331453815	-0.1614375454
C	-1.9165908268	-0.3180987053	1.2274907668
H	1.3807439698	-0.031798142	-1.6860901945
H	-1.7301840595	-0.3583801412	4.2120564205
H	2.570542289	-0.0744620234	4.2337143672
H	3.8180151695	0.0656983035	1.8671927319
H	4.0758000481	-2.8031705703	6.4389090256
H	2.6305848229	-2.0148430492	7.1020151997
H	3.2266032475	-3.5348478748	7.8235244739
H	0.0012782078	-0.3421283773	10.9929161645
H	0.816911099	-0.5405344544	9.4169916185
H	-0.6642776444	-1.4504159654	9.77239752
H	0.6386157911	1.9491684008	9.053535817
H	-0.1400039724	2.0611768287	10.6558524596
H	-0.9632470911	2.7095743984	9.217951701
H	3.0397557111	-5.6648467747	6.3748404192
H	3.841830857	-4.7949164888	5.0456387938
H	2.236728354	-5.5044842398	4.7926912625
H	-2.4075448354	2.098345105	5.8382613791
H	2.0206844286	-3.3580073822	3.4803383867
H	-4.1421434075	1.8695294431	6.1959382837
H	-3.3236132074	3.3765996233	6.6824606803
H	0.9912360842	-4.3605971835	2.4308126277
H	-4.7471254901	1.4706952883	8.7340278694
H	-3.3859083294	1.6111850175	9.8798671582
H	-3.958745093	3.0417068896	9.0052588724
H	-2.4039053349	-1.7746240527	6.2635300991
H	-1.6400828885	-3.143206789	3.7016629317
H	-4.1068701309	-1.4980597422	6.7574826044
H	-1.345500652	-4.8242528591	3.1838695823
H	-3.1948961971	-2.9001459362	7.3749940192
H	-1.7896349163	-4.4794686788	4.8724273685
H	-4.4685566592	-0.9859809434	9.2975951113
H	-3.3945025587	-2.3408525635	9.7138558834
H	-3.0027599935	-0.7020680941	10.2708594302
H	-0.8639685066	-3.1592939541	8.2602558567
H	-0.9745006168	-6.5598348915	6.9735198874
H	-1.5530698661	-4.7860447301	8.4984887629
H	0.1413132285	-4.4688291269	8.9460450689

H 0.7725461503 -6.4096160688 7.2697271183
H 0.1223895146 -6.2465196872 5.616130727
H 0.5749952157 -2.65628458 2.7415049452
H 6.2159600374 2.9838420894 1.8024471726
H 6.0428163877 2.1212925834 0.2609020418
H 6.8430573552 3.7160300587 0.3043354523
H 7.9398049105 0.4361711413 -4.0804492445
H 7.0731751143 0.6317814569 -2.5317757858
H 6.4743242897 1.4381400289 -3.9947658696
H 6.885758846 -1.8845971841 -2.3884839877
H 7.7732351008 -1.9793903587 -3.9342787213
H 6.1791784165 -2.7531741134 -3.7731732098
H 5.3568313662 5.7523125424 0.8654874892
H 4.7652433933 4.8832033792 2.3013467613
H 3.6138385904 5.4528297459 1.0793808889
H 2.5376423135 -2.4164149406 -3.0747080247
H 2.5842716757 3.2372460146 1.7084152451
H 1.8366088706 -2.2850804868 -4.7119294239
H 2.8141931236 -3.7097506465 -4.2731309913
H 1.0614338873 4.101897242 1.3924547107
H 3.5545501889 -1.8126568887 -6.6603374339
H 5.2744264084 -1.8027678313 -6.1831460488
H 4.3453586485 -3.310072326 -6.1167868457
H 2.5715776592 1.4584991246 -3.4346354449
H 0.7788710547 2.7347404405 -1.450748718
H 2.0475265343 1.1001506645 -5.113002516
H 0.3631637987 4.4062771754 -0.9879035912
H 2.9579090979 2.5872493941 -4.7399259142
H 1.5539801007 4.1114788312 -2.277287778
H 3.9727597725 0.6846310872 -6.8280480014
H 4.8129988075 2.1284862346 -6.2184857469
H 5.6208558659 0.5492915698 -6.1634173595
H 4.9864264771 3.0487343838 -3.3339626565
H 3.5711517184 6.3615289876 -2.8371798072
H 4.6721131678 4.6290373144 -4.0967909649
H 6.0011127054 4.4618857455 -2.9228750558
H 4.7891184721 6.3618810815 -1.5413611476
H 3.0709072561 6.0656524599 -1.1620255698
H 1.2356263313 2.3866143886 0.9433375304
H -1.5154785069 0.642264091 -0.734853559
H -2.4959484803 -1.2459236959 1.3177924998
H -1.3980011056 -1.1118477927 -0.7828510374
H -2.6274080493 0.507363318 1.3585755231

VIaaH^{+}
C 0.0399418028 -0.618336715 0.041371745
C 0.2536433297 -0.5292940998 1.4324663212
C 1.5118065617 -0.2644115903 2.0232764429
C 2.5924912764 -0.0728749864 1.1250310573
C 2.3860671938 -0.144183573 -0.2497552141
C 1.1046778244 -0.4253747132 -0.8150909039

C	1.5474255295	-0.1956878319	3.4439408169
C	0.3937341551	-0.3795721315	4.1896685685
C	-0.8625085603	-0.6347563817	3.5536267338
C	-0.9299311529	-0.716543085	2.1821436261
P	0.5356333995	-0.3918846222	6.0624397493
N	0.2241409352	-2.0323254464	6.3609836588
P	0.7481030774	-3.4626876834	6.071163535
N	0.4556209873	-4.2071148451	4.5674444912
C	-0.9458430667	-4.4545299333	4.2141460888
P	3.7988542095	0.2307064167	-1.3112157501
N	4.3554588794	1.7267516142	-1.0979949146
P	3.7468444713	3.1194103801	-0.6356629847
N	2.0980899512	3.3735983139	-0.5249408239
C	1.2793512608	3.4411039079	-1.7405473539
N	-0.9452291495	0.3555583824	6.3958227187
P	-1.6413144721	0.544894043	7.7864587706
N	-0.7456966517	0.8943293975	9.1896041494
C	-0.051204796	2.1876001125	9.2239681232
N	-2.6445485049	1.9016872802	7.6638791852
C	-3.1040398424	2.3637430738	6.3561146795
N	-2.4731473102	-0.8538991718	8.2818628299
C	-3.0431162165	-1.7120225919	7.2400179336
C	-3.5091273323	2.3023015563	8.7699879476
N	2.4438222071	-3.6119283549	6.167305597
C	3.1476218406	-2.7607440468	7.1291063341
N	-0.0697554374	-4.5176267807	7.1178063164
C	-0.5367062689	-4.0235813782	8.411889932
C	-3.2301010916	-0.8978401534	9.5334825054
C	3.1145948585	-4.895984816	5.957965633
C	0.1825579371	-5.9561078878	7.0978959761
C	1.2451774554	-3.8071621116	3.397591383
C	0.0672408967	-0.1748297915	9.7847871373
N	3.4023462631	-0.2148477871	-2.8107538339
P	4.3048074267	-0.2645079296	-4.1211517739
N	5.8972344099	-0.7882321237	-4.012531672
C	6.1799082389	-2.2218935222	-3.860789026
N	3.5770694461	-1.3619164618	-5.1483301823
C	2.2340178205	-1.9001321076	-4.9183970195
N	4.4825530464	1.2812619504	-4.7695822571
C	3.3213864427	2.1774218529	-4.7284206192
C	4.0554633087	-1.4926602127	-6.5263666629
N	4.2958577962	3.4457748774	0.9134069056
C	5.4046650834	2.7121163844	1.5258363711
N	4.2035212024	4.2503898003	-1.8028556425
C	5.4549085627	4.0506397536	-2.541032726
C	5.3687684445	1.5597655258	-5.9052577789
C	3.9724594756	4.6984831558	1.6015535454
C	3.8687464995	5.6662658869	-1.6132248334
C	1.3141467246	3.0094459775	0.6643098663
C	6.9084517473	0.0576287466	-3.3618665687
C	-1.4291397392	-0.9180737407	-0.2091709996

C	-2.0777425064	-0.9806877231	1.220649449
H	0.9997590736	-0.4801810533	-1.8947099711
H	-1.7409818552	-0.7513769234	4.1834813016
H	2.492208926	-0.0032491055	3.9518254529
H	3.5796378657	0.1567663971	1.5240676126
H	4.1813358982	-2.6176522488	6.7884194114
H	2.6708362891	-1.7816345225	7.1953970772
H	3.1809306762	-3.2122757293	8.1348042198
H	0.2672874633	0.0762504119	10.833982677
H	1.0294592975	-0.2949883197	9.2640276161
H	-0.4635752184	-1.1257399535	9.7475696088
H	0.8971357917	2.1565367149	8.665164745
H	0.1673057743	2.44434842	10.2678440088
H	-0.683142195	2.9679493214	8.7965572388
H	3.2089831097	-5.4720407693	6.8921903721
H	4.1261548205	-4.7158941885	5.5698140236
H	2.5711559322	-5.5002479686	5.2298337645
H	-2.38556865	2.0668556813	5.5915873486
H	2.2582666701	-3.532511815	3.6930307773
H	-4.0938847345	1.9503148843	6.1006696333
H	-3.1855943473	3.4592878047	6.3692144114
H	1.2973149557	-4.6512701799	2.6967640743
H	-4.5115882253	1.850117331	8.7027259995
H	-3.0605213667	2.025520538	9.7259390727
H	-3.6299980013	3.3935738082	8.7559188198
H	-2.3487364209	-1.7870809471	6.404577127
H	-1.4247900916	-3.553711255	3.7989275212
H	-4.0201948115	-1.3423479825	6.8857814651
H	-0.9881385557	-5.2501258707	3.4593133382
H	-3.1890386285	-2.7172119207	7.6538509231
H	-1.5090778874	-4.7750959943	5.0920635399
H	-4.2752895322	-0.5764455677	9.4006964631
H	-3.2401107152	-1.9290386082	9.9107247981
H	-2.7619431199	-0.2637549759	10.2881730556
H	-0.7723895158	-2.9624531444	8.3353626536
H	-0.7323711968	-6.4870787196	7.3926875133
H	-1.4447539419	-4.5711292011	8.6986103132
H	0.2145419386	-4.1727779642	9.205472391
H	0.9823998521	-6.250121829	7.7964178491
H	0.4555572444	-6.283077961	6.0928815943
H	0.794443671	-2.951231587	2.8774482491
H	5.1400346083	2.4447630783	2.557185733
H	5.60921146	1.8015787593	0.9632686964
H	6.3178785966	3.3240581641	1.5500159219
H	7.8772897043	-0.0994714799	-3.8506066952
H	7.0134259485	-0.1919920856	-2.2963954689
H	6.6413137613	1.1107993764	-3.4428784897
H	6.1902574164	-2.522274933	-2.8025908147
H	7.1652609506	-2.4350179233	-4.2907258703
H	5.4321758021	-2.8166161832	-4.3863868147
H	4.8108461249	5.4080201364	1.5577257861

H 3.7536434345 4.4880215358 2.6558925761
H 3.0909536379 5.1679131141 1.1629200147
H 1.9761818618 -1.8159685916 -3.8633330206
H 1.9619996746 2.8322270834 1.5210208973
H 1.4816762558 -1.3663317426 -5.5173649191
H 2.2186648831 -2.9587675074 -5.2057272899
H 0.6238910972 3.8284160435 0.9025811331
H 3.4710844626 -0.872484053 -7.2216665088
H 5.1075872186 -1.2090356889 -6.5981516753
H 3.967013033 -2.5390090344 -6.8419111703
H 2.6981582685 1.9446927274 -3.8667203832
H 0.8710295595 2.4526367568 -1.9965818361
H 2.7150854217 2.0947313976 -5.6435053604
H 0.4414019272 4.1271637488 -1.5688173821
H 3.6690994675 3.2116024816 -4.6329205719
H 1.8681554039 3.8146571837 -2.5786367899
H 4.8403228262 1.473841022 -6.8659994394
H 5.7482797757 2.5848856253 -5.8148133698
H 6.2197617673 0.8778077364 -5.9101646229
H 5.5715443316 2.9964376509 -2.7878362546
H 3.8197924378 6.152851857 -2.5942370343
H 5.4065648581 4.6284782731 -3.4715562633
H 6.3337841881 4.3928364208 -1.972617472
H 4.619723605 6.1938555479 -1.0062195298
H 2.8924460686 5.7686644544 -1.1359881931
H 0.7339770165 2.099103216 0.4812801695
H -1.8941629219 -0.1422466602 -0.829535932
H -2.5364187988 -1.9588643991 1.4083736598
H -1.5523658183 -1.8647548483 -0.7488986813
H -2.8737987366 -0.2353037424 1.3333614968
H 4.8468558193 -0.5906559215 -0.8438745359

VIbb
C -0.0837292033 -0.2839297756 -0.399300461
N -0.0574535751 0.0873598499 1.0198467067
C 1.3062361417 0.3974283054 1.4778219473
C 2.1855198844 0.079446776 0.2526136516
C 1.2352433272 0.2833487781 -0.9391634095
P -1.4758140833 0.3381912763 1.9015483747
N -0.9051658448 0.9154674456 3.3663770147
C -0.8560428059 2.3199613156 3.8091015715
C -0.3968494906 2.2353610375 5.2770336039
C 0.3757451682 0.9084290326 5.3431664296
C -0.4411849475 -0.0041280662 4.4216499899
N -2.3266729261 -0.9696454857 1.9214984236
P -3.9276854599 -1.3460187211 2.4455210541
N -3.8993887011 -3.0088869917 2.7195240237
P -3.3393040819 -4.2293041109 1.9302429915
N -1.6759105131 -4.524465218 1.8106617107
C -0.8268964352 -3.7169854855 0.8974367141
C 0.3762825003 -3.2984533788 1.7519479048

C	0.49558335	-4.4408758817	2.770782578
C	-0.9715412907	-4.7491310006	3.0953713121
C	-3.8700824956	-0.77238909	4.2314164681
C	-3.9643439148	0.595935404	4.4612495495
C	-3.8446034355	1.1292118091	5.7708777098
C	-3.6642371315	0.1933248568	6.812191246
C	-3.5983168762	-1.1980777942	6.6035088153
C	-3.6974381301	-1.6854703326	5.3167739333
C	-3.8973622892	2.4986866136	6.1561788336
C	-3.8166103554	2.8793862865	7.4866306321
C	-3.6018839737	1.8933675742	8.5076402636
C	-3.534195928	0.5615092665	8.1693271533
P	-3.9156395365	4.6772763787	8.0189124497
N	-4.657782279	5.4120627801	6.7091125689
P	-6.017794561	5.3723188436	5.9337026186
N	-7.2711362516	4.268206102	6.1723074251
C	-7.2069848677	2.8717627107	5.6841262805
C	-7.4115120122	2.0237988857	6.9406436366
C	-8.4409925184	2.8559021933	7.7199762435
C	-7.9848993244	4.3068586677	7.4788231856
N	-5.1238194618	4.4725143513	9.2147989169
P	-5.2337069123	4.7638664541	10.7393646594
N	-6.4035435932	5.9078078075	11.1891275071
C	-6.2877129714	7.2536985548	10.5768850555
C	-7.6210034197	7.4631906889	9.8497591738
C	-8.6101504231	6.7254314146	10.7642066618
C	-7.8231510811	5.4716573517	11.1830546663
N	-3.8760758704	5.3544606858	11.5339230418
C	-2.587107344	4.644707501	11.3927177097
C	-1.6479297393	5.4222756382	12.3231279527
C	-2.5820876195	5.8571801732	13.4631367032
C	-3.8812838199	6.2255238709	12.7295456245
N	-5.7017218922	3.3673117027	11.5481532555
C	-6.29315294	2.1782831881	10.9261371197
C	-5.9401963541	1.0750769979	11.9278594455
C	-6.1252741062	1.787663138	13.2806126584
C	-5.6475101241	3.234541521	13.0118833571
N	-6.8474581803	6.8128263295	6.2460955289
C	-8.063772685	7.1972859228	5.5234832682
C	-8.5040530817	8.4585607385	6.2734299878
C	-7.1649251903	9.1671191891	6.5707907059
C	-6.1057590286	8.0329514698	6.6267716918
N	-5.6841528172	5.1715584769	4.2955871366
C	-6.6327440161	4.9657624837	3.2035935507
C	-5.8522368345	4.0372970477	2.253963327
C	-4.3697557626	4.4604174472	2.4452880249
C	-4.3650789369	5.3827516035	3.6930629412
N	-3.7832831205	-4.2852328777	0.3096746017
C	-5.1711712389	-3.8994180394	-0.0367521605
C	-5.3251357386	-4.3605803907	-1.4936696214
C	-4.421211581	-5.6018887683	-1.559170282

C -3.2086666416 -5.1706263644 -0.7235055165
N -3.9014485454 -5.5915666527 2.7361488207
C -4.5441148016 -5.5688398651 4.0602139888
C -5.0835349133 -6.997993416 4.2043516391
C -4.0206437219 -7.8387330263 3.477619277
C -3.644871611 -6.9610737305 2.2700087696
N -2.2491656168 1.6739541411 1.1852390808
C -1.4963618096 2.816880978 0.6405551655
C -2.0657205654 3.0134178919 -0.7711851972
C -3.5445882049 2.6336940904 -0.5913767253
C -3.520750558 1.4887511434 0.4411290783
C -3.4041607485 -1.8798411543 7.9510161532
C -3.362422713 -0.7046785939 8.9965766536
H -3.6394191793 -2.7459752879 5.0865406336
H -3.5266748678 2.2150058716 9.5445555134
H -4.0361756855 3.253636274 5.3898851478
H -4.1002250861 1.2802657025 3.6233487677
H -5.2737050939 8.2365979948 5.9382674776
H -3.9841761503 4.997902803 1.5723874075
H -5.6704097518 7.8977760514 7.6192338385
H -9.0026060929 8.1700278405 7.2059567631
H -7.1976708913 9.74239283 7.5019826421
H -6.9226716365 9.8690559938 5.7643629206
H -9.1972078726 9.0783851093 5.6939714792
H -8.7992226057 6.3882251537 5.5504406502
H -7.858255524 7.4321877533 4.4634427387
H -6.8677185008 5.9200976381 2.6998583063
H -6.1913162059 4.1215942995 1.215497799
H -5.9927812113 2.9967292245 2.5662735583
H -3.7315171058 3.5843610245 2.5914972687
H -6.2645565962 2.6669673955 5.1776861404
H -3.5788463756 5.156341585 4.4145823476
H -8.0226597794 2.6966612994 4.9647391202
H -7.7504568629 1.0073507891 6.7131606639
H -6.4675524277 1.9629735449 7.4927943333
H -8.4719915877 2.6165253834 8.7879666909
H -9.4465777412 2.6922022262 7.3099105199
H -7.29542523 4.622185531 8.2668501643
H -8.8267812417 5.0095826776 7.4401996172
H -7.5809982441 6.9839540096 8.8659577557
H -7.8666078354 8.5215836324 9.7078036583
H -6.1415622118 8.0077566743 11.364130621
H -8.8451238542 7.3398415955 11.6431677885
H -5.426576792 7.2987901756 9.901510258
H -9.5537537006 6.4703570016 10.2696776911
H -7.9915178723 4.6627520667 10.4604227164
H -8.1159265953 5.0995584437 12.1723247757
H -2.2572898454 4.6488384087 10.3500563721
H -2.6783486543 3.5946128315 11.7197268178
H -6.2869709941 3.973305201 13.5175883533
H -4.6201873144 3.396466099 13.3655572675

H	-5.5750116792	1.3151792743	14.1011192699
H	-7.1882311885	1.7898160831	13.5526102876
H	-4.8936860814	0.7750723002	11.7884057429
H	-6.5697660713	0.1848672022	11.8223693941
H	-5.884356993	2.0284992492	9.9264277975
H	-7.3895836384	2.272707121	10.8370964319
H	-0.7990184188	4.8183337872	12.6605181376
H	-2.7605840906	5.0125350904	14.141976113
H	-2.1891015688	6.6888089894	14.0579340306
H	-1.253154275	6.3027708282	11.8003928579
H	-4.7743295821	6.0680617143	13.3416464395
H	-3.8730550697	7.2821485757	12.4250500804
H	-4.2426386209	6.4357403222	3.3893075573
H	-7.5694415576	4.537771364	3.5658123199
H	-0.9674987167	0.1332831604	-0.8944071663
H	-4.0265887702	2.3278400816	-1.5260515646
H	-0.1190319122	-1.375266764	-0.5287835442
H	2.5109252663	-0.9679350337	0.2890580869
H	1.5727734939	-0.2172433802	-1.8531884833
H	1.122292456	1.3535243392	-1.1577318024
H	3.0816506699	0.7075011299	0.2108095425
H	1.5928968627	-0.2070352495	2.3471365306
H	1.3996465412	1.4530290558	1.7720199754
H	-0.4224123333	2.60681258	0.6365869897
H	-1.5745529089	2.325552185	-1.4707397287
H	-1.9247012277	4.0332829327	-1.1466235393
H	-4.103336041	3.4889105741	-0.1968533668
H	-1.8363528022	2.7994015044	3.7149871057
H	-4.3833728431	1.5281341564	1.1148299287
H	-0.1406416028	2.8980889891	3.204289337
H	0.204009375	3.1030548703	5.56905269
H	-1.2681304261	2.1941987741	5.9365643391
H	0.4539009847	0.5099831868	6.360364356
H	1.3915321848	1.0337926399	4.9442097733
H	-1.2920006626	-0.4491534227	4.9502254841
H	0.1532883657	-0.8236848057	4.0018955496
H	0.1365966886	-2.3583587917	2.2563138103
H	1.2842163959	-3.1495183749	1.156330224
H	-0.5110416293	-4.3393944503	0.047545247
H	0.9748922553	-5.3162664148	2.3123716504
H	-1.3761185016	-2.8508111692	0.5232049722
H	1.0681313896	-4.1708318275	3.6651030148
H	-1.3419303485	-4.0641265554	3.8732180062
H	-1.1264477495	-5.7768657185	3.4451899118
H	-5.3185198366	-2.8229225635	0.0949040622
H	-5.8941183934	-4.4172014141	0.6125601777
H	-2.5974215073	-7.0931404065	1.9679679642
H	-4.2730155483	-7.2001393584	1.3970940804
H	-4.3734052	-8.8322546467	3.1806982747
H	-3.1455894223	-7.9725683674	4.1269580908
H	-6.0503609722	-7.0862377785	3.6915446267

H -5.2256240275 -7.2902332888 5.2504179202
H -5.3177431525 -4.7987748948 4.100528089
H -3.8124350337 -5.3466688211 4.8542160521
H -6.3683706405 -4.5639497321 -1.7574160997
H -4.9208038065 -6.4561664751 -1.082665117
H -4.1487080219 -5.8929631952 -2.5797243037
H -4.9482622908 -3.5876618017 -2.1763823052
H -2.6663922265 -6.0103565107 -0.2795788663
H -2.4949975019 -4.6097718313 -1.3453983056
H -3.5414707056 0.5057027092 -0.0419074991
H -1.6587930478 3.7161155564 1.2529132833
H -2.4164744034 -0.6957808044 9.5530236697
H -4.1618682675 -0.8038455664 9.7420509112
H -4.2215116248 -2.579311071 8.1676103749
H -2.4772485548 -2.4671687566 7.9727514054

VIbbH^+
C -0.0318312672 -0.0162757369 -0.1175219143
N 0.0015983789 -0.0054810294 1.3523209708
C 1.3793222865 -0.0117842309 1.8744696866
C 2.2409833853 -0.2221734627 0.6166565676
C 1.3943304615 0.3943971135 -0.5089334522
P -1.4028875198 0.1202785973 2.2640344709
N -0.8357809163 0.2212650125 3.8392075165
C -0.6548315144 1.4320246231 4.6525757091
C -0.4305752041 0.8905708299 6.0756242249
C 0.1896050896 -0.4954328066 5.8378037688
C -0.5794215905 -1.0082507838 4.6141407043
N -2.3848214242 -1.0481658318 1.9552011045
P -3.9962004332 -1.4568715407 2.3821480446
N -3.9963155658 -3.1265767695 2.5791974286
P -3.597191487 -4.2786334701 1.5939294902
N -1.9741801675 -4.5918219152 1.2549789425
C -1.2011511659 -3.7349124711 0.3176387603
C 0.1454548432 -3.5097161309 1.0199630953
C 0.2999663405 -4.7580981127 1.9000793504
C -1.128787545 -4.9826811757 2.4069589026
C -4.071639859 -0.9970262558 4.2097637148
C -3.9643121189 0.3457118394 4.5531965822
C -4.0445711057 0.7629003838 5.9036808771
C -4.26785283 -0.2412606334 6.8723555266
C -4.393449448 -1.6064003905 6.5452236334
C -4.2933021722 -1.9848108939 5.2223369414
C -3.9483699009 2.0906020383 6.3911599203
C -4.0917047399 2.3584398521 7.746883167
C -4.3079161883 1.3154134614 8.7050724735
C -4.3897999312 0.0148712788 8.2539452503
P -4.1027864271 4.0939727718 8.2737300539
N -5.1938442462 4.999646049 7.488412761
P -5.964611341 4.9039325967 6.1016972417
N -6.7512269115 3.4727422065 5.8286578507

C	-6.8028231636	2.7314966956	4.5480558246
C	-7.4539647728	1.3937541351	4.9321757157
C	-8.3426496461	1.7558559768	6.1322450971
C	-7.4863322004	2.7685984483	6.9023860086
N	-4.2187059232	4.0983074274	9.870869083
P	-4.7628828639	5.1345933886	10.9458215747
N	-6.3332280404	5.6865472819	10.8430420336
C	-6.7492175041	6.7735343542	9.9163178047
C	-8.122076081	6.3233001539	9.3981009474
C	-8.6708918348	5.4615611712	10.5445908702
C	-7.4277159343	4.6995411053	11.0165841773
N	-3.8761758546	6.5477053643	10.9506140589
C	-2.3957100239	6.4380197295	10.8402612031
C	-1.8828277008	7.7882067102	11.3640610837
C	-2.942565878	8.1855308106	12.4024649511
C	-4.2477277194	7.7640723681	11.7188295492
N	-4.6818594	4.3070236187	12.3832796021
C	-4.3743329849	2.8659520601	12.5300579563
C	-4.2246159085	2.6965773156	14.0478725819
C	-5.2161610098	3.7242387527	14.6156475127
C	-5.0458009782	4.9261538424	13.6738702723
N	-7.1538744533	6.0711507928	6.047116568
C	-8.5542252813	5.8424789492	5.626979487
C	-9.1734519909	7.248509815	5.6759520517
C	-7.9887074506	8.1739856131	5.3562186013
C	-6.8291575953	7.5093202375	6.1108318737
N	-4.917642955	5.1754145533	4.8210762465
C	-5.4054043375	5.5669369781	3.4791358256
C	-4.3480583146	6.5551457137	2.9700453453
C	-3.0565843575	6.0315952664	3.6152309958
C	-3.5050540533	5.5886931254	5.0156273776
N	-4.229847085	-4.1279986804	0.0453816567
C	-5.6517371421	-3.7182072671	-0.0722240131
C	-6.0139635981	-4.058254884	-1.525209952
C	-5.1430206471	-5.2895154893	-1.8182751734
C	-3.8199564794	-4.927943356	-1.1318751717
N	-4.117778762	-5.6938039615	2.3230465273
C	-4.6909751669	-5.7816276311	3.6775061931
C	-5.2148846913	-7.2237076411	3.7436996817
C	-4.2220638994	-7.995537867	2.859758964
C	-3.9602704047	-7.0185470639	1.7023134677
N	-2.0465224499	1.6639074211	1.9260257113
C	-1.1768672744	2.8426729203	1.7305487605
C	-1.8107614852	3.6085902478	0.5586552655
C	-3.2993814771	3.2525083558	0.6851379986
C	-3.2698585709	1.7750288298	1.0925342033
C	-4.6202068476	-2.3907138665	7.8291359358
C	-4.6071935659	-1.3090048319	8.9710942817
H	-4.362302362	-3.0216257188	4.9049430581
H	-4.4051092571	1.5762228987	9.7540823365
H	-3.7956800298	2.8913738424	5.6741784546

H	-3.7889643664	1.0980009209	3.7884434228
H	-5.8534131292	7.7092038377	5.6524789235
H	-2.2583772337	6.7788788446	3.6585470776
H	-6.7854555068	7.8504479889	7.1535945871
H	-9.5480661969	7.4561322008	6.6855181701
H	-8.1525012514	9.2105025337	5.6662438907
H	-7.7864757843	8.171859986	4.2775614441
H	-10.009395897	7.3549929742	4.9782219258
H	-9.0644628735	5.1433662291	6.2976862552
H	-8.6013237361	5.4229559071	4.6126021058
H	-6.4039587843	6.0118041094	3.5438792515
H	-4.5718915343	7.5662814284	3.3325967251
H	-4.3015144487	6.5898830152	1.8771932942
H	-2.6793926307	5.1696371181	3.054167984
H	-5.7999414154	2.5977061137	4.1329503583
H	-2.8921103201	4.7634858092	5.3930653825
H	-7.4123583592	3.2728876449	3.8102665823
H	-8.0089109288	0.9555446739	4.0976730589
H	-6.6807297838	0.6805740466	5.2329537305
H	-8.6085882368	0.8899332066	6.74625897
H	-9.2716383176	2.2310602055	5.7920513796
H	-6.7819939442	2.264896978	7.576395938
H	-8.08626524	3.4651055534	7.4996668372
H	-7.986223367	5.7198628837	8.4956483956
H	-8.7684069188	7.1708023949	9.1476994852
H	-6.8266494757	7.7152130947	10.4742776311
H	-9.0524947675	6.0970690221	11.3532842409
H	-6.0228629705	6.8990560725	9.1103799495
H	-9.4759312211	4.7869328024	10.2362645721
H	-7.2589288133	3.8100411797	10.3909216691
H	-7.4916809927	4.3745853471	12.0600684974
H	-2.0992034068	6.2440062445	9.8046620517
H	-2.0205139009	5.6107952169	11.4591122169
H	-5.9602557349	5.5243046126	13.5826253203
H	-4.2420786076	5.5908293377	14.0230064261
H	-5.0255997595	3.9839574786	15.6612475585
H	-6.2405228692	3.3370156012	14.5450744781
H	-3.2019124863	2.9500494085	14.3542331571
H	-4.4305358621	1.6721110607	14.3728039515
H	-3.4716700078	2.6025994441	11.9742029112
H	-5.2012678906	2.2530886539	12.1430315261
H	-0.872825986	7.7116484658	11.7776994809
H	-2.7998481026	7.6131258238	13.3281947632
H	-2.9264043142	9.2502204929	12.6554614209
H	-1.8594893052	8.5252946877	10.5516537622
H	-5.0615628741	7.5615144552	12.4199012909
H	-4.5902638835	8.5429852175	11.0245600859
H	-3.4368347553	6.4107375695	5.7397300863
H	-5.4717155997	4.6876961095	2.8252849311
H	-0.7911420641	0.6788571687	-0.4957428109
H	-3.8673131147	3.4169295456	-0.2363250603

H -0.2786820678 -1.0165763148 -0.4997173919
H 2.3760238791 -1.2948909348 0.434471854
H 1.6771078874 0.042104396 -1.5060855444
H 1.4871050396 1.4883769652 -0.4968469744
H 3.2330846407 0.2302644577 0.7132411994
H 1.5333343994 -0.809110812 2.6105543559
H 1.622009182 0.9412289075 2.3683086544
H -0.1450402302 2.5388359444 1.5209621324
H -1.4166306731 3.2328745352 -0.3937540239
H -1.6112423237 4.6850192111 0.6017463489
H -3.7646393861 3.8522807871 1.4779633007
H -1.5368270846 2.0804501422 4.5939312922
H -4.1616131257 1.4718630196 1.6505435277
H 0.2146685139 2.0149088185 4.3097267573
H 0.2030161454 1.552651341 6.6754258315
H -1.3931155946 0.7860631197 6.5871852823
H 0.0936600962 -1.1637084748 6.6998002946
H 1.2565500634 -0.3984822943 5.5988294426
H -1.5233303472 -1.4887296424 4.9015996284
H -0.0052636643 -1.7365078671 4.0298082825
H 0.076913429 -2.6116714667 1.6407211776
H 0.9687193911 -3.3765308765 0.3095861539
H -1.0658388072 -4.2674334785 -0.6334275189
H 0.6266814116 -5.6153770257 1.2975038593
H -1.7210986909 -2.792880834 0.1254238356
H 1.0149263324 -4.6287417697 2.7200208781
H -1.331918848 -4.3438166601 3.2804127956
H -1.3242596344 -6.0222793896 2.6929857976
H -5.7675407436 -2.6564383229 0.1656037642
H -6.2806026734 -4.2908251189 0.6268989071
H -2.9599380613 -7.1378329277 1.2682242132
H -4.6933156711 -7.161763951 0.8929302235
H -4.6024054923 -8.9625204265 2.5151261601
H -3.2910086015 -8.176221298 3.4124267812
H -6.2223796364 -7.276458712 3.3110191627
H -5.2672336091 -7.6032404137 4.7695607061
H -5.4736933167 -5.0315969505 3.8186360908
H -3.9187076659 -5.6062778283 4.4429260953
H -7.0859206612 -4.2399354942 -1.6538758962
H -5.5799917418 -6.180639213 -1.3482786968
H -5.0203524411 -5.4948987594 -2.8869800963
H -5.7291208038 -3.2317128513 -2.1890796633
H -3.2295321201 -5.8005971569 -0.8394526696
H -3.197142297 -4.3145916938 -1.7986882329
H -3.2148256112 1.1222309227 0.2112172293
H -1.1578641907 3.4618169324 2.6397076459
H -3.8072811237 -1.5011202949 9.6965196795
H -5.547430124 -1.3080082453 9.5358623793
H -5.5731382443 -2.9328047276 7.8028762833
H -3.8373718198 -3.1432034849 7.982074459
H -2.826851807 4.5920536304 7.9172377776

VIaa

C 0.11997003 0.0218196352 0.1097058661
C 0.0180401064 0.0097602627 1.5798635114
C 1.3527561352 0.0184641462 2.0574922409
C 2.2676839926 0.033394926 0.9747669918
C 1.4418470397 0.0326374876 -0.2458608647
C 1.71553888 0.0127332346 3.406194341
C 3.1176847948 0.0344226532 3.6731408239
C 4.0400958143 0.065177424 2.6336892423
C 3.6185262653 0.0537984761 1.2659657203
C -1.0043830823 -0.016625062 2.509289845
C -0.6829643149 -0.0404166447 3.9037779196
C 0.6364606931 -0.0150878888 4.3403688843
P 5.8480398249 0.2323213951 3.0772470007
N 6.4941351925 -0.6510357995 1.7767326543
P 7.9805624818 -0.7843669083 1.3233248017
N 8.5134327108 0.5557466992 0.4158077263
C 9.8645522007 0.6364789127 -0.1335826708
P -2.0244798686 -0.2175211061 5.1930950934
N -3.2330155713 0.6794004809 4.4029087023
P -4.7473198163 0.8085388167 4.7539668722
N -5.6632304667 -0.5222498141 4.2126001084
C -7.1069056591 -0.6122352089 4.4158443133
N -2.5166844909 -1.8264034774 4.9309270573
P -1.9570209612 -3.2678509173 4.8241161794
N -3.3034190058 -4.3061402065 4.7027873352
C -3.127806281 -5.7523155267 4.6306854766
N -1.0408560637 -3.7867443474 3.4912264136
C 0.3788798108 -3.4227810278 3.3825979009
N -0.9167390471 -3.6959873403 6.103443709
C -0.3036870968 -5.0175464552 6.2107886159
C -1.7014421324 -3.7797093883 2.1818152092
N 6.1462486991 1.8455362689 2.6224443229
P 5.6128128062 3.2868201801 2.8221371671
N 6.7256279414 4.328939904 2.0602444006
C 6.5420399725 5.7753491567 2.0983322823
N 4.1579497716 3.817481346 2.1239442359
C 2.8700450018 3.4505457505 2.7297537376
N 5.3446660418 3.6986413433 4.4532031307
C 4.8696452724 5.0173331332 4.864582275
C 4.0824959638 3.821714448 0.6592036556
N -5.2886495675 0.9198381461 6.368756662
C -5.2120054995 -0.2886588159 7.2010093253
N -5.2920355158 2.2789410872 4.1092077333
C -6.7000182011 2.6506918918 4.1719198744
C -4.8609366856 2.1120461512 7.112021353
C -4.5585415942 2.9141215641 3.0166669352

C	-5.2110748876	-1.211362754	3.002473592
C	-1.0943489567	-3.0427553232	7.3988854688
C	-4.5648185931	-3.9028463667	5.3173369063
N	9.2545820948	-0.9202937552	2.4508971986
C	9.6154220203	0.2754720237	3.2248039004
N	8.1203864677	-2.243849629	0.4724755098
C	9.3709080224	-2.6239984699	-0.1725329434
C	9.2444238566	-2.1229377738	3.2936174799
C	6.9358179616	-2.8643209494	-0.1162809922
C	7.521384268	1.2624433723	-0.3969691827
C	6.1372588348	3.0307359045	5.4837564888
C	8.1243593021	3.9214481774	1.9644088141
H	4.3821133175	0.0553450248	0.4915199509
H	-2.0533888631	-0.013993944	2.2223175642
H	0.847761173	-0.0279398955	5.4095506031
H	3.4711849184	0.0372869302	4.7041651235
H	-0.122389846	-2.9798334788	7.9065420812
H	-1.4776820931	-2.0298138996	7.2677415003
H	-1.7840612677	-3.6045136483	8.0520540802
H	-5.8801960521	-0.1654913941	8.0632247486
H	-4.1912266033	-0.4690675027	7.5706217325
H	-5.5288405718	-1.1640715249	6.6345238752
H	-3.8206901815	2.0252544539	7.4626270231
H	-5.514417545	2.2375986736	7.9847358293
H	-4.9431467445	2.9994144155	6.4818309461
H	-0.9062862067	-5.7032523952	6.8289034177
H	0.6863835852	-4.923872649	6.6783322074
H	-0.1725711804	-5.4626732111	5.2234342416
H	-3.5100762581	2.6184121115	3.061181064
H	0.8228853138	-3.2977057301	4.3701287517
H	-4.9689496559	2.6357155018	2.0313473323
H	-4.6307536026	4.0056815621	3.1204862667
H	0.9127803626	-4.2233574857	2.8529272249
H	-7.2658337009	2.3088665804	3.2892216462
H	-7.1644533465	2.2376098683	5.0697328955
H	-6.783028761	3.7450520809	4.2202826831
H	-4.1233245024	-1.2540879612	2.9883719268
H	-1.6599951763	-2.7851338182	1.7129482826
H	-5.5801328802	-0.7219915165	2.0847939598
H	-1.1903237545	-4.4928479095	1.5219398984
H	-5.5913396338	-2.240432213	3.0181303913
H	-2.7460746043	-4.080264897	2.2817311773
H	-7.6756763325	-0.1753478792	3.5786739593
H	-7.3937155311	-1.6696956398	4.5003971009
H	-7.3974082144	-0.1055590045	5.3378721875
H	-4.6587883171	-2.8179589856	5.2753425251
H	-3.9750708074	-6.1928115358	4.0874484573
H	-5.3971119525	-4.3569930709	4.7613660744
H	-4.6399194488	-4.2323749708	6.3684805841
H	-3.0864809291	-6.2226708335	5.627662016
H	-2.2125882031	-5.9984526595	4.088608423

H 0.5139098478 -2.4885896893 2.8258569713
H 5.5419747985 2.9558114195 6.4037875733
H 6.405344015 2.0218170175 5.1668286365
H 7.0598692998 3.5885289131 5.7194470928
H 10.6247471066 0.1387150804 3.6342846193
H 8.918978632 0.4509067336 4.0587007384
H 9.6127801376 1.1591864903 2.587051512
H 8.5196871899 -2.0392853694 4.1184062449
H 10.245472972 -2.2635391389 3.7209050297
H 8.9918706588 -3.0006355886 2.6959497765
H 5.7015972253 5.6979526797 5.1095025554
H 4.2417860177 4.9159534135 5.7607572095
H 4.2670921186 5.472784797 4.0773470129
H 6.0502697101 -2.5643339212 0.4445451116
H 2.9722382556 3.3186621754 3.8068479017
H 6.8061767369 -2.5776678454 -1.1735172072
H 7.0397365036 -3.9572981457 -0.0692821713
H 2.1447145653 4.2528691314 2.5383269152
H 9.4279716147 -2.2776289236 -1.2179255855
H 10.2230638555 -2.2214695721 0.378945801
H 9.4569974231 -3.7192575214 -0.1771112786
H 6.5751921474 1.3126089722 0.139264706
H 3.8088992385 2.8313312741 0.2653912763
H 7.3721617713 0.7818771841 -1.3791163659
H 3.3151010346 4.5410481802 0.3446886494
H 7.8702844884 2.2886020422 -0.5674939255
H 5.0406981593 4.1203659209 0.2299441904
H 9.9314810548 0.2065637618 -1.1465043896
H 10.1654292233 1.6914692477 -0.19640975
H 10.5739008833 0.1177797486 0.5137979751
H 8.1814349163 2.8376542724 1.8652554274
H 7.0095916767 6.2227463643 1.2105004783
H 8.5741806323 4.3871036969 1.0762464596
H 8.7108917737 4.2357172441 2.8454797523
H 7.0021475314 6.2360542563 2.9888188639
H 5.4791909336 6.0247472076 2.0837879674
H 2.4765943916 2.5192037648 2.3069115373
H 1.828754667 0.0404852139 -1.2594391505
H -0.7232848451 0.0208853973 -0.5729532352

VIaaH^+
C 0.1377288905 0.844445263 -0.1560236994
C -0.0552833585 0.602203801 1.2843496632
C 1.2438034862 0.4719168585 1.8360612251
C 2.2244987422 0.6080161531 0.8218621575
C 1.4778049913 0.8456857478 -0.426945054
C 1.5275073315 0.2245020017 3.1821662564
C 2.9129471462 0.1179014969 3.5281958037
C 3.8923046606 0.2533426597 2.5593071042
C 3.5504073865 0.4920380735 1.1845983626
C -1.1401651175 0.4656381973 2.1324656354

C	-0.8895932806	0.1968479522	3.5124974086
C	0.4016962634	0.0854489301	4.0334196264
P	5.6944050074	0.2340323135	3.0838012785
N	6.2867802095	-0.6365873087	1.759423058
P	7.7851884139	-0.9283004944	1.4010996393
N	8.508023968	0.3721211693	0.5765473201
C	9.8873445927	0.2995867092	0.0922206637
P	-2.2622801905	-0.1132955761	4.6468566752
N	-3.6092935767	0.3721769551	3.9037474301
P	-5.0921016923	0.4954509412	4.4706776451
N	-5.8297949089	-1.019398516	4.5130935322
C	-7.1474962466	-1.2366262844	5.1209417996
N	-2.2319313461	-1.6017139976	5.2589093594
P	-1.7159904708	-3.0249742619	4.7738063619
N	-3.0149891712	-4.0895103904	4.9426047081
C	-2.8282467871	-5.5165077074	4.6562163661
N	-1.2321349945	-3.3026090792	3.1987583437
C	0.1282491603	-3.0158139163	2.7191531999
N	-0.3593908456	-3.4206330285	5.6745629427
C	0.3139199875	-4.7139065533	5.5268693863
C	-2.2193862182	-3.3266295176	2.1133680207
N	6.1069221972	1.8370645089	2.7118039577
P	5.8065524682	3.3011807482	3.1343880783
N	7.0221565941	4.2746036477	2.4648717828
C	7.0277496171	5.7227039346	2.6568003846
N	4.4061619169	4.0886166965	2.5733956399
C	3.1065446656	3.7477717515	3.163936782
N	5.6335156345	3.5182099424	4.8170223826
C	5.3641373456	4.8365204745	5.3947957658
C	4.2997476806	4.3102336237	1.1270817226
N	-5.3357659702	1.0534141057	6.036075158
C	-4.9713174871	0.210538208	7.1841756051
N	-5.8713969277	1.6104723498	3.5034042084
C	-7.3175300278	1.8000492976	3.6365309728
C	-5.2011354971	2.4876847429	6.3241377541
C	-5.30314134	2.1247931435	2.2548178948
C	-5.5675753578	-1.9345738879	3.3961851335
C	0.0050071004	-2.7091792287	6.9010317488
C	-4.011649747	-3.8401759857	5.9893245358
N	8.945709463	-1.2227852513	2.6081192948
C	9.4149183708	-0.0956864159	3.4247527282
N	7.8190377298	-2.3713129049	0.520230901
C	9.0657125023	-2.893935016	-0.0314403896
C	8.7730443656	-2.4409792153	3.4092534485
C	6.6213894757	-2.8451114345	-0.170179531
C	7.6482656802	1.2043732032	-0.2696034615
C	6.4547523103	2.6804413404	5.694482227
C	8.3512575595	3.7096359547	2.2383347935
H	4.3610983857	0.5691640612	0.4644533806
H	-2.1667734092	0.5497469398	1.7877340801
H	0.5513054458	-0.1331843784	5.0899151848

H	3.201754741	-0.0618332385	4.5633618845
H	1.0844171283	-2.509257218	6.8990866489
H	-0.5325605204	-1.7631807242	6.9605426726
H	-0.234555596	-3.3074894439	7.7919794906
H	-5.6686405542	0.4031836962	8.0080865017
H	-3.9526191553	0.430237389	7.5342372561
H	-5.0234863392	-0.8453575471	6.9203490671
H	-4.1639094663	2.7540534261	6.5756980153
H	-5.8397983997	2.7376526116	7.1789807591
H	-5.516862299	3.0812533586	5.4656062008
H	0.0296558659	-5.4083486433	6.330130448
H	1.4000977242	-4.5648500286	5.5684402074
H	0.0742632205	-5.1706241244	4.5656926063
H	-4.221529662	1.9957290103	2.2579404496
H	0.8119023119	-2.865021883	3.5528751936
H	-5.725560371	1.6069716233	1.381399256
H	-5.5337333721	3.193920042	2.169487846
H	0.4816707227	-3.8619082122	2.1166717746
H	-7.8763050666	1.1982543958	2.905100549
H	-7.6508580811	1.5341214405	4.6418897861
H	-7.5608457263	2.8561734916	3.4702835438
H	-4.5763543866	-1.7506589282	2.9851558719
H	-2.3169849041	-2.3357167279	1.6471172737
H	-6.3153571002	-1.8201370851	2.5965205988
H	-1.889240551	-4.0370625548	1.346286261
H	-5.6031419038	-2.9663665084	3.7615020634
H	-3.1924575261	-3.6458360411	2.4873298633
H	-7.9586900195	-1.1258709724	4.3865968454
H	-7.1877316853	-2.2563270759	5.5229685546
H	-7.3173995954	-0.5373341115	5.940308574
H	-4.2285530943	-2.774440223	6.0441022047
H	-3.7945174344	-5.9503658259	4.3740553772
H	-4.9323314557	-4.3749787061	5.7279858862
H	-3.6786443093	-4.1974311256	6.9762411278
H	-2.4458228263	-6.0689796418	5.5275187431
H	-2.1394599052	-5.6528275235	3.8205660911
H	0.1388879485	-2.1131851906	2.0998502834
H	5.962175052	2.5994123517	6.6719767066
H	6.5568120821	1.6762836804	5.2803285238
H	7.458889789	3.1077896619	5.8545599566
H	10.3910531327	-0.3529052472	3.8548340861
H	8.7192563247	0.1300432309	4.2473207757
H	9.5257853897	0.7999243766	2.814025067
H	8.0334520648	-2.2989516631	4.2125565336
H	9.7356913304	-2.705413154	3.8639561068
H	8.4465902758	-3.2676013697	2.7758933894
H	6.2906443596	5.3918243523	5.6109085239
H	4.8184519763	4.7111490372	6.3395553794
H	4.7493164892	5.434478958	4.7202947816
H	5.7340607781	-2.4529817926	0.3273726167
H	3.2169645461	3.5044882656	4.2213417464

H 6.6092216385 -2.5335612948 -1.2276496662
H 6.5973468591 -3.9430302238 -0.1381538106
H 2.4346357216 4.6109898994 3.0681418256
H 9.2414351577 -2.5531187848 -1.0641722152
H 9.9134019747 -2.5953030444 0.5880566901
H 9.0233564384 -3.9911555745 -0.0412477656
H 6.7023732166 1.3896124387 0.2373246736
H 3.927326561 3.4155095378 0.6046276149
H 7.4640174227 0.745888831 -1.2556997169
H 3.5980375377 5.1337847697 0.9441593912
H 8.1441691599 2.1692170446 -0.4305649142
H 5.2715729509 4.5792715498 0.7103604226
H 9.9523355604 -0.1236991706 -0.9226030013
H 10.3107227992 1.312161153 0.0629416323
H 10.4988394346 -0.3052227025 0.7639397556
H 8.2675619446 2.640798132 2.0434053055
H 7.4879607322 6.2011538518 1.7820524694
H 8.8050832586 4.1974791315 1.3650763043
H 9.0184617765 3.869813557 3.1018082242
H 7.6031418672 6.0231691454 3.5470540313
H 6.0082443275 6.1005321618 2.7531664814
H 2.6419994023 2.8904924548 2.6575701286
H 1.9289016197 0.9961144668 -1.4013915415
H -0.6595711776 0.9951295913 -0.8750438139
H -2.0196069402 0.7160240637 5.7622845899

VIIbb
C 0.0181851595 -0.0177182644 -0.1010847051
N 0.0212692058 -0.0103253901 1.3665280815
C 1.385272483 -0.0004270185 1.9182715307
C 2.27599107 -0.211046185 0.6807144393
C 1.4505882692 0.3994101406 -0.4641336072
P -1.4056984536 0.1294585627 2.2529650602
N -0.8593588707 0.2389499447 3.831623392
C -0.6765176625 1.4560932882 4.6403044121
C -0.4692540313 0.9235055389 6.0688349454
C 0.1314392223 -0.4737108437 5.8495217742
C -0.6337728849 -0.9863450688 4.6234997213
N -2.3789035162 -1.0428065512 1.9226002613
P -4.0065182876 -1.4226647742 2.3372392959
N -4.0148312271 -3.0989885149 2.5494247152
P -3.5812144221 -4.2631162445 1.6044859251
N -1.9470918635 -4.5853368712 1.3086447019
C -1.1567697774 -3.739469957 0.3751992384
C 0.1739387792 -3.4955042588 1.1019969979
C 0.308288402 -4.7165161197 2.0228612611
C -1.1330057896 -4.9289276811 2.4976224895
C -4.1029212674 -0.9412029818 4.1538907326
C -3.9852435701 0.4092403124 4.4703442953
C -4.0288199099 0.8566274777 5.8192351951
C -4.23590175 -0.127372096 6.7862315383

C -4.3876818996 -1.5056868878 6.5061805611
C -4.313887022 -1.9146281704 5.184132272
C -3.8892857487 2.1873176007 6.3002782734
C -3.9986831673 2.4792196825 7.6568536439
C -4.211712209 1.4324265952 8.6119716276
C -4.3238985378 0.1217819328 8.1759691034
P -3.8351190853 4.2805181665 8.1761596805
N -5.2370259792 5.0064239248 7.4877023473
P -5.9890454892 4.8721440298 6.1286518649
N -6.7963107897 3.4327042277 5.8477091944
C -6.8769624807 2.6757339991 4.5871336122
C -7.4717901383 1.3201837008 5.0066526917
C -8.2958399415 1.6594138229 6.2585204058
C -7.4270871684 2.7058227451 6.96706718
N -4.2158477995 4.0780314452 9.8098453323
P -4.6957835219 5.0931903114 10.8939206192
N -6.2739466808 5.7025275668 10.8831336029
C -6.6764143346 6.7696033133 9.9287803727
C -8.0412154335 6.3162109848 9.3900669891
C -8.6009722991 5.4422274497 10.5213632301
C -7.3537235467 4.6958727828 11.0062477735
N -3.8067067039 6.5195812852 10.9748768737
C -2.3323878644 6.4069095688 10.8571541326
C -1.8201933369 7.7711352177 11.3418402801
C -2.8649364094 8.1770566766 12.3931661754
C -4.1787491386 7.728547746 11.7399275912
N -4.6195654784 4.2683685053 12.3542741148
C -4.3416174491 2.8269430441 12.480968584
C -4.1762187515 2.6362365312 13.9953577602
C -5.1482328603 3.6725109128 14.5824842303
C -4.9771303901 4.8777625816 13.6424960032
N -7.2140565935 6.0239671511 6.0066166073
C -8.6253732399 5.7522015896 5.691748798
C -9.2843400115 7.1412040696 5.7641981545
C -8.144265687 8.099618594 5.381763977
C -6.9279703087 7.4621557436 6.0689404348
N -4.9940480503 5.1144249132 4.7693232077
C -5.5398656342 5.4312125832 3.4344686624
C -4.5588484537 6.4577393777 2.8462668838
C -3.2281689838 6.0737888589 3.5104230455
C -3.6455172605 5.707500857 4.9392528574
N -4.1724817449 -4.1602738036 0.0323658422
C -5.5836639248 -3.7351477279 -0.1347192433
C -5.9088491935 -4.1055856958 -1.5891535853
C -5.045106627 -5.3540150897 -1.8276140417
C -3.737997376 -4.9900892343 -1.1113071712
N -4.115781932 -5.6721517761 2.3442386674
C -4.694952173 -5.7322748945 3.6977504874
C -5.206332775 -7.1767287074 3.7937580734
C -4.1925112095 -7.9598090922 2.9439228904
C -3.9220113061 -7.008744033 1.765850086

N -2.0321951285 1.6656924147 1.8744554512
C -1.1725723354 2.8560880843 1.721725524
C -1.7842746066 3.6286329232 0.5424018596
C -3.2729998051 3.2615395496 0.6333755083
C -3.2436534725 1.7799579363 1.0266454119
C -4.5823774298 -2.152904866 7.8161814574
C -4.5446582141 -1.1964771043 8.7975172598
H -4.39445825 -2.9562410333 4.8863006487
H -4.2949184614 1.7125006848 9.6583080375
H -3.7263732088 2.9826003114 5.5777768187
H -3.8268798015 1.1460146449 3.687387666
H -5.9849457572 7.6954061668 5.5595013348
H -2.4830076032 6.8761730657 3.4900407634
H -6.8340740538 7.8035872576 7.1102542428
H -9.6108417798 7.3444217263 6.791399995
H -8.3194297717 9.132253357 5.7021064237
H -8.0016483557 8.1013697698 4.2930153148
H -10.1591638159 7.2207919166 5.1102465649
H -9.0745636864 5.0502188975 6.403990476
H -8.7350039928 5.3126424599 4.6886743343
H -6.560518868 5.8245313887 3.5109323069
H -4.849110003 7.4725800911 3.1478536812
H -4.5263573363 6.4273717947 1.7515197415
H -2.7949293032 5.1993966893 3.0098990117
H -5.8881029706 2.561930713 4.1318162608
H -2.9547684862 5.0026281678 5.4118687886
H -7.528828703 3.1880265881 3.861707438
H -8.0627613088 0.8647291135 4.2053729886
H -6.6657985953 0.627445582 5.2656487595
H -8.4916944464 0.7875243168 6.8912465322
H -9.2606011502 2.1011241123 5.9741272959
H -6.6653390419 2.2347495096 7.600378225
H -8.012485419 3.3822513585 7.6018746783
H -7.8838003618 5.7174896329 8.4889490547
H -8.6900813121 7.1612884965 9.1336897926
H -6.76051056 7.7241292176 10.4667740509
H -9.0057193374 6.067506899 11.3283037715
H -5.9433495208 6.8766406935 9.1250562474
H -9.3912754155 4.7579542555 10.1929884225
H -7.1558837562 3.8226520248 10.3659795298
H -7.4375296434 4.3467978595 12.0420358174
H -2.0445212964 6.1757867831 9.8271183855
H -1.9507910343 5.5998423578 11.5016922283
H -5.8914728106 5.4791150559 13.5595073591
H -4.174282502 5.5432824285 13.9988015693
H -4.9440088564 3.9230269683 15.6290015674
H -6.1774143075 3.2954159263 14.5199902093
H -3.1468505413 2.8755524622 14.2931411202
H -4.3911732503 1.6106519105 14.3139454548
H -3.4518854597 2.5510873919 11.9095805542
H -5.1817404264 2.2273611377 12.0971708644

H	-0.8001960425	7.7155152622	11.7365654263
H	-2.6983795583	7.6185263276	13.3241478771
H	-2.855470041	9.2464944188	12.6313292612
H	-1.8234828606	8.4920467384	10.5137757868
H	-4.9739711819	7.5203753459	12.4613774561
H	-4.5496332615	8.5055384553	11.0551187381
H	-3.678335713	6.5965458664	5.5842793921
H	-5.5800379568	4.5280846887	2.8090701532
H	-0.7361123452	0.6752548593	-0.4934430066
H	-3.821753129	3.4309467465	-0.2991872995
H	-0.2168623582	-1.0182664358	-0.4928055959
H	2.418340179	-1.2841172659	0.503768495
H	1.7565443706	0.0476607953	-1.4552500119
H	1.5372237738	1.4939551169	-0.4504196476
H	3.2645181247	0.2455017538	0.7976405126
H	1.5302329077	-0.791154217	2.6637453206
H	1.6110035145	0.9571190412	2.4116754919
H	-0.1312171978	2.566634354	1.5374850936
H	-1.3654118487	3.2643794292	-0.4046946154
H	-1.5949195859	4.7060897077	0.6030976121
H	-3.7589488047	3.8497241451	1.4210928632
H	-1.5522310792	2.1093994925	4.573598718
H	-4.1427143672	1.4710854214	1.5688220773
H	0.2010259098	2.0274822213	4.2980872074
H	0.1681792571	1.5839329244	6.6657067487
H	-1.435279065	0.8398827566	6.5752744106
H	0.0121825598	-1.131840953	6.7164187649
H	1.2031105311	-0.3984029334	5.6201845851
H	-1.5892347729	-1.4442819421	4.9073550628
H	-0.0670744408	-1.7314482428	4.0518998749
H	0.0906246712	-2.5799290402	1.6936660722
H	1.0138643659	-3.3820695922	0.4073187493
H	-0.9997023005	-4.2865434396	-0.5648452746
H	0.6529747844	-5.5915846075	1.4559420401
H	-1.6740916257	-2.8010610318	0.1592094044
H	1.0001036344	-4.5586753132	2.857793076
H	-1.3620399533	-4.2596141027	3.3409152175
H	-1.3330178062	-5.9586877578	2.8159087484
H	-5.6917749076	-2.6668954441	0.075922261
H	-6.240169743	-4.2824837469	0.5594323049
H	-2.9101279984	-7.1237376036	1.356766295
H	-4.633922656	-7.1885131542	0.9440362316
H	-4.5565786013	-8.9400585312	2.6179987419
H	-3.2683494309	-8.1156459628	3.5157123851
H	-6.2069371449	-7.250573268	3.3477986698
H	-5.2697959039	-7.53028772	4.8284067223
H	-5.4832089397	-4.9848397846	3.8168216923
H	-3.929715358	-5.5326098165	4.4639622941
H	-6.9792018183	-4.2771487588	-1.7447452414
H	-5.5061167251	-6.2278026897	-1.3475599264
H	-4.894642922	-5.5877645352	-2.8874409031

H -5.5940930669 -3.2988009795 -2.2640122439
H -3.1653206386 -5.8619715147 -0.7825109488
H -3.089245747 -4.4008423666 -1.7762602965
H -3.1729072618 1.1339580189 0.1406665103
H -1.1890844942 3.465105492 2.6368182597
H -4.658634867 -1.3731538777 9.8620377558
H -4.7311823894 -3.216584131 7.970607162

VIIbbH+
C -0.0096274931 -0.0088015211 -0.1123161998
N 0.0052751064 0.0080166235 1.3580609036
C 1.3762004167 -0.0088092441 1.8980869893
C 2.251803252 -0.2341213182 0.6526632247
C 1.4258198397 0.3847937448 -0.4869513932
P -1.4114885185 0.1243553213 2.2509618009
N -0.873906231 0.2230732536 3.8365505806
C -0.6505414517 1.4340395273 4.6380822986
C -0.4217613831 0.8950301948 6.0610697287
C 0.1778693647 -0.499701 5.8219817359
C -0.6098024097 -1.0076313431 4.6079534396
N -2.38648083 -1.0473459546 1.9313639577
P -3.9989113499 -1.458619797 2.3542697365
N -3.9902560276 -3.1245181991 2.5837644569
P -3.583493401 -4.2897433655 1.6153953148
N -1.9589474519 -4.5913884803 1.2765226435
C -1.1968105201 -3.7463430738 0.3196037424
C 0.1541855137 -3.5046649541 1.0080420065
C 0.3194513875 -4.7362059784 1.9096948085
C -1.1048481355 -4.9565559839 2.4304643924
C -4.0795685791 -0.974501358 4.1784562807
C -3.9684505894 0.370932763 4.5053536686
C -4.030917598 0.8028011029 5.8608131188
C -4.240940221 -0.1862810717 6.8230300003
C -4.3758661903 -1.5618312194 6.5296681063
C -4.2883690132 -1.9564747928 5.2052033598
C -3.9292118538 2.1296461675 6.347742929
C -4.0562215969 2.3914601573 7.7111914151
C -4.2604683888 1.3474095863 8.6704767385
C -4.3500159757 0.0433974319 8.2176616871
P -4.0782172696 4.1257916359 8.2435214085
N -5.1849425681 5.0196878485 7.4672567232
P -5.9750793832 4.9025614067 6.0927905324
N -6.7423227569 3.4560767664 5.8460613196
C -6.8286855045 2.7161782393 4.5667825534
C -7.4658666375 1.3772490542 4.9691382697
C -8.3316529291 1.7413881787 6.1851687115
C -7.4628107673 2.7586615303 6.9350925262
N -4.1809550534 4.121223231 9.8409118333
P -4.7224384524 5.1512168824 10.9247125605
N -6.295977973 5.6947885374 10.8344414755
C -6.7249908014 6.7838139887 9.9160298248

C	-8.1010323565	6.3300692702	9.4093786833
C	-8.6354617499	5.4616342173	10.5576222793
C	-7.3849519994	4.7027625329	11.0150013264
N	-3.8424163193	6.5684069425	10.9253662379
C	-2.3620329446	6.4654709667	10.8048337367
C	-1.8511967739	7.815222298	11.3317419252
C	-2.9057011627	8.2033640215	12.3788703656
C	-4.2135754465	7.7800112713	11.7017134986
N	-4.6256307511	4.3185760036	12.3576547675
C	-4.3054033968	2.8792416783	12.4974945601
C	-4.1464971722	2.7051261029	14.0138788485
C	-5.1414354964	3.7241267961	14.5911409348
C	-4.9835277919	4.9307879555	13.6534562357
N	-7.1795210982	6.053469355	6.0433330286
C	-8.5837082544	5.8037047122	5.6483040761
C	-9.2213135233	7.2012109621	5.7031153098
C	-8.0553075589	8.1416399039	5.3589792191
C	-6.8731878073	7.4962951811	6.0951002128
N	-4.9470186673	5.1746969237	4.796188576
C	-5.4574914844	5.5436972395	3.4557524406
C	-4.4251271129	6.5462224603	2.9237781276
C	-3.1170230247	6.0559062722	3.5613837056
C	-3.5427259656	5.62439525	4.9720986986
N	-4.2231941141	-4.1680899634	0.0674499943
C	-5.6501852277	-3.7750113215	-0.0498588861
C	-6.0175417423	-4.1474475045	-1.4935866288
C	-5.1355821521	-5.3751193471	-1.7678518349
C	-3.8122594635	-4.9861393302	-1.0972814598
N	-4.0883419618	-5.6966389151	2.3701048169
C	-4.6590431842	-5.7669697425	3.7269858655
C	-5.1606367572	-7.2154160925	3.8224124041
C	-4.1595112486	-7.9890516912	2.9496034575
C	-3.9172473072	-7.030667713	1.7727330325
N	-2.0579040415	1.665452742	1.9044684935
C	-1.1921091096	2.8497111522	1.722060913
C	-1.8161608492	3.6157492556	0.5448323066
C	-3.303646822	3.2481778568	0.6498045284
C	-3.2671753868	1.7689456821	1.0491206993
C	-4.576928907	-2.2246396419	7.8316817045
C	-4.5611245801	-1.2826870976	8.8245615086
H	-4.3568824838	-2.9951688654	4.8932788409
H	-4.3469019891	1.6117169153	9.7198043889
H	-3.7854975438	2.935089042	5.6344418879
H	-3.8026242559	1.1146078824	3.7305259513
H	-5.9089066826	7.7073876876	5.6178984149
H	-2.3327143898	6.818570659	3.5860739677
H	-6.8149173585	7.8420092547	7.1356400576
H	-9.5809364581	7.407455059	6.7183455956
H	-8.2279451977	9.17695893	5.6681109707
H	-7.872277497	8.1380668752	4.276913439
H	-10.0707279287	7.2934251298	5.0198098676

H	-9.0724943537	5.0999568375	6.3298886194
H	-8.6422619989	5.3803492072	4.6361221343
H	-6.4635261631	5.9704045673	3.5275210199
H	-4.6647183935	7.5571646195	3.2766363233
H	-4.3908530663	6.5687200686	1.830178239
H	-2.7295460866	5.1933897043	3.008015692
H	-5.83705607	2.5858159856	4.1242552134
H	-2.907200884	4.8199346376	5.3571170753
H	-7.459838131	3.2558610776	3.8460445671
H	-8.0356608959	0.9332692723	4.147815681
H	-6.6848000335	0.6681888784	5.2594647994
H	-8.5823507701	0.8768774682	6.807150853
H	-9.2682584291	2.2131484287	5.861398288
H	-6.7485439955	2.2583893983	7.6007833615
H	-8.0524274589	3.4590425538	7.5379741685
H	-7.9717062661	5.7307112856	8.5032201314
H	-8.7530215821	7.1760642833	9.1687687578
H	-6.8007498378	7.7229484525	10.4784046421
H	-9.0119693116	6.0924679919	11.3723098542
H	-6.0069144555	6.9156773398	9.1036092084
H	-9.4406137873	4.7849039886	10.2543425313
H	-7.2187370841	3.816073808	10.3847469555
H	-7.437728759	4.3739083133	12.0578234363
H	-2.0715784738	6.2778133654	9.766355711
H	-1.9793953694	5.6368487884	11.4171429705
H	-5.9013613022	5.5249337442	13.5708501251
H	-4.1804672545	5.5977357864	13.9997142174
H	-4.9467333284	3.9808163729	15.6367084087
H	-6.1637412478	3.3308750827	14.5244884338
H	-3.1237837949	2.9638175689	14.3157194877
H	-4.3440510141	1.6780772995	14.3356483123
H	-3.4030450454	2.6253463563	11.936720794
H	-5.1294012021	2.2614115203	12.1123652293
H	-0.8382590513	7.740664947	11.7384699015
H	-2.7547228362	7.6272139804	13.3009386362
H	-2.8921172034	9.2669000388	12.6367630631
H	-1.8360013663	8.5562398335	10.5227373517
H	-5.0220938396	7.5710733568	12.4070349851
H	-4.5635157285	8.5605845679	11.0130830197
H	-3.4871868439	6.4589892669	5.6827663247
H	-5.5144591394	4.656535373	2.8118586213
H	-0.7562364607	0.6932170393	-0.5029736924
H	-3.860620921	3.4133012968	-0.2781650516
H	-0.2630335278	-1.0080792509	-0.4929049118
H	2.378714108	-1.3089877846	0.4780275551
H	1.7176990837	0.0239492903	-1.47836582
H	1.5298417937	1.4777726261	-0.4797534758
H	3.2468181847	0.2093984406	0.7597154075
H	1.5134203701	-0.80318002	2.6405147701
H	1.6215753458	0.9450631255	2.3890180131
H	-0.1561676911	2.5518742827	1.5241507893

H -1.4069339641 3.2469636697 -0.4038970159
H -1.6249511413 4.6933548322 0.5948611757
H -3.7841624232 3.839641816 1.4400014593
H -1.5170959652 2.1037425613 4.5857423812
H -4.1659222763 1.453286582 1.5887168257
H 0.2286523183 1.9936586867 4.2812841665
H 0.2258494594 1.5505450624 6.6529533974
H -1.3808630804 0.803288139 6.5815662251
H 0.0812933887 -1.1632463755 6.6872089314
H 1.2435236889 -0.41695899 5.5720343226
H -1.5517882594 -1.484105068 4.9075294184
H -0.0480056869 -1.7389742583 4.015705898
H 0.0876215121 -2.5959572643 1.6133065196
H 0.9716651782 -3.3819604161 0.2891755715
H -1.0665323013 -4.293480791 -0.6237304504
H 0.6450791756 -5.6032024041 1.3206572048
H -1.7217420908 -2.8093780249 0.1160506222
H 1.0395281539 -4.5892938766 2.7221299552
H -1.3048461667 -4.3018335464 3.2928136331
H -1.2942049938 -5.9911115588 2.7377874573
H -5.7753737352 -2.7099560897 0.1674379039
H -6.2689675861 -4.3399557526 0.6642235712
H -2.9180324643 -7.1446713505 1.3348712268
H -4.6532294987 -7.198362111 0.9707822079
H -4.5265380865 -8.968056889 2.6251482118
H -3.2236646137 -8.1448995711 3.5017075868
H -6.1687535866 -7.291644734 3.3948132717
H -5.2036381215 -7.5757706457 4.8555308969
H -5.4539561762 -5.0273089774 3.8544865531
H -3.8886531767 -5.5645921423 4.4874476409
H -7.0882877389 -4.3427225284 -1.6117902734
H -5.5601209938 -6.2612746915 -1.2775696303
H -5.0173473641 -5.6002838982 -2.8330445174
H -5.7454983691 -3.3312704365 -2.175375995
H -3.2103580091 -5.8465117957 -0.79219565
H -3.2006735787 -4.3786873884 -1.7796134481
H -3.1895867502 1.1223462996 0.1649006254
H -1.1876709754 3.4665116408 2.6329359844
H -4.6831665165 -1.4717693435 9.8851836859
H -4.71513092 -3.2910644864 7.9718361862
H -2.8106575402 4.63719851 7.8765444026

VIIIaa
C -0.4609520895 0.7274857477 -0.4386028613
C 0.1203512445 0.3872558065 0.9404801831
C 1.6213650557 0.0746560087 0.8254821367
C 2.4111583212 1.2527276932 0.2268975323
C 1.819689876 1.633980255 -1.1449762863
C 0.3029555888 1.8981155454 -1.0841595112
P -0.7776784838 -0.9556708317 1.8928119544
N -2.3018528965 -0.1934530731 1.9363910693

P	-3.5201735114	-0.4540563424	2.8678690746
N	-3.3450210489	-0.958709002	4.490326571
C	-2.9657695679	-2.3510153444	4.7582414356
P	2.4451392937	2.7252863019	1.4107502868
N	3.3158560506	2.0161281204	2.7135349882
P	2.7845825239	1.9895905893	4.1873454688
N	1.4117735959	2.8644635376	4.674983938
C	1.5162769836	4.3248467319	4.7388842048
N	3.6338436663	3.6467773856	0.5845645019
P	3.5407254096	5.1977914163	0.3878570049
N	4.6840184108	5.6202289744	-0.7906491787
C	5.8184398053	4.7393196599	-1.0598724919
N	2.1377605608	5.9564325677	-0.2202744163
C	1.7687603072	5.6664092762	-1.6101637686
N	3.7450671371	6.0757239507	1.8368325437
C	3.6755784092	7.5324784759	1.9032066936
C	0.9538286676	6.0337745131	0.6451209966
N	-0.9517067685	-2.1442876012	0.6731110308
P	-0.380585498	-3.5413652678	0.321360714
N	-0.6866031603	-3.9775340175	-1.3039560451
C	-0.2031375679	-3.0069240362	-2.2970220448
N	1.2988305404	-3.7349879794	0.3995723512
C	2.1119271683	-4.5977512346	-0.4479532878
N	-1.0445919963	-4.7870679982	1.2690544231
C	-0.5996616028	-6.1668850799	1.1364000671
C	1.9563380428	-3.338592004	1.6401105497
N	2.3562565074	0.416259064	4.6726396423
C	1.6627692787	0.1050974822	5.9176587506
N	3.9845457467	2.6298062904	5.2186967432
C	3.8115160512	2.5318790206	6.6642929772
C	3.1596170842	-0.7003097497	4.180772121
C	5.384712099	2.557466652	4.8041422323
C	0.0906705734	2.4276411609	4.203330249
N	-4.3350202245	1.0193430544	3.0742480727
C	-5.5551652658	1.1247810189	3.8637049536
N	-4.5274552509	-1.6880992304	2.2599461657
C	-5.7990014744	-2.0252974819	2.8934663645
C	-4.1684747623	2.0885332194	2.0925486164
C	-4.5259906471	-1.9153879317	0.8120993552
C	-2.6364418865	-0.0290881209	5.3779549416
C	-2.3492941392	-4.6419814025	1.9007368501
C	-2.0559156209	-4.4063344178	-1.5986588986
C	4.6133711157	5.4986375635	2.8621044173
C	4.931301178	7.0181913792	-1.1203269191
H	2.3502970973	2.5040427901	-1.5519944274
H	0.1156452288	2.8100923103	-0.499369656
H	-0.0823021037	2.0887908601	-2.0971385944
H	-1.5284580139	0.9639540871	-0.348739949
H	-0.3851988218	-0.1609941398	-1.0798497805
H	2.0286645224	-0.1862572472	1.8056481306
H	1.7505347637	-0.8101158635	0.1817625679

H	3.4669165956	0.9682048453	0.1030869847
H	1.9998316951	0.8003548046	-1.843693293
H	0.370045545	5.1023139816	0.6216878553
H	1.2469553587	6.2207474927	1.6789222339
H	0.3178560353	6.8611430003	0.3016159389
H	0.7251573464	4.7057836216	5.3985973212
H	1.4008274234	4.7846478826	3.7459146155
H	2.4848542144	4.616931667	5.1477496304
H	-0.1824948344	2.9301951919	3.2660689627
H	-0.6614232166	2.666273082	4.9686107087
H	0.0755670221	1.351829888	4.0254063462
H	1.122936593	6.4733222502	-1.9808165256
H	1.2219930304	4.7158113359	-1.7008336736
H	2.6611853665	5.6156101622	-2.2362554978
H	3.6603586875	-0.4194953447	3.2533448779
H	5.5485812182	3.7145087469	-0.8039353349
H	3.9217708649	-1.0039715254	4.9192052537
H	2.5072323285	-1.560709668	3.9848952885
H	6.0731502106	4.7948649018	-2.1276051624
H	2.3597903515	-0.2219887171	6.7070440046
H	1.1109410486	0.9726967291	6.2817750945
H	0.9470749916	-0.7092315792	5.736060335
H	5.4458420025	2.5469620005	3.7156217974
H	5.7217577978	7.4645128452	-0.4936965723
H	5.8775340929	1.6491449528	5.1915004302
H	5.2525909066	7.0916767526	-2.1683283718
H	5.9268807942	3.4312141261	5.1921424599
H	4.0182193392	7.6061277423	-1.0053174722
H	4.1517505386	1.5614804193	7.0644916484
H	4.3981212403	3.3214732612	7.1527085008
H	2.7619119095	2.6719707545	6.9329060418
H	4.5813508476	4.4121235505	2.8077081349
H	4.6658256327	8.0014287396	1.7801342417
H	5.6585102553	5.8360402795	2.7525398306
H	4.2581780331	5.8058485827	3.8544728979
H	3.2821252394	7.8328599634	2.8847587175
H	3.007263868	7.9212242043	1.1332307831
H	6.709503817	5.0316945379	-0.4788517755
H	2.9028431813	-2.8295980247	1.4168988273
H	1.3301240118	-2.6417651548	2.2029427572
H	2.1684045237	-4.2123330576	2.2785819123
H	-3.1923708685	-2.5804506061	5.8079147859
H	-1.8935188847	-2.5264317568	4.5827817821
H	-3.5347489296	-3.0308357018	4.1242667115
H	-1.5447542251	-0.1230768812	5.2779546998
H	-2.9123611998	-0.2472005107	6.4181471454
H	-2.9184175849	1.000563479	5.1498245403
H	2.5013530118	-5.4624502394	0.1156577971
H	2.9756927235	-4.0365357781	-0.8338083811
H	1.525935886	-4.967799968	-1.2900542022
H	-3.2166728087	1.9621290652	1.5758626814

H 0.795712325 -2.654742878 -2.0292465599
H -4.9831459043 2.0915782651 1.3491624946
H -4.1738646368 3.0588715048 2.6090235549
H -0.1513234364 -3.4957519511 -3.2783780902
H -6.4644894045 1.0167724819 3.2494234509
H -5.5681013323 0.3668928881 4.6494908827
H -5.5917982649 2.1124534919 4.3443442351
H -3.5300055684 -1.7306238843 0.4078840853
H -2.7766953703 -3.5729228312 -1.5522294163
H -5.2566170819 -1.2718504487 0.2934064949
H -2.0821735247 -4.8294045179 -2.6101648626
H -4.7956692908 -2.962467586 0.6185167771
H -2.3747331105 -5.1832919009 -0.9006975938
H -6.6340497272 -1.4188908152 2.5053480104
H -6.0321193296 -3.0810794942 2.6983857915
H -5.7382824522 -1.8848911412 3.9745979312
H -2.6274394435 -3.5902800643 1.9472289109
H -1.2478644061 -6.7512733732 0.4605651373
H -3.1354085577 -5.1892279839 1.3515133218
H -2.3123119853 -5.0418079222 2.9238452141
H -0.6114354417 -6.6559299334 2.1204124243
H 0.4194557904 -6.2097964931 0.7500034955
H -0.8642858404 -2.1304227491 -2.3662427472
H -0.0002453184 1.2670945657 1.5864692492

VIIIaaH^+
C 1.8409904567 -0.8413373143 -0.2923855501
C 1.0791711202 -0.6079808003 1.018096935
C 2.0626312612 -0.3030419554 2.1590467548
C 3.0100998022 0.8812118867 1.8517198642
C 3.6906653015 0.7226162716 0.4726934761
C 2.7001236139 0.3778686324 -0.6507362675
P -0.0355787704 -2.0595984174 1.4959648197
N -0.9703599069 -1.1721399885 2.6206249744
P -1.9450705032 -1.7285886622 3.7112084507
N -1.48863268 -2.9973860609 4.7511119599
C -1.4384425297 -4.362000244 4.2031075304
P 2.2095194042 2.5163516748 2.0792084634
N 1.5046405146 2.4842827503 3.521694776
P 1.1398011911 3.4941566461 4.6787062425
N 0.0420240768 4.7333033664 4.3594910022
C 0.4191524923 5.6822118581 3.3028538059
N 3.2964348229 3.695779998 1.8733457665
P 3.6709289667 4.7255094544 0.7349294975
N 5.091540031 4.2216618306 -0.018839638
C 6.1175351811 3.5375635656 0.7790940542
N 2.6315204516 4.9771538827 -0.5551473093
C 2.5264619618 4.0009519486 -1.6468873291
N 3.7726838613 6.2528650413 1.4424545567
C 4.2253842093 7.3913356177 0.6342246046
C 1.4188813809 5.7873473603 -0.398315767

N -1.0137519409 -2.1131086638 0.102866184
P -1.2614210903 -3.0883858118 -1.089043228
N -2.3103527145 -2.3979521606 -2.2420217837
C -1.9199636688 -1.062526918 -2.7113771067
N 0.0860494494 -3.4632979761 -2.0442654895
C 0.0887353787 -3.6675163242 -3.4899573268
N -1.9270823464 -4.5746252702 -0.6357014128
C -2.1174149256 -5.652023679 -1.6010698306
C 1.2118425614 -4.0976672004 -1.3641622519
N 0.4213215227 2.5980020355 5.8947603485
C 0.1972972639 3.2417613333 7.1918771582
N 2.5004019544 4.3556543894 5.1938557547
C 2.4061192338 5.532244175 6.0647943448
C 0.585423086 1.1405666195 5.9689514569
C 3.7371044583 3.5943294488 5.4097445017
C -1.3962144273 4.4316209695 4.3419858989
N -2.2225283925 -0.4644797356 4.8183644991
C -3.1206877147 -0.6699565924 5.9533943784
N -3.3966761743 -2.3300500332 3.0609387118
C -4.4497146132 -2.9019513729 3.9020573965
C -2.2731949776 0.9002129047 4.2955111234
C -3.9266099017 -1.6701305812 1.8627769684
C -0.3364319043 -2.7282541064 5.6214078263
C -2.6978253878 -4.7377993157 0.5906992616
C -3.7458351912 -2.4794287475 -1.9617002028
C 4.1476073075 6.3706817378 2.8555104188
C 5.6589405923 4.9536499285 -1.15773629
H 4.2698495428 1.6159712443 0.215760789
H 2.0368257919 1.2320204373 -0.8450341307
H 3.2570912011 0.2013782378 -1.5812077588
H 1.1398676135 -1.0674394261 -1.1018117894
H 2.4973138018 -1.7185679777 -0.1854154631
H 1.5200661702 -0.1232386056 3.0908755676
H 2.6952984912 -1.1883231083 2.3250433252
H 3.7911649845 0.9212956909 2.6279420605
H 4.4136617703 -0.1003817738 0.5636719802
H 0.5438036098 5.1556421096 -0.1859879674
H 1.539817453 6.5058550749 0.4123921813
H 1.2286490184 6.3343847341 -1.3296385844
H -0.0896623252 6.6370752934 3.4778680871
H 0.1263248488 5.3118604814 2.3086791424
H 1.4948316978 5.8547314525 3.3005567745
H -1.6956474692 3.8898650057 3.4321416352
H -1.9450348539 5.3787343862 4.3809995882
H -1.6735566491 3.8342091849 5.2090527824
H 2.468710139 4.5305161276 -2.6060251811
H 1.6221310742 3.3878911268 -1.5365656065
H 3.3931221873 3.3399108174 -1.6616721069
H 0.6006651679 0.7106882287 4.969865822
H 5.6558349263 3.0015916903 1.6082454131
H 1.5047525068 0.8609002202 6.5049811466

H	-0.2736889064	0.7215265407	6.5020649551
H	6.6443838769	2.8166602823	0.1429539404
H	1.0581388804	3.1200678762	7.86653314
H	-0.0010522619	4.3086751722	7.0652755927
H	-0.679084242	2.788965334	7.6701391059
H	3.8131236314	2.7751400643	4.6949848742
H	6.3932605382	5.7056609887	-0.8348675679
H	3.790109814	3.1864929592	6.4307771695
H	6.1669433154	4.2420063881	-1.8195481616
H	4.5975498155	4.2570172241	5.260920882
H	4.8753057136	5.4521834514	-1.7296527435
H	2.4823142585	5.2620463077	7.1281901161
H	3.2313614609	6.2155722848	5.8280874226
H	1.4640283474	6.0559159579	5.9011431079
H	3.7521398631	5.5240798878	3.4146784013
H	5.3205402717	7.4943983123	0.6445652742
H	5.2400469839	6.4165896407	2.9861470496
H	3.7175000643	7.2959281792	3.2584153002
H	3.7921063037	8.3114228997	1.0433751343
H	3.8910311711	7.2890025501	-0.3998253164
H	6.8551708282	4.2491424229	1.1800681862
H	2.156498182	-3.7260651587	-1.7822175797
H	1.200950288	-3.8691564545	-0.2948966407
H	1.1864557212	-5.1928535999	-1.4818900962
H	-1.4461590367	-5.0735514471	5.0379124558
H	-0.5323972467	-4.5287348397	3.6026045013
H	-2.3077101605	-4.5572879578	3.5755065776
H	0.6176845485	-2.8190834018	5.0790451548
H	-0.3389716938	-3.4524307573	6.4450138347
H	-0.4058181519	-1.7232927024	6.0408607395
H	0.2288571284	-4.7305238546	-3.7450050888
H	0.9170307922	-3.1049103153	-3.9442951244
H	-0.8516607673	-3.3297925864	-3.9257791329
H	-1.5126998226	1.0277268608	3.5246860872
H	-0.8486175835	-1.0348918584	-2.9259761312
H	-3.2615032639	1.1499193529	3.8730845014
H	-2.0683591525	1.5975741522	5.1150757594
H	-2.4653098933	-0.8353320992	-3.6357896666
H	-4.174060318	-0.4676601026	5.701232837
H	-3.0380940366	-1.6926876739	6.3253030442
H	-2.834667406	0.0139252296	6.7644800776
H	-3.1077989719	-1.3507004647	1.2175124732
H	-4.0619660056	-1.7714262273	-1.1785325721
H	-4.5597477702	-0.8045346188	2.1194755331
H	-4.2981809178	-2.2471809485	-2.8797862657
H	-4.5442459976	-2.3884143205	1.3088920743
H	-4.0195829252	-3.4894315487	-1.6506161967
H	-5.1954489692	-2.1475482828	4.1976586991
H	-4.971803176	-3.6884866273	3.3408681764
H	-4.027049841	-3.3464391034	4.8042769529
H	-2.5142968529	-3.9061088646	1.2679124755

H -3.152988788 -5.690053896 -1.976728361
H -3.7806038229 -4.7925458814 0.3874348053
H -2.4001374772 -5.6691447031 1.0912357129
H -1.8935670788 -6.6162698535 -1.1260143384
H -1.4502186617 -5.5314261335 -2.4550017891
H -2.1393037015 -0.2864682683 -1.9617804347
H 0.3822215624 0.2344012141 0.8839949789
H 1.209899325 2.6334130197 1.085077044

VIIIbb
C -0.3419115638 -0.7799683712 -0.9858559625
N 0.1246255265 -0.0719399215 0.2383739156
C 1.5889704906 -0.2516737528 0.3893813802
C 1.9427438167 -1.3909644724 -0.5740148473
C 0.9482384209 -1.1707247188 -1.7223463557
P -0.8085972158 -0.1336754327 1.6547455116
N -0.2305736787 1.1982766656 2.5032298087
C 0.260334258 2.4514494645 1.9063586683
C 0.3420394463 3.4104282134 3.1033185802
C 0.703805508 2.4699731213 4.26361778
C -0.1494884794 1.225715793 3.9773414615
N -0.8360428358 -1.332573084 2.6468208343
P -1.0495266318 -3.0085630902 2.5629162477
C 0.7853068549 -3.5088806733 2.736227022
C 1.0179959058 -4.9726225212 2.335631569
C 0.4308104333 -5.9498835245 3.3641267064
C 1.0784059615 -5.7167320059 4.7312098949
C 0.8472740947 -4.2664795704 5.192879106
C 1.3411379322 -3.2448608731 4.1499286133
P 0.4458811952 -7.6997707286 2.6872429879
N 2.1144014575 -7.940295005 2.5463951227
P 3.1641357058 -8.983521787 3.017863057
N 2.6574593903 -10.5877169882 2.857855345
C 1.5868442824 -10.935834336 1.8906396231
C 1.847032695 -12.4149404104 1.5407157569
C 2.6880855029 -12.9323520126 2.719933481
C 3.5736607424 -11.7236477612 3.0381311427
N -1.2612753066 -3.361558743 0.8990893849
P -1.9915565147 -4.4064930999 0.0077736757
N -0.9853241246 -5.5423455585 -0.7279660626
C 0.3434990364 -5.2098139739 -1.2577488918
C 1.1202400943 -6.5262397829 -1.1259674421
C 0.0419738468 -7.587385612 -1.4031689042
C -1.2426122892 -6.9943744498 -0.7874554545
N -3.2298371114 -5.3070520829 0.7147179287
C -4.3673307987 -5.8538016706 -0.0479068517
C -4.8311770206 -7.0557410302 0.7883637143
C -4.47140547 -6.6433124727 2.2241824069
C -3.126552983 -5.9242885094 2.0532799807
N -2.7358224123 -3.6582457886 -1.2991397493
C -3.5991797145 -2.4821539737 -1.1282921617

C -3.7618224648 -1.9550164255 -2.5595108419
C -3.7436453463 -3.2436918393 -3.398136606
C -2.6769641888 -4.1049224267 -2.6991073142
N -2.3464562029 0.1705924084 0.9934002342
C -2.6786243765 1.4199818796 0.3047237221
C -4.091711915 1.1429546739 -0.2260198563
C -4.7314357565 0.2590697369 0.8766583214
C -3.5452121562 -0.236120657 1.7488648853
N -0.0442459223 -8.5874145226 4.0744639599
P -1.3004628609 -9.4371805119 4.4242725336
N -1.042878939 -10.5696471523 5.656094459
C -0.087778315 -11.6846475649 5.4271608306
C 0.4340045512 -12.0553537448 6.8353215611
C -0.545136321 -11.366100236 7.8023800242
C -0.9150040042 -10.0897214973 7.0432147642
N -1.9220676666 -10.4422640272 3.2131986292
C -2.1836678544 -9.8596378486 1.8739078982
C -3.1189968676 -10.8703932876 1.1931977004
C -3.8874380844 -11.4853295234 2.3719142464
C -2.7938565678 -11.616131415 3.4384530831
N -2.5663024008 -8.4773051301 4.9972000299
C -2.3384045663 -7.1400302094 5.585043237
C -3.7521191832 -6.6693995898 5.9388003916
C -4.444308349 -7.9687801775 6.3814032073
C -3.8621538927 -9.0345565633 5.4318836936
N 3.7322246596 -9.0235349265 4.6182016871
C 4.4279155999 -7.8183559967 5.1348817389
C 3.8630772413 -7.6089114105 6.5497440794
C 3.4466259008 -9.0254414912 6.9721314264
C 2.8694733555 -9.6009902864 5.6749935078
N 4.5765417659 -8.7053734597 2.13860519
C 4.732590712 -7.6108568767 1.176904263
C 5.9614885888 -6.8510268176 1.7057281493
C 6.856989311 -7.9591857246 2.3165157067
C 5.9155533319 -9.1902547524 2.4877035543
H 1.0912405686 -2.2235731973 4.4665142141
H 1.304999255 -2.8523443959 2.0242604303
H 0.5814613076 -5.1567731222 1.352552709
H 2.0978362035 -5.1694386971 2.2469865465
H -0.6412905536 -5.7256705081 3.4546854933
H 0.6865416478 -6.4287688743 5.4683810751
H 2.15657057 -5.9087397548 4.6391572622
H -0.2278576716 -4.1158130187 5.3658848085
H 1.3505246731 -4.0867694045 6.1548030915
H 2.4418744836 -3.3016245744 4.1047580931
H -3.5740069369 0.2379111712 2.742921791
H 0.5019403171 2.8980350419 5.2514177699
H -3.5401159655 -1.3175425853 1.9019875543
H -4.021820182 0.5887107818 -1.1679657543
H -5.2837719097 -0.5799909843 0.4413915282
H -5.4398657781 0.8318843509 1.4849133991

H -4.6550155235 2.0629865721 -0.418017967
H -1.9493865881 1.6393512952 -0.4805342162
H -2.6941214092 2.2749564436 1.0060129234
H -0.4027989964 2.8195998518 1.1179154162
H -0.639344478 3.8687755552 3.2844798079
H 1.0686841374 4.2151180503 2.9456458486
H 1.7705045614 2.2130969403 4.2174552057
H 1.8529022475 -0.4788687141 1.4278399045
H 0.2878803371 0.2984882904 4.3560256476
H 2.1110529421 0.6733116494 0.1022425466
H 2.991956492 -1.3674503616 -0.8896883872
H 1.7461776425 -2.3549847622 -0.0917787886
H 0.8029784213 -2.0612812285 -2.3441847579
H 1.2931531681 -0.3546374975 -2.3716904163
H -0.9022163373 -1.6777977613 -0.7161445839
H -0.9842058633 -0.1249277238 -1.5860745813
H -2.9039172743 -1.3208661038 -2.8180168598
H -4.6742585823 -1.3640605295 -2.6929304003
H -4.5781599304 -2.7640845096 -0.7036835033
H -4.7219080135 -3.7385342495 -3.3376260201
H -3.1328917529 -1.7589768445 -0.4565761331
H -3.5161683231 -3.0757281672 -4.4564218118
H -1.680850307 -3.9204697216 -3.1279093922
H -2.8799546662 -5.1782003202 -2.8017653231
H -2.9476421405 -5.1600888763 2.8149828209
H -2.2977558872 -6.6437532843 2.0978774696
H -2.1232418022 -7.2290087281 -1.4032313606
H -1.4044475006 -7.3959492186 0.2170629972
H 0.2857924981 -8.558202097 -0.9585289212
H -0.0835685626 -7.7276738519 -2.4848179067
H 1.4994146283 -6.6409212358 -0.1055910422
H 1.9667891409 -6.5862885892 -1.8195078071
H 0.7825411375 -4.3855203734 -0.6891865286
H 0.2851565072 -4.8955275118 -2.3148273623
H -4.394604508 -7.4898062336 2.9127036696
H -4.2585944803 -7.9469141734 0.5022110704
H -5.8949868879 -7.2782448631 0.6499604578
H -5.2255499721 -5.9497333721 2.6185017733
H -4.0827860019 -6.1497293658 -1.0636888882
H -5.1660189536 -5.101802079 -0.1392653678
H -1.1501027015 1.3247387754 4.4244720543
H 1.255068393 2.3128156632 1.4566970744
H 1.6156144236 -10.2919166231 1.0035727496
H 6.4717064058 -6.2757765444 0.9248256262
H 0.6110278073 -10.8006148787 2.3668692801
H 2.0448734804 -13.1579906483 3.5799897116
H 0.9158414049 -12.9734237649 1.3959851216
H 2.4286979044 -12.4904421873 0.6131260716
H 3.2673390187 -13.8308179156 2.4801681865
H 3.9825463797 -11.7376737821 4.0537159801
H 4.4240520523 -11.6800322172 2.3364209568

H 6.217820534 -9.9939236813 1.7985076968
H 7.6936820897 -8.2075607887 1.6534968423
H 7.2855090511 -7.6461558143 3.2745678592
H 5.6385342463 -6.1482715375 2.4824038678
H 4.2537992082 -6.9487770851 4.491464273
H 3.8209424215 -7.0140481109 1.1364661725
H 5.5121258113 -7.9988729761 5.1652758368
H 4.5909609583 -7.1463773263 7.2252560065
H 2.9796378931 -6.9630561524 6.50612317
H 2.7164442116 -9.0352318475 7.7895081525
H 4.3234335833 -9.6043794915 7.2921376581
H 1.8224603489 -9.2989899774 5.5385618475
H 2.9168868878 -10.6936797396 5.6429079018
H 1.4406356671 -11.6505484398 6.9828331423
H 0.4903697295 -13.1388323795 6.9839152909
H -0.6143780674 -12.5242263293 4.9583243811
H -1.4421136358 -11.9808590706 7.9490440785
H 0.7284656123 -11.3834537584 4.7609162696
H -0.1056543037 -11.1609728332 8.7844647653
H -0.1204836723 -9.3320177911 7.1476145853
H -1.8543571464 -9.6422851446 7.38294826
H -1.2521919991 -9.7011285884 1.3243446675
H -2.6696996119 -8.8809257676 1.9697026211
H -3.73968199 -10.0023026662 5.9354272819
H -4.5154728428 -9.1982738687 4.5628093974
H -5.5374225641 -7.9204960724 6.3337045911
H -4.1664493297 -8.2022913081 7.4173395345
H -4.2413946676 -6.2558029163 5.049795804
H -3.7501966476 -5.8956454041 6.7132590176
H -1.8366316332 -6.4854799177 4.8737845027
H -1.708178396 -7.1957901236 6.4865961288
H -3.7658234364 -10.3967342244 0.4472545993
H -4.6681214634 -10.7940342673 2.7155517878
H -4.3615287083 -12.4437121433 2.133093112
H -2.5317633793 -11.6456235088 0.6838460395
H -3.1779502364 -11.6417921109 4.460264929
H -2.2210864785 -12.5429450235 3.278836497
H 4.9308318796 -8.0183325706 0.1714831384
H 5.9221998506 -9.6043270367 3.4987865644

VIIIbbH^+
C -0.8366473182 0.1468459173 -0.6890016114
N -0.0493074346 0.4759658278 0.5299615273
C 1.387908976 0.6519885138 0.17971234
C 1.4868041861 0.1475517616 -1.2654135212
C 0.1104423637 0.5140720389 -1.8375934881
P -0.4411730385 -0.1665431542 2.0196118348
N 0.3391383961 0.8543721267 3.0732182895
C 0.425198374 2.3252049422 2.9113074122
C 0.4236463078 2.8489989977 4.3527631438
C 1.1608825957 1.7366069312 5.1144091342

C	0.6088718804	0.4537089782	4.4769733679
N	0.0393833673	-1.6282102673	2.4114668583
P	-0.3024935375	-3.1148708958	1.9037862015
C	1.261234868	-4.085181221	1.8983165893
C	0.9946807756	-5.5995592116	1.7436954154
C	0.5053954009	-6.2740683601	3.033092291
C	1.4713083167	-6.0073597882	4.1865126785
C	1.6872841498	-4.4995663522	4.3986705083
C	2.176319115	-3.8107893276	3.1133250447
P	0.1329516088	-8.0951231227	2.6515371278
N	1.6996167591	-8.6620222098	2.4383880297
P	2.7517883963	-9.6102170497	3.0934861189
N	2.1641962683	-11.1491961852	3.408285744
C	1.0898058718	-11.7086452856	2.551509127
C	1.142065726	-13.2243563537	2.8175054675
C	1.7748200504	-13.3239852248	4.2142618716
C	2.8346936525	-12.2177838472	4.1714989059
N	-1.0761700471	-3.1833522837	0.498170124
P	-1.560113548	-4.0100541405	-0.755509885
N	-0.4835391357	-4.3646705441	-1.9803447116
C	-0.0316084955	-3.3080061472	-2.9301449247
C	1.4072657108	-3.7065269123	-3.2962554052
C	1.4185715285	-5.2264778964	-3.0770427114
C	0.5950023451	-5.3681977093	-1.7964041375
N	-2.1519565559	-5.4948442194	-0.2852239611
C	-2.6369358157	-6.5436807153	-1.2056301109
C	-2.8122789402	-7.74454529	-0.271732823
C	-3.393278292	-7.0956791325	0.9937193445
C	-2.6551643216	-5.7475291971	1.0908048607
N	-2.6769129068	-3.0276065919	-1.5328675733
C	-3.6762901217	-2.3058787869	-0.6995135606
C	-4.7987473973	-1.9596273918	-1.6855875001
C	-4.7787027094	-3.1512140196	-2.6545301099
C	-3.2764395368	-3.3863825326	-2.8455812298
N	-2.1140131846	-0.0652519051	2.0493354303
C	-2.8338247993	1.2183732428	1.944130845
C	-4.2993572644	0.7788591942	1.9072058548
C	-4.3196095962	-0.3738283273	2.9254320234
C	-2.960259808	-1.0786321718	2.7314597709
N	-0.4074605788	-8.5780307868	4.204236327
P	-1.6954802993	-9.3777466751	4.6043636832
N	-1.5974618025	-10.0070883959	6.1686324467
C	-0.4774159477	-10.9291381538	6.4972143959
C	0.008365568	-10.4783912895	7.8848362707
C	-1.2394158467	-9.8235955109	8.4961312386
C	-1.8307119507	-9.0764537547	7.2972220801
N	-2.1282032319	-10.7343782684	3.7064386418
C	-2.4685806835	-10.6014350022	2.2751111343
C	-2.7488575735	-12.0434620723	1.837546478
C	-3.3758562958	-12.660397689	3.097382304
C	-2.5401409415	-12.0530385595	4.2337626112

N -3.0787135231 -8.4166751769 4.593275559
C -3.0704096426 -6.9533611475 4.7514769602
C -4.5504586121 -6.5831000416 4.5980014271
C -5.2642408336 -7.761694494 5.2812950857
C -4.4165742821 -8.9808073595 4.8715928225
N 3.49049263 -9.2468196779 4.5788042497
C 4.5586033718 -8.2186562756 4.6295396341
C 4.3603214959 -7.5027402542 5.9782451137
C 3.6268658658 -8.5454061747 6.83508037
C 2.6958456492 -9.2241148961 5.8247042605
N 4.0486480957 -9.6730400701 2.0271613271
C 4.3321543731 -8.6202955177 1.0344591231
C 5.8604672273 -8.6562042306 0.9065075942
C 6.1828294129 -10.1464990495 1.0957106504
C 5.1903396503 -10.5904678178 2.1871400819
H 2.2890882391 -2.7317243953 3.2644474285
H 1.7590832804 -3.7057756186 0.9914813341
H 0.2882050063 -5.7969963682 0.9308471885
H 1.9375325892 -6.0792300706 1.4462121395
H -0.4739407984 -5.8633104825 3.3173662508
H 1.0948147281 -6.4785195592 5.1015180821
H 2.4303161522 -6.4830428038 3.9485138803
H 0.7472567314 -4.0363432426 4.7342843481
H 2.4184955042 -4.3253196932 5.2002501353
H 3.1707291818 -4.2079497293 2.8608368424
H -2.5275533082 -1.395943217 3.687015408
H 0.9953717526 1.7652299573 6.1956572352
H -3.0505960384 -1.9644153704 2.0946464661
H -4.5509993249 0.4134851113 0.9038612631
H -5.1607798388 -1.0593827801 2.7829188976
H -4.3921949933 0.0321226099 3.9413829789
H -4.9900100061 1.5892719507 2.1594632479
H -2.5194832371 1.7698582606 1.0530484985
H -2.6467946376 1.8571895104 2.822297609
H -0.397901967 2.7175191392 2.3084126983
H -0.6072288046 2.9389910048 4.7197949589
H 0.9016264911 3.8295386184 4.4407890285
H 2.2408995455 1.8114994011 4.9358073192
H 2.0334758099 0.1005278854 0.869897348
H 1.3087689776 -0.3852170854 4.5063770453
H 1.6542584314 1.7148174207 0.2407643514
H 2.3230230993 0.5973433527 -1.809652701
H 1.6193654423 -0.942372917 -1.2730726198
H -0.1388458631 -0.0166796568 -2.7618868151
H 0.0629852461 1.590309685 -2.0458235643
H -1.0852020013 -0.918319603 -0.71796747
H -1.7692755117 0.7188575184 -0.7129067252
H -4.5595468165 -1.033088824 -2.2232044519
H -5.760990558 -1.8193845039 -1.1832159034
H -4.0649374512 -2.9540875567 0.0993475884
H -5.2425908084 -4.0282219712 -2.1840262648

H	-3.2232093124	-1.4318717281	-0.2273335595
H	-5.2968556882	-2.9575384636	-3.5988207544
H	-2.8826967292	-2.7155537578	-3.6208548953
H	-3.0283080753	-4.4104053808	-3.1366242431
H	-3.3108996211	-4.9416672574	1.4415934479
H	-1.8129504815	-5.8216440531	1.777876843
H	0.1807625283	-6.367283566	-1.6421675163
H	1.2195618946	-5.1264557708	-0.9270160319
H	2.4243317884	-5.6438345421	-2.969842624
H	0.920605388	-5.7398755738	-3.9090019589
H	2.1184484258	-3.2274696852	-2.6111226021
H	1.670787446	-3.4079020739	-4.3154051494
H	-0.0884876702	-2.313738875	-2.4792770634
H	-0.6769933752	-3.3134149756	-3.8155057865
H	-3.2331315025	-7.6978497121	1.8911608114
H	-1.8354900135	-8.1856200897	-0.0417557765
H	-3.4606307903	-8.5167875153	-0.6980528629
H	-4.4721497979	-6.9330534818	0.8750138187
H	-1.9205440294	-6.7133945491	-2.0137818305
H	-3.6010433192	-6.2627856593	-1.6594686255
H	-0.316767446	0.1373508769	4.9794440379
H	1.3632885131	2.5911219365	2.4051246544
H	1.250381986	-11.4669874207	1.4943243255
H	6.2149299354	-8.256368257	-0.0496929389
H	0.1287259792	-11.2849968123	2.85416908
H	1.0293520151	-13.0985495513	4.9870767719
H	0.1520445269	-13.6884865411	2.7563930037
H	1.7881043283	-13.7198063983	2.0818994296
H	2.2033699071	-14.3083284145	4.4293464694
H	3.1357011049	-11.8674459285	5.1615306939
H	3.7395850832	-12.5834133806	3.6637603395
H	4.8827572568	-11.6332449342	2.0481495638
H	5.983255999	-10.6917001904	0.1646857881
H	7.2230111753	-10.3370809097	1.3785585557
H	6.3163529669	-8.0661582392	1.7124004402
H	4.4966586407	-7.518239597	3.7878003868
H	3.9484023974	-7.6521168557	1.3684062855
H	5.5432227302	-8.7028714337	4.578609783
H	5.3089642303	-7.1730627568	6.4145680934
H	3.7257423085	-6.6191837599	5.8473294631
H	3.0797118018	-8.1045931486	7.6754944621
H	4.3389930598	-9.2753365376	7.2406600632
H	1.7560487689	-8.6701506273	5.6929876063
H	2.4350657283	-10.2416695863	6.1286432823
H	0.8015740518	-9.7291318812	7.7822620322
H	0.4027602597	-11.3086608765	8.4795542724
H	-0.8506903709	-11.9609099504	6.5311664883
H	-1.9426400536	-10.5888351837	8.8482664074
H	0.3097546065	-10.8755686272	5.7383927568
H	-1.0138005695	-9.1554902081	9.333611168
H	-1.3034035377	-8.1204451209	7.1507582368

H -2.89962506 -8.8640658853 7.4061411703
H -1.6547609725 -10.1277440204 1.7175033925
H -3.3668865339 -9.9775758289 2.1447883192
H -4.3779750964 -9.740633071 5.6620076931
H -4.8148744564 -9.4652333429 3.9689975844
H -6.3142376744 -7.864947572 4.9896207427
H -5.2314858507 -7.6318678487 6.3702522784
H -4.8169928381 -6.5423065983 3.5342643101
H -4.7899189905 -5.6138100965 5.0480510205
H -2.4286140285 -6.4904277994 3.9993006825
H -2.6945678876 -6.6477781782 5.7407940864
H -3.3982539232 -12.0942040683 0.9575890125
H -4.4225533542 -12.3427586176 3.1884158354
H -3.3540329958 -13.7549612265 3.1052037892
H -1.8071011355 -12.5509753965 1.5941288729
H -3.0968565183 -11.9539311865 5.1703826492
H -1.6550435424 -12.6738666822 4.434298976
H 3.8454646447 -8.8501306136 0.0748984835
H 5.6331694491 -10.5066028568 3.1904600053
H -1.1249238493 -3.7169210774 2.8807939534

IXaa
C 0.1279484149 -0.8693987268 -0.0844778775
C 0.153589693 -1.0698997503 1.4341862003
C 1.5725455739 -1.1659546328 2.0139395609
C 1.5810960394 -1.375727295 3.5310452087
P 3.2884714881 -1.5555322011 4.2759372506
N 3.8140315456 0.0366944242 3.9857613669
P 4.9939092816 0.8537241447 4.5862927082
N 5.4203493781 2.0485151212 3.4589603252
C 4.4639518142 2.4610769894 2.4347907422
P -1.6002991298 -0.7462647636 -0.7792069129
N -1.2151809122 -0.8294820814 -2.4524233866
P -1.7191738143 0.1084516227 -3.5944086683
N -0.7295874571 1.4824492105 -3.785145145
C 0.6839179479 1.3790467626 -3.4273544701
N -2.0852243087 -2.3457018668 -0.4130243403
P -3.5251404576 -2.8569113346 -0.1002291733
N -4.6065432138 -1.9831066437 0.8918008959
C -5.2391516518 -0.7737161282 0.3510736555
N -4.4738552562 -3.0430770685 -1.5089474873
C -3.7617943075 -3.4124499202 -2.73577158
N -3.3726960904 -4.3019719806 0.7708446517
C -2.1296223444 -5.0673834396 0.7310357716
C -5.8287257766 -3.5876516042 -1.4564910813
N 2.8686128463 -1.554703823 5.9468848785
P 2.8116425266 -2.7711977607 6.9193849328
N 3.4873630065 -4.2885888055 6.5410064922
C 4.9481225006 -4.3877354112 6.4646129448
N 3.6580263271 -2.3851541031 8.3468871915
C 3.8228281201 -0.9782219413 8.7036907386

N 1.2002793579 -3.2223321892 7.2340326208
C 0.1305137925 -2.2468505661 7.0420451505
C 3.6296311719 -3.2784905125 9.4995480633
N 6.5153462667 0.1494599557 4.9203094839
C 7.2653679567 -0.340526347 3.7570996285
N 4.6199131312 1.5102981519 6.1156333295
C 3.2237208279 1.8715202559 6.3681931216
C 6.5993215295 -0.7902818536 6.0453359018
C 5.5669408539 2.3195423163 6.877007475
C 6.4163403291 3.062413256 3.7803768481
N -3.2571766267 0.8449992111 -3.5751459178
C -3.5006298225 1.9150576519 -2.5986514035
N -1.8136909406 -0.8149532212 -5.0187137978
C -1.0272287416 -2.0404930925 -5.143037157
C -4.4254513576 -0.0277244706 -3.731448124
C -2.1006004863 -0.1759383978 -6.2975538191
C -0.9973540565 2.535494572 -4.7595757347
C 0.8337647991 -4.3223027333 8.1182340635
C 2.8339521099 -5.0981443017 5.504891752
C -4.5320015207 -5.0714636237 1.2012327765
C -4.1757898371 -1.7857194894 2.2816877006
H 2.1069904196 -1.9920852226 1.5202358851
H 0.6585003171 0.0552680891 -0.3549538561
H 1.0256745624 -2.2896086315 3.7920346028
H -0.378449683 -0.2410287021 1.9274679593
H -1.3111417124 -4.4140596727 0.4278991945
H -1.9186048269 -5.4727859623 1.7311419278
H -4.3205733497 -5.5345580043 2.1756253271
H -5.400987232 -4.4212438497 1.3192396288
H -3.7205282313 -2.6987920816 2.6699009819
H -3.4463786398 -0.9660255692 2.372077983
H -5.053750633 -1.5422078135 2.893911198
H -6.125983485 -0.5396712133 0.9552046704
H -4.5570212452 0.0889804714 0.3738879004
H -5.554299674 -0.9351971662 -0.6802567818
H -6.3332438543 -3.2754830041 -0.5400547387
H -6.4034617981 -3.2069218799 -2.311995278
H -4.6524640673 -0.5877594171 -2.8123251867
H -5.2937454892 0.592061858 -3.9898308789
H -4.3531710464 -3.0883964592 -3.6013270559
H -3.6116804145 -4.5031006562 -2.8125927873
H -2.7938227408 -2.9127617589 -2.7688362696
H -4.3010312864 2.5662882635 -2.9762211197
H -2.5990649145 2.5127215621 -2.456359374
H 1.0197751777 2.3248930819 -2.9800837008
H 0.8260924629 0.5760412207 -2.7039523047
H 1.3129221736 1.175489609 -4.3105601777
H -0.662650167 3.5002564945 -4.3528354307
H -0.06365934 -1.8577106377 -5.6481816676
H -1.5905184151 -2.7741218892 -5.7368683128
H -2.066685007 2.6050048053 -4.965465326

H	-2.7764812585	0.6708411543	-6.1597644289
H	-2.5938096529	-0.8998187587	-6.9605085964
H	-1.1878688122	0.1782741232	-6.805527096
H	-3.7986986808	1.5126027585	-1.620509919
H	-0.4640868625	2.3682606463	-5.7098945134
H	-2.1961407877	-5.9123498824	0.0255807672
H	-4.2557649884	-0.7441559235	-4.5366624237
H	-0.8304550776	-2.4497269583	-4.151991774
H	-4.7846227237	-5.8787561575	0.4939544382
H	-5.8396953086	-4.6891103718	-1.5070209967
H	0.4625249901	-1.4706684126	6.3524092695
H	-0.7511230042	-2.7474176413	6.6171539491
H	-0.0663609914	-4.8190663326	7.7285976484
H	1.6349921542	-5.0620861248	8.162595166
H	1.7502148238	-4.9816317838	5.5579143309
H	3.167623656	-4.8099987847	4.4969964234
H	3.0820663259	-6.1555002085	5.6694962765
H	5.2427959109	-5.4342815023	6.6196998045
H	5.3249700097	-4.0604639191	5.4834705524
H	5.4092287045	-3.7754540702	7.2415481993
H	3.6108836007	-4.3206152812	9.1736338584
H	4.5394471237	-3.1256520608	10.095538361
H	6.220057787	-1.7865242071	5.7762840203
H	7.6505295263	-0.8869608852	6.3477001657
H	4.7554503317	-0.8582239131	9.2728373011
H	2.9921272992	-0.609518994	9.3301075026
H	3.8772979638	-0.3770223227	7.7967658569
H	8.3171774143	-0.4690250444	4.0432982842
H	7.2097813885	0.3813318323	2.9403287759
H	5.0073855076	2.7145238158	1.5137513131
H	3.7741056753	1.6421840455	2.2309250171
H	3.8854893874	3.3469046247	2.7470872814
H	6.9274334591	3.3725907786	2.8585514861
H	2.9823236811	2.8803840328	5.9917743039
H	3.0443299394	1.8616658838	7.4513041616
H	7.1674096972	2.6576058926	4.4619313448
H	6.5920503647	2.0090855823	6.6675003424
H	5.3790659306	2.1805487325	7.9511747708
H	5.4720338407	3.3954718483	6.6557640313
H	6.8797406332	-1.3074768846	3.397089096
H	5.9702717846	3.9621834903	4.2365797075
H	-0.1681013257	-1.7770225498	7.9944308215
H	6.0218171553	-0.4273711645	6.8946817196
H	2.5639737062	1.1397338226	5.9036418125
H	0.6108295136	-3.9787757663	9.1416935469
H	2.7623026884	-3.0956259379	10.1560739339
H	1.088107168	-0.5336943365	4.0393656591
H	-0.4042155994	-1.983781474	1.6799709656
H	2.1318684155	-0.2510570131	1.7756250896
H	0.6284964729	-1.7032605919	-0.5978184877

IXaaH+

C	-1.0198628119	0.4852191123	0.3272236233
P	-0.2896827438	0.348043224	2.0522947894
N	1.3405398038	0.2756989449	1.5277123369
P	2.6256243336	0.6959809486	2.3096387677
N	3.100150944	-0.4592336154	3.4618627412
C	4.2985371468	-0.3007984096	4.2855453081
C	-0.5746427593	1.7585153932	-0.3944992309
C	-0.8717243368	1.7466153753	-1.9005467051
C	-0.2832974806	2.9869580123	-2.593622212
P	-0.2401329329	2.8830242629	-4.4058820151
N	0.7692224172	3.9591733716	-5.0678130466
P	2.3533492886	4.0581569822	-5.0344126679
N	2.8155870307	4.6925654837	-6.5155344095
C	4.2174386377	5.0522432902	-6.7488269629
N	-1.746803588	2.9815256049	-4.9556105355
P	-2.2719341946	2.9413333311	-6.4604459062
N	-1.6476826164	1.7949070024	-7.5199161801
C	-0.2780339592	1.9417152682	-8.0307873337
N	-1.9090863846	4.3844119528	-7.254300324
C	-2.2471113497	4.5767484386	-8.6705468499
N	-3.897326577	2.5711533267	-6.3709098093
C	-4.7178131621	2.5651377263	-7.5849155377
C	-1.9592274196	5.6372975063	-6.4887562894
N	-0.6964044148	-1.2823232234	2.3536110897
P	-1.3346309802	-1.8857844224	3.6524872536
N	-1.0192095659	-1.2621599014	5.2019591093
C	0.3142592269	-1.4711153072	5.7745306771
N	-0.7700309988	-3.4771113204	3.8043874606
C	-1.3193956228	-4.3581351666	4.8318065847
N	-3.033253089	-1.7948286045	3.6251201499
C	-3.8748127233	-2.2491437177	4.7310663476
C	-0.2660648444	-4.1934951502	2.6336046808
N	2.8453851523	4.9381890058	-3.686870324
C	4.2645911946	5.1519943805	-3.3774926457
N	3.2739277041	2.658949897	-4.8966480032
C	3.3624927651	1.9528861164	-3.6096022083
C	1.9821135061	6.0033249443	-3.1613260341
C	3.3950025966	1.7528905357	-6.0454629115
C	1.8646884096	5.4237512908	-7.3591905359
N	3.8338930681	1.0030029168	1.1478246183
C	5.1946771503	1.3373125423	1.557460051
N	2.6998158984	2.127546845	3.2330645865
C	2.5304314595	3.3852943424	2.4973498456
C	3.7638574007	0.3459128501	-0.1535817574
C	2.0087782603	2.1680752232	4.5289376063
C	2.7790721976	-1.8630439371	3.1833156987
C	-3.7165414189	-1.8440866908	2.3336863411
C	-1.5741772598	0.0503567628	5.5576683311
C	-4.6663424535	2.6974841362	-5.1279691082
C	-2.04682723	0.3882169611	-7.3757981442

H	-0.4305800654	0.8365385185	-2.3334963711
H	-2.1142753455	0.4373216977	0.4068282586
H	0.7562979479	3.1406872934	-2.2734766609
H	-1.0426452111	2.6416495828	0.0656172872
H	2.7213203122	0.1408878244	-0.4003376858
H	4.2002156777	1.0093737681	-0.9152382349
H	5.6599436995	1.9670094361	0.786403469
H	5.1833704159	1.9012442078	2.492021909
H	3.0742959876	3.3448616222	1.5515378224
H	1.4699156244	3.597298079	2.2871302623
H	2.9352850971	4.2071446461	3.101295972
H	2.4406142632	2.9753298237	5.1342053139
H	0.9311033531	2.3524959822	4.4094215221
H	2.1397592965	1.2270266387	5.0629406809
H	4.5005857563	0.7548650798	4.4742880883
H	4.1398311538	-0.7983482381	5.2516094349
H	1.0586878306	-0.7790457403	5.3540614475
H	0.2594830178	-1.31198755	6.8585892875
H	2.6949480486	-2.4032959482	4.1337725321
H	3.5639325421	-2.3504567614	2.5812198381
H	1.8256207737	-1.9312698975	2.6603137419
H	-1.7086713963	0.0979542601	6.6464054358
H	-2.5424448943	0.1978020347	5.077453492
H	-4.6010290765	-1.1930659316	2.3625772332
H	-3.0470155229	-1.4980145549	1.5459729619
H	-4.0522674302	-2.8657341487	2.0891786896
H	-4.7736195034	-1.6198002786	4.7832798453
H	-1.0526815381	-4.8042845424	2.1602266085
H	0.5472543628	-4.8642244354	2.9427184313
H	-3.3427792558	-2.1629938974	5.6794676755
H	-1.5856108639	-3.7884984335	5.7244982083
H	-0.5597291148	-5.0981425048	5.1154478644
H	-2.2099179611	-4.9047835466	4.4816066712
H	-0.9107231941	0.8688423738	5.2453102616
H	-4.2005931048	-3.293406252	4.6014392769
H	4.3267184267	-0.6026124417	-0.1708850428
H	0.6503657235	-2.4919038989	5.588425156
H	0.1110353111	-3.4791403475	1.9019152143
H	5.8270039892	0.4442406564	1.6883200635
H	5.186916662	-0.7521778776	3.8160592848
H	0.9386663063	5.7962617092	-3.3980483294
H	2.0938100705	6.0481573045	-2.0715811828
H	4.3875547503	5.2032311752	-2.2890656439
H	4.8714941237	4.3261108999	-3.7511037177
H	3.244265181	2.6457348304	-2.7766093999
H	2.5946103306	1.1700937017	-3.5248066968
H	4.3456511745	1.4752361643	-3.5288208732
H	4.3621160645	1.239539469	-5.9954596745
H	2.6007108302	0.9910292434	-6.0416464179
H	3.3420309673	2.3121141188	-6.9801864611
H	4.8817424777	4.398379031	-6.1800973587

H 4.4471979815 4.9246718533 -7.813046532
H 0.4433309242 1.4033380156 -7.3982589042
H -0.2242450437 1.5242357604 -9.0432329264
H 2.1087330504 5.2359817097 -8.4122049022
H 1.917902373 6.5083963791 -7.1812799691
H 0.850169669 5.0793295302 -7.158914703
H -1.974008613 -0.1023370851 -8.3531430723
H -3.0760711025 0.3163615773 -7.0232621175
H -5.3783745955 1.8656611675 -5.0639880357
H -3.9963206435 2.6586449576 -4.2700299632
H -5.2299355465 3.6413194999 -5.1033264901
H -5.4794242773 1.7809585547 -7.4995269808
H -2.977969376 6.0520538466 -6.4496127024
H -1.3085692351 6.3717572498 -6.9768700986
H -4.1074530002 2.3472566903 -8.4635409789
H -2.1828639064 3.6338618984 -9.2152264767
H -1.5301491072 5.2788477063 -9.1129297576
H -3.2569583922 4.9929638091 -8.7988366879
H -1.3948724015 -0.1462367529 -6.6687802653
H -5.2296035777 3.5266146362 -7.7355483947
H 2.2533571782 6.9846167436 -3.5784133781
H 0.0113417288 2.9916043156 -8.0642440867
H -1.5945067321 5.4784313527 -5.4752907487
H 4.6380763707 6.0911496017 -3.8102517851
H 4.4246560792 6.0970966222 -6.4749559495
H -0.8379840118 3.8965354994 -2.3300949109
H 0.5096226796 1.8687701165 -0.2562344699
H -1.9512618031 1.6898654353 -2.0883621109
H 0.2749019379 1.5796701935 -4.6419244258
H -0.6813392391 -0.4086701919 -0.2166918534

IXbb
C 0 0.1533817363 0.4270069746 0.965968093
N 0 -0.5308982648 -0.0163881237 2.1973719
C 0 0.4277797818 0.1248419202 3.300695581
C 0 1.0734932543 1.4838889173 2.999816031
C 0 1.1327095064 1.5300843593 1.451027005
P 0 -1.664893109 -1.2694957183 2.11837262
N 0 -0.9116536505 -2.7713827112 2.2469689
C 0 -1.2200857471 -3.8661362223 1.2985408
C 0 -0.4878212694 -5.0803761582 1.8937800
C 0 -0.4905906688 -4.7908187118 3.4033125
C 0 -0.2228855305 -3.2801367973 3.4460225
N 0 -2.6161890463 -1.2807790907 0.9014082
P 0 -3.1927113909 -0.5900319506 -0.527587
N 0 -2.8287141508 1.0909270692 -0.4813185
P 0 -3.8916216831 2.1974423567 -0.1447093
N 0 -5.5226116727 1.7893120179 0.09180782
C 0 -6.3442678491 1.4889922001 -1.1031866
C 0 -7.5460504151 0.7265238278 -0.5341367
C 0 -6.9094034418 -0.0938458842 0.5980496

C	0	-5.9046286464	0.8918589768	1.21209044
C	0	-1.8826010405	-1.1246534931	-1.756848
C	0	-2.2610540874	-0.711867758	-3.1820309
C	0	-1.4684802306	-1.4163778708	-4.292242
C	0	-2.0099775664	-1.0640640171	-5.692763
P	0	-3.2575928098	-2.2782383501	-6.424095
N	0	-4.0395473491	-1.1633175057	-7.479474
P	0	-4.3081064146	-1.2902794733	-9.013331
N	0	-3.8610271402	-2.6847919564	-9.872490
C	0	-2.4275515339	-2.848423912	-10.217888
C	0	-2.2823415344	-4.3544312657	-10.46926
C	0	-3.2471608118	-4.9574859373	-9.437326
C	0	-4.4425090717	-3.9959483485	-9.487700
N	0	-4.2864906126	-2.4937672849	-5.104574
P	0	-5.7647453569	-2.5202673906	-4.648630
N	0	-6.5811626119	-1.0623640052	-4.870340
C	0	-7.9909064767	-0.8090524362	-4.554472
C	0	-8.2310917528	0.5724440047	-5.1726958
C	0	-6.910707785	1.3108622292	-4.87676878
C	0	-5.8215418825	0.2098028251	-4.8701612
N	0	-6.8934329368	-3.5758850394	-5.374838
C	0	-7.2524309934	-3.3400807004	-6.794119
C	0	-7.5258585706	-4.7388670786	-7.371527
C	0	-6.6052793872	-5.6420943978	-6.538207
C	0	-6.7247430096	-5.0282709796	-5.137372
N	0	-5.7874392762	-2.9955355321	-3.039430
C	0	-4.6417222136	-3.6578807548	-2.383808
C	0	-5.1355175904	-3.8858550403	-0.949862
C	0	-6.6441769101	-4.1157634521	-1.133716
C	0	-7.011690797	-3.1032343969	-2.2306059
N	0	-5.9506928685	-1.1992967827	-9.357270
C	0	-6.8214166675	-0.331637901	-8.5428967
C	0	-8.2489268293	-0.5858340264	-9.098722
C	0	-8.0843766812	-1.7379058094	-10.11304
C	0	-6.6391738631	-1.5640053105	-10.59583
N	0	-3.5270505917	-0.042161285	-9.8232530
C	0	-3.7647648721	0.2872442345	-11.237038
C	0	-2.7375771692	1.3939091026	-11.535433
C	0	-1.5791798478	1.0608167077	-10.581613
C	0	-2.3042392836	0.6051143164	-9.3079799
N	0	-2.3970800888	-1.0656711037	3.6460328
C	0	-3.0255225233	0.261693402	3.882355750
C	0	-4.3410889657	-0.0371204236	4.6251780
C	0	-4.7054057049	-1.4492716767	4.1429983
C	0	-3.3342808857	-2.1315284255	4.0737198
N	0	-3.5564660218	2.9871609787	1.30072447
C	0	-2.1630141844	3.1441751388	1.76593948
C	0	-2.2888564101	3.8932484117	3.11831786
C	0	-3.7966039125	3.8825165454	3.44143113
C	0	-4.4381397418	3.8873368398	2.04971554
N	0	-3.9116214495	3.3208341443	-1.3921200

C	0	-4.5824327257	4.6268666989	-1.3141420
C	0	-4.3688447416	5.2293630793	-2.7124844
C	0	-4.3554660664	3.9909920117	-3.6218689
C	0	-3.5890481241	2.9539339511	-2.7890660
H	0	-0.406381969	-1.1407458404	-4.2094692
H	0	-2.1339632791	0.3738854751	-3.2853316
H	0	-3.3233042031	-0.9193455558	-3.352304
H	0	-1.1959076067	-1.0227379213	-6.431145
H	0	-1.5149231833	-2.5040015301	-4.131536
H	0	-4.4003573646	-4.6135375392	-2.875851
H	0	-3.7539139845	-3.0301240972	-2.434487
H	0	-4.6253227532	-4.7261987481	-0.465835
H	0	-4.95023946	-2.9851852976	-0.35380924
H	0	-7.2247806244	-3.9646187912	-0.216628
H	0	-6.8326703412	-5.1373573371	-1.489907
H	0	-7.2654757823	-2.1319434734	-1.782213
H	0	-7.8659003278	-3.4265942268	-2.838183
H	0	-8.1724781498	-0.7703440794	-3.467551
H	0	-8.627555295	-1.5958903978	-4.9678702
H	0	-8.3868399025	0.4734008504	-6.2523288
H	0	-9.1070412248	1.077175616	-4.74935483
H	0	-6.6928322993	2.0958804427	-5.6085778
H	0	-6.9699352174	1.788634647	-3.89289310
H	0	-7.6059477558	-5.4249688253	-4.609651
H	0	-5.1814657309	0.2950182339	-3.9834117
H	0	-5.8478757608	-5.2334750472	-4.517604
H	0	-5.1750289264	0.2391826048	-5.7514143
H	0	-6.4326316936	-2.8525875987	-7.331648
H	0	-8.5748950636	-5.0216610455	-7.212954
H	0	-8.1271119834	-2.6834283624	-6.872036
H	0	-7.3254913143	-4.7853067204	-8.447477
H	0	-5.5692075054	-5.5549885321	-6.888600
H	0	-8.9592654949	-0.8324259248	-8.302603
H	0	-8.6255916764	0.3115307215	-9.6034267
H	0	-8.1831941864	-2.7072438382	-9.611111
H	0	-8.8146589199	-1.6972459112	-10.92881
H	0	-6.5870922989	-0.7636237224	-11.35487
H	0	-6.2049720025	-2.4654201756	-11.03261
H	0	-5.1766575445	-4.3143077756	-10.24359
H	0	-4.9571337608	-3.9357347674	-8.526409
H	0	-2.5633271852	1.4597549217	-8.6666558
H	0	-2.7974732797	-4.9194655641	-8.438511
H	0	-1.7120310445	-0.0847069046	-8.701443
H	0	-1.7747741481	-2.5433189223	-9.387170
H	0	-1.2485395072	-4.695188133	-10.349561
H	0	-2.6072549265	-4.6012329157	-11.48896
H	0	-0.9828744787	0.2336871787	-10.988901
H	0	-0.9069591937	1.9071218887	-10.403878
H	0	-2.444663297	1.4178585111	-12.5905478
H	0	-3.1603106962	2.3742609421	-11.280532
H	0	-3.6052214332	-0.5890884712	-11.88541

H	0	-4.7938672584	0.6302174628	-11.395617
H	0	-3.5310669142	-5.9925033878	-9.657832
H	0	-2.17101451	-2.2423718146	-11.0939493
H	0	-6.7141552444	-0.598292063	-7.4904121
H	0	-6.5426997636	0.728644364	-8.64557684
H	0	-6.8894658421	-6.7000211583	-6.566442
H	0	-2.4783663131	-0.0695823223	-5.686686
H	0	-2.3035826733	-4.0370737104	1.2430221
H	0	-0.880263521	-3.6215649473	0.28744355
H	0	-0.9739949628	-6.0255436012	1.6297047
H	0	0.5454608818	-5.1192344338	1.52481199
H	0	0.2532382572	-5.3695701444	3.96191732
H	0	-1.4801496296	-5.0061487497	3.8278046
H	0	0.8590611594	-3.083044431	3.390952533
H	0	-0.6028936605	-2.7987166685	4.3524210
H	0	1.1910776524	-0.6732509719	3.27899021
H	0	-0.082950378	0.078798024	4.2658992849
H	0	0.4292913815	2.2837314098	3.381757782
H	0	2.0571887104	1.5924239106	3.469917433
H	0	0.8389089999	2.5136838258	1.070363776
H	0	2.146063031	1.3307457052	1.0861073481
H	0	-3.0366274042	-2.5092000677	5.0640372
H	0	0.7100624014	-0.4033558622	0.50155775
H	0	-3.3163876292	-2.9740886231	3.3762586
H	0	-0.584567526	0.795325946	0.2511001279
H	0	-3.229586743	0.7683599189	2.932800363
H	0	-4.1791164048	-0.0398612561	5.7109636
H	0	-2.3561604678	0.9089195465	4.46181580
H	0	-5.1129207844	0.7081984061	4.40388422
H	0	-5.1426948724	-1.4091771808	3.1385690
H	0	-1.689585515	3.4183779137	3.902290825
H	0	-1.934829432	4.9260288903	3.016740838
H	0	-4.0716364996	2.9608505576	3.96658306
H	0	-4.1089197264	4.7337179274	4.05659650
H	0	-4.4359512033	4.9116612427	1.63903360
H	0	-5.4680685439	3.5254498372	2.03314021
H	0	-6.3695544836	1.4795359579	2.01861374
H	0	-5.0344768332	0.3803174185	1.63018450
H	0	-2.5057783803	3.0189336753	-2.9582342
H	0	-6.365705373	-0.9471444731	0.17785219
H	0	-3.8890719482	1.9285472508	-3.0147859
H	0	-5.8028558207	0.8516310015	-1.8152076
H	0	-8.0404579373	0.1111866041	-1.2928958
H	0	-8.286850218	1.4294641303	-0.13037512
H	0	-5.3810599481	3.6447418172	-3.7994455
H	0	-3.8891516041	4.1707291766	-4.5963139
H	0	-5.1423994065	5.958951059	-2.97551605
H	0	-3.396404988	5.7365833812	-2.75427630
H	0	-5.656636129	4.5175390832	-1.09079207
H	0	-4.1430334526	5.2494958034	-0.5272373
H	0	-7.6354800011	-0.4645490265	1.3301355

H 0 -6.6283034926 2.4155848559 -1.6143453
H 0 -1.7041190455 2.1613879521 1.87491960
H 0 -1.5591574231 3.7194652019 1.04869588
H 0 -5.4074857661 -1.9670111372 4.8063396
H 0 -1.8218624141 -2.2193900306 -1.677978
H 0 -0.9019190605 -0.7244403857 -1.467358

IXbbH^+
C -0.2166115862 0.8985744092 0.1576818896
C -0.0997249834 0.9797690696 1.6879965725
C 1.3528524941 1.1744688044 2.1453295155
C 1.5386307813 0.9447911467 3.6486758392
H -0.4646810396 0.0372031749 2.1078477764
H 1.9666860351 0.4348768271 1.6176039819
H 1.72153185 2.1674075145 1.8504729158
H 0.5786341005 0.254996634 -0.237199515
H -0.7474055909 1.7762750409 2.0817346664
P -1.7815884824 0.1807985516 -0.4448305788
N -1.7794409404 -0.0113886049 -2.0403869164
N -2.0961088855 -1.0955515158 0.4906618223
P -1.1893247444 -0.9320039846 -3.1867111119
P -3.4680075143 -1.8509782836 0.7888438599
N -3.6888108833 -3.1571745445 -0.2186267169
C -2.5471755481 -4.0896462561 -0.4114126129
C -3.2141381783 -5.4578765548 -0.602460869
C -4.5580528277 -5.1012695948 -1.2551883928
C -4.9800382749 -3.8202952128 -0.5201634557
N -3.3616212005 -2.3449708207 2.3655130329
C -4.2527616843 -3.360662022 2.9577103611
C -3.6186738149 -3.6309510097 4.3297521576
C -3.015431476 -2.2694698284 4.7099798438
C -2.4692503959 -1.7295937295 3.3803592647
C -5.8909254022 0.9298859204 -0.3849448131
C -6.3797665967 0.8079260599 1.0664291983
C -5.3037346145 -0.0688922063 1.7190389573
C -4.359574494 -0.3355479891 -4.7359487796
C -4.7579707421 -1.7397567153 -4.2522979963
C -3.4439017407 -2.3399657674 -3.6945040142
N -2.343787928 -1.5069694953 -4.2602957577
C -2.931568181 -0.5629713379 -5.2428104043
N -0.1144611367 -0.0214822898 -4.0642768158
C 0.0804920883 1.4401523717 -3.9313465817
C 1.3265405679 1.6998049971 -4.7879485048
C 1.1944112614 0.6664529696 -5.9176966321
C 0.6684988855 -0.5800387769 -5.1892034286
N -0.4603993793 -2.3400384443 -2.6606513154
C 1.5200627414 -3.4426577188 -1.9021186834
C 0.5982996933 -4.452616076 -2.599543081
C -0.2840902126 -3.5628132136 -3.4834449774
H -0.7887470332 1.9911877039 -4.3189885516
H 0.2126603267 1.7204541228 -2.8838681179

H 1.3733524188 2.7316090529 -5.1487155817
H 2.2324060575 1.5042115634 -4.2001737451
H 2.1330445626 0.4744593357 -6.4459394203
H 0.4566649355 1.0077586927 -6.6545848542
H 1.4957251007 -1.1951873846 -4.807421138
H 0.0454777862 -1.2126408246 -5.8319185688
H 0.2142672345 -3.3434164582 -4.437047637
H -1.250137743 -4.0198007888 -3.7099475989
H -0.0168042665 -4.9800606805 -1.8605435708
H 1.1405230973 -5.2028997073 -3.1830554203
H 1.9809076082 -3.8286554137 -0.9885437069
H 2.3264903249 -3.1422067692 -2.5812548341
H -2.9387298558 -1.0382211959 -6.233277891
H 1.140953528 -1.2982577143 -1.6953572968
H -2.337320589 0.3507069269 -5.3138807365
H 0.1432354815 -2.3031360876 -0.6239939615
H -3.4193649225 -2.3093531849 -2.6010351557
H -5.1199599074 -2.3383602104 -5.0961465624
H -3.3207077422 -3.3845328722 -4.0020727992
H -5.5506129089 -1.7242515548 -3.4976056973
H -4.3387787234 0.3717529278 -3.8980210875
H -2.605629974 -6.1363521954 -1.20886731
H -3.3821675595 -5.9371484282 0.3700549344
H -4.4145125872 -4.8900505508 -2.321814029
H -5.3091191187 -5.8919181676 -1.1674984863
H -5.5409968139 -4.0534269182 0.3939439084
H -5.6100138459 -3.1792222026 -1.1419414548
H -6.1913169246 -1.1185432492 -1.0540329036
C -5.3744486795 -0.4829299085 -0.6884978385
H -4.5795430869 -0.4936940713 -1.4407505959
H -1.4339251967 -2.0420178042 3.2060744515
H -5.0714164086 1.6564626664 -0.4493836694
H -2.4981564083 -0.637546498 3.3295084812
H -4.4579402035 0.5476797176 2.0572063154
H -6.4883570024 1.772121877 1.5717129665
H -7.3503889313 0.2977795254 1.0968394192
H -3.79995751 -1.6050379269 5.0929553037
H -2.234695642 -2.3405974607 5.4732243239
H -4.3458974715 -4.0026540849 5.0577541126
H -2.8261791951 -4.3824265235 4.2291228824
H -5.279949468 -2.9782596016 3.0617368975
H -4.28969203 -4.2587110503 2.3324384269
N -4.8966099854 -0.9924108316 0.6256703307
C 0.5968732437 -2.2470025283 -1.6201860194
H -6.6738832719 1.2437761772 -1.0820701706
H -5.6746411199 -0.6336957734 2.5795299666
H -1.9758953242 -3.7823660257 -1.2917833835
H -1.8714429152 -4.0671473366 0.4506325985
H -5.0294990311 0.0581991886 -5.506972583
H -0.0979453847 1.878517897 -0.3228526856
P 3.3088032699 0.479218205 4.1167494465

N	3.0612111967	-0.0814975658	5.6823708534
N	3.434277091	-0.8955676118	3.0809409424
P	2.5801619511	-1.150113478	6.6927221657
P	4.7953311001	-1.5681158407	2.6695065248
N	5.0945692633	-3.0287096637	3.4397744379
C	3.979547178	-3.9935515432	3.5736945463
C	4.6729489757	-5.3625073941	3.6346635736
C	6.0337075304	-5.0331530464	4.2668744472
C	6.4021338162	-3.6906638283	3.6184714677
N	4.7136254684	-1.8927411089	1.0191590069
C	5.6964536228	-2.7345869024	0.3202048185
C	5.2236077604	-2.7099535329	-1.1411464847
C	4.6002774459	-1.3114167266	-1.278243449
C	3.9117283545	-1.0901285617	0.078463832
C	7.1544967949	1.0410239714	4.199186463
C	7.593692204	1.2062590688	2.7364007544
C	6.5510255042	0.3747773441	1.9782876332
C	5.9853710548	-1.1157438601	8.1965885968
C	6.0508405739	-2.5867344974	7.7599344323
C	4.7280113927	-2.7803206528	7.002624108
N	3.7712364319	-1.8595472008	7.6714707679
C	4.5106982924	-0.95094684	8.5851642423
N	1.5275192173	-0.4381519058	7.7979344638
C	1.217438921	1.0106900676	7.7369699647
C	0.4959033872	1.291111495	9.0669468102
C	1.0840613335	0.239989388	10.0211728965
C	1.180092993	-0.9966815652	9.1193288904
N	1.8913194939	-2.5645762999	6.0500507742
C	0.5511931999	-3.9443604307	4.6357243917
C	0.9931638386	-4.7226375155	5.9026866977
C	1.3331198418	-3.6162080284	6.9108015412
H	2.1399863853	1.5972743547	7.6465065261
H	0.5975132953	1.2460679299	6.8651917854
H	0.6457770221	2.3210244977	9.4066568871
H	-0.5844108503	1.1282614939	8.9570464726
H	0.4724560669	0.0687473118	10.9133678633
H	2.0876013443	0.5423367598	10.3471731292
H	0.2095385895	-1.5152526588	9.0805668116
H	1.930921081	-1.7199776291	9.4491301523
H	0.4173173884	-3.273018526	7.4242767045
H	2.0547044014	-3.9179583572	7.6737712622
H	1.8908330448	-5.3149258538	5.6937680747
H	0.2228821522	-5.4056112175	6.2770297394
H	0.9962678414	-4.3701257693	3.7306294354
H	-0.5357058029	-3.9799277728	4.5069646203
H	4.3438287161	-1.2603172565	9.6272337426
H	0.158825961	-1.828319179	5.0491919557
H	4.1644846039	0.0821030258	8.4820219941
H	1.5933056376	-2.0797839527	4.0204702984
H	4.848594996	-2.5209931831	5.9442155963
H	6.0894865198	-3.240574702	8.6406281065

H	4.363287182	-3.8129712272	7.0508867591
H	6.9195913143	-2.8164348124	7.1332520646
H	6.2184708658	-0.4578484884	7.3509280902
H	4.0954259612	-6.0965297244	4.2071591857
H	4.8170180961	-5.763910052	2.622924076
H	5.9231457363	-4.9057777638	5.3505410906
H	6.7946315673	-5.800694344	4.0925536547
H	6.9223657445	-3.8454502498	2.6625514237
H	7.0601254657	-3.0953007026	4.2561045814
H	7.6221553489	-1.065532492	4.5008216582
C	6.7516263939	-0.4377317207	4.2635142913
H	5.9810820798	-0.6269361472	5.0160686867
H	2.8714470084	-1.4463775542	0.068774215
H	6.2791688416	1.6672427875	4.4035004901
H	3.8877050845	-0.0348236907	0.3656057017
H	5.6498421945	0.9787969855	1.7980159215
H	7.6109163811	2.2487587332	2.4026676965
H	8.5960439394	0.7842232935	2.5869434305
H	5.3897013287	-0.5634199478	-1.4236057691
H	3.9028995956	-1.2273827427	-2.1200638518
H	6.0389628896	-2.894415421	-1.848021578
H	4.4604103712	-3.4820489879	-1.2973908412
H	6.716070735	-2.3279967749	0.4142344738
H	5.7050960131	-3.7491501887	0.7333807337
N	6.2658627431	-0.7603436977	2.8931866104
C	1.0266858566	-2.484383575	4.8618050629
H	7.9416923963	1.2896991212	4.9189213331
H	6.9174073803	0.005848214	1.0132967137
H	3.4174497203	-3.7823381463	4.4878201839
H	3.2864790086	-3.9125758107	2.7273383348
H	6.669071263	-0.8741482813	9.0172636968
H	1.2663296975	1.8316545783	4.2357851077
H	0.891792742	0.1246829085	3.9837563662
H	-2.7499384689	1.1853301381	-0.2267450846

Cartesian coordinates of neutral and protonated forms of monosubstituted analogs of compounds **Ia**-**IXa**

Ia

C -0.0452599654 0.1206169597 -0.0578409678
C -0.0728821988 0.0141485616 1.3628387956
C 1.1716396965 -0.1020653099 2.0654640535
C 2.3837517756 -0.0999225077 1.3192961084
C 2.3897968235 0.0226857559 -0.0551710063
C 1.1479645903 0.1245918662 -0.7439297111
C 1.1512596492 -0.2055479112 3.4833659027
C -0.0404184935 -0.1918176732 4.1786641309
C -1.2708033236 -0.0768960285 3.4831207018
C -1.2837150426 0.0221244464 2.1067168629
H -0.0405090551 -0.2706076616 5.263055019
P 4.031621753 0.1357533203 -0.9404876115
N 4.0774943668 1.7916558133 -1.3304449047
P 3.9687230627 3.2059325505 -0.70959192
N 2.4742824791 3.8553163818 -0.2197833401
C 1.4649018949 4.0449735591 -1.2672713092
N 3.5570993248 -0.5857573121 -2.4018858749
P 4.3367966192 -0.6783973039 -3.7503393101
N 6.0168139785 -0.9725522031 -3.7974547827
C 6.4595700344 -2.2764933234 -3.2867609885
N 3.7300349723 -2.0165062149 -4.5934815703
C 2.4096973408 -2.5520075823 -4.2691623586
N 4.2496091931 0.7622226694 -4.6557507743
C 3.0367954366 1.5724082451 -4.525341773
C 4.1761660227 -2.3172079766 -5.948251353
N 4.8764875656 3.4047830335 0.7189511803
C 6.0920479046 2.609169121 0.8844805419
N 4.4103008144 4.3150043006 -1.9240180873
C 5.3357903075 3.8910839669 -2.9706470394
C 4.8935251929 0.9049357811 -5.9593220947
C 4.9185075457 4.6668451093 1.4540381326
C 4.4542525218 5.7470802244 -1.650432193
C 1.8642487478 3.4007327438 1.0377049303
C 6.9146661213 0.1071447862 -3.3651935826
H -0.9900110459 0.2004392922 -0.5939023028
H -2.2269991852 0.1089790896 1.57079761
H 1.1635951704 0.1951966739 -1.8286118633
H -2.204950519 -0.0682908578 4.0393159212
H 2.0972391135 -0.2953236185 4.0138669799
H 3.3273596002 -0.1877353408 1.8585356755
H 6.2619338647 2.4307602753 1.9547431872
H 5.9878573028 1.642480183 0.3893368156
H 6.9801940899 3.1244131001 0.4801575559

H 7.9193089853 -0.0901374817 -3.7611104659
H 6.9769113803 0.1751015388 -2.2684673134
H 6.5702783629 1.0677471281 -3.7477084536
H 6.4847546695 -2.298560328 -2.1861529398
H 7.4702616459 -2.4786101353 -3.6634523601
H 5.7911290434 -3.0655751711 -3.6356696771
H 5.7606191391 5.3030652164 1.1358636809
H 5.0393001171 4.4597497125 2.5264702079
H 3.9901230155 5.2232694196 1.3168803939
H 2.1679076466 -2.3225251661 -3.2311274402
H 2.6312018293 3.1462407069 1.7693800966
H 1.6262945887 -2.1320571 -4.9222306843
H 2.419409967 -3.6423378427 -4.4047492008
H 1.2421252864 4.2096824583 1.4436400423
H 3.5454478546 -1.8376421907 -6.715157617
H 5.2098984552 -1.9953628524 -6.0905372303
H 4.1341602991 -3.4029215543 -6.1100818749
H 2.687048506 1.5480733814 -3.4945866883
H 0.9345682263 3.1073646141 -1.4944553731
H 2.2356401524 1.2289546867 -5.2020627275
H 0.7296945657 4.7831957905 -0.9218244409
H 3.2767850194 2.6126096979 -4.7789541704
H 1.9313171129 4.4156812936 -2.1818264164
H 4.230082179 0.6093042118 -6.7885114564
H 5.1709967535 1.9573581598 -6.1105865291
H 5.8022416079 0.3023494715 -6.0069242622
H 5.1964921761 2.8289790832 -3.1711673991
H 4.2578590391 6.2992436558 -2.5796928662
H 5.126002326 4.4594009932 -3.8876733423
H 6.3888009008 4.0721825684 -2.6930820592
H 5.4355917581 6.0731623846 -1.2666597786
H 3.6853584919 6.0200928396 -0.9249805059
H 1.2314878549 2.5183881094 0.8838991735

IaH^+
C 0.0142412556 -0.3011632 0.2460441945
C 0.1660034488 -0.2165947129 1.6579059059
C 1.4787739768 -0.0177512397 2.2030839301
C 2.5768640919 0.0983217375 1.3131776421
C 2.3985423381 0.0162288206 -0.0567728943
C 1.0996720745 -0.1924360677 -0.5954348044
C 1.6373213232 0.0706717698 3.61401102
C 0.5462044386 -0.031255625 4.44908204
C -0.7520021904 -0.2284125591 3.9106467511
C -0.9375920762 -0.3198941267 2.5477399343
H 0.6754779395 0.0357981909 5.525643304
P 3.8404202007 0.2368817829 -1.1305706108
N 4.5351839042 1.6733653214 -0.9311427342
P 4.0179712558 3.1503135074 -0.6435481497
N 2.3884080942 3.5041561165 -0.53117589
C 1.5426820927 3.5116850128 -1.7309016152

N 3.3799267405 -0.2062040092 -2.6095931428
P 4.2659499438 -0.491454201 -3.9043897276
N 5.8015807489 -1.1442535267 -3.7271719535
C 5.953930362 -2.5733035066 -3.4206677507
N 3.4307758151 -1.6185659846 -4.8064979761
C 2.0592005759 -2.0374242379 -4.5066654037
N 4.5793896225 0.9533865569 -4.7133416521
C 3.4988786572 1.9454689991 -4.7741951364
C 3.8906230063 -1.9650725026 -6.1537900234
N 4.6209325106 3.6135069726 0.850619958
C 5.741978148 2.9158483248 1.4861709224
N 4.5076272068 4.109770305 -1.9405920175
C 5.7293387737 3.7569037422 -2.6724119568
C 5.4620491951 1.0128401528 -5.8853162284
C 4.4074269626 4.9681240371 1.3699111333
C 4.2470428714 5.5544469824 -1.9224534548
C 1.6176118802 3.3108085338 0.7051383655
C 6.9042189968 -0.3301909609 -3.1954037645
H -0.9801654949 -0.457538039 -0.1656295498
H -1.932353111 -0.4738489822 2.1365757645
H 0.977751617 -0.2644449525 -1.6715445272
H -1.6027413805 -0.3097328065 4.5813968628
H 2.6341222841 0.2171504366 4.0239861847
H 3.5674119797 0.2692911091 1.7328063385
H 5.5535152232 2.8392920104 2.564487752
H 5.8443639013 1.9140806863 1.069811182
H 6.6864438013 3.4594894548 1.3385282916
H 7.8428772675 -0.6468347303 -3.6650703675
H 7.0032855549 -0.4512387528 -2.1073848891
H 6.7441412108 0.7255506378 -3.4117831159
H 5.9730066163 -2.7531302086 -2.3358057031
H 6.8990453305 -2.9284345677 -3.8468756013
H 5.1358277517 -3.1474015932 -3.8568452003
H 5.2715024927 5.6180163393 1.1716810777
H 4.2567381987 4.9186474697 2.4552002106
H 3.5182460065 5.4194297514 0.9271955837
H 1.8152065713 -1.7988809652 -3.4723372979
H 2.2808983501 3.1827428698 1.5595412722
H 1.3379550024 -1.5408090086 -5.171434487
H 1.9745905091 -3.1217076414 -4.6505902281
H 0.9842976722 4.1905161889 0.874982455
H 3.3438990239 -1.4048826046 -6.9256994922
H 4.9587584928 -1.7658268544 -6.2612299567
H 3.7298465273 -3.0358053396 -6.3270045168
H 2.8892255285 1.8907541025 -3.8737899371
H 1.058662887 2.5359106757 -1.8783027906
H 2.8562625938 1.7914464658 -5.6544771124
H 0.7609483936 4.2712145132 -1.6121878572
H 3.9375283956 2.9469984577 -4.8360121846
H 2.133053227 3.756092746 -2.6140767153
H 4.907581141 0.8593279042 -6.8224656644

H 5.9309712701 2.0033389704 -5.9238388805
H 6.2504960281 0.2625588164 -5.8162131529
H 5.7837910494 2.6771971043 -2.8007828224
H 4.1799208508 5.9151248185 -2.9553330941
H 5.6902651215 4.2272626352 -3.6616331495
H 6.6377209053 4.1119747357 -2.1618803902
H 5.0471496652 6.1132690515 -1.4152061398
H 3.2981102741 5.7676712639 -1.4273796205
H 0.9770887718 2.4255477945 0.6279513925
H 4.8040767354 -0.6692329052 -0.6418107277

Ib
C 0.0006026913 -0.0129555194 -0.030501095
N 0.0254447793 -0.0303103703 1.4381124517
C 1.3983027414 0.0054358223 1.9664449075
C 2.2732375329 -0.1597552782 0.7107062227
C 1.4140269488 0.4515257368 -0.4080240217
P -1.3940481763 0.1195158437 2.3369470118
N -0.8255787635 0.1598514203 3.9138474436
C -0.8073032751 1.3181267865 4.825469966
C -0.660133461 0.6897787116 6.2211753928
C 0.0684746505 -0.6351995875 5.9464940147
C -0.5586966976 -1.1037972454 4.6278450964
N -2.3892088258 -1.0153926094 1.946428098
P -4.020429032 -1.3905317357 2.3395866034
N -4.0393953728 -3.0704109504 2.5081703167
P -3.6204208972 -4.2212619047 1.5404514419
N -1.9898384097 -4.5607473568 1.2430652153
C -1.1820636654 -3.6948822893 0.3436522021
C 0.1456676311 -3.4901978434 1.0881269681
C 0.2592020692 -4.7441106485 1.9666321847
C -1.1879177545 -4.9565656542 2.4237251414
C -4.119639875 -0.9650989141 4.1716224799
C -4.2102329777 0.361611105 4.5521214361
C -4.2666374966 0.7527495626 5.9153767328
C -4.2610259349 -0.2612292105 6.9301160353
C -4.2047701643 -1.6220496758 6.5225181194
C -4.1319443881 -1.9612964199 5.1883415568
C -4.3012452678 2.1190404356 6.3119762693
C -4.3223876979 2.4709162398 7.6449579263
C -4.318737376 1.4669184893 8.6484754595
C -4.2933730936 0.1335182965 8.2958510077
H -4.3433554863 3.5192354389 7.9328927675
N -4.2057849901 -4.0781311166 -0.0294532302
C -5.6115032062 -3.6342359249 -0.1944926848
C -5.926956745 -3.9548333974 -1.6628085009
C -5.0723805175 -5.2028447954 -1.9342394226
C -3.7683911653 -4.8755261844 -1.1949561832
N -4.1765406201 -5.6375457262 2.2483792869
C -4.7498964231 -5.7260924072 3.6018769866
C -5.3376939925 -7.1434324509 3.6360185514

C	-4.3592630196	-7.9420785801	2.7592339274
C	-4.0284030149	-6.9588928669	1.6228103608
N	-1.9892113503	1.683228691	2.0475850764
C	-1.093631138	2.8538724171	1.957844537
C	-1.6077343926	3.6371534415	0.7421602812
C	-3.1167456309	3.3562290358	0.7889454656
C	-3.1849771354	1.8752215055	1.1891148341
H	-4.2092789753	-2.3979600869	7.2871217533
H	-4.2901528763	-0.6380784614	9.0635438232
H	-4.0762575805	-3.0008881784	4.8784246628
H	-4.3367652985	1.7539444373	9.6969941802
H	-4.3035004966	2.8858275625	5.539350172
H	-4.2113252262	1.1486947026	3.8003871479
H	-0.7793997925	0.6624879276	-0.400941199
H	-3.628504545	3.5505933847	-0.159381979
H	-0.2102269813	-1.0132633565	-0.4351247569
H	2.4383186978	-1.2256674521	0.5104353304
H	1.713141377	0.1285607755	-1.4109349908
H	1.4709634482	1.5475659042	-0.3726736421
H	3.2524096214	0.318083449	0.820909797
H	1.576658404	-0.7962889011	2.6926226766
H	1.6104657755	0.9572053188	2.4764750904
H	-0.0499156859	2.5413603995	1.8458878531
H	-1.1752947238	3.225659447	-0.1794634956
H	-1.3579331817	4.7027816732	0.7879065002
H	-3.5882278964	3.9797435956	1.5599997078
H	-1.7256462757	1.9061758921	4.7355739107
H	-4.106718659	1.6292448181	1.7248478525
H	0.044145299	1.9780630366	4.5969270731
H	-0.122042461	1.3452273658	6.914114888
H	-1.6497194291	0.4914776336	6.643301115
H	-0.0576352962	-1.3695907309	6.7489996006
H	1.1438863402	-0.4587585466	5.8100381312
H	-1.489644708	-1.6575380656	4.8007994874
H	0.1113595631	-1.7538299954	4.0515269743
H	0.0683916378	-2.5940496039	1.7098448757
H	0.9923016058	-3.3621996957	0.4041304544
H	-1.0237783573	-4.2130751749	-0.6123768466
H	0.5979869847	-5.6027738775	1.371840249
H	-1.6875315046	-2.7444071582	0.1541193535
H	0.9467304053	-4.6238813806	2.8113455975
H	-1.414201057	-4.3151559703	3.289425432
H	-1.4023232494	-5.9945155422	2.7036598991
H	-5.7119239151	-2.5722002157	0.0485811788
H	-6.2788197974	-4.1970494065	0.4765259729
H	-3.0150908742	-7.1027506364	1.2256664874
H	-4.7322537651	-7.0757767501	0.7833556192
H	-4.7712437073	-8.8872111338	2.3897567616
H	-3.4497958292	-8.1710363848	3.3299796479
H	-6.3374694867	-7.1447582363	3.1823222475
H	-5.4267184002	-7.536450277	4.6544442445

H -5.4921201617 -4.9403624129 3.7605920101
H -3.9695457061 -5.607065653 4.370818106
H -6.9973147283 -4.1113759536 -1.8329642349
H -5.5459453006 -6.0875756138 -1.4876583352
H -4.9142108432 -5.4030288931 -2.999744446
H -5.5991352622 -3.1295579345 -2.308353105
H -3.2076775074 -5.7632160883 -0.8886007511
H -3.1075921314 -4.2720496515 -1.8346879065
H -3.1491231763 1.220832642 0.3082239358
H -1.1631220387 3.4644447658 2.8703833879

IbH^+
C 0.0258717759 -0.0371165249 -0.0305185873
N 0.0387774727 -0.0784239821 1.4465745562
C 1.4155537154 0.0146578885 1.9834874478
C 2.2960983034 -0.1366272829 0.7325241993
C 1.4322104204 0.460304967 -0.3894178386
P -1.3587011491 0.1473052131 2.3253497863
N -0.879815082 0.1502076937 3.9111776763
C -0.7900386605 1.3300860483 4.7998444191
C -0.6156610005 0.7124757472 6.194839312
C 0.1224186946 -0.6057075073 5.9132361723
C -0.5376467637 -1.1027159828 4.6207051528
N -2.4043997889 -0.9850818829 1.9317715042
P -3.8120702607 -1.5028777156 2.5369748662
N -4.068640874 -3.0785430966 2.4709938874
P -3.5990096738 -4.2496124355 1.4986599887
N -1.9710411108 -4.5196173738 1.2674873997
C -1.1385291993 -3.7086011707 0.3384703706
C 0.2001593735 -3.5493315125 1.0729541689
C 0.2776794741 -4.8089152928 1.9480512721
C -1.167904793 -4.9731296531 2.4299738686
C -4.0800085058 -1.0166906317 4.2703416259
C -4.1414928218 0.3222253697 4.6104164671
C -4.2632924916 0.7335747821 5.9619272435
C -4.3241045739 -0.2674879095 6.9872040282
C -4.2820980162 -1.6377122105 6.6085910719
C -4.16318824 -2.0080016836 5.2874969133
C -4.296002082 2.1069016507 6.330917918
C -4.3782740403 2.4756942959 7.6558273771
C -4.4380336676 1.4856547932 8.6711567044
C -4.4153941306 0.1465253473 8.3441843655
H -4.4013991524 3.5268588771 7.9295644889
N -4.1642525088 -4.0254950352 -0.052943772
C -5.5724310032 -3.5707428519 -0.2216089433
C -5.9082646979 -3.934173531 -1.6759321666
C -5.0565500648 -5.1869281236 -1.9290114331
C -3.741659288 -4.8410536227 -1.2221022971
N -4.1782487425 -5.6274548431 2.2181990508
C -4.8011514166 -5.7093984336 3.5591144556
C -5.3721370169 -7.1332041017 3.588476224

C	-4.3697304869	-7.9305297382	2.7393614127
C	-4.0341551156	-6.9602973088	1.5960856765
N	-1.9769562928	1.664919051	1.9790820645
C	-1.0946886105	2.8483494563	1.83835569
C	-1.6905190314	3.618318983	0.6537632209
C	-3.1924177364	3.3327134673	0.7954650326
C	-3.2363703142	1.8546597552	1.2120719226
H	-4.3444981065	-2.3978641219	7.3840534474
H	-4.4650248825	-0.6109452902	9.1228120744
H	-4.1351811051	-3.0547339501	5.0027546497
H	-4.5054820964	1.7898248229	9.7119867962
H	-4.2533314268	2.8625321033	5.5492699053
H	-4.0704065809	1.0952298342	3.8494512595
H	-0.765837209	0.6269694031	-0.3958620695
H	-3.7599451884	3.515723435	-0.1217342374
H	-0.1598121533	-1.0377877715	-0.4413419682
H	2.4835658633	-1.1989575057	0.5349288457
H	1.7453837016	0.1478550178	-1.3901018184
H	1.4653164024	1.5565732036	-0.3516410839
H	3.2638922552	0.3600794654	0.8481396748
H	1.6107729955	-0.7711159648	2.7203470245
H	1.5879469844	0.9812862998	2.4762474237
H	-0.0578547194	2.5428528758	1.6662871265
H	-1.3124136156	3.20671844	-0.2907002776
H	-1.4466184693	4.6845248472	0.6802023892
H	-3.6201543236	3.9611069442	1.5864986545
H	-1.6917769801	1.9440933383	4.7242084569
H	-4.1169031349	1.6299260031	1.8227916659
H	0.0751075151	1.953156197	4.5318490631
H	-0.0706414115	1.376500167	6.8721289892
H	-1.5971797228	0.5091433494	6.6352747024
H	0.0335120341	-1.3316330793	6.7270039524
H	1.1893552037	-0.4157442879	5.7415106765
H	-1.4462745237	-1.6790596782	4.8351050151
H	0.1273124015	-1.7350754931	4.0214841744
H	0.1664615071	-2.6502103355	1.6959488022
H	1.0428384892	-3.4527935222	0.3806433089
H	-1.0095979273	-4.2559625792	-0.6035413381
H	0.5741591807	-5.6765167973	1.3458990656
H	-1.6130761587	-2.7479426464	0.1257100525
H	0.9824251338	-4.7190659271	2.7807811028
H	-1.3560728545	-4.3391447462	3.309899747
H	-1.4201162759	-6.0050794273	2.693834153
H	-5.6582885315	-2.4980400331	-0.0219940949
H	-6.2367641882	-4.0984176697	0.4770071849
H	-3.0215432574	-7.1063608451	1.2015107045
H	-4.7407497168	-7.0732505377	0.7611883011
H	-4.7667362526	-8.8821683171	2.3739257871
H	-3.4668243179	-8.144273222	3.3251701094
H	-6.3629313472	-7.1487850869	3.1174136425
H	-5.4764242648	-7.5171492852	4.6077928215

H -5.5609149968 -4.9346829401 3.6830015263
H -4.0437301769 -5.5722209435 4.3449895809
H -6.9801340676 -4.0979670262 -1.8210605304
H -5.5201213215 -6.0628173212 -1.4569731352
H -4.9148788242 -5.4092067841 -2.9911408728
H -5.5963918253 -3.1262911967 -2.3495118052
H -3.1695856648 -5.7191692204 -0.9114527279
H -3.0986497401 -4.2353796574 -1.8742357121
H -3.2638103354 1.1922092009 0.3377847697
H -1.121039513 3.4526748476 2.7549997761
H -4.8395263079 -0.8347499693 1.830780925

IIa
C 0.5376597285 -0.3801789728 -0.0425440729
C 0.3321287353 -0.1217381779 1.3115202872
C 0.6878460364 1.1249986999 1.8687801083
C 1.2574882652 2.1068426839 1.0645393006
C 1.4833441407 1.8334353098 -0.2931088548
C 1.1362882155 0.5941771804 -0.855669852
C 0.3648181754 1.1429155437 3.3501887353
C -0.1929185695 -0.2478768048 3.5877667436
C -0.2082722594 -0.9739657139 2.3773295596
C -0.6467466396 -0.8323203307 4.7657035709
C -1.1178698955 -2.1529433511 4.7335972009
C -1.1318679054 -2.874248305 3.5318921761
C -0.6771872152 -2.2899791029 2.3450365483
H -1.5008699604 -3.897020575 3.5226764504
P 1.4075628955 0.1529989719 -2.6514058955
N 2.7204443839 -0.9138860808 -2.5603361594
P 3.7024814215 -1.6877504186 -1.6466751498
N 4.4653221824 -1.033549065 -0.2734599154
C 5.4112624014 0.0656790704 -0.4806947915
N 2.0661882182 1.6356208992 -3.1816058953
P 2.4578935826 1.9526194588 -4.6646264021
N 1.6307921678 1.2696774416 -5.9860479559
C 0.2312234353 1.6715699839 -6.1726437915
N 2.2083245659 3.6098658009 -4.9254138132
C 2.1197023828 4.5254119951 -3.7907904819
N 4.0513405725 1.4710936881 -5.01392813
C 5.0202656559 1.4409866675 -3.9203545387
C 2.665019831 4.2396470463 -6.1587586703
N 2.9720737374 -3.0654506028 -0.9688140822
C 1.7793200986 -3.6366388637 -1.5840648761
N 5.0686822218 -2.0744637178 -2.5932387884
C 4.9161104676 -2.1679934099 -4.0430983195
C 4.667955531 1.6273469322 -6.3272124248
C 3.6577686341 -3.9901953462 -0.0727358784
C 6.1029806751 -2.9563764284 -2.0626347201
C 3.7323733337 -0.8833024692 0.9890587947
C 1.830923358 -0.1546154669 -6.2862878873
H 0.25640831 -1.3434762576 -0.465351329

H -0.6904628553 -2.8500391279 1.4128404192
H -1.4750835211 -2.6208439089 5.6475722111
H 1.9440920219 2.5794011815 -0.9346528397
H 1.536597913 3.0736442714 1.4808165424
H -0.63771259 -0.2765959942 5.7012368631
H -0.3672736664 1.9232373558 3.6021759965
H 1.2551132691 1.3375662319 3.9647062465
H 1.0613860189 -3.9237633116 -0.8017360706
H 1.3090982542 -2.906119844 -2.243494628
H 2.019640373 -4.5377881616 -2.1720372944
H 1.6040012577 -0.328260486 -7.3467538012
H 1.1780009368 -0.7940445758 -5.6749548929
H 2.8650160593 -0.4439456399 -6.0977638761
H -0.4450234781 1.1088432052 -5.5112896762
H -0.056325753 1.4785596819 -7.2142698475
H 0.1149194664 2.7374837376 -5.9687213803
H 4.0051164345 -4.891794212 -0.6027485119
H 2.9705012345 -4.3101676351 0.7237334496
H 4.5169869486 -3.5090900264 0.3960741643
H 1.7129341622 3.9978136108 -2.928354493
H 2.9625877208 -1.6475390283 1.086382477
H 3.1029883714 4.9469890157 -3.5217627251
H 1.4507648468 5.3567966732 -4.0518206142
H 4.4393621881 -0.984992307 1.8239511968
H 3.7077374482 4.5926954702 -6.0917552732
H 2.5818306743 3.5430704223 -6.995769384
H 2.0291138556 5.1077496819 -6.3773414804
H 4.5013443596 1.262350182 -2.981010982
H 4.8972657477 1.0379091077 -0.5410283166
H 5.5890107765 2.3840953438 -3.8516140438
H 6.1103480109 0.0985867249 0.3653000018
H 5.7300328678 0.6205514882 -4.0850055712
H 5.9796396393 -0.0905004896 -1.3988368799
H 5.2370574103 2.5675860173 -6.4102830944
H 5.3659371883 0.7961000463 -6.5005634952
H 3.9116215647 1.605612112 -7.1135833214
H 4.1906931286 -1.4278944192 -4.3797577637
H 7.04908533 -2.7560935718 -2.5828645832
H 5.8859834231 -1.9644656468 -4.517571775
H 4.5828940705 -3.1696879328 -4.3654922523
H 5.8618278232 -4.0241459379 -2.200775041
H 6.2556395748 -2.7657180294 -0.998249323
H 3.2459995423 0.0974981551 1.0579812911

IIaH^+
C 0.6604917504 -0.3109711107 0.0437796053
C 0.4256118089 -0.105226185 1.400490434
C 0.7740250812 1.122355856 2.0089284767
C 1.349396582 2.1434697273 1.2598756205
C 1.59765865 1.9358827303 -0.1038722689
C 1.2643905398 0.7119397737 -0.7045410087

C	0.4286468015	1.0798604487	3.48276594
C	-0.1305087784	-0.3187196253	3.6564547847
C	-0.1311885076	-0.9985690451	2.4208329444
C	-0.6010216576	-0.9487971796	4.8046118501
C	-1.0721318164	-2.2648565177	4.7098222785
C	-1.0731079222	-2.9399947229	3.4805165454
C	-0.6021012735	-2.3108834549	2.3250855312
H	-1.447175512	-3.9588164356	3.4273106508
P	1.6912914815	0.3568161969	-2.4250110517
N	2.6929593143	-0.8889286332	-2.5812941092
P	3.5961048797	-1.8802338447	-1.7365876294
N	4.5845346417	-1.2881091771	-0.5117000683
C	5.8432957703	-0.6301486659	-0.8749986202
N	2.1508880247	1.7427145032	-3.0971741588
P	2.6082764235	2.0873397384	-4.5841623287
N	1.7282893709	1.4358519623	-5.8625831195
C	0.4731294957	2.082562479	-6.2639342542
N	2.5363221814	3.7485979586	-4.6948400095
C	1.6294976616	4.5666460705	-3.8838639437
N	4.1573270414	1.5264900231	-4.8897001823
C	5.1689708133	1.7169279022	-3.8453537936
C	3.0838664767	4.4377058985	-5.8642122388
N	2.6624053186	-3.0099775796	-0.928248616
C	1.4820548521	-3.5592845382	-1.6009969646
N	4.599984987	-2.61102819	-2.8571132316
C	4.972939948	-1.9905630667	-4.1269816365
C	4.7316598856	1.3435767162	-6.2257157555
C	3.1278364299	-3.8458815423	0.1841972634
C	5.4430627217	-3.7411397951	-2.4689177557
C	4.0144472748	-0.7527860881	0.7341739507
C	1.7047587645	-0.0211458962	-6.058614021
H	0.4070638645	-1.2615046912	-0.418074883
H	-0.6107941586	-2.8325809748	1.3705911106
H	-1.4433562764	-2.7680223302	5.5983459747
H	2.0549910999	2.7157190575	-0.7052494606
H	1.6114155429	3.0922974584	1.7218518068
H	-0.6061055149	-0.4325084982	5.7615109955
H	-0.3063890697	1.8523716324	3.747584617
H	1.3099316279	1.2571712917	4.1140844664
H	0.6821365141	-3.7081430091	-0.8648164693
H	1.1261057783	-2.8738839	-2.3719155785
H	1.7041169337	-4.5290937681	-2.0687941679
H	1.6282224696	-0.2403973853	-7.1301368887
H	0.8459609056	-0.4815774546	-5.5491186212
H	2.6164382075	-0.4777530604	-5.6722819843
H	-0.378850054	1.7385573497	-5.6585200202
H	0.271819429	1.8407222539	-7.3134070518
H	0.5496813787	3.1668103294	-6.1741219721
H	3.2616157079	-4.8883477548	-0.1381655636
H	2.3822618292	-3.8285919761	0.9893133188
H	4.0745565219	-3.4769784481	0.5785080043

H 1.2853778214 3.9966251916 -3.0212293381
H 3.074024219 -1.2454571337 0.9764917784
H 2.1663467387 5.4555156431 -3.5314131753
H 0.7588340593 4.8974758354 -4.4693993649
H 4.7239492403 -0.9299015015 1.5509537941
H 3.602050255 5.347857332 -5.5400771857
H 3.8017356592 3.8076830008 -6.3905522599
H 2.2934767429 4.7250772783 -6.5732142584
H 4.6926358549 1.7722711224 -2.8662361989
H 5.7053926215 0.4482009007 -1.0424857848
H 5.7498928142 2.6353067745 -4.0139196731
H 6.5615776687 -0.7619380512 -0.0579174789
H 5.8567986496 0.8641030147 -3.8499295438
H 6.2686400538 -1.0718832184 -1.777182844
H 5.4393226794 2.1492798571 -6.4689282103
H 5.2783738959 0.3923449835 -6.2576952584
H 3.9483586048 1.3219146129 -6.9836087339
H 4.3452087804 -1.1194341469 -4.3124502297
H 6.4787982067 -3.4273701626 -2.2693077054
H 6.028468074 -1.676527183 -4.1172122043
H 4.8401684442 -2.7113047525 -4.9440048661
H 5.4571697876 -4.4794859733 -3.2794724163
H 5.0556084177 -4.2297283305 -1.5743360036
H 3.8236884054 0.3257193844 0.6586437703
H 0.4772118897 -0.0747609927 -3.0052333687

IIIa

C -0.1059168567 0.0531229285 0.0294083799
C -0.009371155 0.0478719358 1.4296501621
C 1.2724296081 0.0694600882 2.0282039514
C 2.4376905104 0.0910634302 1.255569385
C 2.3176675725 0.0940883693 -0.134263219
C 1.0515624972 0.0758398522 -0.7449384667
C -1.0767395921 0.0354023898 2.4168770658
C -0.5800422893 0.0566236302 3.740405902
S 1.1890971586 0.0715426925 3.794875401
C -1.4481666563 0.0812692201 4.8360138882
C -2.8236526211 0.105384634 4.5996248539
C -3.3449838028 0.0927083511 3.2912444038
C -2.4618131968 0.0348466463 2.2111469995
P -5.1714114337 0.1554563605 2.8961547061
N -5.7728407133 0.1196653636 4.4914240779
P -7.2919106055 0.1290066178 4.8742569453
N -7.9212049784 1.7071309877 4.9405055136
C -9.3201714751 2.0055387294 5.2295968195
N -5.364771948 1.7598774992 2.3872182159
P -4.6772796601 3.085580872 1.9796442776
N -5.8780138122 4.2966532428 2.00457014
C -5.580300108 5.631749096 1.4965249183
N -3.4574174138 3.8523625036 2.8853291522
C -2.0786439284 3.3495087343 2.8543638285

N	-3.9065645508	2.9624082391	0.4678871013
C	-3.1733692782	4.0566959238	-0.1610019415
C	-3.8145323281	4.3582542331	4.2131384241
N	-8.4705904533	-0.6342334123	3.9124439857
C	-8.8276851426	-0.0289365753	2.6220451238
N	-7.4592021107	-0.6996546379	6.3438553841
C	-8.7316830131	-0.6783422842	7.0560406051
C	-8.4130046146	-2.0984907256	3.8264265487
C	-6.2997374495	-0.9048607714	7.2083760808
C	-7.0144526068	2.7997754248	5.2873402228
C	-4.3324808144	1.93857306	-0.4800590041
C	-7.2760545006	3.909007662	1.8341300832
H	0.9762127846	0.0779447301	-1.82914228
H	-2.8514848485	0.0160158434	1.1948937704
H	-1.0841131161	0.0369161334	-0.4447435095
H	3.2140363141	0.1106833413	-0.7487280414
H	-3.5205195254	0.1455469154	5.4321129011
H	-1.0608184721	0.0947351719	5.8520016238
H	3.4167495897	0.1060951695	1.7268211997
H	-3.4511449724	1.5078795357	-0.9776261086
H	-4.8684262709	1.1420317844	0.0377900164
H	-4.9912740234	2.3558111726	-1.259542213
H	-9.8397970834	-0.3537865681	2.3459374955
H	-8.1303521175	-0.3285701688	1.8264334783
H	-8.8164107847	1.0588657214	2.6930624933
H	-7.6577230901	-2.4346381929	3.0996489267
H	-9.3947389912	-2.4727303595	3.5087798407
H	-8.1745866674	-2.5243746656	4.8026093193
H	-3.7838450363	4.5771605427	-0.9164995264
H	-2.2793406861	3.6600374084	-0.6631154066
H	-2.8481137861	4.7832004699	0.5844964991
H	-5.3984778549	-0.9705281336	6.5992492695
H	-1.8499612471	2.8967647048	1.8906770736
H	-6.1791331847	-0.0878230418	7.9396233994
H	-6.4277639592	-1.8443891153	7.7629259889
H	-1.3906583979	4.1890381292	3.0222458951
H	-8.8220975819	0.187624963	7.7329817918
H	-9.5639378525	-0.6612051803	6.3490131895
H	-8.8215273742	-1.5899951336	7.6612645981
H	-6.0004221986	2.538685703	4.9921160411
H	-3.7497038936	3.570032046	4.9797847751
H	-7.0373823597	3.021880333	6.367897338
H	-3.1180202721	5.1611572255	4.4878728166
H	-7.3094971932	3.7049552668	4.741812649
H	-4.8287779162	4.7603075159	4.2066742137
H	-9.4885053142	2.2096931816	6.2994255026
H	-9.6238838379	2.8987481916	4.6655072846
H	-9.9620857413	1.1768889789	4.9263236175
H	-7.4425890163	2.9393688784	2.3025461889
H	-6.2623884797	6.355306393	1.9622817367
H	-7.9158462301	4.6590835493	2.319196276

H -7.5674680949 3.8487974996 0.7712745398
H -5.7049354363 5.7060854254 0.4028594884
H -4.5580203708 5.9171174799 1.7536005931
H -1.9067569734 2.595773998 3.6326855187

IIIaH+
C -0.1072753798 -0.0035606944 0.0380618987
C -0.0182484335 0.0144509464 1.4391273162
C 1.2572164535 0.0321785609 2.047851283
C 2.4297103921 0.0324324779 1.2859431719
C 2.3172143241 0.0157256238 -0.1033907068
C 1.0559427017 -0.0025815384 -0.725463388
C -1.0871205375 0.0259269776 2.4224790059
C -0.5944337106 0.055673189 3.7520982519
S 1.1632354849 0.0563593454 3.8150380682
C -1.462793839 0.0904618188 4.8495768662
C -2.8361878857 0.1197925608 4.619895809
C -3.3401040741 0.1117230818 3.3040963076
C -2.4686860001 0.0484916486 2.2129744384
P -5.106274481 0.2936848558 2.9668642765
N -5.869408946 0.1471997306 4.3738727623
P -7.4231787168 0.1466042802 4.730196761
N -8.0562035946 1.6969686565 4.682384166
C -9.4840177755 2.014655677 4.5875927292
N -5.4449555428 1.5906990155 2.081956173
P -4.7866058995 2.9304357993 1.5460100244
N -6.0769756218 3.9071158222 1.1242770931
C -5.8533538455 5.1662660743 0.4146830186
N -3.791088833 3.850202686 2.5394563628
C -2.4423830426 3.3990396463 2.9153225512
N -3.793169437 2.6196322432 0.2333750658
C -2.8146201277 3.5644012922 -0.3180252866
C -4.4053940243 4.6898713218 3.5725191637
N -8.4723443987 -0.7058508101 3.728037171
C -8.6526462257 -0.2794874443 2.3324936281
N -7.5253203564 -0.497931055 6.2632221805
C -8.7878033955 -0.4559320015 7.0026672669
C -8.5997619017 -2.1580797141 3.899622035
C -6.5441102842 -1.4448559747 6.8015340259
C -7.2609577931 2.7753490438 5.2785595631
C -4.2082052085 1.6012920112 -0.7358754385
C -7.4111239326 3.7768861073 1.7071523376
H 0.991686818 -0.0213200257 -1.8096334805
H -2.8554362066 0.0517874531 1.1974604443
H -1.0806135196 -0.0276776794 -0.4459145456
H 3.2173426081 0.0139251271 -0.7114337806
H -3.5295562395 0.1581724535 5.4543083765
H -1.0749257877 0.1017246646 5.864134971
H 3.4054186184 0.0442092382 1.762806802
H -3.3207563727 1.0730631369 -1.1062318932
H -4.8784977132 0.8780907959 -0.2687311582

H -4.7229763032 2.0530495992 -1.5959815328
H -9.6825978026 -0.4892714716 2.0205555682
H -7.9705534154 -0.8156471089 1.657079546
H -8.4666160128 0.7898479089 2.2279568428
H -7.8231000557 -2.7073249755 3.3466358189
H -9.5796971046 -2.472815255 3.524032875
H -8.5366993314 -2.4309823856 4.9537053902
H -3.1736685488 3.9956271374 -1.2634017213
H -1.8733845299 3.0365953125 -0.5171468439
H -2.6162276867 4.3743468372 0.3837953718
H -5.6267497656 -1.4040655781 6.2148473112
H -2.0130527968 2.7653484655 2.1411808013
H -6.3148041233 -1.1737332736 7.8390309687
H -6.9345796815 -2.473261412 6.7904918822
H -1.7999945691 4.2778378031 3.0452884387
H -8.5862306333 -0.213275372 8.052641171
H -9.4536976504 0.3078115772 6.5994639857
H -9.3112551706 -1.4226361811 6.9666412561
H -6.2013335535 2.5200673621 5.2516406308
H -4.5118553514 4.1484494377 4.5238911379
H -7.5583443187 2.9626867227 6.3205268766
H -3.7697315614 5.5658445204 3.7446267211
H -7.4121583501 3.6950762093 4.7027612001
H -5.3901282908 5.0383152606 3.2576867458
H -9.8832596832 2.3471431065 5.5566386756
H -9.6264089381 2.82908255 3.8656925005
H -10.0519157038 1.1468112868 4.2517884642
H -7.4758315816 2.855717442 2.2853590508
H -5.895679018 6.0289985554 1.0964433867
H -7.6355445044 4.6301413287 2.366121717
H -8.1644107947 3.756128953 0.9091434307
H -6.626679587 5.2948074454 -0.352047826
H -4.8823999072 5.171071045 -0.0810919684
H -2.4557113145 2.8297857354 3.8536352685
H -5.4252430053 -0.8030482746 2.1354502891

IIIb
C -0.1595322744 -0.0690517425 -0.1359130421
N -0.0798151485 -0.0159259955 1.3429116274
C 1.3339502781 0.0002549071 1.7840111649
C 2.1488454844 -0.1493612726 0.4873047366
C 1.2134213412 0.4462707411 -0.5759778883
P -1.3339737655 -0.6870662723 2.2585265318
N -2.6582243678 -0.8447912752 1.4382723548
P -3.8711282203 0.2247033749 0.8746556831
C -3.4401675873 1.9119974358 1.6007223307
C -2.3208124872 2.6313672677 1.1278556876
C -2.0049841064 3.9041164331 1.5998426566
C -2.8380996658 4.4878308981 2.5598601494
C -3.9790662788 3.8055103185 3.0345085469
C -4.2686878065 2.5259709032 2.5412819584

C	-4.6898994634	4.5659845275	4.0494343587
C	-4.0713718595	5.8099372124	4.317808949
S	-2.6234970676	6.065048426	3.3332734897
C	-4.5924169487	6.6936136924	5.2679666521
C	-5.7459719753	6.3251287951	5.9608129954
C	-6.3711009571	5.0913748085	5.7081840788
C	-5.8486700032	4.2152540366	4.7598512434
N	-0.6912713372	-2.1804465043	2.7394201388
C	-0.1899135855	-3.1449056094	1.7512858468
C	0.3826064294	-4.2608525121	2.632133749
C	-0.6312896204	-4.3279519782	3.787404636
C	-1.0830574328	-2.8638395832	3.9873114049
N	-1.4194716136	0.1984272354	3.6827193407
C	-0.5482064521	1.3283802376	4.0589148817
C	-1.3848982142	2.1244181935	5.0722471613
C	-2.2880673533	1.0593973949	5.7103181518
C	-2.6298879082	0.1358274862	4.5332637152
N	-5.2079083042	-0.0748922978	1.8870311513
P	-6.2609487741	-1.2266708051	1.7622272072
N	-7.4187852216	-0.9186113495	2.9360496203
C	-7.39365147	0.2381044259	3.8501410809
C	-8.770542653	0.1869624889	4.5357698497
C	-9.1347434378	-1.3062645033	4.5019560628
C	-8.6158150292	-1.7463880711	3.1259192289
N	-5.8314187773	-2.8318511042	2.0767873377
C	-4.9807672049	-3.5632088224	1.0986982138
C	-3.8633088902	-4.1803346796	1.9469361126
C	-4.5460905917	-4.4067110746	3.3026521633
C	-5.3867398731	-3.1341243086	3.4588579257
N	-6.9562029589	-1.3842504169	0.2404195765
C	-7.3200669859	-0.1467979944	-0.4911125827
C	-8.2288809732	-0.6440142471	-1.6255698966
C	-8.9163205485	-1.8713979273	-1.007285264
C	-7.7739801788	-2.5262576163	-0.2202061986
H	-7.269109968	4.820300267	6.2573605975
H	-6.3322710685	3.2614305148	4.5646448259
H	-5.1340104651	1.9780097028	2.8996866748
H	-1.6720988352	2.1870396308	0.3797754731
H	-6.1631746393	7.0008157511	6.7029932794
H	-4.109280648	7.6470265588	5.4644258
H	-1.1280834719	4.4286679542	1.2285171637
H	0.5683599632	-2.678188004	1.1147199582
H	1.4638516515	0.1421434183	-1.5977448573
H	-0.98837296	-3.5266805534	1.0951524646
H	-1.4874470942	-4.9435066468	3.4882345298
H	0.4872837673	-5.2111763677	2.0977117618
H	1.371669191	-3.9662589349	3.0050904917
H	-0.2144387873	-4.7617859327	4.7023160041
H	-2.1648512838	-2.8149365704	4.1671567279
H	-0.5861825192	-2.3853666546	4.840781907
H	1.5306660746	-0.8020802409	2.5051212076

H 2.3311651463 -1.2097613638 0.2701017108
H 3.1214098474 0.3507368219 0.546586195
H 1.2368674215 1.5430924482 -0.5302251862
H -0.2949338738 1.93343318 3.1835321015
H -0.9897382092 0.5328704792 -0.5120472813
H 0.3866753906 0.9640921325 4.5121541083
H -0.7601250468 2.6545027611 5.7990798328
H -1.9937864934 2.8633984181 4.5451470781
H -3.1857233021 1.4821443439 6.1739379608
H -1.7362888046 0.5030489353 6.4797739866
H -3.5132340693 0.481192313 3.9876929514
H -2.8318215414 -0.8885860025 4.8655939294
H -3.0627502765 -3.4428280681 2.0466525682
H -3.4519641364 -5.0943715498 1.5039489346
H -5.5816176773 -4.3375576464 0.6018274652
H -5.1962478775 -5.2908009835 3.2641799687
H -4.5839141431 -2.8914602366 0.3312679194
H -3.8435696625 -4.5412498903 4.1324512073
H -4.7697831251 -2.3175766027 3.8624021938
H -6.2499505346 -3.268271782 4.1206406602
H -6.4226733597 0.3645532068 -0.8513395709
H -7.8647641837 0.5490653799 0.1655933975
H -8.3664848039 -2.8132981741 3.0855652444
H -9.3680130105 -1.5503297406 2.3434459002
H -10.2052459009 -1.4977738568 4.6316181444
H -8.593961586 -1.8447044912 5.2911134873
H -9.5017988533 0.7610568771 3.952015129
H -8.7470889886 0.605113451 5.5476723259
H -7.2360222435 1.1720643361 3.302636934
H -6.5744585175 0.1394602454 4.5764753042
H -8.9296294309 0.1263713386 -1.9639207849
H -9.7108530095 -1.5519642956 -0.3193164886
H -9.3587841162 -2.5457194555 -1.7487383154
H -7.6217449024 -0.9502893014 -2.4872028018
H -8.1184539161 -3.1384479903 0.6179221728
H -7.1765912031 -3.1741215786 -0.8787143016
H -0.3229952407 -1.0951151522 -0.4976016091
H 1.566614205 0.953852615 2.2756002675

IIIbH^+
C 0.0304820296 -0.0483680486 0.0039834368
N 0.016409631 -0.0121443772 1.4906295614
C 1.4000591167 -0.0128735871 2.0255481793
C 2.2642077315 -0.4169695018 0.825199666
C 1.5023212025 0.2044605855 -0.3538998732
P -1.2625600454 -0.621535676 2.3816026416
N -2.5045675919 -0.9144267237 1.4284257205
P -3.5904472574 0.0278544829 0.6881377076
C -3.592498718 1.7515440812 1.2700840751
C -2.535942248 2.6287001924 0.9485533604
C -2.5151499914 3.9318181384 1.4363380818

C	-3.5591428126	4.3564123598	2.2667970836
C	-4.6321952252	3.4921911756	2.5950025591
C	-4.6416031987	2.1917941976	2.0789945049
C	-5.5964970055	4.1242944131	3.4781385488
C	-5.227069142	5.4500957192	3.8004011871
S	-3.7122230139	5.9367653757	3.0244741706
C	-6.0123638251	6.2427492821	4.6430969817
C	-7.1814152523	5.6943728686	5.1684598785
C	-7.5635874281	4.3768925063	4.8580766408
C	-6.7788056787	3.5932108337	4.0177826673
N	-0.8166321421	-2.0549320881	3.1078627134
C	-0.4548666722	-3.2313256727	2.2870828869
C	0.5741639448	-3.9721428979	3.1503744552
C	0.0884833044	-3.677350591	4.5777923504
C	-0.358373456	-2.2082802201	4.5080512676
N	-1.5288458524	0.4260051327	3.6385002074
C	-0.7605520079	1.6573010253	3.9368735074
C	-1.6889270318	2.4384867579	4.8777153119
C	-2.4748337477	1.3365292484	5.6050622164
C	-2.7265681655	0.3043697403	4.4986353348
N	-5.1094298503	-0.4586873627	0.75962463
P	-5.8788534855	-1.8387029272	0.936131801
N	-7.4586489728	-1.3907455403	1.1836962197
C	-7.9342760025	-0.0188398271	1.4651499137
C	-9.4566970132	-0.1489641959	1.3351408763
C	-9.7284085274	-1.5722781631	1.8474404124
C	-8.5521456191	-2.3811033695	1.2763417513
N	-5.4966515609	-2.841737253	2.2107411843
C	-4.3054209072	-3.7334885142	2.2112753301
C	-3.8211604158	-3.7157378171	3.6690800405
C	-5.0973089601	-3.4151621347	4.4689950512
C	-5.8120583205	-2.3869817465	3.5859852006
N	-5.6772621982	-2.8240951069	-0.3898360322
C	-5.7319471249	-2.1998594328	-1.7410358039
C	-5.9850132532	-3.3842281933	-2.6862972329
C	-6.8020501427	-4.3552846155	-1.820911821
C	-6.1108284133	-4.2440425205	-0.4577714942
H	-8.4801068672	3.9709306278	5.2767004038
H	-7.0783248487	2.5763828912	3.7754823395
H	-5.465153864	1.5193184647	2.2969861289
H	-1.7212389994	2.2965269383	0.312090385
H	-7.8046188407	6.2955076071	5.8244472639
H	-5.7213405696	7.2613001818	4.8829481439
H	-1.7007262653	4.6029303717	1.1787710479
H	-0.0508571363	-2.9235101316	1.3162514022
H	1.7676667331	-0.2293976031	-1.3225013543
H	-1.3396633962	-3.8527712401	2.0989696059
H	-0.7688246076	-4.3168240302	4.8205567568
H	0.6229270486	-5.040858777	2.9210278895
H	1.5735731095	-3.5464319998	2.9941076662
H	0.8552015868	-3.838060726	5.3413509164

H -1.1639287716 -1.9909393432 5.2166908939
H 0.4736098775 -1.5290248648 4.7370545991
H 1.4952538851 -0.707789948 2.8653024512
H 2.2865107326 -1.510121798 0.729435263
H 3.2957900463 -0.0630506083 0.9119959402
H 1.7002856441 1.2824558568 -0.4016704595
H -0.5335101036 2.2064925988 3.0193722667
H -0.6309394645 0.7127638487 -0.4204003246
H 0.1860756915 1.4050096913 4.4343999768
H -1.1319397712 3.0905371072 5.5569868108
H -2.3711581039 3.0615264186 4.2916216397
H -3.4062160245 1.6967059945 6.0522045244
H -1.8633695171 0.892786976 6.4006135027
H -3.6324345367 0.548723013 3.9292118067
H -2.8405419742 -0.7137494912 4.886528487
H -3.0906396513 -2.9114848357 3.7978050684
H -3.3441593573 -4.6581127731 3.9571815128
H -4.6082570753 -4.7425546156 1.9065590601
H -5.7123299101 -4.3181501688 4.5662233217
H -3.5433138007 -3.3700332093 1.5174269893
H -4.9003921414 -3.0303479881 5.4747348183
H -5.413525479 -1.3773414923 3.7708841983
H -6.8947324826 -2.3596438367 3.7444821936
H -4.7980138538 -1.673137084 -1.9602617371
H -6.5521564593 -1.4707740904 -1.7976092546
H -8.2720574998 -3.2250963617 1.9184893483
H -8.7913134784 -2.7806776169 0.2806758627
H -10.6976421675 -1.9714993444 1.5345740865
H -9.6959253955 -1.5888689183 2.9441971442
H -9.749762087 -0.0622964229 0.281516062
H -9.9896567114 0.6225953485 1.899142279
H -7.4949457832 0.6957462604 0.7661375127
H -7.6550048427 0.2869766199 2.4858951212
H -6.4994006611 -3.076232461 -3.601349915
H -7.8425639734 -4.013825277 -1.7459183625
H -6.808020002 -5.3801122014 -2.204664391
H -5.0336824673 -3.8484829297 -2.9743611229
H -6.7581891197 -4.5053713944 0.3832549949
H -5.2285739516 -4.8964241775 -0.4176465275
H -0.3093254092 -1.0229575948 -0.3681680387
H 1.6669148391 0.989988683 2.3843671636
H -3.196553696 0.1317278082 -0.6656837375

IVa
C 0.2982053171 -0.1689262037 0.0337482288
C 0.0692674561 -0.060900542 1.41048247
C 1.1722383228 0.0002735049 2.2858250163
C 2.4910640276 -0.0420684456 1.853928799
C 2.6980863919 -0.1501443106 0.4744642662
C 1.6170054871 -0.2125208402 -0.4238809179
C -1.1053990197 0.0186897561 2.2597424931

C	-0.6121222727	0.1235980084	3.5738386701
O	0.7700877236	0.1085620122	3.5995433571
C	-1.438175377	0.2338367045	4.684602895
C	-2.8163773319	0.25110942	4.4442637441
C	-3.3508668273	0.1557182281	3.142670726
C	-2.4879878808	0.0229707164	2.0489915719
P	-5.1864065199	0.2139294425	2.7812125632
N	-5.7560070218	0.0505910838	4.3769427423
P	-7.2497829519	0.086746101	4.83868987
N	-7.8281793442	1.6755187406	5.0229059871
C	-9.2015396447	1.9892948589	5.4054803232
N	-5.4249577173	1.8534529188	2.419037547
P	-4.7900866494	3.1732937366	1.9246655738
N	-5.9790049558	4.3852660059	2.0775457186
C	-5.7289706262	5.7377147013	1.5912493038
N	-3.4816707973	3.9332180262	2.7052258967
C	-2.1170560416	3.4219319517	2.526583067
N	-4.1773366271	3.0513972952	0.3414138237
C	-3.551530673	4.1673827035	-0.3616768791
C	-3.7063067592	4.3924122379	4.0791195002
N	-8.4995733202	-0.5915242584	3.90039411
C	-8.8873477961	0.0916469152	2.658552269
N	-7.3639967774	-0.8184742042	6.2676505397
C	-8.5985787521	-0.8122961275	7.0436519187
C	-8.4786236586	-2.049729106	3.73018861
C	-6.1670245296	-1.0930922406	7.0590502008
C	-6.874506519	2.7190206558	5.3974505924
C	-4.7221095927	2.0394961635	-0.5589460729
C	-7.3878939177	4.0033166385	2.0406007569
H	1.8134726414	-0.2975491146	-1.4893240286
H	-2.8904491141	-0.0629689037	1.0407530887
H	-0.5345253012	-0.2204293241	-0.6628861477
H	3.7145324704	-0.1871884697	0.091771761
H	-3.5110546952	0.3392332048	5.2752226539
H	-1.0264375682	0.3070810012	5.6870252527
H	3.3149450776	0.00634842	2.5589667569
H	-3.9169550058	1.6452044374	-1.1949705989
H	-5.1522930567	1.2146297148	0.010833201
H	-5.5024273674	2.4560882927	-1.2177743315
H	-9.9077226979	-0.213156827	2.3910425491
H	-8.2137809033	-0.1610102422	1.8264816489
H	-8.8701256453	1.1729662476	2.7949893757
H	-7.7600404697	-2.3606178909	2.9562423524
H	-9.480183839	-2.3848177071	3.4316243357
H	-8.2121158539	-2.5352495011	4.6705776516
H	-4.2660971344	4.6957593643	-1.0135317119
H	-2.7338182753	3.7912919	-0.9927444488
H	-3.1311337286	4.8827450093	0.3464675379
H	-5.2985132802	-1.1389593823	6.4020302377
H	-1.9928382221	2.9828806213	1.5373204924
H	-5.994512421	-0.3213977617	7.8282441283

H	-6.2862676418	-2.0604264985	7.5657134475
H	-1.408503501	4.2539224222	2.6366573646
H	-8.6347444259	0.0143011152	7.7729007396
H	-9.4642238915	-0.7365117045	6.3820161773
H	-8.6798772868	-1.7556224014	7.5996816326
H	-5.8862143519	2.4660507798	5.0187824876
H	-3.5795626053	3.575108384	4.8066787884
H	-6.827928304	2.8561477861	6.4913439147
H	-2.978299785	5.1791324561	4.3156652932
H	-7.1816245885	3.6694465109	4.9431243849
H	-4.7124168848	4.8020383024	4.1832835663
H	-9.3081479177	2.1289089086	6.4935427351
H	-9.5090691682	2.923934333	4.9154806051
H	-9.8812413932	1.1974244832	5.086516722
H	-7.5072430189	3.019593423	2.4938452717
H	-6.3463031621	6.4458964516	2.1602680605
H	-7.9727433637	4.736691788	2.6130244256
H	-7.7877435602	3.9777332014	1.0121534677
H	-5.9784905301	5.8565542891	0.5232658331
H	-4.6813189991	6.0073019224	1.7395038396
H	-1.8735893811	2.6549218644	3.2720403856

IVaH^{+}

C	0.2056701191	-0.1235770547	-0.050332798
C	0.0032105731	-0.078412811	1.3348272732
C	1.1182128645	-0.0881365219	2.1932617215
C	2.4298422777	-0.140240087	1.7437311109
C	2.610369043	-0.1836088627	0.358217937
C	1.5154847904	-0.1754489622	-0.5270676002
C	-1.1526518566	-0.0151199363	2.2087346435
C	-0.6338147008	0.008971472	3.5229147467
O	0.7331997052	-0.0377111363	3.5221322141
C	-1.4333061041	0.0751374442	4.6572231897
C	-2.8140669189	0.1344323449	4.4549515483
C	-3.3602956111	0.1305805212	3.155084286
C	-2.5331792671	0.0444320125	2.0255048729
P	-5.137529685	0.3249686478	2.889181942
N	-5.8457495057	0.1656429189	4.3236784361
P	-7.3852944889	0.1513979023	4.7363774155
N	-8.0265582656	1.6994540763	4.7362065776
C	-9.4582084888	2.0115561218	4.6987974304
N	-5.5121287457	1.630182972	2.0313370472
P	-4.8823568391	2.9851249803	1.5004911704
N	-6.191905612	3.9606412243	1.1405240694
C	-6.0033349819	5.2261807357	0.4322671975
N	-3.8588042016	3.8908147347	2.4782051659
C	-2.5021186492	3.4292305922	2.808264925
N	-3.9311329083	2.7079797951	0.1505200863
C	-2.9719604099	3.6667326251	-0.409042219
C	-4.4369283253	4.7267267659	3.5344399917
N	-8.4667415216	-0.6905465308	3.7603593875

C -8.7038491702 -0.2434462458 2.3799524255
N -7.4285049968 -0.5163840468 6.2623206214
C -8.6628057298 -0.4906075151 7.0485676572
C -8.5863271977 -2.145145187 3.9157596892
C -6.4244842103 -1.4669422904 6.7497743613
C -7.2148538556 2.7713784523 5.3217619325
C -4.3583557566 1.6959648206 -0.8198827397
C -7.5005607113 3.8188441678 1.7762944668
H 1.69692113 -0.2146040535 -1.5971683306
H -2.9528067155 0.0477090222 1.023057116
H -0.6366266127 -0.125796762 -0.7372827056
H 3.6188998818 -0.2270593307 -0.0425453841
H -3.4847312387 0.1899341379 5.3066145939
H -0.9981326973 0.0806150718 5.6511957916
H 3.2648319272 -0.1485515871 2.4363625036
H -3.4760088815 1.1708207471 -1.2070193064
H -5.0214093236 0.9692390193 -0.3480131831
H -4.8861549849 2.1539131725 -1.6686394783
H -9.7466794033 -0.4455807882 2.1081534013
H -8.0524210628 -0.7718203304 1.6691697904
H -8.5182078107 0.8266258189 2.2829203446
H -7.8327175221 -2.6851991472 3.3232830089
H -9.580686614 -2.4556475383 3.576180731
H -8.4796735386 -2.4334265615 4.9622007577
H -3.3560414792 4.1108522827 -1.3384486712
H -2.0326937617 3.148430935 -0.6405543521
H -2.7607675139 4.4661784032 0.3010763276
H -5.5296835407 -1.4136772102 6.130149914
H -2.1071294169 2.7873416761 2.0220497942
H -6.157789368 -1.2101402025 7.7819664772
H -6.8112912999 -2.4967253619 6.7380343259
H -1.8463720779 4.3017321153 2.9121933649
H -8.4232212402 -0.2635367317 8.0940297292
H -9.3454649796 0.2768713934 6.6822628994
H -9.1842450456 -1.458547474 7.0168002841
H -6.1558675149 2.5213332283 5.25236837
H -4.5172302469 4.1799323549 4.4852949186
H -7.4751347435 2.9395854122 6.3768487595
H -3.7919732589 5.5985870004 3.6927124937
H -7.3904910451 3.7001362232 4.7677682041
H -5.4289132738 5.081814615 3.2517113309
H -9.8233008748 2.3262978881 5.6870501764
H -9.6305088221 2.8368345286 3.995982358
H -10.0341509716 1.1464299252 4.3697688899
H -7.5374994204 2.8929181438 2.3493858565
H -6.0168874145 6.0835271851 1.1219693911
H -7.7022899506 4.6657077999 2.4506062149
H -8.285632855 3.8002234597 1.0094696038
H -6.8107786313 5.3586595866 -0.2977308645
H -5.055994059 5.2371649173 -0.1072408949
H -2.4887631455 2.8638054324 3.7489181403

H -5.4917535678 -0.7622098171 2.0597822439

IVb

C -0.0614448605 0.0445166734 0.0059992788
N -0.0539171495 0.0435002685 1.4876823331
C 1.3366344906 0.064565751 1.9978678071
C 2.2156818424 -0.0180051736 0.7374116462
C 1.320531405 0.6013073222 -0.3468668726
P -1.3386040094 -0.6773998192 2.318468237
N -2.6216084825 -0.8144972866 1.4312512322
P -3.8884537954 0.2194735753 0.9337182951
C -3.5127253683 1.9075236038 1.6897318654
C -2.3717616012 2.6328722159 1.276393858
C -2.0885417337 3.9226922026 1.7340020677
C -3.002527369 4.4866011005 2.6143738794
C -4.1621055826 3.8134289025 3.0352442816
C -4.4165405785 2.5197319535 2.5661348717
C -4.8244318774 4.7224637495 3.9533390541
C -4.006431815 5.8693862617 4.0056533492
O -2.8997334314 5.7383395404 3.1940510538
C -4.2988682488 6.9787647492 4.7874741905
C -5.4705901749 6.9199256328 5.550229741
C -6.3057006577 5.7879304822 5.5201117716
C -5.9916550664 4.6834540153 4.7248384135
N -0.6945196199 -2.1839154273 2.7612150318
C -0.1249926122 -3.0888586492 1.7538263411
C 0.4113592249 -4.24209776 2.6082901732
C -0.6741624566 -4.3888804912 3.6888411793
C -1.1796661652 -2.9470057441 3.9275911661
N -1.4932918906 0.1468595437 3.7743045366
C -0.6889533985 1.3094799366 4.1999108106
C -1.6111240688 2.0670749501 5.1663081363
C -2.4901550241 0.9594433194 5.7633553142
C -2.7402668508 0.0305500942 4.568039147
N -5.1912279711 -0.1631891408 1.9673496392
P -6.2549905751 -1.2932186475 1.7544894318
N -7.3794071094 -1.1163977223 2.9872142403
C -7.1679602357 -0.3123381187 4.2025674005
C -8.5350862976 -0.3694839176 4.8978142584
C -9.0434846794 -1.7757102506 4.541477565
C -8.5850168549 -1.9544449772 3.0836466977
N -5.8079919315 -2.9229785309 1.8646385464
C -4.9323974847 -3.5106002535 0.8151662226
C -3.8088023837 -4.2023433825 1.5959830077
C -4.5030932739 -4.6134641734 2.9018755655
C -5.3781958298 -3.391366568 3.203649567
N -7.0234889033 -1.2800527124 0.2654375039
C -7.411034118 0.0244852344 -0.3205879476
C -8.3123140732 -0.3594700049 -1.5044024598
C -8.9660339358 -1.6689327751 -1.0354856894
C -7.8066276244 -2.3826185349 -0.3282641373

H	-7.2086247446	5.7755022181	6.1249029162
H	-6.6392517532	3.8107093792	4.7019448573
H	-5.2966567059	1.9679926373	2.8798952065
H	-1.6732298648	2.1762744455	0.5836447941
H	-5.7386344287	7.7661509565	6.1772311105
H	-3.644886088	7.8450635547	4.8008158244
H	-1.199811393	4.4613468895	1.4190091487
H	0.6617815851	-2.5812475247	1.1875760581
H	1.6258255525	0.3414540092	-1.3659272137
H	-0.8789506253	-3.4457562816	1.0338763446
H	-1.4902765281	-5.0107540428	3.3039134374
H	0.5685758707	-5.1600262949	2.0317019779
H	1.3683379844	-3.9519707645	3.059890677
H	-0.3065858663	-4.8569468247	4.6079741082
H	-2.2748175918	-2.9304726673	4.010065005
H	-0.7735443561	-2.5075724313	4.847181408
H	1.5131096686	-0.763057846	2.6950924888
H	2.430604061	-1.0652337881	0.4885440252
H	3.1735620766	0.4977993535	0.8634161525
H	1.3196682172	1.6957503264	-0.2587618691
H	-0.4105320568	1.9275404768	3.3417805247
H	-0.8848319769	0.644730802	-0.3874468547
H	0.2321753968	0.9795392633	4.7046764482
H	-1.0496867372	2.630167874	5.9196641952
H	-2.2308456417	2.7715717088	4.6045815576
H	-3.425060373	1.3371651897	6.1909503794
H	-1.9452723564	0.4249643822	6.5530464212
H	-3.6115474616	0.3442110855	3.9853488539
H	-2.9144434206	-1.0038589554	4.8853508304
H	-3.0277490005	-3.4641751935	1.7980150028
H	-3.3689650482	-5.0438587034	1.0489630967
H	-5.5107267791	-4.2310400994	0.2198583012
H	-5.1301187026	-5.5005644225	2.7419526327
H	-4.5436363582	-2.7419037725	0.1395904388
H	-3.8094187382	-4.8346100577	3.7201489414
H	-4.7877633511	-2.6216210567	3.7236915058
H	-6.248192985	-3.6303998187	3.826398831
H	-6.5241524686	0.5888465428	-0.6233949938
H	-7.9646976169	0.6306340013	0.412750484
H	-8.3662215552	-3.0028755892	2.8438152895
H	-9.3557778532	-1.6073309676	2.3781046444
H	-10.1258721339	-1.8936904056	4.6586971483
H	-8.5520590685	-2.5214175276	5.1798834112
H	-9.2026182004	0.3914385068	4.4728401274
H	-8.4640243854	-0.1947669304	5.9766670158
H	-6.8572439955	0.7027359865	3.9457852743
H	-6.3817908609	-0.7475895987	4.8396095024
H	-9.0340811715	0.4268480049	-1.7487782957
H	-9.7677505495	-1.4539002168	-0.3161447639
H	-9.3913663691	-2.2627450254	-1.8519756427
H	-7.700934099	-0.5459053628	-2.3966616115

H -8.1348713739 -3.0974347812 0.4311116126
H -7.191970424 -2.9326508334 -1.0562955187
H -0.1863933219 -0.969903155 -0.4010607345
H 1.5273632127 1.0009306183 2.5380795138

IVbH+
C -0.050650269 0.0064623188 0.0155453226
N -0.0488565152 0.0266595196 1.5027897371
C 1.3408804068 0.0441291084 2.0222528626
C 2.1996282268 -0.3229634289 0.8061306234
C 1.4105764237 0.2983747406 -0.3550222846
P -1.3035010587 -0.6241770712 2.3997239752
N -2.5549736677 -0.9155162736 1.4598306809
P -3.6755088463 0.019020164 0.7625590331
C -3.700615848 1.7349730072 1.367092654
C -2.6421126838 2.6151081525 1.055680826
C -2.619727699 3.9274470749 1.5296385986
C -3.6846277049 4.3214609227 2.3306073727
C -4.7600284446 3.4688018776 2.6581897658
C -4.7707726767 2.1639115212 2.1627498278
C -5.6357576003 4.2568633704 3.5049363747
C -5.0140057209 5.5145142954 3.6168943068
O -3.8287039134 5.5570017112 2.9025584043
C -5.5389965523 6.565522855 4.3552031127
C -6.7505712969 6.3215629041 5.008582897
C -7.3972743168 5.0742822046 4.9166308061
C -6.8495742922 4.0333119958 4.1667577573
N -0.8195020811 -2.0637657793 3.0883908245
C -0.4370597292 -3.2122302814 2.2382507823
C 0.613888488 -3.9496285394 3.0777753181
C 0.1306102802 -3.6996253035 4.5145266733
C -0.3500952108 -2.2399725977 4.4821815878
N -1.5694507868 0.3905885818 3.6835248971
C -0.8249873632 1.6332557809 3.9937041684
C -1.7675755263 2.3854173409 4.9435001828
C -2.5248001714 1.2592392504 5.6638643326
C -2.7610863148 0.2346421239 4.5471013065
N -5.1840064905 -0.4995278197 0.8594111022
P -5.8966651328 -1.918144476 0.96752205
N -7.4953379456 -1.5465153739 1.2153777009
C -8.0365656388 -0.198744456 1.4996532403
C -9.55158069 -0.4000356759 1.3669172775
C -9.7569337159 -1.8413054758 1.8592826903
C -8.5454057157 -2.5848265048 1.2750893836
N -5.4866309007 -2.9551973299 2.2059248123
C -4.2640421157 -3.803346194 2.1804121081
C -3.7783585072 -3.809344625 3.6375369747
C -5.0647817722 -3.584971057 4.4457165129
C -5.8195660766 -2.5579665847 3.5952325288
N -5.6363722351 -2.8412940703 -0.3941342407
C -5.7139374698 -2.1669268748 -1.7200397781

C	-5.9176312351	-3.3216716501	-2.7126778815
C	-6.6971260662	-4.3579739116	-1.8895505589
C	-6.0137802339	-4.2736465479	-0.5205901377
H	-8.3386974414	4.9243291967	5.4371461854
H	-7.3535151307	3.0730602073	4.0954031555
H	-5.5933168621	1.4875729954	2.3724702546
H	-1.8192814395	2.2760976862	0.4340213617
H	-7.2024431921	7.1138507508	5.5983346706
H	-5.0316407756	7.5226338377	4.4157624991
H	-1.8128918	4.6117411182	1.288502168
H	-0.0459704765	-2.8728704146	1.2726743835
H	1.6745802657	-0.1150243394	-1.3328977755
H	-1.3086398614	-3.8492100157	2.040744846
H	-0.710271834	-4.3641689781	4.7471145555
H	0.6853412362	-5.0112558528	2.8228814074
H	1.6023828484	-3.4976893348	2.9258789415
H	0.905665831	-3.8605502341	5.2695751216
H	-1.1564246144	-2.0582390942	5.1999399042
H	0.467271225	-1.547179597	4.7228546251
H	1.4590438409	-0.6625532145	2.849317374
H	2.2451401805	-1.4138036601	0.6936734268
H	3.2240736186	0.0524984427	0.8866035178
H	1.5833201064	1.3812309023	-0.3888257668
H	-0.6096987313	2.1965677594	3.0820169076
H	-0.7353048388	0.7553704761	-0.393693503
H	0.1269875285	1.3945704255	4.4877832369
H	-1.2243468214	3.0460548061	5.6255853299
H	-2.4673683002	2.9949680401	4.3630620133
H	-3.4606571957	1.5936768379	6.1215232175
H	-1.8982463713	0.8204313017	6.4503295321
H	-3.6746275876	0.4668766927	3.9845469545
H	-2.8521957336	-0.789886529	4.9241847001
H	-3.0810361657	-2.9799966923	3.7897746554
H	-3.2627886843	-4.739566744	3.8973120902
H	-4.5312357878	-4.8138367079	1.8480664659
H	-5.6435019082	-4.5142878485	4.5135057008
H	-3.5172661593	-3.3934661707	1.4960465099
H	-4.8829712839	-3.2249777923	5.4633965214
H	-5.4607410092	-1.5399218133	3.8123639887
H	-6.9024326021	-2.5780657326	3.7532930929
H	-4.8014613529	-1.5953181796	-1.9160497787
H	-6.5626098035	-1.4694996162	-1.7509618211
H	-8.2311929507	-3.4323694885	1.8960943752
H	-8.7638532393	-2.968080061	0.2680351874
H	-10.7070775148	-2.2803186294	1.5411475691
H	-9.7214859026	-1.8725813732	2.9556213414
H	-9.8491494551	-0.3134353163	0.3144816442
H	-10.120562253	0.3378364101	1.9407115661
H	-7.6321180602	0.5379252047	0.8023241565
H	-7.7708029697	0.1170630804	2.5202016627
H	-6.4418683523	-2.9979780245	-3.6166609675

H -7.7505579177 -4.0612899612 -1.8049059309
H -6.6615224494 -5.3661355827 -2.3137212761
H -4.9479493025 -3.7359115245 -3.0154513253
H -6.6524013965 -4.5935181797 0.3068565196
H -5.1064175969 -4.8916278676 -0.5026373323
H -0.3707166061 -0.9719110782 -0.3641449114
H 1.5918024367 1.0459617243 2.395011921
H -3.311318222 0.1495127969 -0.5975146996

Va
C 0.2928489102 -0.1851076395 -0.4167213868
C -0.0096500357 -0.0224199298 0.9105499668
C 0.756714398 0.8133488118 1.7765791966
C 1.8459417952 1.5167176137 1.3211330704
C 2.1665099102 1.3501373909 -0.0556246193
C 1.4373693058 0.5178036025 -0.9083032711
C -0.9076324737 -0.3942294422 2.0671085627
C -0.1407920798 0.4441997985 2.931099826
C -0.4372854818 0.587068087 4.2661266982
C -1.5577902892 -0.1532441544 4.7375107481
C -2.3055183984 -0.9711749791 3.8946298044
C -1.9909884648 -1.1124780275 2.513456225
H -3.1538211184 -1.5163772083 4.3012184091
P 1.8369800837 0.367479985 -2.7298885238
N 1.7928768092 -1.3091984392 -2.9359656668
P 2.5377461248 -2.5135679129 -2.2590819947
N 3.451852311 -2.4094400862 -0.8387289314
C 4.7178974504 -1.6699034273 -0.8490233943
N 3.4864285597 0.8372814452 -2.6799501221
P 4.1434937495 1.8868754853 -3.6406953705
N 3.2199672877 2.9931985463 -4.5359730732
C 2.4215106512 3.964855635 -3.7767210015
N 5.1095155095 2.9434491194 -2.717137402
C 5.8002568847 2.4116584329 -1.5440034414
N 5.0439882675 1.1210985146 -4.86080459
C 5.4788511749 -0.2603042107 -4.6730298074
C 5.7923893769 4.0918291403 -3.306330594
N 1.3870245642 -3.6883747185 -1.8503667932
C 0.0311179152 -3.6376239268 -2.3866893833
N 3.7091280183 -3.1303265941 -3.3401605147
C 3.3866717938 -3.0940494017 -4.7673396172
C 5.9029001067 1.8869833729 -5.758098517
C 1.7734860861 -4.9999854887 -1.3456364371
C 4.5625398889 -4.2632451756 -2.9895898241
C 2.7987360819 -2.3629734687 0.473749624
C 2.5227605202 2.5339577499 -5.7438116824
H 2.4495444819 2.1605403647 1.9567519174
H 3.0266585063 1.8711829874 -0.4654312224
H -1.8373705027 -0.0764135949 5.7854446723
H 0.1337592271 1.2209882064 4.9393176501
H -0.2736287667 -0.8353799514 -1.0776396032

H -2.5869703404 -1.753750462 1.8694231263
H -0.6912219696 -3.8693294008 -1.5904120875
H -0.1689336237 -2.640265576 -2.7787235094
H -0.1069393506 -4.3704146855 -3.1985385538
H 2.3544036197 3.3966826222 -6.4021893792
H 1.5529237774 2.0770948939 -5.5005439309
H 3.1292829741 1.7997548079 -6.2759983218
H 1.4482837069 3.5448204574 -3.4835460457
H 2.2490338882 4.8506615346 -4.4022207264
H 2.9540428084 4.2726365158 -2.8748639464
H 1.8044435685 -5.7582734667 -2.1450895871
H 1.0449483417 -5.3327849332 -0.5929940328
H 2.7535041143 -4.955713024 -0.8668818579
H 5.2380325613 1.5727705291 -1.1340134587
H 1.8185431045 -2.8380912863 0.4325094014
H 6.8211597032 2.0707903949 -1.7880753913
H 5.8783018507 3.1965782839 -0.7793897706
H 3.4232367612 -2.9007260751 1.2006673527
H 6.8126776548 3.8458685878 -3.6435867661
H 5.2257322855 4.4799154447 -4.1543475792
H 5.8709691199 4.887418443 -2.5527754642
H 4.7837790854 -0.7851452853 -4.0193782277
H 4.5574625872 -0.6099623193 -0.6166232506
H 6.490840953 -0.3156141678 -4.2360081994
H 5.3913249895 -2.109537191 -0.1004427894
H 5.5014400155 -0.7676176216 -5.6469543012
H 5.1909023845 -1.7308511156 -1.8292600471
H 6.9277371113 1.9928701481 -5.3654861999
H 5.9650572381 1.37395436 -6.7273685992
H 5.4883192611 2.8825703442 -5.9291194031
H 2.8055459732 -2.1989501665 -4.9908749401
H 5.50350926 -4.1910231497 -3.5518434837
H 4.3190386062 -3.0697246731 -5.3460256662
H 2.8118961392 -3.9819370803 -5.0826204502
H 4.0964536914 -5.2317057586 -3.2361937609
H 4.7994703771 -4.2503610641 -1.9244202936
H 2.6660216777 -1.3303708277 0.8194574709

VaH+
C 0.4977282079 -0.1633428024 -0.0428346575
C 0.2225874938 -0.239374633 1.2964339583
C 1.0790077919 0.3371748088 2.2860033948
C 2.2257933744 1.0193126086 1.958884128
C 2.521784921 1.1085900251 0.5706432697
C 1.6991670439 0.5316579945 -0.3955078633
C -0.7002133813 -0.7276094542 2.3856753058
C 0.1545297613 -0.1539395712 3.3694617514
C -0.1140500332 -0.2315226226 4.7165033974
C -1.303181706 -0.9240393561 5.0657658683
C -2.1412555106 -1.4850289386 4.1020455713
C -1.8555884707 -1.3978341706 2.713552483

H	-3.0413289884	-2.0021785685	4.4229065954
P	2.1600862665	0.5785171593	-2.1419233382
N	2.4026352226	-0.8752989567	-2.7799224909
P	2.5888490008	-2.3973958903	-2.3797295294
N	3.5250594534	-2.8172480016	-1.0487081877
C	4.9853029333	-2.8196691016	-1.1726313466
N	3.3560395823	1.6426246771	-2.289741396
P	4.1447700598	2.1719436414	-3.5681153929
N	3.2677988175	2.5228299985	-4.9612101235
C	2.5668791704	3.8092916174	-5.0542460037
N	4.9112503788	3.5654390748	-3.0702447913
C	4.4095091908	4.4107761752	-1.9827802957
N	5.2502466704	1.0359539912	-4.1128512079
C	6.0570634142	0.3376379618	-3.1066617129
C	5.8984007893	4.2175282579	-3.9318174991
N	1.1155240138	-3.1019049875	-2.0143097123
C	-0.0683396907	-2.7115048087	-2.7853267745
N	3.2964674339	-3.1149065798	-3.7144758786
C	4.1163009592	-2.3849602086	-4.6794776728
C	5.8612267383	1.0367482578	-5.4452255226
C	0.9486668017	-4.380264326	-1.3152869019
C	3.4262182425	-4.5692108441	-3.7957355467
C	3.0779378073	-2.5246116136	0.321344719
C	2.5673014835	1.4417934131	-5.6701586272
H	2.8819769804	1.4740900935	2.6950563901
H	3.413760971	1.6366729914	0.2461613433
H	-1.5694739917	-1.017501012	6.1149171009
H	0.5243886196	0.2024822074	5.480428894
H	-0.1377561442	-0.6081662595	-0.803855262
H	-2.524795238	-1.8360449772	1.9785704653
H	-0.9378999811	-2.6696997623	-2.1175557448
H	0.0760208726	-1.7283666499	-3.2358729058
H	-0.2810223373	-3.4364241015	-3.5840500341
H	2.5593231555	1.6604449621	-6.7445020408
H	1.5265616206	1.3452198008	-5.3288295228
H	3.0686644202	0.4867868692	-5.5111884585
H	1.5728630454	3.7711370328	-4.5839068998
H	2.4393309665	4.0656776565	-6.1117977019
H	3.1469041611	4.6020117836	-4.5802273972
H	0.6245394214	-5.1690074775	-2.0091705116
H	0.180970162	-4.2729286754	-0.5383615207
H	1.8804114522	-4.6896968845	-0.8417971295
H	3.6996396461	3.851393546	-1.3739176118
H	1.9906978705	-2.4832743658	0.3750474706
H	5.250974761	4.7220946802	-1.3522274806
H	3.9173709891	5.3136053854	-2.3739590453
H	3.4340811906	-3.318152299	0.9889190163
H	6.7428959015	4.5610415285	-3.3228012078
H	6.2806770406	3.5263323295	-4.6836880513
H	5.4712092884	5.0881693131	-4.4508347128
H	5.5204070436	0.2938767226	-2.158519789

H 5.4161676924 -1.8405594117 -0.9165425104
H 7.0229786208 0.8392205895 -2.9491945474
H 5.3998088151 -3.5673047072 -0.4867598624
H 6.2482597806 -0.6864206683 -3.4458461308
H 5.2903049588 -3.0805590875 -2.1870024096
H 6.8979301526 1.4013932869 -5.4084020722
H 5.877269646 0.0113196768 -5.8363501248
H 5.2913776811 1.6634456234 -6.1314962076
H 4.0261830303 -1.3127968004 -4.5081834634
H 4.4405188319 -4.902784554 -3.5293769102
H 5.1754493101 -2.6737386375 -4.5927886909
H 3.7831734133 -2.6166043515 -5.6993812465
H 3.2144712201 -4.8966402894 -4.8206364013
H 2.7181323636 -5.0643947003 -3.1308305858
H 3.4719705948 -1.5627867768 0.6736771067
H 1.0040302072 1.0679819044 -2.7905235561

Vb
C -0.0090433099 -0.0178232043 -0.0014807622
N 0.0044012306 0.0111641926 1.4673287621
C 1.37409411 0.0041866294 2.005476442
C 2.2499376905 -0.2375350602 0.7628043548
C 1.4255015225 0.3738582279 -0.3816748148
P -1.4105878018 0.2706285347 2.3489011298
N -0.8546166238 0.3376145175 3.9285815389
C -0.7579638988 1.5255354023 4.7953291883
C -0.6320804308 0.9387572944 6.2110514796
C 0.0311611314 -0.4272410766 5.9759151616
C -0.6398620508 -0.9118307285 4.6851761619
N -2.467064133 -0.8179456724 1.9907152771
P -4.1022477804 -1.12115187 2.4262525817
N -4.176543842 -2.7889872142 2.6665674487
P -3.8135160367 -3.9977565068 1.748790037
N -2.2012241232 -4.4157049286 1.4521458891
C -1.3692376507 -3.6227112789 0.5084585734
C -0.0225244466 -3.4497472391 1.2267736164
C 0.0463308602 -4.6695481598 2.1564253119
C -1.4022580838 -4.7956005376 2.6398057536
C -4.1694895949 -0.5993298061 4.2316465886
C -4.268796868 -1.5263075782 5.272695959
C -4.168019588 0.799810528 4.5215238277
C -4.2264351828 1.1606053113 5.8428827553
C -4.2075848713 2.2780533901 6.8583695515
C -4.1302743306 3.6287690867 7.0992871671
C -4.1473130011 4.0196503957 8.4684246724
C -4.2350598675 3.0855745358 9.496790572
C -4.3117662847 1.6876472041 9.237765911
C -4.2963346035 1.3192376082 7.9133167979
H -4.0905167264 5.0768083411 8.7163066582
C -4.3109131256 0.2036427233 6.8971647978
N -4.4095550192 -3.901450104 0.1791903493

C -5.7989798859 -3.4106168895 0.00777306
C -6.1453720535 -3.7883335259 -1.4398476646
C -5.34380972 -5.0808472926 -1.659699577
C -4.0187382636 -4.7702054993 -0.9514740179
N -4.416718471 -5.3574574191 2.5256983863
C -4.986067811 -5.3592119079 3.8839775043
C -5.6168775454 -6.7543533469 3.9934069285
C -4.6732050521 -7.6261930677 3.1488179789
C -4.3250019895 -6.7112823438 1.9621274486
N -1.9191805163 1.8520052885 1.995665456
C -0.9623034304 2.9700560769 1.8720895069
C -1.418661613 3.728603167 0.6182722169
C -2.9408107302 3.5279593581 0.6529827387
C -3.0912058641 2.0697579343 1.110190064
H -4.2451109956 3.43205942 10.5273771356
H -4.2764275217 -2.5791820983 5.0049090136
H -4.408644412 -1.9006351088 7.4231249005
H -4.3774120546 0.9735510466 10.0544479573
H -4.0964836513 1.5326003408 3.7232101346
H -4.0605593593 4.3717671284 6.3090021392
H -0.7566578245 0.6777342207 -0.4000099496
H -3.4289819304 3.7099330349 -0.3101111711
H -0.2594274244 -1.0208533668 -0.3757346265
H 2.3710464114 -1.3152430309 0.5974296035
H 1.7175968782 0.0067658851 -1.3713722484
H 1.5288329253 1.4670764618 -0.3802576032
H 3.2476974892 0.2017382551 0.8663289946
H 1.512848548 -0.781732745 2.757271601
H 1.6238483552 0.9610921785 2.4879775858
H 0.0649313912 2.5996509837 1.7887261727
H -0.9960529405 3.2581035699 -0.2793580488
H -1.1142688253 4.7808938809 0.6251074633
H -3.3894391895 4.2054194567 1.3914168808
H -1.6413030264 2.1630635805 4.690765301
H -4.0325338477 1.8926644793 1.6393981319
H 0.1284506518 2.1256067315 4.536526267
H -0.0618589138 1.5913872251 6.8803249022
H -1.6278647533 0.7973727351 6.6417002176
H -0.1152482922 -1.1254213497 6.8067513264
H 1.1110988198 -0.3052760723 5.8172126997
H -1.5955279748 -1.4091620988 4.891778237
H -0.0138651436 -1.6158390884 4.1233062576
H -0.0487284095 -2.5256782981 1.8106717743
H 0.8189637886 -3.3899988806 0.5272408307
H -1.2486108854 -4.1842358084 -0.4283284855
H 0.3369587083 -5.5671546614 1.5945962804
H -1.8345796457 -2.6583672196 0.28812566
H 0.7507608544 -4.5458189497 2.9865968661
H -1.5875804298 -4.1076575198 3.4791718664
H -1.6592521282 -5.8094653823 2.9678288112
H -5.8549114542 -2.335083533 0.2004858278

H -6.4792602237 -3.9138947867 0.712203767
H -3.3236067831 -6.9097729611 1.5585974065
H -5.0454096319 -6.8430229117 1.1389563301
H -5.1184052848 -8.5750438349 2.8310132476
H -3.7649087445 -7.854805173 3.7215879163
H -6.6208944275 -6.7469843219 3.5492221373
H -5.7076666201 -7.0934008919 5.0308873301
H -5.7040277614 -4.5445724764 4.0049637097
H -4.1985799843 -5.2236861024 4.6425677543
H -7.2231204108 -3.9102174379 -1.5905905023
H -5.8457916074 -5.9235614737 -1.1654515107
H -5.2072674533 -5.3381965984 -2.7158797487
H -5.7937611934 -3.008621388 -2.1280905848
H -3.4888966379 -5.6636359427 -0.6090842286
H -3.3435159845 -4.2242711559 -1.6268297903
H -3.0763679017 1.3793049422 0.2567667523
H -1.0116593739 3.6199742304 2.7583038314
C -4.3425539644 -1.1466761752 6.641832208

VbH^+
C 0.0026353436 -0.0700222014 -0.0082043289
N 0.0112887534 -0.0405089898 1.4692165513
C 1.3900114304 0.02274219 2.0054229037
C 2.2660098969 -0.2211754285 0.7658499622
C 1.4276259195 0.3549923145 -0.3859068418
P -1.3752461383 0.2961632219 2.3307464142
N -0.8990405087 0.3673558938 3.9162542296
C -0.7250973921 1.5936114935 4.7257740496
C -0.5389899432 1.054098912 6.151586306
C 0.1305047527 -0.3113551838 5.9318869637
C -0.5965526679 -0.8545758712 4.6954672944
N -2.4723301711 -0.8063948049 2.0004678119
P -3.8985689057 -1.2300061533 2.6378417415
N -4.2300534121 -2.7932710114 2.6303709752
P -3.8062882906 -4.0226336884 1.7116580475
N -2.1914313773 -4.3774192223 1.5024388394
C -1.3185815224 -3.6491076308 0.5425642763
C 0.0242169289 -3.5217469967 1.2756620309
C 0.0388625122 -4.7436134269 2.2057934642
C -1.4142097815 -4.8164566904 2.6876960882
C -4.127040821 -0.650715215 4.3452640321
C -4.2705833199 -1.5810848264 5.3744221224
C -4.0925345494 0.7543057591 4.6142616036
C -4.1883691594 1.1178135495 5.9303997083
C -4.1878271728 2.2317409821 6.948035551
C -4.1114750887 3.5834360458 7.1889219715
C -4.1675360675 3.9684164057 8.5553657947
C -4.2916283122 3.0324946076 9.5816545975
C -4.3695086772 1.638196384 9.3223166032
C -4.3146694551 1.2755292119 7.9965987999
H -4.1143693109 5.0239327228 8.8078198846

C	-4.3153958817	0.1594235988	6.9827394784
N	-4.3515265075	-3.8371810017	0.1475087304
C	-5.7346062903	-3.3212997745	-0.0506774727
C	-6.0830480751	-3.7347533399	-1.4886714924
C	-5.292762655	-5.0378269325	-1.6792042446
C	-3.965039387	-4.7235096671	-0.9817912381
N	-4.4504637999	-5.3430036715	2.4829470826
C	-5.0875811089	-5.3397454345	3.8196221847
C	-5.7113377356	-6.7388912802	3.9094872598
C	-4.7352840602	-7.6108121144	3.1040205922
C	-4.3585698075	-6.7064477803	1.9205847531
N	-1.9224984063	1.8185623502	1.8960675617
C	-0.9876915331	2.9497977324	1.6836931897
C	-1.555595898	3.6778613671	0.4594552169
C	-3.0679483563	3.469121801	0.622423574
C	-3.1755459964	2.0203284307	1.1214099299
H	-4.3321620065	3.3799037408	10.6103115449
H	-4.3066169774	-2.6347081143	5.1152268701
H	-4.472549228	-1.9375483694	7.5209673968
H	-4.467722955	0.9240392296	10.1347409221
H	-3.9715087361	1.4807244974	3.8171816388
H	-4.0186760522	4.3283907309	6.4035702209
H	-0.7626406741	0.6053290698	-0.4075167565
H	-3.6322338195	3.6260445546	-0.30159793
H	-0.2203779842	-1.0814070287	-0.371376305
H	2.4127453911	-1.2980364	0.6190220171
H	1.7298444898	-0.0166596795	-1.369585388
H	1.5033514222	1.449695234	-0.4001678568
H	3.252099994	0.2427711139	0.8612940815
H	1.5530421953	-0.7351599682	2.7785287483
H	1.6000466648	1.0036635594	2.4533591918
H	0.032781909	2.5872992616	1.5248567162
H	-1.2022220948	3.1971451371	-0.4616746832
H	-1.263636266	4.7316979536	0.4247005956
H	-3.4620495551	4.1592287028	1.3787050814
H	-1.5980181953	2.2464430846	4.6349148571
H	-4.0627113341	1.8689094621	1.7448550737
H	0.1610986006	2.1553513897	4.3974550841
H	0.0525253489	1.7321412505	6.7736120825
H	-1.5159365124	0.9209790058	6.6276050462
H	0.0376973402	-0.9804149864	6.7926096
H	1.1982266684	-0.1815949564	5.7145990462
H	-1.5262271346	-1.3657949717	4.9741754531
H	0.0142944284	-1.5580574132	4.1185869111
H	0.0316895134	-2.5953334812	1.8581873578
H	0.8725018097	-3.4960826186	0.5839212212
H	-1.2133115605	-4.243183912	-0.3737775502
H	0.2946291416	-5.6502768756	1.6438388126
H	-1.7462778103	-2.6774761914	0.2854095416
H	0.7450821897	-4.6509666256	3.0369804123
H	-1.5736571803	-4.1352679065	3.5374993647

H -1.7165470164 -5.8222074814 2.9963229367
H -5.7674001099 -2.2375536843 0.0986892943
H -6.4271223281 -3.7821105484 0.6675191519
H -3.3517746848 -6.9097059588 1.5367104004
H -5.067454503 -6.8282942257 1.0888058523
H -5.1658951628 -8.5627097424 2.7795682068
H -3.8437257508 -7.8309032331 3.7047128013
H -6.6996769881 -6.7394119458 3.4330369511
H -5.8350863088 -7.0718605764 4.9444473319
H -5.8195010656 -4.5330151806 3.8999879701
H -4.3324102309 -5.1935552356 4.6060084625
H -7.1611794666 -3.8526563712 -1.6320360981
H -5.7998553336 -5.8672594804 -1.1694909944
H -5.1588097993 -5.3157911369 -2.7291666921
H -5.7298579137 -2.975078493 -2.1970617407
H -3.4362750279 -5.6129638912 -0.629482765
H -3.2920365989 -4.1790020262 -1.6572556436
H -3.2350484267 1.3117432554 0.2856656826
H -0.979744143 3.6062716163 2.5640823495
C -4.3672740478 -1.1913113667 6.7388234702
H -4.8985381491 -0.5436349315 1.9092517333

VIa
C 0.0025187988 0.1432664461 -0.0898169059
C -0.0097128248 -0.0044892488 1.4234286362
C 1.3215253919 -0.0179036944 1.8890200615
C 2.2685805812 0.1106723996 0.8464570211
C 1.5241067941 0.2180398362 -0.4754259899
C 1.6778383403 -0.1390069817 3.2519178129
C 3.0725181946 -0.1173200707 3.5423831304
C 4.0093733433 0.0224692862 2.5295729174
C 3.6070770571 0.128497788 1.160776706
C -1.0356071237 -0.1159619465 2.3391967927
C -0.7094333758 -0.2421186637 3.7207493217
C 0.6007275676 -0.2554817954 4.1744476863
P 5.8083263447 0.1789344041 3.0125405217
N 6.4814046896 -0.6492526486 1.6918685924
P 7.9766872342 -0.7693607417 1.2647844685
N 8.521940208 0.5966571107 0.4029251239
C 9.8775791311 0.6804025831 -0.1354120129
H -1.5198644994 -0.3311140186 4.4408224424
N 6.1026563809 1.8108622996 2.618674682
P 5.5952683139 3.2431176168 2.9198662022
N 6.7019218025 4.3201834532 2.1999061401
C 6.5346213122 5.7631496449 2.3341154361
N 4.1291540686 3.8274743991 2.2836005646
C 2.857567236 3.3998696248 2.883014753
N 5.3610455207 3.5608295496 4.5776263846
C 4.9000419577 4.8551756808 5.0741906901
C 4.0304907394 3.9201845476 0.8234023559
N 9.2297248527 -0.9311416486 2.4117478867

C 9.5805343535 0.2459893762 3.2177281599
N 8.1361407182 -2.2042945017 0.3783069157
C 9.4019617053 -2.5807736245 -0.2381096607
C 9.2042273953 -2.1530203345 3.2263003633
C 6.965088098 -2.8166152355 -0.2447800056
C 7.5393853634 1.3234163486 -0.403894952
C 6.1830256356 2.8385209452 5.5474286349
C 8.0952707001 3.9072081278 2.055260607
H 4.3783393487 0.214692801 0.3991706504
H -2.0788658133 -0.1097280835 2.0307207084
H 0.8106128424 -0.3536597377 5.2374761278
H 3.4063573848 -0.200523125 4.576427933
H 5.6168525249 2.7199079328 6.4810047834
H 6.433913688 1.8452050264 5.1724161328
H 7.1157469947 3.3799432029 5.7812706824
H 10.5811498997 0.0957978158 3.6434502378
H 8.8692641969 0.4067297904 4.0421259571
H 9.5939510364 1.1429874587 2.5989188348
H 8.4674370191 -2.0864332182 4.0418086147
H 10.1984511822 -2.3064700119 3.6648567133
H 8.9584016788 -3.0161154592 2.6050115092
H 5.7391028014 5.5210373954 5.3346479429
H 4.2964856965 4.7039894938 5.9800462958
H 4.2777108566 5.3539129004 4.3296460233
H 6.0668860136 -2.5205672502 0.2978028177
H 2.9764303342 3.2168576567 3.951083431
H 6.8623029007 -2.5184690629 -1.3017393268
H 7.0654956179 -3.9102908063 -0.2067045708
H 2.1121315845 4.1939934732 2.740525575
H 9.4859101137 -2.2241983193 -1.2781207992
H 10.2405774181 -2.1855741258 0.3386930473
H 9.4862784343 -3.6761785595 -0.2515069521
H 6.5920032471 1.3752403037 0.1303204912
H 3.7708039661 2.9498856562 0.3728123272
H 7.3882759889 0.8579960453 -1.3930408568
H 3.2459332551 4.6436936737 0.5656210785
H 7.8997898895 2.3479942285 -0.5597282434
H 4.9762620244 4.2626041381 0.3995224237
H 9.9509998395 0.2654518979 -1.1541307017
H 10.1828462135 1.734949444 -0.1798216724
H 10.5799957699 0.1494705395 0.5097102211
H 8.1416830964 2.8302549114 1.895372507
H 6.9899539557 6.2618699948 1.4676188867
H 8.533027929 4.4181153063 1.1862897341
H 8.6997980388 4.1675563117 2.9416457297
H 7.0170196832 6.1610244431 3.2427858372
H 5.4745545548 6.0235611199 2.3570856511
H 2.4814300237 2.4803663102 2.4190706046
H 1.7605004644 1.1574645926 -0.9911218084
H -0.4933526199 -0.70526955 -0.5773917215
H 1.8047169213 -0.5928294281 -1.1588080263

H -0.5367222954 1.0446898496 -0.4070115314

VIaH^+

C 0.0410675373 0.0010314812 -0.088398684
C 0.0128980708 -0.0929138683 1.4270879457
C 1.3377782452 -0.0440974804 1.9128217414
C 2.2900984065 0.0812124844 0.8775750901
C 1.5634915639 0.1139975129 -0.4559465826
C 1.6728496995 -0.1114888683 3.2862374224
C 3.0562871374 -0.0355103939 3.5974368471
C 3.9974928718 0.0925604712 2.5820665531
C 3.6278343365 0.1476279659 1.2030609467
C -1.0179819869 -0.2098268588 2.3349588973
C -0.7097577371 -0.2792982696 3.7250194446
C 0.5902624388 -0.2340181307 4.2007376307
P 5.7347870677 0.2552490681 3.0703477365
N 6.6138003295 0.016961474 1.7408968332
P 8.1849524161 -0.1916456967 1.5794637549
N 8.9660982434 1.3022193136 1.5596483042
C 10.4297196136 1.414522313 1.5279416977
H -1.5285054064 -0.3741254844 4.4334931446
N 6.0102264359 1.5794481183 3.9414544164
P 5.4269106387 3.0574193312 3.984996632
N 6.7195323477 4.1072742839 3.7168928597
C 6.5095864178 5.5589367887 3.7766876994
N 4.2980045159 3.5904148017 2.8729341187
C 2.8583003699 3.3373257711 3.0227606263
N 4.6480388357 3.2555397958 5.4560817551
C 4.08546613 4.5501102504 5.8519533333
C 4.6872654013 3.8436016897 1.4808369642
N 9.0352723349 -1.0245299156 2.7629114519
C 9.2809386833 -0.4093823549 4.0754371806
N 8.3968641617 -1.0959265543 0.1937688585
C 9.7433703106 -1.3178942258 -0.3384454227
C 8.9305589038 -2.4884735049 2.8354338617
C 7.3143904586 -1.3960136021 -0.7465283201
C 8.3041024644 2.4031804365 0.8491580458
C 4.8661783294 2.344472771 6.5824216555
C 8.0651522194 3.704838409 4.141312484
H 4.4042751874 0.2346098299 0.4487923152
H -2.0558405399 -0.2520277938 2.0143972521
H 0.7863138042 -0.2946806027 5.2685293598
H 3.3693983427 -0.0647551049 4.6401557647
H 3.9084104987 2.1533540041 7.0828638733
H 5.2768846655 1.4001160456 6.2263108311
H 5.5598265609 2.7778546787 7.3175113152
H 10.2417223954 -0.7663055965 4.4645145768
H 8.4949050794 -0.6751929223 4.7964711585

H 9.3189897335 0.6762451972 3.9920085886
H 8.0987242977 -2.8019984303 3.4831142911
H 9.8622130558 -2.8900513384 3.2498259546
H 8.7790184567 -2.9107494755 1.8414965249
H 4.7713704133 5.1053700886 6.5073425654
H 3.1478450242 4.3851669201 6.3967896246
H 3.8624713354 5.1615775667 4.9763704031
H 6.350020138 -1.2569443407 -0.259445975
H 2.6217365762 3.035704582 4.0422107848
H 7.3679539182 -0.7477246563 -1.6330118631
H 7.4006107493 -2.4396028162 -1.0740887052
H 2.3060872774 4.2554860005 2.7860183767
H 9.9832011827 -0.6091462501 -1.1439676722
H 10.4915346716 -1.2230503086 0.4510727718
H 9.8099176214 -2.3346351363 -0.7437797457
H 7.2227693819 2.3041773132 0.9289840519
H 4.5079084751 2.9583476875 0.8543077601
H 8.5864081741 2.4273085372 -0.2145896376
H 4.088482655 4.6737193926 1.0874111618
H 8.5997498166 3.3524606989 1.3080627106
H 5.7408617226 4.117254254 1.4215353909
H 10.815064892 1.4430672146 0.4982998874
H 10.7231974276 2.3450365303 2.0285290666
H 10.8948864916 0.5802804075 2.0543289117
H 8.2245830942 2.6524004982 3.912314658
H 7.2614620986 6.0512755904 3.1491771688
H 8.7978328031 4.3009026238 3.5851973942
H 8.2319028343 3.8763003209 5.2159478063
H 6.6072819095 5.9505505042 4.799929866
H 5.5229239961 5.8207999545 3.3905982359
H 2.5312915356 2.5430307756 2.3431234021
H 1.7782094027 1.0391639839 -1.0041151152
H -0.4181153874 -0.8808752471 -0.5497564348
H 1.8837300279 -0.7130458482 -1.1004794409
H -0.5238225556 0.8704938772 -0.4445234985
H 5.9587956667 -0.7981376183 3.9809675134

VIb
C 0 -0.0627326401 0.1755482265 -0.0003918703
C 0 -0.0398786993 0.0609357919 1.516179127
C 0 1.2995350925 -0.0934718012 1.9366861246
C 0 2.2150426645 -0.0957608366 0.8646389409
C 0 1.4432518648 0.0611587074 -0.4362754129
C 0 3.5606548525 -0.2293651311 1.1396594408
C 0 3.9700976471 -0.3609231794 2.4987956613
C 0 3.0652982118 -0.3606327627 3.5493893778
C 0 1.6712676502 -0.223669622 3.2940886473
C 0 -1.0344830762 0.1089492795 2.4649104251
C 0 -0.700781603 0.0267139525 3.8569961676
C 0 0.6168589381 -0.1700625612 4.2478984558
H 0 5.0311614854 -0.4659093499 2.7146345407

P	0	-2.103376177	0.1016612981	5.108045215
N	0	-2.9178138236	1.4583002669	4.4536777467
P	0	-4.3334453994	1.7036103492	3.8511560666
N	0	-4.1900519761	2.343795686	2.3021028154
C	0	-3.0139950072	3.0373863187	1.7688513742
C	0	-3.2190278639	2.9077617837	0.257010771
C	0	-4.7385486384	3.1155003934	0.1126751667
C	0	-5.333557647	2.4597107469	1.3825589837
N	0	-1.3133971278	0.6252522338	6.4907484417
P	0	-0.6427512594	1.929518105	7.0385159625
N	0	0.7643885724	1.4933256828	7.8514681611
C	0	1.825337407	2.3745903145	8.3336576214
C	0	3.1170703008	1.6272833353	7.9344419721
C	0	2.7071747676	0.1263413791	7.9127857691
C	0	1.1749917291	0.1214562968	8.1534412979
N	0	-0.128935563	3.2373594399	6.1032163316
C	0	1.0484016639	3.1142761699	5.2126876796
C	0	0.5292772296	3.5387404673	3.8370591917
C	0	-0.4691168654	4.6483189371	4.1990496368
C	0	-1.1558025244	4.1062439699	5.4647549821
N	0	-1.7197024815	2.7269656153	8.0762642014
C	0	-1.2720991504	3.8252301126	8.9417876647
C	0	-2.5685841652	4.2425195551	9.6426306802
C	0	-3.2770532719	2.8932962266	9.8968632459
C	0	-2.7135019192	1.9240615103	8.8222504363
N	0	-5.2954317062	2.831063717	4.6739627212
C	0	-5.5651025659	2.5648959655	6.1078576588
C	0	-5.0438978352	3.8107730473	6.8326571084
C	0	-5.3454600335	4.9254442962	5.8205252962
C	0	-4.9843300586	4.2698083894	4.477736126
N	0	-5.3808688024	0.3973030676	3.7290569082
C	0	-4.9269051536	-0.8407867181	3.0596427747
C	0	-6.1764142412	-1.7301414643	3.0680542508
C	0	-7.3188500744	-0.7136771132	2.916999597
C	0	-6.8578266519	0.4626951272	3.7914382394
H	0	4.3077631243	-0.2360879358	0.3488971812
H	0	-2.0748935997	0.244123991	2.1789029854
H	0	0.8523346652	-0.2463038724	5.3056057022
H	0	3.4178810874	-0.4644692519	4.5735015825
H	0	-2.2531811866	1.0436227844	9.2929883367
H	0	3.2294813799	-0.4569160021	8.6789491985
H	0	-3.4730483364	1.5547994699	8.1301772164
H	0	-3.1621546619	4.8688399191	8.9671001067
H	0	-4.366455764	2.9824482957	9.8350101065
H	0	-3.0394824719	2.521850499	10.9001279157
H	0	-2.3885214955	4.811114098	10.5615956719
H	0	-0.8151429896	4.6224582443	8.3485157741
H	0	-0.5271329313	3.4851709529	9.6835111893
H	0	1.7691524509	2.4792245957	9.4306568833
H	0	3.9395851137	1.8332810797	8.6283257065
H	0	3.4353758999	1.9505056433	6.9374898308

H 0 2.9441738542 -0.3249947575 6.9437270563
H 0 1.4494238069 2.0997558076 5.2175720007
H 0 0.6227435692 -0.584491157 7.5320849041
H 0 1.8438298128 3.7947804462 5.555077644
H 0 1.3305631308 3.8706622024 3.1677978579
H 0 0.0126923293 2.6932236977 3.3702200218
H 0 -1.1931209962 4.8565284714 3.4044629833
H 0 0.0672342143 5.58139708 4.4180844723
H 0 -2.0326231719 3.5094788281 5.196955775
H 0 -1.4649023262 4.905754863 6.149064995
H 0 -3.9629744688 3.7177492206 6.9834376756
H 0 -5.5189219569 3.9673231663 7.8073438388
H 0 -6.6460184068 2.4354804809 6.2640861013
H 0 -6.4121824508 5.1838231358 5.8452964023
H 0 -5.0644531316 1.6463339083 6.4320094461
H 0 -4.7720577536 5.8420272864 5.9965625392
H 0 -3.9192444616 4.4251873299 4.261647162
H 0 -5.5585847241 4.6776709864 3.6371949548
H 0 -4.0835874281 -1.2833682766 3.5970314397
H 0 -4.6036757319 -0.6318244611 2.0256972257
H 0 -6.1458167117 3.0679790023 1.8074503588
H 0 -5.7465553589 1.4639434198 1.1746050001
H 0 -5.1498344993 2.6861775968 -0.8068409271
H 0 -4.9642318482 4.1890623427 0.105848402
H 0 -2.9343731902 1.898312856 -0.0654605054
H 0 -2.6311019303 3.6314120353 -0.318012325
H 0 -2.099146775 2.5770366848 2.1427979695
H 0 -3.0037251625 4.1028226626 2.0589529627
H 0 -6.1565575387 -2.4858572698 2.2759051089
H 0 -7.3985555591 -0.3943311783 1.8692716815
H 0 -8.2950551848 -1.1037700069 3.2244399353
H 0 -6.2561978987 -2.2490455289 4.031679845
H 0 -7.2351599982 1.4275724547 3.4402583327
H 0 -7.1958553302 0.3345975093 4.8298704378
H 0 0.9560493802 -0.1149464811 9.2077786361
H 0 1.7409073342 3.3719118516 7.897408338
H 0 -0.6728935436 -0.615102637 -0.4546784407
H 0 -0.4990105835 1.1301408768 -0.3204642692
H 0 1.7695251482 0.9496282201 -0.9912830826
H 0 1.6033140694 -0.7980776956 -1.0995468179

VIbH^{+}
C 0.0536575639 0.5067377535 0.1132133964
C 0.0442510269 0.2624464107 1.6126835168
C 1.3594179436 -0.0307693546 2.0338193072
C 2.2916122742 0.0105351792 0.9742683095
C 1.558079394 0.3530247115 -0.3109556758
C 3.6185467245 -0.2390073184 1.2531176976
C 3.9980803879 -0.5346501424 2.5952945542
C 3.0816372365 -0.5792692342 3.6328658322
C 1.7072757534 -0.3193640809 3.3739720496

C	-0.9674628225	0.2862876632	2.5469666305
C	-0.6473104236	0.0145326086	3.9135929638
C	0.646655532	-0.2896824191	4.3174658776
H	5.0456033882	-0.7340694935	2.8060806749
P	-1.9551950443	0.1404650187	5.1743284183
N	-3.3481206249	0.068412413	4.3944528732
P	-4.8244159611	0.5947967108	4.6711644273
N	-5.6353734922	0.2806605257	3.2575428714
C	-5.0197732132	-0.1620981929	1.9863264017
C	-6.2338869521	-0.5483006564	1.1319871157
C	-7.3140231712	0.4433989944	1.5920708972
C	-7.0844408923	0.5299740149	3.1093354983
N	-1.7738953726	1.3979950159	6.1771923883
P	-0.5524295753	2.3399527737	6.5694905266
N	0.5499625675	1.5079747466	7.5166391095
C	1.5541518536	2.223816337	8.3401807404
C	1.5777824718	1.4444304018	9.6601986761
C	1.3096115052	0.0051574011	9.1978551476
C	0.2461625422	0.1755120133	8.1023153555
N	0.2867384776	3.0088095423	5.3064313675
C	1.7520843197	2.9565084763	5.0980823126
C	1.9181501145	3.3453746383	3.6219495665
C	0.7190878496	4.2690348646	3.3560537864
C	-0.4135746816	3.6004808762	4.1451994178
N	-1.1441708019	3.6426714132	7.4261719392
C	-0.702737454	5.0475707448	7.2698203755
C	-1.4259569111	5.7784502463	8.4127731796
C	-1.5533038135	4.7031550844	9.5028553288
C	-1.8818255778	3.4434680845	8.6915373428
N	-5.0791979281	2.2131172027	4.9782056078
C	-4.8497752512	2.833322873	6.3109520774
C	-4.2650930105	4.2161459016	5.9886255793
C	-4.851894923	4.538625758	4.6065278766
C	-4.7950513966	3.1834600329	3.8930960243
N	-5.5036009283	-0.1474711617	5.9991992398
C	-5.2832810606	-1.6104350494	6.1691457532
C	-6.3880465072	-2.0409357223	7.1459949606
C	-7.5344924471	-1.0694757093	6.8280446241
C	-6.7947846751	0.2556529046	6.6153907844
H	-1.9991184223	0.4943450624	2.2801182929
H	0.8704385918	-0.4873704895	5.3626091439
H	3.4082471978	-0.8118838427	4.6438506754
H	-1.5619629116	2.5215678336	9.1900800929
H	0.9672141308	-0.6546526371	10.0005243529
H	-2.9595283928	3.3638453437	8.4997524893
H	-2.4226767089	6.0946215139	8.0818369318
H	-2.318142373	4.9326387735	10.2508706061
H	-0.5963105294	4.576263008	10.024927372
H	-0.8820749686	6.6692927177	8.7404235002
H	-0.9804935562	5.4459867668	6.2886131258
H	0.3874967771	5.1387355069	7.3664177749

H	1.2731497061	3.2729350229	8.474102582
H	0.7684460045	1.7907183346	10.3156714941
H	2.5229585591	1.5589315114	10.1992552644
H	2.2236577018	-0.4280055588	8.7731091257
H	2.1424140709	1.9584167263	5.3135226266
H	0.3063823658	-0.6164767007	7.349246069
H	2.2587080926	3.676122457	5.7562720928
H	2.8830165254	3.8240615119	3.4303591084
H	1.8524707277	2.4511256662	2.9942160718
H	0.4779549978	4.3682107554	2.2932345396
H	0.9140177494	5.272193552	3.7553630368
H	-0.9003935809	2.8162160084	3.5516860706
H	-1.1856495762	4.3106399002	4.4617275816
H	-3.1745219682	4.1444432663	5.9354992496
H	-4.5205602865	4.9606175237	6.7497271329
H	-5.8074531874	2.9225769516	6.8384886967
H	-5.8931722553	4.8708305605	4.6994095624
H	-4.1706799225	2.2254070732	6.9129275101
H	-4.2970792162	5.3128466942	4.0670273757
H	-3.794871081	3.0148036984	3.4649257446
H	-5.530539889	3.0879835491	3.0879804366
H	-4.2772164561	-1.8069331732	6.5527223659
H	-5.3857808079	-2.1323383643	5.2074700129
H	-7.363776801	1.5066698556	3.5225779126
H	-7.6624145533	-0.2389205649	3.6418264216
H	-8.3314794327	0.1277907492	1.3431138665
H	-7.1434957365	1.4247544829	1.131647385
H	-6.5411663171	-1.5762150658	1.3617763216
H	-6.0227135084	-0.4899877401	0.0598546965
H	-4.3264878998	-0.9882131337	2.1577073922
H	-4.4583283302	0.6620143213	1.5209640233
H	-6.6597061007	-3.0932218455	7.0206212408
H	-8.0423179117	-1.3713136562	5.902887001
H	-8.284827625	-1.0073360232	7.6222553692
H	-6.0537061932	-1.9001120447	8.181388792
H	-7.3375864229	0.9615984308	5.9814343416
H	-6.600827081	0.7490471467	7.5769453681
H	-0.7675386384	0.1542404884	8.5216650064
H	2.5357185609	2.1969701131	7.8484378172
H	-0.585164171	-0.2159812629	-0.4081516779
H	-0.3352038767	1.5022779616	-0.1322780062
H	1.9459820833	1.2770394274	-0.755933403
H	1.6848376058	-0.4336051073	-1.0636736304
H	-1.7938415491	-1.0347126574	5.9445102033
H	4.3773611463	-0.2167269324	0.4749605857

VIIa

C	0.0336351222	-0.0964179335	0.0127739451
C	0.030773634	-0.0645546472	1.4378685198
C	1.2863931875	-0.0217933704	2.0493851954
C	2.5255813709	-0.004333833	1.3641585175

C	2.5010821581	-0.0328443405	-0.0197261165
C	1.2381519546	-0.079464667	-0.6798033227
C	1.4980079636	0.0187707159	3.4517332025
C	2.959509102	0.0599136837	3.6351916108
C	3.5636718602	0.0481566367	2.4080393146
C	-1.0945778965	-0.0540965365	2.3199203341
C	-0.9184684701	0.0017981293	3.6952767156
C	0.3913960876	0.0272438306	4.2762263088
P	-2.4382782245	0.1593624704	4.7739139838
N	-2.2656184586	1.7597865943	5.3291574288
P	-2.1188192989	3.2149857735	4.8158455201
N	-3.2170261424	3.6247502735	3.5779237894
C	-4.5151472339	2.9515477217	3.5542699394
H	1.2223396151	-0.1030342001	-1.7668322887
N	-1.8826192898	-0.7557227994	6.0913953301
P	-2.5256617772	-0.9297252742	7.5030405712
N	-1.985372307	-2.4090564725	8.1257665842
C	-0.7744633459	-3.0325818667	7.5962381157
N	-2.1894179931	0.3807715179	8.539297966
C	-0.9169583387	1.0815991124	8.3533739845
N	-4.2136889611	-1.0606645331	7.710051731
C	-4.8499080003	-2.2478773631	7.1251510884
C	-2.6686071524	0.4229880953	9.9189807624
N	-2.2576052584	4.2317702451	6.1742362984
C	-3.0595209606	3.7915468927	7.3122023369
N	-0.6656626346	3.7837482474	4.1415148079
C	0.5127851316	3.799327615	5.015105477
C	-2.1819964103	5.6823142728	6.0407071575
C	-0.3097840326	3.4217307045	2.762419728
C	-3.2596740786	4.9594252556	2.9839188706
C	-5.0269381556	0.1449179619	7.5011017878
C	-2.3246347672	-2.8267470516	9.4804998296
H	0.4726591248	0.049081197	5.3603838526
H	3.4143929095	-0.0202251571	-0.610689766
H	-0.907544761	-0.1323236888	-0.5317680527
H	-2.1036735379	-0.0775628142	1.9091250469
H	-4.8793704017	2.9117017952	2.5190627578
H	-4.4231913035	1.9288988813	3.9231160918
H	-5.2680885844	3.4849113781	4.1596889135
H	-5.9946345254	0.0094903798	8.0014476806
H	-5.208441573	0.3367934877	6.4326719324
H	-4.5317884129	1.0184414851	7.9248961135
H	-4.9948299862	-2.141846068	6.0387907635
H	-5.8307268096	-2.3913429261	7.5960134418
H	-4.2393850816	-3.133471979	7.3100614408
H	-3.9696200661	5.6222733048	3.5053644378
H	-3.5798058841	4.8808804492	1.9357660698
H	-2.2717122162	5.4220303891	3.0046236117
H	-0.6133340369	-2.7030011516	6.5696163017
H	-1.2053283093	3.2982893177	2.1531076782
H	0.1131955549	-2.7782751995	8.1992916715

H -0.895330174 -4.1246185814 7.6096114995
H 0.3042697734 4.2238423297 2.3313147831
H -1.5693262677 -2.5114127979 10.2192558868
H -3.2952089254 -2.4215355785 9.7739164247
H -2.3907776591 -3.9227570441 9.515063965
H -0.7000594548 1.17370092 7.2903289677
H 1.0019662917 2.814458671 5.0535151165
H -0.0830303327 0.5710996321 8.8648056498
H 1.2350086474 4.5277494996 4.6242765885
H -1.0066484821 2.0920665121 8.7712215767
H 0.2313622748 4.0930445213 6.0280012086
H -1.9575083977 -0.037509272 10.6242304347
H -2.8084609061 1.4705567968 10.2194067621
H -3.6290930888 -0.0878280177 10.0072297234
H -3.0177298917 2.7052403046 7.3886637527
H -1.7820205546 6.1110428293 6.969887751
H -2.6506358053 4.2314321872 8.2326861025
H -4.1133091974 4.1084565595 7.2243165611
H -3.1683973587 6.1400644799 5.8566996841
H -1.5104848149 5.9555862061 5.2245266817
H 0.2634784765 2.487734345 2.7243915119
H 3.4657358612 0.0941232712 4.5939033308
H 4.6322220392 0.0720065577 2.2233091955

VIIaH^+
C 0.0308962325 0.0706774524 0.0403877785
C 0.0690261179 0.1120260125 1.4614788671
C 1.3383714281 0.0322714198 2.0404277666
C 2.5568823571 -0.0688512482 1.3212288696
C 2.4980938396 -0.095746568 -0.0582418673
C 1.2147866349 -0.0329337311 -0.6871587534
C 1.5953325949 0.0410206357 3.4330972317
C 3.0590978191 -0.0605598225 3.5760546914
C 3.6251893273 -0.1241299482 2.3349030728
C -1.0333098335 0.2093342091 2.3665117261
C -0.8000204172 0.21765492 3.7317018365
C 0.5144037665 0.1341693892 4.2884768854
H -1.6441019993 0.2933382313 4.411581756
P 1.083311292 -0.1576037021 -2.4914314697
N 0.3092975024 -1.4860400322 -2.961258717
P 0.1905749919 -2.9832807569 -2.4377986415
N -1.4065066051 -3.2381170922 -1.9953622896
C -2.487760655 -2.3600533418 -2.4506715125
N 2.5574312866 0.1238377079 -3.0753437721
P 3.0435365685 0.377367219 -4.5715042685
N 4.4164682223 1.3175420781 -4.4642945255
C 5.0879348316 1.6280854096 -3.1997783978
N 3.2709188069 -1.0955514331 -5.3585152475
C 3.8613336115 -2.1934278501 -4.5826689021
N 2.0435506675 1.2034528815 -5.6366730271
C 1.9118139157 2.6626871206 -5.5214997688

C 3.6098598008 -1.172145733 -6.7851843776
N 0.7588629314 -3.9859546648 -3.6688212098
C 0.6226383286 -3.5528008392 -5.0640065537
N 1.0514814826 -3.5203518455 -1.1104048798
C 2.4990502517 -3.7459732957 -1.1986778498
C 0.6973515132 -5.4445131891 -3.5126510432
C 0.5916145343 -3.3146973501 0.2704811196
C -1.8724016977 -4.5583486465 -1.5594043459
C 0.8269251628 0.5658815723 -6.1612034981
C 5.2189343588 1.5831050377 -5.6608092166
H 0.6370530377 0.1473288941 5.3685531938
H 3.3864765784 -0.1610873571 -0.6796765191
H -0.9288707362 0.1070557048 -0.4730152592
H -2.04880575 0.2790530486 1.9843431429
H -3.2028139336 -2.2150234125 -1.6311027695
H -2.0846394344 -1.3922293771 -2.7476006523
H -3.0253177164 -2.797469526 -3.3045902585
H 0.6302105289 0.9462882872 -7.1702939122
H -0.0464503976 0.7871557338 -5.5312401336
H 0.9490191569 -0.515497541 -6.2137201458
H 1.0996457992 2.94201935 -4.8343797684
H 1.6859365575 3.0766733784 -6.5106417865
H 2.8421781816 3.1036073139 -5.1620750639
H -2.3386667455 -5.1155667251 -2.3842797312
H -2.6180488219 -4.4332856344 -0.7648303124
H -1.0479144646 -5.1487555505 -1.1570339869
H 4.406945144 1.4614136181 -2.3661359175
H -0.4601528929 -3.0319657464 0.2900491729
H 5.9841618537 1.0059945801 -3.0637828615
H 5.3938243448 2.6816864267 -3.2033593872
H 0.7198237624 -4.2470099191 0.8345325999
H 6.0817946833 0.9053448583 -5.7288064102
H 4.6138781344 1.4779050298 -6.5635488577
H 5.5927276334 2.6134051311 -5.625595038
H 3.5542045159 -2.1239954923 -3.5402688884
H 3.056782634 -2.8638502459 -0.8543519691
H 4.9607260253 -2.1821531416 -4.6359254393
H 2.7688409618 -4.5955179029 -0.5600950961
H 3.5044585739 -3.1468574149 -4.986025878
H 2.7887865392 -3.9758467347 -2.2241320045
H 4.6975020465 -1.166484732 -6.9472744329
H 3.2083852034 -2.1073707595 -7.1934743348
H 3.1680344171 -0.3401219929 -7.3346553013
H 0.8507287851 -2.4913202002 -5.1445946947
H 1.4890451968 -5.8992106273 -4.1192122914
H 1.3402020672 -4.1137515159 -5.6737211868
H -0.3855525047 -3.7435197527 -5.4629232851
H -0.2674824453 -5.856244494 -3.8433095849
H 0.8642678079 -5.727290816 -2.4718894875
H 1.1722403788 -2.525349701 0.7596551056
H 3.5913671849 -0.0783531769 4.520120457

H 4.6847648853 -0.2000377384 2.1196864165
H 0.2151138051 0.8885589355 -2.8662940697

VIIb

C 0.12335331 0.6968679621 0.2135611995
C 0.0859302444 0.3498494313 1.6450087011
C 1.4029008479 -0.0484869517 1.9776611011
C 2.2489052651 0.0410846847 0.8447067129
C 1.3959607361 0.5174043536 -0.2575510245
C 3.5838123628 -0.2870612101 1.0070153893
C 4.0347910724 -0.6977078634 2.2966789995
C 3.1871010726 -0.7768134138 3.3946106299
C 1.8089777847 -0.4377859098 3.2567574832
C -0.864772812 0.3671401211 2.6477877334
C -0.4977107026 -0.0124312841 3.9796984137
C 0.7994041465 -0.4236123782 4.2652619731
H 5.0838529119 -0.9563276557 2.4200717772
P -1.7543527675 0.0204128216 5.3896960667
N -3.1300825591 0.2338440029 4.4345284507
P -4.5721102493 0.7399997501 4.747786847
N -5.4670095644 0.4512926575 3.3573776558
C -4.8910121469 0.0437051193 2.0650213874
C -6.1283423117 -0.3385406685 1.2422405887
C -7.1924965627 0.6504828116 1.7458368695
C -6.9056737018 0.734894496 3.25575863
N -1.490026629 1.5617714261 6.1051669372
P -0.2327934265 2.4567421973 6.3371080274
N 1.0248071382 1.6604886023 7.1536298666
C 2.128020472 2.4162283039 7.779228129
C 2.2035484532 1.8675186121 9.2090742577
C 1.8432648656 0.3890870145 9.001928413
C 0.7282023255 0.4373058826 7.9430542752
N 0.5177155341 3.1653333773 5.0132327647
C 1.9319687415 3.0235754417 4.6114138111
C 1.9292156812 3.3540490093 3.109609571
C 0.7152859273 4.2813719525 2.9428444173
C -0.312652931 3.6739072301 3.9045973235
N -0.6740433452 3.7983616435 7.2621693687
C -0.2874972336 5.1980127948 7.0217090071
C -0.9863965782 5.9658827814 8.1572875024
C -1.0475144011 4.9370797941 9.2974033248
C -1.3628105146 3.6336935255 8.5509934859
N -4.8755639105 2.3738329432 5.0656858387
C -4.5288194348 2.9734112147 6.3814522541
C -3.8959485274 4.331009311 6.0381569058
C -4.509425309 4.6799507398 4.6745205134
C -4.5478314944 3.3179929785 3.9738074587
N -5.3033291837 0.0165999721 6.0774140715
C -5.1171791366 -1.44579266 6.2446813544

C	-6.1773406976	-1.8339118118	7.2859212817
C	-7.3148444544	-0.8387144546	7.0078802628
C	-6.5515345542	0.4631841573	6.7316667517
H	4.29247271	-0.2359582833	0.1830817787
H	-1.8971134925	0.6605633193	2.4752574713
H	1.0603936484	-0.7288007743	5.2762572785
H	3.5748655409	-1.0918347456	4.3613080387
H	-0.9992991289	2.7466240231	9.0819424792
H	1.5182371222	-0.1155008205	9.9177347295
H	-2.4451552284	3.5091710372	8.406651593
H	-2.003303568	6.2395879976	7.8497873256
H	-1.7939373714	5.1786575733	10.0616589683
H	-0.0689125881	4.8606867884	9.7898598108
H	-0.4555226094	6.8855147578	8.4251118638
H	-0.6136927167	5.5469598393	6.03546534
H	0.803698865	5.3330967561	7.0669880697
H	1.9335711238	3.492491404	7.7489102508
H	1.4487292013	2.3591539732	9.8370245222
H	3.1844700571	2.0182922839	9.6731707379
H	2.7145102907	-0.1533217132	8.6120449049
H	2.3011924722	2.0133872037	4.8097554192
H	0.7228250238	-0.4545625704	7.3091521882
H	2.5602451322	3.7331415709	5.1707958798
H	2.8705475366	3.8110593538	2.7865942228
H	1.781614002	2.4392630564	2.529317442
H	0.345419686	4.3214636884	1.9129967249
H	0.9663655591	5.3032287425	3.2575512238
H	-0.8713399791	2.857659828	3.4293421713
H	-1.0437851487	4.4106202695	4.2592865844
H	-2.8135610444	4.2057159539	5.9479510293
H	-4.0909110033	5.0877871719	6.8064693903
H	-5.4450712939	3.1018524441	6.9738552664
H	-5.5283243373	5.0703688715	4.7973027784
H	-3.8344845657	2.3352334708	6.9344522345
H	-3.9279448459	5.4189764414	4.1119418899
H	-3.5663812384	3.0884325651	3.5303959822
H	-5.3013370007	3.2635373167	3.1795705929
H	-4.0948177102	-1.6708955358	6.5627151862
H	-5.2966033571	-1.971793689	5.2943962372
H	-7.1512554733	1.7204134085	3.6730165111
H	-7.4910682795	-0.0145932707	3.8113568552
H	-8.2198655212	0.33759972	1.531165442
H	-7.0361180584	1.6329474593	1.2815025762
H	-6.428881393	-1.3682661172	1.4759038985
H	-5.9520258853	-0.2727295235	0.1632968177
H	-4.1812918317	-0.7756845064	2.1990611907
H	-4.3500760933	0.878770606	1.5902561781
H	-6.4812970723	-2.8822082386	7.1977691221
H	-7.8725132235	-1.1448882495	6.1125257023
H	-8.0261354142	-0.7431518143	7.8357074908
H	-5.7854303761	-1.6797739001	8.2997607471

H -7.1052156163 1.1678596947 6.1047909378
H -6.3165092612 0.9754725468 7.6763479874
H -0.2644471124 0.4984843605 8.4052104086
H 3.0711203293 2.2329666471 7.2427686441
H -0.7266873437 1.0412187667 -0.3658819069
H 1.7310351107 0.6939237488 -1.2739546626

VIIbH^+
C 0.0993644876 0.5925717064 0.1301004401
C 0.0735792156 0.3236915815 1.5788558049
C 1.4014505244 -0.0223684022 1.9353932603
C 2.2413358848 0.0263512144 0.7958982827
C 1.374363626 0.4202411836 -0.3292488717
C 3.580385725 -0.2689588878 0.9680433416
C 4.0480329258 -0.6091681615 2.2753069943
C 3.2108271179 -0.6534469331 3.3785969331
C 1.8237489172 -0.3471853021 3.2265129911
C -0.8812018392 0.3538094972 2.5750682196
C -0.4844738264 0.0463787865 3.9160264246
C 0.823490365 -0.3039120224 4.2373687238
H 5.1014969418 -0.8435968262 2.4014676991
P -1.7106158922 0.2042051824 5.2543871101
N -3.1462888422 0.1385775331 4.5574387172
P -4.6026083282 0.6723595071 4.9188522247
N -5.4956244727 0.3573753347 3.5566258892
C -4.9583730892 -0.0935575515 2.2527795185
C -6.2226606162 -0.4773082428 1.47345737
C -7.2696478119 0.5204662977 1.993133912
C -6.9504643504 0.6109855833 3.4938487062
N -1.4512283688 1.4731358163 6.224189026
P -0.1991499248 2.4187336922 6.4957621366
N 0.9756679422 1.5974589278 7.3627522708
C 2.0485505568 2.325939585 8.0831880553
C 2.1694395627 1.5865302399 9.4207341536
C 1.8513821842 0.1374628951 9.0258429611
C 0.705014132 0.2883987432 8.0145101482
N 0.5337048858 3.056831954 5.1540239126
C 1.9781840823 3.0080570984 4.8299775548
C 2.0219934813 3.3737489087 3.3394113748
C 0.8067232627 4.2969286268 3.1601353303
C -0.2582558412 3.6439226014 4.0502943869
N -0.7182166694 3.7383030672 7.3730290542
C -0.2847838685 5.1387685269 7.1618471773
C -0.9190745503 5.8908544482 8.3432697708
C -0.9701356487 4.8334904685 9.4568096216
C -1.361800629 3.5626354613 8.692460709
N -4.829215123 2.2925966265 5.2355740316
C -4.5125791106 2.9156344084 6.5492544513
C -3.9458342877 4.2957802481 6.1857121521
C -4.618347421 4.6159326976 4.8426853454
C -4.6100163962 3.2585582688 4.1314627082

N	-5.2035420578	-0.0639819013	6.2868888279
C	-4.9873496406	-1.5294888473	6.4416083218
C	-6.0358134723	-1.951639488	7.4821930652
C	-7.1893374594	-0.9676620384	7.2364552284
C	-6.4502277475	0.3504260611	6.982395885
H	4.2834355686	-0.2494059647	0.1390399765
H	-1.9216478277	0.597117193	2.381121308
H	1.0995180687	-0.5287781355	5.2643251785
H	3.6068839665	-0.9199641639	4.3557119659
H	-1.0094608263	2.647615092	9.1815335604
H	1.5681388345	-0.4940122565	9.873097216
H	-2.4508291169	3.4845519128	8.5808143472
H	-1.9361021107	6.2069999016	8.0814350203
H	-1.6771986816	5.0784809359	10.2550418497
H	0.0221149338	4.7101768429	9.9092655646
H	-0.3490396706	6.7838767101	8.6158078048
H	-0.6334194233	5.5230672398	6.1978542926
H	0.8098807827	5.2269446299	7.1759289668
H	1.7889549768	3.3817539759	8.2068725437
H	1.4180332817	1.9620732194	10.1270772915
H	3.1554512254	1.7058723337	9.8795829154
H	2.7241849123	-0.3191071132	8.5427969906
H	2.3898280541	2.0153237867	5.0301647167
H	0.6946938668	-0.5276019688	7.2851716201
H	2.5317595979	3.7407102782	5.4338099772
H	2.9680559835	3.8474492713	3.0612787247
H	1.9019579306	2.4712491409	2.731892558
H	0.4793512014	4.3793427672	2.1194731882
H	1.0355964581	5.3053544414	3.5269852346
H	-0.7990264832	2.8577570272	3.5084091719
H	-0.9970125395	4.3617776967	4.4235434963
H	-2.8610745801	4.2205614997	6.0630470135
H	-4.1501772164	5.0433169228	6.9591295613
H	-5.4340000858	3.0088616465	7.1371195793
H	-5.6508395613	4.9510947585	5.0002201824
H	-3.7976214321	2.3075917893	7.1084092349
H	-4.096867942	5.3869715579	4.2666667841
H	-3.6386959189	3.0857869271	3.6426979677
H	-5.3941678095	3.1627211209	3.3738269831
H	-3.9620931043	-1.7361479382	6.7642378669
H	-5.1519763712	-2.0483861516	5.486989434
H	-7.2020521491	1.5897364145	3.9198865217
H	-7.4980817839	-0.1545458668	4.062037157
H	-8.3012661216	0.2082130555	1.8059882297
H	-7.1228988616	1.4996105352	1.5200106584
H	-6.5197813107	-1.5031600988	1.7246805846
H	-6.0749412525	-0.4235989964	0.3905824862
H	-4.2600386445	-0.9224076769	2.3859441173
H	-4.4221214376	0.7262578285	1.7515240003
H	-6.3253476195	-3.0006991563	7.370692881
H	-7.7553662718	-1.2615759666	6.3430963527

H -7.8894285343 -0.9002321887 8.0748543595
H -5.638291387 -1.8175737674 8.4959017466
H -7.0229996724 1.0642258932 6.384743868
H -6.1920644893 0.8385089647 7.9314798772
H -0.2715246601 0.2934990271 8.5145266233
H 2.9874742823 2.2729095487 7.5162961669
H -0.759650139 0.8796263381 -0.4655052619
H 1.7049751522 0.5476008313 -1.3536954319
H -1.5118065687 -0.9622874757 6.0288685838

VIIIa
C -0.0798803571 -0.5557146402 -0.0395981404
C -0.0220429792 -0.8846011003 1.4599295387
C 1.4268916085 -0.854516238 1.9708060324
C 2.3205291452 -1.8025390667 1.1542028799
C 2.2819509568 -1.5114160516 -0.3583728293
C 0.8231813166 -1.4936288536 -0.8612209644
H 1.4616305172 -1.1297439911 3.034145145
P 3.2113204207 0.0698038843 -0.8653155524
N 3.4300242894 -0.2957108589 -2.5327658917
P 2.7796974677 0.3540963553 -3.7911450044
N 4.0003072688 0.8539217214 -4.8726756681
C 5.2925939095 0.1745907821 -4.8241897741
N 4.6981670419 -0.3409154191 -0.1452920635
P 6.1340681956 0.2237780672 -0.3457193838
N 6.9060163511 -0.4499278003 -1.7090978226
C 6.5765077277 -1.8373803458 -2.0428174468
N 6.4344166047 1.8852142396 -0.6058968702
C 6.1095201581 2.7825017672 0.5099278901
N 7.0105677993 -0.0487975835 1.0824017962
C 6.5773169349 -1.0772133878 2.0241635452
C 6.0216631497 2.4609044437 -1.8915384905
N 1.692926973 -0.7333161917 -4.5255310526
C 0.9167485049 -0.4061433094 -5.7168787947
N 1.8196091544 1.7578393257 -3.7258655358
C 2.4748138899 3.0341543714 -3.4270418221
C 1.8901992584 -2.1695520598 -4.3425423019
C 0.4892936117 1.6753023978 -3.1137561894
C 3.6570427726 1.3408928625 -6.2053833456
C 8.4389773245 0.2377271484 1.141283629
C 8.2485871968 -0.0631007487 -2.1328719046
H 0.7860357721 -1.2195546442 -1.9223141959
H 0.2468410991 0.4828670288 -0.1841907554
H -1.1144702607 -0.6173740279 -0.4060489906
H -0.6466055871 -0.1820403086 2.028969618
H -0.442555046 -1.8891535473 1.6275492411
H 3.3550981227 -1.7591592991 1.5097628512
H 1.9711838202 -2.8359717901 1.3172947806
H 2.8348161513 -2.3058329545 -0.8816256121
H 0.4176372817 -2.5164262774 -0.7937192435
H 2.597463064 3.1813274741 -2.34302035

H 3.4561184055 3.0762111556 -3.9020972018
H 1.8573061684 3.8522669869 -3.8214358262
H 6.5789005569 3.3923045538 -2.0582657266
H 4.9468500948 2.688863622 -1.9096270409
H 6.2351669994 1.7738724308 -2.709002052
H 5.0271911445 2.9750452758 0.5801773746
H 6.6242800739 3.7392918872 0.3547390135
H 6.4481618778 2.350773443 1.4532822733
H -0.1550444209 2.4491121665 -3.5528454177
H 0.535493889 1.8333485 -2.0271161096
H 0.0397623286 0.6994365025 -3.3014624892
H 5.4987481658 -1.2092584735 1.9480944426
H 2.4579778571 -2.3479362016 -3.4291368374
H 7.0695547723 -2.0455380913 1.8321056681
H 6.8307834075 -0.7612416371 3.0456048821
H 0.9135808863 -2.6659131993 -4.2575255422
H 9.0547862099 -0.6250901575 0.8366191026
H 8.6847167959 1.0887158405 0.5026513477
H 8.7129367865 0.4978024909 2.1727448344
H 5.5182142202 -2.0187876703 -1.8574547065
H 1.4076921012 -0.7499907977 -6.6417935849
H 7.1819668537 -2.5570764548 -1.4658851189
H -0.0651223145 -0.8967295759 -5.6566130544
H 6.7767616018 -2.0047787163 -3.1090959387
H 0.7538654569 0.6706277342 -5.7862067404
H 9.0284241043 -0.7037129884 -1.6897297843
H 8.3216010451 -0.1530507728 -3.2260676999
H 8.4529662394 0.973919868 -1.8615708798
H 5.5114900149 -0.1188021146 -3.7988791179
H 3.5492496645 0.5243336083 -6.9388179675
H 5.3152049959 -0.7212444907 -5.4687637464
H 6.0737851364 0.8633416416 -5.1751430894
H 4.4545086422 2.0073402727 -6.560273904
H 2.7268529926 1.9119979886 -6.1759229374
H 2.4302215188 -2.6175383061 -5.1936238931
H 1.8160865192 0.1709706363 1.8957980476

VIIaH^+
C 0.1929407089 0.3529302899 0.2141658172
C 0.2844020989 0.1404289117 1.7329590202
C 1.7036324235 -0.272210336 2.153351895
C 2.1621878285 -1.528663532 1.397198892
C 2.0773958378 -1.3646810189 -0.1356929495
C 0.6698698285 -0.8858531102 -0.5656598623
H 1.7407758279 -0.4604467852 3.2331218309
P 3.4137078306 -0.326623447 -0.8588699795
N 3.4420748984 -0.5101314149 -2.4583338392
P 2.8512219909 0.1904883661 -3.7473733121
N 4.1027829094 0.2976742468 -4.8669439574
C 5.2164930718 -0.6557935232 -4.8184096076
N 4.7709537228 -0.7192722356 -0.0941811112

P	6.3110783181	-0.4309268413	-0.3584764513
N	6.8497560963	-1.2956822937	-1.7029192262
C	6.3742356015	-2.6780949205	-1.8552326518
N	6.8112260311	1.133841874	-0.7186053363
C	6.8918961932	2.1283966627	0.3603252352
N	7.1111558898	-0.8012707424	1.0581924032
C	6.4854858994	-1.5265794234	2.1670498108
C	6.407005014	1.7313973088	-1.9977043125
N	1.5032928455	-0.6719377368	-4.2695933016
C	0.7385611276	-0.2565682891	-5.4523921231
N	2.2737308239	1.7618851677	-3.6784501929
C	3.186669262	2.9097210722	-3.6579383024
C	1.4099263524	-2.1142132497	-4.0122766657
C	0.9601016851	2.0728311233	-3.1011285026
C	3.8249607283	0.791702764	-6.2210472529
C	8.5766263019	-0.8168596912	1.0767690455
C	8.1913372239	-1.099799703	-2.2670283263
H	0.6326512343	-0.7044781265	-1.6460585603
H	0.7974410944	1.2290400935	-0.0599799509
H	-0.8378130252	0.5900043089	-0.0776703112
H	-0.0219192519	1.0517366043	2.260746361
H	-0.4218090519	-0.6486359102	2.0305560399
H	3.1772407351	-1.8152448752	1.6878503788
H	1.5051585638	-2.366486532	1.6716676931
H	2.2797019889	-2.3393818765	-0.6047755769
H	-0.0271444356	-1.712896277	-0.3710805337
H	3.3404467128	3.2744757726	-2.6318795828
H	4.1521783733	2.6401670492	-4.0858270072
H	2.7543217235	3.7262275917	-4.2489449438
H	7.1703777227	2.4506165948	-2.3170045143
H	5.4507594838	2.2651937429	-1.9017425044
H	6.2982381855	0.9686927472	-2.7677994234
H	5.9184353483	2.6076196731	0.5449661393
H	7.6097469985	2.9035631409	0.0699968011
H	7.233544225	1.6639610561	1.2855248804
H	0.479601744	2.8585198258	-3.6966474111
H	1.058896338	2.4353069923	-2.068964595
H	0.317306719	1.1929992858	-3.1016849626
H	5.4027495891	-1.4274346713	2.1114622146
H	1.9768805455	-2.3756525793	-3.1194724854
H	6.7502412962	-2.593500364	2.1463099728
H	6.8341909584	-1.0999765112	3.1154587411
H	0.3584522904	-2.3816664915	-3.8542467783
H	8.9746029758	-1.8286823185	0.9130089554
H	8.9803669433	-0.1496703128	0.3124807449
H	8.9287547411	-0.4629203596	2.0528457231
H	5.3367939795	-2.761451203	-1.5325926077
H	1.0940400152	-0.7580797666	-6.3636576181
H	6.9898856595	-3.3869714555	-1.2811228236
H	-0.3154867678	-0.5204165164	-5.3054305837
H	6.430008186	-2.9566543939	-2.9135537596

H 0.8021965026 0.8226994341 -5.5973565216
H 8.9276201817 -1.781913461 -1.8179733247
H 8.1531924018 -1.3001084279 -3.344733891
H 8.5270534509 -0.0729939827 -2.1190187331
H 5.4179773435 -0.9393514335 -3.7865887566
H 3.5263605169 -0.0190986736 -6.9013883174
H 5.0062912533 -1.559856689 -5.4101947114
H 6.1090349911 -0.1743415705 -5.2365718596
H 4.7321900938 1.2581244705 -6.6219596168
H 3.0362929938 1.54605749 -6.2034661806
H 1.7926172561 -2.700386298 -4.8608421007
H 2.401848306 0.5564323179 1.9605649057
H 3.0826768163 1.0199036509 -0.5676474907

VIIIb

C -0.1680487647 0.0292389773 -0.0656539596
N -0.0788368349 -0.0356344169 1.40555486
C 1.3490060776 -0.0041292722 1.7388493033
C 1.955768475 -0.9643241446 0.7060457893
C 1.0793876296 -0.7527448702 -0.5571630198
P -1.238915531 0.8000805021 2.3289739406
N -2.6058317614 0.9334233069 1.5993159843
P -3.7408654375 -0.1006431131 0.8974508424
N -3.3552044065 -1.6627910965 1.495639203
P -3.5308608611 -3.1314526128 1.0134266428
N -2.1591069807 -4.0949345999 1.2173005814
C -0.8573698645 -3.6219271672 0.6945095564
C 0.1922309976 -4.3019839618 1.5958879443
C -0.5539022807 -5.5118349909 2.1815856201
C -1.9675955187 -4.9593788914 2.3932863011
N -1.0586642054 0.0074483846 3.8190969185
C -0.9518901137 -1.4760380283 3.8563216996
C -1.6461849018 -1.8840406196 5.1661140811
C -2.7127065764 -0.7950472144 5.3404842237
C -1.9589701685 0.4663920868 4.9061599548
N -0.7238931641 2.3529106884 2.7082016942
C 0.2827230664 2.697907859 3.7269881353
C 0.5933029326 4.1738030023 3.437169356
C -0.7526857375 4.7069920186 2.9216056756
C -1.2652447713 3.5558097395 2.044163181
C -5.2083093667 0.2800328524 2.0589921124
C -6.4654637652 -0.5207728384 1.6596446964
C -7.1619082906 0.0423023954 0.4075991867
C -7.4834518575 1.5364797716 0.5635256296
C -6.2179831709 2.3374788657 0.9080162262
C -5.5359192104 1.7810415026 2.1691358913
H -8.0797206009 -0.5275703664 0.20354987
N -4.7311925168 -3.9254308571 1.8895728797
C -5.1321926273 -3.5606155684 3.2535945003
C -6.5206671947 -4.196124751 3.3736009832
C -6.3469116146 -5.5202102992 2.6086170221

C	-5.4012992117	-5.1563814155	1.4418517347
N	-3.8930922607	-3.3965420683	-0.6086540611
C	-3.5310871236	-4.6056155703	-1.3790829587
C	-4.6344330627	-4.7135133675	-2.4431394809
C	-5.0391677129	-3.2493175966	-2.6738871011
C	-4.9972327797	-2.6587031006	-1.2591297224
H	-4.6177071604	2.3386438973	2.3874815406
H	-4.8543196172	-0.0719637664	3.0388714318
H	-6.2136638332	-1.577082641	1.5017410181
H	-7.1814533905	-0.4959492804	2.4981827971
H	-6.5067885724	-0.0897592167	-0.4639486318
H	-7.9473710014	1.9244573006	-0.3539659905
H	-8.2225255122	1.6655192358	1.3706576735
H	-5.5172292709	2.2887584943	0.0625365162
H	-6.4669878977	3.3979967088	1.0531959577
H	-6.214277893	1.9305882476	3.0263835938
H	-0.1324085097	1.0708495388	-0.4236416688
H	-0.6661521126	5.6484166649	2.3687183733
H	-1.1129386179	-0.4014132247	-0.4047140261
H	1.862225908	-1.9935637711	1.0677349794
H	0.7969944301	-1.7100481214	-1.0071006483
H	1.6120591028	-0.1802708827	-1.324026751
H	3.0185119813	-0.7667135674	0.5277275117
H	1.5161764559	-0.3107696665	2.7753661556
H	1.7683642448	1.010813386	1.6105482053
H	1.1692215757	2.0600361956	3.6610116624
H	1.3534020571	4.2496245531	2.6482445471
H	0.9671102804	4.7031579188	4.3204275731
H	-1.4374589121	4.870725506	3.7642554592
H	-2.6274226663	1.2578887755	4.5518236943
H	-2.3551449805	3.5036827331	1.9831031717
H	-1.3637146521	0.8696083086	5.7388776181
H	-3.1076590708	-0.7249695731	6.3602555857
H	-3.5450129743	-0.9802679884	4.6520877374
H	-2.0674149389	-2.8937778386	5.1070228153
H	-0.9393748018	-1.8620284014	6.0064240471
H	-1.4695962735	-1.9156819461	2.999926278
H	0.1003417854	-1.7826350828	3.8316938189
H	0.4834738749	-3.6209865115	2.4034705667
H	1.0994315968	-4.5764682514	1.0475207628
H	-0.7433104046	-3.9252187064	-0.3557988628
H	-0.5816088315	-6.3337519633	1.4547104567
H	-0.788461404	-2.5315881833	0.7478530334
H	-0.1078181295	-5.8891858938	3.108197958
H	-2.019110894	-4.3950303651	3.3380076852
H	-2.7302987058	-5.7437388082	2.4319535752
H	-4.7979250559	-1.5840883336	-1.2470368125
H	-5.9523506979	-2.8313771453	-0.7360442103
H	-4.6894981827	-5.9699971715	1.2373648761
H	-5.9533880521	-4.9681382863	0.5113582081
H	-7.2918554307	-5.9485752752	2.2582621363

H -5.8672970317 -6.2621360499 3.2596696943
H -7.2589573101 -3.5565388678 2.8741685909
H -6.8358883822 -4.3392839177 4.4126905008
H -5.1280278462 -2.4756196535 3.3695035832
H -4.4451252113 -3.9839039984 4.0075638287
H -6.0213512046 -3.1364711068 -3.1446561714
H -5.4872874603 -5.2778170428 -2.0430443886
H -4.290626801 -5.2216321598 -3.350595584
H -4.2980499481 -2.7478035223 -3.3094514068
H -3.4703733396 -5.4937276172 -0.7432016103
H -2.5458923092 -4.4729293118 -1.8499566083
H -0.8801184365 3.6452624683 1.017180811
H -0.126909827 2.58260424 4.7416452084

VIIbH^+
C -0.0467005169 0.2166895092 -0.0221518213
N -0.0080395133 -0.097333996 1.4321532999
C 1.390177626 -0.0330004381 1.9057612877
C 2.1664131559 -0.5397320652 0.6896676759
C 1.4318276212 0.1456437938 -0.476375345
P -1.3166301116 0.2099441562 2.4382559631
N -2.689419738 0.0342695407 1.6700099781
P -3.3870511846 -0.3074436336 0.267533715
N -3.0910135954 -1.7161404343 -0.4593340985
P -3.3368049965 -3.2379688533 -0.0623140778
N -1.9646527895 -3.8845616766 0.6333614156
C -0.6303567113 -3.5133334833 0.0897101039
C 0.3140360493 -4.590002026 0.6474564678
C -0.5933157469 -5.8197240823 0.8023784776
C -1.9010911585 -5.2030377288 1.3099051696
N -1.0313686676 -0.8074141307 3.7251067798
C -0.7753510117 -2.2491601283 3.4642604896
C -1.0872666515 -2.9303629457 4.8040869494
C -2.2052949334 -2.0556224705 5.3895148551
C -1.7584611374 -0.6377456966 5.0139918274
N -1.3341292474 1.7178100109 3.1419760759
C -0.3544117843 2.1788482543 4.1564726014
C -0.1188898133 3.6484796765 3.7921546177
C -1.4995553293 4.0849466195 3.2780360768
C -1.9694278413 2.8740339599 2.4574490151
C -5.1985620211 -0.0730730506 0.5291219808
C -6.005125795 -0.1897763467 -0.7847215711
C -5.8930223029 1.0653181853 -1.6687372495
C -6.2469512005 2.3423268613 -0.8894795924
C -5.4053996056 2.4754279823 0.3898637324
C -5.5462355776 1.2300898296 1.2798680179
H -6.5532521372 0.9564703262 -2.5378769032
N -4.5163676786 -3.631812883 1.0525670194
C -4.3945696741 -3.2746541969 2.4896812969
C -5.8487589823 -3.2183529442 2.9915214147
C -6.6051626386 -4.1354291177 2.0168696828

C -5.9331281906 -3.8337077861 0.6730532329
N -3.7423201556 -4.0204261614 -1.466467781
C -3.7090885314 -5.4930193294 -1.5974823435
C -4.3463389437 -5.7528036924 -2.9731426339
C -4.0212158751 -4.474234072 -3.761309992
C -4.2045746564 -3.3704461362 -2.7137090892
H -4.9323465023 1.3161803543 2.1823892018
H -5.4489566669 -0.9248072676 1.1755971981
H -5.7006543028 -1.0806625229 -1.3457966301
H -7.0600012404 -0.3351941224 -0.5101957745
H -4.8731012557 1.1523189627 -2.0694152701
H -6.113155196 3.2238801998 -1.5282274221
H -7.3116662585 2.3121341719 -0.6139781747
H -4.3491841893 2.6274117848 0.121574888
H -5.7097053052 3.3660095802 0.9537345688
H -6.5918156811 1.1510411266 1.6120320236
H -0.4692596847 1.2110682736 -0.2163418419
H -1.4732929719 5.0013302312 2.6810265512
H -0.6673132546 -0.5200154381 -0.540372971
H 2.0739057426 -1.6305380769 0.6224750859
H 1.5407669043 -0.3896237664 -1.4242223268
H 1.829496327 1.15620656 -0.6224035779
H 3.2303656786 -0.2881216902 0.7319555152
H 1.522404874 -0.6404166709 2.8043284787
H 1.6904296596 1.0002280088 2.1436825332
H 0.5559040548 1.5742679404 4.1432072434
H 0.6268791501 3.7237494233 2.9899518728
H 0.2355444151 4.239153429 4.642327076
H -2.1780680931 4.2530287901 4.1234985368
H -2.5925653173 0.0614005408 4.9094361805
H -3.0563605475 2.7616637989 2.4406025022
H -1.0700637406 -0.2347564568 5.7673120431
H -2.3310346071 -2.1789321835 6.4695275674
H -3.16241838 -2.288900209 4.9084310919
H -1.377712193 -3.9777556038 4.6737634882
H -0.207047807 -2.9055414081 5.4580573382
H -1.4305635732 -2.6232750963 2.6701269023
H 0.2597843657 -2.4056345007 3.1454167744
H 0.6969957283 -4.2831653536 1.6287306744
H 1.1736462262 -4.7630388694 -0.0069067423
H -0.6431675834 -3.5281672071 -1.0086222376
H -0.7535524839 -6.299155777 -0.1713335781
H -0.3571032632 -2.5085062633 0.4175749243
H -0.1922996892 -6.5725443528 1.4880213894
H -1.8550965614 -5.0559749073 2.3982818618
H -2.7840223987 -5.8093642013 1.096256795
H -3.6202154706 -2.4704463766 -2.914314076
H -5.2635351712 -3.0828132613 -2.6335751793
H -6.02695319 -4.6507648602 -0.0475611486
H -6.364584918 -2.9306578329 0.2167578008
H -7.6826348969 -3.9470104125 1.9948737752

H -6.4486718999 -5.1876840662 2.2838268796
H -6.2358419216 -2.1944097341 2.9205608266
H -5.9369068363 -3.5318775398 4.0360734442
H -3.8733853422 -2.3206818017 2.6242295512
H -3.8215173751 -4.046641216 3.0172548271
H -4.6654097996 -4.331157033 -4.6340090702
H -5.4329728324 -5.8608860046 -2.8675129798
H -3.9629385289 -6.6644941555 -3.4405548135
H -2.9796597298 -4.4934668539 -4.1057401759
H -4.2613774047 -5.984468166 -0.7864879727
H -2.6722216723 -5.853807394 -1.5629836217
H -1.6236868884 2.9553068632 1.4172354811
H -0.7938156435 2.0965578041 5.159930411
H -2.9953710914 0.6398007886 -0.6956277752

IXa
C -0.5059987996 1.2987636214 -1.3725167505
H -0.8785026552 1.918379447 -0.5458994305
C 0.8320451162 0.6409685978 -1.0043557555
C 1.4965753312 -0.1676281429 -2.1357243411
C 0.935640762 -1.5756766021 -2.3981156767
P -0.8098396451 -1.7178911864 -3.078720571
N -0.7431859733 -3.3493394611 -3.619878107
P -1.4785769643 -4.5775843894 -2.996445213
N -2.5497323485 -5.259109012 -4.1326598941
C -3.2395639716 -6.516466003 -3.863573123
N -0.5502669901 -0.7942746246 -4.485803579
P -1.5521057137 -0.4894657879 -5.641673455
N -3.2354194468 -0.3881735832 -5.384891395
C -3.9854092163 -1.6285841689 -5.154316939
N -1.4874016001 -1.6807946235 -6.859592346
C -2.2720768342 -1.5719471621 -8.086328397
N -1.2136822864 1.0629473264 -6.2359144512
C -1.9408196296 1.6273494853 -7.3660358171
C -0.2147049511 -2.3808594351 -7.050757835
N -0.3401264933 -5.7086946007 -2.433836868
C -0.7164519774 -6.973442174 -1.8132951577
N -2.4929617339 -4.4512428607 -1.634675629
C -1.8703868509 -4.1181385528 -0.348368836
C 1.0157807772 -5.7035272991 -2.9769325153
C -3.7991746996 -3.803980858 -1.7909397958
C -2.3075780459 -5.0214374093 -5.553225979
C 0.1058868143 1.6532748188 -6.0278993232
C -3.6831903379 0.6884003647 -4.4917541219
H 1.4530564751 0.4093099974 -3.0693930501
H -0.3917314928 1.9428367177 -2.2540020635
H 1.5741924483 -2.0899882773 -3.1307175243
H 1.5301046613 1.4299987688 -0.6884762335
H 1.237566714 -4.7199971298 -3.3920246272
H 1.7326844057 -5.9242950855 -2.1734798559
H 0.0161820953 -7.2279135186 -1.0343762235

H -1.6966462602 -6.8914417862 -1.340116195
H -0.8893692391 -4.5898021371 -0.268292785
H -1.749007132 -3.0310004368 -0.2273165908
H -2.5072751353 -4.4917637841 0.4646537887
H -4.4645971744 -4.1485773264 -0.988036766
H -3.7164522753 -2.7080440147 -1.733426923
H -4.2416536353 -4.0734298445 -2.751281462
H -3.486311002 -6.5977543979 -2.8029811472
H -4.1785074325 -6.5409134022 -4.432975333
H -3.8971582067 -1.972616775 -4.1142861977
H -5.0459785376 -1.4460796317 -5.373232957
H -3.2668698279 -5.0365406113 -6.089337717
H -1.6567926826 -5.7938629101 -5.997987756
H -1.8427038606 -4.0458860717 -5.685800453
H -4.7492274729 0.8782478287 -4.671444614
H -3.1251505804 1.6042655628 -4.6949407933
H -0.0015069198 2.7348453798 -5.8646571789
H 0.5709446663 1.2034957591 -5.1509879378
H 0.7637767548 1.5017116237 -6.9000548828
H -2.0104486549 2.717071758 -7.2430134866
H 0.4760807729 -1.8157509388 -7.6994722694
H -0.4121642428 -3.3496572961 -7.528157309
H -2.9557923525 1.2270303543 -7.4054409299
H -3.222418247 -1.0714034114 -7.8910103426
H -2.4886684208 -2.5797547751 -8.466830200
H -1.7390517577 -1.0204900791 -8.878306060
H -3.5458368444 0.4229887831 -3.4321396839
H -1.44167654 1.4290799139 -8.3290550097
H 1.1418032332 -6.463667416 -3.7662436
H -3.624218718 -2.4227543501 -5.8069947266
H 0.2565313239 -2.5569959854 -6.0838521278
H -0.7391417606 -7.8046323735 -2.536921589
H -2.6438086162 -7.3964736676 -4.158016327
H 0.9610412027 -2.1717169047 -1.4730409604
H 0.6933006679 -0.0103658483 -0.1278547362
H 2.5644164153 -0.277948836 -1.893592435
H -1.2706173744 0.5521132015 -1.6104143589

IXaH^+
C -0.7390124796 0.5812822381 -0.6412749557
H -1.0384857931 1.1217078695 0.2634920008
C 0.7880346928 0.4608696903 -0.7257590998
C 1.3332536779 -0.0773486098 -2.0584964986
C 0.9201600563 -1.523913616 -2.4098114609
P -0.6051632931 -1.7541759319 -3.385510438
N -0.7898420418 -3.3071314547 -3.780733661
P -1.4652642679 -4.5334507918 -3.032402973
N -2.5379046689 -5.2472558089 -4.109624276
C -3.2140740874 -6.498529923 -3.7484030776
N -0.5104795561 -0.744875625 -4.6315205018
P -1.5458581857 -0.4552228653 -5.807272543

N	-3.179502633	-0.3054487179	-5.4432622086
C	-3.9704627924	-1.4977718519	-5.110643181
N	-1.5397699933	-1.7226448342	-6.920617587
C	-2.4317493705	-1.698208463	-8.0885325084
N	-1.1332128489	1.0382380882	-6.4305304959
C	-1.8528507056	1.5799873749	-7.5875732817
C	-0.2575440155	-2.3837631742	-7.207395140
N	-0.2571253243	-5.5420146179	-2.444273494
C	-0.576992232	-6.765147298	-1.6976487142
N	-2.4104454303	-4.2646939912	-1.671401982
C	-1.7826710334	-3.9118266778	-0.391209042
C	1.0435536022	-5.6071279828	-3.1227853208
C	-3.7781745642	-3.747740957	-1.7946157807
C	-2.3701786726	-5.0691644837	-5.555916910
C	0.1907377067	1.6351782959	-6.2209487822
C	-3.6445746016	0.8934708844	-4.7313532793
H	1.0444392927	0.5918604314	-2.8787730268
H	-1.1439473888	1.1242295566	-1.5053566315
H	1.6877486147	-1.9992667998	-3.0348340089
H	1.2311936645	1.4513266308	-0.5607333018
H	1.2460038898	-4.6680266718	-3.6373131341
H	1.8268922228	-5.7789093225	-2.3752368735
H	0.2156308712	-6.9502891629	-0.963229
H	-1.5199235005	-6.6551409033	-1.159712516
H	-0.7652224203	-4.3010729267	-0.341110754
H	-1.7530166818	-2.8212320514	-0.248912791
H	-2.3651276445	-4.346825676	0.4293056775
H	-4.399103805	-4.1780651052	-1.0000908725
H	-3.8010305854	-2.652369585	-1.6921866629
H	-4.2043534118	-4.0203213383	-2.760271828
H	-3.3937380762	-6.5430645089	-2.672591656
H	-4.1847595208	-6.5380103818	-4.256002767
H	-3.9747202248	-1.6845721569	-4.026924228
H	-5.0061861892	-1.3437765796	-5.435335966
H	-3.3577191444	-5.1072116103	-6.032101677
H	-1.747063659	-5.8634462091	-5.993945635
H	-1.9139181078	-4.1015917443	-5.760630071
H	-4.6942581048	1.073574075	-4.9891188231
H	-3.0577922057	1.7647194529	-5.0236052937
H	0.0754482874	2.7165109617	-6.078816497
H	0.6563781571	1.2076721501	-5.3339654148
H	0.8453643969	1.4660103313	-7.0879578982
H	-1.9219893626	2.6696557143	-7.4879079963
H	0.3386365962	-1.8221583232	-7.9423793787
H	-0.4633290551	-3.3767566487	-7.622221077
H	-2.8675266084	1.1801119067	-7.6340648814
H	-3.3734572871	-1.2037281788	-7.846400789
H	-2.6516081503	-2.7303866396	-8.385940308
H	-1.9718755406	-1.1853502531	-8.945579950
H	-3.5707634463	0.7688328335	-3.6404843403
H	-1.335916506	1.3531084512	-8.5309052188

H 1.0765820542 -6.429038905 -3.8532324408
H -3.571697796 -2.3794650281 -5.6108603895
H 0.3187147964 -2.5074335266 -6.291542764
H -0.6441208011 -7.6410522424 -2.358443872
H -2.6323499029 -7.3816669862 -4.04962708
H 0.8261668841 -2.1461934458 -1.5091829374
H 1.1499838325 -0.1737959871 0.0957724497
H 2.4289858498 -0.048855015 -2.014521984
H -1.2251832914 -0.4011385914 -0.601776950
H -1.6764350633 -1.3783251088 -2.540020514

IXb
C 0.0809466083 0.0667508933 0.1428072781
N -0.2044653504 -0.1717565938 1.5806092155
C 1.0615482471 -0.1740845871 2.3539769021
C 2.1005260064 0.4214341125 1.3950776575
C 1.6065737986 -0.0751006029 0.027992635
P -1.5382295692 0.584136152 2.3127023854
N -1.7525135951 -0.2834474727 3.737950324
C -1.6064654198 -1.7440643132 3.8590461132
C -1.0345350594 -1.9343686736 5.2706450855
C -1.7129921598 -0.7989604832 6.0533688071
C -1.6891434382 0.3762202737 5.0623086647
N -1.5239033689 2.0955082849 2.6946501599
P -0.7884625567 3.4236236708 1.9306798286
C -1.5534841443 4.7425111754 3.0173349618
C -1.1100886474 4.6630002742 4.4820578885
C -1.6618261873 5.7896116847 5.3739846082
C -3.1881875847 5.7687599864 5.5312790452
N -1.7143856794 3.6996241106 0.5040157285
P -1.153604535 4.1033680683 -0.9018006132
N -2.1147091027 5.3425098464 -1.5046948889
C -2.8857396207 6.2546120111 -0.6350928654
C -2.8078764122 7.5980853536 -1.3707660773
C -2.8175524388 7.1750361899 -2.8483608114
C -1.9534179187 5.8991588988 -2.8594446277
N 0.4342855919 4.6795244546 -1.0819712761
C 0.7253569177 6.0260366362 -0.5335409025
C 2.245767772 6.022004092 -0.3240014146
C 2.5266972318 4.5645464143 0.071660523
C 1.592326512 3.7775576384 -0.8553742254
N -1.1396440415 2.8283943312 -2.0029428578
C -2.2170401839 1.8244343911 -1.9856321703
C -1.7675222098 0.7605766509 -3.0263953901
C -0.436010834 1.2960287193 -3.6050243353
C -0.5154349012 2.8023788261 -3.3280834783
N -2.7384550528 0.211212593 1.1833054703
C -3.9645702171 1.0364024343 1.1542132167
C -4.8356963363 0.3755853803 0.0523052487
C -4.1208732047 -0.9555383312 -0.302678611
C -3.1303084479 -1.1623358835 0.852302724

H	-3.694912345	5.933885194	4.5735027343
H	-2.6416486613	4.6368711399	2.918463776
H	-3.5277560518	6.5468968844	6.2267091308
H	-0.766202888	0.9630635002	5.1599849287
H	-2.5259609884	1.0680501596	5.1964427299
H	-1.2085431284	-0.5555352561	6.9944910059
H	-2.7487576985	-1.0771587421	6.2886352811
H	0.0532223687	-1.7855914425	5.2556174767
H	-1.2374268617	-2.9305994196	5.6787552152
H	-0.9572328555	-2.1406111577	3.071867414
H	-2.5826604456	-2.2461576734	3.7731053669
H	-3.6338464023	-1.6545217651	1.7030834513
H	-2.256527599	-1.7631894527	0.5831425526
H	-3.5686096025	-0.8536631122	-1.24292318
H	-5.8507274995	0.1959522971	0.4225946166
H	-4.8130675918	-1.797701564	-0.4119314252
H	-4.921927989	1.0235973225	-0.825798913
H	-3.6963619399	2.0736094718	0.9449762779
H	-0.2344707427	1.0676175644	-0.168456085
H	2.0078915814	0.4959169526	-0.8165602705
H	1.8852202526	-1.1274823425	-0.1153043377
H	3.1238017676	0.1080735604	1.6299020515
H	1.3274969192	-1.2051654902	2.6332371257
H	0.964016152	0.4088119679	3.2755796488
H	2.0509846555	1.5160256349	1.4422350781
H	0.4527425942	3.305492975	-3.317680045
H	-1.1452159085	3.2941822938	-4.0899866279
H	0.4157057599	0.8750516395	-3.0577843072
H	-3.1806952999	2.2713660479	-2.2791054994
H	-0.3069154098	1.0650315	-4.6681462607
H	-1.6416779554	-0.2248050967	-2.566801632
H	-2.5204695501	0.6563055956	-3.8155456802
H	-3.9269929568	5.9111467228	-0.5489584836
H	-2.4688592589	6.2804508159	0.373763031
H	-1.864182378	8.1039399839	-1.1277954478
H	-3.6310237585	8.2717218284	-1.1090808332
H	-0.8979028626	6.1318843441	-3.0669640082
H	-2.2897212542	5.1892667985	-3.6243943021
H	-3.8424732319	6.9354320008	-3.1592253159
H	-2.4325786662	7.9427453594	-3.5281001065
H	0.3940892636	6.8059836975	-1.2286675
H	0.2118086783	6.189016423	0.4257730927
H	2.7577385371	6.2684333698	-1.2637217497
H	2.5597701906	6.7479481979	0.4333563287
H	2.2283017232	4.3918852107	1.1122986147
H	3.5771303453	4.2760427333	-0.0463907812
H	1.2755802176	2.8299278475	-0.4149926343
H	2.0850798468	3.5572967134	-1.8147794768
H	-0.4549983093	-0.6565958797	-0.48207229
H	-4.4836161697	1.0055848445	2.1255454603
H	-3.5262359985	4.7986548518	5.9195788494

H -1.4187992232 3.690600782 4.8892547594
H -0.0103170562 4.6831238396 4.529069401
H -1.3498992297 6.7627618814 4.9650098666
H -1.2000245697 5.7123597775 6.3695782346
H -1.2728170255 5.7167054619 2.5873497209
H -2.3281646251 1.4203673079 -0.9798543919

IXbH^+

C -0.1099316667 0.0430677347 -0.1833331694
N -0.1048587134 -0.1001673054 1.2935594002
C 1.2523545508 -0.4710651205 1.7756018461
C 2.1549502603 -0.2858612615 0.5477902396
C 1.2131074635 -0.5969373817 -0.6242999333
P -1.1109264965 0.851168626 2.229099608
N -0.9340660185 0.3087081724 3.7929782689
C -1.2366139638 -1.0927599352 4.17675184
C -0.4016129026 -1.3172122591 5.4472208572
C -0.3126071246 0.0898407309 6.0577893297
C -0.115586431 0.983396091 4.828214294
N -0.7785065123 2.3937719253 2.2171526482
P -1.2213361694 3.9135175944 1.9879832214
C -2.5637998533 4.4696328255 3.0987722394
C -2.4861264132 3.9370698958 4.5354645152
C -3.6446192385 4.4206818966 5.4255456686
C -5.0171104919 3.8791263344 5.004759414
N -1.6798727103 4.3962809081 0.5196328813
P -0.9083768525 4.4081970183 -0.8788823648
N -1.5242910755 5.7057781964 -1.7036981476
C -2.0839296404 6.9189212611 -1.0566379283
C -1.973458841 7.985961926 -2.1540149759
C -2.1454962984 7.1780748921 -3.4493506621
C -1.3640721138 5.8868322565 -3.1613674005
N 0.7582188986 4.5688780472 -0.8787841344
C 1.3588196793 5.9215351805 -0.7270378025
C 2.8216686647 5.6276802219 -0.3716202557
C 2.7192521292 4.3405056012 0.4602639682
C 1.6530373235 3.5343091492 -0.2927305335
N -1.1484641102 2.9940885298 -1.7199240972
C -2.4958353604 2.3684546408 -1.7275238701
C -2.6037941039 1.7472498753 -3.1256207075
C -1.1511664236 1.3649602861 -3.4485403722
C -0.3364794476 2.5351852217 -2.8750926158
N -2.6130078047 0.4929555771 1.6044017326
C -3.761912267 1.4192336847 1.7678198767
C -4.9929221725 0.5829372755 1.3574356406
C -4.4054252558 -0.5762836568 0.535625157
C -3.0942432389 -0.8654979223 1.2692431283
H -5.3158898815 4.2368749873 4.012230594
H -3.4991982419 4.1814047442 2.6091283188
H -5.7948270307 4.192339503 5.7098924432
H 0.9458827195 1.0174745501 4.5413282137
H -0.4510430758 2.010075173 4.9788170835

H	0.5007310716	0.1970913165	6.7817103721
H	-1.2531783271	0.3462605169	6.5614534368
H	0.6010919928	-1.6731240969	5.1794475606
H	-0.8534856041	-2.0561254618	6.1154765688
H	-0.9768158213	-1.7929525547	3.374552538
H	-2.3092160239	-1.1985629871	4.3859421628
H	-3.2864742699	-1.4603311679	2.1739444248
H	-2.3582651811	-1.4010190932	0.6664195807
H	-4.1936762445	-0.2547374694	-0.491789892
H	-5.4935069962	0.1882480114	2.2492342886
H	-5.0635493976	-1.4491831802	0.4918631814
H	-5.7251367737	1.1780653696	0.8038340292
H	-3.6248507657	2.2867335689	1.1133520734
H	-0.1562066352	1.0988527473	-0.4707765999
H	1.5647045281	-0.198282737	-1.5813385712
H	1.0924784638	-1.6813541739	-0.7361634805
H	3.0383845782	-0.9306668946	0.5785048243
H	1.2499919338	-1.5158922376	2.1137417847
H	1.5692108261	0.1521234319	2.6159037257
H	2.4967906476	0.7549763135	0.4904586949
H	0.6601357773	2.2240419634	-2.5497226578
H	-0.208310562	3.3300903465	-3.6193739259
H	-0.8837509202	0.4333036875	-2.9363490604
H	-3.2755584889	3.1109134924	-1.5313836966
H	-0.9663660475	1.222715896	-4.5173711966
H	-3.2905172411	0.8952419481	-3.1518139434
H	-2.9672901806	2.4936083347	-3.8427145782
H	-3.1262364255	6.7399810009	-0.7653023324
H	-1.5270240546	7.1821687775	-0.1529277162
H	-0.979150387	8.4497168677	-2.1287175149
H	-2.7181143518	8.7790663838	-2.0376043663
H	-0.3032546728	5.9941205752	-3.4348280673
H	-1.768410973	5.027787315	-3.70689294
H	-3.2050717235	6.9443828365	-3.6100447401
H	-1.7753876988	7.6962305619	-4.3388427863
H	1.249117241	6.4966183274	-1.6512767113
H	0.8745630147	6.4859426382	0.0832464987
H	3.4014795437	5.4427969236	-1.2842559276
H	3.2906408013	6.4578039696	0.1647503591
H	2.3693805442	4.5711077363	1.4747878389
H	3.6676834993	3.8010641551	0.5452138888
H	1.1052546133	2.8496598539	0.3602910275
H	2.1104556193	2.942868818	-1.0972385207
H	-0.9810396654	-0.4573133765	-0.618001493
H	-3.8503067041	1.778854065	2.7993045571
H	-5.0144348516	2.7811170693	4.9794229468
H	-2.483798617	2.8393067975	4.5161962677
H	-1.5324769544	4.2431303425	4.9875506528
H	-3.6653546348	5.5194031467	5.4346467826
H	-3.4381122691	4.1091112562	6.4578283945
H	-2.5346524817	5.5676725148	3.0835006769

H	-0.0932603715	4.6551813761	2.405623054
H	-2.5499375853	1.6058743806	-0.941379722
